# Time-dependent effects of rapid-acting antidepressants in iPSC-derived neurons from treatment-resistant depression and healthy volunteers

**DOI:** 10.1038/s41380-026-03768-0

**Published:** 2026-07-24

**Authors:** Jenessa N. Johnston, Gregory H. Jones, Shiyong Peng, Peixiong Yuan, Mani Yavi, Bashkim Kadriu, Ioline D. Henter, Brandi Quintanilla, Abdel Elkahloun, Ruin Moaddel, Anton Schulmann, Nirmala Akula, Mark D. Kvarta, Francis J. McMahon, Carlos A. Zarate

**Affiliations:** 1 Experimental Therapeutics and Pathophysiology Branch, National Institute of Mental Health, National Institutes of Health, Bethesda, MD USA; 2 Microarrays and Single-Cell Genomics Core, National Human Genome Research Institute, Bethesda, MD USA; 3 Laboratory of Clinical Investigation, National Institute on Aging, National Institutes of Health, Baltimore, MD USA; 4 Section on the Genetic Basis of Mood and Anxiety Disorders, National Institute of Mental Health, National Institutes of Health, Bethesda, MD USA

**Keywords:** Neuroscience, Biomarkers, Stem cells

## Abstract

Rapid-acting antidepressants like ketamine and serotonergic psychedelics show promise for treatment-resistant depression (TRD), but the molecular mechanisms that contribute to their therapeutic effects remain unclear. Induced pluripotent stem cells (iPSCs) offer a platform to model human cortical neurons and investigate drug effects in a human-relevant system. Here, iPSCs from individuals with TRD and healthy volunteers (HVs) were differentiated into mature cortical-like neurons and treated for six and 24 h with agents being investigated as rapid-acting antidepressants, including (2 *R*,6 *R*)-hydroxynorketamine (HNK), psilocybin, lysergic acid diethylamide (LSD), and 2,5-Dimethoxy-4-iodoamphetamine (DOI). Bulk and single-cell RNA sequencing assessed global and cell-type-specific transcriptomic responses. Synaptic proteins were evaluated via Western blotting and immunocytochemistry. To validate translational relevance, transcriptomic results were compared to CSF proteomics from ketamine-treated HVs. Despite differing initial pharmacological targets, overall gene expression across all compounds was highly correlated at matched timepoints compared to vehicle control, suggesting shared downstream effects. Both glutamatergic and serotonergic drugs converged on pathways involving inflammation, mTORC1 signaling, and cellular growth. At the single-cell level, (2 *R*,6 *R*)-HNK showed distinct cell-type specific alterations: upregulation in excitatory neurons and concomitant downregulation of inhibitory neuron populations. Differentially expressed genes from (2 *R*,6 *R*)-HNK-treated neurons also overlapped with CSF proteomic signatures from ketamine-treated individuals, supporting the model’s translational relevance. This study is the first to assess multiple putative rapid-acting antidepressants in parallel using an iPSC-derived neuron model. Both convergent and drug-specific changes in gene expression and pathway enrichment were observed across diverse compounds, supporting the use of human iPSC-derived neurons in antidepressant drug discovery. **Clinical Trial Registry**: www.clinical trials.gov, NCT02484456.

## Introduction

Approximately one-third of individuals with major depressive disorder (MDD) have treatment-resistant depression (TRD), defined as lack of response to two or more conventional antidepressants such as selective serotonin reuptake inhibitors (SSRIs) [1]. Novel, rapid-acting antidepressants are urgently needed, but developing therapeutic targets using animal models has proven challenging. Determining molecular neural mechanisms within human-derived systems may more effectively propel drug discovery efforts [2].

The development of induced pluripotent stem cell (iPSC) technology has opened new avenues for studying neuropsychiatric conditions using patient-derived cells [3, 4]. iPSC modeling allows the examination of genetic contributions to disease mechanisms and cell type-specific therapeutic responses [5], capturing individual genetic variation that animal models cannot fully assess. Studies using iPSCs have yielded insights into treatment response mechanisms in depression, including altered serotonergic signaling in SSRI non-responders [6, 7]. While this model provides unique access to human tissue, several inherent limitations should be acknowledged from the outset; specifically, iPSC-derived neurons are developmentally immature and may not fully reflect the adult human brain, glial cell types are not represented, and epigenetic signatures are partly erased during reprogramming [8]. Nevertheless, the ability of patient-derived models to enable direct study of the molecular mechanisms underlying therapeutic response remains valuable. The glutamatergic modulator ketamine has emerged as a promising rapid-acting antidepressant for TRD, improving symptoms within hours through increased α-amino-3-hydroxy-5-methyl-4-isoxazolepropionic acid receptor (AMPAR) transmission [9, 10]. (2 *R*,6 *R*)-hydroxynorketamine (HNK), a major ketamine metabolite essential to its antidepressant actions [10, 11], increases dendritogenesis and AMPAR transmission in rodent models [12]. iPSC studies of ketamine found increased neural progenitor proliferation via cAMP-IGF2 signaling [13] and enhanced structural plasticity via AMPAR-driven brain-derived neurotrophic factor (BDNF) and mechanistic target of rapamycin (mTOR) signaling [12], paralleling observations in animal models.

Serotonergic psychedelics (SPs) like psilocybin similarly demonstrate rapid and sustained antidepressant effects in TRD [14, 15]. Although they act on different primary receptors, psilocybin appears to converge with ketamine on common downstream mechanisms involving cortical glutamate release and synaptic plasticity via BDNF-mTOR signaling [16]. Other SPs, including lysergic acid diethylamide (LSD) and 2,5-Dimethoxy-4-iodoamphetamine (DOI), act through similar 5-HT2A receptor-dependent mechanisms [16, 17]. However, previous iPSC studies examined single agents only, and none incorporated cell lines from individuals with MDD or TRD.

Synaptic plasticity and glutamatergic signaling have been the most intensively studied mechanisms underlying rapid-acting antidepressant effects [18]. However, additional pathways—including neuroimmune and neuroendocrine signaling—likely also shape both their immediate and sustained therapeutic actions [19, 20]. Despite different primary receptor targets, ketamine and SPs appear to share clinical applicability across mood, anxiety, and substance use disorders, suggesting common transdiagnostic mechanisms [21]. Importantly, it is precisely this combination of divergent primary pharmacology and convergent clinical profiles that motivated the present study, as it raises the possibility that downstream circuit- and synapse-level adaptations may be critical drivers of their therapeutic effects. This study used a consistent experimental framework in neurons derived from both TRD patients and healthy volunteers (HVs) to evaluate and compare time-dependent molecular changes induced by (2 *R*,6 *R*)-HNK, psilocybin, LSD, and DOI. To our knowledge, this is the largest sample of iPSC-derived neurons assessed with these putative rapid-acting antidepressants. As an exploratory validation, transcriptomic signatures from (2 *R*,6 *R*)-HNK were compared to CSF proteomic changes after ketamine infusion in a previously examined group of HVs.

## Materials and methods

### Study participants

Peripheral blood mononuclear cells (PBMCs) were collected from five individuals with TRD enrolled in a crossover trial at the National Institutes of Health (NIH) Clinical Center (NCT02484456). Fibroblasts from five HVs were also collected for reprogramming following standard procurement procedures. All participants were female (age range=20-68 years) in order to conserve statistical power within a small sample size. Further demographic information can be found in Table S1. The study protocol was approved by the Institutional Review Board of the NIH (registration number 01-M-0254) and written informed consent was obtained from all participants prior to any study procedures. The study was conducted in accordance with NIH ethical guidelines and the Declaration of Helsinki (2000).

### Generation of iPSC-derived neurons

iPSCs were reprogrammed at the National Heart, Lung, and Blood Institute (NHLBI/NIH) iPSC Core Facility using non-integrating approaches. No clonal abnormalities were detected, and a normal karyotype was confirmed across all cell lines. iPSCs were generated and differentiated into cortical neurons using established non-integrating reprogramming and neural induction protocols. Further details can be found in the Supplementary Methods. At 10 weeks, neurons differentiated under this protocol form synapses and have spontaneous synaptic activity alongside other neuronal markers of maturity [22]. All cell lines expressed pluripotency markers and were consistently assessed to be mycoplasma-free. At 10 weeks, the maturity of cortical neurons was confirmed via immunocytochemical staining for MAP2, post-synaptic density protein 95 (PSD-95), and β-tubulin III. A CCK-8 cell counting kit was used to determine viability.

### Drug treatment

At 10 weeks of maturity, primary cortical neurons were treated for six or 24 h with acetonitrile vehicle (0.1%), (2 *R*,6 *R*)-HNK (1 uM), ketamine, LSD (10 uM), psilocybin (10 uM), or DOI (10 uM). All cell lines were exposed to each treatment and timepoint in replicate wells (*n* = 5/condition). A schematic of the experimental design appears in Figure S1. Justification of drug concentration and timepoints can be found in the Supplementary Methods. Expression of N-methyl-D-aspartate (NMDA) receptors and serotonin receptors was verified to determine whether the iPSC-derived forebrain neurons contained the canonical signalers for ketamine and psychedelic actions (Fig. S2).

### Bulk RNA sequencing

RNA was isolated from treated cells, sequenced with the NovaSeq XPlus platform, and analyzed for differential gene expression using standard pipelines (STAR, featureCounts, Dream). For bulk RNA-sequencing analyses, each cell line (HV = 5, TRD = 5) was exposed to all treatments (*n* = 2 wells/cell line/treatment). Thus, 100 samples were processed for RNA-sequencing (10 cell lines, five treatments, two wells/treatment). Given the difference in cell lineage (fibroblasts and PBMCs), diagnosis was treated as a random effect in data processing to infer conclusions regarding treatment effects across cell lines. Significant contributors to variance as determined by VariancePartition [23], such as sequencing batch and subject, were also included as random effects. After removing lowly expressed genes (defined as those with counts per million (CPM) ≤ 0.1 in fewer than five samples), 22,448 genes remained. Differential gene expression analysis was conducted with Dream, a linear mixed model that is a variation of limma/voom analysis for assessing drug-treatment effects [24]. Given the exploratory nature of the model analyses, a relatively liberal threshold was applied for defining differentially expressed genes (DEGs), using an uncorrected *p* < 0.01 and an absolute log2 fold change>1.5. All subsequent pathway-level analyses were conducted using false discovery rate (FDR)–corrected *p*-values.

Batch corrections were applied as appropriate. De-identified data and analysis codes supporting the findings of this study are shared within the Open Science Framework (osf.io).

### Single-cell RNA sequencing

After 24-hour treatment, cells were processed for scRNA-seq using the 10x Genomics platform, and data were processed with Cellranger and Seurat (V5) in R [25, 26]. Initial quality control involved filtering cells based on median absolute deviations (MADs) to remove outliers [27]. For mitochondrial percentage, an upper threshold was set to filter cells with high mitochondrial reads ( > 3 MAD). For genes and unique molecular identifiers (UMIs), cells were filtered that were either above or below three MADs. scRNA-seq was performed on neurons derived from one HV and one individual with TRD across matched treatment conditions. The HV donor contributed four samples (vehicle control, HNK, LSD, and psilocybin); the TRD donor contributed three samples (vehicle control, HNK, and psilocybin), as the TRD LSD sample was excluded due to a high proportion of low-complexity cells. After quality control, doublet removal, and cluster-level filtering, 26,641 cells were retained for downstream analysis. Per-sample sequencing metrics including mean reads per cell, median genes per cell, and mapping rates are provided in Tables S2 and S3. For the remaining samples, the data were then integrated across samples using Canonical Correlation Analysis (CCA). Two clusters were subsequently excluded from downstream analysis. The first contained (7-49) cells across conditions, preventing statistically meaningful comparisons. The second exhibited clear signs of being driven by technical rather than biological variation (i.e., elevated mitochondrial and ribosomal percentages coupled with reduced gene and UMI counts). Because more stringent global filtering parameters resulted in excessive removal of cells from biologically relevant clusters, targeted cluster removal was used to maintain data integrity for downstream analysis. Manual annotation of integrated cell clusters was performed; details are provided in the Supplementary Methods. To confirm that drug treatment did not significantly alter cell type composition, cell type proportions were compared across conditions using *propeller* [28], with donor treated as a random effect. Proportions were compared across the four treatment conditions of interest (vehicle control, HNK, LSD, and psilocybin), mirroring the analytical approach in Bulk RNA samples.

### Western blotting

Protein lysates were analyzed by Western blot for synaptic and signaling proteins, with normalization to total protein (Ponceau); this method was used because typical housekeeping genes could potentially be expressed at different levels within TRD and HV cell lines. Prior to Western blotting, cells were homogenized in cold lysis buffer. Ten μg of protein were electrophoretically resolved in Mini-PROTEAN® TGX Precast 4-12% gels (BioRad, Hercules, CA) then transferred onto nitrocellulose membranes via a semi-dry transfer method in the Trans-Blot Turbo Transfer System (BioRad, Hercules, CA). Further details are provided in the Supplementary Methods.

### Immunocytochemistry

Fixed neuronal cultures were stained for synaptic and neuronal markers, including synaptophysin, PSD-95, and MAP2. Statistical analyses were conducted in RStudio. A negative binomial regression model was conducted to examine the effects of treatment and timepoint on synapse count, controlling for diagnosis and subject as random effects. The dispersion parameter (3.11) suggested overdispersion, justifying the use of negative binomial regression over Poisson regression. Further details regarding imaging and processing can be found in the Supplementary Methods.

### Clinical comparison with CSF proteomics

DEGs from in vitro experiments were compared to CSF proteomics data from HVs post-ketamine infusion. HVs (mean age=27 ± 6; F:M 4:5) were recruited in a separate study (NCT03065335) and had serial CSF collection after ketamine infusion. Details of the previous study can be found in [29].

Proteomics were performed using the Olink Explore platform (Inflammation I, Oncology I, Cardiometabolic I, and Neurology I reagent kits). Because the primary intention was to identify overlapping iPSC targets that correspond to the “ground-truth” (i.e., human CSF post-ketamine), and because of limited assay coverage of DEGs in the Olink platform (see Discussion), differential CSF protein expression was FDR-corrected for significance, while DEGs from the iPSC conditions were kept uncorrected (*p* < 0.05). To determine whether there was significant overlap between the patterns of differential CSF protein and iPSC gene expression over time, 22,810 genes tested for differential expression and the 1461 proteins that are able to be assessed by the Olink Explore platform (see Supplementary Table S5 in [29]) were used as background (*n* = 1235 overlapping analytes).

## Results

### Bulk RNA sequencing revealed overlapping and time-dependent effects of drug treatment

Assessing the neural molecular mechanisms of novel rapid-acting antidepressants such as (2 *R*,6 *R*)-HNK and SPs is a clear priority. To determine time-dependent effects, these therapeutics were applied to iPSC-derived cortical neurons from participants with TRD and HVs. Neurons were collected at six and 24 h after treatment. Each agent was first compared to vehicle-control treated iPSC-derived neurons to determine time-dependent drug effects. In addition to differences from vehicle-control treated neurons, similarities and differences in drug treatment effects were categorized to ascertain overlapping and distinct mechanisms between drug types (glutamatergic and serotonergic). Expression of canonical ketamine/(2 *R*,6 *R*)-HNK and SP receptors was verified (Fig. S2).

Differential expression analysis revealed significant alterations in gene expression across time and treatment groups (Fig. S3A). At six hours, 718 DEGs were observed for (2 *R*,6 *R*)-HNK-treated neurons, 246 DEGs were observed for psilocybin, 299 DEGs were observed for LSD, and 493 DEGs were observed for DOI. A higher number of DEGs were observed at 24 h ((2 *R*,6 *R*)-HNK = 790; psilocybin=1419; LSD = 1822; DOI = 389). Because disparate and overlapping mechanisms between putative rapid-acting antidepressants are of great interest for future drug development, common DEGs between drug treatment groups were also assessed. As expected, the canonical SPs (psilocybin and LSD) had the most DEGs in common, with over 22.6% overlap at 24 h (hypergeometric *p* < 2.2e-16, OR = 7.48).

A highly correlated directionality of log-fold changes between drugs at six and 24 h was found when comparing genes that survived the *p* < 0.05 correction within any condition (*n* = 4325) (see Fig. S3B for Spearman correlation values). Correlations across different drugs within the same timepoints were also high, particularly at 24 h (r_s_ 0.74–0.83). Interestingly, correlations between the same drugs at the two different timepoints were negative, a reversal of transcriptomic effects that suggests response [30]. All DEG lists are presented in Tables S4-S11.

**Fig. 1:**
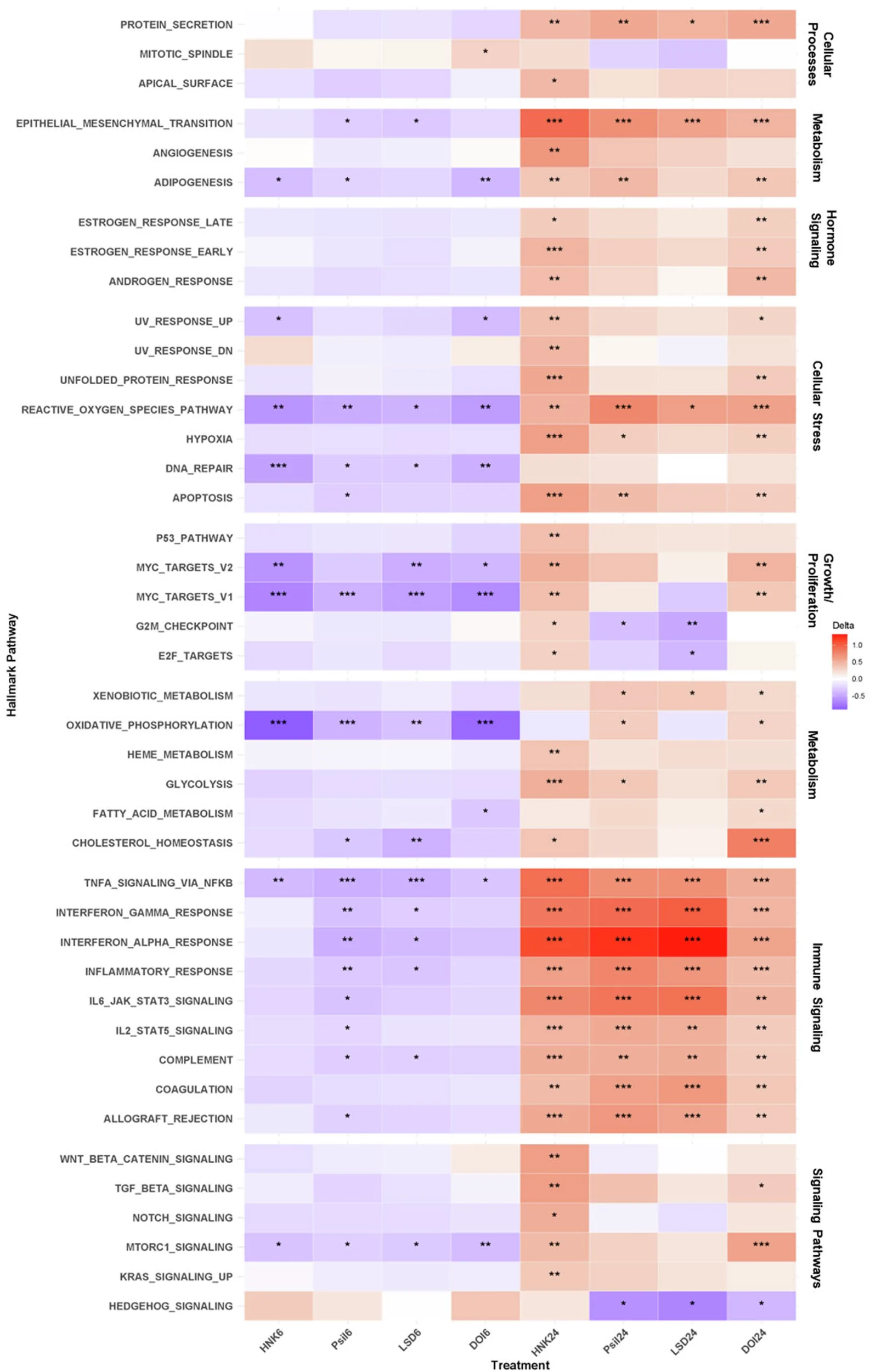
Most hallmark signaling pathways were downregulated at six hours and upregulated at 24 h after all drug treatments. Data is from bulk RNA-sequencing analysis. Color scale indicates delta scores. Blue represents downregulated pathways and red represents up-regulated pathways compared to vehicle-treated controls. *p < 0.05, **p < 0.01, ***p < 0.001 as false discovery rate (FDR)-corrected values.

Gene set enrichment analysis was conducted using the Molecular Signatures Database (MSigDB) as reference. Striking time-dependent effects were found in the activation of functional enrichment pathways. Downregulated signaling pathways at six hours were completely reversed at 24 h, demonstrating primarily upregulated signaling. Across all drug treatments at six hours, many hallmark signaling pathways were downregulated compared to vehicle control, including oxidative phosphorylation (*p* < 0.01 in all treatment groups) and tumor necrosis factor alpha (TNF-α) signaling via nuclear-factor kappa beta (NFκβ) (*p* < 0.05 in all treatment groups). At six hours, changes in hallmark signaling pathways were most similar between psilocybin and LSD, and significant decreases in cholesterol homeostasis and immunomodulatory pathways were absent after treatment with DOI or (2 *R*,6 *R*)-HNK (Fig. 1).

However, at 24 h, all drug effects were reversed; specifically, hallmark pathways involved in immune signaling, cellular metabolism, cellular stress, and canonical signaling were upregulated (Fig. 1). (2 *R*,6 *R*)-HNK had the most widespread impact across pathways, significantly upregulating 36 of 50 MSigDB hallmark signaling pathways at 24 h. Interferon gamma and alpha response, interleukin-6 (IL6)-Jak-STAT3, IL2-STAT5, allograft rejection, and complement and coagulation pathways were upregulated at 24 h across all treatments. Mechanistic target of rapamycin complex 1 (mTORC1) signaling, often associated with rapid-acting antidepressant drug response, was decreased across all treatments at six hours but significantly upregulated at 24 h by (2 *R*,6 *R*)-HNK and DOI (Fig. 1). Notably, hedgehog signaling was the only pathway significantly downregulated at 24 h after all SP treatments. All FDR corrected *p*-values can be found in Table S12. Non-specific cellular stress could, in principle, contribute to the observed time-dependent differences; however, no significant between-group differences were detected in mitochondrial gene expression, and most DEGs involved synaptic processes, suggesting that metabolic failure is unlikely to be the primary driver of these effects.

Diagnosis was included as a random effect in all analyses given the difference in cell lineages between HV (fibroblast-derived) and TRD (PBMC-derived) samples; however, an exploratory analysis of diagnostic differences in hallmark signaling pathways was also conducted (see Fig. S4 and Supplementary Results). TRD cell lines had significantly lower expression of genes associated with growth and proliferation pathways, as well as signaling pathways such as Notch and Wnt/β-catenin.

### Single-cell RNA sequencing clustering and cell-type specific drug responses

Bulk RNA-seq provided insights into global transcriptional changes, but our iPSC-derived neurons comprised heterogeneous cell populations at various maturation stages. A comparative analysis of bulk and scRNA-sequencing data can be found in the Supplementary Results. Drug effects are known to be highly cell type- and maturation-dependent, especially for ketamine and 5-HT2A-targeting agents [31, 32]. Because changes in excitatory/inhibitory (E/I) balance also correlate with rapid antidepressant effects [33, 34], scRNA-seq with annotation focused on cell type (excitatory vs. inhibitory) and maturation stage (pseudotime) was used to identify selective drug targets.

**Fig. 2:**
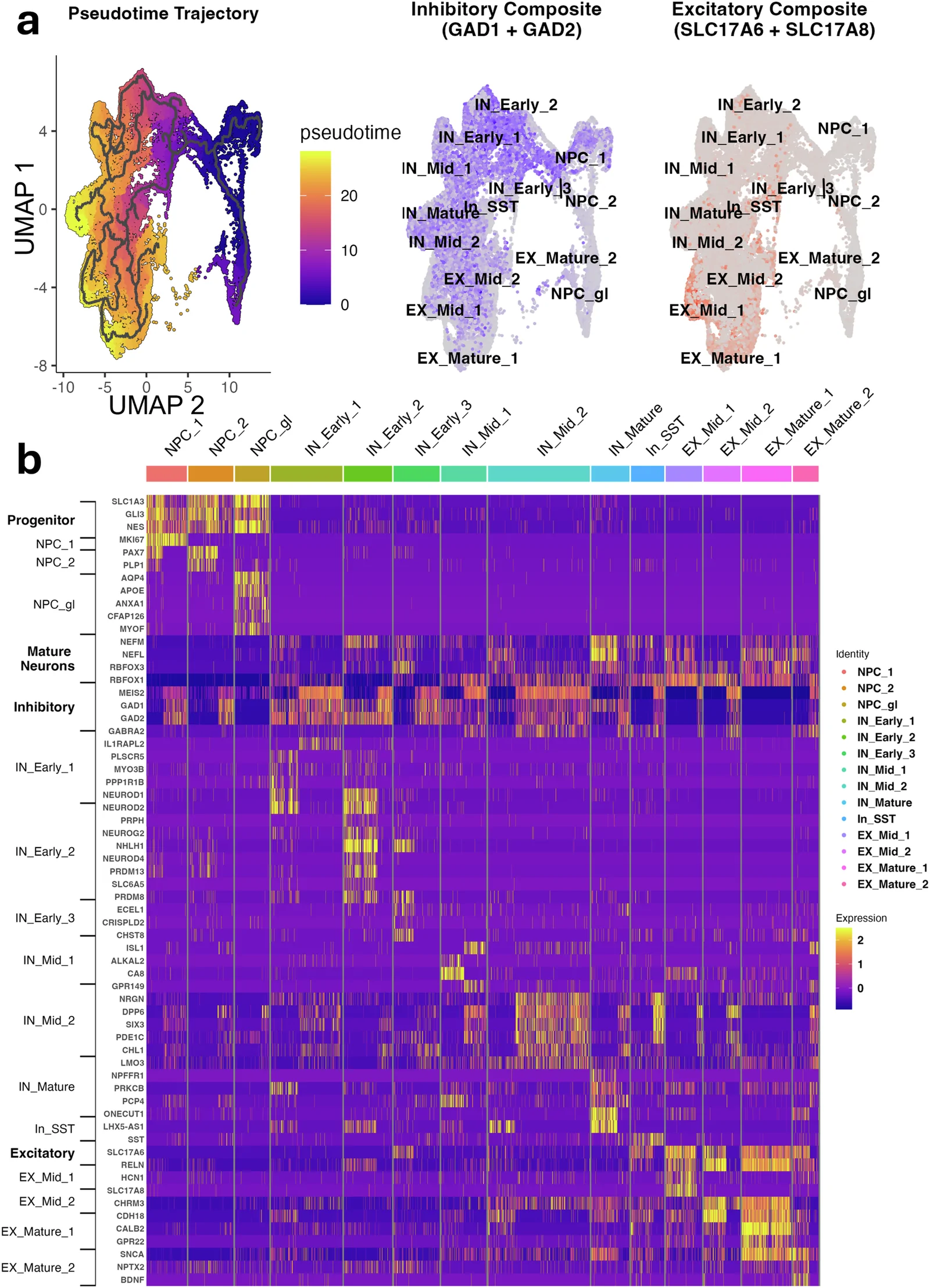
Single cell analysis of iPSC-derived neurons revealed distinct excitatory and inhibitory neuronal populations. (**A**) Uniform Manifold Approximation and Projection (UMAP) embeddings of single cell clusters showing differentiation stages across clusters (pseudotime) and distribution of inhibitory (left panel, GAD1 + GAD2 composite, blue) and excitatory (right panel, SLC17A6 + SLC17A8 composite, red) neuronal populations. The plot shows distinct separation of neural progenitor cells (NPCs), inhibitory neurons (INs), and excitatory neurons (EXs) at various maturation stages. Composite scores for canonical inhibitory/excitatory genes were generated using the AddModuleScore function in Seurat. Pseudotime was performed with Monocle3. (**B**) A heatmap of cell type-specific marker genes across identified clusters showed differential expression patterns of canonical neuronal markers and cluster-specific genes identified via cosine similarity from COSG in R. Ranked median pseudotime values per cluster were used to subdivide clusters into early, mid, and mature types within the excitatory and inhibitory populations. Notable markers included MKI67 for proliferative neural progenitors (NPC_1), AQP4/APOE for glial-fated progenitors (NPC_gl), GAD1/GAD2 for inhibitory neurons, and SLC17A6/SLC17A8 for excitatory neurons. The color gradient represents normalized expression levels from 0 (low, purple) to 2 (high, yellow).

iPSC-derived neurons were treated with vehicle, (2 *R*,6 *R*)-HNK, psilocybin, or LSD for 24 h before single-cell sequencing. After quality control and data integration, unsupervised clustering identified 13 cell populations, annotated using canonical marker genes and developmental trajectory analysis (Table S13). These clusters were grouped into neural progenitor cells (NPCs), inhibitory neurons (INs), and excitatory neurons (EXs), each further subdivided by maturation stage. Drug treatments were proportionally distributed across clusters, enabling assessment of drug effects by cell type and maturation (Fig. S5). To formally confirm this, *propeller* testing revealed no significant differences in cell type proportions across treatment conditions for any of the 14 identified clusters (all F-statistics<0.65, all FDRs>0.99; Table S14), indicating that observed transcriptional differences between conditions were not confounded by systematic shifts in cell type composition. Distinct populations of excitatory and inhibitory neurons were identified based on canonical marker expression. Inhibitory clusters showed strong *GAD1* and *GAD2* expression, while excitatory clusters expressed glutamate transporters *SLC17A6* and *SLC17A8* (Fig. 2A).

Additional annotation details, including conformation of cortical neuronal phenotype (Figs. S6,S7) appear in the Supplementary Results.

### Serotonergic psychedelics showed broad pathway modulation across mature neuronal populations

**Fig. 3:**
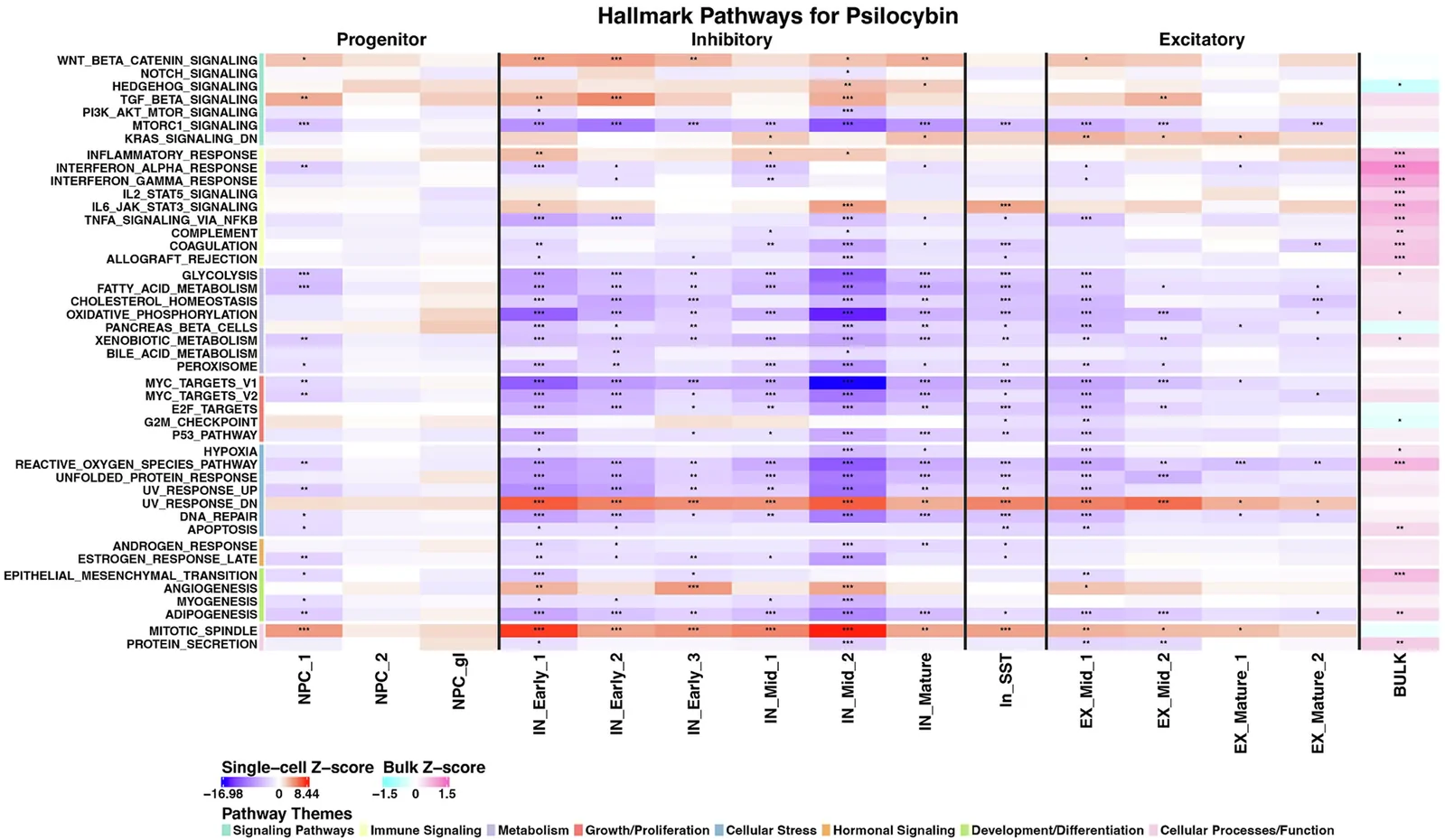
Cell-type specific pathway modulation by psilocybin at 24 h. Heatmap showing hallmark pathway enrichment across neural cell clusters after psilocybin treatment. Color intensity indicates combined z-score enrichment from model-based analysis of single-cell transcriptomics (MAST) bootstrapped gene set enrichment analysis (GSEA) (red = upregulation, blue = downregulation). For comparison, the final column (right) contains the bulk differential expression z-scores from zenith (pink = upregulation, cyan = downregulation). Differences in z-score color ranges between single-cell and bulk should be noted during interpretation. Psilocybin showed broader effects across multiple mature neuronal populations, with pronounced activity in inhibitory neurons. Note the significant downregulation of pancreas beta cells and E2F target pathways across multiple inhibitory clusters and strong upregulation of transforming growth factor-beta (TGF-beta) signaling, angiogenesis, and IL6-JAK-STAT3 signaling in IN_Early_1, IN_Mid_1, and IN_SST clusters. “UV response down” was consistently upregulated across most neuronal clusters. Asterisks indicate significance level: * p < 0.05, ** p < 0.01, *** p < 0.001.

**Fig. 4:**
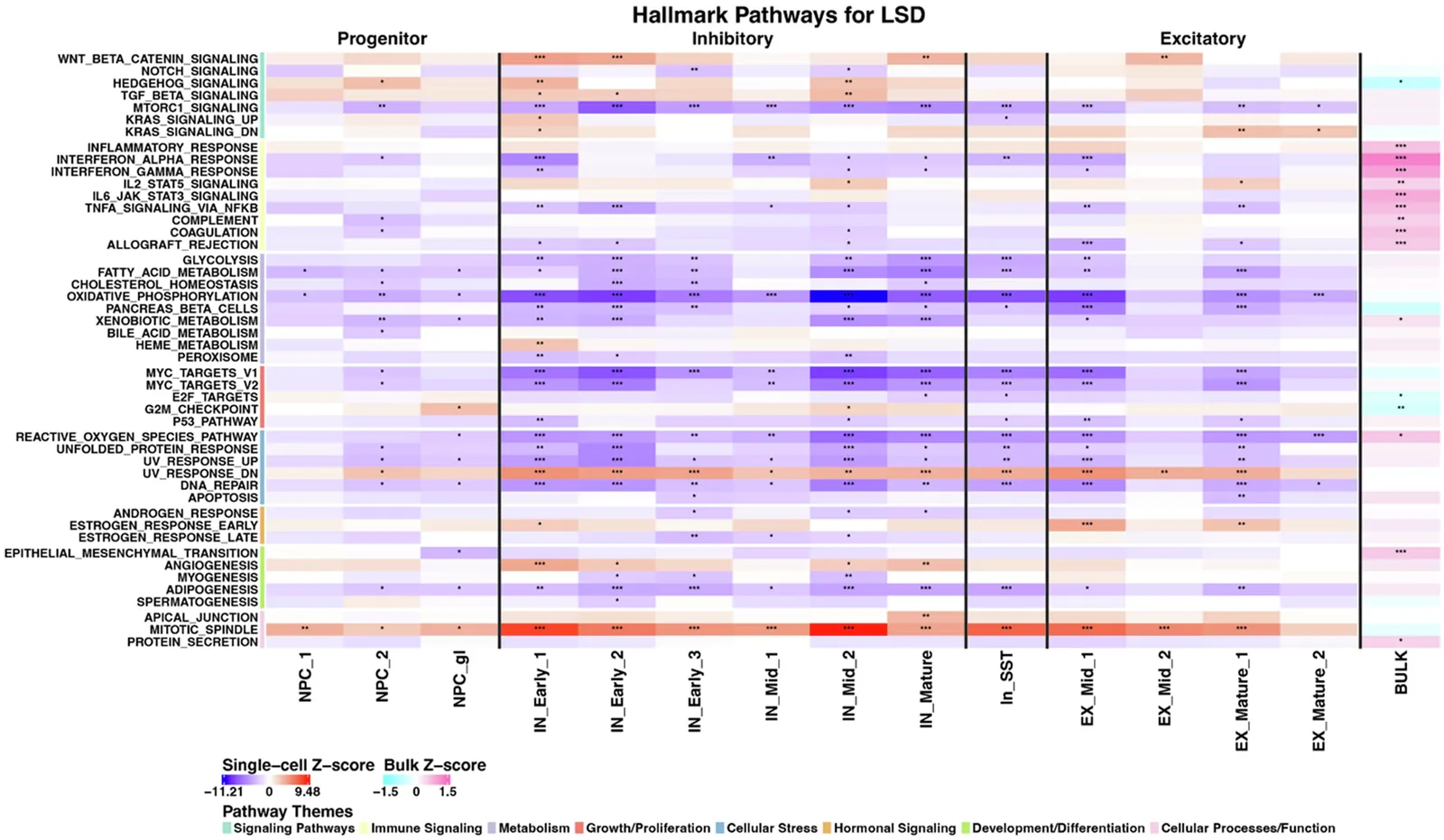
Cell-type specific pathway modulation by LSD at 24 h. Heatmap showing hallmark pathway enrichment across neural cell clusters after treatment with LSD. Color intensity indicates combined z-score enrichment from model-based analysis of single-cell transcriptomics (MAST) bootstrapped gene set enrichment analysis (GSEA) (red = upregulation, blue = downregulation). For comparison, the final column (right) contains the bulk differential expression z-scores from zenith (pink = upregulation, cyan = downregulation). Differences in z-score color ranges between single-cell and bulk should be noted during interpretation. Notable features include significant downregulation of oxidative phosphorylation, MYC targets, and hedgehog signaling across multiple inhibitory clusters, with upregulation of IL2-STAT5 signaling and “UV response down”. “Estrogen response late” was upregulated in IN_SST and several other mature neuronal clusters. Asterisks indicate significance level: *p < 0.05, **p < 0.01, ***p < 0.001.

Examination of hallmark pathway enrichment across cell clusters revealed that psilocybin and LSD exhibited remarkably similar pathway signatures (Figs. 3,4). Both SPs demonstrated significant pathway alterations predominantly in mature neuronal populations, with a slight bias towards inhibitory neurons. The most pronounced effects were observed in the IN_Mid_1, IN_Mid_2, and IN_Mature clusters. In the inhibitory populations, both psilocybin and LSD strongly upregulated pathways associated with cellular proliferation, including mitotic spindle (particularly pronounced in IN_Mid_2) and KRAS signaling. Concurrently, both drugs activated neuroprotective mechanisms by upregulating transforming growth factor beta (TGF-β) signaling across multiple inhibitory clusters. This pattern of enhanced proliferative and neuroprotective signaling may represent a core mechanism underlying the neuroplasticity-enhancing effects of SPs. Notably, both SPs significantly downregulated several metabolic pathways across inhibitory neurons, including glycolysis, fatty acid metabolism, and oxidative phosphorylation. This metabolic suppression was particularly evident in IN_Early_1, IN_Early_2, and IN_Early_3 clusters. The “pancreas beta cells” pathway was also consistently downregulated across multiple inhibitory clusters, suggesting altered energy metabolism regulation.

Inflammatory signaling pathways showed a complex pattern of regulation. Both psilocybin and LSD significantly downregulated TNFα signaling via NFκB, interferon alpha response, and interferon gamma response in inhibitory neurons (particularly IN_Early_1 and IN_Early_2). However, IL2-STAT5 signaling was upregulated for LSD (IN_Mid_2 and EX_Mature_1), and IL6-JAK-STAT3 signaling (IN_Mid_1, IN_SST) and inflammatory response (IN_Early_1, IN_Mid_1, IN_Mid_2) were upregulated for psilocybin, suggesting differential regulation of specific inflammatory pathways.

Cellular stress response pathways, including reactive oxygen species, unfolded protein response, and UV response, were predominantly downregulated across inhibitory clusters, suggesting that SPs may enhance neuronal resilience by reducing stress-related signaling. This effect was particularly pronounced in the IN_Early_1 and IN_Early_2 clusters. In excitatory neurons, both SPs showed less pronounced effects compared to inhibitory populations, though significant pathway alterations were observed in the EX_Mid_1 and EX_Mature_1 clusters. Notably, angiogenesis and epithelial-mesenchymal transition pathways were upregulated in EX_Mature_1 and EX_Mature_2, potentially reflecting enhanced structural plasticity mechanisms.

### (2 R,6 R)-HNK demonstrated highly specific cell-type targeting with bidirectional pathway regulation

**Fig. 5:**
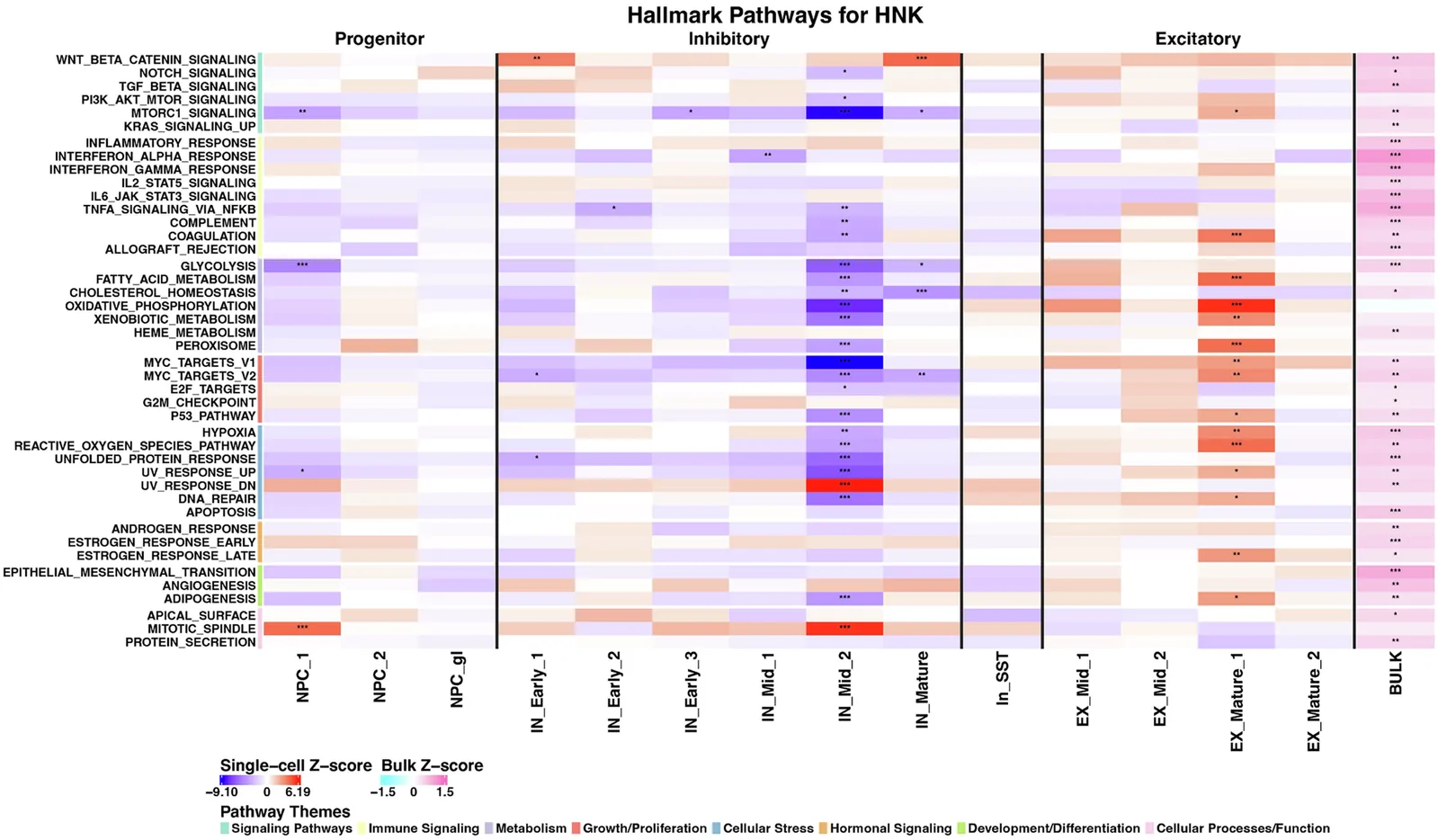
Cell-type specific pathway modulation by (2 *R*,6 *R*)-HNK at 24 h. Heatmap showing hallmark pathway enrichment across neural cell clusters after (2 *R*,6 *R*)-HNK treatment. Color intensity indicates combined z-score enrichment from model-based analysis of single-cell transcriptomics (MAST) bootstrapped gene set enrichment analysis (GSEA) (red = upregulation, blue = downregulation). For comparison, the final column (right) contains the bulk differential expression z-scores from zenith (pink = upregulation, cyan = downregulation). Differences in z-score color ranges between single-cell and bulk should be noted during interpretation. (2 *R*,6 *R*)-HNK had highly specific effects primarily in EX_Mature_1 and IN_Mid_2 clusters, with bidirectional regulation of metabolic and signaling pathways. Note the striking upregulation of oxidative phosphorylation, fatty acid metabolism, MYC targets, and mTORC1 signaling in EX_Mature_1, with concurrent downregulation of these same pathways in IN_Mid_2. Asterisks indicate significance level: *p < 0.05, **p < 0.01, ***p < 0.001.

In contrast to the broad effects of SPs, (2 *R*,6 *R*)-HNK exhibited high cell-type specificity, predominantly affecting only two neuronal populations: one inhibitory (IN_Mid_2) and one excitatory (EX_Mature_1) (Fig. 5). This targeted pattern of activity suggests a precise mechanism of action that may underlie its distinct therapeutic profile. (2 *R*,6 *R*)-HNK induced bidirectional regulation of several key pathways between these two clusters. Pathways governing metabolism, growth, and proliferation, including mTORC1 signaling, oxidative phosphorylation, fatty acid metabolism, glycolysis, and MYC target pathways (V1 and V2) were significantly upregulated in the excitatory EX_Mature_1 cluster and simultaneously downregulated in the inhibitory IN_Mid_2 cluster. Cellular stress pathways, including reactive oxygen species, UV response, and unfolded protein response were also regulated in opposite directions between these two clusters. The excitatory EX_Mature_1 cluster showed upregulation of these pathways, potentially reflecting increased metabolic activity, while the inhibitory IN_Mid_2 cluster showed downregulation, suggesting reduced cellular stress and metabolism. This mutual shifting mirrors the known mechanistic correlates of ketamine and its metabolites in humans [34] and may act to restore cellular balance to neuronal E/I.

Further KEGG pathway analysis of these (2 *R*,6 *R*)-HNK-responsive clusters revealed significant enrichment for “Morphine Addiction” and “Neuroactive Receptor-Ligand Interaction” pathways in both clusters, with the inhibitory cluster additionally showing enrichment for “Oxytocin Signaling”. These pathways appeared relatively specific to the (2 *R*,6 *R*)-HNK responsive clusters, with only two other cell types (IN_Early1, In_SST) showing similar pathway enrichment patterns (Figs. S8, S9).

Together, these findings suggest that while both SPs and glutamatergic modulators ultimately converge on increased synaptic plasticity, they appear to engage distinct cellular targets and molecular pathways to achieve these effects. The cell-type specificity of (2 *R*,6 *R*)-HNK suggests a highly targeted mechanism of action, while the broader activity of SPs across multiple cell types may explain their unique subjective and therapeutic profiles.

### Protein expression changes revealed similar time-dependent changes after drug exposure

Synaptic-specific and immediate early genes, such as PSD-95 and mTORC1, which have previously been implicated in putative rapid-acting antidepressant response [10], were measured through Western blotting to determine if time-dependent changes paralleled the bulk RNA-sequencing as well as previous research in animal models. Rapid-acting antidepressants like ketamine converge on mTORC1 and related synaptic signaling pathways, where they rapidly enhance synaptogenesis and synaptic protein expression in prefrontal circuits that are impaired in MDD. These rapid synaptic changes are a distinctive feature of rapid-acting agents and are not typically observed with conventional antidepressants, making mTORC1-centered synaptic signaling a particularly suitable focus for assessing novel rapid-acting antidepressants. In addition, the treatment- and time-dependent effects found on mTORC1 signaling within the bulk and single-cell data warranted further exploration of related protein signalers. All significant results are presented in Table S15 with Tukey Honestly Significant Difference (HSD) values for post-hoc analyses compared to vehicle control. Other significant comparisons between drug treatments, rather than comparison to vehicle, are noted in the text.

Total ERK, an indicator of cell survival, was affected by treatment and by the interaction of time and treatment, with (2 *R*,6 *R*)*-*HNK significantly increasing total ERK expression from vehicle and DOI at 24 h (Fig. S10A). Phospho-eukaryotic translation elongation factor 2 (eEF2) was significantly impacted by treatment and interaction effects. Tukey’s post-hoc tests revealed significant differences between (2 *R*,6 *R*)*-*HNK and LSD treatment at six hours (*p* = 0.031) as well as significant increases in phospho-eEF2 expression compared to vehicle after (2 *R*,6 *R*)*-*HNK treatment at 24 h (Fig. S10B). Similarly, total eEF2 expression was increased by (2 *R*,6 *R*)*-*HNK at 24 h (Fig. S10C). Total eIF4E expression was increased by treatment, whereas phospho-eIF4E expression had significant interaction effects. Post-hoc testing revealed that (2 *R*,6 *R*)*-*HNK increased levels from vehicle control at 24 h for both phospho-eIF4E and total eIF4E (Figs. S10D,S10E).

Total 4EBP1 was upregulated by LSD compared to (2 *R*,6 *R*)-HNK at six hours (*p* = 0.016) (Fig. S10F). PSD-95 was similarly affected, with LSD increasing PSD-95 expression from (2 *R*,6 *R*)*-*HNK at six hours (*p* = 0.047) and (2 *R*,6 *R*)*-*HNK increasing PSD-95 levels from both vehicle control and DOI (*p* < 0.001) at 24 h (Fig. S10G). Synaptotagmin was increased at 24 h by (2 *R*,6 *R*)-HNK compared to DOI (*p* = 0.001) (Fig. S10H). While a significant interaction between time and treatment was noted for Synapsin I, post-hoc testing revealed no specific differences. No significant differences were observed in levels of Dab1, phospho-ERK, GluA1, phospho-mTOR, mTOR, NR2B, phospho-4EBP1, phospho-TrkB, or TrkB.

### Synaptic counts were increased after drug treatment at 24 h

Given the increase in synaptic-specific proteins and transcriptomic changes in mTORC1 signaling and protein secretion, immunocytochemistry was conducted to ascertain histological changes after drug treatment. Immunofluorescent co-expression of PSD-95, MAP2, and synaptophysin was used as a proxy measure to assess synapse count on neurons. The negative binomial regression model demonstrated good fit and all fixed effects were statistically significant (*p* < .001). The model also revealed significant treatment × timepoint interactions (*p* < .001), indicating that treatment effects varied across timepoints. At six h, LSD and psilocybin significantly decreased synapse count compared to vehicle (*p* < .001). At 24 h, all treatments significantly increased synapse count compared to vehicle (*p* < 0.001), with psilocybin showing the largest effect (b = 1.63, *p* < .001) and LSD the smallest (b = 0.34, *p* < .001) (Fig. S11).

### Changes in iPSC-derived neurons mirrored those of CSF proteomics after ketamine treatment

The relative novelty of iPSC-based modeling for depression raises a pertinent question of comparability to living human neural processes. Preliminary evidence from our laboratory and others previously suggested that (2 *R*,6 *R*)-HNK’s NMDA-independent effects may drive ketamine’s longer-term antidepressant activity [11, 35]. Here, iPSC-based genetic responses to proteomic data were assessed from CSF collected over 24 h (six timepoints) after a racemic ketamine infusion (0.5 mg/kg over 40 min) in a previous study of nine HVs [29]. The present exploratory analysis sought to identify overlapping DEGs (uncorrected *p* < 0.05) between the (2 *R*,6 *R*)-HNK-treated iPSC neurons and the serial CSF proteomic data.

**Fig. 6:**
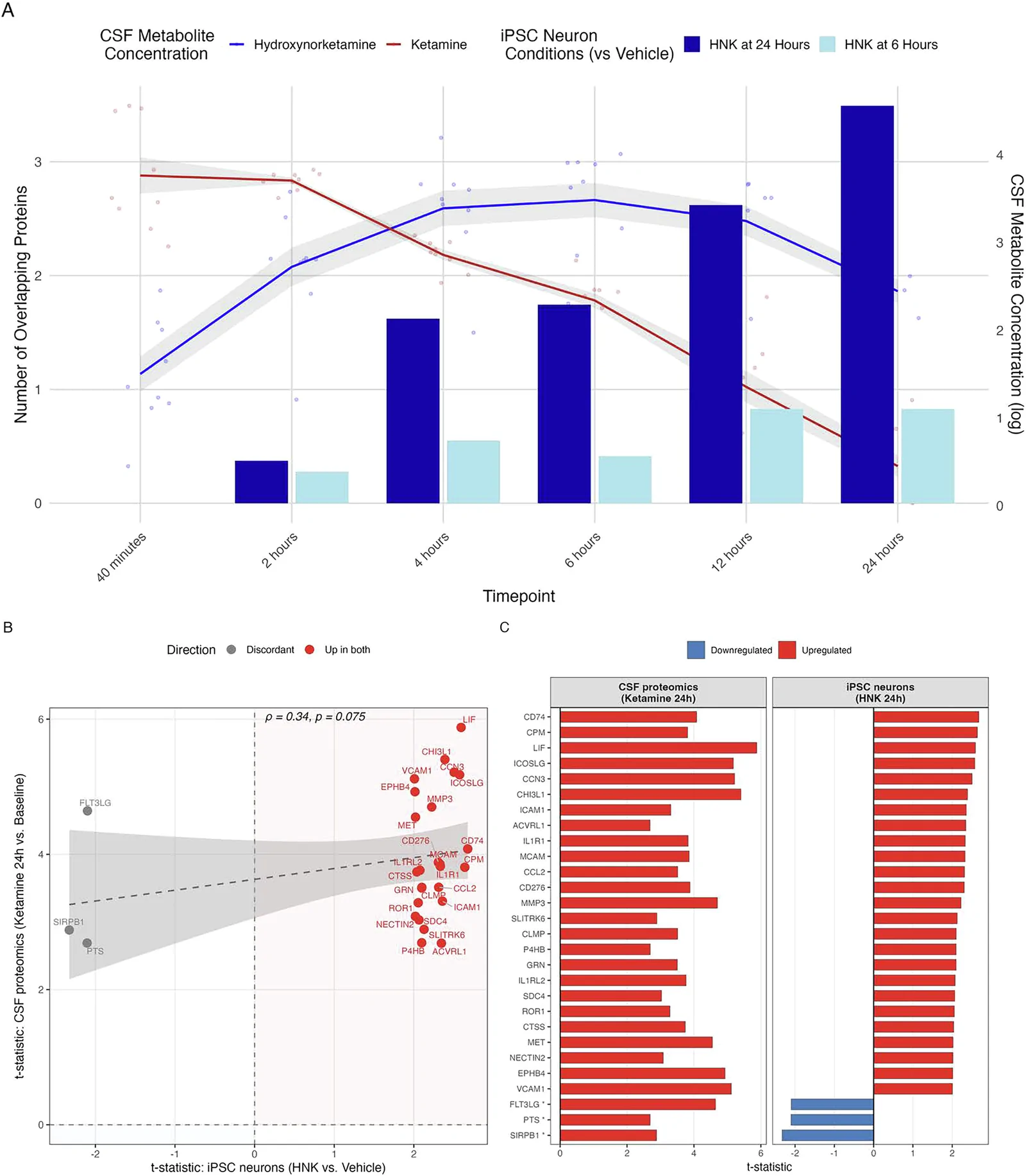
Induced pluripotent stem cell (iPSC)-derived neuronal gene expression after (2 *R*,6 *R*)-hydroxynorketamine (HNK) treatment mirrors cerebrospinal fluid (CSF) proteomic signatures following ketamine infusion. (**A**) Number of overlapping differentially expressed genes (DEGs) from (2 *R*,6 *R*)-HNK-treated iPSC neurons (uncorrected p < 0.05; dark blue: 24 h, light blue: 6 h) and differentially expressed CSF proteins at each serial timepoint following IV ketamine infusion (false discovery rate (FDR) < 0.05), overlaid with mean ( ± SE) log-transformed CSF concentrations of (2 *R*,6 *R*)-hydroxynorketamine (blue) and ketamine (red) across nine healthy volunteers (HVs). Overlap peaks at 24 h, coinciding with maximal (2 *R*,6 *R*)-HNK concentration in CSF. (B) Scatter plot of *t*-statistics for 25 of *t*he 28 directionally concordant overlapping genes/proteins at the 24-hour timepoint. X-axis: *t*-statistic from iPSC neuron bulk RNA-seq (HNK vs. vehicle); y-axis: *t*-statistic from CSF proteomics (ketamine 24 h vs. baseline). Shading indicates concordant quadrants (red: upregulated in both; blue: downregulated in both). Spearman’s ρ = 0.34, p = 0.075. Three discordant genes are shown in grey. (C) *t*-statistics for all 28 overlapping genes displayed side-by-side across both datasets. Red bars indicate upregulation, blue bars indicate downregulation. Twenty-five of 28 genes showed consistent change in direction across datasets (permutation p = 1.3e-5).

As shown in Fig. 6A, differential gene expression after six-hour (2 *R*,6 *R*)-HNK exposure in neurons demonstrated minimal overlap or discernible patterns with CSF protein expression. In contrast, gene expression associated with (2 *R*,6 *R*)-HNK at 24 h aligned progressively with CSF protein profiles, peaking at the equivalent 24-hour timepoint, which coincided with the observed maximal antidepressant effects that follow a single ketamine infusion [9]. Given this trend, a hypergeometric test was conducted to assess significant enrichment between the DEGs from (2 *R*,6 *R*)-HNK-treated iPSC neurons (24-hour condition) and differentially expressed proteins from the matched 24-hour CSF draw. Seventy-two iPSC-derived neuronal genes and 364 CSF proteins were differentially expressed, and 28 of them overlapped, indicating a nominally significant enrichment (permutation test: *p* = 0.043). Twenty-five of 28 were differentially expressed in the same direction in both datasets (Figs. 6B,6C), far above what would be expected by chance after factoring in the proportion of u*p*- and down-regulated genes in the background for both datasets (P_(X > 0.52)_ = 1.3e-5). Spearman’s rank correlations revealed a nonsignificant but trending relationship for the 28 overlapping genes/proteins at the 24-hour CSF draw (ρ = 0.34, *p* = 0.07; FDR = 0.099) that was stronger for the 21 overlapping genes/proteins at 12 h (ρ = 0.61, *p* = 0.004; FDR = 0.02), corresponding to peak (2 *R*,6 *R*)-HNK concentration in CSF (Fig. 6A). Consistent with overall trends in the bulk RNAseq pathway analysis, inflammatory cytokine signaling was significantly enriched in the KEGG pathway analysis.

The same analysis was repeated for each cell type cluster of the single cell data. DEGs were assessed after correction (p_fdr_<0.05). No individual cell types showed significant enrichment after correction (all *p* > 0.05). Ranked correlation for expression levels revealed a modest inverse relationship (ρ = -0.25; p_fdr_ = 0.073) for the EX_Mature_1 cluster (*n* = 124 genes/proteins). KEGG enrichment for this cluster returned one term: “Cell Adhesion Molecules” (*n* = 15 genes; p_fdr_ = 0.015). Four differentially expressed genes/proteins were common to all three datasets: CD276 (*B7-H3*), CD112 (*NECTIN2*), P4HB (*PDIA1*), and GRN (progranulin). Most DEGs ( > 90%) overlapping with CSF were upregulated for our single cell data across clusters. STRING analysis was then performed for protein-protein interactions on the group of upregulated genes across all single-cell clusters that showed directional agreement with differentially expressed CSF proteins at the 24-hour timepoint (*n* = 102 proteins/genes). Using STRING’s in-built gene ontology enrichment, the top three molecular processes identified were insulin-like growth factor receptor activity (GO:0005010), insulin-like growth factor binding (GO:0005520), and neurotrophin receptor activity (GO:0005030) (all FDR < 0.05) (Fig. S12).

## Discussion

This study is the first to report the effects of (2 *R*,6 *R*)-HNK and SPs in an iPSC-based neuronal model system. The significant positive correlations of gene expression changes across all drugs—but not between time conditions—suggests overlapping downstream mechanisms previously hypothesized to promote rapid antidepressant effects [36].

Bulk RNA-sequencing revealed large time-dependent effects across all treatments, with hallmark signaling pathways generally downregulated at six hours and u*p*-regulated at 24 h. Immune signaling (TNF-α via NF-κβ, interferon gamma/alpha response, IL6-Jak-STAT3, complement pathways) was upregulated across treatments at 24 h. Interestingly, while peripheral inflammation is elevated in depression [37], the effects of ketamine and SPs on these systems appeared mixed [38]. Another study found increased proinflammatory signals in the CSF 24 h post-ketamine infusion in HVs [29], paralleling our iPSC findings. Classic inflammatory signaling pathways such as IL-6-STAT3 may flip to neuroprotective phenotypes in the CNS, in contrast with their effects in the periphery [39, 40].

mTORC1 signaling was significantly downregulated at six hours across all treatments but upregulated at 24 h by (2 *R*,6 *R*)-HNK and DOI, coinciding with increased synapse number and cellular outgrowth markers. This complex temporal pattern warrants further exploration in human iPSC models—particularly given paradoxical findings that the mTOR inhibitor rapamycin prolonged ketamine’s antidepressant effects in a two-week follow-up [41]. Extending ketamine’s clinical effects by administering rapamycin suggests that dampening mTORC1 signaling may help modulate neuroinflammation and stabilize synapses newly formed after the administration of rapid-acting antidepressants. The upregulation of mTORC1 at 24 h, the increases in synapse number and other indicators of cellular outgrowth, and the increased inflammation observed here further align with mTORC1’s known promotion of inflammation. The complex role of mTORC1 warrants further exploration in in vitro models such as this, which allow for neuronal cell resolution in participants. In addition, the preliminary exploration of diagnostic differences revealed similar time-dependent effects as well as diagnosis-dependent effects on inflammatory and proliferative signaling that should be assessed further without confounding cell lineages.

At the single-cell level, drug effects predominantly occurred in mature neuronal clusters, suggesting that differentiation time is critical for future iPSC studies of these compounds. Psilocybin and LSD showed substantial transcriptional overlap, consistent with their mechanistic similarities [42]. Both agents upregulated inflammatory signaling (IL6-Jak-STAT3 (LSD), IL2-STAT5 (psilocybin), and TGF-β (both)), suggesting compensatory pro- and anti-inflammatory responses.

Notably, TGF-β signaling may serve as an instructive signal for neurons to switch from a “growth state” to a “synaptogenic state” [43, 44]—in line with downregulation of insulin-responsive genes (i.e., “hallmark pancreas beta cells”) with both SPs. In contrast to SPs, insulin signaling upregulation is a hallmark of treatment with ketamine and (2 *R*,6 *R*)-HNK [45–47]. For instance, STRING analysis showed enrichment for insulin-like growth factor receptor activity with (2 *R*,6 *R*)-HNK, and CSF insulin levels rose three- to four-fold at 12-24 h post-ketamine [29]. This divergence suggests that while ketamine and SPs may achieve similarly rapid antidepressant effects, they may have distinct metabolic/energetic profiles. Whether this is related to why SPs impart a more durable clinical response than ketamine—even when psychotherapy is minimized [48, 49]—remains a testable and potentially impactful hypothesis going forward.

(2 *R*,6 *R*)-HNK activity was restricted to two cell clusters, with metabolic pathways (mTOR, OXPHOS, β-oxidation) downregulated in IN_Mid_2 and upregulated in EX_Mature_1. These clusters were enriched for neuroactive ligand-receptor interactions, opioid, and oxytocin signaling—all established ketamine mechanisms [38, 50, 51]. The pattern of expression also fits the E/I balance hypothesis well [52]. While such findings should not be over-interpreted in the context of our limited sample size, this pattern fits with both ketamine’s canonical plasticity-inducing and metabolic signals [38, 53], as well as with the E/I balance hypothesis underlying its most robust clinical biomarker, namely increased magnetoencephalographic γ-power [52].

Finally, Western blotting confirmed time-dependent changes in synaptic proteins (PSD-95) and increased phospho-eEF2, total eEF2, eIF4E, and phospho-eIF4E by (2 *R*,6 *R*)-HNK, implicating protein synthesis upregulation and translation mechanisms that parallel ketamine’s [54, 55]. Given the enrichment found within the bulk RNA-sequencing, it would be valuable to explore protein levels of inflammation-related signaling; however, technical limitations prevented assessment for the current study. Immunocytochemistry confirmed increased synapse counts after all treatments at 24 h, supporting the hypothesis that cortical synapse increases are crucial for rapid-acting antidepressant effects [56].

Despite these intriguing findings, several limitations warrant attention. First, different starting lineages prevented diagnostic comparisons, though iPSC lines from the same donor were highly similar regardless of source cell type [57]. Second, the nature of reprogramming somatic cells to iPSCs strips away epigenetic effects that may deeply impact the molecular pathology of TRD and the effects of various treatments. Future research should attempt to identify and mirror common epigenetic changes within TRD. Third, practical constraints prevented us from generating single-cell RNA sequencing data for every cell line, precluding robust deconvolution of the bulk RNA-seq data to more fully account for the potential effects of cell-type composition. Cell collection at six and 24 h after drug treatment primarily captures early transcriptional responses, and future work should investigate how these drug-induced changes stabilize, evolve, or dissipate over longer timescales. Fourth, psilocybin was used rather than psilocin, though psilocybin is rapidly converted via alkaline phosphatase (*ALPL*) [58], which is an iPSC marker gene and was thus highly expressed in our sample (68^th^ percentile of all expressed genes by mean CPM). While no direct evidence of in vitro psilocybin metabolism was observed in our cell lines, the dephosphorylation of psilocybin has been observed within the human brain and across species, even those that do not express canonical enzymes such as *ALPL* [59]. Nevertheless, results from the psilocybin condition should be interpreted with caution. Bulk RNA-sequencing and scRNA-sequencing results diverged, likely reflecting both technical factors (including modest single-cell sample size) and the fact that these approaches capture different aspects of the transcriptomic response. Bulk RNA-seq measures a composition-weighted average across all cells that can dilute or mask changes confined to specific lineages, whereas scRNA-seq reveals robust, cell-type-specific programs, suggesting that particular neuronal subpopulations may drive a disproportionate share of psychedelic-induced transcriptional changes. The exploratory comparison between iPSC-derived neuronal gene expression and CSF proteomics should be interpreted with caution given several cross-platform limitations. These datasets differ with regard to biological substrate (transcriptomics vs. proteomics), measurement platform (bulk RNA-seq vs. Olink proximity extension assay), and study cohort (TRD patients and HVs vs. HVs only), precluding formal statistical harmonization across modalities. The Olink platform’s limited gene coverage (1461 proteins) further constrains the detectable overlap, and the modest Spearman correlation (ρ = 0.34, *p* = 0.075) at 24 h did not survive FDR correction, though directional agreement among the 28 overlapping analytes was robust (25/28; *p* = 1.3e-5) and the correlation was significant at the 12-hour timepoint (ρ = 0.61, *p* = 0.004), coinciding with peak (2 *R*,6 *R*)-HNK concentration in CSF. Rank-based statistics were used throughout to provide a platform-agnostic analytical framework. Taken together, these findings are best regarded as hypothesis-generating, and replication in larger, matched cohorts with concurrent transcriptomic and proteomic profiling are needed to establish the translational validity of this iPSC model system.

In sum, this study is the first comprehensive comparison of ketamine, (2 *R*,6 *R*)-HNK, and SPs in iPSC-derived neurons from TRD participants and HVs. Cell-type specific and time-dependent changes revealed convergent downstream mechanisms despite different receptor targets, with increased synaptic protein expression and outgrowth at 24 h. Synaptic-specific protein expression and synaptic outgrowth were found at 24 h post-treatment. The significant time-dependent overlap found between CSF protein expression and gene expression in our iPSC-derived neurons after treatment suggests that this model may provide direct insight to drug effects in patients, providing a promising platform for developing future rapid-acting antidepressants.

## Supplementary information

Supplementary Methods and Results

Supplementary Tables

## Data Availability

De-identified data and analysis codes supporting the findings of this study are shared within the Open Science Framework (osf.io). Analysis code and differential expression data are available within Supplementary data tables and the Appendix.
