# Supplementary material for: Time-dependent effects of rapid-acting antidepressants in iPSC-derived neurons from treatment-resistant depression and healthy volunteers
Source: Mol Psychiatry. 2026 Jul 24;31(12):7157–69. doi: 10.1038/s41380-026-03768-0 (PMC13736488; doi:10.1038/s41380-026-03768-0)
Supplement: Supplementary file 1 [file 41380_2026_3768_MOESM1_ESM.docx]

*Johnston et al — Time-Dependent Effects of Rapid-Acting Antidepressants in iPSC-Derived Neurons from Treatment-Resistant Depression and Healthy Volunteers*

**SUPPLEMENT**

**Supplementary Methods**

*Generation of induced pluripotent stem cells (iPSC)-derived neurons*

The STEMdiff^TM^ Forebrain Neuron Differentiation Kit (Stem Cell Technologies, Canada) was used to generate a mixed population of excitatory and inhibitory forebrain-type neurons (FOXG1+) in a consistent and replicable manner. Neural differentiation was carried out by following the STEMdiff™ embryoid body protocol for 19 days, resulting in the generation of single-cell neural progenitor cells (NPCs). Neural induction was verified by visual inspection, and neural rosettes were manually selected. The NPCs were then seeded at a density of 1.5×10⁶ cells/cm² onto dishes coated with poly-L-ornithine (PLO) and laminin, with half of the cultures plated on poly-L-lysine-coated coverslips. Cells were maintained at 37°C in a humidified atmosphere with 5% CO₂ and cultured in BrainPhys™ Neuronal Medium supplemented with 1% N2 Supplement-A, 2% Neurocult™ SM1 Neuronal Supplement, 20 ng/ml glial cell line-derived neurotrophic factor (GDNF), 20 ng/ml brain-derived neurotrophic factor (BDNF), 1 mM dibutyryl-cAMP, and 200 nM ascorbic acid. The medium was replaced by half every two days, and the cultures were maintained under these conditions for 10 weeks to promote the differentiation and maturation of primary cortical neurons.

*Drug Treatment*

*In vitro* concentrations of (2*R*,6*R*)-hydroxynorketamine (HNK) and ketamine were chosen based on previous research demonstrating enhanced structural plasticity in iPSC-derived mesencephalic neurons from healthy volunteers (HVs) [1, 2]. With regard to the concentration of serotonergic psychedelics (SPs) across treatments, preclinical studies found effects relevant to structural plasticity for both lysergic acid diethylamide (LSD) and psilocybin at 10uM [3-5]. While this is considerably higher than concentrations in human pharmacokinetic studies [6], direct translation from plasma concentrations to *in vitro* assays is often imperfect. Because preliminary dose-response curve measures (ranging from 1uM – 10uM) within our cell cultures indicated a peak of treatment-response at 10uM, this concentration was selected going forward. In agreement with this model, multiple studies have found that higher cumulative psilocybin doses are significantly associated with superior antidepressant effects [7]. Taken together with the favorable safety profile at higher doses (including in terminally ill patients) [8-10], the 10uM *in vitro* concentration was chosen because it maintains a balance between cross-study comparison potential, clinical relevance, and signal detection for treatment effects. Timepoints were chosen based on prior research conducted in mixed primary cell cultures after ketamine treatment, which noted that ketamine’s effects appear within 30 minutes of infusion and peak at 24 hours [11]. Similar time-scales have been observed in iPSC-derived dopaminergic neurons [2].

*Cell cluster annotation*

Manual annotation of integrated cell clusters was performed with input from three different sources. First, top marker genes for each cluster were identified with the COSG package, using cosine similarity for marker detection (marker specificity penalization; μ=100, minimum expression in 10% of cells) [12]. Based on marker genes, clusters were then generally separated into three categories: (1) progenitor, (2) inhibitory, and (3) excitatory. Clearly distinct cell type clusters were then labeled by canonical marker genes. For progenitor cells, a single highly proliferative cluster (NPC_1 expressing KI67) and another with prominent glial markers (i.e., NPC_gl) were labeled as such. A cluster expressing high levels of intraneuronal markers (i.e., In_SST) was also separated from the excitatory/inhibitory (E/I) groups. Trajectory analysis was then performed with Monocole3 [13]. The remaining E/I cells were then subdivided based on median pseudotime within each cluster (i.e., early, mid, mature, and numerically increasing with pseudotime within each of those subgroups).

*Western blotting*

Technical replicates were conducted in triplicate across all samples. Membranes were blocked using 5% (w/v) milk for unphosphorylated proteins and 5% (w/v) BSA for phosphorylated proteins for one hour at room temperature. After measurement of phospho-mTOR (#2971S, CST), phospho-ERK (extracellular signal-regulated kinase) (#9101S, CST), phospho-eEF2 (eukaryotic translation elongation factor 2) (#2331, CST), phospho-4EBP1 (eukaryotic translation initiation factor 4E-binding protein 1) (#2855, CST), or phospho-eIF4E (eukaryotic translation initiation factor 4E) (#9741, CST), the same blot was stripped and analyzed for mTOR (#2972S, CST), ERK (#5376S, CST), eEF2 (#2332, CST), 4EBP1 (#9452, CST), or eIF4E (#9742, CST) to provide individual ratios of activity. Other proteins analyzed included postsynaptic density-95 protein (PSD95) (#2507S, CST), synapsin I (#6710, CST), GluA1 (#13185S, CST), disabled adaptor protein (Dab1) (#3328S, CST), TrkB (#4603, CST), and GluN2b (UC Davis). Downstream density analyses were pooled across diagnoses.

*Immunocytochemistry*

Each PFA-fixed neuronal culture was washed three times with 0.5 mL of PBS at room temperature. To neutralize residual formaldehyde, cultures were treated with either 10 mM ethanolamine solution in PBS or 0.1 M glycine in PBS for five minutes, followed by another series of PBS washes.

Blocking buffer (1% BSA) was added to each well and incubated for one hour at room temperature to prevent non-specific binding. Primary antibodies—rabbit anti-synaptophysin (1:500), mouse anti-PSD-95 (1:1000), and chicken anti-MAP2 (1:3000)—were diluted in blocking buffer and incubated overnight at 4°C for optimal binding. The following day, coverslips were washed three times with PBS for 10 minutes each, with gentle agitation. Fluorophore-conjugated secondary antibodies (goat anti-rabbit, goat anti-mouse, and goat anti-chicken) were diluted with REDDOT (1:2000) in blocking buffer. Each secondary antibody solution was applied to the corresponding coverslip and incubated for one hour at room temperature. After the incubation, the coverslips were washed three times with PBS and mounted using Precision Mounting-GTM for imaging.

All coverslips were imaged using the Stellaris P8 microscopy system using a 63x oil lens to ensure consistency. For each experimental condition (treatment × timepoint), five to seven independent iPSC cultures derived from seven different donors were analyzed. Within each culture, an average of 22 (range: 3-58) non-overlapping fields of view were acquired using systematic grid sampling selected to contain well-focused MAP2-positive dendrites and intact DAPI-stained nuclei. This yielded a total of 37,964 to 363,045 MAP2-positive neurons analyzed per condition, with an average of 1,053 neurons per field. Fields were excluded only if they showed imaging artifacts (out-of-focus planes, edge effects, or tissue damage visible during acquisition). All quantitative data were nested appropriately in the statistical model to account for donor-level and culture-level variability.

Image acquisition parameters for each fluorophore were maintained rigorously constant within each experiment. For every staining run, all images for a given antibody (MAP2, PSD-95, synaptophysin, DAPI) were acquired in a single imaging session using identical settings: laser power, detector gain/photomultiplier tube voltage, exposure time, optical zoom, pixel dimensions (X, Y, Z), and z-step interval. These parameters were determined on a representative sample at the beginning of each experiment, ensuring that signal intensity remained within the linear range of the detector, and were saved as an acquisition template that was applied to all slides for that experimental replicate. Verification that the physical pixel dimensions were correctly embedded in the metadata was done prior to analysis; when the Denoise Processing software (SNIR, NIMH Neuroscience Imaging core facility, NIH) stripped this information, the X, Y, and Z dimensions were manually corrected in Arivis Vision 4D (Pixel Size→Edit) to ensure accurate volumetric and length measurements. Quantification of neuronal structures and synapse distribution was performed using machine learning-assisted neuron tracing pipeline in Arivis Vision4D, with manual validation conducted as needed to ensure accuracy in the automated analysis. First, MAP2-positive neuronal processes were traced automatically in ZEISS Arivis Vision 4D using the "Neuron Trace finding" mode with fixed tubularity and seed filter thresholds. These linear traces were then converted to volumetric segments using the "Threshold-based Reconstructor" function, which transforms one-dimensional tracings into three-dimensional volumes. This function retains all traces connected to a neuron cell soma while filtering out unconnected neurites.

The converted dendritic volumes were imported into a Vision 4D analysis pipeline to serve as parent compartments using the "Object Math_ Nuclei_Intensity Threshold Segmenter" operation. Subsequently, synaptic puncta—representing the postsynaptic marker PSD-95 and the presynaptic marker synaptophysin—were detected as child objects with the Blob Finder algorithm using fixed parameters (diameter, probability threshold, and split sensitivity). To distinguish true synaptic puncta from non-specific background or artifacts, multiple filtering steps were implemented. The probability threshold within the Blob Finder was calibrated to exclude low-intensity noise while capturing dim but genuine puncta. This threshold was validated by visual inspection of overlay images, confirming that detected objects corresponded to punctate synaptic structures rather than diffuse background haze.

After initial blob detection, object feature filters were applied based on volume and mean intensity to exclude artifacts that were either too small (noise) or too large (aggregates or non-punctate staining). Cutoff values were determined empirically by clicking on known true puncta and artifacts in the Objects Table and examining their quantitative properties (volume, mean/max intensity).

Using the "Compartments" operation, only puncta that physically overlapped or were in close proximity to the MAP2-positive dendritic volumes were counted. This established a parent-child relationship, ensuring that reported synapse counts were specific to these dendrites and excluded any off-target or background signals. Linear density was calculated as puncta per micrometer of MAP2-positive dendrite for each marker individually (PSD-95-only, synaptophysin-only) and for colocalized PSD-95/synaptophysin puncta (our primary measure of functional synapses).

**Supplementary Results**

*Bulk RNA sequencing revealed diagnostic differences in response to drug treatment*

iPSC-derived forebrain neurons from participants with TRD exhibited significant downregulation of growth- and proliferation-related signaling pathways, as well as synaptic signaling cascades such as Wnt–β‑catenin, which has previously been implicated in depressive pathophysiology [14]. Strong diagnostic differences were also observed in response to treatment, though these results should be interpreted with caution given the cell lineage confound. Again, time-specific distinctions were found with both diagnoses, with limited changes observed at six hours. HVs had a surge in immune and inflammatory signaling pathways at 24 hours after all treatments (tumor necrosis alpha (TNF-α) signaling, interferon gamma and alpha response, inflammatory response, and IL6-JAK-STAT3, p<0.001), but a downregulation in growth and proliferation pathways after exposure to SPs (G2M checkpoint and E2F targets, p<0.001). Treatment-resistant depression (TRD) cell lines had the opposite result, with downregulation across immune signaling pathways, particularly after psilocybin, LSD, and 2,5-Dimethoxy-4-iodoamphetamine (DOI) (interferon gamma, interferon alpha, inflammatory response, IL6-JAK-STAT3, p<0.01). SPs also increased Wnt/β-catenin signaling while (2*R*,6*R*)-HNK and DOI increased mechanistic target of rapamycin complex 1 (mTORC1) signaling.

*Cell Type Annotation Details (scRNAseq)*

As shown in **Figure 2**, distinct populations of putative excitatory (red) and inhibitory (blue) neurons were readily identified based on composite expression scores of canonical markers. The inhibitory populations showed strong expression of GAD1 and GAD2, while excitatory populations predominantly expressed vesicular glutamate transporters VGLUT2 (SLC17A6) and VGLUT3 (SLC17A8). VGLUT1 (SLC17A7) expression was notably minimal in our cultures (**Supplementary Fig. S6A**). Combined with the prominent VGLUT2 expression in our data, this likely indicates an earlier developmental stage for glutamatergic neurons [15]. In support, the excitatory populations also showed robust reelin (RELN) and calretinin (CALB2) (**Supplementary Fig. S6B**) expression without GAD1/2, VGAT (SLC32A1), or canonical interneuron marker co-expression (**Supplementary Fig. S7**), suggesting a Cajal-Retzius-like population with a marginal zone/layer I excitatory cortical neuron phenotype [16]; these cells use VGLUT2 as their primary vesicular glutamate transporter [17] and are distinct from RELN-expressing gamma aminobutyric acid (GABA)-ergic interneurons that co-express GAD1/2s [18-20].

The EX_Mid_1 cluster demonstrated a distinctive co-expression pattern of VGLUT2 (*SLC17A6*) and VGLUT3 (*SLC17A8*), a characteristic typically associated with specialized neurotransmitter-releasing populations including serotonergic dorsal raphe neurons, GABAergic neurons in the olfactory bulb, cholinergic interneurons of the striatum, and non-canonical glutamatergic neurons in the cerebral cortex [21, 22]. To determine the specific identity of this VGLUT2+/VGLUT3+ population, the expression of canonical markers was systematically assessed for multiple neuronal subtypes (**Supplementary Fig. S6**). Analysis revealed that EX_Mid_1 showed minimal to no expression of serotonergic (*TPH2, SERT*), dopaminergic (*TH, VMAT2*), cholinergic (*CHAT*), or progenitor (*ASCL1*) markers (**Supplementary Fig. S4A**). Additionally, this cluster lacked expression of GAD1, GAD2, and VGAT (*SLC32A1*), as well as interneuron-associated neuropeptide markers including *NPY*, *CCK*, *CRF*, *TAC*3, and cannabinoid receptor *CB1R*. The absence of these cell type-specific markers distinguished EX_Mid_1 from canonical VGLUT3-expressing neurons that typically co-release other neurotransmitters [21, 23-25].

In support of a glutamatergic identity, EX_Mid_1 showed robust co-expression of reelin (*RELN*) and calretinin (*CALB2*) alongside VGLUT2 and VGLUT3 (**Supplementary Fig. S6B**). This RELN+/CALB2+/VGLUT2+/GAD- expression profile is characteristic of Cajal-Retzius neurons, specialized glutamatergic neurons that populate the marginal zone/layer I of the developing cortex. The co-expression pattern, combined with the absence of GABAergic and interneuron markers, suggests EX_Mid_1 represents a Cajal-Retzius-like population with a marginal zone/layer I excitatory cortical neuron phenotype [16], which use VGLUT2 as their primary vesicular glutamate transporter [17] and are distinct from RELN-expressing GABAergic interneurons that co-express GAD1/2 [18].

While there is some overlap between VGLUT2 and VGLUT3 in human and rodent cortex, VGLUT2 is most highly expressed in thalamic cells, and VGLUT3 has relatively lower expression levels in the cerebral cortex [26, 27]. As a result, the next step was to further confirm the cortical identity of EX_Mid_1 and other neuronal populations in the dataset. Gene expression scores (Seurat v5 AddModuleScore) [28] demonstrated high expression of canonical cortical transcription factors (*BCL11B, CUX1, CUX2, POU3F2, EMX2, FOXGI, TLE4, RELN*) [29-33] across all clusters including EX_Mid_1, while subcortical markers (*GATA2, GATA3, LHX1, LHX2*) [33, 34] showed minimal expression (**Supplementary Fig. S7A,B**). Marker-level analysis revealed robust expression of cortical layer I-V genes and the dorsal telencephalic determinant EMX2, with absent or minimal subcortical lineage genes (**Supplementary Fig. S7C**), confirming cortical rather than thalamic or brainstem fate. Together, these findings suggest that EX_Mid_1 represents a specialized subset of cortical Cajal-Retzius-like glutamatergic neurons with a unique molecular identity in our iPSC-derived neuronal cultures.

Regarding progenitor cells, three distinct subtypes of progenitor clusters could be differentiated: a highly proliferative NPC subtype (NPC_1, expressing high MKI67), a more quiescent NPC population (NPC_2), and a glial-fated group (NPC_gl); these expressed canonical markers like AQP4 and APOE. GLI3 and PAX7 emerged as top markers for NPC_2. Lastly, the predicted inhibitory neuron populations showed more diverse molecular signatures, particularly IN_Early_1 and In_SST clusters. The IN_Early1 cluster, characterized by markers such as IL1RAPL2, PPP1R1B, and SSTR2, likely represents an early-stage inhibitory population involved in neurodevelopmental processes and receptor-mediated signaling. In contrast, the In_SST cluster, defined by SST, GRIN2A, and CASZ1, represents a mature somatostatin-expressing interneuron population (**Figure 3B**). Compared to other types, both In_SST and IN_Early1 garnered relatively greater KEGG pathway enrichment with regard to neurotransmission (see **Supplementary Fig. S8**).

*Comparative analysis of single-cell and bulk RNA-seq*

When comparing the single-cell pathway enrichment patterns with those observed in bulk RNA-seq, several consistent signatures emerged that likely represent core mechanisms of action for these rapid-acting antidepressants. For the SPs (psilocybin, LSD, and DOI), the upregulation of IL2-STAT5 signaling (for LSD) and IL6-JAK-STAT3 signaling (for psilocybin) observed in specific inhibitory neuronal clusters showed directional agreement with the bulk RNA-seq data. Similarly, the downregulation of the "pancreas beta cells" pathway across multiple inhibitory clusters aligned with the bulk findings. For (2*R*,6*R*)-HNK, the EX_Mature_1 cluster appeared to best reflect the bulk RNA-seq at 24 hours, showing aligned upregulation of mTORC1, fatty acid metabolism, peroxisome activity, MYC targets (V1 and V2), and cellular stress pathways in line with its metabolic upregulation.

**Supplementary References**

1.	Cavalleri L, Dassieni I, Marcotto GS, Zoli M, Pich EM, Collo G. Cortisol-dependent impairment of dendrite plasticity in human dopaminergic neurons derived from hiPSCs is restored by ketamine: Relevance for major depressive disorders. *Neuroscience Applied* 2024; **3:** 104049.

2.	Cavalleri L, Merlo Pich E, Millan MJ, Chiamulera C, Kunath T, Spano PF *et al.* Ketamine enhances structural plasticity in mouse mesencephalic and human iPSC-derived dopaminergic neurons via AMPAR-driven BDNF and mTOR signaling. *Mol Psychiatry* 2018; **23**(4)**:** 812-823.

3.	Ly C, Greb AC, Cameron LP, Wong JM, Barragan EV, Wilson PC *et al.* Psychedelics promote structural and functional neural plasticity. *Cell Rep* 2018; **23**(11)**:** 3170-3182.

4.	Dong C, Ly C, Dunlap LE, Vargas MV, Sun J, Hwang I-W *et al.* Psychedelic-inspired drug discovery using an engineered biosensor. *Cell* 2021; **184**(10)**:** 2779-2792.e2718.

5.	Psilocybin’s effects on neuritogenesis in cancer associated neurons. Available at: <https://openworks.mdanderson.org/cgi/viewcontent.cgi?params=/context/sumexp23/article/1034/&path_info=Psilocybin_s_Effects_on_Neuritogenesis_in_Cancer_Associated_Neuro.pdf>. <https://openworks.mdanderson.org/cgi/viewcontent.cgi?article=1034&context=sumexp23>, 2024, Accessed Date Accessed 2024 Accessed.

6.	Otto ME, van der Heijden KV, Schoones JW, van Esdonk MJ, Borghans LGJM, Jacobs GE *et al.* Clinical pharmacokinetics of psilocin after psilocybin administration: A systematic review and post-hoc analysis. *Clin Pharmacokinet* 2025; **64:** 53-66.

7.	Yu C-L, Liang C-S, Yang F-C, Tu Y-K, Hsu C-W, Carvalho AF *et al.* Trajectory of antidepressant effects after single- or two-dose administration of psilocybin: A systematic review and multivariate meta-analysis. *J Clin Med* 2022; **11**(4)**:** 938.

8.	Gasser P, Holstein D, Michel Y, Doblin R, Yazar-Klosinski B, Passie T *et al.* Safety and efficacy of lysergic acid diethylamide-assisted psychotherapy for anxiety associated with life-threatening diseases. *J Nerv Ment Dis* 2014; **202**(7)**:** 513-520.

9.	Yerubandi A, Thomas JE, Bhuiya NMMA, Harrington C, Villa Zapata L, Caballero J. Acute adverse effects of therapeutic doses of psilocybin: A systematic review and meta-analysis. *JAMA Netw Open* 2024; **7**(4)**:** e245960.

10.	Vargas AS, Luís Â, Barroso M, Gallardo E, Pereira L. Psilocybin as a new approach to treat depression and anxiety in the context of life-threatening diseases—A systematic review and meta-analysis of clinical trials. *Biomedicines* 2020; **8**(9)**:** 331.

11.	Lepack AE, Bang E, Lee B, Dwyer JM, Duman RS. Fast-acting antidepressants rapidly stimulate ERK signaling and BDNF release in primary neuronal cultures. *Neuropharmacology* 2016; **111:** 242-252.

12.	Dai M, Pei X, Wang X-J. Accurate and fast cell marker gene identification with COSG. *Brief Bioinform* 2022; **23**(2)**:** bbab579.

13.	Trapnell C, Cacchiarelli D, Grimsby J, Pokharel P, Li S, Morse M *et al.* The dynamics and regulators of cell fate decisions are revealed by pseudotemporal ordering of single cells. *Nat Biotechnol* 2014; **32**(4)**:** 381-386.

14.	Voleti B, Duman RS. The roles of neurotrophic factor and Wnt signaling in depression. *Clin Pharmacol Ther* 2012; **91:** 333-338.

15.	Miyazaki T, Fukaya M, Shimizu H, Watanabe M. Subtype switching of vesicular glutamate transporters at parallel fibre-Purkinje cell synapses in developing mouse cerebellum. *Eur J Neurosci* 2003; **17**(12)**:** 2563-2572.

16.	Anstötz M, Quattrocolo G, Maccaferri G. Cajal-Retzius cells and GABAergic interneurons of the developing hippocampus: close electrophysiological encounters of the third kind. *Brain Res* 2018; **1697:** 124-133.

17.	Anstötz M, Lee SK, Maccaferri G. Glutamate released by Cajal-Retzius cells impacts specific hippocampal circuits and behaviors. *Cell Rep* 2022; **39:** 110822.

18.	Anstötz M, Maccaferri G. A toolbox of criteria for distinguishing Cajal–Retzius cells from other neuronal types in the postnatal mouse hippocampus. *eNeuro* 2020; **7:** ENEURO.0516-0519.2019.

19.	Hevner RF, Neogi T, Englund C, Daza RAM, Fink A. Cajal–Retzius cells in the mouse: transcription factors, neurotransmitters, and birthdays suggest a pallial origin. *Brain Res Dev Brain Res* 2003; **141:** 39-53.

20.	Pohlkamp T, Dávid C, Cauli B, Gallopin T, Bouché E, Karagiannis A *et al.* Characterization and distribution of Reelin-positive interneuron subtypes in the rat barrel cortex. *Cereb Cortex* 2014; **24:** 3046-3058.

21.	Favier M, Pietrancosta N, El Mestikawy S, Gangarossa G. Leveraging VGLUT3 functions to untangle brain dysfunctions. *Trends Pharmacol Sci* 2021; **42**(6)**:** 475-490.

22.	Herzog E, Gilchrist J, Gras C, Muzerelle A, Ravassard P, Giros B *et al.* Localization of VGLUT3, the vesicular glutamate transporter type 3, in the rat brain. *Neuroscience* 2004; **123**(4)**:** 983-1002.

23.	Zimmermann J, Herman MA, Rosenmund C. Co-release of glutamate and GABA from single vesicles in GABAergic neurons exogenously expressing VGLUT3. *Front Synaptic Neurosci* 2015; **7:** 16.

24.	Trudeau L-E, Mestikawy SE. Glutamate cotransmission in cholinergic, GABAergic and monoamine systems: Contrasts and commonalities. *Front Neural Circuits* 2018; **12:** 113.

25.	Bhaduri A, Sandoval-Espinosa C, Otero-Garcia M, Oh I, Yin R, Eze UC *et al.* An atlas of cortical arealization identifies dynamic molecular signatures. *Nature* 2021; **598:** 200-204.

26.	Vigneault É, Poirel O, Riad M, Prud’homme J, Dumas S, Turecki G *et al.* Distribution of vesicular glutamate transporters in the human brain. *Front Neuroanat* 2015; **9:** 23.

27.	El Mestikawy S, Wallén-Mackenzie A, Fortin GM, Descarries L, Trudeau L-E. From glutamate co-release to vesicular synergy: vesicular glutamate transporters. *Nat Rev Neurosci* 2011; **12:** 204-216.

28.	Hao Y, Stuart T, Kowalski MH, Choudhary S, Hoffman P, Hartman A *et al.* Dictionary learning for integrative, multimodal and scalable single-cell analysis. *Nat Biotechnol* 2024; **42**(2)**:** 293-304.

29.	Lennon MJ, Jones SP, Lovelace MD, Guillemin GJ, Brew BJ. Bcl11b-A critical neurodevelopmental transcription factor-roles in health and disease. *Front Cell Neurosci* 2017; **11:** 89.

30.	Casingal CR, Nakagawa N, Yabuno-Nakagawa K, Meyer C, Liu S, Gkini V *et al.* TSC tunes progenitor balance and upper-layer neuron generation in neocortex. *Nature* 2025; **Dec 3 [online ahead of print]**.

31.	Shoemaker LD, Arlotta P. Untangling the cortex: Advances in understanding specification and differentiation of corticospinal motor neurons. *Bioessays* 2010; **32:** 197-206.

32.	Muzio L, Mallamaci A. Foxg1 confines Cajal-Retzius neuronogenesis and hippocampal morphogenesis to the dorsomedial pallium. *J Neurosci* 2005; **25:** 4435-4441.

33.	Azzarelli R, Hardwick LJA, Philpott A. Emergence of neuronal diversity from patterning of telencephalic progenitors. *Wiley Interdiscip Rev Dev Biol* 2015; **4:** 197-214.

34.	Subramanian L, Remedios R, Shetty A, Tole S. Signals from the edges: The cortical hem and antihem in telencephalic development. *Semin Cell Dev Biol* 2009; **20:** 712-718.

**Supplementary Figures**

**Supplementary Figure S1. Experimental methodology.** (A) Experimental diagram of methods. (B) Experimental groups, drug treatment concentrations, and timepoints. *Ketamine subgroup excluded from Western blot and RNA-sequencing analyses due to low sample viability after cell dissociation. (2*R*,6*R*)-HNK: (2*R*,6*R*)-hydroxynorketamine; DOI: 2,5-Dimethoxy-4-iodoamphetamine; LSD: lysergic acid diethylamide; PBMC: peripheral blood mononuclear cell.

**Supplementary Figure S2: (A – J)** Expression of N-methyl-D-aspartate (NMDA) and serotonin receptors in induced pluripotent stem cells (iPSC)-derived forebrain neurons. Transcripts per million (TPKM) expression levels of *ALPL, GRIN1, GRIN2A, GRIN2B, GRIN2C, GRIN2D, HTR1A, HTR1B, HTR2A*, and *HTR2C* across treatments and timepoints. **(K)** Relative distribution of all canonical receptor subtypes within the bulk RNA-sequencing gene dataset, from low (*HTR1A, HTR1B*) to medium (*ALPL, GRIN2C, HTR2A, HTR2C*) to high (*GRIN2A, GRIN2D, GRIN1, GRIN2B*) expression levels. HNK: (2*R*,6*R*)-hydroxynorketamine; LSD: lysergic acid diethylamide; DOI: 2,5-Dimethoxy-4-iodoamphetamine

**Supplementary Figure S3.** Differential expression was highly correlated between drug treatments at six and 24 hours, but not between timepoints. **(A)** UpSet plot of differentially expressed genes (DEGs) from bulk RNA-sequencing data after drug treatment at six and 24 hours, as well as overlapping differential gene expression between drug groups. Lysergic acid diethylamide (LSD) and psilocybin at 24 hours had the most overlapping DEGs. **(B)** Spearman correlation matrix of gene log-fold changes after drug treatment. Directionality of gene changes in comparison to vehicle control were highly correlated within timepoints between drugs. HNK: (2*R*,6*R*)-hydroxynorketamine; DOI: 2,5-Dimethoxy-4-iodoamphetamine.

**Supplementary Figure S4:** Healthy volunteer (HV) and treatment-resistant depression (TRD) cell lines demonstrated diagnostic differences across many of the hallmark signaling pathways measured. In addition, cell lines had diagnosis-specific reactions to treatments at six and 24 hours. Data are from bulk RNA-sequencing analysis. Color scale indicates delta scores. Blue represents downregulated pathways and red represents up-regulated pathways compared to vehicle-treated controls. *p<0.05, **p<0.01, ***p<0.001 as false discovery rate (FDR)-corrected values. HNK: (2*R*,6*R*)-hydroxynorketamine; LSD: lysergic acid diethylamide; DOI: 2,5-Dimethoxy-4-iodoamphetamine

**Supplementary Figure S5:** Cell counts for individual conditions across single cell clusters. HNK: (2*R*,6*R*)-hydroxynorketamine; LSD: lysergic acid diethylamide.

**Supplementary Figure S6.** Expression of neurotransmitter-related and cell type-specific marker genes across neuronal clusters. **(a)** Violin plots comparing expression of canonical neurotransmitter synthesis, transport, and cell-type markers in EX_Mid_1 versus representative excitatory, inhibitory, and interneuronal clusters. Markers include: serotonergic (*TPH2, SERT*), dopaminergic (*TH, VMAT2*), cholinergic (*CHAT*), interneuron/neuropeptide (*CCK, TAC3, NPY, CRF*), cannabinoid (*CB1R*), calcium-binding protein (*CALB2*), and progenitor (*ASCL1*) markers, suggesting EX_Mid_does not represent canonical VGLUT3+ co-releasing neuron populations. (**b**) Dot plot showing average expression levels (color intensity) and percentage of expressing cells (dot size) for vesicular glutamate transporters (*SLC17A6, SLC17A7, SLC17A8*), gamma aminobutyric acid (GABA)-ergic markers (*GAD1, GAD2*), intraneuronal (*PVALB, SST, VIP*), and excitatory Cajal-Retzius markers (*RELN, CALB2*). The distinct RELN+ / CALB2+ / SLC17A6+ / GAD- / Interneuron- profile suggests marginal zone/layer I like excitatory activity for these clusters, distinguishing them from GABAergic interneuronal populations that are canonically RELN+.

**Supplementary Figure S7.** Induced pluripotent stem cells (iPSC)-derived neurons display cortical telencephalic identity. **(a,b)** Module scores for cortical transcription factors (*BCL11B, CUX1, CUX2, POU3F2, EMX2, FOXGI, TLE4, RELN*) [29-33] and subcortical markers (*GATA2, GATA3, LHX1, LHX2*) [33, 34] projected onto a uniform manifold approximation and projection (UMAP). Cortical gene lineage and transcription factor (TF) module scores calculated with Seurat v5 (AddModuleScore) [28] were high across neuronal populations **(a)**, while subcortical module scores were minimal **(b)**. **(c)** DotPlot of cortical versus subcortical marker expression across cell types from **(a,b)**. Excitatory clusters showed robust expression of cortical layer I-V markers and dorsal telencephalic determinant EMX2 [33], with absent or minimal subcortical lineage genes, confirming cortical identity rather than thalamic or brainstem fate.

**Supplementary Figure S8.** KEGG pathway enrichment for single-cell clusters. Top KEGG pathway enrichment terms for all clusters except EX_Mature_1 and IN_Mid_2 (shown separately in **Supplementary Figure S9** to highlight (*2R,6R*)-hydroxynorketamine (HNK) responsive cell types). Highly enriched pathways were identified using the top 150 marker genes per cluster (COSG package) and analyzed with the clusterProfiler EnrichKEGG function. To broadly characterize enrichment across cell types, significance was set at an uncorrected p < 0.05.

**Supplementary Figure 9.** KEGG Pathway Enrichment for Hydroxynorketamine (HNK)-Responsive (EX_Mature_1 and IN_Mid_2) Cell-Type Clusters. Top KEGG pathway enrichment terms for EX_Mature_1 and IN_Mid_2, which were analyzed separately from other clusters (see **Figure 5** and **Supplementary Figure S8**). The heatmap (top right) provides an overview of the full single-cell RNA-seq dataset shown in Figure 5, with boxed regions highlighting the two HNK-responsive cell-type clusters (EX_Mature_1 and IN_Mid_2) that exhibited the most significant pathway enrichment hits. Network visualization displays enriched pathways and associated genes for (2*R*,6*R*)-HNK highly-responsive clusters, with node colors corresponding to cluster identity. Pathways related to neuroactive ligand-receptor interaction, nicotine and morphine addiction, and oxytocin signaling were enriched in these clusters. Pathways highly enriched were identified using the top 150 marker genes per cluster (COSG package) and analyzed with the clusterProfiler EnrichKEGG function. To broadly characterize enrichment across cell types, significance was set at an uncorrected p<0.05.

**Supplementary Figure S10. Significant changes in protein expression after treatment at six hours and 24 hours.** At 24 hours, (2*R*,6*R*)-hydroxynorketamine (HNK) significantly increased multiple proteins related to **(A – C)** cell survival (ERK, p-eEF2, eEF2), **(D, E)** protein synthesis regulation (p-eIF4E, eIF4E), and **(F – H)** synaptic strengthening (4EBP1, PSD-95, Synaptotagmin). **(I)** Representative Western blotting images of each significant protein. All data normalized to total protein expression (Ponceau). Data expressed as mean ± SEM.

**Supplementary Figure S11. Treatment with all drugs increased synapse counts at 24 hours.** Average synapse counts were determined by immunofluorescent co-expression of postsynaptic density protein 95 (PSD-95) (red), MAP2 (blue), synaptophysin (green), and DAPI (blue) as a nuclear marker in Arivis Vision4D. At six hours, lysergic acid diethylamide (LSD) and psilocybin (PSI) decreased average synapse counts. At 24 hours, all treatments significantly increased synapse count from vehicle controls. Data expressed as mean ± SEM. HNK: (2*R*,6*R*)-hydroxynorketamine; DOI: 2,5-Dimethoxy-4-iodoamphetamine; KET: ketamine; ACN: Acetonitrile.

**Supplementary Figure S12.** Gene ontology molecular function (GO:MF) enrichment analysis of overlapping upregulated genes. Dot plot showing the top 10 enriched GO molecular function terms for the 102 upregulated genes that overlapped between induced pluripotent stem cell (iPSC)-derived neuronal cultures and CSF proteomics at 24 hours post-treatment. The x-axis represents enrichment strength, dot size indicates gene count, and color intensity represents false discovery rate (FDR) values. Growth factor signaling pathways, particularly insulin-like growth factor receptor activity and neurotrophin receptor activity, showed the strongest enrichment, suggesting these molecular functions may represent translatable mechanisms between *in vitro* and *in vivo* ketamine responses.
