# Supplementary material for: Time-dependent effects of rapid-acting antidepressants in iPSC-derived neurons from treatment-resistant depression and healthy volunteers
Source: Mol Psychiatry. 2026 Jul 24;31(12):7157–69. doi: 10.1038/s41380-026-03768-0 (PMC13736488; doi:10.1038/s41380-026-03768-0)
Supplement: Supplementary file 2 [file 41380_2026_3768_MOESM2_ESM.docx]

*Johnston et al — Time-Dependent Effects of Rapid-Acting Antidepressants in iPSC-Derived Neurons from Treatment-Resistant Depression and Healthy Volunteers*

**SUPPLEMENTARY TABLES**

**Supplementary Table S1. Participant Demographics.**

|  | **HV (n = 5)** | **TRD (n = 5)** |
| --- | --- | --- |
| **Age (in years)** | | |
| Mean (SD) | 46.5 (20.5) | 40.2 (8.6) |
| Range | 20 - 68 | 31 - 47 |
| **Sex** (n, (%)) | | |
| Female | 5 (100) | 5 (100) |
| Male | 0 (0) | 0 (0) |
| **Self-described Race** (n, (%)) | | |
| White | 5 (100) | 3 (60) |
| Black or African American | 0 (0) | 2 (40) |
| Other | 0 (0) | 0 (0) |
| **Baseline MADRS score** | | |
| Mean (SD) | N/A | 30.7 (3.1) |
| Range | N/A | 29 – 38 |
| **Number of Failed Antidepressant Trials** | | |
| Mean (SD) | N/A | 6.7 (4.2) |
| Range | N/A | 3 - 13 |
| **Age of Onset** | | |
| Mean (SD) | N/A | 15.5 (4.3) |
| Range | N/A | 10 – 23 |

HV: healthy volunteer; TRD: treatment-resistant depression; MADRS: Montgomery-Asberg Depression Rating Scale

**Supplementary Table S2. scRNA-seq sample summary by diagnosis group.**

| Group | Conditions (n) | Cells Retained | Mean Reads / Cell | Median Genes / Cell | Mapping Rate (%) |
| --- | --- | --- | --- | --- | --- |
| HV (n=1 donor) | 4 | 15,312 | 44,472 | 3,423 | 73 |
| TRD (n=1 donor) | 3 | 11,329 | 47,853 | 3,547 | 78 |
| **Total** | **—** | **26,641** | **—** | **—** | **—** |

Cell counts and sequencing quality metrics for single-cell RNA-seq samples, aggregated by diagnosis. Mean reads per cell, median genes per cell, and mapping rate reflect averages across samples within each group. HV: healthy volunteer; TRD: treatment-resistant depression.

**Supplementary Table S3. scRNA-seq sequencing quality metrics by treatment condition and diagnosis.**

|  | Group | Cells Retained | Mean Reads/Cell | Median Genes/Cell | Mapping Rate (%) |
| --- | --- | --- | --- | --- | --- |
| **Control** | | | | | |
|  | HV | 4,928 | 42,022 | 4,189 | 72.7 |
|  | TRD | 3,206 | 51,804 | 3,256 | 80.4 |
| Total | — | 8,134 | — | — | — |
| **HNK** | | | | | |
|  | HV | 4,250 | 20,152 | 1,887 | 74.3 |
|  | TRD | 5,249 | 24,679 | 3,258 | 76.4 |
| Total | — | 9,499 | — | — | — |
| **LSD** | | | | | |
|  | HV | 3,974 | 40,500 | 4,914 | 73.5 |
| Total | — | 3,974 | — | — | — |
| **Psilocybin** | | | | | |
|  | HV | 2,160 | 75,212 | 2,702 | 71.7 |
|  | TRD | 2,874 | 67,076 | 4,126 | 77.2 |
| Total | — | 5,034 | — | — | — |
| **Grand Total** | — | 26,641 | — | — | — |

Cell counts and sequencing quality metrics for each treatment condition, stratified by diagnosis. LSD data were available for the HV donor only, as the corresponding TRD sample was excluded during quality control due to an elevated proportion of low-complexity cells. Mean reads per cell and median genes per cell reflect CellRanger summary metrics for the corresponding sample. HV: healthy volunteer; TRD: treatment-resistant depression; HNK: (2*R*,6*R*)-hydroxynorketamine.

**Supplementary Table S4:** Differentially Expressed Genes after (2*R*,6*R*)-HNK at Six Hours

| **Gene Name** | **logFC** | **AveExpr** | **t** | **P.Value** | **adj.P.Val** | **B** | **z.std** |
| --- | --- | --- | --- | --- | --- | --- | --- |
| ACTL7A | 3.16084784 | -6.4623904 | 5.53796691 | 3.0337E-07 | 0.00677762 | -1.6394224 | 5.12134033 |
| LRRC52-AS1 | -2.8952537 | -6.2565009 | -4.0832169 | 0.00010256 | 0.99994333 | -3.0254553 | -3.8844514 |
| H2AC14 | -2.4187542 | -6.6666084 | -3.8991248 | 0.00019807 | 0.99994333 | -3.2031556 | -3.7214663 |
| SNHG25 | -2.9342172 | -4.5985705 | -3.806432 | 0.00027117 | 0.99994333 | -3.4022441 | -3.641393 |
| DEFB134 | 2.55097855 | -5.9911923 | 3.61867015 | 0.00051343 | 0.99994333 | -3.352109 | 3.47365179 |
| LINC02405 | -2.3563037 | -6.1824692 | -3.5361442 | 0.00067209 | 0.99994333 | -3.4924016 | -3.4007166 |
| GSTT2 | -2.0617137 | -4.97747 | -3.474954 | 0.00082372 | 0.99994333 | -3.50319 | -3.3446987 |
| DHFRP1 | -1.7156524 | -3.0801332 | -3.4637148 | 0.00086171 | 0.99994333 | -3.6410382 | -3.3321712 |
| FAM138B | -2.7277976 | -5.4291018 | -3.4116358 | 0.00101185 | 0.99994333 | -3.6270784 | -3.2872116 |
| TMEM256 | -0.715149 | 0.8713271 | -3.3003773 | 0.00144805 | 0.99994333 | -3.7041855 | -3.1848989 |
| GTF2A2 | -0.3206196 | 5.17466855 | -3.3014363 | 0.00146572 | 0.99994333 | -3.6467617 | -3.1813872 |
| LINC02226 | -2.0994778 | -5.7685403 | -3.2586257 | 0.0016351 | 0.99994333 | -3.6056382 | -3.1495709 |
| DYNLL1 | -0.3365204 | 6.96602355 | -3.2560714 | 0.00166541 | 0.99994333 | -3.6717828 | -3.1442015 |
| UQCRQ | -0.4860904 | 5.48457392 | -3.2434083 | 0.00173317 | 0.99994333 | -3.677526 | -3.1325132 |
| LINC02338 | 1.70642926 | -4.043712 | 3.22197367 | 0.00184857 | 0.99994333 | -3.7401179 | 3.11354009 |
| CRYGD | -2.5361295 | -3.9563032 | -3.2164462 | 0.00187819 | 0.99994333 | -3.802514 | -3.108847 |
| UBL5 | -0.5613139 | 5.32625606 | -3.2053101 | 0.00188115 | 0.99994333 | -3.7086634 | -3.1083817 |
| SH3BGR | -1.0607349 | -1.6644513 | -3.2137895 | 0.00190389 | 0.99994333 | -3.7478621 | -3.1048292 |
| ZMPSTE24-DT | -0.2942333 | 3.8535908 | -3.2078515 | 0.0019356 | 0.99994333 | -3.6925285 | -3.09994 |
| LINC01936 | -1.3867992 | -1.7096561 | -3.1511946 | 0.00229135 | 0.99994333 | -3.7359568 | -3.0496149 |
| H2BC17 | -2.8162023 | -5.2314824 | -3.0824935 | 0.00272878 | 0.99994333 | -3.7787231 | -2.9967456 |
| ZFPL1 | -0.3754933 | 2.19102473 | -3.0858096 | 0.00279645 | 0.99994333 | -3.7466077 | -2.9892698 |
| MTND4LP1 | -1.5622646 | -6.5677691 | -3.0752642 | 0.0028558 | 0.99994333 | -3.7256456 | -2.9828474 |
| MRPL41 | -0.4583812 | 4.89382813 | -3.0684082 | 0.00296566 | 0.99994333 | -3.8031499 | -2.9712747 |
| MIR548AT | 2.20379408 | -5.8340645 | 3.04374267 | 0.00306764 | 0.99994333 | -3.6988354 | 2.96087742 |
| RN7SL481P | 1.45144257 | -1.4619489 | 3.01691485 | 0.00339979 | 0.99994333 | -3.8334753 | 2.929069 |
| UBE2V1P14 | 1.93516545 | -5.6832764 | 3.01547727 | 0.00342172 | 0.99994333 | -3.6965772 | 2.92706999 |
| CIAO2B | -0.4454648 | 5.38305344 | -3.0061527 | 0.00352071 | 0.99994333 | -3.8171706 | -2.9181886 |
| RPS27AP5 | -1.413897 | -2.5211669 | -3.0027055 | 0.00355869 | 0.99994333 | -3.9442407 | -2.9148416 |
| RNU6ATAC16P | 1.65078295 | -3.963212 | 2.94655518 | 0.00419432 | 0.99994333 | -3.8268328 | 2.86316473 |
| EML4-AS1 | -2.5064517 | -5.6514456 | -2.9482583 | 0.00419934 | 0.99994333 | -3.8347986 | -2.8627863 |
| COPS9 | -0.592118 | 4.53308087 | -2.9397315 | 0.00426862 | 0.99994333 | -3.8887274 | -2.8575968 |
| NAP1L1P1 | -1.7995323 | -1.8989367 | -2.9386331 | 0.00429618 | 0.99994333 | -3.8753778 | -2.8555538 |
| MRPS21 | -0.2640909 | 6.11057328 | -2.9335122 | 0.00436176 | 0.99994333 | -3.8407777 | -2.8507402 |
| NDUFA1 | -0.7571576 | 4.45130055 | -2.9253867 | 0.00443455 | 0.99994333 | -3.8652887 | -2.8454733 |
| AASDH | 0.27178756 | 4.28779694 | 2.92292691 | 0.00450108 | 0.99994333 | -3.8330491 | 2.84072713 |
| LINC02886 | -2.3523615 | -6.1473842 | -2.9089623 | 0.00457369 | 0.99994333 | -3.8522946 | -2.8356196 |
| KRT18P40 | 2.28799826 | -5.5036918 | 2.91824222 | 0.00459959 | 0.99994333 | -3.8458838 | 2.83381519 |
| LGALS9B | -2.3977788 | -5.4797538 | -2.9001913 | 0.00479666 | 0.99994333 | -3.912184 | -2.8203816 |
| FKTN-AS1 | -2.2441854 | -5.9035358 | -2.8914657 | 0.00490168 | 0.99994333 | -3.8714308 | -2.8134251 |
| SNRPD2 | -0.3815841 | 5.93176024 | -2.8900686 | 0.00495901 | 0.99994333 | -3.8787937 | -2.8096847 |
| CDPF1P1 | -0.784163 | -0.2039303 | -2.8733278 | 0.0052123 | 0.99994333 | -3.9068453 | -2.7936119 |
| RNU6-1048P | 1.72868254 | -3.4011205 | 2.87271068 | 0.00532881 | 0.99994333 | -3.8405509 | 2.7864544 |
| CDHR18P | 0.81804561 | -0.8265239 | 2.85887108 | 0.00542166 | 0.99994333 | -3.8645693 | 2.78085134 |
| MTUS1-DT | -2.2302839 | -5.2577195 | -2.841346 | 0.00565669 | 0.99994333 | -3.8592376 | -2.7670451 |
| GGTLC3 | 1.53821292 | -4.5594655 | 2.84227071 | 0.00568299 | 0.99994333 | -3.8689244 | 2.76553302 |
| NOP10 | -0.4582167 | 4.77684911 | -2.8375304 | 0.00573614 | 0.99994333 | -3.9196917 | -2.7624952 |
| LTN1 | 0.2346632 | 5.97964391 | 2.83966112 | 0.00576171 | 0.99994333 | -3.8826715 | 2.76104282 |
| CHCHD1 | -0.4466216 | 4.07730595 | -2.8223991 | 0.0058953 | 0.99994333 | -3.9148944 | -2.7535487 |
| C4orf48 | -0.5511254 | 6.10160274 | -2.7942731 | 0.00648822 | 0.99994333 | -3.9318137 | -2.7220302 |
| GOLGB1 | 0.26592783 | 7.69587605 | 2.79071627 | 0.00658602 | 0.99994333 | -3.9187289 | 2.71708261 |
| RPL6P9 | 1.73831945 | -3.8419612 | 2.78350603 | 0.0066938 | 0.99994333 | -3.9114036 | 2.71170565 |
| PLGRKT | -0.3581225 | 3.76078449 | -2.7876539 | 0.00672452 | 0.99994333 | -3.9362044 | -2.7101876 |
| EPX | 0.54219527 | 1.14345701 | 2.77224378 | 0.00689316 | 0.99994333 | -3.9046032 | 2.70196204 |
| ZKSCAN8P2 | 2.0050639 | -5.9306356 | 2.76460506 | 0.00706675 | 0.99994333 | -3.9176487 | 2.69368232 |
| MARF1 | 0.20978039 | 6.92135529 | 2.75585844 | 0.00724071 | 0.99994333 | -3.9358737 | 2.68556583 |
| PRDX2P3 | 1.74426416 | -3.4576736 | 2.74459169 | 0.00744598 | 0.99994333 | -3.9110834 | 2.67621056 |
| GOLGA7B-DT | -1.8338727 | -3.6304411 | -2.7446027 | 0.00749652 | 0.99994333 | -3.9955675 | -2.6739431 |
| BOLA2B | -1.5062444 | -2.9669234 | -2.7427396 | 0.0075503 | 0.99994333 | -4.0079624 | -2.6715447 |
| HINT1 | -0.3379381 | 6.74624608 | -2.7356406 | 0.00764337 | 0.99994333 | -3.9469211 | -2.6674305 |
| KRT18P50 | -2.0342191 | -6.2520056 | -2.7349981 | 0.00765713 | 0.99994333 | -3.9374936 | -2.6668259 |
| DNAJB12P1 | -1.9350272 | -4.3861012 | -2.7295555 | 0.00776408 | 0.99994333 | -3.9932233 | -2.6621607 |
| FAAP20 | -0.2524404 | 5.7331599 | -2.7306714 | 0.00776434 | 0.99994333 | -3.9571113 | -2.6621493 |
| LINC02093 | -2.1407865 | -5.7831796 | -2.7292921 | 0.00779504 | 0.99994333 | -3.9579482 | -2.660821 |
| LINC00670 | 1.93165613 | -6.1196229 | 2.72158191 | 0.00782341 | 0.99994333 | -3.9129432 | 2.65959724 |
| TXNDC17 | -0.3497927 | 3.82009318 | -2.7224818 | 0.00797826 | 0.99994333 | -3.9888716 | -2.6529885 |
| RNU6-444P | 1.99940296 | -5.5184858 | 2.71128747 | 0.00805746 | 0.99994333 | -3.9680345 | 2.64965243 |
| ELOCP2 | -2.4882793 | -4.8915333 | -2.7143416 | 0.0080853 | 0.99994333 | -3.9427387 | -2.6484867 |
| ETFDH | 0.31033699 | 4.90019757 | 2.71294472 | 0.00816823 | 0.99994333 | -3.92874 | 2.64503578 |
| DCTN6-DT | -0.6506506 | 0.62219799 | -2.7102677 | 0.00820123 | 0.99994333 | -3.9519066 | -2.6436711 |
| RN7SL236P | -2.4459934 | -5.9637502 | -2.6993966 | 0.00857165 | 0.99994333 | -3.9801591 | -2.6286818 |
| TMEM258 | -0.456131 | 4.22386403 | -2.6919612 | 0.00861225 | 0.99994333 | -3.9902096 | -2.6270743 |
| KIAA0825 | 0.35610346 | 2.51154554 | 2.68817742 | 0.00872207 | 0.99994333 | -3.9397115 | 2.62275984 |
| GLYATL1B | 1.84279324 | -4.3189066 | 2.68536264 | 0.00882689 | 0.99994333 | -4.0314391 | 2.61868699 |
| MAP3K7CL | -1.4118778 | -2.0471501 | -2.6813834 | 0.00900716 | 0.99994333 | -4.0597035 | -2.6117822 |
| RAB44 | 2.30532126 | -3.8711614 | 2.66905131 | 0.00915508 | 0.99994333 | -3.9868358 | 2.60620831 |
| KRT33B | 1.32782683 | -6.5589766 | 2.66887163 | 0.00917536 | 0.99994333 | -3.8987214 | 2.60545035 |
| RICTOR | 0.26004049 | 5.68808058 | 2.66934716 | 0.00917552 | 0.99994333 | -3.9737205 | 2.60544432 |
| MED30 | -0.3556411 | 3.20730894 | -2.6627832 | 0.00934673 | 0.99994333 | -3.993703 | -2.5991044 |
| MIR7107 | 1.99599812 | -5.082662 | 2.65912581 | 0.00940872 | 0.99994333 | -3.9722816 | 2.5968347 |
| MRPL33 | -0.4725501 | 3.80733781 | -2.6608874 | 0.009445 | 0.99994333 | -4.0054839 | -2.5955122 |
| TBL1XR1-AS1 | 1.88061187 | -5.8641406 | 2.65806556 | 0.00945197 | 0.99994333 | -3.946389 | 2.59525904 |
| RN7SL181P | -0.9497273 | -1.841149 | -2.6509091 | 0.00950561 | 0.99994333 | -3.9697726 | -2.5933135 |
| KCNA7 | 2.12970554 | -5.0209997 | 2.65438091 | 0.00953708 | 0.99994333 | -3.9498019 | 2.59217668 |
| IL18RAP | -1.9397229 | -5.943436 | -2.6492968 | 0.00967113 | 0.99994333 | -3.9304697 | -2.5873713 |
| SCARNA18 | 2.02974529 | -5.4169033 | 2.63886082 | 0.00989349 | 0.99994333 | -3.989203 | 2.57952984 |
| NTM-AS1 | -2.3475751 | -5.4600276 | -2.6418146 | 0.00989722 | 0.99994333 | -4.0114804 | -2.5793998 |
| FABP5 | -0.452741 | 3.79011616 | -2.6424211 | 0.00991426 | 0.99994333 | -4.0638176 | -2.5788056 |
| NOL8 | 0.22307124 | 5.03182835 | 2.63426134 | 0.01013313 | 0.99994333 | -3.9899144 | 2.571253 |
| LINC00934 | -2.0760048 | -6.0213916 | -2.6326709 | 0.01016117 | 0.99994333 | -3.9969274 | -2.5702957 |
| RBBP6 | 0.1746956 | 6.89219435 | 2.63230694 | 0.01017689 | 0.99994333 | -4.0000055 | 2.56976042 |
| MRPL51 | -0.4060016 | 5.12568369 | -2.6336665 | 0.01020454 | 0.99994333 | -4.0164129 | -2.5688202 |
| ANKRD18CP | 0.56295412 | -0.0807385 | 2.62667722 | 0.01031775 | 0.99994333 | -3.9739474 | 2.56499426 |
| AHCYP3 | -2.4406364 | -5.3863113 | -2.6244417 | 0.01036958 | 0.99994333 | -4.0686689 | -2.5632551 |
| COMMD6 | -0.2971807 | 4.91623866 | -2.6199279 | 0.01049311 | 0.99994333 | -4.0280894 | -2.5591412 |
| LINC02636 | 1.48075176 | -2.9388018 | 2.61848484 | 0.01050158 | 0.99994333 | -4.0061764 | 2.55886078 |
| DHRS9 | -2.1269895 | -5.5480995 | -2.6163241 | 0.01063125 | 0.99994333 | -4.0603769 | -2.5545913 |
| NAA38 | -0.3733809 | 4.86670796 | -2.6150735 | 0.01067243 | 0.99994333 | -4.0187579 | -2.5532455 |
| NUDT8 | -0.5388562 | 1.49949893 | -2.6067748 | 0.01090454 | 0.99994333 | -4.0326101 | -2.5457432 |
| DNPEP-AS1 | -1.754206 | -3.2947658 | -2.5938699 | 0.01119407 | 0.99994333 | -4.0799946 | -2.5365815 |
| KIAA1671-AS1 | -2.1304482 | -4.5134532 | -2.5964916 | 0.01124454 | 0.99994333 | -4.0665306 | -2.5350059 |
| RPLP1 | -0.4462865 | 8.58278093 | -2.5946523 | 0.01124845 | 0.99994333 | -4.0275661 | -2.5348841 |
| IFNWP9 | 1.97587886 | -5.9460171 | 2.58840661 | 0.01125185 | 0.99994333 | -3.988832 | 2.53477824 |
| NAIPP2 | 0.37203455 | 1.61386616 | 2.59439614 | 0.01125905 | 0.99994333 | -3.9846913 | 2.53455424 |
| RPA3 | -0.2705732 | 3.32175527 | -2.5948794 | 0.01128495 | 0.99994333 | -4.0455246 | -2.5337491 |
| LRIG2 | 0.20551681 | 5.47264643 | 2.58944751 | 0.01142583 | 0.99994333 | -4.0079279 | 2.52939822 |
| RPS15AP1 | -1.3505933 | -2.1750557 | -2.5840704 | 0.0115696 | 0.99994333 | -4.05873 | -2.5250068 |
| POLR2L | -0.6074255 | 4.72001414 | -2.5841191 | 0.01160129 | 0.99994333 | -4.0635894 | -2.5240457 |
| RNGTTP1 | 1.99374737 | -5.4132485 | 2.5774986 | 0.01171149 | 0.99994333 | -3.9425922 | 2.52072043 |
| ROMO1 | -0.6818832 | 4.46750838 | -2.5907733 | 0.01179932 | 0.99994333 | -4.038334 | -2.5180899 |
| COX7C | -0.4089936 | 7.00310012 | -2.5741197 | 0.01186732 | 0.99994333 | -4.0364339 | -2.5160651 |
| BAD | -0.33679 | 4.24570781 | -2.571903 | 0.01191632 | 0.99994333 | -4.0480706 | -2.5146127 |
| NENF | -0.4090242 | 5.16328567 | -2.5743634 | 0.01193338 | 0.99994333 | -4.0462366 | -2.5141083 |
| SIGIRR | -0.5396076 | 1.98986512 | -2.5700848 | 0.01202775 | 0.99994333 | -4.0281075 | -2.5113293 |
| LGALSL-DT | -0.6789015 | -0.5746771 | -2.5656704 | 0.01216857 | 0.99994333 | -4.0296146 | -2.5072177 |
| ELOB | -0.2880801 | 6.99510076 | -2.561847 | 0.01222678 | 0.99994333 | -4.0399364 | -2.5055308 |
| NAB2 | -0.3318794 | 6.03162751 | -2.563231 | 0.01222845 | 0.99994333 | -4.0339551 | -2.5054823 |
| TAS2R4 | 0.68337206 | 0.36952885 | 2.56285515 | 0.01223242 | 0.99994333 | -3.9934819 | 2.50536766 |
| MIR657 | -1.6995409 | -4.7485232 | -2.5586267 | 0.01232743 | 0.99994333 | -4.0128123 | -2.5026301 |
| COX6B1 | -0.421927 | 6.18023302 | -2.5605386 | 0.0123341 | 0.99994333 | -4.0438463 | -2.5024386 |
| KRT10 | -0.6268272 | 4.0821819 | -2.5522459 | 0.01240675 | 0.99994333 | -4.0575256 | -2.500359 |
| RNF208 | -0.2201399 | 5.26528228 | -2.5566947 | 0.01247982 | 0.99994333 | -4.0430416 | -2.4982784 |
| COX14 | -0.3961885 | 3.12720318 | -2.555015 | 0.01251695 | 0.99994333 | -4.051095 | -2.4972251 |
| IFNA5 | -1.835301 | -6.1427194 | -2.5518475 | 0.01255175 | 0.99994333 | -4.0312512 | -2.4962405 |
| TRAF6 | 0.22417594 | 5.28643601 | 2.54909785 | 0.01274681 | 0.99994333 | -4.036979 | 2.49076594 |
| PGP | -0.2144681 | 5.2412862 | -2.5484416 | 0.01278127 | 0.99994333 | -4.0368131 | -2.4898064 |
| UBE2M | -0.2923905 | 6.67128176 | -2.5427449 | 0.01289487 | 0.99994333 | -4.0433419 | -2.4866598 |
| LINC01435 | -1.7616888 | -5.9376075 | -2.5380992 | 0.01309644 | 0.99994333 | -4.0645139 | -2.481136 |
| ANXA8 | -2.1442896 | -6.0884374 | -2.5276499 | 0.01324276 | 0.99994333 | -4.0087893 | -2.4771734 |
| NAT14 | -0.3780144 | 5.73208621 | -2.5332087 | 0.0132524 | 0.99994333 | -4.0607365 | -2.4769134 |
| ZNF546 | 0.37145298 | 3.6320044 | 2.53142775 | 0.01326807 | 0.99994333 | -4.030811 | 2.47649178 |
| ATP5MK | -0.3897452 | 5.14341796 | -2.5274809 | 0.01341928 | 0.99994333 | -4.0666605 | -2.4724439 |
| DYNLRB2-AS1 | -1.6608163 | -3.193434 | -2.5247217 | 0.01353858 | 0.99994333 | -4.1121642 | -2.4692786 |
| MAL2-AS1 | 2.16294502 | -5.4481785 | 2.51977035 | 0.01369648 | 0.99994333 | -4.0560339 | 2.46512662 |
| UBE2J2 | -0.2175776 | 5.9667725 | -2.5158289 | 0.01383891 | 0.99994333 | -4.0515401 | -2.4614178 |
| THAP12P3 | -2.0680035 | -6.0408799 | -2.5122383 | 0.01393643 | 0.99994333 | -4.0749363 | -2.4588977 |
| HMGN1P37 | -2.0020166 | -3.7661629 | -2.5088301 | 0.01407172 | 0.99994333 | -4.0997655 | -2.4554273 |
| PSME2P5 | 2.28210796 | -5.7697625 | 2.50649413 | 0.01421019 | 0.99994333 | -4.0443516 | 2.4519058 |
| KLF16 | -0.499241 | 4.08448516 | -2.5058079 | 0.01428824 | 0.99994333 | -4.0978677 | -2.449934 |
| SBNO1 | 0.24312314 | 6.20672785 | 2.50106808 | 0.01443682 | 0.99994333 | -4.0633489 | 2.44620682 |
| NPM3 | -0.3493372 | 3.64474773 | -2.5003834 | 0.01446495 | 0.99994333 | -4.0689513 | -2.4455049 |
| STK24-AS1 | -0.8697542 | -0.8418074 | -2.4963558 | 0.01458516 | 0.99994333 | -4.0692749 | -2.442519 |
| ATP6V1G1 | -0.307326 | 6.37100105 | -2.4953612 | 0.01461576 | 0.99994333 | -4.0757089 | -2.4417624 |
| RNU6-701P | 1.86170547 | -6.2110062 | 2.48734989 | 0.0148329 | 0.99994333 | -4.0329748 | 2.4364335 |
| SNHG7 | -0.336077 | 5.14920406 | -2.4862645 | 0.01499401 | 0.99994333 | -4.0742058 | -2.4325236 |
| POMP | -0.2890563 | 5.83276475 | -2.4826293 | 0.01506856 | 0.99994333 | -4.0812381 | -2.430727 |
| C1orf122 | -0.3450087 | 5.80335167 | -2.4842476 | 0.01511162 | 0.99994333 | -4.0892266 | -2.4296929 |
| MIR4441 | 1.90423004 | -5.619978 | 2.47664258 | 0.01514271 | 0.99994333 | -4.0851482 | 2.42894788 |
| QRSL1P3 | 2.01298744 | -4.0065355 | 2.48219031 | 0.01519269 | 0.99994333 | -4.0683216 | 2.42775297 |
| TMEM141 | -0.3593638 | 2.36485864 | -2.4768369 | 0.01536014 | 0.99994333 | -4.0904124 | -2.4237745 |
| CSTB | -0.3599452 | 5.09256469 | -2.4771853 | 0.01536075 | 0.99994333 | -4.097618 | -2.4237601 |
| INTS6L-AS1 | -2.1433407 | -4.7458547 | -2.4698502 | 0.015414 | 0.99994333 | -4.0726388 | -2.4225031 |
| PROP1 | -1.7254544 | -6.2230586 | -2.472522 | 0.01549259 | 0.99994333 | -4.0741724 | -2.4206547 |
| JMJD1C | 0.24064774 | 6.90028186 | 2.47188002 | 0.01555542 | 0.99994333 | -4.0792567 | 2.41918275 |
| MIR93 | -1.4863992 | -5.5861083 | -2.470577 | 0.01558424 | 0.99994333 | -4.070157 | -2.4185095 |
| SLC25A31 | 1.95430156 | -4.673406 | 2.46893172 | 0.01576778 | 0.99994333 | -4.047598 | 2.41424666 |
| CYC1 | -0.2686279 | 6.50862977 | -2.4616261 | 0.0160072 | 0.99994333 | -4.0819126 | -2.4087513 |
| INHBC | -0.5575571 | 0.11478396 | -2.4621031 | 0.01601454 | 0.99994333 | -4.0910397 | -2.408584 |
| ZNF195 | 0.23907261 | 5.89707543 | 2.46003427 | 0.01604985 | 0.99994333 | -4.0821974 | 2.40777993 |
| LINC01996 | -1.474109 | -6.581205 | -2.4502015 | 0.01642637 | 0.99994333 | -4.0717398 | -2.3993019 |
| PET117 | -1.0223452 | -2.374642 | -2.4460976 | 0.01652622 | 0.99994333 | -4.0919917 | -2.3970822 |
| RPL7AP64 | -2.4707154 | -5.7231227 | -2.4469744 | 0.01653159 | 0.99994333 | -4.1068918 | -2.3969631 |
| PHF10P1 | -1.7829478 | -5.1148365 | -2.4437679 | 0.01660198 | 0.99994333 | -4.0535362 | -2.3954057 |
| SLIRP | -0.4362097 | 4.30474919 | -2.4460939 | 0.01664686 | 0.99994333 | -4.0976851 | -2.3944159 |
| RBAK | 0.21395194 | 5.15562928 | 2.44366302 | 0.01673247 | 0.99994333 | -4.0786993 | 2.39253412 |
| LACTB2-AS1 | 1.21724654 | -2.4698118 | 2.44312603 | 0.0167589 | 0.99994333 | -4.0620834 | 2.39195502 |
| LCORL | 0.20470256 | 4.74948524 | 2.44282292 | 0.01677752 | 0.99994333 | -4.0979508 | 2.39154729 |
| CSPG4P5 | -1.3472103 | -6.1264355 | -2.4414843 | 0.01678965 | 0.99994333 | -4.0832443 | -2.391282 |
| DNPH1 | -0.4978284 | 4.27930936 | -2.4424194 | 0.01682357 | 0.99994333 | -4.1176056 | -2.3905409 |
| CCDC186 | 0.2757692 | 5.52778155 | 2.4396148 | 0.01695818 | 0.99994333 | -4.0833676 | 2.38761325 |
| MAP6D1 | -0.3373541 | 1.39034065 | -2.4385509 | 0.01699057 | 0.99994333 | -4.0941751 | -2.3869117 |
| ACTN3 | 1.01730947 | -1.7905564 | 2.44246028 | 0.01701774 | 0.99994333 | -4.0666566 | 2.38632421 |
| POLR2I | -0.574785 | 2.6396626 | -2.4350835 | 0.01705901 | 0.99994333 | -4.1128769 | -2.3854334 |
| RPS4XP4 | 1.94733897 | -5.5261305 | 2.4307526 | 0.01706465 | 0.99994333 | -4.0571422 | 2.38531187 |
| CENPX | -0.2756715 | 4.84473182 | -2.436392 | 0.01709357 | 0.99994333 | -4.1010685 | -2.3846889 |
| SPINK1 | -2.1042001 | -5.3196384 | -2.4349653 | 0.01713192 | 0.99994333 | -4.1497987 | -2.3838642 |
| RPL41 | -0.4847525 | 7.46728399 | -2.432232 | 0.01720341 | 0.99994333 | -4.1073744 | -2.3823314 |
| MCIDAS | -1.3081791 | -5.9945075 | -2.4290937 | 0.0173365 | 0.99994333 | -4.0491024 | -2.3794924 |
| POU2F1 | 0.29821612 | 6.39726192 | 2.42738253 | 0.01739788 | 0.99994333 | -4.0966831 | 2.3781896 |
| MIR331 | 1.4252237 | -3.9246236 | 2.4233239 | 0.01750875 | 0.99994333 | -4.0853142 | 2.37584637 |
| HMBS | -0.2677464 | 4.48791833 | -2.426398 | 0.01753059 | 0.99994333 | -4.1018896 | -2.3753864 |
| SH2D1A | -1.953769 | -5.507607 | -2.4227855 | 0.01765094 | 0.99994333 | -4.1250183 | -2.3728602 |
| POLR2F | -0.3795731 | 5.1276048 | -2.4211687 | 0.01770235 | 0.99994333 | -4.1160445 | -2.3717859 |
| MIR1-1HG | -1.6667746 | -6.4445989 | -2.4202768 | 0.01772812 | 0.99994333 | -4.1083463 | -2.3712481 |
| MPC1L | 1.91486869 | -6.0844498 | 2.41906915 | 0.01778059 | 0.99994333 | -4.0954729 | 2.37015573 |
| SPINK7 | 2.03056918 | -5.814452 | 2.41182392 | 0.01793713 | 0.99994333 | -4.0936101 | 2.36691335 |
| SPCS1 | -0.2521238 | 5.983138 | -2.4164064 | 0.01798138 | 0.99994333 | -4.1132992 | -2.3660013 |
| C10orf95 | -1.8847941 | -5.5960354 | -2.412315 | 0.01809182 | 0.99994333 | -4.1567934 | -2.3637335 |
| SNRPF | -0.3049861 | 5.08645913 | -2.4127678 | 0.0181083 | 0.99994333 | -4.1146115 | -2.3633961 |
| RPL7P47 | -2.3547447 | -4.983565 | -2.4100882 | 0.01826191 | 0.99994333 | -4.1524858 | -2.3602644 |
| RAC1P2 | -0.5878508 | -0.4601703 | -2.4088465 | 0.01833893 | 0.99994333 | -4.1280261 | -2.358703 |
| SRP14P1 | -1.727248 | -4.9865105 | -2.4000556 | 0.01846483 | 0.99994333 | -4.0780214 | -2.3561627 |
| RPL7P23 | -1.2760153 | -1.6214434 | -2.405231 | 0.01847067 | 0.99994333 | -4.1347515 | -2.3560452 |
| FAM192BP | -0.8528147 | -2.1135999 | -2.4045292 | 0.01849084 | 0.99994333 | -4.1056079 | -2.3556397 |
| HSD17B10 | -0.2597665 | 5.48330459 | -2.4054423 | 0.01850035 | 0.99994333 | -4.1247347 | -2.3554487 |
| OXLD1 | -0.2974338 | 4.27816335 | -2.4053307 | 0.018504 | 0.99994333 | -4.1117353 | -2.3553754 |
| NDUFS3 | -0.2712773 | 5.88009748 | -2.4029975 | 0.01851341 | 0.99994333 | -4.1118045 | -2.3551865 |
| SNHG9 | -1.0826115 | -0.5612129 | -2.4091312 | 0.01879234 | 0.99994333 | -4.1163852 | -2.3496249 |
| CNTNAP3P5 | 1.69621146 | -5.0549357 | 2.39459231 | 0.01893641 | 0.99994333 | -4.0907424 | 2.34678051 |
| H3P4 | 1.68169146 | -5.4344188 | 2.39418623 | 0.01901119 | 0.99994333 | -4.1008092 | 2.34531141 |
| MED15P8 | -1.7975203 | -6.1353796 | -2.391408 | 0.01909614 | 0.99994333 | -4.1560737 | -2.3436488 |
| TMEM135 | 0.20695456 | 4.13782883 | 2.3905255 | 0.01917154 | 0.99994333 | -4.1043221 | 2.34217863 |
| SPDEF | -0.6021563 | -0.1114341 | -2.3888066 | 0.01922494 | 0.99994333 | -4.0835952 | -2.3411402 |
| MRPL57 | -0.2427657 | 5.51959564 | -2.3869123 | 0.01936232 | 0.99994333 | -4.128521 | -2.3384807 |
| BRI3P1 | 1.92379032 | -4.6383609 | 2.38488036 | 0.01937477 | 0.99994333 | -4.1150576 | 2.33824063 |
| RN7SL704P | 1.59742615 | -4.0008623 | 2.38444288 | 0.01941019 | 0.99994333 | -4.1052468 | 2.33755803 |
| RNU6-48P | 1.60807435 | -3.8407233 | 2.38269549 | 0.01943045 | 0.99994333 | -4.1383135 | 2.33716795 |
| NDUFC1 | -0.3499578 | 4.70972288 | -2.3839932 | 0.01943056 | 0.99994333 | -4.1299108 | -2.3371658 |
| KLRK1 | -1.5810465 | -6.5011644 | -2.3830957 | 0.0194403 | 0.99994333 | -4.0959411 | -2.3369785 |
| EIF6 | -0.3384743 | 4.8748651 | -2.3850848 | 0.01944671 | 0.99994333 | -4.129599 | -2.3368552 |
| PELP1-DT | 1.35417951 | -3.8195012 | 2.3786273 | 0.01969795 | 0.99994333 | -4.1302733 | 2.33205208 |
| RPL7P32 | -2.2495553 | -4.1943948 | -2.3740587 | 0.01975539 | 0.99994333 | -4.134284 | -2.3309615 |
| LAMTOR4 | -0.2846246 | 5.21860871 | -2.3771036 | 0.01991912 | 0.99994333 | -4.1326502 | -2.3278679 |
| CCL28 | -1.8997946 | -3.2793496 | -2.3753573 | 0.01997548 | 0.99994333 | -4.175013 | -2.3268082 |
| TMSB10P1 | -2.1205357 | -4.7055204 | -2.371125 | 0.0201663 | 0.99994333 | -4.1634925 | -2.3232394 |
| LINC02154 | -2.3359352 | -5.9890575 | -2.3736416 | 0.02017041 | 0.99994333 | -4.1369298 | -2.3231628 |
| BZW1P2 | -0.5410907 | -0.2365407 | -2.3688602 | 0.02018722 | 0.99994333 | -4.1676611 | -2.3228498 |
| FAM207BP | -2.1244841 | -5.5448379 | -2.3683461 | 0.02023811 | 0.99994333 | -4.1549361 | -2.3219039 |
| MRPS26 | -0.2926929 | 5.43720142 | -2.3688159 | 0.02025475 | 0.99994333 | -4.1292962 | -2.321595 |
| COX7A2 | -0.3185202 | 6.26569686 | -2.3673363 | 0.02027872 | 0.99994333 | -4.1299469 | -2.3211506 |
| GLRX3P2 | -2.172992 | -4.9064392 | -2.3663418 | 0.02032142 | 0.99994333 | -4.1293084 | -2.3203598 |
| SPIN2A | -1.1235319 | -2.2498342 | -2.3647405 | 0.02039123 | 0.99994333 | -4.1563564 | -2.3190702 |
| TMEM126A | -0.277051 | 3.83364793 | -2.3647039 | 0.02043101 | 0.99994333 | -4.1525498 | -2.318337 |
| SRP9P1 | -0.5903881 | -0.1205049 | -2.3635842 | 0.02043297 | 0.99994333 | -4.1425749 | -2.3183009 |
| GFUS | -0.2432086 | 5.05679108 | -2.3647483 | 0.02044385 | 0.99994333 | -4.1436129 | -2.3181006 |
| CCDC124 | -0.3366816 | 5.92270059 | -2.3643829 | 0.02044742 | 0.99994333 | -4.1317476 | -2.3180351 |
| ATP5F1E | -0.509966 | 6.41964008 | -2.3629381 | 0.02046593 | 0.99994333 | -4.1378641 | -2.3176946 |
| GIT2 | 0.20577658 | 4.73944162 | 2.36382629 | 0.02054526 | 0.99994333 | -4.1158123 | 2.31623837 |
| MALSU1 | -0.2425536 | 3.86955338 | -2.3649753 | 0.02057119 | 0.99994333 | -4.1290466 | -2.3157633 |
| RPL21P34 | 1.75345153 | -4.4731689 | 2.35938646 | 0.02065323 | 0.99994333 | -4.1153417 | 2.3142642 |
| RPS19BP1 | -0.3006392 | 5.3432213 | -2.3584071 | 0.02072507 | 0.99994333 | -4.1356699 | -2.3129559 |
| RNU6-8 | -2.1580317 | -4.6830367 | -2.3536331 | 0.02090694 | 0.99994333 | -4.1109579 | -2.3096608 |
| CALHM3 | 1.71634679 | -4.5483572 | 2.35455424 | 0.02101515 | 0.99994333 | -4.0927102 | 2.30771224 |
| PRPF18 | 0.20367846 | 5.2875866 | 2.35089982 | 0.02119526 | 0.99994333 | -4.1301046 | 2.30448808 |
| ERH | -0.3257749 | 6.30028932 | -2.3498393 | 0.0212024 | 0.99994333 | -4.1432987 | -2.3043608 |
| MYLKP1 | 1.84040691 | -4.2723416 | 2.35107063 | 0.0212443 | 0.99994333 | -4.0981963 | 2.30361443 |
| PRADC1P1 | 1.79868607 | -3.5566296 | 2.35214527 | 0.02129332 | 0.99994333 | -4.1453421 | 2.30274276 |
| RPS12 | -0.3604506 | 7.64259345 | -2.3469587 | 0.02138534 | 0.99994333 | -4.1476303 | -2.3011113 |
| PICSAR | -1.6221017 | -6.1718027 | -2.3455112 | 0.02140423 | 0.99994333 | -4.1370833 | -2.3007772 |
| C6orf226 | -0.456194 | 3.33788335 | -2.3457681 | 0.02157073 | 0.99994333 | -4.1377752 | -2.297843 |
| PAXX | -0.2821821 | 4.95077077 | -2.3431583 | 0.02165266 | 0.99994333 | -4.1401381 | -2.2964064 |
| HCFC1R1 | -0.3140529 | 5.14673539 | -2.3408629 | 0.02169359 | 0.99994333 | -4.15028 | -2.2956903 |
| SBNO1-AS1 | -0.5844799 | 0.82394298 | -2.340516 | 0.02171599 | 0.99994333 | -4.1311903 | -2.2952991 |
| ACAP2 | 0.21332406 | 5.96422421 | 2.34049796 | 0.02178117 | 0.99994333 | -4.1427893 | 2.29416235 |
| GABARAP | -0.530112 | -0.3795087 | -2.3390421 | 0.02182334 | 0.99994333 | -4.1523549 | -2.2934285 |
| ITPA | -0.2850474 | 5.1867142 | -2.3392936 | 0.02189584 | 0.99994333 | -4.138838 | -2.2921699 |
| LARP1P1 | 1.80345041 | -5.9628921 | 2.33643248 | 0.02189855 | 0.99994333 | -4.1391992 | 2.29212286 |
| DMP1 | 1.95221093 | -4.0734932 | 2.33829835 | 0.02193699 | 0.99994333 | -4.1396661 | 2.29145716 |
| LINC02674 | -1.1559562 | -5.850659 | -2.3360388 | 0.02196272 | 0.99994333 | -4.1274625 | -2.291012 |
| API5P1 | -1.475383 | -3.2469408 | -2.3335926 | 0.02197674 | 0.99994333 | -4.1615465 | -2.2907696 |
| HSPA8P1 | -1.259919 | -0.9850399 | -2.3344774 | 0.02198179 | 0.99994333 | -4.1640574 | -2.2906824 |
| KLF1 | -1.9109339 | -3.0566273 | -2.3336852 | 0.02207755 | 0.99994333 | -4.1906464 | -2.289031 |
| TUBG1 | -0.2539545 | 5.22185874 | -2.3332689 | 0.02214963 | 0.99994333 | -4.1487621 | -2.2877921 |
| LINC01610 | -1.5133325 | -6.4679452 | -2.3313395 | 0.02216764 | 0.99994333 | -4.1451687 | -2.2874831 |
| KANSL1L | 0.22647231 | 4.97712352 | 2.33321897 | 0.02219275 | 0.99994333 | -4.1427201 | 2.28705267 |
| MIR1286 | 1.77088281 | -5.9611233 | 2.33066869 | 0.02229289 | 0.99994333 | -4.1179315 | 2.2853402 |
| NR2F6 | -0.2673229 | 4.92218332 | -2.3296929 | 0.02233049 | 0.99994333 | -4.1593159 | -2.2846988 |
| ZNF527 | 0.22406146 | 3.94227606 | 2.33040806 | 0.02233953 | 0.99994333 | -4.1327659 | 2.2845449 |
| RN7SL809P | 1.52179245 | -3.2799702 | 2.32776439 | 0.02234383 | 0.99994333 | -4.1231105 | 2.28447155 |
| TPSP2 | 1.80404786 | -5.2896072 | 2.32352631 | 0.02244306 | 0.99994333 | -4.1338826 | 2.28278464 |
| MED4 | -0.2277755 | 4.76460338 | -2.3259445 | 0.0225122 | 0.99994333 | -4.1495183 | -2.281613 |
| PRDX3 | -0.2126744 | 6.58303781 | -2.3256701 | 0.02258271 | 0.99994333 | -4.1554951 | -2.2804214 |
| CTSLP4 | 1.50820809 | -5.505381 | 2.32437287 | 0.02260517 | 0.99994333 | -4.1565891 | 2.28004252 |
| TMPRSS11F | 1.7479932 | -4.8615002 | 2.31738643 | 0.02295177 | 0.99994333 | -4.1330939 | 2.27423648 |
| MYL6 | -0.2734444 | 7.98848046 | -2.3186604 | 0.02296388 | 0.99994333 | -4.1580444 | -2.2740351 |
| NDUFA2 | -0.4236352 | 3.76348236 | -2.3139026 | 0.02298015 | 0.99994333 | -4.1593574 | -2.2737645 |
| PAPOLB | -1.8919749 | -4.474654 | -2.317675 | 0.02303809 | 0.99994333 | -4.1815086 | -2.2728024 |
| SDHAF2 | -0.3417093 | 2.84436855 | -2.3164497 | 0.02304262 | 0.99994333 | -4.1589475 | -2.2727272 |
| MRPL3P1 | -1.7430192 | -4.3651089 | -2.3093209 | 0.02322809 | 0.99994333 | -4.1668839 | -2.2696621 |
| ST3GAL1P1 | 1.81569626 | -4.5256427 | 2.31095859 | 0.0232624 | 0.99994333 | -4.1560489 | 2.26909739 |
| TAS2R5 | 0.72842333 | -0.5894868 | 2.31281564 | 0.02329198 | 0.99994333 | -4.141273 | 2.26861127 |
| RN7SL608P | 1.1547544 | -2.4999367 | 2.30998481 | 0.02344198 | 0.99994333 | -4.1533626 | 2.26615354 |
| ABHD17AP5 | -2.0637095 | -4.1867793 | -2.3061403 | 0.02344304 | 0.99994333 | -4.1601798 | -2.2661363 |
| PFDN6 | -0.422946 | 4.36056987 | -2.3087622 | 0.02349336 | 0.99994333 | -4.1701972 | -2.2653149 |
| KRT18P4 | 1.97668926 | -3.5990288 | 2.30431116 | 0.02352019 | 0.99994333 | -4.1551731 | 2.26487765 |
| IL1R2 | -2.0695658 | -4.6117774 | -2.3078198 | 0.02358351 | 0.99994333 | -4.1974847 | -2.2638473 |
| FARSA-AS1 | -1.9502213 | -4.7045368 | -2.3068667 | 0.02364229 | 0.99994333 | -4.1709124 | -2.2628929 |
| ZNF37A | 0.23988749 | 5.14613846 | 2.30628982 | 0.02366206 | 0.99994333 | -4.1476268 | 2.26257245 |
| CEACAM20 | 1.67657866 | -6.2601363 | 2.30377692 | 0.02370047 | 0.99994333 | -4.1253531 | 2.26195037 |
| CPHXL2 | 1.97237473 | -5.2532188 | 2.30125233 | 0.02372618 | 0.99994333 | -4.1295962 | 2.26153448 |
| MIR3677HG | -0.5825432 | 0.63991116 | -2.3059883 | 0.02372811 | 0.99994333 | -4.1510952 | -2.2615033 |
| RNF7 | -0.2673265 | 4.92279348 | -2.3038764 | 0.02374936 | 0.99994333 | -4.1667725 | -2.2611599 |
| RNA5SP345 | 1.58233321 | -5.5564298 | 2.30004026 | 0.02398468 | 0.99994333 | -4.1140378 | 2.25737448 |
| GDNF | 1.28726779 | -1.9527954 | 2.30110478 | 0.02401106 | 0.99994333 | -4.1615735 | 2.25695217 |
| RPL22P1 | -0.7135885 | -0.7027955 | -2.2996498 | 0.02407484 | 0.99994333 | -4.1869402 | -2.2559328 |
| CCAT2 | -1.5391309 | -6.1947271 | -2.2982897 | 0.02408187 | 0.99994333 | -4.1735968 | -2.2558205 |
| MPRIP-AS1 | -1.9815988 | -4.5243459 | -2.2944524 | 0.02413671 | 0.99994333 | -4.1706246 | -2.2549462 |
| ZNF568 | 0.21023816 | 4.28632169 | 2.29813497 | 0.0241866 | 0.99994333 | -4.155709 | 2.25415215 |
| RPL6P26 | -1.9062402 | -5.920159 | -2.2930367 | 0.02424201 | 0.99994333 | -4.158863 | -2.253272 |
| NAT8B | -2.0086147 | -5.3003192 | -2.2950462 | 0.02434833 | 0.99994333 | -4.181085 | -2.2515879 |
| LSM7 | -0.3165843 | 5.49847584 | -2.2937271 | 0.02441426 | 0.99994333 | -4.1654707 | -2.2505467 |
| NDUFS8 | -0.2598359 | 6.40540222 | -2.2945582 | 0.02443247 | 0.99994333 | -4.168573 | -2.2502597 |
| LINC02051 | -2.0168934 | -4.579846 | -2.2894462 | 0.02459983 | 0.99994333 | -4.2129306 | -2.2476295 |
| CLCC1 | 0.1520208 | 5.56081606 | 2.28974002 | 0.02468838 | 0.99994333 | -4.1656707 | 2.24624399 |
| TIMM13 | -0.3462161 | 5.48648893 | -2.2872574 | 0.02482348 | 0.99994333 | -4.1704495 | -2.2441387 |
| OAZ1 | -0.246229 | 7.67182739 | -2.282884 | 0.02484157 | 0.99994333 | -4.1647925 | -2.2438576 |
| CDC20-DT | -1.7860226 | -4.1920568 | -2.2844026 | 0.0248986 | 0.99994333 | -4.1515355 | -2.2429724 |
| LAMTOR5 | -0.319584 | 5.17333127 | -2.2854988 | 0.02493337 | 0.99994333 | -4.1745086 | -2.2424335 |
| SLC22A25 | 1.59129532 | -6.056162 | 2.28222625 | 0.0250321 | 0.99994333 | -4.1369335 | 2.24090695 |
| OR7E100P | 1.67821234 | -5.8759148 | 2.28174761 | 0.02506767 | 0.99994333 | -4.1475348 | 2.24035828 |
| IKBKE-AS1 | -1.7511488 | -3.1695652 | -2.2801202 | 0.02524844 | 0.99994333 | -4.1658165 | -2.2375802 |
| ATP6V1G1P4 | -1.8035035 | -5.9499509 | -2.2771084 | 0.02534056 | 0.99994333 | -4.1680708 | -2.2361711 |
| AP2S1 | -0.3153885 | 6.05113494 | -2.2762621 | 0.02546034 | 0.99994333 | -4.1773414 | -2.2343457 |
| HIGD1AP1 | -1.8074183 | -3.834494 | -2.2721029 | 0.02549697 | 0.99994333 | -4.2073162 | -2.2337889 |
| MRPS12 | -0.3735182 | 3.93824892 | -2.2742285 | 0.02558544 | 0.99994333 | -4.1738691 | -2.232447 |
| ID2B | -2.0035003 | -5.1139368 | -2.2701292 | 0.02560111 | 0.99994333 | -4.1720746 | -2.2322096 |
| UQCRFS1P3 | 1.75743467 | -5.4281713 | 2.27483304 | 0.02562127 | 0.99994333 | -4.1719825 | 2.23190455 |
| RN7SL68P | 1.75527376 | -5.1034376 | 2.27152825 | 0.02566356 | 0.99994333 | -4.183149 | 2.23126533 |
| SNORD127 | 1.56269586 | -3.9035963 | 2.26813811 | 0.02575785 | 0.99994333 | -4.1638357 | 2.22984321 |
| DYNLT1 | -0.2224208 | 6.06425952 | -2.2723761 | 0.02577008 | 0.99994333 | -4.1783583 | -2.2296591 |
| RPLP2 | -0.4681937 | 7.71704407 | -2.2711162 | 0.02581368 | 0.99994333 | -4.1801168 | -2.2290034 |
| RPL26P30 | -0.8552102 | -1.6270762 | -2.2695 | 0.02581856 | 0.99994333 | -4.1916023 | -2.2289301 |
| NDUFA4 | -0.3229371 | 7.04284214 | -2.2679713 | 0.02595557 | 0.99994333 | -4.1799613 | -2.2268759 |
| RARA | -0.2589463 | 5.41926344 | -2.2696549 | 0.02596325 | 0.99994333 | -4.1744588 | -2.226761 |
| ZNF141 | 0.35378928 | 5.22842249 | 2.26630856 | 0.02608749 | 0.99994333 | -4.1616854 | 2.22490694 |
| MLYCD | 0.32103639 | 3.90995426 | 2.26215294 | 0.02616334 | 0.99994333 | -4.1501664 | 2.22377859 |
| ANAPC11 | -0.3203092 | 5.63926471 | -2.2665529 | 0.02623096 | 0.99994333 | -4.1840628 | -2.2227752 |
| LINC02052 | -1.8227595 | -5.8386914 | -2.2637159 | 0.02632266 | 0.99994333 | -4.1873164 | -2.2214181 |
| RPL27AP | -1.6457767 | -5.5127441 | -2.2587923 | 0.02635536 | 0.99994333 | -4.1250188 | -2.2209351 |
| PDE6H | -1.8977746 | -4.9830876 | -2.2573818 | 0.02644486 | 0.99994333 | -4.1762379 | -2.2196158 |
| ADAM20 | 1.49498161 | -2.8211505 | 2.25660944 | 0.02647975 | 0.99994333 | -4.1278267 | 2.21910248 |
| NDUFB7 | -0.2941934 | 6.02148057 | -2.2597733 | 0.02650741 | 0.99994333 | -4.1816895 | -2.218696 |
| RPL21P125 | 0.89451964 | -1.3129408 | 2.25959186 | 0.02654073 | 0.99994333 | -4.1908696 | 2.21820691 |
| BMP2K | 0.1775596 | 4.40377404 | 2.26045852 | 0.02654078 | 0.99994333 | -4.1613823 | 2.21820614 |
| PSMA7 | -0.2762352 | 6.80472613 | -2.2605184 | 0.02654642 | 0.99994333 | -4.1835107 | -2.2181234 |
| RPL21P32 | 1.4961301 | -5.6098976 | 2.25869667 | 0.02655294 | 0.99994333 | -4.1338767 | 2.21802775 |
| SHARPIN | -0.1835893 | 5.3982821 | -2.2584836 | 0.02664679 | 0.99994333 | -4.1853941 | -2.2166533 |
| RNY4P19 | 1.32041896 | -3.2954838 | 2.2602253 | 0.02673371 | 0.99994333 | -4.1731365 | 2.21538415 |
| FAUP1 | -1.5639825 | -3.0349612 | -2.2556664 | 0.02679265 | 0.99994333 | -4.2183539 | -2.2145254 |
| GON7 | -0.2364059 | 3.18315486 | -2.256494 | 0.02679317 | 0.99994333 | -4.1912647 | -2.214518 |
| PTPN11P2 | 1.17399319 | -1.9473498 | 2.25506339 | 0.02682508 | 0.99994333 | -4.1539977 | 2.21405375 |
| RPL7AP70 | -1.2479368 | -1.9070906 | -2.2544266 | 0.02683354 | 0.99994333 | -4.2177108 | -2.2139308 |
| MAPK6P5 | -2.0043067 | -5.2353979 | -2.2509844 | 0.02683637 | 0.99994333 | -4.1640245 | -2.2138897 |
| ATP5PF | -0.2978545 | 4.98392698 | -2.2542513 | 0.02684006 | 0.99994333 | -4.191289 | -2.2138361 |
| RNU6-181P | 1.32705067 | -3.4425364 | 2.24890909 | 0.02700031 | 0.99994333 | -4.1741431 | 2.2115133 |
| REPIN1-AS1 | -0.3363291 | 1.08465272 | -2.2526784 | 0.02703301 | 0.99994333 | -4.1745355 | -2.2110409 |
| UPK3BL3P | -0.8270867 | -1.479548 | -2.2526767 | 0.02709346 | 0.99994333 | -4.1526044 | -2.2101687 |
| CFAP410 | -0.271244 | 4.27921563 | -2.2472256 | 0.02713712 | 0.99994333 | -4.1687513 | -2.2095398 |
| B3GAT3P1 | -1.6267829 | -3.566896 | -2.2496037 | 0.02713798 | 0.99994333 | -4.2006954 | -2.2095273 |
| MAPK6P4 | -1.7722237 | -4.2088609 | -2.2484546 | 0.02724925 | 0.99994333 | -4.2092714 | -2.2079287 |
| ABHD17AP4 | -2.1405041 | -5.7198607 | -2.2444644 | 0.02726832 | 0.99994333 | -4.2142911 | -2.2076552 |
| GRM3-AS1 | 0.57071196 | 0.07995774 | 2.24696963 | 0.02730061 | 0.99994333 | -4.14852 | 2.2071926 |
| CHCHD5 | -0.3585463 | 3.71645681 | -2.2458064 | 0.02751374 | 0.99994333 | -4.2052622 | -2.2041508 |
| GPS1 | -0.1216183 | 7.39631404 | -2.2413677 | 0.027752 | 0.99994333 | -4.1900049 | -2.2007743 |
| TSSC4 | -0.2287659 | 5.08567954 | -2.2403341 | 0.02777971 | 0.99994333 | -4.1867604 | -2.2003833 |
| CRIP2 | -0.2966488 | 7.35184044 | -2.2405673 | 0.02780951 | 0.99994333 | -4.1933377 | -2.1999632 |
| ATP5MGP8 | 1.597343 | -4.9818706 | 2.23965066 | 0.02783264 | 0.99994333 | -4.1703415 | 2.19963735 |
| RPS27A | -0.3004429 | 7.76241309 | -2.2392524 | 0.02788706 | 0.99994333 | -4.1951537 | -2.1988714 |
| PRDX5 | -0.3306586 | 7.23811361 | -2.2359824 | 0.0280682 | 0.99994333 | -4.1947844 | -2.1963319 |
| RPLP0P6 | -0.6925814 | 1.62808925 | -2.2362415 | 0.02808948 | 0.99994333 | -4.2322257 | -2.1960344 |
| DDTP1 | 1.5613163 | -3.8433833 | 2.23713369 | 0.02812289 | 0.99994333 | -4.1543092 | 2.19556788 |
| HIKESHI | -0.1783953 | 4.86446165 | -2.2321664 | 0.02835515 | 0.99994333 | -4.1979615 | -2.1923374 |
| ADGRG4 | 1.9425165 | -5.4181726 | 2.23190342 | 0.02837641 | 0.99994333 | -4.1472048 | 2.19204294 |
| METTL26 | -0.2315341 | 5.15017358 | -2.2324294 | 0.0284078 | 0.99994333 | -4.197039 | -2.1916083 |
| KPNA7 | 1.35078156 | -4.0926065 | 2.23178426 | 0.02844025 | 0.99994333 | -4.1713596 | 2.19115954 |
| COX7CP1 | -1.8466549 | -4.0247227 | -2.2281503 | 0.02860199 | 0.99994333 | -4.1912718 | -2.1889291 |
| AURKAIP1 | -0.3332193 | 5.80556303 | -2.2288899 | 0.02861026 | 0.99994333 | -4.1984716 | -2.1888154 |
| ATP5MC1 | -0.3134176 | 5.42643423 | -2.22597 | 0.02875375 | 0.99994333 | -4.1941103 | -2.1868463 |
| SETD5 | 0.15575608 | 7.99606487 | 2.22594014 | 0.02887204 | 0.99994333 | -4.1975111 | 2.18522931 |
| CYP2C9 | 1.49941569 | -5.4250132 | 2.2247321 | 0.02887846 | 0.99994333 | -4.1920235 | 2.18514163 |
| PLEKHJ1 | -0.2782587 | 5.63132769 | -2.224561 | 0.02891208 | 0.99994333 | -4.1969159 | -2.1846833 |
| HLTF | 0.19626727 | 6.31509285 | 2.22379401 | 0.02895834 | 0.99994333 | -4.1929128 | 2.18405325 |
| TAGAP | 0.95224039 | -2.343939 | 2.22260505 | 0.02897323 | 0.99994333 | -4.1687257 | 2.18385051 |
| TMSB4X | -0.2874401 | 10.3536351 | -2.2227447 | 0.02901661 | 0.99994333 | -4.1948488 | -2.1832608 |
| PIGCP1 | 0.29765584 | 2.04705564 | 2.22269503 | 0.02908074 | 0.99994333 | -4.183438 | 2.18239027 |
| FTLP6 | -1.8962897 | -6.0979418 | -2.2183726 | 0.02908642 | 0.99994333 | -4.1961046 | -2.1823132 |
| DUSP15 | -0.3141491 | 3.79364113 | -2.2233435 | 0.02908993 | 0.99994333 | -4.190663 | -2.1822658 |
| NDUFB4 | -0.2733564 | 5.89835203 | -2.223886 | 0.0291577 | 0.99994333 | -4.2006884 | -2.1813478 |
| CADM2-AS1 | 1.94806532 | -4.9385176 | 2.21706773 | 0.02916057 | 0.99994333 | -4.1708607 | 2.181309 |
| H2AZ2P1 | -0.4841718 | 0.07323875 | -2.2225189 | 0.02917803 | 0.99994333 | -4.1838844 | -2.1810728 |
| IL1RL2 | -1.9507516 | -4.1149077 | -2.2204485 | 0.02919934 | 0.99994333 | -4.1869129 | -2.1807847 |
| TOMM7 | -0.4918174 | 5.34348126 | -2.2227477 | 0.029234 | 0.99994333 | -4.1997894 | -2.1803166 |
| FAM153A | 1.02760437 | -1.866127 | 2.22035692 | 0.02926641 | 0.99994333 | -4.2130506 | 2.17987932 |
| CELF6 | 1.19625592 | -3.2436929 | 2.22068259 | 0.02932764 | 0.99994333 | -4.2037305 | 2.17905418 |
| SCAND1 | -0.3207272 | 5.85910706 | -2.2171654 | 0.02936368 | 0.99994333 | -4.2009527 | -2.1785692 |
| JAZF1-AS1 | -1.3405195 | -2.4198381 | -2.220406 | 0.02937527 | 0.99994333 | -4.1990885 | -2.1784134 |
| TRIML2 | -2.4553125 | -5.5104535 | -2.2199294 | 0.02939055 | 0.99994333 | -4.2256141 | -2.178208 |
| RAB5IF | -0.3597514 | 3.49689972 | -2.2175287 | 0.02948601 | 0.99994333 | -4.1984248 | -2.1769271 |
| ABCA15P | -2.3460745 | -4.2121888 | -2.2153331 | 0.02949223 | 0.99994333 | -4.2337065 | -2.1768437 |
| TMEM100 | -0.7963111 | 1.15426312 | -2.2149206 | 0.02959402 | 0.99994333 | -4.2728493 | -2.175482 |
| SUGT1P2 | 1.65479171 | -3.9667067 | 2.21761626 | 0.02963212 | 0.99994333 | -4.1841225 | 2.17497324 |
| SNRNP25 | -0.2269193 | 4.77802745 | -2.2150152 | 0.0296403 | 0.99994333 | -4.204207 | -2.1748641 |
| SCDP1 | -1.8949597 | -3.0378194 | -2.2152314 | 0.02969343 | 0.99994333 | -4.2411308 | -2.1741559 |
| COX5B | -0.3902158 | 6.41203139 | -2.212301 | 0.02970995 | 0.99994333 | -4.2028591 | -2.1739359 |
| KERA | 1.16504334 | -4.7501761 | 2.21220109 | 0.02980775 | 0.99994333 | -4.1851057 | 2.1726356 |
| PTPN2P1 | -1.7992588 | -5.6296205 | -2.21114 | 0.02983367 | 0.99994333 | -4.2094918 | -2.1722917 |
| GABPAP | -1.482641 | -4.2343439 | -2.2085468 | 0.03001284 | 0.99994333 | -4.1814726 | -2.1699209 |
| ZNF280B | 0.26217691 | 3.33906524 | 2.20768992 | 0.0301349 | 0.99994333 | -4.1991453 | 2.16831271 |
| DCLRE1C | 0.23994588 | 4.36578335 | 2.20840032 | 0.03021494 | 0.99994333 | -4.1880967 | 2.1672612 |
| C5orf58 | -1.8685671 | -4.6001992 | -2.2039442 | 0.03028768 | 0.99994333 | -4.1890404 | -2.1663077 |
| MYL12B | -0.2814703 | 6.78046408 | -2.2044952 | 0.03029545 | 0.99994333 | -4.2077051 | -2.166206 |
| POP7 | -0.3713941 | 4.49031613 | -2.2042983 | 0.03037398 | 0.99994333 | -4.2056958 | -2.165179 |
| EIF4A1P10 | -0.842452 | -0.0443194 | -2.205158 | 0.0303752 | 0.99994333 | -4.2178376 | -2.1651631 |
| MRPS34 | -0.3355692 | 4.82448368 | -2.2038049 | 0.03042893 | 0.99994333 | -4.208217 | -2.1644618 |
| ELOF1 | -0.280109 | 5.22487043 | -2.2039967 | 0.03044977 | 0.99994333 | -4.2056019 | -2.16419 |
| MRPL14 | -0.3113957 | 3.94089935 | -2.2022496 | 0.03055062 | 0.99994333 | -4.2146652 | -2.1628773 |
| SNF8 | -0.1511699 | 5.17658339 | -2.2014961 | 0.03062274 | 0.99994333 | -4.2012276 | -2.1619408 |
| ZNF182 | 0.19421581 | 3.53662879 | 2.2008444 | 0.03066199 | 0.99994333 | -4.1991755 | 2.16143189 |
| SERPINA10 | 1.43643931 | -3.1379991 | 2.20059353 | 0.03067178 | 0.99994333 | -4.201088 | 2.16130513 |
| FAM27B | -1.3677826 | -3.7722071 | -2.1990497 | 0.03072175 | 0.99994333 | -4.2583709 | -2.1606582 |
| UBE2SP2 | -1.5248281 | -2.3466198 | -2.1976011 | 0.03082339 | 0.99994333 | -4.2877631 | -2.1593452 |
| MORC3 | 0.16687705 | 5.38537905 | 2.19848027 | 0.03083286 | 0.99994333 | -4.2036817 | 2.15922308 |
| TSPAN8 | -1.7119178 | -5.0840342 | -2.1956251 | 0.03096702 | 0.99994333 | -4.1918234 | -2.1574963 |
| CYB5D1 | -0.4948634 | -1.5300164 | -2.1974719 | 0.03098047 | 0.99994333 | -4.1901059 | -2.1573235 |
| DDX50P2 | -1.9177312 | -5.5026326 | -2.194101 | 0.03107113 | 0.99994333 | -4.1765762 | -2.1561606 |
| FAU | -0.3189674 | 6.67741015 | -2.1933743 | 0.03114719 | 0.99994333 | -4.2150339 | -2.1551872 |
| PCMTD1 | 0.16502611 | 5.67078164 | 2.19394469 | 0.0311651 | 0.99994333 | -4.2061347 | 2.15495824 |
| BICD1P1 | 1.83128257 | -5.8327326 | 2.18917562 | 0.03118915 | 0.99994333 | -4.2020706 | 2.15465111 |
| MCRIP1 | -0.2954291 | 6.78351246 | -2.1916984 | 0.03129183 | 0.99994333 | -4.2101169 | -2.1533417 |
| RPL8P4 | -2.0541137 | -5.5697281 | -2.1899785 | 0.03132495 | 0.99994333 | -4.2122612 | -2.1529202 |
| PTPA | -0.2393925 | 6.85859204 | -2.1887184 | 0.03147544 | 0.99994333 | -4.2149428 | -2.1510096 |
| RPS21-DT | -0.3215883 | 1.71675038 | -2.1883219 | 0.03154884 | 0.99994333 | -4.2091888 | -2.1500807 |
| GAU1 | 0.54400019 | -0.1062825 | 2.18611168 | 0.03169145 | 0.99994333 | -4.1851627 | 2.14828084 |
| KRT77 | -1.6232567 | -5.6255789 | -2.1837982 | 0.03193029 | 0.99994333 | -4.2260369 | -2.1452822 |
| RPL21P116 | -1.7381899 | -5.6111394 | -2.178637 | 0.03224763 | 0.99994333 | -4.1837558 | -2.1413275 |
| RPL5P1 | -1.0174995 | -0.5174101 | -2.1791589 | 0.03228907 | 0.99994333 | -4.2435302 | -2.1408136 |
| CBR1-AS1 | 1.4092191 | -4.995221 | 2.17738525 | 0.03235511 | 0.99994333 | -4.1941804 | 2.13999571 |
| UBE2V1P1 | -1.9863557 | -5.1699942 | -2.1720584 | 0.03250047 | 0.99994333 | -4.2051094 | -2.1382006 |
| RBM41 | 0.2213321 | 4.77409761 | 2.17556873 | 0.03256572 | 0.99994333 | -4.2136716 | 2.1373969 |
| FAM3B | 1.65166574 | -6.3654878 | 2.17396914 | 0.03258217 | 0.99994333 | -4.2033825 | 2.13719456 |
| BBLN | -0.362982 | 6.33561712 | -2.1727963 | 0.032677 | 0.99994333 | -4.2182133 | -2.1360296 |
| PPP4C | -0.2467887 | 6.29475913 | -2.1742036 | 0.03270544 | 0.99994333 | -4.2220762 | -2.1356808 |
| AAMP | -0.2645718 | 6.73775121 | -2.1717922 | 0.03275092 | 0.99994333 | -4.2164871 | -2.1351236 |
| COX6A1 | -0.4105796 | 4.64886855 | -2.1705696 | 0.03281024 | 0.99994333 | -4.2240912 | -2.1343977 |
| LINC00671 | -1.5072205 | -5.8908377 | -2.1693095 | 0.03293871 | 0.99994333 | -4.1885146 | -2.1328296 |
| REX1BD | -0.2548974 | 5.43247318 | -2.1700043 | 0.03302363 | 0.99994333 | -4.2198236 | -2.1317958 |
| SIDT1 | 0.42849621 | 2.45347349 | 2.16946666 | 0.03304104 | 0.99994333 | -4.2139232 | 2.1315842 |
| CLEC4GP1 | -1.582226 | -6.0584179 | -2.1673956 | 0.03322678 | 0.99994333 | -4.2229069 | -2.1293322 |
| ADRM1 | -0.2198126 | 5.31087725 | -2.1650679 | 0.03329878 | 0.99994333 | -4.2175609 | -2.1284622 |
| RANBP1 | -0.2423699 | 6.44567696 | -2.1642384 | 0.03340699 | 0.99994333 | -4.2216737 | -2.1271577 |
| LVRN | 0.55869777 | 0.61719194 | 2.16396829 | 0.03340774 | 0.99994333 | -4.1876345 | 2.1271486 |
| ALKBH2 | -0.2405912 | 3.92574793 | -2.164061 | 0.03341291 | 0.99994333 | -4.2153067 | -2.1270863 |
| NEDD8 | -0.3944562 | 4.10781384 | -2.1625699 | 0.03343812 | 0.99994333 | -4.2357864 | -2.1267829 |
| RPS27AP11 | -1.865921 | -5.5609559 | -2.1631182 | 0.03352632 | 0.99994333 | -4.2226418 | -2.1257232 |
| RPS2P17 | -0.8879479 | -1.7749122 | -2.1631597 | 0.03353239 | 0.99994333 | -4.2635796 | -2.1256503 |
| GJB1 | -1.8396384 | -5.5483323 | -2.1612423 | 0.03366518 | 0.99994333 | -4.2229037 | -2.1240594 |
| MYO3B | 0.67638579 | 0.51144498 | 2.16069527 | 0.03368133 | 0.99994333 | -4.2181465 | 2.12386629 |
| PRKD3-DT | -1.5885026 | -5.4311707 | -2.1589631 | 0.03380286 | 0.99994333 | -4.1875371 | -2.1224155 |
| THEM7P | 1.95910784 | -5.7798501 | 2.1582936 | 0.03382928 | 0.99994333 | -4.2337983 | 2.12210078 |
| LINC02887 | 1.06675347 | -1.7421573 | 2.15847562 | 0.03384934 | 0.99994333 | -4.2205873 | 2.12186187 |
| EMD | -0.2245052 | 5.94423177 | -2.158898 | 0.03386469 | 0.99994333 | -4.2303278 | -2.1216791 |
| RNU6-1011P | 1.60821644 | -4.2408923 | 2.15429266 | 0.03393697 | 0.99994333 | -4.1953234 | 2.12081977 |
| RPL32P18 | -0.8122443 | -0.4942219 | -2.1546848 | 0.03419714 | 0.99994333 | -4.2537208 | -2.1177394 |
| RPL21P134 | -2.0141471 | -4.8640587 | -2.1510308 | 0.03421631 | 0.99994333 | -4.2331372 | -2.1175132 |
| RPL24 | -0.351634 | 7.58583878 | -2.1536019 | 0.03425332 | 0.99994333 | -4.2311167 | -2.1170769 |
| KCNJ15 | -1.820237 | -5.7018544 | -2.152157 | 0.03436669 | 0.99994333 | -4.2356661 | -2.1157428 |
| THAP11 | -0.2239269 | 4.82685873 | -2.1521747 | 0.03439852 | 0.99994333 | -4.2318402 | -2.1153689 |
| MUC20 | 0.4578337 | 3.42847021 | 2.15176756 | 0.03442704 | 0.99994333 | -4.2044808 | 2.11503414 |
| GPX4 | -0.2967264 | 7.39649469 | -2.1515871 | 0.03459916 | 0.99994333 | -4.2314846 | -2.1130187 |
| HCAR2 | 1.5877785 | -5.6244026 | 2.1491898 | 0.03463506 | 0.99994333 | -4.2217357 | 2.11259957 |
| TPM4P3 | -1.423121 | -3.5513593 | -2.1483399 | 0.03465101 | 0.99994333 | -4.243137 | -2.1124134 |
| TCEA2 | -0.174118 | 6.45193029 | -2.1493311 | 0.03466666 | 0.99994333 | -4.2297587 | -2.1122307 |
| PHPT1 | -0.2981114 | 4.97987384 | -2.1470075 | 0.03479905 | 0.99994333 | -4.2482155 | -2.110689 |
| CCDC12 | -0.3333763 | 4.47516386 | -2.1463633 | 0.03481127 | 0.99994333 | -4.2275813 | -2.110547 |
| H2AZP1 | 1.392681 | -6.3243489 | 2.14657318 | 0.03487808 | 0.99994333 | -4.2356575 | 2.10977109 |
| NDUFB3 | -0.3389628 | 4.2810344 | -2.1455166 | 0.03489749 | 0.99994333 | -4.2436187 | -2.1095459 |
| KLRB1 | -1.5366739 | -5.8906484 | -2.1447698 | 0.03492337 | 0.99994333 | -4.198777 | -2.1092458 |
| NHLRC2 | 0.2740359 | 5.77048144 | 2.1445722 | 0.03505318 | 0.99994333 | -4.2290437 | 2.10774357 |
| POLR2J | -0.3114305 | 4.76167493 | -2.1450063 | 0.03519571 | 0.99994333 | -4.2381426 | -2.1060995 |
| PHF5A | -0.2708033 | 4.53847591 | -2.1422084 | 0.03521952 | 0.99994333 | -4.2352346 | -2.1058254 |
| NUTF2 | -0.1754764 | 6.61830355 | -2.1436326 | 0.03523485 | 0.99994333 | -4.2350282 | -2.105649 |
| YDJC | -0.3303829 | 3.84724595 | -2.1428801 | 0.03524417 | 0.99994333 | -4.2293102 | -2.1055419 |
| SSBP4 | -0.2344483 | 6.72950651 | -2.1418663 | 0.03525962 | 0.99994333 | -4.2351997 | -2.1053641 |
| SLMAP | 0.13473663 | 5.7379287 | 2.13867593 | 0.03552678 | 0.99994333 | -4.2335744 | 2.10230253 |
| INKA1 | -0.5186105 | 0.7547545 | -2.137341 | 0.03565157 | 0.99994333 | -4.2369746 | -2.1008792 |
| MTND2P40 | 1.24365519 | -2.511451 | 2.13521603 | 0.03566726 | 0.99994333 | -4.201657 | 2.10070058 |
| TIMM8B | -0.3730759 | 4.89419625 | -2.1328775 | 0.03570093 | 0.99994333 | -4.2448922 | -2.1003174 |
| LINC01629 | 1.63253862 | -5.6142982 | 2.13335473 | 0.03582915 | 0.99994333 | -4.1861488 | 2.09886111 |
| SNRPC | -0.3078038 | 5.49394025 | -2.1347807 | 0.03583532 | 0.99994333 | -4.2397712 | -2.0987911 |
| HMBOX1 | 0.33770604 | 4.46678965 | 2.13211582 | 0.0360843 | 0.99994333 | -4.2251886 | 2.09597622 |
| PEX16 | -0.2036763 | 4.74698487 | -2.1304244 | 0.03615312 | 0.99994333 | -4.2340829 | -2.0952011 |
| VASP | -0.2726214 | 4.30194924 | -2.1307949 | 0.0361985 | 0.99994333 | -4.2468028 | -2.0946906 |
| RINT1 | 0.17919379 | 4.40612586 | 2.12855118 | 0.03629483 | 0.99994333 | -4.2239713 | 2.09360892 |
| RPS20P22 | 0.66425601 | -0.7462276 | 2.12885253 | 0.03634033 | 0.99994333 | -4.2303984 | 2.09309885 |
| ZFP57 | -1.4455509 | -6.1966081 | -2.1264966 | 0.03643612 | 0.99994333 | -4.2349221 | -2.0920268 |
| NDUFS6 | -0.4584023 | 5.27546375 | -2.1293049 | 0.03644667 | 0.99994333 | -4.2427147 | -2.0919088 |
| MARCHF6-DT | -0.2902008 | 1.9408432 | -2.1269156 | 0.03648466 | 0.99994333 | -4.2500046 | -2.0914844 |
| HCCS-DT | -0.7180927 | -1.590536 | -2.1262305 | 0.0365035 | 0.99994333 | -4.236681 | -2.0912741 |
| MIR7845 | -1.4046681 | -3.2405768 | -2.1262129 | 0.03663812 | 0.99994333 | -4.2545739 | -2.0897739 |
| MIR4766 | 1.74123824 | -5.203662 | 2.12661242 | 0.03669391 | 0.99994333 | -4.2375478 | 2.08915349 |
| FTH1P4 | -1.8795176 | -4.3621435 | -2.1223265 | 0.03675586 | 0.99994333 | -4.2444084 | -2.0884656 |
| KIF6 | 0.38944419 | 1.58994155 | 2.12326079 | 0.03684026 | 0.99994333 | -4.2456045 | 2.08752993 |
| TRIR | -0.2863033 | 6.92581336 | -2.122506 | 0.03684482 | 0.99994333 | -4.2416797 | -2.0874794 |
| PPP1R14B | -0.3088189 | 6.83294618 | -2.1210337 | 0.03698267 | 0.99994333 | -4.2425331 | -2.0859553 |
| FUT10 | 0.28625337 | 3.55216641 | 2.12156423 | 0.03698662 | 0.99994333 | -4.2331721 | 2.08591171 |
| KIAA1191P2 | 1.70174877 | -4.7719489 | 2.11818572 | 0.03699319 | 0.99994333 | -4.2389427 | 2.08583924 |
| CEP295 | 0.217067 | 4.42716861 | 2.1215528 | 0.03700773 | 0.99994333 | -4.2275573 | 2.08567876 |
| AIP | -0.264044 | 4.99975033 | -2.1205246 | 0.03703808 | 0.99994333 | -4.2450649 | -2.0853441 |
| MSRB1 | -0.2722978 | 3.81055262 | -2.1212922 | 0.03703813 | 0.99994333 | -4.2704585 | -2.0853435 |
| MIR6865 | -1.5383624 | -4.2936247 | -2.1197732 | 0.03706178 | 0.99994333 | -4.2504435 | -2.0850828 |
| HELZ-AS1 | -0.6730594 | -1.1910544 | -2.1163838 | 0.03711596 | 0.99994333 | -4.2202428 | -2.0844862 |
| DENND4A | 0.18237743 | 5.18279255 | 2.11903982 | 0.03716449 | 0.99994333 | -4.230854 | 2.08395244 |
| LINC01581 | -1.876772 | -5.8650554 | -2.117153 | 0.03727476 | 0.99994333 | -4.2326577 | -2.0827419 |
| C7orf50 | -0.3079913 | 6.14776907 | -2.1175608 | 0.03732626 | 0.99994333 | -4.2463189 | -2.0821775 |
| PFN1P2 | 0.6438631 | -0.0167298 | 2.11649098 | 0.03735754 | 0.99994333 | -4.2240222 | 2.08183502 |
| TMEM161B | 0.23675857 | 4.76989858 | 2.11664258 | 0.03740197 | 0.99994333 | -4.2321981 | 2.08134905 |
| CEP57 | 0.23544546 | 6.12543601 | 2.11621929 | 0.03740844 | 0.99994333 | -4.240758 | 2.08127825 |
| WDR54 | -0.2330056 | 5.25056103 | -2.1159215 | 0.03748336 | 0.99994333 | -4.2509312 | -2.08046 |
| CKS1B | -0.3456063 | 3.69623043 | -2.1144132 | 0.03762997 | 0.99994333 | -4.262328 | -2.0788628 |
| NEXN | 0.51060874 | 2.02324307 | 2.11411261 | 0.03765998 | 0.99994333 | -4.2274414 | 2.0785364 |
| PKP4-AS1 | 1.07012755 | -1.5952759 | 2.11242749 | 0.03766332 | 0.99994333 | -4.2407327 | 2.07850015 |
| LINC03072 | -0.2318309 | 5.48817133 | -2.1137851 | 0.03766872 | 0.99994333 | -4.2443927 | -2.0784414 |
| BLVRA | -0.2765799 | 4.63008703 | -2.1144082 | 0.03771845 | 0.99994333 | -4.2523884 | -2.0779014 |
| LINC01284 | 0.77196651 | -1.5078183 | 2.11386122 | 0.03780036 | 0.99994333 | -4.2478959 | 2.07701301 |
| TARID | 1.09354157 | -2.3558134 | 2.11232725 | 0.03781631 | 0.99994333 | -4.2562418 | 2.07684019 |
| RNU6-293P | 1.50985567 | -6.413323 | 2.10977907 | 0.03793014 | 0.99994333 | -4.2254432 | 2.07560886 |
| LINC02775 | -1.720796 | -6.2422309 | -2.1088335 | 0.03800395 | 0.99994333 | -4.2587432 | -2.0748122 |
| EDF1 | -0.3806733 | 6.84823559 | -2.1089911 | 0.03803546 | 0.99994333 | -4.2494227 | -2.0744724 |
| NAV2-IT1 | -1.6392977 | -5.7782263 | -2.1067856 | 0.03821229 | 0.99994333 | -4.2483653 | -2.0725703 |
| DRAP1 | -0.3215686 | 6.69407933 | -2.1069525 | 0.03821935 | 0.99994333 | -4.2507155 | -2.0724945 |
| MTND4P26 | 1.67208259 | -3.7141757 | 2.10640397 | 0.03821988 | 0.99994333 | -4.2591739 | 2.07248885 |
| PFN1P4 | 1.03865022 | -1.9546147 | 2.10552616 | 0.0382998 | 0.99994333 | -4.228828 | 2.0716317 |
| ACTA2-AS1 | 0.90496837 | -2.5437891 | 2.10622609 | 0.03830706 | 0.99994333 | -4.2168371 | 2.07155391 |
| HDHD3 | -0.219134 | 3.87925496 | -2.1050512 | 0.03835112 | 0.99994333 | -4.2460047 | -2.0710822 |
| PDE4DIPP1 | 1.08908967 | -3.0239782 | 2.10567355 | 0.03837089 | 0.99994333 | -4.2489104 | 2.07087061 |
| YWHAQP4 | -1.8492059 | -4.3739067 | -2.1041582 | 0.0383982 | 0.99994333 | -4.2651553 | -2.0705785 |
| GALK1 | -0.3491104 | 3.98345546 | -2.1058249 | 0.03840381 | 0.99994333 | -4.2591302 | -2.0705185 |
| MON2 | 0.15533836 | 6.51422739 | 2.10496946 | 0.03842887 | 0.99994333 | -4.2435523 | 2.07025069 |
| ZIM2 | 0.83374298 | -2.7159915 | 2.10457083 | 0.0384349 | 0.99994333 | -4.1743219 | 2.07018631 |
| JMY | 0.3109002 | 6.01669472 | 2.1038856 | 0.03847791 | 0.99994333 | -4.2455355 | 2.06972704 |
| ATP5ME | -0.5502028 | 1.82510007 | -2.1036735 | 0.03848316 | 0.99994333 | -4.2624783 | -2.069671 |
| ERO1B | 0.32157975 | 4.38277678 | 2.1029979 | 0.03854008 | 0.99994333 | -4.2354078 | 2.06906402 |
| PAX4 | 1.67270385 | -6.2585375 | 2.10434083 | 0.03857841 | 0.99994333 | -4.2423243 | 2.06865565 |
| HDAC3 | -0.1725638 | 5.86693892 | -2.1026692 | 0.03868288 | 0.99994333 | -4.2442415 | -2.0675445 |
| NTHL1 | -0.2237331 | 3.71491612 | -2.102261 | 0.03871587 | 0.99994333 | -4.2548497 | -2.0671941 |
| PKIG | -0.2294735 | 5.11325637 | -2.1018723 | 0.03878791 | 0.99994333 | -4.2527347 | -2.0664299 |
| RABAC1 | -0.4870915 | 2.39221805 | -2.0989381 | 0.03891231 | 0.99994333 | -4.2582546 | -2.065113 |
| PCBP1 | -0.4002519 | 3.71834019 | -2.0986171 | 0.03892447 | 0.99994333 | -4.2566704 | -2.0649845 |
| PHC3 | 0.25470686 | 6.46564681 | 2.09936515 | 0.03895037 | 0.99994333 | -4.2474879 | 2.06471085 |
| RPS2P5 | -0.4263817 | 6.37647533 | -2.0994339 | 0.03895709 | 0.99994333 | -4.2596116 | -2.0646399 |
| SLC66A2 | -0.2309116 | 6.14208906 | -2.0985652 | 0.03901543 | 0.99994333 | -4.2525503 | -2.0640241 |
| ZNF197 | 0.19363647 | 5.86182644 | 2.09868969 | 0.03902592 | 0.99994333 | -4.2503144 | 2.06391346 |
| PIM1 | -0.3167325 | 4.46731209 | -2.0971742 | 0.03910055 | 0.99994333 | -4.2452808 | -2.0631272 |
| UBA52 | -0.291183 | 7.5374416 | -2.0966004 | 0.03915801 | 0.99994333 | -4.2560169 | -2.0625226 |
| ZNF283 | 0.21976609 | 3.80248987 | 2.09499541 | 0.03928715 | 0.99994333 | -4.2312921 | 2.0611666 |
| GABRR3 | -1.8443756 | -5.4911642 | -2.0942595 | 0.03944245 | 0.99994333 | -4.2846083 | -2.0595409 |
| DCTPP1 | -0.2998242 | 4.30120775 | -2.092662 | 0.03953904 | 0.99994333 | -4.2682324 | -2.0585325 |
| RNU6-387P | 1.1710022 | -3.3908319 | 2.09141976 | 0.03958792 | 0.99994333 | -4.202447 | 2.05802303 |
| AIDAP2 | 1.65855704 | -3.9207102 | 2.08844836 | 0.03959827 | 0.99994333 | -4.2431654 | 2.05791524 |
| GAPVD1 | 0.15235269 | 6.50636686 | 2.0925406 | 0.03966687 | 0.99994333 | -4.2490184 | 2.05720123 |
| MRPL43 | -0.2419057 | 5.11217703 | -2.0906123 | 0.03981889 | 0.99994333 | -4.2556345 | -2.0556227 |
| PPIAP7 | 1.38021513 | -5.8441271 | 2.08843886 | 0.03983072 | 0.99994333 | -4.2179284 | 2.05550019 |
| ELP5 | -0.170528 | 5.17770658 | -2.0885191 | 0.03986162 | 0.99994333 | -4.2491393 | -2.05518 |
| ABHD17A | -0.1689444 | 7.1039686 | -2.0881951 | 0.04001598 | 0.99994333 | -4.2577204 | -2.053584 |
| CCDC88C-DT | -1.5917225 | -5.1222205 | -2.0872002 | 0.0400537 | 0.99994333 | -4.2620305 | -2.0531947 |
| ARPC1A | -0.2934221 | 3.63416412 | -2.0864108 | 0.04006292 | 0.99994333 | -4.2588653 | -2.0530995 |
| LINC02048 | -1.8682342 | -4.4191527 | -2.0861257 | 0.0402037 | 0.99994333 | -4.2912334 | -2.0516499 |
| TRIM68 | 0.16825337 | 4.26940598 | 2.08629967 | 0.04022432 | 0.99994333 | -4.2517276 | 2.05143792 |
| ZRANB2-DT | 0.41948472 | 0.63424832 | 2.083151 | 0.04033586 | 0.99994333 | -4.2562093 | 2.05029284 |
| HAVCR1 | 1.28667721 | -2.8740036 | 2.08397228 | 0.04038126 | 0.99994333 | -4.2392668 | 2.04982755 |
| NFKBIE | -0.2385297 | 3.64284021 | -2.0826356 | 0.04045729 | 0.99994333 | -4.2522422 | -2.0490494 |
| DDT | 0.64504325 | -0.7591436 | 2.08241378 | 0.04057676 | 0.99994333 | -4.2339018 | 2.04782898 |
| LINC01723 | -1.4497159 | -6.5135009 | -2.0796823 | 0.04068273 | 0.99994333 | -4.2523403 | -2.0467491 |
| CNIH2 | -0.2868063 | 7.50762523 | -2.0809037 | 0.04069103 | 0.99994333 | -4.2611704 | -2.0466646 |
| RPL9 | -0.3371607 | 7.90862453 | -2.0799628 | 0.04070739 | 0.99994333 | -4.2629046 | -2.0464981 |
| FAM209A | 1.35859197 | -3.9181435 | 2.07833553 | 0.04075271 | 0.99994333 | -4.2553146 | 2.04603722 |
| DAPK3 | -0.1826317 | 6.34676087 | -2.0803391 | 0.04076513 | 0.99994333 | -4.2596079 | -2.045911 |
| TNS1-AS1 | 1.96505042 | -4.4385655 | 2.0786795 | 0.04076621 | 0.99994333 | -4.2636721 | 2.04590004 |
| FBXO34-AS1 | -1.292604 | -3.56296 | -2.0792121 | 0.04078092 | 0.99994333 | -4.2863014 | -2.0457505 |
| TRMT112 | -0.3481435 | 5.34533532 | -2.0804794 | 0.0408177 | 0.99994333 | -4.2664351 | -2.0453771 |
| ARID2 | 0.23373136 | 6.01065407 | 2.07898073 | 0.04083007 | 0.99994333 | -4.2577251 | 2.04525148 |
| PRR4 | 1.20111107 | -2.0470474 | 2.07779127 | 0.04088664 | 0.99994333 | -4.2291542 | 2.04467778 |
| MIR4285 | 1.42384702 | -5.964975 | 2.07724534 | 0.0408914 | 0.99994333 | -4.2528671 | 2.04462949 |
| KLB | 0.62659419 | -0.7418142 | 2.07875846 | 0.04089307 | 0.99994333 | -4.2636197 | 2.04461253 |
| TAF1 | 0.14991158 | 6.11494909 | 2.0771434 | 0.04093417 | 0.99994333 | -4.2585586 | 2.04419622 |
| SNRPE | -0.2454285 | 5.01962381 | -2.0775806 | 0.0409611 | 0.99994333 | -4.267723 | -2.0439235 |
| RNF139-DT | 0.39729852 | 2.26895567 | 2.07907174 | 0.04101763 | 0.99994333 | -4.2396806 | 2.04335179 |
| DCP1B | 0.16228366 | 4.2569143 | 2.07741442 | 0.04103947 | 0.99994333 | -4.2584687 | 2.04313102 |
| LINC02549 | 1.43152854 | -4.0273704 | 2.07479454 | 0.04114249 | 0.99994333 | -4.2640128 | 2.04209117 |
| SPON1-AS1 | 1.26818772 | -4.170589 | 2.0712878 | 0.04123905 | 0.99994333 | -4.2583767 | 2.04111855 |
| COMMD4 | -0.2707572 | 5.34735828 | -2.0741877 | 0.04130796 | 0.99994333 | -4.2612745 | -2.0404255 |
| ATP9B | 0.13764953 | 5.11334022 | 2.07374432 | 0.04134091 | 0.99994333 | -4.2580708 | 2.04009459 |
| GNB2 | -0.2576595 | 8.39382347 | -2.0736579 | 0.04135057 | 0.99994333 | -4.2642607 | -2.0399976 |
| TMEM147 | -0.3364508 | 5.58304067 | -2.0737674 | 0.04138146 | 0.99994333 | -4.2667912 | -2.0396875 |
| HNRNPMP1 | -1.7112218 | -6.0021412 | -2.0721228 | 0.04138342 | 0.99994333 | -4.2725728 | -2.0396678 |
| ACSBG2 | 0.64765751 | -0.6903209 | 2.07061612 | 0.04156917 | 0.99994333 | -4.2472072 | 2.03780769 |
| BEX2 | -0.3167376 | 6.23192588 | -2.0710059 | 0.04163727 | 0.99994333 | -4.2626618 | -2.0371274 |
| POP5 | -0.2189901 | 4.39067597 | -2.0699773 | 0.04167383 | 0.99994333 | -4.2627795 | -2.0367627 |
| PAIP1P2 | -0.3088922 | 2.21317701 | -2.0682733 | 0.04178305 | 0.99994333 | -4.2767816 | -2.0356745 |
| STARD13-AS | -1.5450573 | -3.639498 | -2.0682321 | 0.04187661 | 0.99994333 | -4.279432 | -2.0347443 |
| ITGBL1 | -1.6738407 | -3.995869 | -2.0667076 | 0.04193853 | 0.99994333 | -4.2180299 | -2.0341296 |
| ZNF33A | 0.1724515 | 6.53824669 | 2.06554379 | 0.04213869 | 0.99994333 | -4.2627447 | 2.03214781 |
| FKTN | 0.15161785 | 5.86598848 | 2.06478115 | 0.04216714 | 0.99994333 | -4.2641773 | 2.03186686 |
| OGA | 0.19813079 | 8.20619322 | 2.06471041 | 0.04217798 | 0.99994333 | -4.2658792 | 2.03175978 |
| RNU2-69P | 1.46174885 | -4.3141073 | 2.06298287 | 0.04222104 | 0.99994333 | -4.2540419 | 2.03133488 |
| NHP2 | -0.3538592 | 5.16877947 | -2.0642607 | 0.04225046 | 0.99994333 | -4.271144 | -2.0310447 |
| ZNF638 | 0.19458738 | 7.43667844 | 2.06386413 | 0.04233723 | 0.99994333 | -4.2623629 | 2.03019002 |
| MAP2K3 | -0.2276599 | 4.3067623 | -2.0633231 | 0.04235235 | 0.99994333 | -4.2537423 | -2.0300412 |
| TMEM26-AS1 | -1.8343426 | -5.1593797 | -2.060833 | 0.04247002 | 0.99994333 | -4.2703214 | -2.0288848 |
| KCNMB3 | 0.42632006 | 0.90406173 | 2.06155426 | 0.04247742 | 0.99994333 | -4.2437279 | 2.02881224 |
| LINC01821 | 1.49243029 | -4.9110184 | 2.06087728 | 0.04253182 | 0.99994333 | -4.281505 | 2.02827865 |
| FGF12-AS2 | -1.6915397 | -4.0884831 | -2.060842 | 0.04259363 | 0.99994333 | -4.2999583 | -2.0276731 |
| VN1R1 | 0.36836157 | 1.59759813 | 2.06356801 | 0.0426657 | 0.99994333 | -4.2535773 | 2.02696791 |
| PDC | 0.89311032 | -1.8587259 | 2.05853548 | 0.04266927 | 0.99994333 | -4.2354001 | 2.026933 |
| TMIGD2 | -1.3106304 | -3.1147945 | -2.0577634 | 0.04276099 | 0.99994333 | -4.265162 | -2.026037 |
| PTPRD-DT | -1.2260384 | -2.4669917 | -2.0582622 | 0.04286384 | 0.99994333 | -4.2982453 | -2.0250343 |
| KBTBD12 | -1.5026 | -4.2376624 | -2.0559439 | 0.04297256 | 0.99994333 | -4.2686073 | -2.0239766 |
| BTLA | 1.35816426 | -3.4648797 | 2.05349709 | 0.04321188 | 0.99994333 | -4.2435132 | 2.02165618 |
| ZNF513 | -0.2447751 | 5.5775748 | -2.0535925 | 0.04324636 | 0.99994333 | -4.2675731 | -2.0213228 |
| ICAM3 | 0.93273217 | -2.5127525 | 2.05624061 | 0.04327793 | 0.99994333 | -4.2357854 | 2.02101776 |
| MRPL21 | -0.2582726 | 4.61452958 | -2.0524664 | 0.04332924 | 0.99994333 | -4.2658575 | -2.0205223 |
| CEP290 | 0.25421064 | 5.90974243 | 2.05259276 | 0.04337491 | 0.99994333 | -4.2651178 | 2.02008173 |
| ZNF891 | 0.38920328 | 4.70351872 | 2.05209459 | 0.04345292 | 0.99994333 | -4.2612454 | 2.01933009 |
| LINC02341 | -1.6748117 | -5.5117201 | -2.0500057 | 0.0434908 | 0.99994333 | -4.2342692 | -2.0189655 |
| LY6L | -1.3591926 | -6.0169035 | -2.0508814 | 0.04351271 | 0.99994333 | -4.2679356 | -2.0187548 |
| MRPS36P1 | -1.6894863 | -5.7324042 | -2.0473344 | 0.04355758 | 0.99994333 | -4.2484663 | -2.0183235 |
| MTRF1LP1 | 1.52056091 | -3.3370512 | 2.04742731 | 0.04360638 | 0.99994333 | -4.278876 | 2.01785489 |
| NDUFB2 | -0.2430606 | 4.63647428 | -2.0474892 | 0.04383391 | 0.99994333 | -4.2698721 | -2.0156756 |
| HMGA1P4 | -1.4262164 | -6.520677 | -2.0472121 | 0.04388282 | 0.99994333 | -4.2585911 | -2.0152084 |
| PIN1 | -0.2032653 | 6.59400766 | -2.0474213 | 0.04389694 | 0.99994333 | -4.2704872 | -2.0150736 |
| MTCO1P42 | 1.69618469 | -4.0293318 | 2.04630705 | 0.0440401 | 0.99994333 | -4.2645826 | 2.01370899 |
| UBE2B | -0.170543 | 5.64477953 | -2.0454575 | 0.04410275 | 0.99994333 | -4.2702818 | -2.0131129 |
| NDUFB6 | -0.2958531 | 4.91013312 | -2.0443117 | 0.04414759 | 0.99994333 | -4.2789665 | -2.0126868 |
| SURF2 | -0.2346012 | 3.9634697 | -2.0433266 | 0.04416313 | 0.99994333 | -4.2682242 | -2.0125392 |
| INO80D | 0.33838913 | 5.21372001 | 2.04315464 | 0.04426904 | 0.99994333 | -4.264954 | 2.01153437 |
| HSD17B2-AS1 | 1.4763296 | -3.9986312 | 2.03995885 | 0.04432773 | 0.99994333 | -4.2777121 | 2.0109785 |
| JMJD4 | -0.2372256 | 2.75653704 | -2.0400485 | 0.0445404 | 0.99994333 | -4.2753853 | -2.0089692 |
| KPTN | -0.2467524 | 3.3425987 | -2.041327 | 0.04455972 | 0.99994333 | -4.2752263 | -2.0087871 |
| ZC3H8 | 0.20030112 | 4.74689276 | 2.04095109 | 0.04458484 | 0.99994333 | -4.2679964 | 2.00855039 |
| MRPL12 | -0.4209668 | 1.73790353 | -2.0388642 | 0.04471339 | 0.99994333 | -4.2928337 | -2.0073408 |
| EID2 | -0.2663377 | 5.50478691 | -2.0387704 | 0.04482039 | 0.99994333 | -4.275409 | -2.0063362 |
| NUS1P3 | -1.7432028 | -5.9535794 | -2.0370876 | 0.04491225 | 0.99994333 | -4.2901249 | -2.0054754 |
| LINC02334 | 0.87681539 | -0.9956097 | 2.0378502 | 0.04491937 | 0.99994333 | -4.2933151 | 2.0054087 |
| DPCD | -0.2336205 | 4.44391179 | -2.0374524 | 0.04493089 | 0.99994333 | -4.2823947 | -2.0053009 |
| COX8A | -0.4047279 | 4.52210708 | -2.0368606 | 0.04493625 | 0.99994333 | -4.298587 | -2.0052508 |
| SMYD4 | 0.14671856 | 4.75066892 | 2.03654801 | 0.04502104 | 0.99994333 | -4.2740742 | 2.0044578 |
| KIAA1328 | 0.17923325 | 4.16766564 | 2.03616888 | 0.04506912 | 0.99994333 | -4.2664277 | 2.00400881 |
| DGUOK | -0.2447825 | 5.17883976 | -2.0358711 | 0.0450772 | 0.99994333 | -4.2779533 | -2.0039333 |
| FAM50A | -0.2147519 | 5.94372255 | -2.0347134 | 0.04519157 | 0.99994333 | -4.2801889 | -2.002867 |
| KRT18P63 | -1.2489923 | -3.0781876 | -2.0344995 | 0.04525147 | 0.99994333 | -4.316995 | -2.0023093 |
| PAPOLA-DT | -0.3447132 | 0.02059232 | -2.0334867 | 0.04528217 | 0.99994333 | -4.2631637 | -2.0020238 |
| GATD3 | -0.2753958 | 2.65711215 | -2.0336919 | 0.04531275 | 0.99994333 | -4.29323 | -2.0017396 |
| EXOSC3 | 0.21080595 | 4.17488369 | 2.03294667 | 0.04532826 | 0.99994333 | -4.2651761 | 2.0015954 |
| RNU6-1191P | -1.3968097 | -6.1276655 | -2.0327234 | 0.04532984 | 0.99994333 | -4.2766303 | -2.0015808 |
| NDUFA8 | -0.2953263 | 4.90513011 | -2.0319666 | 0.04543213 | 0.99994333 | -4.2850462 | -2.0006314 |
| ZNF286A | 0.16395281 | 4.72901477 | 2.03150742 | 0.04553452 | 0.99994333 | -4.2735803 | 1.99968283 |
| MRPL13 | -0.2392694 | 4.30958274 | -2.0322009 | 0.04560003 | 0.99994333 | -4.2852861 | -1.9990769 |
| MRPL34 | -0.3405951 | 4.1641534 | -2.0298678 | 0.04562795 | 0.99994333 | -4.2856564 | -1.9988189 |
| OR7E128P | -1.8261967 | -4.0956407 | -2.0292885 | 0.04567017 | 0.99994333 | -4.2957849 | -1.998429 |
| CHFR-DT | -1.7948134 | -3.6198749 | -2.0298471 | 0.04577321 | 0.99994333 | -4.2928264 | -1.9974787 |
| PROK1 | -0.9032663 | -1.8622789 | -2.0287932 | 0.04577769 | 0.99994333 | -4.2724754 | -1.9974374 |
| NIP7 | -0.2113423 | 3.94590241 | -2.0292495 | 0.04580728 | 0.99994333 | -4.2763071 | -1.9971648 |
| PHOX2B-AS1 | 1.07912309 | -6.8612274 | 2.02748136 | 0.04598523 | 0.99994333 | -4.2813174 | 1.99552886 |
| C19orf53 | -0.2749624 | 5.14803375 | -2.0275244 | 0.04605178 | 0.99994333 | -4.292389 | -1.9949184 |
| ATG4D | -0.1743762 | 4.63978507 | -2.0257285 | 0.04613011 | 0.99994333 | -4.275504 | -1.9942009 |
| ANKRD26 | 0.21546028 | 4.97996376 | 2.0250752 | 0.04615512 | 0.99994333 | -4.2709057 | 1.99397195 |
| ASB14 | 0.37550853 | 0.31790949 | 2.02393671 | 0.04619447 | 0.99994333 | -4.2685338 | 1.99361206 |
| TAF9P3 | 1.00619487 | -2.9345824 | 2.02129343 | 0.04623746 | 0.99994333 | -4.2822506 | 1.99321912 |
| MTND4P20 | 1.18801818 | -6.5579569 | 2.0236544 | 0.04624716 | 0.99994333 | -4.2649536 | 1.99313049 |
| LINC00460 | 1.7042254 | -6.2648264 | 2.02409106 | 0.04627402 | 0.99994333 | -4.2860862 | 1.99288516 |
| IDH1-AS1 | -0.7586236 | -1.7357484 | -2.024646 | 0.04629635 | 0.99994333 | -4.3238939 | -1.9926814 |
| HDAC4-AS1 | -0.6143677 | -0.6064973 | -2.0231092 | 0.04636999 | 0.99994333 | -4.302331 | -1.9920098 |
| NPM1P26 | 1.17442993 | -2.6978532 | 2.02447097 | 0.0464156 | 0.99994333 | -4.2623328 | 1.99159425 |
| ECHS1 | -0.200602 | 6.37860247 | -2.0229331 | 0.04642236 | 0.99994333 | -4.2861543 | -1.9915326 |
| PIMREGP1 | 1.63034457 | -4.5739576 | 2.01938479 | 0.04644653 | 0.99994333 | -4.26852 | 1.99131258 |
| FAM230A | -1.2225772 | -6.2522622 | -2.0216558 | 0.04648884 | 0.99994333 | -4.2656316 | -1.9909277 |
| RPL7P11 | -1.7226754 | -6.141077 | -2.0188831 | 0.0464925 | 0.99994333 | -4.2919329 | -1.9908944 |
| COX4I1 | -0.2385491 | 7.76745146 | -2.0218077 | 0.04654372 | 0.99994333 | -4.2834687 | -1.9904288 |
| CTU2 | -0.1885143 | 4.15891568 | -2.0219624 | 0.0465492 | 0.99994333 | -4.2744515 | -1.990379 |
| RNU1-16P | 1.3724538 | -3.6492049 | 2.01965434 | 0.04657054 | 0.99994333 | -4.2662662 | 1.99018516 |
| ARF4P2 | 1.72485119 | -4.7227867 | 2.02000511 | 0.04660809 | 0.99994333 | -4.2587381 | 1.98984428 |
| MIR6796 | 1.36723224 | -4.998594 | 2.02058328 | 0.04662572 | 0.99994333 | -4.2496361 | 1.98968432 |
| LINC01215 | -1.5704358 | -6.1420407 | -2.0198317 | 0.04671013 | 0.99994333 | -4.2794082 | -1.9889191 |
| CXXC4-AS1 | 0.6614901 | -0.8692659 | 2.018155 | 0.04690982 | 0.99994333 | -4.2553603 | 1.98711347 |
| STX10 | -0.2524222 | 4.95856604 | -2.018341 | 0.04697945 | 0.99994333 | -4.2827494 | -1.9864854 |
| FLOT1 | -0.2065498 | 7.19682653 | -2.0166145 | 0.04701485 | 0.99994333 | -4.2879452 | -1.9861664 |
| NXNL1 | 1.6436732 | -6.1851108 | 2.01416511 | 0.04702754 | 0.99994333 | -4.2677274 | 1.98605211 |
| ADORA2A-AS1 | 0.72974513 | -0.1647313 | 2.01663201 | 0.04703947 | 0.99994333 | -4.2589395 | 1.98594468 |
| TAS2R6P | 1.02261422 | -2.6593185 | 2.02099102 | 0.04706754 | 0.99994333 | -4.2660428 | 1.98569198 |
| RPL21P94 | 1.43259355 | -4.8083201 | 2.01597792 | 0.04710486 | 0.99994333 | -4.2721568 | 1.98535619 |
| DELEC1 | -0.9501336 | -2.1587069 | -2.0172243 | 0.04712865 | 0.99994333 | -4.3152446 | -1.9851423 |
| SLC4A8 | 0.265584 | 7.34073384 | 2.01634836 | 0.04718374 | 0.99994333 | -4.2850678 | 1.98464718 |
| PSMG3 | -0.2660019 | 4.44317912 | -2.0151554 | 0.04718636 | 0.99994333 | -4.2899741 | -1.9846237 |
| UBN2 | 0.33848977 | 5.89193085 | 2.01416483 | 0.04726533 | 0.99994333 | -4.2806867 | 1.98391485 |
| SPATA2L | -0.2890343 | 4.45403988 | -2.0137203 | 0.04733056 | 0.99994333 | -4.2857182 | -1.9833301 |
| ZNF780B | 0.25515235 | 4.98777488 | 2.01180193 | 0.04747836 | 0.99994333 | -4.2806087 | 1.98200783 |
| NAA20 | -0.1899554 | 5.02221055 | -2.0123637 | 0.04748444 | 0.99994333 | -4.2845926 | -1.9819536 |
| FMR1-AS1 | 1.29206141 | -3.4974956 | 2.00982611 | 0.04753007 | 0.99994333 | -4.2713342 | 1.98154602 |
| LNCTSI | -1.5471159 | -6.3292187 | -2.0090793 | 0.04754468 | 0.99994333 | -4.2764978 | -1.9814157 |
| PSMB4 | -0.2090701 | 7.06719344 | -2.0136285 | 0.0475588 | 0.99994333 | -4.2892142 | -1.9812897 |
| CAP1P2 | -1.6804311 | -3.6321316 | -2.0089731 | 0.04758743 | 0.99994333 | -4.302897 | -1.9810343 |
| COPS5P2 | -1.6638949 | -5.488686 | -2.008818 | 0.04782986 | 0.99994333 | -4.2853482 | -1.978877 |
| RPL41P5 | -0.8840347 | -1.9091282 | -2.0083659 | 0.04787477 | 0.99994333 | -4.3109341 | -1.9784783 |
| BCKDK | -0.1596421 | 5.72332675 | -2.0086079 | 0.04793078 | 0.99994333 | -4.2890132 | -1.9779816 |
| ATP5MFP2 | 1.19546919 | -3.6178684 | 2.0079098 | 0.04793511 | 0.99994333 | -4.2621892 | 1.97794326 |
| MRPL58 | -0.2351105 | 3.62510607 | -2.0125226 | 0.04798097 | 0.99994333 | -4.2868741 | -1.9775369 |
| LINC00620 | -0.8247975 | -1.3861411 | -2.0084712 | 0.04807124 | 0.99994333 | -4.3027422 | -1.9767381 |
| RPL7P1 | -0.6048831 | 0.25412813 | -2.0072818 | 0.04809812 | 0.99994333 | -4.306701 | -1.9765005 |
| THADA | 0.20255015 | 4.80805895 | 2.007221 | 0.04811381 | 0.99994333 | -4.2933287 | 1.97636187 |
| MZT2B | -0.3719358 | 4.42644529 | -2.006019 | 0.04813831 | 0.99994333 | -4.2973826 | -1.9761454 |
| NFKBIB | -0.2634451 | 4.50572203 | -2.0073589 | 0.04816885 | 0.99994333 | -4.2958517 | -1.9758758 |
| PRODH | 0.66030317 | 0.23146668 | 2.00587515 | 0.0482207 | 0.99994333 | -4.2715419 | 1.9754183 |
| ALKBH4 | -0.1857015 | 3.8989142 | -2.0051415 | 0.04826964 | 0.99994333 | -4.2854936 | -1.9749868 |
| FIS1 | -0.2454896 | 6.02279587 | -2.0052499 | 0.04829653 | 0.99994333 | -4.2916419 | -1.97475 |
| RNU6-1156P | -1.6985094 | -6.0518412 | -2.0017561 | 0.04833962 | 0.99994333 | -4.2904737 | -1.9743706 |
| PIM3 | -0.2697042 | 5.11011358 | -2.0046778 | 0.04841668 | 0.99994333 | -4.2875512 | -1.9736929 |
| ELOC | -0.2439167 | 5.59510685 | -2.0033706 | 0.04846104 | 0.99994333 | -4.2923637 | -1.9733031 |
| RPL26L1 | -0.2665128 | 4.28969808 | -2.0026618 | 0.04854013 | 0.99994333 | -4.2962414 | -1.972609 |
| ANKRD20A11P | 0.83630724 | -5.5787617 | 2.00296426 | 0.048542 | 0.99994333 | -4.2579854 | 1.97259254 |
| LINC00315 | 1.61688104 | -4.5120043 | 2.00352881 | 0.04861269 | 0.99994333 | -4.3053114 | 1.971973 |
| IFNGR2 | -0.3169456 | 4.95105489 | -2.0012896 | 0.04867451 | 0.99994333 | -4.2916861 | -1.9714317 |
| C12orf71BP | -1.6748384 | -6.0591859 | -2.0002991 | 0.04871404 | 0.99994333 | -4.2729599 | -1.971086 |
| MAGI1-IT1 | -1.6849755 | -5.8040698 | -2.0012544 | 0.04874007 | 0.99994333 | -4.2873259 | -1.9708584 |
| ZNF451 | 0.17304275 | 5.72282969 | 2.00085819 | 0.0488415 | 0.99994333 | -4.2900019 | 1.96997273 |
| FBXL20 | 0.24480182 | 6.02280602 | 1.99807914 | 0.04900952 | 0.99994333 | -4.2890109 | 1.96850886 |
| CRACDL | -0.6037718 | 2.47080239 | -1.9990274 | 0.04903639 | 0.99994333 | -4.2708452 | -1.9682751 |
| HSPBP1 | -0.2542811 | 6.18686775 | -1.9986677 | 0.04905805 | 0.99994333 | -4.2962264 | -1.9680868 |
| ADAT3 | -0.3914381 | 1.94023245 | -1.9968538 | 0.04924763 | 0.99994333 | -4.2849976 | -1.9664415 |
| MTCO3P29 | 1.62985809 | -5.1215438 | 1.9948598 | 0.04927224 | 0.99994333 | -4.2886885 | 1.96622833 |
| RNU1-91P | 1.46662872 | -4.9255682 | 1.9952746 | 0.04930758 | 0.99994333 | -4.2678318 | 1.96592236 |
| RN7SL787P | 1.21910187 | -5.9656269 | 1.99415649 | 0.04948555 | 0.99994333 | -4.2664311 | 1.96438422 |
| CYP51A1P1 | -1.2271585 | -2.8814139 | -1.9938112 | 0.04948604 | 0.99994333 | -4.2821245 | -1.96438 |
| ATP5MC3 | -0.3468284 | 6.5342201 | -1.9935055 | 0.04949305 | 0.99994333 | -4.2946659 | -1.9643195 |
| RPS27 | -0.4951073 | 7.84327869 | -1.9947974 | 0.04961959 | 0.99994333 | -4.2976345 | -1.9632288 |
| IFI27L2 | -0.4254118 | 4.33894134 | -1.993516 | 0.04963981 | 0.99994333 | -4.3168337 | -1.9630548 |
| GPX2 | 0.93017972 | -2.4196147 | 1.99808709 | 0.04964774 | 0.99994333 | -4.3046896 | 1.96298656 |
| HNRNPA1P1 | -1.5066104 | -6.5720852 | -1.9892782 | 0.04975591 | 0.99994333 | -4.2932155 | -1.9620565 |
| FAM136A | -0.1596867 | 5.1353759 | -1.9911748 | 0.04985113 | 0.99994333 | -4.2943742 | -1.9612392 |

**Supplementary Table S5:** Differentially Expressed Genes after Psilocybin at Six Hours

| gene_name | logFC | AveExpr | t | P.Value | adj.P.Val | B | z.std |
| --- | --- | --- | --- | --- | --- | --- | --- |
| ITPKB-IT1 | -3.1740104 | -6.0282751 | -3.9957379 | 0.00014283 | 0.99982423 | -3.3426498 | -3.8032078 |
| ALPG | 2.41832498 | -6.0473513 | 3.68785468 | 0.00040653 | 0.99982423 | -3.3468705 | 3.53581045 |
| LINC01996 | -2.2958043 | -6.581205 | -3.5914699 | 0.00056192 | 0.99982423 | -3.5592003 | -3.4493565 |
| H2AC14 | -2.3279765 | -6.6666084 | -3.5713614 | 0.00060049 | 0.99982423 | -3.5447353 | -3.431391 |
| LRRC52-AS1 | -2.5167094 | -6.2565009 | -3.3975947 | 0.00105047 | 0.99982423 | -3.5995654 | -3.2766495 |
| LINC02612 | 2.60499147 | -5.9471122 | 3.24581137 | 0.00169741 | 0.99982423 | -3.6660153 | 3.1386271 |
| MUC16 | 2.87283078 | -5.8126012 | 3.2377844 | 0.00173172 | 0.99982423 | -3.6664749 | 3.13275892 |
| RPL21P110 | 2.61973497 | -5.5622317 | 3.23400294 | 0.00174933 | 0.99982423 | -3.7413805 | 3.12978748 |
| LINC00523 | 2.87203615 | -5.698662 | 3.20945427 | 0.0018469 | 0.99982423 | -3.6682681 | 3.11380735 |
| LINC01795 | -2.4093451 | -6.0421413 | -3.2002223 | 0.00196364 | 0.99982423 | -3.7827088 | -3.0956766 |
| UHRF2P1 | 2.69074804 | -4.0345766 | 3.18898809 | 0.00207436 | 0.99982423 | -3.7066868 | 3.07937378 |
| CLEC4A | 2.43965559 | -5.8034134 | 3.16333146 | 0.00219762 | 0.99982423 | -3.750841 | 3.06213879 |
| LINC02669 | -2.8909184 | -4.6752452 | -3.1567455 | 0.0022811 | 0.99982423 | -3.8502994 | -3.0509618 |
| FABP5P7 | -1.740396 | -2.8608895 | -3.0642202 | 0.00296352 | 0.99982423 | -4.0328457 | -2.9714967 |
| CSTA | 2.24138997 | -6.0556931 | 3.0548466 | 0.0030342 | 0.99982423 | -3.8081063 | 2.96425197 |
| STON1-GTF2A1L | -2.3827883 | -5.386914 | -2.9520473 | 0.00415331 | 0.99982423 | -3.9559787 | -2.8662768 |
| TTC4 | -0.7860078 | -0.8751225 | -2.952858 | 0.00424298 | 0.99982423 | -3.9634988 | -2.8595082 |
| KLRB1 | -2.3025327 | -5.8906484 | -2.9401111 | 0.00425786 | 0.99982423 | -3.8994521 | -2.8583983 |
| TRIM60P13 | 1.16541496 | -6.5394726 | 2.91914456 | 0.00454184 | 0.99982423 | -3.864121 | 2.83785099 |
| LAMTOR3P2 | 2.37208649 | -4.8796433 | 2.90966912 | 0.00456413 | 0.99982423 | -3.8578305 | 2.83628812 |
| RPL6P9 | 1.86695626 | -3.8419612 | 2.91434256 | 0.00460978 | 0.99982423 | -3.8944442 | 2.83310825 |
| LINC01643 | 2.08311609 | -5.6793158 | 2.90292695 | 0.00478031 | 0.99982423 | -3.8647412 | 2.8214765 |
| LINC02226 | -1.9554825 | -5.7685403 | -2.8858912 | 0.00499854 | 0.99982423 | -3.9097736 | -2.8071281 |
| TMPRSS11D | 2.45780765 | -5.0960055 | 2.86822756 | 0.00526336 | 0.99982423 | -3.8949406 | 2.79045746 |
| MIR4527HG | -2.2533486 | -5.468121 | -2.8532413 | 0.0055301 | 0.99982423 | -3.9891054 | -2.7744153 |
| LINC01877 | -2.6388484 | -5.4657499 | -2.8424601 | 0.00565672 | 0.99982423 | -3.978969 | -2.7670433 |
| MYO5BP2 | 1.25143407 | -5.9758904 | 2.83297242 | 0.00581804 | 0.99982423 | -3.8799173 | 2.75786386 |
| MTUS1-DT | -2.2909681 | -5.2577195 | -2.769646 | 0.00693095 | 0.99982423 | -3.9754712 | -2.700144 |
| TBC1D3D | 1.39677824 | -5.4806828 | 2.75247926 | 0.00731188 | 0.99982423 | -3.9730011 | 2.68229579 |
| LINC00334 | 0.99982153 | -0.6371542 | 2.75175235 | 0.00735093 | 0.99982423 | -3.9789519 | 2.6805135 |
| TMEM125 | 2.60358997 | -4.6844752 | 2.73792384 | 0.00786126 | 0.99982423 | -4.0322524 | 2.65797134 |
| RPS25P9 | -1.9073719 | -5.6573957 | -2.7250141 | 0.00788773 | 0.99982423 | -3.9789752 | -2.6568381 |
| RPL21P125 | 1.09361798 | -1.3129408 | 2.70593718 | 0.00830801 | 0.99982423 | -4.0201779 | 2.63928915 |
| TPSAB1 | 1.63294711 | -5.8795261 | 2.67882284 | 0.00896138 | 0.99982423 | -3.9597482 | 2.61352384 |
| MIA2-AS1 | -0.8562247 | -0.7410001 | -2.6706876 | 0.00914662 | 0.99982423 | -4.0894828 | -2.606525 |
| CEP57L1P1 | 2.08870475 | -6.2912612 | 2.66109202 | 0.00924607 | 0.99982423 | -4.0087002 | 2.60281932 |
| LMAN1L | 2.0989386 | -5.2845784 | 2.6632069 | 0.00934301 | 0.99982423 | -4.0388633 | 2.59924137 |
| ANXA8 | -2.3880586 | -6.0884374 | -2.6368248 | 0.00986801 | 0.99982423 | -4.0305129 | -2.5804205 |
| LINC02424 | -1.9323307 | -5.7761459 | -2.6199548 | 0.01046018 | 0.99982423 | -4.1111868 | -2.5602336 |
| FABP5 | -0.4617256 | 3.79011616 | -2.602261 | 0.01104777 | 0.99982423 | -4.1271468 | -2.5411842 |
| TMEM167B-DT | -0.4932613 | 1.15976083 | -2.5807677 | 0.01171597 | 0.99982423 | -4.1325837 | -2.5205856 |
| LINC01756 | -1.7795236 | -6.5631668 | -2.5748565 | 0.01182228 | 0.99982423 | -4.0923556 | -2.517405 |
| LINC02338 | 1.4423323 | -4.043712 | 2.57416878 | 0.01191216 | 0.99982423 | -4.1290741 | 2.51473596 |
| BICD1P1 | 2.18780143 | -5.8327326 | 2.56583327 | 0.01195788 | 0.99982423 | -4.0553378 | 2.5133848 |
| ANAPC1P1 | 1.70990141 | -4.7374504 | 2.57230217 | 0.01200083 | 0.99982423 | -4.0321415 | 2.51212005 |
| MIR93 | -1.6258825 | -5.5861083 | -2.5602254 | 0.01231551 | 0.99982423 | -4.088976 | -2.5029726 |
| CCL4L2 | 1.69089214 | -6.6877502 | 2.53474048 | 0.01316236 | 0.99982423 | -4.065674 | 2.47934577 |
| MEF2C-AS1 | 0.78414896 | -1.8093715 | 2.53021851 | 0.0133861 | 0.99982423 | -4.063061 | 2.4733286 |
| LINC01583 | 1.83054103 | -5.8148263 | 2.51285798 | 0.01376675 | 0.99982423 | -4.0586496 | 2.46329273 |
| LINC01614 | -2.6504488 | -5.4063968 | -2.514677 | 0.0138669 | 0.99982423 | -4.1223138 | -2.4606928 |
| FAM183BP | 2.24912011 | -5.543569 | 2.51114353 | 0.01398119 | 0.99982423 | -4.0879809 | 2.45774641 |
| RPL21P46 | -2.3443035 | -5.1044252 | -2.5091554 | 0.01414375 | 0.99982423 | -4.1413249 | -2.4535917 |
| HSPB1P1 | 2.32427811 | -5.2206211 | 2.4776552 | 0.01511257 | 0.99982423 | -4.1258969 | 2.42967009 |
| ATP5F1CP1 | -2.1737482 | -4.3488205 | -2.4793356 | 0.01519003 | 0.99982423 | -4.1485708 | -2.4278164 |
| LINC01821 | 1.83039338 | -4.9110184 | 2.47190332 | 0.01553987 | 0.99982423 | -4.137528 | 2.41954661 |
| KRT18P50 | -1.9350028 | -6.2520056 | -2.4693712 | 0.01563721 | 0.99982423 | -4.1306273 | -2.4172748 |
| CDC14C | -2.1599218 | -5.9543078 | -2.449678 | 0.01637988 | 0.99982423 | -4.1457688 | -2.4003393 |
| IL17REL | 1.85374933 | -3.7759935 | 2.44952579 | 0.01656191 | 0.99982423 | -4.1028744 | 2.39629163 |
| SLC12A3 | -2.0556726 | -4.9720337 | -2.4292064 | 0.01739581 | 0.99982423 | -4.1445598 | -2.3782334 |
| CLEC20A | 1.71849606 | -6.0599058 | 2.41841408 | 0.0177814 | 0.99982423 | -4.092615 | 2.37013897 |
| ATP6V1B1 | 1.64214514 | -4.8549926 | 2.41887833 | 0.01783761 | 0.99982423 | -4.1146248 | 2.36897197 |
| ASAP1-IT2 | 1.87355877 | -3.8378135 | 2.42108984 | 0.01793894 | 0.99982423 | -4.143013 | 2.36687602 |
| CCT6P1 | 1.50719489 | -3.2714811 | 2.41813504 | 0.01794591 | 0.99982423 | -4.1476763 | 2.36673227 |
| LINC00934 | -1.9658932 | -6.0213916 | -2.4108256 | 0.01820675 | 0.99982423 | -4.1472024 | -2.3613863 |
| AMHR2 | 1.96823257 | -5.9317742 | 2.40450882 | 0.01844101 | 0.99982423 | -4.1300117 | 2.35664217 |
| LINC01722 | -1.8230765 | -5.635379 | -2.4024581 | 0.01857117 | 0.99982423 | -4.1523965 | -2.3540288 |
| SPATA45 | 1.33103571 | -3.3265757 | 2.39848852 | 0.01873646 | 0.99982423 | -4.125945 | 2.35073316 |
| TAS2R30 | 2.13875478 | -5.3823198 | 2.39597896 | 0.01884418 | 0.99982423 | -4.1184284 | 2.34859917 |
| DNPEP-AS1 | -1.6978833 | -3.2947658 | -2.3946206 | 0.01885826 | 0.99982423 | -4.2122944 | -2.3483209 |
| TACR3-AS1 | 1.48778738 | -5.4594123 | 2.3946754 | 0.01896637 | 0.99982423 | -4.1791873 | 2.34619127 |
| SNORD115-22 | -1.7172403 | -5.2692643 | -2.3897133 | 0.01930007 | 0.99982423 | -4.1889983 | -2.3396839 |
| HMGN2P46 | -0.7383366 | -1.4411265 | -2.3845242 | 0.01955122 | 0.99982423 | -4.2091634 | -2.3348508 |
| MTND1P21 | 1.65268444 | -6.3539018 | 2.37298248 | 0.01998882 | 0.99982423 | -4.1436788 | 2.3265577 |
| MRPS36P4 | -1.9238866 | -6.1674757 | -2.3756807 | 0.02007102 | 0.99982423 | -4.186162 | -2.3250176 |
| RNU6-644P | 1.93943549 | -4.962226 | 2.36649023 | 0.02032669 | 0.99982423 | -4.1420694 | 2.32026241 |
| RAB37 | 0.97718919 | -0.8557096 | 2.36529976 | 0.02041598 | 0.99982423 | -4.1759469 | 2.31861392 |
| BMS1P20 | 0.52004454 | -0.7926686 | 2.36572277 | 0.02044473 | 0.99982423 | -4.1486014 | 2.3180844 |
| RPL23AP95 | -2.1488075 | -4.8928073 | -2.3594975 | 0.02049635 | 0.99982423 | -4.2139013 | -2.3171355 |
| MIR4441 | 1.87767873 | -5.619978 | 2.35473281 | 0.02072308 | 0.99982423 | -4.1822138 | 2.31299191 |
| RPL27P5 | 1.85417332 | -4.7872767 | 2.35144189 | 0.02113959 | 0.99982423 | -4.1252243 | 2.30548214 |
| MIR3145 | -1.9384618 | -5.8283802 | -2.3500034 | 0.02127685 | 0.99982423 | -4.196323 | -2.3030354 |
| LINC00710 | -2.2667276 | -5.4761226 | -2.3449284 | 0.02144529 | 0.99982423 | -4.1855792 | -2.3000517 |
| LINC01053 | 1.86557618 | -6.2511621 | 2.3400612 | 0.02152789 | 0.99982423 | -4.1749837 | 2.29859606 |
| RPL24P2 | -2.139594 | -4.4770787 | -2.3466341 | 0.02153613 | 0.99982423 | -4.223424 | -2.2984511 |
| MIR4488 | -2.159891 | -5.0609882 | -2.3452519 | 0.02155867 | 0.99982423 | -4.2441291 | -2.2980547 |
| MC3R | -1.7517233 | -6.3841332 | -2.3414795 | 0.0216003 | 0.99982423 | -4.1954974 | -2.2973239 |
| NPC1L1 | 1.88422077 | -3.5400652 | 2.34134313 | 0.02166228 | 0.99982423 | -4.221629 | 2.29623788 |
| RNU6-8 | -2.268748 | -4.6830367 | -2.3393388 | 0.02167211 | 0.99982423 | -4.1718561 | -2.296066 |
| LINC01412 | 1.88675346 | -5.5301723 | 2.33951989 | 0.02172412 | 0.99982423 | -4.1910163 | 2.29515719 |
| ECMXP | 1.77292143 | -5.8187106 | 2.34173944 | 0.0217351 | 0.99982423 | -4.1643898 | 2.29496557 |
| ATP5MFP4 | 1.89783815 | -5.743633 | 2.33451835 | 0.02180697 | 0.99982423 | -4.1343985 | 2.29371325 |
| MCIDAS | -1.3136223 | -5.9945075 | -2.3362794 | 0.02193404 | 0.99982423 | -4.1440913 | -2.2915081 |
| RBPMS-AS1 | -2.2892298 | -4.2797034 | -2.3293494 | 0.02234524 | 0.99982423 | -4.2152387 | -2.2844476 |
| PSPHP1 | -0.5659941 | -4.2639955 | -2.3281397 | 0.02244618 | 0.99982423 | -4.1735059 | -2.2827316 |
| EIF2AP4 | 2.29083081 | -5.6044092 | 2.32283859 | 0.02245592 | 0.99982423 | -4.1702674 | 2.28256639 |
| RAB44 | 2.1059878 | -3.8711614 | 2.32306853 | 0.02263274 | 0.99982423 | -4.2003172 | 2.27957773 |
| LINC01637 | -1.6980351 | -3.0622142 | -2.3212829 | 0.0228266 | 0.99982423 | -4.1825958 | -2.2763244 |
| SH3BGR | -0.7743476 | -1.6644513 | -2.3147987 | 0.02324983 | 0.99982423 | -4.2074205 | -2.2693042 |
| GK4P | -1.5336637 | -3.9839322 | -2.3091163 | 0.02349494 | 0.99982423 | -4.244796 | -2.2652891 |
| PLA2G4D | 1.93745022 | -4.9826655 | 2.30709257 | 0.0236336 | 0.99982423 | -4.1962492 | 2.26303394 |
| GTF2IP23 | 0.68398496 | -0.5451983 | 2.30171386 | 0.02392023 | 0.99982423 | -4.184538 | 2.25840806 |
| RN7SL812P | 1.63082425 | -4.8888578 | 2.29936315 | 0.0241841 | 0.99982423 | -4.1907533 | 2.25419181 |
| SETP14 | -0.424474 | 0.21763894 | -2.2957474 | 0.02427081 | 0.99982423 | -4.2093132 | -2.2528151 |
| TXNP1 | -1.7694836 | -5.9878081 | -2.289583 | 0.02461126 | 0.99982423 | -4.2090374 | -2.2474503 |
| NDUFV1-DT | 2.05529326 | -4.4489954 | 2.28755493 | 0.02477171 | 0.99982423 | -4.2161957 | 2.24494424 |
| ASTILCS | -2.0566996 | -4.51057 | -2.2871955 | 0.02484371 | 0.99982423 | -4.2062678 | -2.2438243 |
| RPL18AP3 | -0.6470743 | 0.88571209 | -2.284422 | 0.02495347 | 0.99982423 | -4.2598462 | -2.2421223 |
| MIR3127 | -1.9884867 | -5.7346901 | -2.2803523 | 0.02499831 | 0.99982423 | -4.2114193 | -2.2414289 |
| DYNLL1P4 | 1.91255775 | -4.9914137 | 2.27587921 | 0.02524524 | 0.99982423 | -4.1886391 | 2.23762926 |
| RNU6-484P | 1.72307629 | -5.2782071 | 2.27825052 | 0.02527461 | 0.99982423 | -4.1667784 | 2.23717951 |
| TATDN1P1 | -2.1942192 | -4.9527811 | -2.2746612 | 0.02531621 | 0.99982423 | -4.2531149 | -2.2365432 |
| NAV2-IT1 | -1.8955429 | -5.7782263 | -2.277569 | 0.0253739 | 0.99982423 | -4.2280034 | -2.2356622 |
| CAV3 | -2.2605197 | -5.1908733 | -2.2698607 | 0.02561808 | 0.99982423 | -4.225721 | -2.2319528 |
| PABPC1P11 | 2.15636081 | -5.9031815 | 2.26986493 | 0.02584412 | 0.99982423 | -4.2021274 | 2.22854615 |
| SCARNA18 | 1.8291018 | -5.4169033 | 2.26824585 | 0.02584576 | 0.99982423 | -4.213904 | 2.22852143 |
| RN7SL265P | 1.92435926 | -5.7769712 | 2.2676475 | 0.02605832 | 0.99982423 | -4.1897097 | 2.22534158 |
| HAFML | 1.59858611 | -5.1274738 | 2.26388073 | 0.02624509 | 0.99982423 | -4.1845507 | 2.22256586 |
| INGX | 2.0682124 | -5.552465 | 2.26191177 | 0.02630629 | 0.99982423 | -4.1880422 | 2.22165996 |
| LINC02335 | -1.5071889 | -6.484512 | -2.2623337 | 0.02630771 | 0.99982423 | -4.2242036 | -2.221639 |
| PRRT3-AS1 | 1.21466596 | -3.0171006 | 2.2599097 | 0.02639395 | 0.99982423 | -4.1774456 | 2.22036575 |
| DSG2-AS1 | -1.7745263 | -5.2491178 | -2.2576775 | 0.02639845 | 0.99982423 | -4.1859062 | -2.2202994 |
| RPL21P87 | 1.69978924 | -5.8796387 | 2.2608997 | 0.02648705 | 0.99982423 | -4.1822043 | 2.21899515 |
| KBTBD12 | -1.756934 | -4.2376624 | -2.2576313 | 0.02663085 | 0.99982423 | -4.2318645 | -2.2168865 |
| GTF3C6P2 | 1.91670231 | -5.2695892 | 2.25390842 | 0.02664406 | 0.99982423 | -4.1908797 | 2.21669321 |
| TBCEL-TECTA | 0.68110392 | -1.7106812 | 2.25369862 | 0.02683799 | 0.99982423 | -4.1845184 | 2.21386614 |
| TMEM89 | 1.14496954 | -2.3179683 | 2.26342621 | 0.02686775 | 0.99982423 | -4.1960583 | 2.21343383 |
| PTCHD1-AS | -1.6305819 | -3.942903 | -2.2550586 | 0.0269447 | 0.99982423 | -4.2451416 | -2.212318 |
| TMEM183AP3 | 1.92676675 | -4.5858943 | 2.25364322 | 0.02704377 | 0.99982423 | -4.2350014 | 2.21088546 |
| H2BC17 | -2.0800828 | -5.2314824 | -2.2419189 | 0.02743876 | 0.99982423 | -4.2213444 | -2.2052186 |
| BUB1B-PAK6 | 1.49897181 | -6.1703845 | 2.2446732 | 0.02747069 | 0.99982423 | -4.1875241 | 2.20476356 |
| TBXT | -1.7304013 | -5.6787866 | -2.2451049 | 0.02748186 | 0.99982423 | -4.2145599 | -2.2046045 |
| SH3PXD2A-AS1 | 1.93510091 | -5.5774177 | 2.24022764 | 0.02781548 | 0.99982423 | -4.2092789 | 2.19987907 |
| ARLNC1 | 1.76623263 | -5.1191052 | 2.24190832 | 0.02782094 | 0.99982423 | -4.1911036 | 2.19980205 |
| RPL22L1 | -0.4140136 | 2.39455067 | -2.2400104 | 0.02789565 | 0.99982423 | -4.2635301 | -2.1987507 |
| RPL15P21 | -1.9410156 | -5.9339042 | -2.2356334 | 0.02806252 | 0.99982423 | -4.2387775 | -2.1964113 |
| CBLC | 1.54973248 | -6.3697584 | 2.23154727 | 0.02835996 | 0.99982423 | -4.1978152 | 2.19227078 |
| LINC00524 | 1.09789686 | -6.7100787 | 2.23172582 | 0.02836569 | 0.99982423 | -4.2065675 | 2.19219133 |
| ARMH2 | 1.63937744 | -5.1223854 | 2.2313498 | 0.02837172 | 0.99982423 | -4.1823765 | 2.19210791 |
| IL1A | 1.76922324 | -3.3749798 | 2.23104979 | 0.0284286 | 0.99982423 | -4.2393053 | 2.19132066 |
| EEF1A1P7 | 1.66276226 | -3.3822176 | 2.22645998 | 0.02850638 | 0.99982423 | -4.2339064 | 2.1902463 |
| LINC01605 | 1.94038571 | -4.8244176 | 2.22728273 | 0.0286345 | 0.99982423 | -4.2004485 | 2.1884821 |
| PPP1R12A-AS2 | -1.4651609 | -4.8325945 | -2.2230883 | 0.02907475 | 0.99982423 | -4.2662966 | -2.1824715 |
| LINC01284 | 0.82748419 | -1.5078183 | 2.22134017 | 0.02929882 | 0.99982423 | -4.2262235 | 2.17944235 |
| GPR142 | -1.8217707 | -6.1825079 | -2.2181684 | 0.02931521 | 0.99982423 | -4.2547636 | -2.1792215 |
| LINC01907 | -1.6404215 | -3.6348504 | -2.219156 | 0.02942392 | 0.99982423 | -4.2499545 | -2.1777598 |
| SCGB3A1 | -2.272281 | -4.7979113 | -2.2143877 | 0.0295597 | 0.99982423 | -4.2461821 | -2.1759406 |
| LINC01116 | -1.8666673 | -6.1043955 | -2.2142795 | 0.0296168 | 0.99982423 | -4.2540473 | -2.1751777 |
| PIRAT1 | -1.690777 | -6.2305147 | -2.2070179 | 0.03005585 | 0.99982423 | -4.2339055 | -2.1693535 |
| SCARA5 | 1.45226904 | -3.8048439 | 2.20588104 | 0.03021671 | 0.99982423 | -4.1720261 | 2.16723807 |
| MPC1L | 1.81993942 | -6.0844498 | 2.20455572 | 0.03028154 | 0.99982423 | -4.2339875 | 2.16638815 |
| RNU6-1191P | -1.6132175 | -6.1276655 | -2.2047487 | 0.03028303 | 0.99982423 | -4.2506047 | -2.1663687 |
| TAS2R4 | 0.62126052 | 0.36952885 | 2.20494931 | 0.03029248 | 0.99982423 | -4.2216059 | 2.16624485 |
| CYTL1 | 1.82068222 | -1.9822827 | 2.20319797 | 0.0304065 | 0.99982423 | -4.2633837 | 2.16475439 |
| LINC01215 | -1.7962766 | -6.1420407 | -2.1990781 | 0.03072936 | 0.99982423 | -4.2424882 | -2.1605598 |
| HIGD1B | 1.49977707 | -2.8868477 | 2.19991902 | 0.03091734 | 0.99982423 | -4.2794236 | 2.15813492 |
| MIR4470 | 1.60776203 | -6.2715322 | 2.19557265 | 0.03093038 | 0.99982423 | -4.2108261 | 2.15796728 |
| RNU6-126P | -1.8901437 | -3.8597201 | -2.1965112 | 0.03099517 | 0.99982423 | -4.2699638 | -2.1571347 |
| RN7SL68P | 1.75164729 | -5.1034376 | 2.19342596 | 0.03103964 | 0.99982423 | -4.248472 | 2.1565641 |
| PRR34 | -0.9977101 | -1.1504495 | -2.1947693 | 0.031236 | 0.99982423 | -4.2887103 | -2.1540532 |
| MAL2-AS1 | 1.96706445 | -5.4481785 | 2.19153118 | 0.03125611 | 0.99982423 | -4.2475972 | 2.15379677 |
| MRPS36P1 | -1.9139681 | -5.7324042 | -2.1825995 | 0.03168396 | 0.99982423 | -4.2338366 | -2.1483752 |
| GOLGA6D | -1.861665 | -5.563182 | -2.1789002 | 0.03201087 | 0.99982423 | -4.267613 | -2.1442749 |
| EPPIN | -1.6759674 | -6.1001888 | -2.1736639 | 0.03271123 | 0.99982423 | -4.2527474 | -2.1356099 |
| PKP4-AS1 | 1.13105678 | -1.5952759 | 2.17188499 | 0.03272839 | 0.99982423 | -4.2426075 | 2.13539951 |
| RCAN3AS | 1.75356184 | -5.039757 | 2.17152126 | 0.03273031 | 0.99982423 | -4.2390054 | 2.13537603 |
| LINC02549 | 1.54233094 | -4.0273704 | 2.17211595 | 0.03273378 | 0.99982423 | -4.2506988 | 2.13533352 |
| RNU6ATAC16P | 1.2887442 | -3.963212 | 2.16999049 | 0.03293497 | 0.99982423 | -4.2602483 | 2.13287508 |
| GUSBP7 | -1.8233275 | -5.2932258 | -2.1668705 | 0.03324191 | 0.99982423 | -4.2754134 | -2.1291492 |
| LINC01800 | 1.7801669 | -4.048997 | 2.16690868 | 0.03327967 | 0.99982423 | -4.2690333 | 2.12869293 |
| ZNF114 | -0.517455 | 1.76220735 | -2.1634951 | 0.03346065 | 0.99982423 | -4.2778051 | -2.1265121 |
| RPL17P9 | -2.0161099 | -4.2528835 | -2.1626913 | 0.03364642 | 0.99982423 | -4.3027412 | -2.1242838 |
| LINC01254 | -1.9371718 | -4.6792942 | -2.1585489 | 0.03378548 | 0.99982423 | -4.2923726 | -2.1226227 |
| ZSCAN4 | -1.8996972 | -5.9679024 | -2.1561624 | 0.03401018 | 0.99982423 | -4.2661543 | -2.119951 |
| RARRES2P2 | 1.70971378 | -4.9818794 | 2.15119858 | 0.03443077 | 0.99982423 | -4.2334184 | 2.11499031 |
| MIR135B | 1.77892613 | -3.7515075 | 2.15022109 | 0.03451639 | 0.99982423 | -4.2661229 | 2.11398683 |
| DDX3ILA1 | -1.8028349 | -5.5104426 | -2.1433051 | 0.03482764 | 0.99982423 | -4.2609592 | -2.1103567 |
| INSRR | 1.13341401 | -1.6406999 | 2.14444051 | 0.0349465 | 0.99982423 | -4.2234309 | 2.10897774 |
| FNDC8 | -1.131788 | -2.3409946 | -2.1418019 | 0.03511612 | 0.99982423 | -4.2897032 | -2.1070168 |
| RCCD1-AS1 | 0.89035691 | -0.8997567 | 2.13626406 | 0.0357212 | 0.99982423 | -4.2498106 | 2.10008687 |
| GSTCD-AS1 | 0.9434615 | -2.6274429 | 2.13426056 | 0.03580828 | 0.99982423 | -4.2419382 | 2.09909781 |
| IL18RAP | -1.6236537 | -5.943436 | -2.1328735 | 0.03591527 | 0.99982423 | -4.2345703 | -2.0978854 |
| VN1R107P | 0.69838786 | -1.4334375 | 2.13289527 | 0.03597002 | 0.99982423 | -4.2635093 | 2.09726617 |
| RN7SKP97 | 1.81592115 | -4.7497272 | 2.13051423 | 0.03609937 | 0.99982423 | -4.239398 | 2.09580639 |
| RN7SL827P | -1.8261399 | -4.9147652 | -2.1266955 | 0.03622082 | 0.99982423 | -4.2915611 | -2.0944398 |
| TARS3 | 0.18681013 | 4.80555974 | 2.13110246 | 0.03632 | 0.99982423 | -4.2629428 | 2.09332667 |
| LINC02341 | -1.8375732 | -5.5117201 | -2.125525 | 0.03648831 | 0.99982423 | -4.2419403 | -2.0914437 |
| NDUFB4P12 | -1.9373576 | -5.9794 | -2.1203653 | 0.03674256 | 0.99982423 | -4.2806823 | -2.0886132 |
| HNRNPA1P36 | -1.7350327 | -4.3832305 | -2.1220355 | 0.03686916 | 0.99982423 | -4.2970588 | -2.08721 |
| MAPK6P5 | -1.9883563 | -5.2353979 | -2.1162683 | 0.03709886 | 0.99982423 | -4.2665536 | -2.0846745 |
| NDUFB4P6 | 1.61950501 | -6.1140662 | 2.11826481 | 0.03719498 | 0.99982423 | -4.2604317 | 2.0836174 |
| KLHL30-AS1 | -1.7976874 | -6.0669461 | -2.1143516 | 0.03770998 | 0.99982423 | -4.2864785 | -2.0779932 |
| LENEP | -1.9353685 | -5.1642034 | -2.1096548 | 0.03773077 | 0.99982423 | -4.2954935 | -2.0777676 |
| ARMCX7P | -1.723667 | -6.1782519 | -2.1107729 | 0.03775648 | 0.99982423 | -4.2811428 | -2.0774886 |
| RN7SL481P | 1.07822845 | -1.4619489 | 2.1096586 | 0.03793562 | 0.99982423 | -4.3023759 | 2.07554964 |
| KRT18P40 | 1.75469133 | -5.5036918 | 2.10807898 | 0.03825261 | 0.99982423 | -4.2866351 | 2.07213766 |
| FTLP6 | -1.8854121 | -6.0979418 | -2.0987453 | 0.03867136 | 0.99982423 | -4.284922 | -2.0676669 |
| ARPC4-TTLL3 | 1.18175122 | -5.8195289 | 2.10090064 | 0.03872824 | 0.99982423 | -4.2664688 | 2.06706272 |
| MIR4284 | -1.4983848 | -5.7383215 | -2.0950169 | 0.03923395 | 0.99982423 | -4.2883771 | -2.0617248 |
| CHRNA2 | 1.63632284 | -3.6229584 | 2.09376126 | 0.03939234 | 0.99982423 | -4.2604676 | 2.06006489 |
| RPL9P18 | -1.9371448 | -4.4623453 | -2.0911659 | 0.03961242 | 0.99982423 | -4.2775762 | -2.0577679 |
| RNA5SP108 | 1.7697898 | -4.7692802 | 2.08464087 | 0.03995141 | 0.99982423 | -4.2842944 | 2.05425098 |
| MIR649 | -1.6607255 | -5.7890432 | -2.0864468 | 0.04003334 | 0.99982423 | -4.2905101 | -2.0534048 |
| FCGR1A | 1.21913715 | -2.2840833 | 2.08197135 | 0.04041967 | 0.99982423 | -4.2918518 | 2.04943423 |
| ACSBG2 | 0.66950531 | -0.6903209 | 2.07528588 | 0.0411248 | 0.99982423 | -4.2720343 | 2.04226961 |
| SMARCAL1-AS1 | 1.59685973 | -5.2295671 | 2.07461068 | 0.04119736 | 0.99982423 | -4.2904628 | 2.04153818 |
| CEP83-DT | 0.38702877 | 0.17036751 | 2.0726466 | 0.04132419 | 0.99982423 | -4.2854978 | 2.04026252 |
| LINC01237 | 0.77536199 | -0.2384749 | 2.07493755 | 0.04137777 | 0.99982423 | -4.2833464 | 2.03972452 |
| HSP90AB2P | -1.3332708 | -2.6074593 | -2.0708365 | 0.04143674 | 0.99982423 | -4.2880915 | -2.0391332 |
| ACTN3 | 0.90140794 | -1.7905564 | 2.07524957 | 0.04150554 | 0.99982423 | -4.2747283 | 2.03844412 |
| KRT18P31 | -1.8452466 | -5.6688339 | -2.0714675 | 0.04150668 | 0.99982423 | -4.3071492 | -2.0384327 |
| CRYBA1 | 1.76839579 | -5.5846429 | 2.06615744 | 0.04170512 | 0.99982423 | -4.284789 | 2.03645071 |
| VIL1 | -1.6944919 | -4.8333898 | -2.0652311 | 0.04199102 | 0.99982423 | -4.2921925 | -2.0336091 |
| CD93 | 1.51381988 | -4.3669027 | 2.0658154 | 0.04202436 | 0.99982423 | -4.2421884 | 2.03327887 |
| C1orf202 | -1.7554839 | -3.5539706 | -2.0648785 | 0.04229334 | 0.99982423 | -4.2899948 | -2.0306221 |
| LINC03026 | 0.62560649 | -0.150537 | 2.06077288 | 0.04256667 | 0.99982423 | -4.2940061 | 2.02793712 |
| ASXL3-DT | 1.54521955 | -5.1654218 | 2.06085244 | 0.0425753 | 0.99982423 | -4.2945795 | 2.0278525 |
| REM1 | 1.65191179 | -6.1312676 | 2.05855302 | 0.04286224 | 0.99982423 | -4.2942714 | 2.0250499 |
| HCAR2 | 1.55859339 | -5.6244026 | 2.04312118 | 0.04432596 | 0.99982423 | -4.2937173 | 2.01099526 |
| NQO2-AS1 | -1.2906821 | -3.1310893 | -2.0422468 | 0.04439563 | 0.99982423 | -4.3138446 | -2.0103361 |
| TM4SF18 | -1.8289333 | -4.768799 | -2.0424509 | 0.04441056 | 0.99982423 | -4.3572496 | -2.010195 |
| ALG1L2 | -0.6508514 | -1.6943889 | -2.041507 | 0.04451612 | 0.99982423 | -4.2953331 | -2.0091982 |
| MTCO1P42 | 1.73318394 | -4.0293318 | 2.03836762 | 0.04484025 | 0.99982423 | -4.2907313 | 2.00614993 |
| FRG2C | 1.66908059 | -5.5095638 | 2.03620532 | 0.04497979 | 0.99982423 | -4.2898005 | 2.00484338 |
| CPB2 | 1.58328183 | -3.6340852 | 2.03381917 | 0.04512989 | 0.99982423 | -4.2922935 | 2.00344176 |
| GK-IT1 | -1.7186807 | -6.1538754 | -2.0306805 | 0.04545271 | 0.99982423 | -4.2987382 | -2.0004406 |
| PPIAP41 | 1.78535889 | -4.6365828 | 2.03128274 | 0.04562027 | 0.99982423 | -4.3137042 | 1.99888983 |
| RPS5P2 | -1.7240219 | -6.2123105 | -2.0271699 | 0.04562201 | 0.99982423 | -4.3151752 | -1.9988738 |
| MIR9-1 | 1.4792001 | -4.0766808 | 2.0241007 | 0.04645258 | 0.99982423 | -4.3041434 | 1.99125759 |
| FLJ38576 | -0.542659 | -0.0564253 | -2.0223945 | 0.04652004 | 0.99982423 | -4.3146172 | -1.990644 |
| LMX1A-AS2 | 1.23662731 | -6.4632746 | 2.02061145 | 0.04670448 | 0.99982423 | -4.3129518 | 1.98897033 |
| CD101-AS1 | 1.20417476 | -2.8090052 | 2.01661191 | 0.04673391 | 0.99982423 | -4.2976796 | 1.98870373 |
| ZNF280B | 0.24898826 | 3.33906524 | 2.01627524 | 0.04713417 | 0.99982423 | -4.3098412 | 1.98509256 |
| LBX2-AS1 | -1.0103796 | -2.2237545 | -2.0153757 | 0.0473438 | 0.99982423 | -4.3189379 | -1.9832116 |
| BCL10-AS1 | 0.65863176 | -1.3756238 | 2.01360721 | 0.04742752 | 0.99982423 | -4.2959205 | 1.98246229 |
| CT62 | 1.5182651 | -3.284392 | 2.00502607 | 0.04826491 | 0.99982423 | -4.307368 | 1.97502849 |
| ADGRF5 | 1.82679438 | -4.7331763 | 2.00451554 | 0.04836893 | 0.99982423 | -4.2862763 | 1.97411271 |
| UBE2V1P14 | 1.40540163 | -5.6832764 | 2.00396873 | 0.04839552 | 0.99982423 | -4.3052024 | 1.97387888 |
| RPS2P55 | -0.7084923 | -0.1725244 | -2.0046509 | 0.04843468 | 0.99982423 | -4.3659202 | -1.9735346 |
| FAM153A | 0.95740855 | -1.866127 | 2.00301653 | 0.04861354 | 0.99982423 | -4.3259245 | 1.97196554 |
| PARP11-AS1 | 0.41215521 | -0.3451171 | 2.00032619 | 0.04883547 | 0.99982423 | -4.3242038 | 1.9700253 |
| CHRND | -1.9960528 | -3.8514668 | -2.0020134 | 0.04889898 | 0.99982423 | -4.3540015 | -1.9694714 |
| LINC01581 | -1.862053 | -5.8650554 | -1.9945204 | 0.04940898 | 0.99982423 | -4.3183662 | -1.9650454 |
| CCT5P2 | 1.55298048 | -6.3015549 | 1.99444978 | 0.04940969 | 0.99982423 | -4.3154362 | 1.96503933 |
| PGBD4 | 0.23782672 | 2.89324895 | 1.99365993 | 0.04961102 | 0.99982423 | -4.328399 | 1.96330262 |
| KTN1-AS1 | -0.3130276 | 2.05394725 | -1.9936144 | 0.04962006 | 0.99982423 | -4.3348224 | -1.9632248 |
| LINC02019 | -0.572049 | -0.1990418 | -1.9935929 | 0.04970285 | 0.99982423 | -4.3173554 | -1.9625125 |

**Supplementary Table S6:** Differentially Expressed Genes after LSD at Six Hours

| **Gene Name** | **logFC** | **AveExpr** | **t** | **P.Value** | **adj.P.Val** | **B** | **z.std** |
| --- | --- | --- | --- | --- | --- | --- | --- |
| LRRC52-AS1 | -2.6814284 | -6.2565009 | -3.5917718 | 0.00055812 | 0.99989949 | -3.4981601 | -3.4511881 |
| MIR4441 | 2.63065675 | -5.619978 | 3.4083301 | 0.00098163 | 0.99989949 | -3.6209157 | 3.29574071 |
| LINC01776 | 2.36487942 | -5.8918059 | 3.2801207 | 0.00152613 | 0.99989949 | -3.6957645 | 3.16966863 |
| RPS25P9 | -2.3300233 | -5.6573957 | -3.2401231 | 0.00173933 | 0.99989949 | -3.7433843 | -3.1314704 |
| LMAN1L | 2.5077349 | -5.2845784 | 3.22515023 | 0.0018197 | 0.99989949 | -3.7405255 | 3.11818275 |
| VN1R67P | 2.45211459 | -5.982166 | 3.22005573 | 0.00185253 | 0.99989949 | -3.6915138 | 3.11290921 |
| KRT18P40 | 2.54245832 | -5.5036918 | 3.16192429 | 0.00223635 | 0.99989949 | -3.7725381 | 3.05690577 |
| SLC25A5P3 | -2.7659887 | -5.5475783 | -3.1183638 | 0.00252122 | 0.99989949 | -3.8891885 | -3.0207833 |
| LINC01583 | 2.17743843 | -5.8148263 | 3.03352147 | 0.00316331 | 0.99989949 | -3.7631068 | 2.95140591 |
| NODAL | 1.05739383 | -0.436475 | 2.94881204 | 0.00417618 | 0.99989949 | -3.8690854 | 2.86453805 |
| ZNF541 | -2.568467 | -4.2346702 | -2.9476796 | 0.00417912 | 0.99989949 | -4.0124669 | -2.8643151 |
| PSPN | 0.2562786 | 2.85588604 | 2.94902493 | 0.00423219 | 0.99989949 | -3.8759421 | 2.86031632 |
| H2AC14 | -1.8740462 | -6.6666084 | -2.8938095 | 0.00488615 | 0.99989949 | -3.9042994 | -2.8144454 |
| TOMM20L-DT | 1.48415338 | -3.2423558 | 2.85396106 | 0.00544448 | 0.99989949 | -3.9272619 | 2.77948695 |
| LARP1P1 | 2.24433194 | -5.9628921 | 2.85233511 | 0.00548464 | 0.99989949 | -3.9219986 | 2.77709951 |
| CYP51A1P1 | -1.9682147 | -2.8814139 | -2.8393424 | 0.00569533 | 0.99989949 | -4.0082208 | -2.7648253 |
| RN7SL812P | 1.95877961 | -4.8888578 | 2.82524359 | 0.00601156 | 0.99989949 | -3.9239751 | 2.74714999 |
| AQP7 | 2.24612832 | -5.7213169 | 2.80299385 | 0.00630868 | 0.99989949 | -3.9232405 | 2.73129075 |
| CCDC88C-DT | -2.3157157 | -5.1222205 | -2.7911879 | 0.00656608 | 0.99989949 | -4.0351912 | -2.7180859 |
| LY6L | -1.9442843 | -6.0169035 | -2.7580257 | 0.00718101 | 0.99989949 | -4.0122048 | -2.6883315 |
| LINC00664 | 0.98251673 | -0.7819856 | 2.73200335 | 0.00775121 | 0.99989949 | -3.9888508 | 2.66271925 |
| RN7SL15P | 2.1994686 | -5.6887231 | 2.71827855 | 0.00788028 | 0.99989949 | -3.9433067 | 2.65715663 |
| P2RX1 | 2.15163827 | -5.8087512 | 2.71367643 | 0.00816808 | 0.99989949 | -3.9986483 | 2.64504173 |
| GPR148 | 2.52524789 | -5.7442837 | 2.69920882 | 0.00844032 | 0.99989949 | -4.0225459 | 2.63392875 |
| LINC02321 | 2.36163634 | -4.6914571 | 2.68284743 | 0.00869561 | 0.99989949 | -4.0423847 | 2.62379521 |
| PKMP5 | -2.2513735 | -6.0293613 | -2.682022 | 0.00882886 | 0.99989949 | -4.0309932 | -2.6186108 |
| RPS7P1 | -0.8761312 | 0.27206874 | -2.6821721 | 0.00885901 | 0.99989949 | -4.1240024 | -2.6174474 |
| H1-8 | -1.716195 | -6.699243 | -2.6797534 | 0.00888998 | 0.99989949 | -4.0245614 | -2.6162564 |
| CST7 | 2.15492973 | -5.7132561 | 2.66292075 | 0.00929591 | 0.99989949 | -3.9917635 | 2.60097553 |
| GGT6 | 2.11038022 | -6.2366157 | 2.6571625 | 0.00933456 | 0.99989949 | -4.0123339 | 2.59955158 |
| RN7SL481P | 1.34544351 | -1.4619489 | 2.64931266 | 0.00967375 | 0.99989949 | -4.0861707 | 2.58727812 |
| HNRNPKP5 | 2.08664606 | -4.6494285 | 2.64812038 | 0.00974524 | 0.99989949 | -3.9685067 | 2.58474031 |
| RN7SKP30 | -2.1900888 | -4.9734944 | -2.6187347 | 0.01052564 | 0.99989949 | -4.0991658 | -2.5580649 |
| ZKSCAN7-AS1 | 0.63959598 | -0.1120548 | 2.60449179 | 0.01080488 | 0.99989949 | -4.0032523 | 2.54894662 |
| NDUFA5P4 | 1.79128482 | -6.3509994 | 2.60196909 | 0.01100478 | 0.99989949 | -4.0203269 | 2.54254691 |
| DDX50P2 | -2.4922597 | -5.5026326 | -2.6013956 | 0.01101958 | 0.99989949 | -4.0671619 | -2.5420774 |
| LINC00294 | 0.36874407 | 3.88768369 | 2.59698745 | 0.0111999 | 0.99989949 | -4.0598309 | 2.53639909 |
| RPL7P32 | -2.6515896 | -4.1943948 | -2.5738012 | 0.0117183 | 0.99989949 | -4.1244069 | -2.5205157 |
| MAN2A1-DT | 1.62763496 | -2.9072546 | 2.57827452 | 0.01173667 | 0.99989949 | -4.0631294 | 2.5199644 |
| MIR579 | 1.74531066 | -5.9303363 | 2.57731352 | 0.01189062 | 0.99989949 | -4.0186403 | 2.51537391 |
| MARCHF6-DT | -0.3730794 | 1.9408432 | -2.5715124 | 0.0119648 | 0.99989949 | -4.1176265 | -2.5131807 |
| RNY4P19 | 1.52868281 | -3.2954838 | 2.57106663 | 0.01213698 | 0.99989949 | -4.0571056 | 2.5081363 |
| ETV5-AS1 | -1.8704245 | -3.6811119 | -2.5539262 | 0.01253336 | 0.99989949 | -4.1694009 | -2.4967605 |
| MTND4P26 | 2.04677122 | -3.7141757 | 2.54875704 | 0.01267075 | 0.99989949 | -4.0893385 | 2.49289166 |
| TUBB4B | -0.4022297 | 7.78873355 | -2.5472614 | 0.01278112 | 0.99989949 | -4.1097957 | -2.4898105 |
| SNORD3B-2 | 2.19871646 | -3.8864309 | 2.54322126 | 0.01283858 | 0.99989949 | -4.0389179 | 2.48821568 |
| RNU6-444P | 1.94611859 | -5.5184858 | 2.53418452 | 0.01303923 | 0.99989949 | -4.1074322 | 2.48269598 |
| GGTLC3 | 1.42493145 | -4.5594655 | 2.53325557 | 0.01325845 | 0.99989949 | -4.0835986 | 2.47675051 |
| LINC02669 | -2.2402775 | -4.6752452 | -2.5229359 | 0.0137082 | 0.99989949 | -4.1132852 | -2.4648202 |
| CERS6-AS1 | 1.83614402 | -5.3748074 | 2.51832324 | 0.0137165 | 0.99989949 | -4.1322669 | 2.46460345 |
| LINC02323 | -2.5581948 | -4.8406165 | -2.5158555 | 0.01386356 | 0.99989949 | -4.1455051 | -2.4607794 |
| ATP5F1CP1 | -2.2110185 | -4.3488205 | -2.5089674 | 0.01405821 | 0.99989949 | -4.1398728 | -2.4557726 |
| KRT33B | -1.4337691 | -6.5589766 | -2.5082523 | 0.0141087 | 0.99989949 | -4.1223625 | -2.4544839 |
| KLC2-AS1 | 1.60101848 | -2.3562078 | 2.50473043 | 0.01423503 | 0.99989949 | -4.124608 | 2.45127718 |
| ATP6V1B1 | 1.71499162 | -4.8549926 | 2.50421726 | 0.01430462 | 0.99989949 | -4.0810915 | 2.44952155 |
| GYPE | 1.97794722 | -5.3076327 | 2.49901359 | 0.01447312 | 0.99989949 | -4.1000775 | 2.44530117 |
| DPY19L3-DT | -1.16798 | -1.6182806 | -2.4981982 | 0.01454886 | 0.99989949 | -4.1639464 | -2.4434183 |
| JMJD1C-AS1 | 0.86842225 | -1.5391188 | 2.48796727 | 0.01491725 | 0.99989949 | -4.1055606 | 2.43438173 |
| RNU6-8 | -2.4324041 | -4.6830367 | -2.4822048 | 0.01503308 | 0.99989949 | -4.1160478 | -2.4315813 |
| RGPD5 | -0.7840171 | -0.9904414 | -2.477036 | 0.01524755 | 0.99989949 | -4.132458 | -2.4264452 |
| SH3BGR | -0.836323 | -1.6644513 | -2.462354 | 0.01600445 | 0.99989949 | -4.1570886 | -2.408814 |
| NAP1L4P3 | -2.3186054 | -5.4662607 | -2.448865 | 0.01646659 | 0.99989949 | -4.1836032 | -2.3984063 |
| TPTE2-AS1 | 1.83753337 | -5.4479129 | 2.44221841 | 0.01656331 | 0.99989949 | -4.0676787 | 2.39626058 |
| LINC01722 | -1.8677198 | -5.635379 | -2.4458675 | 0.01661643 | 0.99989949 | -4.1379055 | -2.3950869 |
| TTC14-DT | -1.7890552 | -3.3347405 | -2.4373583 | 0.01698804 | 0.99989949 | -4.1769055 | -2.3869665 |
| HSD17B2-AS1 | 1.79190022 | -3.9986312 | 2.42098473 | 0.01751551 | 0.99989949 | -4.1406758 | 2.375704 |
| ELOCP2 | -2.3306136 | -4.8915333 | -2.4207909 | 0.01768092 | 0.99989949 | -4.145652 | -2.3722334 |
| LINC01996 | -1.5164119 | -6.581205 | -2.4204789 | 0.01773233 | 0.99989949 | -4.1333276 | -2.3711605 |
| CYP4Z1 | 2.01089457 | -4.8520975 | 2.42140725 | 0.01774192 | 0.99989949 | -4.1487583 | 2.37096075 |
| UQCRBP1 | -2.2520085 | -4.352528 | -2.4145596 | 0.01778996 | 0.99989949 | -4.2093353 | -2.369961 |
| H3C12 | -2.1473522 | -5.7225412 | -2.4167168 | 0.01789 | 0.99989949 | -4.1546008 | -2.367887 |
| BLTP3B-DT | 1.58675672 | -2.7141379 | 2.41710836 | 0.01794339 | 0.99989949 | -4.1015515 | 2.36678426 |
| SLC22A25 | 1.73900983 | -6.056162 | 2.41234968 | 0.01805103 | 0.99989949 | -4.1136865 | 2.36456969 |
| MTUS1-DT | -1.9602966 | -5.2577195 | -2.4121088 | 0.01808093 | 0.99989949 | -4.1298934 | -2.3639565 |
| TEDC2-AS1 | 1.10259394 | -0.9717114 | 2.39870449 | 0.01857378 | 0.99989949 | -4.160516 | 2.3539766 |
| JCHAIN | -2.1113312 | -5.0394275 | -2.3986777 | 0.01867147 | 0.99989949 | -4.167689 | -2.352026 |
| RNU6-298P | 1.74966914 | -6.2231803 | 2.38790424 | 0.01904767 | 0.99989949 | -4.1230367 | 2.34459671 |
| LINC01795 | -1.7674341 | -6.0421413 | -2.3920151 | 0.01907946 | 0.99989949 | -4.1528258 | -2.3439749 |
| MROH2A | -2.181024 | -5.467394 | -2.3895439 | 0.01920732 | 0.99989949 | -4.2072562 | -2.3414827 |
| ID2B | -2.2594876 | -5.1139368 | -2.3844962 | 0.01921407 | 0.99989949 | -4.1830416 | -2.3413515 |
| LINC01254 | -2.1759634 | -4.6792942 | -2.3816657 | 0.01953215 | 0.99989949 | -4.2212034 | -2.3352159 |
| FN1-DT | 1.98615648 | -4.3567098 | 2.37685495 | 0.0197637 | 0.99989949 | -4.1722722 | 2.33080402 |
| PRDM7 | -2.0919646 | -5.8849337 | -2.3693633 | 0.02010081 | 0.99989949 | -4.1938003 | -2.3244607 |
| RPL9P18 | -2.2367901 | -4.4623453 | -2.3600221 | 0.02065515 | 0.99989949 | -4.1810243 | -2.3142292 |
| TUBG1 | -0.2707471 | 5.22185874 | -2.3568207 | 0.02088123 | 0.99989949 | -4.1890651 | -2.310125 |
| LDLR | -0.5203145 | 5.7820918 | -2.3548137 | 0.0210268 | 0.99989949 | -4.1961967 | -2.3075029 |
| NPC1L1 | 1.89983911 | -3.5400652 | 2.3497605 | 0.02121002 | 0.99989949 | -4.2212281 | 2.30422486 |
| NANOGNBP2 | 2.12416064 | -5.7666867 | 2.33998281 | 0.02152538 | 0.99989949 | -4.1551588 | 2.29864023 |
| TCF15 | 1.85508897 | -3.5562832 | 2.34279427 | 0.0217146 | 0.99989949 | -4.1553307 | 2.29532332 |
| RIPPLY2 | 0.80348013 | 0.54529784 | 2.33844764 | 0.02193457 | 0.99989949 | -4.1546794 | 2.29149897 |
| LINC01053 | 1.86203913 | -6.2511621 | 2.33207169 | 0.02196522 | 0.99989949 | -4.1794154 | 2.29096873 |
| RPL24P6 | 1.72922239 | -4.7750398 | 2.33404837 | 0.02203517 | 0.99989949 | -4.1545333 | 2.28976105 |
| PHF10P1 | -1.789772 | -5.1148365 | -2.327845 | 0.02229343 | 0.99989949 | -4.1645438 | -2.285331 |
| SCARNA6 | 1.47102082 | -3.1251981 | 2.32429533 | 0.02239784 | 0.99989949 | -4.1605308 | 2.28355254 |
| LINC02637 | 1.30438913 | -3.2332961 | 2.32346069 | 0.02269141 | 0.99989949 | -4.176052 | 2.27859068 |
| TUBA1A | -0.4391495 | 11.8451718 | -2.3232779 | 0.02270646 | 0.99989949 | -4.1933282 | -2.2783378 |
| BOLA2-SMG1P6 | 1.09745266 | -2.1122335 | 2.31671783 | 0.02304303 | 0.99989949 | -4.1712768 | 2.27272053 |
| PTMAP9 | -0.936341 | -2.3363243 | -2.3151757 | 0.02313187 | 0.99989949 | -4.2165251 | -2.2712496 |
| TBC1D3D | 1.23205031 | -5.4806828 | 2.31253337 | 0.0233131 | 0.99989949 | -4.208577 | 2.26826435 |
| PDZPH1P | 2.02597151 | -5.5310602 | 2.30897054 | 0.02343269 | 0.99989949 | -4.1746368 | 2.26630546 |
| FAM246A | -1.8462922 | -3.8120885 | -2.3111453 | 0.02349125 | 0.99989949 | -4.2125039 | -2.2653494 |
| RPL3P4 | -0.5614565 | 1.05345846 | -2.3092682 | 0.0235118 | 0.99989949 | -4.2575946 | -2.2650143 |
| HIGD1B | 1.57669179 | -2.8868477 | 2.31026533 | 0.02364985 | 0.99989949 | -4.2398807 | 2.26277027 |
| TOX4P1 | -2.0945769 | -5.3464058 | -2.3048035 | 0.02366421 | 0.99989949 | -4.2207831 | -2.2625376 |
| HMGA1P4 | -1.6979342 | -6.520677 | -2.3039442 | 0.02378855 | 0.99989949 | -4.1921811 | -2.2605273 |
| PSMB11 | 1.63356734 | -6.1502014 | 2.30021046 | 0.02399677 | 0.99989949 | -4.1923591 | 2.25718103 |
| LINGO1-AS2 | 1.73221691 | -6.4176826 | 2.29400059 | 0.02415118 | 0.99989949 | -4.1802466 | 2.25471575 |
| RPL12P47 | 1.85332797 | -3.2067614 | 2.2930304 | 0.02423372 | 0.99989949 | -4.1884223 | 2.25340351 |
| FSD2 | -0.7616235 | -0.9142327 | -2.2945451 | 0.02428629 | 0.99989949 | -4.2132165 | -2.2525698 |
| HCCS-DT | -0.8325941 | -1.590536 | -2.2935546 | 0.02438774 | 0.99989949 | -4.2209017 | -2.2509652 |
| RPL21P110 | 1.92210909 | -5.5622317 | 2.29177979 | 0.02443752 | 0.99989949 | -4.2183339 | 2.25017998 |
| NDUFB4P8 | 1.7553845 | -4.6917739 | 2.28899903 | 0.02446784 | 0.99989949 | -4.1987957 | 2.24970239 |
| RDH13 | 0.27761661 | 4.00905311 | 2.29385976 | 0.02448037 | 0.99989949 | -4.2000235 | 2.24950518 |
| DSG2-AS1 | -1.8063266 | -5.2491178 | -2.2880556 | 0.02449042 | 0.99989949 | -4.1750434 | -2.2493471 |
| RPL15P11 | -1.8456603 | -4.3595814 | -2.2906104 | 0.02468605 | 0.99989949 | -4.2204784 | -2.2462805 |
| LINC02878 | 0.56732214 | -0.2112072 | 2.28931876 | 0.02469208 | 0.99989949 | -4.1782034 | 2.24618628 |
| FAM133DP | -0.7020543 | -1.3029939 | -2.2864957 | 0.02482209 | 0.99989949 | -4.225816 | -2.2441603 |
| ARIH2OS | -0.3644331 | 1.60521769 | -2.2856233 | 0.02489864 | 0.99989949 | -4.2124338 | -2.2429717 |
| GP6-AS1 | 0.51385506 | 0.78040247 | 2.28216671 | 0.02505062 | 0.99989949 | -4.1802805 | 2.2406213 |
| SMCO1 | 1.96872666 | -4.6191737 | 2.27951641 | 0.02521824 | 0.99989949 | -4.1999963 | 2.23804316 |
| MLIP-AS1 | -2.0578377 | -6.0269491 | -2.2793485 | 0.02536178 | 0.99989949 | -4.2120012 | -2.2358472 |
| CASC17 | 1.69321346 | -5.6018454 | 2.27577192 | 0.02549658 | 0.99989949 | -4.2026813 | 2.23379474 |
| MIR93 | -1.4295925 | -5.5861083 | -2.2754675 | 0.02551607 | 0.99989949 | -4.2051798 | -2.2334988 |
| PDE6H | -2.0209361 | -4.9830876 | -2.2690602 | 0.02569277 | 0.99989949 | -4.220202 | -2.2308243 |
| ARHGAP19-SLIT1 | 0.82230534 | -0.7161272 | 2.2727987 | 0.02570435 | 0.99989949 | -4.2312886 | 2.23064963 |
| RPL21P125 | 0.93620069 | -1.3129408 | 2.27139184 | 0.02578815 | 0.99989949 | -4.2237511 | 2.22938726 |
| C12orf60 | -0.455241 | 0.75965344 | -2.2701968 | 0.02591897 | 0.99989949 | -4.2513018 | -2.2274237 |
| LINC01800 | 1.85789613 | -4.048997 | 2.26866651 | 0.02604057 | 0.99989949 | -4.2279156 | 2.22560616 |
| PSME2P1 | -1.1140452 | -1.7751782 | -2.2681917 | 0.02605277 | 0.99989949 | -4.2440885 | -2.2254242 |
| SNRPEP4 | -1.2370352 | -2.5663837 | -2.2714436 | 0.02606105 | 0.99989949 | -4.2374769 | -2.2253008 |
| FTH1P4 | -2.1385661 | -4.3621435 | -2.2551664 | 0.02672593 | 0.99989949 | -4.2402619 | -2.2154975 |
| COX6A1P2 | -0.7300102 | -1.0407707 | -2.2504281 | 0.02708052 | 0.99989949 | -4.229132 | -2.2103551 |
| ENPP7P1 | 1.32446389 | -4.3267755 | 2.24994976 | 0.0273103 | 0.99989949 | -4.1654856 | 2.20705375 |
| MED4-AS1 | 1.82350978 | -5.385696 | 2.24334263 | 0.02735968 | 0.99989949 | -4.1919522 | 2.20634752 |
| BCL2L2-PABPN1 | 0.82258523 | -1.4035899 | 2.24303754 | 0.02755053 | 0.99989949 | -4.1770003 | 2.20362781 |
| RASGRP4 | 1.11489018 | -1.8813854 | 2.23432762 | 0.02827617 | 0.99989949 | -4.2097205 | 2.19343343 |
| RBISP2 | -1.6388031 | -6.2617811 | -2.2309673 | 0.02842112 | 0.99989949 | -4.2231855 | -2.191424 |
| GAU1 | 0.57885807 | -0.1062825 | 2.23133037 | 0.02842306 | 0.99989949 | -4.2031543 | 2.19139718 |
| TCAP | 0.48280148 | 1.6907872 | 2.22947733 | 0.02855652 | 0.99989949 | -4.1937095 | 2.18955505 |
| SLC25A40 | 0.20591824 | 4.54448786 | 2.22854913 | 0.02872873 | 0.99989949 | -4.2269158 | 2.18718896 |
| LINC01976 | 1.6788938 | -6.4344487 | 2.21560417 | 0.0294809 | 0.99989949 | -4.2200315 | 2.17699557 |
| RNU6-181P | 1.35915738 | -3.4425364 | 2.20906598 | 0.02974953 | 0.99989949 | -4.2300981 | 2.17340922 |
| IFNA5 | -1.6606502 | -6.1427194 | -2.2088558 | 0.02994679 | 0.99989949 | -4.2346677 | -2.1707935 |
| PAGE2 | 1.67927292 | -6.531932 | 2.2052078 | 0.03002138 | 0.99989949 | -4.2269859 | 2.16980817 |
| ZNF157 | 0.50104817 | 1.33004058 | 2.20884201 | 0.03009076 | 0.99989949 | -4.2404055 | 2.16889362 |
| HAUS6P3 | -1.2505727 | -2.9576587 | -2.2072793 | 0.03011917 | 0.99989949 | -4.2505309 | -2.1685196 |
| LINC01284 | 0.83193157 | -1.5078183 | 2.20921467 | 0.03016628 | 0.99989949 | -4.2372565 | 2.1679002 |
| RPL8P4 | -2.188963 | -5.5697281 | -2.2043949 | 0.03025566 | 0.99989949 | -4.2508199 | -2.1667272 |
| DHFRP1 | -1.1109118 | -3.0801332 | -2.2031078 | 0.03048574 | 0.99989949 | -4.2645745 | -2.1637214 |
| MIR5692C2 | 1.68279892 | -5.1791026 | 2.19897949 | 0.03084958 | 0.99989949 | -4.2234521 | 2.15900753 |
| SBSN | 1.62380923 | -3.2705902 | 2.19535773 | 0.03103748 | 0.99989949 | -4.1888262 | 2.15659184 |
| COQ7-DT | 1.49790781 | -4.5787554 | 2.19240035 | 0.03114902 | 0.99989949 | -4.233621 | 2.15516374 |
| LINC02201 | -1.5639831 | -6.2053239 | -2.1928824 | 0.03116591 | 0.99989949 | -4.2338353 | -2.1549479 |
| CCDC196 | 1.36381529 | -2.7715639 | 2.19196126 | 0.03117672 | 0.99989949 | -4.2469096 | 2.15480979 |
| LINC01679 | -2.0483791 | -3.3484891 | -2.1934832 | 0.03119032 | 0.99989949 | -4.2891081 | -2.1546361 |
| BCAS2P2 | -2.0397763 | -5.8369176 | -2.1889727 | 0.03120192 | 0.99989949 | -4.2594462 | -2.154488 |
| ATP5MGP8 | 1.63507301 | -4.9818706 | 2.18590364 | 0.03168086 | 0.99989949 | -4.2356585 | 2.14841432 |
| MFSD13B | 1.47328647 | -3.0062797 | 2.18430041 | 0.03173063 | 0.99989949 | -4.2413712 | 2.1477876 |
| C15orf32 | -1.8139501 | -4.1342447 | -2.1831005 | 0.03186509 | 0.99989949 | -4.2836142 | -2.1460989 |
| RPS4XP16 | 0.74779274 | -1.3160508 | 2.18118298 | 0.03197898 | 0.99989949 | -4.2387721 | 2.14467328 |
| MIR628 | 1.05232869 | -2.815812 | 2.18223742 | 0.0320235 | 0.99989949 | -4.2327859 | 2.14411718 |
| DPP3-DT | 0.34313022 | 2.15238081 | 2.17809025 | 0.03247336 | 0.99989949 | -4.2190262 | 2.13853482 |
| PPIAP32 | 1.61491634 | -4.5857683 | 2.1713465 | 0.03279428 | 0.99989949 | -4.2176025 | 2.13459289 |
| CHCHD2P6 | -1.1651852 | -2.2631155 | -2.1664269 | 0.03322237 | 0.99989949 | -4.2667367 | -2.1293856 |
| PRADC1P1 | 1.73058746 | -3.5566296 | 2.16259602 | 0.03376407 | 0.99989949 | -4.26597 | 2.12287804 |
| RNU2-37P | 1.98683169 | -5.6082489 | 2.1565676 | 0.03391426 | 0.99989949 | -4.2541024 | 2.12108959 |
| OR52I2 | 1.65601572 | -6.0502061 | 2.1567269 | 0.03395906 | 0.99989949 | -4.2026966 | 2.12055745 |
| SLC6A17-AS1 | 1.50274327 | -5.3792677 | 2.15515392 | 0.03412413 | 0.99989949 | -4.2483849 | 2.11860187 |
| LINC01229 | -1.3859648 | -2.4553774 | -2.1518946 | 0.03436998 | 0.99989949 | -4.2918634 | -2.1157041 |
| CHRNA2 | 1.68471568 | -3.6229584 | 2.14939326 | 0.03457221 | 0.99989949 | -4.2386868 | 2.11333372 |
| RPL24P2 | -1.9353311 | -4.4770787 | -2.150667 | 0.03466475 | 0.99989949 | -4.2887022 | -2.1122531 |
| MIR4477B | 1.18147604 | -2.6933495 | 2.14870959 | 0.03467417 | 0.99989949 | -4.2557553 | 2.1121432 |
| TARS3 | 0.18875007 | 4.80555974 | 2.14771523 | 0.03492997 | 0.99989949 | -4.257637 | 2.10916925 |
| CDC42-IT1 | 1.79945103 | -4.3736173 | 2.13789698 | 0.03527984 | 0.99989949 | -4.2476922 | 2.10513173 |
| LRTOMT | 0.87096035 | -2.6147169 | 2.14372373 | 0.03532667 | 0.99989949 | -4.2608153 | 2.10459391 |
| ITFG2-AS1 | -1.8999756 | -5.1548763 | -2.1358094 | 0.03545402 | 0.99989949 | -4.2813186 | -2.1031344 |
| MIR331 | 1.30843404 | -3.9246236 | 2.13497187 | 0.0356591 | 0.99989949 | -4.2563195 | 2.10079347 |
| LINC02405 | -1.4629172 | -6.1824692 | -2.1342616 | 0.03581956 | 0.99989949 | -4.262208 | -2.0989698 |
| TFB1M | -0.2404458 | 2.29310614 | -2.1329769 | 0.036011 | 0.99989949 | -4.2859871 | -2.0968032 |
| TMEM183AP3 | 1.82891113 | -4.5858943 | 2.13357551 | 0.03604068 | 0.99989949 | -4.2823459 | 2.09646822 |
| ZC3HC1 | -0.1531885 | 4.06327547 | -2.1324357 | 0.03611529 | 0.99989949 | -4.2763245 | -2.095627 |
| CFAP20DC | -0.571817 | 1.03038361 | -2.1313127 | 0.03614024 | 0.99989949 | -4.3207614 | -2.095346 |
| RAB44 | 1.95852172 | -3.8711614 | 2.12984211 | 0.03615911 | 0.99989949 | -4.2837851 | 2.09513371 |
| GPC5-AS1 | 1.39834511 | -5.0960598 | 2.12932191 | 0.0363029 | 0.99989949 | -4.255707 | 2.09351839 |
| FAM228A | -0.4303271 | 0.41567214 | -2.1319766 | 0.03633912 | 0.99989949 | -4.2635018 | -2.0931124 |
| ARHGAP31-AS1 | -0.7030281 | -1.1826068 | -2.1277617 | 0.03642211 | 0.99989949 | -4.3161513 | -2.0921835 |
| LINC01877 | -1.8951385 | -5.4657499 | -2.1261308 | 0.03652252 | 0.99989949 | -4.2623237 | -2.0910619 |
| AFG3L2P1 | 1.78649898 | -6.055842 | 2.12235442 | 0.03657064 | 0.99989949 | -4.2455007 | 2.09052526 |
| CRYGD | -1.6758735 | -3.9563032 | -2.1250733 | 0.03668902 | 0.99989949 | -4.2955402 | -2.0892079 |
| RN7SKP87 | 1.08735747 | -2.2054585 | 2.12387188 | 0.03676785 | 0.99989949 | -4.2692886 | 2.08833254 |
| LINC02393 | -1.4010162 | -6.0199954 | -2.1226723 | 0.03682641 | 0.99989949 | -4.2661796 | -2.0876833 |
| USP12P1 | 1.84162466 | -4.393915 | 2.11802947 | 0.0369792 | 0.99989949 | -4.2651403 | 2.08599367 |
| MMP10 | -1.8757294 | -3.0430633 | -2.1176281 | 0.03744217 | 0.99989949 | -4.3684329 | -2.0809097 |
| DGKZP1 | -0.5077422 | -0.5894459 | -2.1154713 | 0.03749595 | 0.99989949 | -4.2953417 | -2.0803227 |
| RN7SL251P | -1.6470651 | -5.7153499 | -2.114768 | 0.03752038 | 0.99989949 | -4.2841378 | -2.0800562 |
| UBE2V1P14 | 1.48785909 | -5.6832764 | 2.11310451 | 0.03765149 | 0.99989949 | -4.2638011 | 2.07862867 |
| GATA6-AS1 | -1.6961925 | -5.7672725 | -2.1133575 | 0.0376872 | 0.99989949 | -4.2694997 | -2.0782407 |
| ADH5P4 | -1.6612582 | -3.2622658 | -2.1096482 | 0.03769028 | 0.99989949 | -4.3081078 | -2.0782072 |
| ITPKB-IT1 | -1.5874617 | -6.0282751 | -2.1123475 | 0.03778426 | 0.99989949 | -4.2503161 | -2.0771875 |
| LINC02765 | -1.9900381 | -3.8435016 | -2.1131633 | 0.03778853 | 0.99989949 | -4.3021515 | -2.0771411 |
| RNU6-705P | 1.28806942 | -4.2665243 | 2.11042837 | 0.03794781 | 0.99989949 | -4.2871957 | 2.07541799 |
| FAM209A | 1.42190786 | -3.9181435 | 2.10739594 | 0.03809853 | 0.99989949 | -4.2706079 | 2.07379311 |
| LAIR1 | -1.5179478 | -5.8309604 | -2.107075 | 0.03816724 | 0.99989949 | -4.3040838 | -2.0730542 |
| ORM2 | 1.51102736 | -3.2579523 | 2.10531384 | 0.03827327 | 0.99989949 | -4.2778822 | 2.07191611 |
| MTND4LP1 | -1.1295394 | -6.5677691 | -2.10425 | 0.03841326 | 0.99989949 | -4.2731754 | -2.0704175 |
| PKP4-AS1 | 1.10454476 | -1.5952759 | 2.10255575 | 0.03854646 | 0.99989949 | -4.2749488 | 2.068996 |
| HSP90AB2P | -1.352176 | -2.6074593 | -2.1011187 | 0.03862298 | 0.99989949 | -4.2760524 | -2.0681812 |
| FKBP1C | -0.8626678 | -1.6941259 | -2.1007969 | 0.03869559 | 0.99989949 | -4.296071 | -2.0674094 |
| FNBP1P1 | 0.30006442 | 2.62496135 | 2.10237419 | 0.03870269 | 0.99989949 | -4.2826497 | 2.06733403 |
| NUP107-DT | -1.425711 | -2.8079811 | -2.099784 | 0.03883147 | 0.99989949 | -4.3223562 | -2.0659683 |
| RNU5F-1 | 1.7073586 | -4.5613655 | 2.10004071 | 0.03893012 | 0.99989949 | -4.255096 | 2.06492475 |
| HMGN1P37 | -1.7530141 | -3.7661629 | -2.0960547 | 0.03914324 | 0.99989949 | -4.3083957 | -2.0626779 |
| LINC01226 | -1.724768 | -5.7655348 | -2.0937104 | 0.03951916 | 0.99989949 | -4.2947439 | -2.0587399 |
| MCIDAS | -1.1800119 | -5.9945075 | -2.0895403 | 0.03977853 | 0.99989949 | -4.2562272 | -2.0560413 |
| RPL6P6 | 1.87274282 | -4.5412875 | 2.08840826 | 0.03981663 | 0.99989949 | -4.3042331 | 2.05564617 |
| MAL2-AS1 | 1.88163244 | -5.4481785 | 2.08898341 | 0.03981888 | 0.99989949 | -4.2892551 | 2.05562293 |
| TACR3-AS1 | 1.31629843 | -5.4594123 | 2.08901286 | 0.03987614 | 0.99989949 | -4.3046368 | 2.05502963 |
| ALLC | 1.88678991 | -3.56839 | 2.08420699 | 0.04000409 | 0.99989949 | -4.2889674 | 2.05370671 |
| ZNF22-AS1 | -0.2815274 | 2.52517709 | -2.0867816 | 0.04011082 | 0.99989949 | -4.3102438 | -2.0526059 |
| IGLV1-50 | 1.76718573 | -4.5025046 | 2.08574105 | 0.04011546 | 0.99989949 | -4.2804263 | 2.05255809 |
| SNORD53B | 1.56675528 | -4.7950282 | 2.08269249 | 0.04015766 | 0.99989949 | -4.2559438 | 2.0521235 |
| ALAD | 0.15262713 | 5.72627279 | 2.08588297 | 0.04016456 | 0.99989949 | -4.2886436 | 2.05205252 |
| PICSAR | -1.503777 | -6.1718027 | -2.0844996 | 0.04021135 | 0.99989949 | -4.286168 | -2.0515712 |
| RPL7P11 | -1.8622805 | -6.141077 | -2.0796763 | 0.04041594 | 0.99989949 | -4.3009951 | -2.0494724 |
| ASXL3-DT | 1.55847509 | -5.1654218 | 2.08295869 | 0.04045901 | 0.99989949 | -4.2850703 | 2.04903172 |
| OR8S1 | 1.51105929 | -6.1331668 | 2.0823899 | 0.040511 | 0.99989949 | -4.2540654 | 2.04850037 |
| MAPK6P3 | -1.5655636 | -3.828636 | -2.0799059 | 0.04064833 | 0.99989949 | -4.301705 | -2.0470993 |
| RBBP4P1 | -1.212695 | -2.1276815 | -2.0729696 | 0.04105087 | 0.99989949 | -4.3365655 | -2.0430158 |
| ITGBL1 | -1.8466245 | -3.995869 | -2.0754168 | 0.04111332 | 0.99989949 | -4.2737976 | -2.0423853 |
| LINC02092 | -1.5150367 | -6.4703951 | -2.0745926 | 0.04114638 | 0.99989949 | -4.2883472 | -2.0420519 |
| COX7CP1 | -1.8143105 | -4.0247227 | -2.0728776 | 0.04131866 | 0.99989949 | -4.2965885 | -2.0403181 |
| RPL22L1 | -0.3827522 | 2.39455067 | -2.0705585 | 0.04166116 | 0.99989949 | -4.3233038 | -2.0368891 |
| LINC01307 | 1.63849746 | -5.8783973 | 2.06939377 | 0.04167844 | 0.99989949 | -4.2996702 | 2.03671665 |
| MTND1P23 | 1.09589223 | 2.1666166 | 2.069729 | 0.04169949 | 0.99989949 | -4.3078679 | 2.03650681 |
| DTX2P1 | 1.50856435 | -5.6929227 | 2.06570846 | 0.04200138 | 0.99989949 | -4.2609032 | 2.03350646 |
| PAICSP1 | 0.56306413 | -0.0323884 | 2.06403951 | 0.04223388 | 0.99989949 | -4.2833097 | 2.03120816 |
| RPL7P9 | -0.5838167 | 0.76375786 | -2.0634501 | 0.04233471 | 0.99989949 | -4.334466 | -2.0302148 |
| SNORD113-2 | 1.40510459 | -6.5088095 | 2.0609411 | 0.04245193 | 0.99989949 | -4.2882113 | 2.02906248 |
| PIGCP1 | 0.28896317 | 2.04705564 | 2.06194069 | 0.04248594 | 0.99989949 | -4.2885276 | 2.02872856 |
| TCP11L1 | 0.22563712 | 4.43203995 | 2.06120764 | 0.04256147 | 0.99989949 | -4.291762 | 2.02798803 |
| ANXA8 | -1.8076667 | -6.0884374 | -2.057283 | 0.04257461 | 0.99989949 | -4.2715511 | -2.0278593 |
| PAPOLA-DT | -0.3669612 | 0.02059232 | -2.0601669 | 0.042599 | 0.99989949 | -4.286644 | -2.0276204 |
| KRT18P50 | -1.592283 | -6.2520056 | -2.0591979 | 0.0426893 | 0.99989949 | -4.2921198 | -2.0267372 |
| EIF2AP4 | 2.05805833 | -5.6044092 | 2.05525075 | 0.04276983 | 0.99989949 | -4.2904031 | 2.02595078 |
| LINC03025 | 1.72772188 | -5.9596842 | 2.05463411 | 0.04285809 | 0.99989949 | -4.2888455 | 2.02509032 |
| TPSP2 | 1.6744079 | -5.2896072 | 2.05455819 | 0.0428709 | 0.99989949 | -4.291714 | 2.0249656 |
| LINC00523 | 1.9277906 | -5.698662 | 2.05325077 | 0.04296767 | 0.99989949 | -4.2882394 | 2.02402413 |
| DSG1 | -1.9811296 | -4.7851406 | -2.0560898 | 0.04299259 | 0.99989949 | -4.3113556 | -2.023782 |
| ZNF256 | 0.265167 | 3.23734613 | 2.05444714 | 0.04319788 | 0.99989949 | -4.2863718 | 2.0217917 |
| LCMT1-AS1 | 0.34683057 | 1.05708313 | 2.05370363 | 0.04323844 | 0.99989949 | -4.2921267 | 2.02139939 |
| ENPP7P8 | 1.52659373 | -4.4838268 | 2.0535504 | 0.04345867 | 0.99989949 | -4.2639421 | 2.01927478 |
| SMLR1 | 1.44606855 | -6.3392295 | 2.04891322 | 0.04360636 | 0.99989949 | -4.2517797 | 2.01785506 |
| TUBB2B | -0.3971584 | 9.89740579 | -2.0495464 | 0.04370341 | 0.99989949 | -4.3052568 | -2.0169244 |
| TNS4 | -1.7001278 | -6.5177488 | -2.0455829 | 0.04390825 | 0.99989949 | -4.3033029 | -2.0149656 |
| PYCR3 | -0.2397797 | 3.985586 | -2.0458347 | 0.04410653 | 0.99989949 | -4.3181897 | -2.013077 |
| TUBB1 | 0.41802701 | -0.1029773 | 2.04580943 | 0.04437314 | 0.99989949 | -4.2958879 | 2.01054874 |
| PPIEL | 1.4303684 | -3.702799 | 2.04076874 | 0.04454225 | 0.99989949 | -4.270682 | 2.00895172 |
| LENEP | -1.8718207 | -5.1642034 | -2.0380696 | 0.04454856 | 0.99989949 | -4.3217862 | -2.0088923 |
| RNU6-460P | 1.40484998 | -4.3105118 | 2.0411501 | 0.04457238 | 0.99989949 | -4.3122693 | 2.00866776 |
| LINC03086 | 1.11857454 | -3.5483279 | 2.03636677 | 0.04502464 | 0.99989949 | -4.2878173 | 2.00442416 |
| USE1 | -0.1773786 | 4.21975207 | -2.0325696 | 0.04537485 | 0.99989949 | -4.304233 | -2.0011628 |
| LINC01068 | 1.14665279 | -3.4718026 | 2.02932905 | 0.04539775 | 0.99989949 | -4.238302 | 2.00095028 |
| YEATS4 | -0.2651479 | 4.52284542 | -2.0305098 | 0.0456061 | 0.99989949 | -4.3118061 | -1.9990208 |
| PGBD4 | 0.24358176 | 2.89324895 | 2.02993323 | 0.04570338 | 0.99989949 | -4.3177731 | 1.99812248 |
| ZKSCAN8P2 | 1.55855773 | -5.9306356 | 2.02902999 | 0.04576557 | 0.99989949 | -4.3098527 | 1.99754911 |
| MIR194-2HG | 1.59515965 | -5.1717786 | 2.02692231 | 0.04592503 | 0.99989949 | -4.2587222 | 1.99608168 |
| L3MBTL4 | -0.317684 | 1.86926839 | -2.0264853 | 0.04605085 | 0.99989949 | -4.3009871 | -1.994927 |
| HYDIN2 | 0.95067822 | -1.4643654 | 2.02457858 | 0.0462472 | 0.99989949 | -4.2929539 | 1.99313008 |
| OR5AK4P | -1.9457429 | -5.8715191 | -2.0240806 | 0.04631049 | 0.99989949 | -4.3305379 | -1.9925523 |
| NUBP1 | -0.2699845 | 2.59414554 | -2.0255199 | 0.04631178 | 0.99989949 | -4.3091805 | -1.9925406 |
| NPM1P27 | -0.5309565 | 2.34892197 | -2.0237347 | 0.04631666 | 0.99989949 | -4.3361695 | -1.992496 |
| CASK-AS1 | 1.60856473 | -4.4094279 | 2.01995632 | 0.04638258 | 0.99989949 | -4.3073373 | 1.99189496 |
| TJP3 | 0.66124626 | -0.2920926 | 2.02283241 | 0.04645402 | 0.99989949 | -4.3010074 | 1.9912444 |
| PPIL1P1 | 1.60438585 | -6.0574798 | 2.01897167 | 0.0464831 | 0.99989949 | -4.2952336 | 1.99097984 |
| GOT2P6 | -1.2616773 | -6.6539396 | -2.0215335 | 0.04649598 | 0.99989949 | -4.3063031 | -1.9908628 |
| ADORA2A-AS1 | 0.75695655 | -0.1647313 | 2.01982751 | 0.04670911 | 0.99989949 | -4.2848958 | 1.98892834 |
| ZNF451 | 0.18215653 | 5.72282969 | 2.01876044 | 0.04690613 | 0.99989949 | -4.3118786 | 1.98714684 |
| HMGN1P38 | 0.84208063 | -2.1640527 | 2.01694748 | 0.04693346 | 0.99989949 | -4.3202547 | 1.98690018 |
| ZNF879 | 0.22383651 | 3.16101777 | 2.01694357 | 0.04706115 | 0.99989949 | -4.3058394 | 1.9857495 |
| CCL4L2 | 1.3620082 | -6.6877502 | 2.014004 | 0.04730327 | 0.99989949 | -4.3012178 | 1.98357474 |
| LINC01614 | -2.055205 | -5.4063968 | -2.0122444 | 0.04747387 | 0.99989949 | -4.3086725 | -1.982048 |
| SIRPAP1 | -1.5534511 | -3.4888193 | -2.0096029 | 0.04778981 | 0.99989949 | -4.3465252 | -1.9792327 |
| GGCTP1 | 1.46803225 | -4.03362 | 2.01117714 | 0.04779816 | 0.99989949 | -4.3085144 | 1.97915849 |
| LINC01994 | 1.56181641 | -5.2150263 | 2.0090257 | 0.04783772 | 0.99989949 | -4.2908082 | 1.97880715 |
| NUDT8 | -0.4366866 | 1.49949893 | -2.0078135 | 0.04805519 | 0.99989949 | -4.3300342 | -1.9768801 |
| FAM153A | 0.97325889 | -1.866127 | 2.00726995 | 0.04814927 | 0.99989949 | -4.3303375 | 1.97604861 |
| ADGRF1 | -1.5757516 | -6.2902506 | -2.0068304 | 0.04818222 | 0.99989949 | -4.3279828 | -1.9757577 |
| SH2D7 | 0.49548866 | -0.157697 | 2.0060668 | 0.04826723 | 0.99989949 | -4.3068855 | 1.97500813 |
| RN7SL806P | 1.63967168 | -5.8504237 | 2.00137279 | 0.04841219 | 0.99989949 | -4.2966689 | 1.97373229 |
| FLJ30679 | -1.5660898 | -3.0309607 | -2.0017489 | 0.04859986 | 0.99989949 | -4.3466048 | -1.9720854 |
| FBXL20 | 0.25574375 | 6.02280602 | 1.99827926 | 0.04898987 | 0.99989949 | -4.3180266 | 1.96867982 |
| OGA | 0.20003891 | 8.20619322 | 1.99760807 | 0.04914601 | 0.99989949 | -4.3220081 | 1.96732278 |
| RNU5D-1 | -1.5848762 | -6.1013404 | -1.9942565 | 0.04921362 | 0.99989949 | -4.3121489 | -1.9667363 |
| MTFMT | -0.1869255 | 3.54396326 | -1.9961204 | 0.0492406 | 0.99989949 | -4.3134996 | -1.9665025 |
| SOCS1 | -0.6462727 | 0.82546113 | -1.9957428 | 0.04931097 | 0.99989949 | -4.3423034 | -1.965893 |
| SNRK-AS1 | 0.54126768 | -0.1002548 | 1.9944583 | 0.0494144 | 0.99989949 | -4.2918623 | 1.96499862 |
| CDRT15 | 1.17582014 | -2.9523009 | 1.99485831 | 0.04943873 | 0.99989949 | -4.3179216 | 1.96478841 |
| LINC02066 | 1.48699868 | -5.8366135 | 1.99365922 | 0.04957523 | 0.99989949 | -4.3134684 | 1.96361096 |
| LINC02822 | -1.4002909 | -6.0940433 | -1.9917859 | 0.04973239 | 0.99989949 | -4.3253106 | -1.9622586 |

**Supplementary Table S7:** Differentially Expressed Genes after DOI at Six Hours

| **Gene Name** | **logFC** | **AveExpr** | **t** | **P.Value** | **adj.P.Val** | **B** | **z.std** |
| --- | --- | --- | --- | --- | --- | --- | --- |
| LRRC52-AS1 | -3.3612374 | -6.2565009 | -4.3864609 | 3.3941E-05 | 0.75828626 | -3.0698475 | -4.1452692 |
| CLDN7 | 2.65259747 | -5.2769434 | 3.60204631 | 0.00054027 | 0.99993162 | -3.4257276 | 3.45995274 |
| RPS25P9 | -2.4857278 | -5.6573957 | -3.4289953 | 0.00095877 | 0.99993162 | -3.6530359 | -3.3023525 |
| CASK-AS1 | 2.5634005 | -4.4094279 | 3.3807448 | 0.00107345 | 0.99993162 | -3.5852022 | 3.27053212 |
| LINC01996 | -2.1662728 | -6.581205 | -3.3858693 | 0.00109585 | 0.99993162 | -3.6737685 | -3.2646883 |
| H2AZP1 | 2.21341265 | -6.3243489 | 3.36919398 | 0.00116525 | 0.99993162 | -3.6621222 | 3.24725162 |
| MIR4754 | -2.1485319 | -3.2499722 | -3.3282922 | 0.00127263 | 0.99993162 | -3.8296956 | -3.2220811 |
| ALG1L7P | -1.3981031 | -1.6127968 | -3.2840862 | 0.00152008 | 0.99993162 | -3.8636079 | -3.170823 |
| GASAL1 | -0.812579 | -0.3818419 | -3.2205147 | 0.00185537 | 0.99993162 | -3.8676648 | -3.1124578 |
| DAB1-AS1 | 2.22340044 | -6.3050771 | 3.19984837 | 0.00194964 | 0.99993162 | -3.7301816 | 3.09779838 |
| COL25A1-DT | -2.3829373 | -3.7689385 | -3.1526152 | 0.00220943 | 0.99993162 | -3.9105653 | -3.060533 |
| LSAMP-AS1 | 1.98314307 | -5.9648768 | 3.12525227 | 0.00245972 | 0.99993162 | -3.7367429 | 3.0282528 |
| SETD5 | 0.22774909 | 7.99606487 | 3.11692714 | 0.00254979 | 0.99993162 | -3.8295746 | 3.01736923 |
| CTNNA1-AS1 | -2.6299374 | -4.5926796 | -3.1011829 | 0.0026792 | 0.99993162 | -3.9182675 | -3.0023313 |
| RPL27AP | -2.4827951 | -5.5127441 | -3.0626561 | 0.00290712 | 0.99993162 | -3.8236794 | -2.9773921 |
| QRSL1P3 | 2.50844359 | -4.0065355 | 3.04835189 | 0.00313493 | 0.99993162 | -3.8135275 | 2.95418806 |
| RPL6P6 | 2.60088542 | -4.5412875 | 3.0068375 | 0.00348912 | 0.99993162 | -3.8785761 | 2.92099766 |
| DYNLRB2-AS1 | -2.1448695 | -3.193434 | -3.0018826 | 0.00357014 | 0.99993162 | -4.000694 | -2.9138389 |
| FAM133DP | -0.9534606 | -1.3029939 | -3.000261 | 0.00357917 | 0.99993162 | -3.9512342 | -2.9130501 |
| H2AC14 | -1.943225 | -6.6666084 | -2.9915593 | 0.00367511 | 0.99993162 | -3.8573123 | -2.9047801 |
| PRKAR1B-AS2 | 2.23762482 | -4.8912996 | 2.9566209 | 0.00407656 | 0.99993162 | -3.8073032 | 2.87217624 |
| ADCY10 | 1.03843188 | -1.1470909 | 2.94321114 | 0.00425161 | 0.99993162 | -3.8806009 | 2.85886404 |
| MTATP6P27 | 2.06959478 | -3.150559 | 2.92138787 | 0.00442571 | 0.99993162 | -3.888605 | 2.84610881 |
| RCAN2-DT | 2.13978651 | -5.0178463 | 2.90169999 | 0.00478723 | 0.99993162 | -3.9225867 | 2.82101283 |
| MIR5692A1 | 2.09914577 | -5.6239101 | 2.89862004 | 0.00480072 | 0.99993162 | -3.8994002 | 2.82010966 |
| POU2F1 | 0.37208958 | 6.39726192 | 2.89867011 | 0.00480574 | 0.99993162 | -3.9263974 | 2.81977426 |
| LINC02766 | -2.4239719 | -5.2931019 | -2.8914108 | 0.00492325 | 0.99993162 | -3.9386731 | -2.8120128 |
| MROCKI | 0.8936301 | -0.2922865 | 2.88772001 | 0.00497675 | 0.99993162 | -3.9412459 | 2.80853493 |
| TEX53 | 1.90008728 | -4.4085674 | 2.87684089 | 0.00509865 | 0.99993162 | -3.8800053 | 2.80073427 |
| PARP11-AS1 | 0.59035587 | -0.3451171 | 2.87849286 | 0.00511671 | 0.99993162 | -3.9465318 | 2.79959295 |
| SPON1-AS1 | 1.78055485 | -4.170589 | 2.86485888 | 0.00520867 | 0.99993162 | -3.9095426 | 2.79383734 |
| ANKRD33BP10 | -2.0872816 | -6.1505089 | -2.8658173 | 0.00529756 | 0.99993162 | -3.9731316 | -2.7883605 |
| MIR7152 | 1.45911323 | -3.6804902 | 2.86535208 | 0.00532341 | 0.99993162 | -3.9331279 | 2.78678309 |
| TMEM263-DT | -0.4166075 | 1.30876501 | -2.837846 | 0.00573937 | 0.99993162 | -3.9894908 | -2.7623116 |
| LINC01399 | -2.3783259 | -5.850495 | -2.8332982 | 0.00578966 | 0.99993162 | -3.9867414 | -2.7594621 |
| GPX2 | 1.33838172 | -2.4196147 | 2.84603873 | 0.00582412 | 0.99993162 | -3.9520821 | 2.75752276 |
| GATA6-AS1 | -2.3078486 | -5.7672725 | -2.8249387 | 0.00596829 | 0.99993162 | -3.9695012 | -2.7495187 |
| RN7SL217P | 2.14684887 | -5.7514952 | 2.82753554 | 0.0059786 | 0.99993162 | -3.928952 | 2.74895303 |
| ITPKB-IT1 | -2.1582488 | -6.0282751 | -2.8019267 | 0.00637334 | 0.99993162 | -3.9491384 | -2.7279286 |
| KRT18P40 | 2.28249733 | -5.5036918 | 2.79414092 | 0.00655364 | 0.99993162 | -3.9777307 | 2.71871336 |
| RPL13P8 | 0.50063323 | 0.61569762 | 2.78954362 | 0.00658611 | 0.99993162 | -3.9286642 | 2.71707786 |
| KPNA7 | 1.70399449 | -4.0926065 | 2.77779644 | 0.00682323 | 0.99993162 | -3.9276954 | 2.70535056 |
| LINC01659 | -2.3383035 | -5.4876467 | -2.7602345 | 0.0071312 | 0.99993162 | -4.0432844 | -2.6906546 |
| LINC01429 | 1.91642663 | -6.3972255 | 2.75706508 | 0.00716213 | 0.99993162 | -3.9317213 | 2.68921026 |
| ST3GAL1P1 | 2.21445618 | -4.5256427 | 2.75095103 | 0.00726294 | 0.99993162 | -3.9845894 | 2.68454124 |
| GPR146 | -1.5465118 | -1.989843 | -2.7513853 | 0.00732781 | 0.99993162 | -4.1148295 | -2.6815677 |
| BOLA2-SMG1P6 | 1.28537975 | -2.1122335 | 2.74705357 | 0.00740811 | 0.99993162 | -3.9548001 | 2.67791935 |
| LINC03002 | -2.2815759 | -5.9557117 | -2.7464163 | 0.00746327 | 0.99993162 | -4.0303377 | -2.6754335 |
| INO80D | 0.47268378 | 5.21372001 | 2.74147349 | 0.00751684 | 0.99993162 | -3.9873541 | 2.67303515 |
| SNORD13D | 2.08969854 | -5.3545378 | 2.72626971 | 0.0077065 | 0.99993162 | -3.9540942 | 2.66466527 |
| SPDYE6 | 1.01679049 | -0.4787018 | 2.73425824 | 0.00770865 | 0.99993162 | -4.0694353 | 2.66457129 |
| ALPP | 2.31539443 | -6.0064422 | 2.72339458 | 0.00792542 | 0.99993162 | -3.9496181 | 2.65523076 |
| TRIM60P13 | 1.10701675 | -6.5394726 | 2.72017465 | 0.00798077 | 0.99993162 | -3.9862608 | 2.65288212 |
| DIPK2B | -2.502225 | -4.4692064 | -2.7134893 | 0.0081188 | 0.99993162 | -4.0768272 | -2.6470888 |
| GPR34 | -2.0877477 | -2.9929216 | -2.7066192 | 0.008256 | 0.99993162 | -4.0677255 | -2.641417 |
| TMEM183AP3 | 2.27894326 | -4.5858943 | 2.70880491 | 0.00830675 | 0.99993162 | -4.0288679 | 2.63934049 |
| ADCY6-DT | -1.3530101 | -1.6079167 | -2.7054269 | 0.00830767 | 0.99993162 | -4.1124932 | -2.6393031 |
| NQO2-AS1 | -1.8132078 | -3.1310893 | -2.7020829 | 0.00839853 | 0.99993162 | -4.0953219 | -2.6356137 |
| MS4A2 | 1.65343472 | -6.605773 | 2.70005804 | 0.00842058 | 0.99993162 | -4.002679 | 2.6347235 |
| PPFIA1P1 | 2.09366593 | -4.6406091 | 2.6929649 | 0.00861596 | 0.99993162 | -3.9350103 | 2.62692786 |
| GOLGB1 | 0.26817254 | 7.69587605 | 2.69386991 | 0.00861788 | 0.99993162 | -4.0308472 | 2.62685196 |
| MRLN | -1.9420894 | -4.3307676 | -2.6863432 | 0.00885268 | 0.99993162 | -4.0639376 | -2.6176915 |
| PPP3CB-AS1 | -0.5690116 | 0.90886305 | -2.6827612 | 0.00888439 | 0.99993162 | -4.0657984 | -2.6164712 |
| RN7SL68P | 2.12620096 | -5.1034376 | 2.67850558 | 0.00889458 | 0.99993162 | -4.0344019 | 2.61607957 |
| MYL12BP1 | 1.20075045 | -2.0947197 | 2.67834537 | 0.00898568 | 0.99993162 | -3.992967 | 2.61259858 |
| UHRF2P1 | 2.35535618 | -4.0345766 | 2.67761328 | 0.00907911 | 0.99993162 | -4.0218564 | 2.60906075 |
| LINC02338 | 1.50205914 | -4.043712 | 2.6693447 | 0.00921732 | 0.99993162 | -4.0931212 | 2.60388685 |
| DANT1 | 2.28090827 | -4.8661279 | 2.66713789 | 0.00924543 | 0.99993162 | -3.960831 | 2.60284288 |
| NPC1L1 | 2.10548397 | -3.5400652 | 2.64910586 | 0.00969351 | 0.99993162 | -4.0885599 | 2.58657499 |
| GAPVD1 | 0.20183713 | 6.50636686 | 2.6508183 | 0.0097327 | 0.99993162 | -4.0444895 | 2.58518406 |
| H3C8 | 2.01380111 | -6.2173477 | 2.64972111 | 0.00977696 | 0.99993162 | -4.0076367 | 2.58361963 |
| LINC02747 | 2.22735706 | -6.3302983 | 2.64416016 | 0.00978655 | 0.99993162 | -4.0275945 | 2.58328137 |
| LTN1 | 0.22909245 | 5.97964391 | 2.6464424 | 0.00984982 | 0.99993162 | -4.0471326 | 2.58105738 |
| GPR176-DT | -2.0468285 | -3.5705506 | -2.6339707 | 0.00995553 | 0.99993162 | -4.0757394 | -2.5773701 |
| OXSM | -0.3096021 | 2.52670161 | -2.6356495 | 0.0101166 | 0.99993162 | -4.0777274 | -2.5718183 |
| DENND4A | 0.23665345 | 5.18279255 | 2.62575223 | 0.01034318 | 0.99993162 | -4.0466939 | 2.5641399 |
| ZNF318 | 0.24264886 | 6.801577 | 2.6220949 | 0.01048828 | 0.99993162 | -4.0603532 | 2.55930144 |
| TOMM20L-DT | 1.37531623 | -3.2423558 | 2.61655601 | 0.01054302 | 0.99993162 | -4.0528371 | 2.5574912 |
| LINC02141 | 2.4487754 | -5.748575 | 2.61123598 | 0.01070362 | 0.99993162 | -4.0436187 | 2.55222892 |
| TMED10P2 | -1.9623606 | -4.8742148 | -2.6101432 | 0.01078232 | 0.99993162 | -4.0665473 | -2.5496757 |
| RBBP6 | 0.18111941 | 6.89219435 | 2.6105533 | 0.01079341 | 0.99993162 | -4.0689108 | 2.54931708 |
| CDK13-DT | -0.5208503 | 0.66179279 | -2.5984359 | 0.01120516 | 0.99993162 | -4.0960666 | -2.5362349 |
| INTS6L-AS1 | -2.4284914 | -4.7458547 | -2.5895555 | 0.01121704 | 0.99993162 | -4.0966619 | -2.5358637 |
| FBXW11P1 | -1.1731184 | -1.6660475 | -2.5872488 | 0.01146447 | 0.99993162 | -4.1686525 | -2.5282132 |
| PRPF18 | 0.23413245 | 5.2875866 | 2.58091777 | 0.0116849 | 0.99993162 | -4.0752113 | 2.5215201 |
| GOT2P6 | -1.6195026 | -6.6539396 | -2.579238 | 0.01169242 | 0.99993162 | -4.0757819 | -2.5212939 |
| LINC02516 | -2.1853715 | -4.8326781 | -2.5723554 | 0.01187644 | 0.99993162 | -4.1331187 | -2.5157946 |
| FABP5 | -0.4653077 | 3.79011616 | -2.574635 | 0.01189448 | 0.99993162 | -4.1517303 | -2.5152595 |
| CXCL13 | 1.99158755 | -5.1775623 | 2.56572238 | 0.01217989 | 0.99993162 | -4.054599 | 2.50688927 |
| LINC02709 | 1.8162773 | -3.2282574 | 2.56212802 | 0.01221485 | 0.99993162 | -4.075458 | 2.50587574 |
| LINC00934 | -2.1221604 | -6.0213916 | -2.5598819 | 0.01235123 | 0.99993162 | -4.0936715 | -2.5019472 |
| PAPOLA-DT | -0.465431 | 0.02059232 | -2.5563422 | 0.01244973 | 0.99993162 | -4.090771 | -2.4991338 |
| RNA5SP60 | 1.87018353 | -4.894015 | 2.54442659 | 0.01285094 | 0.99993162 | -4.0647377 | 2.48787351 |
| RC3H1 | 0.37455684 | 5.74827023 | 2.54116903 | 0.0129527 | 0.99993162 | -4.0960442 | 2.48506726 |
| LCOR | 0.33451998 | 5.98979136 | 2.53694841 | 0.0131198 | 0.99993162 | -4.100144 | 2.48050085 |
| CLEC4A | 2.00853075 | -5.8034134 | 2.53487178 | 0.01318125 | 0.99993162 | -4.0945881 | 2.47883432 |
| GGT6 | 2.01954586 | -6.2366157 | 2.52693872 | 0.01326572 | 0.99993162 | -4.080327 | 2.47655488 |
| EMSY-DT | -0.4699879 | 1.88360653 | -2.5343637 | 0.01327876 | 0.99993162 | -4.147723 | -2.4762043 |
| RBM26 | 0.23025396 | 6.7778808 | 2.52849786 | 0.01336976 | 0.99993162 | -4.1010955 | 2.47376512 |
| FBXL20 | 0.32331526 | 6.02280602 | 2.52591355 | 0.01345782 | 0.99993162 | -4.1004224 | 2.47141853 |
| DNM1P41 | 1.9538167 | -4.3352102 | 2.5265544 | 0.01348009 | 0.99993162 | -4.1264837 | 2.47082717 |
| LINC02674 | -1.323434 | -5.850659 | -2.5234752 | 0.01357743 | 0.99993162 | -4.0997862 | -2.468253 |
| ZNF280B | 0.31398913 | 3.33906524 | 2.5180556 | 0.01380021 | 0.99993162 | -4.1092379 | 2.46242212 |
| PLVAP | -2.3073063 | -4.9679094 | -2.5152862 | 0.01387039 | 0.99993162 | -4.1239917 | -2.4606026 |
| MIR4441 | 2.00258798 | -5.619978 | 2.50890793 | 0.01391117 | 0.99993162 | -4.1165289 | 2.45954909 |
| RICTOR | 0.25601571 | 5.68808058 | 2.51185826 | 0.01399225 | 0.99993162 | -4.1077667 | 2.45746222 |
| LINC01722 | -1.9157697 | -5.635379 | -2.5021997 | 0.01435457 | 0.99993162 | -4.1151009 | -2.4482658 |
| P2RX1 | 2.00591149 | -5.8087512 | 2.49680651 | 0.01461547 | 0.99993162 | -4.1109362 | 2.44176969 |
| HMBOX1 | 0.41306454 | 4.46678965 | 2.4952871 | 0.0146615 | 0.99993162 | -4.1053782 | 2.44063419 |
| SNHG30 | -0.3453198 | 3.54460167 | -2.4986183 | 0.01469465 | 0.99993162 | -4.1266563 | -2.4398184 |
| RPL11P3 | 1.5467842 | -3.2442547 | 2.49670909 | 0.01483859 | 0.99993162 | -4.0282438 | 2.43629469 |
| IGLV1-50 | 2.08444712 | -4.5025046 | 2.48739605 | 0.01489876 | 0.99993162 | -4.1022005 | 2.43483077 |
| RBX1 | -0.2779131 | 5.43660078 | -2.4820397 | 0.01510799 | 0.99993162 | -4.140704 | -2.42978 |
| KHDC1L | 1.80075995 | -5.8428642 | 2.47595248 | 0.0151997 | 0.99993162 | -4.0810313 | 2.42758549 |
| MIR4297 | 1.64453346 | -4.2877303 | 2.47978113 | 0.01520979 | 0.99993162 | -4.0995305 | 2.42734486 |
| GDNF | 1.43224867 | -1.9527954 | 2.48081451 | 0.01522435 | 0.99993162 | -4.1184338 | 2.42699769 |
| COMMD4 | -0.3439846 | 5.34735828 | -2.4801214 | 0.01525712 | 0.99993162 | -4.1398708 | -2.4262177 |
| TBL1XR1 | 0.20920674 | 7.01741325 | 2.47823887 | 0.01527886 | 0.99993162 | -4.1286685 | 2.42570082 |
| ZNF431 | 0.27343489 | 5.67294002 | 2.47689607 | 0.01532991 | 0.99993162 | -4.1209974 | 2.4244898 |
| LINC01795 | -1.8394393 | -6.0421413 | -2.474257 | 0.01544661 | 0.99993162 | -4.1209315 | -2.4217349 |
| IGSF6 | 1.18621074 | -1.4923399 | 2.48467167 | 0.01546898 | 0.99993162 | -4.1041036 | 2.42120899 |
| ZMPSTE24-DT | -0.2378835 | 3.8535908 | -2.4731196 | 0.0155516 | 0.99993162 | -4.1352716 | -2.4192722 |
| FREY1 | 1.14592219 | -1.9611924 | 2.4683474 | 0.01570573 | 0.99993162 | -4.0936988 | 2.41568297 |
| ACTR1AP1 | 1.77153071 | -6.3149306 | 2.46579119 | 0.01574832 | 0.99993162 | -4.0759812 | 2.41469671 |
| DCUN1D3 | 0.26331294 | 3.69097993 | 2.46161987 | 0.01595311 | 0.99993162 | -4.1363173 | 2.40998647 |
| ANAPC11 | -0.3678501 | 5.63926471 | -2.4625055 | 0.01603702 | 0.99993162 | -4.1514174 | -2.4080719 |
| MARF1 | 0.1955233 | 6.92135529 | 2.45835289 | 0.01610787 | 0.99993162 | -4.1357664 | 2.40646227 |
| PCDHGB8P | -2.2287607 | -3.5669652 | -2.4600156 | 0.01612128 | 0.99993162 | -4.20579 | -2.4061583 |
| ERVH48-1 | 1.41748081 | -1.682628 | 2.45127336 | 0.01619986 | 0.99993162 | -4.1274826 | 2.40438134 |
| XIRP1 | 2.11421114 | -4.8055781 | 2.45312464 | 0.01629013 | 0.99993162 | -4.0914529 | 2.4023496 |
| CCT6P1 | 1.54124258 | -3.2714811 | 2.45515695 | 0.01632372 | 0.99993162 | -4.1366252 | 2.40159605 |
| UQCC6 | -0.2634682 | 4.14713275 | -2.4527523 | 0.01638636 | 0.99993162 | -4.1424754 | -2.4001945 |
| BMP2KL | 1.9084993 | -5.9390517 | 2.45003789 | 0.01644615 | 0.99993162 | -4.0740591 | 2.39886109 |
| HDHD3 | -0.2731088 | 3.87925496 | -2.4476256 | 0.01652005 | 0.99993162 | -4.1567863 | -2.397219 |
| PTOV1P1 | -1.7972027 | -2.7018763 | -2.4443128 | 0.01661252 | 0.99993162 | -4.203317 | -2.3951731 |
| JMJD1C-AS1 | 0.85595356 | -1.5391188 | 2.44574938 | 0.01664165 | 0.99993162 | -4.1265915 | 2.39453062 |
| OR6L2P | 1.75277529 | -3.6310157 | 2.44051373 | 0.01665324 | 0.99993162 | -4.1289838 | 2.39427545 |
| NOX1 | -0.912259 | -0.9692982 | -2.4424183 | 0.01669161 | 0.99993162 | -4.1802436 | -2.3934312 |
| EFCAB10-AS1 | 0.38830875 | 1.49749263 | 2.43424013 | 0.01711491 | 0.99993162 | -4.1306615 | 2.38422989 |
| LAMTOR3P2 | 2.02634721 | -4.8796433 | 2.42249773 | 0.01742976 | 0.99993162 | -4.1260654 | 2.37751447 |
| RASA1 | 0.20027182 | 6.17604531 | 2.42753951 | 0.01750198 | 0.99993162 | -4.1492931 | 2.37598912 |
| LINC02669 | -2.1354434 | -4.6752452 | -2.4281144 | 0.01752141 | 0.99993162 | -4.1474934 | -2.3755798 |
| CYP2C9 | 1.69428959 | -5.4250132 | 2.42392202 | 0.01758077 | 0.99993162 | -4.1422714 | 2.37433135 |
| LINC01284 | 0.90825188 | -1.5078183 | 2.42614404 | 0.01762686 | 0.99993162 | -4.14232 | 2.37336458 |
| ACAP2 | 0.23105079 | 5.96422421 | 2.42339358 | 0.01766294 | 0.99993162 | -4.1526058 | 2.37260914 |
| ZNF891 | 0.47740714 | 4.70351872 | 2.42240188 | 0.0176962 | 0.99993162 | -4.1352696 | 2.37191417 |
| C17orf114 | -1.2476305 | -1.1621615 | -2.4241066 | 0.01772191 | 0.99993162 | -4.2056905 | -2.3713778 |
| MTUS1-DT | -1.9732319 | -5.2577195 | -2.4177305 | 0.01782313 | 0.99993162 | -4.1303876 | -2.3692723 |
| POLR2G | -0.2974642 | 5.82303698 | -2.4191517 | 0.01789946 | 0.99993162 | -4.167072 | -2.3676913 |
| TAF9P3 | 1.23893235 | -2.9345824 | 2.41048841 | 0.01797699 | 0.99993162 | -4.1471166 | 2.36609176 |
| COQ7-DT | 1.6383111 | -4.5787554 | 2.4089389 | 0.01821163 | 0.99993162 | -4.1371954 | 2.36128704 |
| LINC01762 | -1.988552 | -5.8589923 | -2.4078755 | 0.01827364 | 0.99993162 | -4.1531899 | -2.3600263 |
| MCIDAS | -1.3750484 | -5.9945075 | -2.4065595 | 0.01836567 | 0.99993162 | -4.1227412 | -2.3581622 |
| SHBG | -0.6334671 | 0.17149125 | -2.4079745 | 0.01838394 | 0.99993162 | -4.2142301 | -2.3577931 |
| HELLPAR | 0.64730746 | -0.1988157 | 2.40825531 | 0.0184338 | 0.99993162 | -4.1135536 | 2.35678741 |
| VCPIP1 | 0.3178661 | 5.19811955 | 2.4042662 | 0.01844985 | 0.99993162 | -4.1524396 | 2.35646413 |
| TRAF6 | 0.22209032 | 5.28643601 | 2.40483928 | 0.01853279 | 0.99993162 | -4.1586379 | 2.35479768 |
| NDUFV2P1 | -0.5035581 | 0.77459283 | -2.4035269 | 0.01857724 | 0.99993162 | -4.1787699 | -2.3539074 |
| ASXL3-DT | 1.79940235 | -5.1654218 | 2.40290432 | 0.01858819 | 0.99993162 | -4.1541973 | 2.35368828 |
| ACER2 | -0.3317458 | 2.1501686 | -2.4033747 | 0.01860158 | 0.99993162 | -4.1773429 | -2.3534205 |
| MTRF1LP1 | 1.82841999 | -3.3370512 | 2.39583294 | 0.01870703 | 0.99993162 | -4.161206 | 2.35131825 |
| LINC00671 | -1.778621 | -5.8908377 | -2.3979433 | 0.01874769 | 0.99993162 | -4.1397378 | -2.3505102 |
| RNU6-130P | 1.59360647 | -3.5410097 | 2.39619784 | 0.01880084 | 0.99993162 | -4.1415756 | 2.34945661 |
| GTF2A2 | -0.2450497 | 5.17466855 | -2.4005352 | 0.01881291 | 0.99993162 | -4.1718834 | -2.3492176 |
| RPL9P18 | -2.2960717 | -4.4623453 | -2.394683 | 0.01892069 | 0.99993162 | -4.1741594 | -2.3470899 |
| FTOP1 | -1.103192 | -2.0466271 | -2.3972858 | 0.01909113 | 0.99993162 | -4.1930458 | -2.3437467 |
| LINC01979 | 0.73866046 | -0.659015 | 2.39209564 | 0.01917437 | 0.99993162 | -4.1523239 | 2.3421234 |
| TRIM33 | 0.21027849 | 7.08882279 | 2.38756016 | 0.01936152 | 0.99993162 | -4.1674927 | 2.33849624 |
| CEP85L | 0.27097664 | 4.45064226 | 2.38482868 | 0.01953212 | 0.99993162 | -4.1594409 | 2.33521644 |
| ZNF451 | 0.21540913 | 5.72282969 | 2.38379369 | 0.01953586 | 0.99993162 | -4.166439 | 2.33514472 |
| ARHGEF7-AS1 | 1.34370539 | -3.2804453 | 2.38289382 | 0.01963984 | 0.99993162 | -4.1673894 | 2.33315834 |
| MIR628 | 1.14804637 | -2.815812 | 2.37893469 | 0.01974427 | 0.99993162 | -4.1481956 | 2.33117233 |
| PRKD3-DT | -1.8966999 | -5.4311707 | -2.3771629 | 0.01979513 | 0.99993162 | -4.1528853 | -2.3302086 |
| BRWD3 | 0.30944989 | 5.59195021 | 2.37603219 | 0.01989753 | 0.99993162 | -4.1619266 | 2.32827464 |
| CEP295NL | 1.77369783 | -3.6719372 | 2.37447048 | 0.02008304 | 0.99993162 | -4.1791462 | 2.32479292 |
| GJA3 | -2.771968 | -3.880047 | -2.3713044 | 0.02012035 | 0.99993162 | -4.2195073 | -2.324096 |
| OR7E100P | 1.80306237 | -5.8759148 | 2.36793667 | 0.02021829 | 0.99993162 | -4.1461857 | 2.32227204 |
| TMEM258 | -0.4259635 | 4.22386403 | -2.3659665 | 0.02035042 | 0.99993162 | -4.2006059 | -2.3198237 |
| NUDT8 | -0.5242307 | 1.49949893 | -2.3663822 | 0.02039447 | 0.99993162 | -4.2060212 | -2.3190105 |
| COX14 | -0.3909021 | 3.12720318 | -2.3642879 | 0.02049473 | 0.99993162 | -4.1986767 | -2.3171653 |
| RPA3 | -0.2624904 | 3.32175527 | -2.3647102 | 0.02051158 | 0.99993162 | -4.2075935 | -2.316856 |
| CERS6-AS1 | 1.74811313 | -5.3748074 | 2.35943875 | 0.02065673 | 0.99993162 | -4.2076587 | 2.31420038 |
| MON2 | 0.18217187 | 6.51422739 | 2.3581373 | 0.02080557 | 0.99993162 | -4.1752627 | 2.31149432 |
| ZNF221 | 0.39503867 | 3.14488085 | 2.35063182 | 0.02131626 | 0.99993162 | -4.1601989 | 2.30233544 |
| IKZF5 | 0.20865283 | 4.64399332 | 2.34396991 | 0.02149973 | 0.99993162 | -4.1811474 | 2.2990917 |
| CMC1 | -0.318484 | 2.87834366 | -2.3446338 | 0.02157489 | 0.99993162 | -4.2117077 | -2.2977699 |
| CDRT15 | 1.38576251 | -2.9523009 | 2.34209762 | 0.02164471 | 0.99993162 | -4.1811713 | 2.29654551 |
| LINC01374 | -2.0318267 | -5.3398542 | -2.3408891 | 0.02168748 | 0.99993162 | -4.2366764 | -2.2957971 |
| PRG4 | -1.3082243 | -2.3902667 | -2.3411508 | 0.02169726 | 0.99993162 | -4.2352098 | -2.2956262 |
| ALG1L14P | -1.8117086 | -5.5988075 | -2.3397034 | 0.02173319 | 0.99993162 | -4.2042928 | -2.2949988 |
| COMMD6 | -0.2810134 | 4.91623866 | -2.3375394 | 0.02187379 | 0.99993162 | -4.211713 | -2.2925524 |
| RNU6-484P | 1.77581352 | -5.2782071 | 2.3360772 | 0.02189734 | 0.99993162 | -4.1433002 | 2.29214396 |
| ALOX15P2 | 1.70062585 | -6.0370435 | 2.33423894 | 0.02204275 | 0.99993162 | -4.1725488 | 2.28963038 |
| MIR4422HG | -1.928385 | -5.8270087 | -2.3291866 | 0.02233978 | 0.99993162 | -4.2096325 | -2.2845405 |
| DDX50P2 | -2.2011531 | -5.5026326 | -2.3275866 | 0.02240897 | 0.99993162 | -4.1795475 | -2.2833634 |
| RNU6-37P | 1.66700121 | -3.7149846 | 2.32275594 | 0.0224605 | 0.99993162 | -4.1759372 | 2.28248871 |
| CPA2 | -1.4856263 | -2.5636438 | -2.3305593 | 0.02250196 | 0.99993162 | -4.2232406 | -2.2817864 |
| PDK4-AS1 | -1.3868314 | -2.424322 | -2.3268189 | 0.02255083 | 0.99993162 | -4.2417499 | -2.2809597 |
| CCDC196 | 1.43994566 | -2.7715639 | 2.32182902 | 0.02268794 | 0.99993162 | -4.1919792 | 2.27864906 |
| C1orf53 | -0.819469 | 0.01650279 | -2.3241665 | 0.02269984 | 0.99993162 | -4.2446992 | -2.2784489 |
| RPS4XP4 | 1.93183255 | -5.5261305 | 2.31753619 | 0.02275729 | 0.99993162 | -4.1582138 | 2.27748477 |
| OR11H4 | 1.81353901 | -6.0369411 | 2.32075729 | 0.02278266 | 0.99993162 | -4.1812288 | 2.27705977 |
| ZNF652 | 0.33283174 | 5.4573723 | 2.31974653 | 0.02279857 | 0.99993162 | -4.1842106 | 2.27679331 |
| KRT18P4 | 2.06186927 | -3.5990288 | 2.31583896 | 0.02285285 | 0.99993162 | -4.1888917 | 2.27588578 |
| LINC01193 | 1.64301624 | -5.5778713 | 2.31239881 | 0.02339545 | 0.99993162 | -4.1838915 | 2.26691447 |
| NPPB | -2.0167772 | -4.3656384 | -2.3105705 | 0.02348757 | 0.99993162 | -4.2475334 | -2.2654094 |
| KDM7A | 0.29360736 | 4.5445173 | 2.30836782 | 0.02355225 | 0.99993162 | -4.1776928 | 2.26435567 |
| LINC00536 | -2.088547 | -6.0253238 | -2.3041448 | 0.02355926 | 0.99993162 | -4.2070362 | -2.2642416 |
| COX6A1P2 | -0.7564322 | -1.0407707 | -2.3065854 | 0.02358486 | 0.99993162 | -4.213037 | -2.2638253 |
| CYYR1-AS1 | 1.24485782 | -2.7265913 | 2.30717173 | 0.02360762 | 0.99993162 | -4.2131289 | 2.26345563 |
| NECTIN4 | -2.1629476 | -4.135406 | -2.3028413 | 0.02363003 | 0.99993162 | -4.2377386 | -2.2630918 |
| C4orf36 | -1.7142949 | -3.4246706 | -2.3055495 | 0.02364143 | 0.99993162 | -4.2302965 | -2.2629068 |
| TMX2P1 | 0.23766832 | 2.67786425 | 2.30570062 | 0.02369124 | 0.99993162 | -4.1805919 | 2.26209978 |
| ASAP1-IT2 | 1.81599393 | -3.8378135 | 2.30869928 | 0.0237709 | 0.99993162 | -4.2015675 | 2.26081196 |
| NHLRC2 | 0.30754917 | 5.77048144 | 2.30269856 | 0.02392198 | 0.99993162 | -4.2007888 | 2.25838003 |
| ELOB | -0.270223 | 6.99510076 | -2.2967518 | 0.02416211 | 0.99993162 | -4.2086867 | -2.2545417 |
| TOB2P1 | -1.1480402 | -1.9174064 | -2.2931153 | 0.02441074 | 0.99993162 | -4.2788871 | -2.2506023 |
| RN7SKP275 | 1.2998653 | -2.3143187 | 2.29035843 | 0.02465406 | 0.99993162 | -4.1630993 | 2.24678045 |
| MMP10 | -2.0538664 | -3.0430633 | -2.2896895 | 0.02478615 | 0.99993162 | -4.3260795 | -2.2447194 |
| ARGFXP2 | 1.93080632 | -3.9417575 | 2.2852558 | 0.02479471 | 0.99993162 | -4.1439494 | 2.24458613 |
| RCAN3AS | 1.87284615 | -5.039757 | 2.28474712 | 0.0248655 | 0.99993162 | -4.2003114 | 2.24348584 |
| PSMD6-AS2 | 0.5882211 | 0.2350725 | 2.28864027 | 0.02487565 | 0.99993162 | -4.1687724 | 2.24332828 |
| ZZEF1 | 0.16493665 | 7.31099883 | 2.28491876 | 0.02492156 | 0.99993162 | -4.2079452 | 2.2426164 |
| JMJD1C | 0.23251076 | 6.90028186 | 2.28549428 | 0.02492271 | 0.99993162 | -4.2088329 | 2.24259857 |
| ARL16 | -0.1538984 | 5.15709337 | -2.2833767 | 0.02502066 | 0.99993162 | -4.2173799 | -2.2410836 |
| LAMTOR4 | -0.2896585 | 5.21860871 | -2.2844528 | 0.02508676 | 0.99993162 | -4.2238829 | -2.2400642 |
| LRP1-AS | 2.10055271 | -5.1573413 | 2.27824413 | 0.02509295 | 0.99993162 | -4.1889664 | 2.23996877 |
| BICDL3P | 1.61411809 | -3.4309531 | 2.28315587 | 0.02511229 | 0.99993162 | -4.1667956 | 2.23967103 |
| TMC5 | 1.08240218 | -2.4913961 | 2.2804084 | 0.02518674 | 0.99993162 | -4.195058 | 2.23852654 |
| DDX59-AS1 | -1.5589922 | -3.2024564 | -2.2828103 | 0.02525383 | 0.99993162 | -4.2643315 | -2.2374976 |
| SOX11 | 0.37037064 | 9.04043593 | 2.27623685 | 0.02552019 | 0.99993162 | -4.215423 | 2.23343632 |
| DHFRP1 | -1.1535754 | -3.0801332 | -2.2741573 | 0.02565643 | 0.99993162 | -4.2410754 | -2.231373 |
| SNORD100 | -1.314535 | -2.8743901 | -2.2726214 | 0.02567298 | 0.99993162 | -4.2518244 | -2.231123 |
| SUMO2P13 | -1.8365675 | -6.2285605 | -2.2684878 | 0.02570594 | 0.99993162 | -4.2252202 | -2.2306256 |
| NODAL | 0.84176158 | -0.436475 | 2.27040012 | 0.02586169 | 0.99993162 | -4.2142886 | 2.22828231 |
| RNA5SP370 | 1.63225755 | -5.6816817 | 2.26506754 | 0.02597121 | 0.99993162 | -4.1916848 | 2.22664197 |
| RN7SL481P | 1.16894795 | -1.4619489 | 2.26600559 | 0.02608554 | 0.99993162 | -4.2502054 | 2.22493599 |
| PMS2P7 | -0.935452 | -2.1693558 | -2.2660571 | 0.02608662 | 0.99993162 | -4.236315 | -2.2249199 |
| DNAJB8 | 1.64931148 | -5.6208716 | 2.26655301 | 0.02610412 | 0.99993162 | -4.1298535 | 2.22465924 |
| TEKT4P2 | -1.5373859 | -6.0758041 | -2.2654963 | 0.02611715 | 0.99993162 | -4.2406535 | -2.2244654 |
| MINDY2-DT | -0.6948741 | -0.4196592 | -2.264421 | 0.02621642 | 0.99993162 | -4.2845097 | -2.2229907 |
| TEDC2-AS1 | 1.04995964 | -0.9717114 | 2.26111043 | 0.02622883 | 0.99993162 | -4.22417 | 2.22280683 |
| ZNF264 | 0.32824903 | 5.84997857 | 2.26219888 | 0.0262438 | 0.99993162 | -4.2159013 | 2.22258493 |
| SLC6A1-AS1 | 0.84510753 | -0.1997903 | 2.262583 | 0.02639913 | 0.99993162 | -4.1693331 | 2.2202894 |
| IER5L-AS1 | 1.67477492 | -5.7883256 | 2.25764633 | 0.02656179 | 0.99993162 | -4.1630508 | 2.21789797 |
| COX7C | -0.3747926 | 7.00310012 | -2.2525228 | 0.02698976 | 0.99993162 | -4.2286823 | -2.2116659 |
| H3C12 | -1.9936424 | -5.7225412 | -2.2509796 | 0.02707453 | 0.99993162 | -4.221985 | -2.2104416 |
| PHC3 | 0.28505269 | 6.46564681 | 2.24804914 | 0.0273405 | 0.99993162 | -4.2219189 | 2.20662167 |
| ROBO4 | 0.99003965 | -1.4667937 | 2.24607713 | 0.02738192 | 0.99993162 | -4.1955721 | 2.20602972 |
| TPI1P2 | -0.5703321 | 0.31345001 | -2.2453558 | 0.02750257 | 0.99993162 | -4.2929157 | -2.2043097 |
| ABCG8 | -1.4509778 | -3.2091372 | -2.2415616 | 0.0277793 | 0.99993162 | -4.2225763 | -2.2003891 |
| PLGRKT | -0.3048337 | 3.76078449 | -2.2413852 | 0.0279651 | 0.99993162 | -4.2436647 | -2.1977757 |
| SPDEF | -0.6081902 | -0.1114341 | -2.2369665 | 0.02803658 | 0.99993162 | -4.2192622 | -2.1967742 |
| CNN2P1 | 1.50533734 | -3.2794304 | 2.22501794 | 0.02861809 | 0.99993162 | -4.2070047 | 2.18870779 |
| FHIP2A | 0.17152648 | 5.87748165 | 2.22944259 | 0.02862975 | 0.99993162 | -4.2284275 | 2.18854743 |
| MIR4527HG | -1.7290735 | -5.468121 | -2.2269754 | 0.0288128 | 0.99993162 | -4.242737 | -2.1860383 |
| FMR1NB | 1.64872859 | -6.053148 | 2.22240713 | 0.02881594 | 0.99993162 | -4.1884416 | 2.18599548 |
| IGLVIVOR22-2 | 1.91273591 | -5.2860641 | 2.22669428 | 0.02889194 | 0.99993162 | -4.2050404 | 2.18495787 |
| C12orf60 | -0.448222 | 0.75965344 | -2.2246303 | 0.02895861 | 0.99993162 | -4.2704348 | -2.1840496 |
| ATG2B | 0.24904311 | 6.23216907 | 2.21908417 | 0.02923249 | 0.99993162 | -4.2327198 | 2.18033704 |
| AKR7A2P2 | 1.8532896 | -6.2165418 | 2.21918373 | 0.02923567 | 0.99993162 | -4.2055959 | 2.18029412 |
| USE1 | -0.1956021 | 4.21975207 | -2.2193158 | 0.02926392 | 0.99993162 | -4.2362462 | -2.1799129 |
| TUT7 | 0.17577884 | 4.97667538 | 2.2173561 | 0.02946343 | 0.99993162 | -4.2275487 | 2.1772297 |
| SLX4 | 0.20318306 | 5.5152009 | 2.21361026 | 0.02972637 | 0.99993162 | -4.2332057 | 2.17371732 |
| ZNHIT1 | -0.1641983 | 6.30580328 | -2.2122043 | 0.02987516 | 0.99993162 | -4.2450522 | -2.1717416 |
| CD28 | -2.2018071 | -5.4600929 | -2.2019392 | 0.03024091 | 0.99993162 | -4.2620469 | -2.1669206 |
| BDP1 | 0.26414232 | 6.45999907 | 2.2046973 | 0.03034474 | 0.99993162 | -4.23974 | 2.16556108 |
| DYNLL1 | -0.2381528 | 6.96602355 | -2.2045647 | 0.0303959 | 0.99993162 | -4.2471174 | -2.1648928 |
| COX7CP1 | -1.9551483 | -4.0247227 | -2.2022598 | 0.03045165 | 0.99993162 | -4.2543607 | -2.1641655 |
| CLDN11 | -1.04715 | -1.1833209 | -2.2009488 | 0.03061385 | 0.99993162 | -4.316909 | -2.1620562 |
| EIF2S2P3 | -1.8742515 | -4.0360329 | -2.1977994 | 0.03068691 | 0.99993162 | -4.2888939 | -2.1611092 |
| ZNF638 | 0.21697828 | 7.43667844 | 2.20064623 | 0.03070074 | 0.99993162 | -4.2411118 | 2.16093005 |
| CSTA | 1.6879006 | -6.0556931 | 2.19881251 | 0.03070087 | 0.99993162 | -4.2475971 | 2.16092837 |
| LINC00315 | 1.83772535 | -4.5120043 | 2.19864102 | 0.03089162 | 0.99993162 | -4.2580217 | 2.15846599 |
| MLYCD | 0.32725769 | 3.90995426 | 2.19380691 | 0.03089821 | 0.99993162 | -4.2255312 | 2.15838115 |
| RPL13P4 | -1.9314608 | -5.8063447 | -2.1955636 | 0.03093872 | 0.99993162 | -4.2650635 | -2.1578599 |
| ZNF780B | 0.29149712 | 4.98777488 | 2.19208407 | 0.03117054 | 0.99993162 | -4.2416597 | 2.15488872 |
| LGALS4 | 0.90330465 | -0.8183431 | 2.18939567 | 0.03122722 | 0.99993162 | -4.2094325 | 2.15416523 |
| CHD9 | 0.2491197 | 7.13582151 | 2.19182199 | 0.03131618 | 0.99993162 | -4.247259 | 2.15303175 |
| LINC02066 | 1.62687755 | -5.8366135 | 2.19133844 | 0.03131746 | 0.99993162 | -4.2324293 | 2.15301548 |
| MRPS21 | -0.2066118 | 6.11057328 | -2.1910699 | 0.03133072 | 0.99993162 | -4.248602 | -2.1528468 |
| NFKBIE | -0.267621 | 3.64284021 | -2.1904855 | 0.03137984 | 0.99993162 | -4.253615 | -2.1522224 |
| PIGCP1 | 0.3089101 | 2.04705564 | 2.18901934 | 0.03153426 | 0.99993162 | -4.2415329 | 2.15026494 |
| UBA5 | 0.1470622 | 6.55346629 | 2.18785135 | 0.03155537 | 0.99993162 | -4.2474734 | 2.14999799 |
| HSPB3 | -1.6632717 | -5.5673327 | -2.1881385 | 0.03156137 | 0.99993162 | -4.2748748 | -2.1499222 |
| HCRT | -1.9580584 | -4.773829 | -2.1817099 | 0.03197966 | 0.99993162 | -4.2483764 | -2.1446647 |
| LCT | 1.82487261 | -4.1734948 | 2.18050503 | 0.03206384 | 0.99993162 | -4.2433629 | 2.14361391 |
| ASIC5 | -1.6929952 | -4.9957822 | -2.1783889 | 0.0323274 | 0.99993162 | -4.2622134 | -2.1403388 |
| PDIA3P1 | 0.29941739 | 2.47195033 | 2.1772094 | 0.0324398 | 0.99993162 | -4.2640973 | 2.138949 |
| FUT5 | -1.5708704 | -4.7540806 | -2.1761699 | 0.03250564 | 0.99993162 | -4.2502693 | -2.1381369 |
| RNU6-444P | 1.69456391 | -5.5184858 | 2.17242041 | 0.0325117 | 0.99993162 | -4.2664335 | 2.13806216 |
| LINC02259 | -1.8502228 | -4.6550723 | -2.1714962 | 0.03276668 | 0.99993162 | -4.2793506 | -2.1349305 |
| PSPHP1 | -0.5483453 | -4.2639955 | -2.1706247 | 0.03294199 | 0.99993162 | -4.2607537 | -2.1327896 |
| PARGP1 | 0.48726443 | 0.68837082 | 2.16928167 | 0.03304867 | 0.99993162 | -4.2565114 | 2.1314915 |
| ENPP7P6 | 1.54296113 | -5.1184302 | 2.16584831 | 0.0331922 | 0.99993162 | -4.2312591 | 2.12975073 |
| RNU6-8 | -2.0743874 | -4.6830367 | -2.1639467 | 0.03328583 | 0.99993162 | -4.2396247 | -2.1286185 |
| CASS4 | -1.251199 | -2.4212403 | -2.1642863 | 0.03361233 | 0.99993162 | -4.2928791 | -2.1246919 |
| STK4-DT | -1.734027 | -3.2822414 | -2.1624205 | 0.03373849 | 0.99993162 | -4.3262688 | -2.1231834 |
| RPL21P125 | 0.8959835 | -1.3129408 | 2.15947016 | 0.03378557 | 0.99993162 | -4.2709436 | 2.1226216 |
| DICER1 | 0.24158704 | 7.12046131 | 2.16057665 | 0.03383227 | 0.99993162 | -4.2597939 | 2.12206507 |
| LINC02755 | -1.6776615 | -5.3229141 | -2.1578751 | 0.03392272 | 0.99993162 | -4.2488581 | -2.120989 |
| PRDX3 | -0.2066555 | 6.58303781 | -2.1573198 | 0.03399667 | 0.99993162 | -4.266883 | -2.1201112 |
| ILF3-DT | -0.2287224 | 5.00420472 | -2.1575079 | 0.03401193 | 0.99993162 | -4.2707451 | -2.1199302 |
| KRT8P33 | 0.80419635 | -0.1068255 | 2.15525896 | 0.03420991 | 0.99993162 | -4.2349871 | 2.11758877 |
| FAM153A | 1.05194958 | -1.866127 | 2.15429282 | 0.03427217 | 0.99993162 | -4.2804929 | 2.11685487 |
| LARP1P1 | 1.74787664 | -5.9628921 | 2.15252824 | 0.0342809 | 0.99993162 | -4.2624126 | 2.11675197 |
| TRIAP1 | -0.3314541 | 4.01094961 | -2.1528067 | 0.03442864 | 0.99993162 | -4.2852109 | -2.1150153 |
| XRN1 | 0.20613886 | 5.98560564 | 2.15033897 | 0.03453062 | 0.99993162 | -4.2573458 | 2.11382027 |
| FAM106C | -1.6501223 | -6.0290219 | -2.1489765 | 0.03456434 | 0.99993162 | -4.2918676 | -2.1134258 |
| PRF1 | -1.592569 | -6.5162529 | -2.1454301 | 0.03491749 | 0.99993162 | -4.2561153 | -2.109314 |
| RPS3P6 | 2.07054393 | -4.7157285 | 2.14405229 | 0.03493488 | 0.99993162 | -4.2679449 | 2.10911238 |
| LINC01168 | -1.9018563 | -5.932647 | -2.1426433 | 0.03507436 | 0.99993162 | -4.269507 | -2.1074989 |
| SPEN-AS1 | -0.3423515 | 3.35531891 | -2.1433459 | 0.03516355 | 0.99993162 | -4.283075 | -2.10647 |
| HSCB | -0.2714185 | 2.37035594 | -2.1425057 | 0.03523579 | 0.99993162 | -4.2945279 | -2.1056382 |
| MDN1 | 0.35503943 | 6.66183374 | 2.13833494 | 0.03555782 | 0.99993162 | -4.2633237 | 2.10194814 |
| LST1 | -1.5250381 | -3.1858922 | -2.1376821 | 0.03571646 | 0.99993162 | -4.2903665 | -2.1001408 |
| ZNF197 | 0.20593689 | 5.86182644 | 2.13591043 | 0.0357714 | 0.99993162 | -4.2692239 | 2.09951641 |
| CEP97 | 0.27211879 | 5.93376978 | 2.1332727 | 0.03591526 | 0.99993162 | -4.2669887 | 2.09788555 |
| NIP7P3 | -1.463144 | -6.6405999 | -2.1311711 | 0.03605528 | 0.99993162 | -4.2685795 | -2.0963035 |
| RNU5D-1 | -1.712089 | -6.1013404 | -2.1288312 | 0.03605743 | 0.99993162 | -4.2651144 | -2.0962792 |
| H2BC17 | -1.9699373 | -5.2314824 | -2.1250917 | 0.03633521 | 0.99993162 | -4.2680756 | -2.0931563 |
| SNRPF | -0.2834752 | 5.08645913 | -2.1285972 | 0.03635124 | 0.99993162 | -4.2802758 | -2.0929767 |
| SDHAF3 | -0.3173928 | 3.12108573 | -2.1275363 | 0.03648038 | 0.99993162 | -4.2955021 | -2.0915323 |
| RNU6ATAC16P | 1.27299991 | -3.963212 | 2.12673123 | 0.03648456 | 0.99993162 | -4.2807982 | 2.09148559 |
| SLC25A34-AS1 | 1.50473939 | -6.294671 | 2.12583957 | 0.03648807 | 0.99993162 | -4.2449929 | 2.09144636 |
| SMIM30 | -0.2784077 | 4.44222967 | -2.1273337 | 0.03648859 | 0.99993162 | -4.3098532 | -2.0914405 |
| LINC00964 | -1.2864326 | -2.3363285 | -2.1271182 | 0.03651807 | 0.99993162 | -4.3040714 | -2.0911115 |
| EPX | 0.44095867 | 1.14345701 | 2.12601031 | 0.03652357 | 0.99993162 | -4.269258 | 2.09105016 |
| UBL5 | -0.3883882 | 5.32625606 | -2.1228264 | 0.03657692 | 0.99993162 | -4.282842 | -2.0904553 |
| ITGB2-AS1 | 1.30650679 | -3.1193037 | 2.12536018 | 0.03659792 | 0.99993162 | -4.2571137 | 2.0902213 |
| LINC02983 | -0.4058082 | 1.3619077 | -2.1212464 | 0.03703174 | 0.99993162 | -4.2879416 | -2.085414 |
| NUBP1 | -0.2856447 | 2.59414554 | -2.1225401 | 0.03703767 | 0.99993162 | -4.2768138 | -2.0853485 |
| FABP5P1 | -2.028244 | -3.8988373 | -2.1200446 | 0.03710163 | 0.99993162 | -4.3367047 | -2.0846439 |
| PCBD1 | -0.3486843 | 3.70864937 | -2.1195558 | 0.03716873 | 0.99993162 | -4.30741 | -2.0839058 |
| HCCS-DT | -0.7673358 | -1.590536 | -2.1183627 | 0.03718651 | 0.99993162 | -4.287074 | -2.0837104 |
| POMP | -0.2588336 | 5.83276475 | -2.1170511 | 0.03726662 | 0.99993162 | -4.2831154 | -2.0828312 |
| SIAH2-AS1 | 1.19201547 | -2.8704388 | 2.11904976 | 0.03727591 | 0.99993162 | -4.2353524 | 2.08272929 |
| PPP1R36 | -0.9830455 | -1.564659 | -2.1176499 | 0.03732121 | 0.99993162 | -4.3079694 | -2.0822328 |
| SCARNA5 | 1.89903956 | -5.6562477 | 2.1134013 | 0.03737187 | 0.99993162 | -4.2696096 | 2.0816782 |
| SPDYE1 | 0.7830549 | -0.7113297 | 2.11762659 | 0.03737246 | 0.99993162 | -4.2878095 | 2.08167176 |
| EVPL | 1.68619024 | -3.9306543 | 2.11518226 | 0.03748292 | 0.99993162 | -4.2550046 | 2.08046483 |
| DNAH17 | 0.3550289 | 2.30000089 | 2.11497283 | 0.03751787 | 0.99993162 | -4.2563852 | 2.08008352 |
| LMAN1L | 1.71660252 | -5.2845784 | 2.11479301 | 0.03753976 | 0.99993162 | -4.288674 | 2.07984491 |
| RPL21P134 | -2.103344 | -4.8640587 | -2.1112408 | 0.0375916 | 0.99993162 | -4.2922615 | -2.0792803 |
| LINC00968 | -1.2952278 | -4.942695 | -2.1140356 | 0.03760179 | 0.99993162 | -4.2897101 | -2.0791693 |
| RNU6-48P | 1.49600098 | -3.8407233 | 2.11162342 | 0.03767834 | 0.99993162 | -4.2918449 | 2.07833695 |
| LAMA5-AS1 | -1.5358849 | -3.5311966 | -2.1135283 | 0.0377076 | 0.99993162 | -4.3110659 | -2.0780191 |
| AWAT1 | 1.61963432 | -5.8592839 | 2.11195033 | 0.03775657 | 0.99993162 | -4.2664136 | 2.07748766 |
| RPS4XP22 | 1.29887794 | -2.689478 | 2.11200094 | 0.03780078 | 0.99993162 | -4.2757426 | 2.07700844 |
| RASGRP4 | 1.06498404 | -1.8813854 | 2.11230658 | 0.03780763 | 0.99993162 | -4.2659544 | 2.07693424 |
| RNA5SP393 | 1.33647258 | -4.6050373 | 2.11025157 | 0.03786365 | 0.99993162 | -4.256585 | 2.07632777 |
| PPFIA2-AS1 | 0.56565562 | 0.22088636 | 2.11041865 | 0.03799286 | 0.99993162 | -4.2488098 | 2.07493172 |
| CBX3 | -0.1738372 | 7.6442381 | -2.1087359 | 0.03805491 | 0.99993162 | -4.2801415 | -2.0742628 |
| PCDHGA8 | 0.78495466 | -0.8873681 | 2.10938924 | 0.03806722 | 0.99993162 | -4.271043 | 2.0741302 |
| RPS6KA2-IT1 | 0.77982077 | -1.467594 | 2.10796114 | 0.03820355 | 0.99993162 | -4.2481249 | 2.07266412 |
| GALNT6 | -1.4645049 | -2.3679224 | -2.1061027 | 0.03834645 | 0.99993162 | -4.3397048 | -2.0711321 |
| PKP4-AS1 | 1.10843814 | -1.5952759 | 2.10408486 | 0.03840961 | 0.99993162 | -4.2757199 | 2.07045655 |
| MBD3L3 | -1.3423333 | -3.7395876 | -2.1050957 | 0.03854709 | 0.99993162 | -4.2761636 | -2.0689893 |
| UBBP1 | -1.9007301 | -5.0364777 | -2.1007422 | 0.03866468 | 0.99993162 | -4.3081689 | -2.0677378 |
| BANK1 | -1.8095111 | -3.6387172 | -2.1011293 | 0.03874111 | 0.99993162 | -4.3143286 | -2.0669262 |
| NAP1L6P | 1.48112799 | -4.6100343 | 2.10171812 | 0.03875508 | 0.99993162 | -4.2869317 | 2.06677799 |
| PIWIL4 | -0.4973364 | -0.5100442 | -2.0985675 | 0.03903283 | 0.99993162 | -4.272219 | -2.0638406 |
| ILDR1 | -1.7258354 | -5.3658257 | -2.0968637 | 0.03911555 | 0.99993162 | -4.3024714 | -2.0629692 |
| LDHAL6A | 0.60377422 | -0.5587306 | 2.09701495 | 0.03918218 | 0.99993162 | -4.266994 | 2.06226851 |
| ADORA2A-AS1 | 0.78813875 | -0.1647313 | 2.09609645 | 0.03919787 | 0.99993162 | -4.2546801 | 2.06210363 |
| GACAT3 | -1.6288733 | -3.2953814 | -2.0956654 | 0.03926947 | 0.99993162 | -4.2913661 | -2.061352 |
| CCDC186 | 0.24781485 | 5.52778155 | 2.09542096 | 0.0393574 | 0.99993162 | -4.2776956 | 2.06043057 |
| MTND4P20 | 1.27991121 | -6.5579569 | 2.09359017 | 0.03937375 | 0.99993162 | -4.266401 | 2.06025935 |
| MTND1P23 | 1.09985011 | 2.1666166 | 2.09419106 | 0.03940323 | 0.99993162 | -4.295184 | 2.05995092 |
| FAM207BP | -1.973772 | -5.5448379 | -2.0932019 | 0.03944656 | 0.99993162 | -4.305991 | -2.0594979 |
| OXLD1 | -0.2749352 | 4.27816335 | -2.0942514 | 0.03945512 | 0.99993162 | -4.2927428 | -2.0594085 |
| BOD1L1 | 0.28180359 | 7.04227309 | 2.09367797 | 0.03945861 | 0.99993162 | -4.2840834 | 2.05937207 |
| LINC01203 | 1.99682344 | -5.0924211 | 2.09235949 | 0.03951785 | 0.99993162 | -4.2568783 | 2.05875363 |
| NOTCH2NLR | -0.9279832 | -1.5092011 | -2.0950571 | 0.03953005 | 0.99993162 | -4.3220733 | -2.0586263 |
| PFN2-AS1 | 0.854603 | -1.3647163 | 2.08990971 | 0.03974168 | 0.99993162 | -4.268652 | 2.05642381 |
| FAM187B2P | -1.9074737 | -4.8958673 | -2.0886184 | 0.03984974 | 0.99993162 | -4.3022443 | -2.0553031 |
| ARHGAP32 | 0.2623601 | 6.93285945 | 2.08958732 | 0.0398915 | 0.99993162 | -4.2860483 | 2.05487066 |
| POP5 | -0.2359082 | 4.39067597 | -2.0875884 | 0.04001184 | 0.99993162 | -4.2984003 | -2.0536266 |
| BOLA1 | -0.3910488 | 2.65290245 | -2.0852436 | 0.04029699 | 0.99993162 | -4.3073981 | -2.0506916 |
| MED4-AS1 | 1.70697285 | -5.385696 | 2.08057343 | 0.04035123 | 0.99993162 | -4.2669913 | 2.05013529 |
| LINC01821 | 1.55304705 | -4.9110184 | 2.08330497 | 0.04038472 | 0.99993162 | -4.2971691 | 2.04979211 |
| TNPO1P2 | 1.81696291 | -5.5197618 | 2.07950356 | 0.04043281 | 0.99993162 | -4.2683666 | 2.04929981 |
| LINC02765 | -1.9640236 | -3.8435016 | -2.083147 | 0.04051849 | 0.99993162 | -4.3132627 | -2.0484238 |
| RANBP2 | 0.31251554 | 7.2415128 | 2.08071524 | 0.04062497 | 0.99993162 | -4.2897731 | 2.0473374 |
| ZSCAN5C | -1.7559031 | -4.9721717 | -2.0766626 | 0.04075508 | 0.99993162 | -4.304347 | -2.0460131 |
| GCAT | -0.2811617 | 4.50040651 | -2.0797677 | 0.04078877 | 0.99993162 | -4.3129606 | -2.0456708 |
| NENF | -0.3486528 | 5.16328567 | -2.0801453 | 0.04079334 | 0.99993162 | -4.3040477 | -2.0456244 |
| PUS7L | 0.27295565 | 5.41158742 | 2.07851099 | 0.04093048 | 0.99993162 | -4.2890437 | 2.04423355 |
| RNY1P16 | -1.6911602 | -4.1673804 | -2.0794468 | 0.04094146 | 0.99993162 | -4.309104 | -2.0441224 |
| LINC01908 | -1.7520279 | -4.2192749 | -2.0771406 | 0.04116385 | 0.99993162 | -4.3004203 | -2.0418758 |
| RHO | 1.68846889 | -4.9509545 | 2.07323144 | 0.04123114 | 0.99993162 | -4.2647172 | 2.04119806 |
| FAM78B-AS1 | -1.6123043 | -4.5408213 | -2.0754005 | 0.04132125 | 0.99993162 | -4.3056575 | -2.0402921 |
| H1-2 | -0.8196676 | 0.60521116 | -2.0731198 | 0.04135262 | 0.99993162 | -4.3490241 | -2.0399769 |
| MIR377 | 1.55290114 | -6.3707882 | 2.07115015 | 0.0414618 | 0.99993162 | -4.2853241 | 2.03888209 |
| TULP2 | -1.9912953 | -5.4641922 | -2.0633515 | 0.04201398 | 0.99993162 | -4.3175009 | -2.0333816 |
| CRYL1 | -0.3461485 | 4.54853301 | -2.0657447 | 0.04215638 | 0.99993162 | -4.3183772 | -2.0319731 |
| CDC26P1 | -1.2345578 | -3.5049724 | -2.0662088 | 0.04223911 | 0.99993162 | -4.3098278 | -2.0311566 |
| MIR217HG | -1.784703 | -4.0357463 | -2.0640988 | 0.04225704 | 0.99993162 | -4.3449479 | -2.0309798 |
| TM6SF1 | 0.43410739 | 0.93808102 | 2.06692726 | 0.04226433 | 0.99993162 | -4.2763084 | 2.03090799 |
| RPS28 | -0.3820571 | 7.264096 | -2.0630803 | 0.04233596 | 0.99993162 | -4.3041206 | -2.0302025 |
| NOC2LP1 | -1.4822657 | -3.6025788 | -2.0615194 | 0.04243515 | 0.99993162 | -4.3150284 | -2.0292272 |
| ANKRD63 | -0.5888439 | 2.55709425 | -2.0607903 | 0.04253542 | 0.99993162 | -4.282925 | -2.0282434 |
| CSGALNACT2P1 | -1.4740128 | -4.4267517 | -2.0595734 | 0.04261199 | 0.99993162 | -4.3039023 | -2.0274933 |
| LINC00994 | 1.69360026 | -5.4696037 | 2.06075405 | 0.04263128 | 0.99993162 | -4.3037098 | 2.02730453 |
| RIC1 | 0.15246779 | 5.08103951 | 2.05963121 | 0.04272102 | 0.99993162 | -4.2938314 | 2.02642729 |
| IQSEC3-AS1 | 1.00449258 | -0.1213517 | 2.05867248 | 0.04274732 | 0.99993162 | -4.2764275 | 2.02617045 |
| IL1R2 | -1.9569934 | -4.6117774 | -2.058627 | 0.04277484 | 0.99993162 | -4.3324464 | -2.0259019 |
| UQCRQ | -0.3220365 | 5.48457392 | -2.0585344 | 0.04284779 | 0.99993162 | -4.3028145 | -2.0251906 |
| CIT | 0.36384895 | 5.96845259 | 2.05800444 | 0.0428542 | 0.99993162 | -4.2972274 | 2.02512821 |
| SBNO1 | 0.20920046 | 6.20672785 | 2.05728538 | 0.04294186 | 0.99993162 | -4.2984212 | 2.02427502 |
| SP2-DT | -0.5134762 | 2.07132675 | -2.0574701 | 0.04299627 | 0.99993162 | -4.323175 | -2.0237463 |
| ZNF37A | 0.22463776 | 5.14613846 | 2.05465917 | 0.04314909 | 0.99993162 | -4.2957816 | 2.02226397 |
| NEDD8P1 | -1.8758753 | -4.3644603 | -2.0526174 | 0.04334004 | 0.99993162 | -4.3141281 | -2.0204181 |
| PRDM10-DT | -1.0605296 | -2.5913568 | -2.0496052 | 0.04336176 | 0.99993162 | -4.303843 | -2.0202085 |
| MAD2L1-DT | -0.760938 | -0.3931722 | -2.0539615 | 0.0434457 | 0.99993162 | -4.3305293 | -2.0193997 |
| CLRN2 | -1.5813568 | -5.5298309 | -2.051535 | 0.04346028 | 0.99993162 | -4.3004323 | -2.0192592 |
| LINC02596 | 1.4341295 | -6.4846001 | 2.05115077 | 0.04347242 | 0.99993162 | -4.2879772 | 2.01914243 |
| LNCTSI | -1.6735442 | -6.3292187 | -2.0468747 | 0.04360772 | 0.99993162 | -4.2993001 | -2.017842 |
| SCARNA18 | 1.67328692 | -5.4169033 | 2.04563401 | 0.04388397 | 0.99993162 | -4.3083653 | 2.01519744 |
| RPL12P26 | 1.16901228 | -6.9583505 | 2.04612057 | 0.04394898 | 0.99993162 | -4.2953337 | 2.01457712 |
| STK24-AS1 | -0.7481803 | -0.8418074 | -2.0430281 | 0.04432317 | 0.99993162 | -4.3081452 | -2.0110217 |
| GJA9 | 1.50165677 | -4.9988532 | 2.03948214 | 0.04435645 | 0.99993162 | -4.2666261 | 2.01070669 |
| HEATR9 | 1.74044771 | -4.6140708 | 2.03800895 | 0.04450651 | 0.99993162 | -4.2774757 | 2.00928885 |
| FOXD2-AS1 | -2.3700341 | -4.2827131 | -2.0429504 | 0.04453966 | 0.99993162 | -4.3441638 | -2.0089762 |
| OSMR-DT | -1.2361947 | -0.347078 | -2.0426062 | 0.0445492 | 0.99993162 | -4.3714226 | -2.0088862 |
| CICP5 | -1.6495256 | -4.9361363 | -2.0397763 | 0.04459626 | 0.99993162 | -4.3318934 | -2.0084427 |
| MIR6867 | -1.512132 | -5.4331332 | -2.0370041 | 0.04465636 | 0.99993162 | -4.3056729 | -2.007877 |
| SLIT3-AS2 | -1.7000111 | -4.2148938 | -2.0366054 | 0.04469934 | 0.99993162 | -4.3357062 | -2.0074729 |
| ZNF114 | -0.495524 | 1.76220735 | -2.0370092 | 0.0449277 | 0.99993162 | -4.3305727 | -2.0053307 |
| MOCS1P1 | 1.54867495 | -6.2376478 | 2.03304626 | 0.04525252 | 0.99993162 | -4.2959939 | 2.00229964 |
| GDAP2 | 0.28884905 | 3.9225182 | 2.03257328 | 0.0453398 | 0.99993162 | -4.3135991 | 2.00148822 |
| AVPR1B | -1.8903926 | -3.4398637 | -2.0322057 | 0.04545405 | 0.99993162 | -4.3525816 | -2.0004281 |
| LAMA3 | 0.67466639 | 1.29627889 | 2.03324473 | 0.04550552 | 0.99993162 | -4.3030636 | 1.99995131 |
| LINC01856 | -1.7348347 | -5.9189201 | -2.0310706 | 0.04551432 | 0.99993162 | -4.3191929 | -1.9998699 |
| RN7SL181P | -0.7593572 | -1.841149 | -2.0274957 | 0.04561153 | 0.99993162 | -4.301501 | -1.9989707 |
| LINC01343 | 1.45093821 | -4.9071692 | 2.0307361 | 0.04563729 | 0.99993162 | -4.261168 | 1.99873261 |
| ERH | -0.2946162 | 6.30028932 | -2.0293609 | 0.04568919 | 0.99993162 | -4.3140615 | -1.9982535 |
| CPSF6 | 0.15799487 | 7.30596402 | 2.02879663 | 0.04583548 | 0.99993162 | -4.3108183 | 1.99690525 |
| RBAK | 0.18620741 | 5.15562928 | 2.02829802 | 0.04585017 | 0.99993162 | -4.3031571 | 1.99677005 |
| SNORD14E | 1.60774069 | -3.9378301 | 2.02679667 | 0.04589767 | 0.99993162 | -4.2773401 | 1.99633318 |
| MYL12B | -0.2709119 | 6.78046408 | -2.0271115 | 0.04590485 | 0.99993162 | -4.3139607 | -1.9962672 |
| RPL26P27 | 1.50336672 | -5.2138716 | 2.02648419 | 0.04604179 | 0.99993162 | -4.2635768 | 1.99501002 |
| LINC02724 | -0.5113584 | 0.05110413 | -2.026127 | 0.04613201 | 0.99993162 | -4.3442849 | -1.9941834 |
| ZNF662 | -1.4812485 | -3.9965362 | -2.0248637 | 0.04616875 | 0.99993162 | -4.3487402 | -1.9938472 |
| ERN1 | 0.18887162 | 4.36912155 | 2.02487172 | 0.04617954 | 0.99993162 | -4.2969664 | 1.99374853 |
| RPL7AP63 | 0.71240104 | -0.9392639 | 2.02486809 | 0.04623738 | 0.99993162 | -4.308433 | 1.99321984 |
| SNRPD2 | -0.2795826 | 5.93176024 | -2.022457 | 0.04648094 | 0.99993162 | -4.3186135 | -1.9909995 |
| RNF223 | 1.86315624 | -4.599774 | 2.02060606 | 0.04663102 | 0.99993162 | -4.3158796 | 1.9896362 |
| GOLGA2P11 | 1.22146152 | -6.096824 | 2.01896512 | 0.04673576 | 0.99993162 | -4.2971099 | 1.988687 |
| CADM2-AS1 | 1.86298245 | -4.9385176 | 2.01661357 | 0.04673876 | 0.99993162 | -4.2974754 | 1.98865986 |
| RPS15AP11 | 1.90537759 | -4.4862814 | 2.01731929 | 0.04693119 | 0.99993162 | -4.3130103 | 1.98692067 |
| LINC01111 | -1.9068351 | -5.3251475 | -2.0159537 | 0.0470691 | 0.99993162 | -4.3456417 | -1.9856779 |
| ZC3HC1 | -0.1458173 | 4.06327547 | -2.0155626 | 0.04728834 | 0.99993162 | -4.3228737 | -1.9837085 |
| GRM7-AS3 | -1.2130787 | -2.8151457 | -2.0151045 | 0.04728992 | 0.99993162 | -4.3432283 | -1.9836944 |
| PPIAP47 | 1.6361947 | -5.7402774 | 2.0136933 | 0.04740893 | 0.99993162 | -4.3043997 | 1.98262856 |
| LINC02134 | -1.3088227 | -1.4755659 | -2.0127099 | 0.04748515 | 0.99993162 | -4.3721294 | -1.9819472 |
| WDR54 | -0.2348456 | 5.25056103 | -2.0128357 | 0.04752036 | 0.99993162 | -4.3282553 | -1.9816328 |
| MTND4P15 | 1.87076073 | -5.4553381 | 2.01200315 | 0.04752874 | 0.99993162 | -4.3117522 | 1.98155796 |
| PGBD4 | 0.2433197 | 2.89324895 | 2.01260187 | 0.04753892 | 0.99993162 | -4.3271697 | 1.98146703 |
| OXCT2P1 | -1.7184397 | -4.092528 | -2.0118769 | 0.04769911 | 0.99993162 | -4.3369371 | -1.9800393 |
| KLF2-DT | 1.42088814 | -5.2896969 | 2.00806233 | 0.04796074 | 0.99993162 | -4.2694226 | 1.9777161 |
| LINC01217 | -1.5557296 | -6.1596248 | -2.009224 | 0.04796757 | 0.99993162 | -4.3258245 | -1.9776556 |
| GJB5 | -1.871804 | -4.0622021 | -2.0077877 | 0.04797747 | 0.99993162 | -4.3586297 | -1.9775679 |
| GK3 | -1.5571086 | -5.4373825 | -2.0070234 | 0.0480831 | 0.99993162 | -4.3156749 | -1.9766332 |
| RNU4-52P | -1.6352747 | -4.9296685 | -2.0065255 | 0.04816737 | 0.99993162 | -4.3160491 | -1.9758888 |
| SMCHD1 | 0.20087144 | 6.92471218 | 2.00709968 | 0.04819748 | 0.99993162 | -4.3199499 | 1.97562314 |
| HSD17B10 | -0.2283645 | 5.48330459 | -2.0061925 | 0.04827267 | 0.99993162 | -4.329433 | -1.9749601 |
| SMDT1 | -0.2472741 | 4.96981681 | -2.0058362 | 0.04830734 | 0.99993162 | -4.3335971 | -1.9746548 |
| RTL10 | 0.16007253 | 5.45369333 | 2.00451562 | 0.04843178 | 0.99993162 | -4.3155672 | 1.97356011 |
| FBXO22 | 0.17924295 | 5.4377228 | 2.00161802 | 0.04863857 | 0.99993162 | -4.3162475 | 1.97174638 |
| RBM33 | 0.31252532 | 7.6353165 | 2.00072147 | 0.04876969 | 0.99993162 | -4.3188191 | 1.97059963 |
| SERPINA10 | 1.3654973 | -3.1379991 | 2.00115429 | 0.04879429 | 0.99993162 | -4.3169387 | 1.97038473 |
| LRTOMT | 0.82092383 | -2.6147169 | 2.00261798 | 0.04886696 | 0.99993162 | -4.3191384 | 1.96975061 |
| GATAD2B | 0.24358086 | 6.90113518 | 1.99988283 | 0.04887977 | 0.99993162 | -4.3221816 | 1.96963894 |
| LINC01215 | -1.6348286 | -6.1420407 | -1.9996737 | 0.04889427 | 0.99993162 | -4.3198038 | -1.9695125 |
| FAAP20 | -0.1937945 | 5.7331599 | -1.9992604 | 0.04895952 | 0.99993162 | -4.3255102 | -1.968944 |
| VSIG10L2 | 1.75057292 | -3.9702534 | 1.99993576 | 0.04896135 | 0.99993162 | -4.3282777 | 1.96892809 |
| ZNF879 | 0.22349542 | 3.16101777 | 1.99899764 | 0.04900997 | 0.99993162 | -4.3158617 | 1.96850493 |
| DGCR11 | 0.32043885 | 0.54867847 | 1.99875479 | 0.04910725 | 0.99993162 | -4.3162529 | 1.96765927 |
| CRIP2 | -0.2765008 | 7.35184044 | -1.9956647 | 0.04935328 | 0.99993162 | -4.326438 | -1.9655269 |
| CCDC194 | -1.7730995 | -3.6834865 | -1.9954707 | 0.04941102 | 0.99993162 | -4.3501637 | -1.9650278 |
| OPN1SW | -1.147172 | -2.3802657 | -1.9954754 | 0.049425 | 0.99993162 | -4.3458182 | -1.964907 |
| BRCA1P1 | 0.60827051 | -1.2448797 | 1.99444763 | 0.04942764 | 0.99993162 | -4.303635 | 1.96488423 |
| NUP153-AS1 | -1.592123 | -3.0135157 | -1.998371 | 0.04944669 | 0.99993162 | -4.3579418 | -1.9647197 |
| GALK1 | -0.3512376 | 3.98345546 | -1.9952461 | 0.04947367 | 0.99993162 | -4.3384842 | -1.9644868 |
| LINC01504 | 1.16113582 | -2.2404 | 1.99627713 | 0.04948797 | 0.99993162 | -4.2949982 | 1.96436332 |
| NHLRC1 | -0.5653885 | 1.01805776 | -1.9935755 | 0.0495887 | 0.99993162 | -4.3454621 | -1.9634949 |
| RAD54L2 | 0.21270315 | 6.2488808 | 1.99231379 | 0.04973843 | 0.99993162 | -4.3213763 | 1.96220666 |
| MIR1302-9HG | -1.0005372 | -1.5964551 | -1.9909976 | 0.04986874 | 0.99993162 | -4.3473502 | -1.9610882 |
| SNORA19 | -1.708888 | -5.5976235 | -1.9877035 | 0.04993068 | 0.99993162 | -4.3317662 | -1.9605574 |
| RPL15P21 | -1.7195427 | -5.9339042 | -1.9891911 | 0.04997698 | 0.99993162 | -4.3296176 | -1.960161 |
| HHLA1 | 1.76413451 | -4.4428221 | 1.99041305 | 0.04997969 | 0.99993162 | -4.3250367 | 1.96013779 |
| ACVR2B | 0.34224602 | 5.90526219 | 1.99059992 | 0.04998709 | 0.99993162 | -4.3255511 | 1.96007443 |

**Supplementary Table S8:** Differentially Expressed Genes after (2*R*,6*R*)-HNK at 24 Hours

| **Gene Name** | **logFC** | **AveExpr** | **t** | **P.Value** | **adj.P.Val** | **B** | **z.std** |
| --- | --- | --- | --- | --- | --- | --- | --- |
| RNU6-583P | -2.1465677 | -5.0909391 | -4.1679801 | 7.6445E-05 | 0.96713357 | -2.1795074 | -3.9552817 |
| MTND5P15 | -1.9671833 | -6.0173454 | -3.9076988 | 0.00019224 | 0.96713357 | -2.5063007 | -3.7289988 |
| DOCK6-AS1 | -1.7437259 | -2.952778 | -3.8682404 | 0.00021563 | 0.96713357 | -2.4931001 | -3.6999651 |
| LINC02970 | 2.49084858 | -5.6240562 | 3.81195045 | 0.00027959 | 0.96713357 | -2.666316 | 3.63350822 |
| DOCK2 | 1.04821237 | -0.9126395 | 3.68479757 | 0.00041298 | 0.96713357 | -2.7279897 | 3.53164771 |
| GIMAP2 | -2.0284387 | -6.2135406 | -3.5970059 | 0.0005558 | 0.96713357 | -2.6844384 | -3.4523128 |
| OR3A2 | -1.738603 | -6.3528106 | -3.56934 | 0.00060578 | 0.96713357 | -2.7383673 | -3.4290141 |
| RAC2 | -1.8500761 | -3.8398624 | -3.5460628 | 0.00064874 | 0.96713357 | -2.8847683 | -3.4103741 |
| PRG4 | -1.1288362 | -2.3902667 | -3.5293061 | 0.00069368 | 0.96713357 | -2.6452105 | -3.3920635 |
| MCM8-AS1 | 1.99404978 | -4.8351253 | 3.41726246 | 0.00095757 | 0.96713357 | -3.0787642 | 3.30270638 |
| LINC02895 | -1.8199665 | -6.2884228 | -3.3791112 | 0.00108305 | 0.96713357 | -2.89429 | -3.2680149 |
| GNGT2 | -1.7066828 | -5.5840751 | -3.3587688 | 0.00118751 | 0.96713357 | -3.0432694 | -3.2418636 |
| TAF12-DT | -1.1756391 | -3.2090904 | -3.3148525 | 0.00137605 | 0.96713357 | -3.0982314 | -3.1996286 |
| ABHD12B | -0.9542562 | -1.7760945 | -3.3009062 | 0.0014484 | 0.96713357 | -3.1356947 | -3.1848287 |
| HMGA2-AS1 | 1.87225755 | -6.0502992 | 3.19945347 | 0.00196524 | 0.96713357 | -3.208911 | 3.09543544 |
| HTATIP2 | 0.97284635 | 0.49475154 | 3.18203125 | 0.00208456 | 0.96713357 | -3.3219733 | 3.07791319 |
| RNASE4 | -1.808427 | -5.4160725 | -3.1168818 | 0.00255031 | 0.96713357 | -3.2831098 | -3.0173073 |
| RPL28P2 | -1.1705519 | -1.3749918 | -3.1127742 | 0.00255206 | 0.96713357 | -3.42492 | -3.0171001 |
| LRRC37A11P | -1.6448698 | -4.1438574 | -3.1170817 | 0.00255694 | 0.96713357 | -3.2213643 | -3.016521 |
| BANCR | 2.04819091 | -5.4743063 | 3.10326181 | 0.00261006 | 0.96713357 | -3.2852528 | 3.01028113 |
| CYCSP8 | -1.5156382 | -3.6424004 | -3.08396 | 0.00281554 | 0.96713357 | -3.3043732 | -2.987191 |
| RPL24P6 | -1.6994361 | -4.7750398 | -3.0584656 | 0.00299268 | 0.96713357 | -3.371665 | -2.968489 |
| CLEC20A | -1.6021682 | -6.0599058 | -3.0453089 | 0.00311028 | 0.96713357 | -3.3134069 | -2.9566229 |
| CDR2-DT | -1.8556774 | -5.1637106 | -3.0229288 | 0.00331335 | 0.96713357 | -3.325208 | -2.9370644 |
| MCMDC2 | -0.4954673 | -0.3013673 | -3.026014 | 0.00335575 | 0.96713357 | -3.3590357 | -2.9331191 |
| NPM1P37 | -1.5725057 | -4.3786884 | -3.0141623 | 0.00340215 | 0.96713357 | -3.418093 | -2.9288532 |
| SUGT1P2 | 1.87261909 | -3.9667067 | 3.02425374 | 0.00343309 | 0.96713357 | -3.5337521 | 2.92603795 |
| ZNF473CR | -0.3688977 | 0.37752955 | -3.0092696 | 0.00350597 | 0.96713357 | -3.3506312 | -2.9194966 |
| RINL | 0.52901822 | 0.58609183 | 3.00267892 | 0.00358074 | 0.96713357 | -3.4696976 | 2.91291336 |
| HNRNPA3P4 | -1.6629791 | -5.8283677 | -2.995832 | 0.00362491 | 0.96713357 | -3.339955 | -2.9090831 |
| OMP | -1.915549 | -5.3315835 | -2.9925869 | 0.00363247 | 0.96713357 | -3.3860411 | -2.9084317 |
| APCDD1L | 2.38540215 | -2.1272077 | 2.99193892 | 0.00368218 | 0.96713357 | -3.5214879 | 2.90417862 |
| RAD21-AS1 | -1.1713867 | -3.0776053 | -2.9880562 | 0.00368503 | 0.96713357 | -3.3742747 | -2.9039365 |
| MIR548I2 | -1.1234409 | -6.6024115 | -2.9893585 | 0.00369558 | 0.96713357 | -3.3412958 | -2.9030414 |
| NETO1-DT | -0.8518765 | -0.9512407 | -2.9830926 | 0.00377568 | 0.96713357 | -3.4347716 | -2.8963191 |
| HSPB9 | -1.3814088 | -3.1827985 | -2.9705277 | 0.00382668 | 0.96713357 | -3.4492026 | -2.8921067 |
| EFCAB10-AS1 | -0.3425649 | 1.49749263 | -2.9679409 | 0.00393622 | 0.96713357 | -3.4422031 | -2.8832286 |
| SPPL2C | -1.3775735 | -2.6761503 | -2.9538935 | 0.00408289 | 0.96713357 | -3.5675733 | -2.8716855 |
| RN7SL812P | -1.4770919 | -4.8888578 | -2.9563004 | 0.00409019 | 0.96713357 | -3.413479 | -2.8711209 |
| DDN-AS1 | -0.6415987 | -1.1112892 | -2.9541505 | 0.00409981 | 0.96713357 | -3.4790208 | -2.870378 |
| DIP2A-IT1 | -1.3802202 | -4.2497041 | -2.9479937 | 0.00412483 | 0.96713357 | -3.403675 | -2.868454 |
| MOCOS | 0.70818122 | -0.1403864 | 2.93505708 | 0.00434934 | 0.96713357 | -3.5234995 | 2.85164676 |
| KLHDC7A | 1.54488696 | -3.1152611 | 2.93338606 | 0.00436425 | 0.96713357 | -3.3768884 | 2.85055839 |
| FGF17 | -0.8653775 | 1.43763814 | -2.9353676 | 0.00436762 | 0.96713357 | -3.448423 | -2.8503129 |
| KCNJ16 | -0.9206813 | 0.87575226 | -2.9172202 | 0.00458301 | 0.96713357 | -3.1307144 | -2.8349691 |
| ROPN1B | -0.8761308 | -1.0893863 | -2.9157605 | 0.00459702 | 0.96713357 | -3.4985214 | -2.8339942 |
| PNN-AS1 | 1.32207997 | -3.3782381 | 2.9132997 | 0.00462215 | 0.96713357 | -3.6039857 | 2.83225124 |
| CD207 | -1.5929947 | -6.0008358 | -2.907383 | 0.00468517 | 0.96713357 | -3.4114594 | -2.8279188 |
| ASXL3-DT | -1.5693739 | -5.1654218 | -2.9033494 | 0.00476276 | 0.96713357 | -3.5115802 | -2.8226562 |
| RPS6P25 | -0.8278216 | -1.4633804 | -2.9001457 | 0.00477562 | 0.96713357 | -3.4582267 | -2.8217914 |
| RBM17P1 | 1.30478259 | -6.7128234 | 2.89207366 | 0.00489045 | 0.96713357 | -3.4747085 | 2.81416258 |
| GAS2L1P2 | -1.445404 | -3.7635055 | -2.8940076 | 0.0048918 | 0.96713357 | -3.4263354 | -2.8140736 |
| TGFB3-AS1 | -1.4676213 | -4.6381159 | -2.8836222 | 0.00500673 | 0.96713357 | -3.4316612 | -2.8066008 |
| WDR81 | 0.18128926 | 5.28814819 | 2.88323172 | 0.00506314 | 0.96713357 | -3.4900534 | 2.80298891 |
| RNU6-600P | -1.6401344 | -4.3249632 | -2.8716962 | 0.00522962 | 0.96713357 | -3.475201 | -2.792539 |
| DEFB134 | -1.5408864 | -5.9911923 | -2.8676695 | 0.0052579 | 0.96713357 | -3.5015943 | -2.7907933 |
| C3 | 2.27090917 | 0.78233119 | 2.86075934 | 0.00542268 | 0.96713357 | -3.4260963 | 2.78079024 |
| GRIK1-AS1 | -0.6648671 | -0.1032732 | -2.8461715 | 0.00561061 | 0.96713357 | -3.5137462 | -2.7697107 |
| PLIN2 | 0.68309626 | 3.71140022 | 2.84349221 | 0.00568758 | 0.96713357 | -3.6144729 | 2.76526941 |
| RELB | 0.70489932 | 3.07444808 | 2.84491877 | 0.00572338 | 0.96713357 | -3.5712493 | 2.76322202 |
| RPS20P10 | -1.715783 | -5.5166817 | -2.8332379 | 0.00580816 | 0.96713357 | -3.4398426 | -2.7584198 |
| GLCCI1-DT | -1.0405615 | -3.2987742 | -2.8339234 | 0.00582366 | 0.96713357 | -3.5859614 | -2.7575482 |
| ATP5IF1 | -0.2409669 | 4.75411495 | -2.841765 | 0.00583292 | 0.96713357 | -3.5120373 | -2.7570291 |
| KLHL41 | -0.4923537 | 0.29037798 | -2.8121643 | 0.00620494 | 0.96713357 | -3.5709577 | -2.7367503 |
| FGF19 | -2.398665 | -3.5606574 | -2.7982911 | 0.00631868 | 0.96713357 | -3.5037926 | -2.7307688 |
| ITIH6 | -1.5247904 | -5.9424538 | -2.7994656 | 0.00639585 | 0.96713357 | -3.4770202 | -2.7267656 |
| FOSL2 | 0.79387702 | 6.07696032 | 2.80315323 | 0.00639887 | 0.96713357 | -3.5982426 | 2.72660984 |
| WBP2P1 | -1.3451116 | -6.6368643 | -2.7960431 | 0.00644792 | 0.96713357 | -3.4774036 | -2.7240884 |
| EIF1P3 | -1.80954 | -5.7018268 | -2.7828362 | 0.00657287 | 0.96713357 | -3.5494561 | -2.7177439 |
| TNIP1 | 0.45481105 | 5.47166478 | 2.78932918 | 0.00659085 | 0.96713357 | -3.5425903 | 2.71683992 |
| IL34 | 0.9475917 | 1.96376999 | 2.78877108 | 0.00661187 | 0.96713357 | -3.6405782 | 2.71578593 |
| MIR4489 | -1.120828 | -3.2338591 | -2.7963899 | 0.00661383 | 0.96713357 | -3.5596632 | -2.7156877 |
| RPL7P17 | -1.4318008 | -3.9119492 | -2.7830169 | 0.00667643 | 0.96713357 | -3.5968579 | -2.7125667 |
| IL21-AS1 | -1.6664811 | -5.8045289 | -2.7768156 | 0.00680619 | 0.96713357 | -3.47783 | -2.7061809 |
| RIC3-DT | -1.0410875 | -2.9471512 | -2.7723949 | 0.00691059 | 0.96713357 | -3.6249275 | -2.7011225 |
| MYH9 | 0.38992232 | 7.51762081 | 2.77263891 | 0.00693034 | 0.96713357 | -3.5736435 | 2.70017327 |
| MIR5695 | -1.7346822 | -5.2513918 | -2.7712955 | 0.00700557 | 0.96713357 | -3.5860969 | -2.6965795 |
| LINC01979 | 0.69242587 | -0.659015 | 2.76952002 | 0.00701678 | 0.96713357 | -3.7491104 | 2.69604704 |
| MRPL35P2 | 1.49504348 | -4.0023933 | 2.76547154 | 0.00707696 | 0.96713357 | -3.6369421 | 2.69320098 |
| ITPKB-IT1 | 1.57348842 | -6.0282751 | 2.75826003 | 0.00720017 | 0.96713357 | -3.6322563 | 2.68744154 |
| LPO | 1.23048327 | -3.2106977 | 2.7522794 | 0.00724941 | 0.96713357 | -3.6901958 | 2.68516434 |
| MIDEAS | 0.30355223 | 6.19927836 | 2.75491526 | 0.00729408 | 0.96713357 | -3.5976882 | 2.68311072 |
| MAL | 1.05985429 | -0.6646216 | 2.75029693 | 0.00736358 | 0.96713357 | -3.6624858 | 2.67993788 |
| RNU6-969P | -1.6362729 | -4.7263258 | -2.7462723 | 0.00740046 | 0.96713357 | -3.55595 | -2.6782652 |
| TTLL10-AS1 | -1.1772477 | -5.0854725 | -2.7438818 | 0.00749003 | 0.96713357 | -3.5622545 | -2.6742336 |
| SEPTIN14 | -1.5350772 | -5.3986859 | -2.7317664 | 0.00769995 | 0.96713357 | -3.5837758 | -2.6649511 |
| DRD5 | -1.304814 | -3.3395865 | -2.7325865 | 0.00773939 | 0.96713357 | -3.6137465 | -2.6632324 |
| MIR6865 | -1.4264443 | -4.2936247 | -2.7299712 | 0.00774716 | 0.96713357 | -3.6239926 | -2.662895 |
| ESAM-AS1 | -0.8889892 | -1.1960287 | -2.7285165 | 0.00778664 | 0.96713357 | -3.7151322 | -2.6611841 |
| AHR | 0.56038227 | 4.0613598 | 2.72829719 | 0.00783803 | 0.96713357 | -3.5586552 | 2.65896831 |
| PCSK6 | 0.93068282 | 3.50746129 | 2.73001785 | 0.00785004 | 0.96713357 | -3.7039519 | 2.6584524 |
| CLDN1 | 1.02968556 | 1.96182313 | 2.72859034 | 0.00785953 | 0.96713357 | -3.5743241 | 2.65804552 |
| RN7SL787P | -1.2950793 | -5.9656269 | -2.7225901 | 0.00791243 | 0.96713357 | -3.630053 | -2.6557841 |
| TMEM139 | -0.7440443 | -1.8641165 | -2.7254072 | 0.0079133 | 0.96713357 | -3.6323973 | -2.6557469 |
| PACRG-AS1 | -0.9192839 | -1.8286021 | -2.7206617 | 0.00796633 | 0.96713357 | -3.6136346 | -2.6534933 |
| TPH2 | -1.6589904 | -2.9217319 | -2.7153825 | 0.00806828 | 0.96713357 | -3.5722961 | -2.6491989 |
| ZNF582-DT | -0.3316473 | 1.48358088 | -2.7152924 | 0.00814226 | 0.96713357 | -3.6327218 | -2.6461129 |
| RNU6-137P | 1.44677237 | -4.9421578 | 2.71318301 | 0.00825622 | 0.96713357 | -3.6631978 | 2.64140805 |
| LINC00427 | 1.62643478 | -5.9640428 | 2.69846581 | 0.00842396 | 0.96713357 | -3.6875066 | 2.63458761 |
| TMEM156 | 1.62354524 | -6.0833755 | 2.69623869 | 0.00847145 | 0.96713357 | -3.6437766 | 2.6326784 |
| PXDNL | 1.37782514 | 2.72947386 | 2.69688995 | 0.00853844 | 0.96713357 | -3.6352011 | 2.63000175 |
| SNORA80E | -1.3367898 | -4.6371247 | -2.6925601 | 0.00854017 | 0.96713357 | -3.5971806 | -2.6299329 |
| LINC02675 | -1.4254454 | -3.2953294 | -2.6949088 | 0.00860341 | 0.96713357 | -3.7224646 | -2.6274237 |
| MATR3P3 | -1.5414968 | -3.7527829 | -2.687972 | 0.00865185 | 0.96713357 | -3.5833231 | -2.6255131 |
| HULC | -0.2915643 | 2.29431394 | -2.6868743 | 0.0087724 | 0.96713357 | -3.700449 | -2.620799 |
| CD74 | 1.43340649 | 2.24833037 | 2.6770422 | 0.00901956 | 0.96713357 | -3.5790853 | 2.61131181 |
| DNAJC8P2 | -1.2815927 | -3.5562646 | -2.6697776 | 0.00905326 | 0.96713357 | -3.6260932 | -2.6100365 |
| RAP2B | 0.22118044 | 5.79779679 | 2.66974578 | 0.00919156 | 0.96713357 | -3.655884 | 2.60484572 |
| FTLP12 | 1.25488555 | -2.6866879 | 2.66853074 | 0.00922698 | 0.96713357 | -3.7245002 | 2.60352763 |
| GUCD1 | 0.24481249 | 5.89819023 | 2.67089379 | 0.0092644 | 0.96713357 | -3.6552541 | 2.60214015 |
| NXPE1 | -1.545801 | -4.4341649 | -2.6657876 | 0.00926824 | 0.96713357 | -3.6420789 | -2.6019981 |
| LINC01589 | -1.2334582 | -3.7290524 | -2.6638054 | 0.00932812 | 0.96713357 | -3.6517179 | -2.5997885 |
| LINC01978 | 0.74039619 | -1.5734623 | 2.66489355 | 0.00936285 | 0.96713357 | -3.787557 | 2.59851298 |
| PPIAP55 | 1.6468341 | -5.9995141 | 2.66183784 | 0.00938996 | 0.96713357 | -3.6926557 | 2.59752029 |
| ACTR3-AS1 | -0.4513369 | 0.48441996 | -2.659452 | 0.00948903 | 0.96713357 | -3.7365367 | -2.5939135 |
| RPL7AP64 | 1.9257268 | -5.7231227 | 2.65482858 | 0.00951645 | 0.96713357 | -3.6831789 | 2.59292161 |
| ZNF277-AS1 | -0.5794089 | -1.0396673 | -2.6536322 | 0.0095334 | 0.96713357 | -3.6509653 | -2.5923093 |
| CXCL1 | 2.0206477 | -2.457304 | 2.65552581 | 0.0097148 | 0.96713357 | -3.6486505 | 2.58581885 |
| VSTM1 | 1.61771972 | -1.5775228 | 2.64669151 | 0.00982123 | 0.96713357 | -3.7769482 | 2.58206083 |
| THAP5P1 | -1.336206 | -3.8534702 | -2.6438191 | 0.00984899 | 0.96713357 | -3.6766849 | -2.5810865 |
| LINC01993 | 1.64021596 | -0.1271592 | 2.64581154 | 0.00985831 | 0.96713357 | -3.8074293 | 2.58076002 |
| VPS13B-DT | -0.4231791 | 1.64756056 | -2.6490192 | 0.00987171 | 0.96713357 | -3.7071493 | -2.5802911 |
| LINC02396 | -1.8048973 | -4.8098583 | -2.6361765 | 0.00991619 | 0.96713357 | -3.7088749 | -2.5787382 |
| CPM | 0.91687724 | -0.2423528 | 2.64022982 | 0.00994789 | 0.96713357 | -3.7002645 | 2.57763538 |
| FMO3 | -1.2040689 | -6.0154981 | -2.6390703 | 0.00998684 | 0.96713357 | -3.6767904 | -2.5762847 |
| LINC01356 | -0.8857009 | -2.7791748 | -2.6363517 | 0.01005126 | 0.96713357 | -3.7012584 | -2.5740608 |
| PYCARD | 1.1872676 | -2.7239649 | 2.63441005 | 0.01011689 | 0.96713357 | -3.7821483 | 2.57180842 |
| SMIM1 | 1.29813102 | -2.8129495 | 2.63116386 | 0.01013187 | 0.96713357 | -3.6243053 | 2.57129603 |
| DES | 1.80507128 | -1.6716199 | 2.63016693 | 0.01022346 | 0.96713357 | -3.6946586 | 2.56817829 |
| P2RX5-TAX1BP3 | 0.24089583 | 1.38880012 | 2.62924859 | 0.01026713 | 0.96713357 | -3.7145708 | 2.56670027 |
| THORLNC | 0.79040606 | -1.7578464 | 2.61068158 | 0.01073064 | 0.96713357 | -3.7353198 | 2.55135019 |
| IRF2 | 0.24015073 | 3.89051417 | 2.6127858 | 0.01074243 | 0.96713357 | -3.7064122 | 2.55096756 |
| IPMKP1 | -1.4720547 | -5.0001399 | -2.6002533 | 0.0109358 | 0.96713357 | -3.7662342 | -2.5447436 |
| FURIN | 0.25669481 | 6.30036021 | 2.60678107 | 0.01093673 | 0.96713357 | -3.7174482 | 2.54471386 |
| CLCA2 | 1.35916932 | -2.8286589 | 2.59675851 | 0.0111765 | 0.96713357 | -3.746279 | 2.53713123 |
| PURG | -0.3578724 | 4.09828314 | -2.5980943 | 0.0111985 | 0.96713357 | -3.6874564 | -2.536443 |
| PAQR5-DT | 1.55616737 | -6.1277218 | 2.58893897 | 0.01125445 | 0.96713357 | -3.754293 | 2.53469724 |
| CDYL | 0.26532064 | 5.0875829 | 2.59400315 | 0.01130035 | 0.96713357 | -3.7209799 | 2.53327121 |
| LIF | 1.02896045 | 1.07420664 | 2.59350686 | 0.01132801 | 0.96713357 | -3.734398 | 2.53241427 |
| ODF1 | -1.3972914 | -6.1450445 | -2.588916 | 0.01135379 | 0.96713357 | -3.7305362 | -2.531617 |
| LINC01101 | 1.71423628 | -6.2712088 | 2.58904754 | 0.01137123 | 0.96713357 | -3.7376707 | 2.53107879 |
| H3P25 | 1.62556021 | -5.4990777 | 2.58907692 | 0.0114317 | 0.96713357 | -3.7094419 | 2.52921798 |
| COLEC11 | -1.2439092 | -1.983241 | -2.5863903 | 0.01159868 | 0.96713357 | -3.8404372 | -2.5241246 |
| NFKB1 | 0.48151781 | 4.36603264 | 2.58084143 | 0.01174025 | 0.96713357 | -3.743818 | 2.51985711 |
| ELF1 | 0.25256751 | 4.15377333 | 2.5711093 | 0.01200537 | 0.96713357 | -3.7077715 | 2.51198648 |
| ICOSLG | 1.15250205 | -0.3061149 | 2.57498772 | 0.01204842 | 0.96713357 | -3.6605745 | 2.51072291 |
| CDHR18P | -0.5491776 | -0.8265239 | -2.5664207 | 0.01213342 | 0.96713357 | -3.7578238 | -2.5082401 |
| ADAMTS7P3 | -1.2850866 | -2.4086464 | -2.5660888 | 0.01214156 | 0.96713357 | -3.7964342 | -2.5080031 |
| TNNC1 | -0.7920314 | -1.8383887 | -2.5628472 | 0.0122391 | 0.96713357 | -3.7572926 | -2.5051745 |
| LINC01465 | 0.45353621 | -0.0676222 | 2.56218742 | 0.0123334 | 0.96713357 | -3.7786853 | 2.50245867 |
| CAAP1 | 0.18835931 | 4.45454642 | 2.55915349 | 0.0124054 | 0.96713357 | -3.7560599 | 2.50039771 |
| FOXD2 | -1.3178048 | -5.7292897 | -2.553135 | 0.01257624 | 0.96713357 | -3.6973549 | -2.4955491 |
| CTRB1 | -1.0059269 | -2.1704658 | -2.545329 | 0.01280342 | 0.96713357 | -3.8740032 | -2.4891908 |
| GBP1 | 1.28144539 | 1.62464706 | 2.54551726 | 0.01287935 | 0.96713357 | -3.7639523 | 2.48708817 |
| NCOA4P2 | -1.4766627 | -3.8511364 | -2.5366476 | 0.01310615 | 0.96713357 | -3.7503098 | -2.4808719 |
| MIR320D1 | -1.3551992 | -4.0638041 | -2.5314682 | 0.01326495 | 0.96713357 | -3.7776597 | -2.4765757 |
| RAB42 | 0.7572339 | 1.2277891 | 2.53235563 | 0.01337555 | 0.96713357 | -3.8350885 | 2.47361042 |
| TRAPPC6A | 0.32199889 | 3.3700052 | 2.53108823 | 0.01345417 | 0.96713357 | -3.7789944 | 2.47151558 |
| NPSR1-AS1 | 1.50797968 | -4.7632867 | 2.5256049 | 0.01350076 | 0.96713357 | -3.8233235 | 2.47027926 |
| KRT17P3 | -1.2587497 | -5.2663618 | -2.5249742 | 0.01351295 | 0.96713357 | -3.7657322 | -2.4699566 |
| ACYP1 | -0.280987 | 3.10459247 | -2.5260942 | 0.01351987 | 0.96713357 | -3.7848286 | -2.4697733 |
| DMGDH | 0.37399377 | 1.76567396 | 2.52762962 | 0.01352998 | 0.96713357 | -3.8587724 | 2.46950591 |
| GASK1A | -0.7079131 | -0.8372744 | -2.5222799 | 0.01363207 | 0.96713357 | -3.6644312 | -2.4668151 |
| GALNT5 | 1.00684632 | 1.69667362 | 2.52207096 | 0.01367729 | 0.96713357 | -3.8355448 | 2.46562898 |
| HOXA11 | -0.7126789 | -5.7811207 | -2.5194881 | 0.0137277 | 0.96713357 | -3.6826791 | -2.4643108 |
| ACTR5 | -0.1928484 | 3.90016972 | -2.5171684 | 0.01380857 | 0.96713357 | -3.808661 | -2.462205 |
| FAAP24 | 0.26379119 | 2.13017747 | 2.51173034 | 0.01400248 | 0.96713357 | -3.783533 | 2.45719966 |
| SLC6A13 | -1.248264 | -2.6377278 | -2.510057 | 0.01402872 | 0.96713357 | -3.7573847 | -2.4565271 |
| DYRK3-AS1 | 1.09625356 | -2.8497237 | 2.51340116 | 0.01410494 | 0.96713357 | -3.8282654 | 2.45457963 |
| LINC00330 | -1.1013272 | -6.2585826 | -2.50863 | 0.01411247 | 0.96713357 | -3.7812278 | -2.4543878 |
| ABHD17AP4 | -1.5588794 | -5.7198607 | -2.5016926 | 0.01417847 | 0.96713357 | -3.7302021 | -2.4527098 |
| CCN3 | 0.91402046 | 3.61289269 | 2.50591 | 0.01426538 | 0.96713357 | -3.7654954 | 2.45051046 |
| ZSCAN16-AS1 | -0.2504624 | 2.37269758 | -2.5029002 | 0.01429789 | 0.96713357 | -3.8060212 | -2.4496908 |
| RPL15P11 | -1.3545833 | -4.3595814 | -2.503378 | 0.01437328 | 0.96713357 | -3.7788341 | -2.4477965 |
| SGMS1-AS1 | 0.3279363 | 3.06686694 | 2.50297572 | 0.01437847 | 0.96713357 | -3.8240616 | 2.44766641 |
| SLC44A1 | 0.27892963 | 6.64738194 | 2.50148127 | 0.01442514 | 0.96713357 | -3.7915041 | 2.4464985 |
| H1-3 | 1.45931983 | -6.0656818 | 2.49917339 | 0.01444331 | 0.96713357 | -3.7822 | 2.44604472 |
| VPS35P1 | -1.601224 | -5.127402 | -2.4937911 | 0.01449741 | 0.96713357 | -3.747998 | -2.4446963 |
| LYPLA1P3 | -1.2567487 | -3.9689489 | -2.495593 | 0.01456389 | 0.96713357 | -3.8002577 | -2.4430458 |
| POLR2KP1 | 1.49004875 | -4.9449511 | 2.48899799 | 0.01465997 | 0.96713357 | -3.7775753 | 2.44067174 |
| NUDT18 | 0.2817728 | 2.69176702 | 2.49499227 | 0.01469175 | 0.96713357 | -3.8232582 | 2.43988958 |
| TTC16 | -0.9812655 | -1.9026095 | -2.4912444 | 0.01476606 | 0.96713357 | -3.80286 | -2.4380665 |
| FABP5P11 | -1.4382164 | -5.6408039 | -2.4903417 | 0.01479593 | 0.96713357 | -3.7572751 | -2.4373358 |
| OR7C1 | -1.3383279 | -4.7358704 | -2.4927307 | 0.01482915 | 0.96713357 | -3.7577103 | -2.4365248 |
| LINC02151 | -1.2464001 | -5.002293 | -2.4886949 | 0.0148588 | 0.96713357 | -3.7784816 | -2.4358024 |
| MGAT1 | 0.18434096 | 6.43882121 | 2.49016013 | 0.01490213 | 0.96713357 | -3.7990987 | 2.4347488 |
| LINC02916 | 0.51267843 | 0.22574804 | 2.47798052 | 0.01529679 | 0.96713357 | -3.8511887 | 2.42527519 |
| PML | 0.2362846 | 5.19131448 | 2.47938837 | 0.01529892 | 0.96713357 | -3.8045918 | 2.42522445 |
| RNU7-140P | -1.5684431 | -3.7880437 | -2.4751882 | 0.01531684 | 0.96713357 | -3.8596571 | -2.4247995 |
| C8G | -0.5261243 | -0.3567798 | -2.4801576 | 0.01532917 | 0.96713357 | -3.9021307 | -2.4245075 |
| CAV1 | 0.94195602 | 3.04936222 | 2.47432369 | 0.01549824 | 0.96713357 | -3.7012308 | 2.42052207 |
| CLPTM1L | 0.16038622 | 6.59739537 | 2.47141379 | 0.01558599 | 0.96713357 | -3.815278 | 2.41846871 |
| GRTP1-AS1 | 1.39288992 | -5.7234685 | 2.46983932 | 0.0155922 | 0.96713357 | -3.8442232 | 2.41832378 |
| RAB28P5 | -0.5185003 | -0.9720005 | -2.4655669 | 0.01563856 | 0.96713357 | -3.7868423 | -2.4172433 |
| EIF3D | 0.22105625 | 6.36165578 | 2.46917043 | 0.01569252 | 0.96713357 | -3.8101582 | 2.41598943 |
| MDFIC2 | -1.1182153 | -6.5197868 | -2.4688913 | 0.01570433 | 0.96713357 | -3.8179926 | -2.4157154 |
| IRF4 | -0.8900065 | -1.6625553 | -2.4653973 | 0.0157666 | 0.96713357 | -3.8184374 | -2.4142739 |
| HMGN3-AS1 | 0.2739085 | 1.47473771 | 2.46399726 | 0.01587583 | 0.96713357 | -3.8048311 | 2.41175761 |
| LNMICC | 0.39689637 | 0.70593801 | 2.46401426 | 0.01590262 | 0.96713357 | -3.785753 | 2.41114278 |
| VPS13C-DT | -0.7748345 | -1.7152642 | -2.4628592 | 0.01590965 | 0.96713357 | -3.8073053 | -2.4109815 |
| BICRA-AS2 | -0.7717443 | -0.9738707 | -2.4562146 | 0.0159942 | 0.96713357 | -3.8651549 | -2.4090479 |
| TFIP11 | 0.10746629 | 5.11167337 | 2.45891307 | 0.0160441 | 0.96713357 | -3.8278435 | 2.40791078 |
| PSKH1 | 0.25556969 | 5.46526313 | 2.46016063 | 0.01606645 | 0.96713357 | -3.807597 | 2.40740252 |
| HCFC2 | -0.17898 | 5.22842855 | -2.4599073 | 0.01608728 | 0.96713357 | -3.8296243 | -2.4069293 |
| LINC02923 | -1.3900802 | -4.0582364 | -2.4533134 | 0.01611519 | 0.96713357 | -3.9061854 | -2.4062963 |
| AOC2 | -0.3124984 | 2.40597775 | -2.4576094 | 0.01615175 | 0.96713357 | -3.9001446 | -2.4054683 |
| XKR8 | 0.51726865 | 2.56187739 | 2.4559248 | 0.01621049 | 0.96713357 | -3.84229 | 2.4041416 |
| GPD2 | 0.24811244 | 4.7617978 | 2.45573403 | 0.01622886 | 0.96713357 | -3.794043 | 2.40372767 |
| ARFGEF1-DT | -0.3704921 | 0.60650608 | -2.4603155 | 0.01623133 | 0.96713357 | -3.8650406 | -2.403672 |
| MTCO1P43 | -1.4294171 | -5.9630836 | -2.4529505 | 0.01625764 | 0.96713357 | -3.8264978 | -2.4030797 |
| H4C12 | -1.4216382 | -3.4891183 | -2.4536252 | 0.01632029 | 0.96713357 | -3.8025727 | -2.401673 |
| CYCSP27 | -1.4503912 | -6.1822206 | -2.4488364 | 0.01639138 | 0.96713357 | -3.7978822 | -2.4000823 |
| IL32 | 1.20212634 | -0.9362115 | 2.452972 | 0.01643955 | 0.96713357 | -3.7655485 | 2.39900811 |
| PABPC1P4 | -1.3633008 | -3.6512965 | -2.4454898 | 0.01658607 | 0.96713357 | -3.7820447 | -2.3957573 |
| GASAL1 | 0.40775083 | -0.3818419 | 2.44586591 | 0.01664888 | 0.96713357 | -3.859494 | 2.39437141 |
| TPO | -0.8829998 | -2.2111938 | -2.4451681 | 0.01669939 | 0.96713357 | -3.8650803 | -2.3932603 |
| ITPRIP-AS1 | -1.3401692 | -4.6424345 | -2.4392538 | 0.01686202 | 0.96713357 | -3.8055868 | -2.3897026 |
| LINC02733 | -1.4005838 | -4.9769794 | -2.4396366 | 0.01686494 | 0.96713357 | -3.7444667 | -2.389639 |
| SLC25A40 | -0.1593529 | 4.54448786 | -2.4376615 | 0.01705311 | 0.96713357 | -3.8482177 | -2.3855607 |
| ERICH2 | -0.3523863 | 2.81780584 | -2.4340055 | 0.01713646 | 0.96713357 | -3.825034 | -2.3837669 |
| SDR42E1 | -1.3721571 | -5.8503918 | -2.4322426 | 0.01718883 | 0.96713357 | -3.817735 | -2.3826436 |
| PRX | 0.43355984 | 3.29950765 | 2.43259688 | 0.01723596 | 0.96713357 | -3.8624773 | 2.38163516 |
| LINC02069 | -1.5234666 | -5.4771532 | -2.4282307 | 0.01730198 | 0.96713357 | -3.8337951 | -2.3802269 |
| PARP11 | 0.15576555 | 4.69546102 | 2.42939871 | 0.0173887 | 0.96713357 | -3.8559079 | 2.37838409 |
| RBM45 | -0.1481573 | 3.78424254 | -2.4253625 | 0.01743817 | 0.96713357 | -3.8517229 | -2.3773366 |
| MIR4326 | -1.5134365 | -5.5450702 | -2.4229442 | 0.01751361 | 0.96713357 | -3.8594188 | -2.3757439 |
| CALHM2 | 0.39854729 | 1.85982995 | 2.4252066 | 0.01755864 | 0.96713357 | -3.9273187 | 2.37479624 |
| WNT10A | 1.1659961 | -0.9747805 | 2.42087834 | 0.01773545 | 0.96713357 | -4.0264551 | 2.37109552 |
| HSPD1P12 | -1.561077 | -4.9312175 | -2.4193047 | 0.01781967 | 0.96713357 | -3.8492916 | -2.3693441 |
| KCTD11 | 0.23775696 | 3.98061546 | 2.41251409 | 0.01813961 | 0.96713357 | -3.8538616 | 2.362756 |
| RPL9P21 | -1.5064619 | -5.6731593 | -2.4100583 | 0.01815854 | 0.96713357 | -3.7910706 | -2.3623695 |
| MIR609 | -1.1695374 | -6.4253182 | -2.4115852 | 0.0181974 | 0.96713357 | -3.8552341 | -2.3615768 |
| ADAM3B | -1.2852251 | -6.1097664 | -2.4072623 | 0.01829217 | 0.96713357 | -3.8134969 | -2.3596503 |
| LINC02928 | -1.2096986 | -3.424668 | -2.4078972 | 0.01830916 | 0.96713357 | -3.9365778 | -2.3593059 |
| LINC02485 | -1.1765533 | -6.6484653 | -2.4022889 | 0.01855999 | 0.96713357 | -3.8302646 | -2.3542527 |
| CISTR | -1.1551907 | -2.9790948 | -2.4015138 | 0.01856881 | 0.96713357 | -3.8949278 | -2.354076 |
| RNU1-94P | -1.0410527 | -3.0477524 | -2.4023402 | 0.01860046 | 0.96713357 | -3.8889246 | -2.353443 |
| CPEB1-AS1 | -0.7043618 | -1.5957702 | -2.4012472 | 0.01869947 | 0.96713357 | -3.861168 | -2.3514687 |
| ITGA6-AS1 | -1.3793494 | -5.1207439 | -2.398083 | 0.01873308 | 0.96713357 | -3.844376 | -2.3508004 |
| HMGB1P37 | -1.0582124 | -2.4957744 | -2.3919506 | 0.01885177 | 0.96713357 | -3.8627417 | -2.3484493 |
| NUAK2 | 0.50772772 | 3.8400967 | 2.39739174 | 0.01888784 | 0.96713357 | -3.8384008 | 2.34773719 |
| HPAT5 | 2.94255259 | -0.9122371 | 2.39289775 | 0.01894211 | 0.96713357 | -4.019929 | 2.34666833 |
| RN7SKP275 | -1.0697947 | -2.3143187 | -2.392409 | 0.01908773 | 0.96713357 | -3.9230149 | -2.3438131 |
| MYH2 | -1.5588544 | -4.3512365 | -2.3906438 | 0.01914358 | 0.96713357 | -3.7312207 | -2.3427231 |
| CDIPTOSP | -0.8711399 | -2.0172265 | -2.3937812 | 0.01914722 | 0.96713357 | -3.9160615 | -2.3426523 |
| EYS | 0.80019377 | -0.147691 | 2.39073825 | 0.01914876 | 0.96713357 | -3.9280016 | 2.34262232 |
| CTDP1-DT | -1.4177919 | -4.6651495 | -2.3896049 | 0.01915089 | 0.96713357 | -3.8615775 | -2.3425808 |
| RCAN2-DT | -1.1663695 | -5.0178463 | -2.3904958 | 0.01915161 | 0.96713357 | -3.8228403 | -2.3425667 |
| CHI3L1 | 2.00015643 | 1.58672863 | 2.39001079 | 0.01921065 | 0.96713357 | -3.7720743 | 2.34141799 |
| RPS3AP38 | -0.632453 | -0.871277 | -2.3886294 | 0.01921613 | 0.96713357 | -3.9060232 | -2.3413114 |
| AK4P1 | 0.55897563 | -1.1922153 | 2.38985183 | 0.01926415 | 0.96713357 | -3.8944373 | 2.34037963 |
| SH3YL1 | -0.1717653 | 5.73354197 | -2.3869456 | 0.01935008 | 0.96713357 | -3.8816871 | -2.3387171 |
| SELENOKP1 | -0.7576317 | -1.8566381 | -2.3802672 | 0.01944658 | 0.96713357 | -3.8442591 | -2.3368578 |
| ASS1 | 0.80267842 | 4.95537136 | 2.38415612 | 0.01949458 | 0.96713357 | -3.84115 | 2.33593598 |
| PTPN6 | -0.6667086 | 0.09189333 | -2.3804064 | 0.01966656 | 0.96713357 | -3.9830328 | -2.3326492 |
| RPL21P94 | -1.2805711 | -4.8083201 | -2.3790037 | 0.0196826 | 0.96713357 | -3.8964991 | -2.3323441 |
| MUL1 | 0.19918118 | 5.45801589 | 2.38024008 | 0.01968769 | 0.96713357 | -3.8828634 | 2.33224717 |
| ACTL7A | -1.0921136 | -6.4623904 | -2.3707787 | 0.01989698 | 0.96713357 | -3.8844564 | -2.3282849 |
| MGMT | 0.39055305 | 2.67266504 | 2.37513191 | 0.01990905 | 0.96713357 | -3.8606849 | 2.32805745 |
| HLA-DRB5 | 1.57821403 | -3.3725919 | 2.37228045 | 0.02007613 | 0.96713357 | -3.8989598 | 2.32492194 |
| TPST2 | 0.48458117 | 4.62544843 | 2.37148062 | 0.02011952 | 0.96713357 | -3.86638 | 2.32411156 |
| GEMIN8 | -0.1867427 | 4.53315952 | -2.3716891 | 0.02014469 | 0.96713357 | -3.8881092 | -2.3236421 |
| HSPE1P2 | -1.3858549 | -4.3130322 | -2.3650795 | 0.02044223 | 0.96713357 | -3.8316392 | -2.3181306 |
| TMEM104 | 0.22846956 | 5.65382172 | 2.36490608 | 0.02047202 | 0.96713357 | -3.8875843 | 2.31758254 |
| DERL3 | -0.5247951 | -0.4540826 | -2.356881 | 0.02087154 | 0.96713357 | -3.8766824 | -2.3103002 |
| ICAM1 | 1.77533515 | 0.84598631 | 2.35863284 | 0.02093019 | 0.96713357 | -3.8829112 | 2.30924138 |
| NRSN2-AS1 | -0.2125529 | 3.617381 | -2.3529672 | 0.02113265 | 0.96713357 | -3.8757141 | -2.3056061 |
| NTMT2 | -0.8964236 | -1.6356996 | -2.3523572 | 0.02116524 | 0.96713357 | -3.8298152 | -2.3050238 |
| SMIM27 | 0.22021303 | 1.56515309 | 2.35072292 | 0.02123615 | 0.96713357 | -3.9210227 | 2.30375943 |
| LRRC63 | -0.9715292 | -2.0421223 | -2.348418 | 0.0213257 | 0.96713357 | -3.8619251 | -2.302168 |
| PIEZO2 | 0.96659387 | 4.86756445 | 2.3479512 | 0.02134678 | 0.96713357 | -3.9211526 | 2.30179418 |
| ANKRD44-DT | -1.2057714 | -5.8854066 | -2.3464476 | 0.02139065 | 0.96713357 | -3.8877602 | -2.3010174 |
| ARID5B | 0.36839966 | 5.33354152 | 2.34649717 | 0.0214115 | 0.96713357 | -3.8936515 | 2.30064867 |
| ACVRL1 | 1.30885782 | -2.7219264 | 2.346004 | 0.02148219 | 0.96713357 | -4.0013503 | 2.29940073 |
| PIK3AP1 | 0.47566455 | 0.93319106 | 2.34510081 | 0.02149542 | 0.96713357 | -3.9375221 | 2.29916772 |
| GBP2 | 1.5676175 | 0.97009987 | 2.34554162 | 0.0215479 | 0.96713357 | -3.9024485 | 2.29824408 |
| GREM1 | 2.28121003 | -0.0656707 | 2.34241633 | 0.02158267 | 0.96713357 | -4.054853 | 2.29763319 |
| LOXL4 | 0.70380397 | -0.2054548 | 2.34385435 | 0.02163233 | 0.96713357 | -3.9329181 | 2.29676243 |
| CHORDC2P | -1.2649888 | -6.0508243 | -2.3405439 | 0.02166379 | 0.96713357 | -3.8818429 | -2.2962116 |
| TM4SF18 | 1.24156156 | -4.768799 | 2.34142852 | 0.02170265 | 0.96713357 | -3.881494 | 2.29553204 |
| GALNT15 | 0.32264426 | 3.46226151 | 2.34100372 | 0.02173894 | 0.96713357 | -3.9083476 | 2.29489848 |
| POLR2J2 | -0.5600689 | -0.7501274 | -2.3414533 | 0.02175936 | 0.96713357 | -3.8577791 | -2.2945424 |
| EIF3LP3 | -1.0724534 | -3.0580834 | -2.3394047 | 0.02182579 | 0.96713357 | -3.9143101 | -2.2933861 |
| NOP53 | 0.30231109 | 7.30255723 | 2.33618219 | 0.02198825 | 0.96713357 | -3.9109798 | 2.29057075 |
| USP7-AS1 | -1.3319496 | -4.2522055 | -2.3336265 | 0.02203156 | 0.96713357 | -3.8948909 | -2.2898234 |
| ARPC3P4 | -1.3061342 | -4.5432951 | -2.3356439 | 0.0220872 | 0.96713357 | -3.9302758 | -2.2888649 |
| AHCYP3 | -1.2901913 | -5.3863113 | -2.3320961 | 0.02214489 | 0.96713357 | -3.8101017 | -2.2878735 |
| IL1R1 | 1.14153961 | 2.69330357 | 2.33260089 | 0.02221339 | 0.96713357 | -3.9425432 | 2.28669918 |
| ANKRD23 | -0.3704743 | 1.28745826 | -2.3385754 | 0.02221983 | 0.96713357 | -3.9799449 | -2.2865888 |
| RPS12P31 | -1.3695549 | -4.5993834 | -2.3298153 | 0.02228285 | 0.96713357 | -3.9396472 | -2.2855116 |
| LINC02384 | 0.82623042 | -1.7622222 | 2.32948249 | 0.02230345 | 0.96713357 | -3.9974558 | 2.28515994 |
| SIRPB1 | -1.0720244 | -3.897464 | -2.3283198 | 0.02237898 | 0.96713357 | -3.9434139 | -2.2838733 |
| CICP5 | 1.31343008 | -4.9361363 | 2.32643468 | 0.02244399 | 0.96713357 | -3.9746161 | 2.28276884 |
| RN7SL416P | -1.2164598 | -6.4432299 | -2.3252387 | 0.02247955 | 0.96713357 | -3.8818575 | -2.2821659 |
| MCAM | 0.40017861 | 5.54066708 | 2.32767279 | 0.02250399 | 0.96713357 | -3.9212384 | 2.28175193 |
| NFKBIA | 0.50910203 | 4.78158344 | 2.32795082 | 0.02251916 | 0.96713357 | -3.9314885 | 2.28149527 |
| THEMIS | 1.81181189 | -5.6403275 | 2.32223229 | 0.02271345 | 0.96713357 | -3.9603054 | 2.27822032 |
| EEF1A1P30 | -1.2518177 | -4.8898338 | -2.3208356 | 0.02282709 | 0.96713357 | -3.8946945 | -2.2763163 |
| LYPLAL1-DT | -0.405323 | -0.4097677 | -2.3199435 | 0.02286508 | 0.96713357 | -3.8826272 | -2.2756814 |
| HLA-DRB1 | 1.32760353 | -0.8308068 | 2.31975676 | 0.0229245 | 0.96713357 | -3.9405469 | 2.27469063 |
| AP1AR-DT | 0.34098073 | 1.52509534 | 2.31893107 | 0.02303373 | 0.96713357 | -3.9633218 | 2.27287472 |
| TRAF7 | 0.1932076 | 6.90197515 | 2.31529458 | 0.02310217 | 0.96713357 | -3.9258954 | 2.27174089 |
| MYL6B-AS1 | 1.11801175 | -3.3023874 | 2.31391346 | 0.02311671 | 0.96713357 | -3.9962962 | 2.27150033 |
| ZFP36L2 | 0.38859251 | 6.51256085 | 2.31588059 | 0.02313328 | 0.96713357 | -3.9149509 | 2.27122628 |
| LINC00443 | -1.2273822 | -5.5940557 | -2.3148882 | 0.02315598 | 0.96713357 | -3.869008 | -2.2708513 |
| LIPT2 | 0.25937272 | 1.47047148 | 2.31760209 | 0.02315817 | 0.96713357 | -3.9749353 | 2.27081517 |
| LINC02775 | 1.34548405 | -6.2422309 | 2.31143263 | 0.02329946 | 0.96713357 | -3.9379276 | 2.26848833 |
| LINC01353 | -1.2714446 | -3.7182537 | -2.3091615 | 0.02340794 | 0.96713357 | -3.8739219 | -2.2667102 |
| CCL2 | 1.05125744 | 3.69980997 | 2.31246227 | 0.02347075 | 0.96713357 | -3.8863211 | 2.26568377 |
| MRPL3P1 | -1.2666826 | -4.3651089 | -2.3045456 | 0.02350645 | 0.96713357 | -3.9517488 | -2.2651016 |
| ZNF706 | -0.1228089 | 6.24007476 | -2.3082027 | 0.02358209 | 0.96713357 | -3.9312809 | -2.2638704 |
| TRIM25 | 0.22133713 | 4.83765833 | 2.3083444 | 0.02358309 | 0.96713357 | -3.9027546 | 2.26385409 |
| TBXA2R | -1.3889093 | -4.2369253 | -2.3076008 | 0.02360237 | 0.96713357 | -3.894468 | -2.2635408 |
| DNAJB5-DT | -1.277261 | -4.3667374 | -2.3054247 | 0.02366516 | 0.96713357 | -3.9330477 | -2.2625222 |
| EIF2AK4 | 0.19968422 | 6.05052107 | 2.3063288 | 0.02366908 | 0.96713357 | -3.9349786 | 2.26245867 |
| CSPG4BP | 1.39680369 | -5.5047169 | 2.30679148 | 0.02368988 | 0.96713357 | -3.933629 | 2.26212184 |
| PSEN1 | 0.15085068 | 6.80505602 | 2.3063189 | 0.02371659 | 0.96713357 | -3.9356791 | 2.26168964 |
| CD276 | 0.24501817 | 7.19344182 | 2.306681 | 0.02372693 | 0.96713357 | -3.9301832 | 2.2615224 |
| LINC01970 | 0.7719692 | -1.6921242 | 2.30552314 | 0.02376003 | 0.96713357 | -4.0086262 | 2.26098757 |
| PIGY-DT | -1.0397338 | -3.8992108 | -2.2996423 | 0.02402004 | 0.96713357 | -3.9830034 | -2.2568086 |
| LGALS14 | -1.1872119 | -6.3259771 | -2.2962169 | 0.02424519 | 0.96713357 | -3.9207917 | -2.2532214 |
| PSMC1P5 | -1.4027395 | -5.8483798 | -2.2954956 | 0.02426509 | 0.96713357 | -3.9030428 | -2.2529058 |
| VTCN1 | -0.3799454 | -0.4087285 | -2.2954142 | 0.02436336 | 0.96713357 | -3.9370873 | -2.2513503 |
| C1S | 1.00055393 | 5.66827481 | 2.29582954 | 0.02437083 | 0.96713357 | -3.9793001 | 2.2512322 |
| SLC25A1P1 | -1.4444821 | -6.1244002 | -2.2899935 | 0.02437294 | 0.96713357 | -3.9275217 | -2.251199 |
| LINC02295 | -1.3948778 | -5.6447925 | -2.2939045 | 0.02438667 | 0.96713357 | -3.9708686 | -2.2509821 |
| LRIT2 | 1.24659213 | -3.3312307 | 2.29294551 | 0.02450047 | 0.96713357 | -3.9703651 | 2.24918914 |
| CA9 | 1.40770609 | -2.578882 | 2.29059946 | 0.02455739 | 0.96713357 | -4.0303683 | 2.24829492 |
| BACE2 | 0.66312854 | 3.77721876 | 2.29221705 | 0.02457041 | 0.96713357 | -3.8852731 | 2.24809071 |
| DRAM1 | 0.53120338 | 3.32569767 | 2.29108431 | 0.02461677 | 0.96713357 | -3.9392179 | 2.24736405 |
| EPHA1 | -0.8122575 | -1.0238792 | -2.285639 | 0.02476494 | 0.96713357 | -3.9861593 | -2.2450498 |
| SNORD17 | -0.966547 | -3.0911111 | -2.2926575 | 0.02482786 | 0.96713357 | -3.9632682 | -2.2440706 |
| TNKS1BP1 | 0.29806763 | 6.94012012 | 2.28774987 | 0.02483436 | 0.96713357 | -3.9486082 | 2.24396956 |
| LINC02389 | -0.9401472 | -3.2815198 | -2.2833384 | 0.02497358 | 0.96713357 | -3.9593605 | -2.2418112 |
| RIPK4 | 0.83222054 | -2.4864468 | 2.28309675 | 0.02504277 | 0.96713357 | -3.9908162 | 2.24074236 |
| SOCS5P2 | -1.030217 | -3.1390071 | -2.2776543 | 0.02528933 | 0.96713357 | -3.9327094 | -2.2369542 |
| RN7SL239P | -1.2353554 | -5.1334117 | -2.2774027 | 0.02530138 | 0.96713357 | -3.9336258 | -2.23677 |
| ETV7 | 1.08562898 | -1.646434 | 2.27898012 | 0.02536958 | 0.96713357 | -4.0364627 | 2.23572818 |
| ACTL8 | 1.47989103 | -5.709197 | 2.27641009 | 0.02537664 | 0.96713357 | -3.9652049 | 2.23562045 |
| UBE2CP2 | 1.30431694 | -3.9211892 | 2.2774703 | 0.0254012 | 0.96713357 | -4.0155596 | 2.23524602 |
| ZNF217 | 0.40231461 | 5.070106 | 2.27490327 | 0.02561226 | 0.96713357 | -3.9516224 | 2.23204085 |
| NAV2-AS5 | 1.39578992 | -5.7074714 | 2.27366853 | 0.0256197 | 0.96713357 | -3.9768449 | 2.23192837 |
| HNRNPRP1 | -1.3943374 | -4.8273321 | -2.2727097 | 0.02567308 | 0.96713357 | -3.923661 | -2.2311215 |
| RARRES2 | 1.38592715 | 0.00962807 | 2.27145272 | 0.02584203 | 0.96713357 | -3.9920965 | 2.22857748 |
| HLA-H | 0.72953341 | 1.36974075 | 2.26874926 | 0.0260343 | 0.96713357 | -3.9489786 | 2.22569969 |
| GLIDR | 0.17746761 | 2.65583954 | 2.26695356 | 0.02609055 | 0.96713357 | -3.9799005 | 2.22486131 |
| SHKBP1 | 0.23540997 | 4.54562655 | 2.26571351 | 0.02619741 | 0.96713357 | -3.9535946 | 2.22327287 |
| CMC2 | -0.1932431 | 4.10746902 | -2.2638637 | 0.02622898 | 0.96713357 | -3.978689 | -2.2228046 |
| H2AZP1 | -0.9941266 | -6.3243489 | -2.2641894 | 0.02626582 | 0.96713357 | -3.9191537 | -2.2222588 |
| NEB | -0.3002946 | 2.30818491 | -2.2645255 | 0.02626974 | 0.96713357 | -3.9840214 | -2.2222008 |
| TYW1 | 0.15628172 | 4.74698506 | 2.26217662 | 0.02643678 | 0.96713357 | -3.9713507 | 2.2197347 |
| RNF32 | -0.2649704 | 1.03901926 | -2.261114 | 0.02648078 | 0.96713357 | -3.9691493 | -2.2190873 |
| TP53 | 0.33867857 | 5.59534137 | 2.26042514 | 0.0265577 | 0.96713357 | -3.9623743 | 2.21795789 |
| TASP1 | 0.15902507 | 3.15057437 | 2.25762333 | 0.02669942 | 0.96713357 | -3.9801683 | 2.21588441 |
| TNFAIP8L3 | 0.80373887 | 0.76536251 | 2.25442108 | 0.02684246 | 0.96713357 | -3.9284145 | 2.21380113 |
| RNA5SP490 | -1.0773115 | -4.1457735 | -2.2535058 | 0.02686632 | 0.96713357 | -3.8906721 | -2.2134546 |
| FNDC8 | 0.87855272 | -2.3409946 | 2.2520573 | 0.02691728 | 0.96713357 | -4.0537994 | 2.21271531 |
| ZNF248-AS1 | 0.51248893 | -1.4179146 | 2.25316084 | 0.02693817 | 0.96713357 | -4.0103499 | 2.21241264 |
| CLDN19 | 1.53377121 | -3.4801625 | 2.2520719 | 0.02703312 | 0.96713357 | -4.0579446 | 2.21103926 |
| LINC02224 | 1.41031965 | -5.8601795 | 2.25279281 | 0.02703524 | 0.96713357 | -3.9683468 | 2.21100866 |
| CAV2 | 0.76045865 | 2.98085813 | 2.25275144 | 0.02704402 | 0.96713357 | -3.9385737 | 2.21088192 |
| CCDC153 | -0.4220452 | 0.46576934 | -2.252261 | 0.02705361 | 0.96713357 | -3.9609808 | -2.2107434 |
| CROCCP1 | 1.37854119 | -4.8099084 | 2.25027046 | 0.0270799 | 0.96713357 | -4.0224961 | 2.21036414 |
| SND1-DT | -1.3495008 | -5.2352549 | -2.2467545 | 0.02711578 | 0.96713357 | -3.9630384 | -2.2098471 |
| HOXD8 | -1.0481577 | -4.1830075 | -2.2507324 | 0.02714204 | 0.96713357 | -3.885952 | -2.209469 |
| NFKB2 | 0.44163377 | 4.05047895 | 2.25080603 | 0.02718521 | 0.96713357 | -3.9426798 | 2.208848 |
| ACTG1P22 | 1.19128629 | -6.5906703 | 2.24942002 | 0.02721902 | 0.96713357 | -3.9804315 | 2.20836237 |
| RNF145 | 0.29080855 | 7.35025473 | 2.24962824 | 0.0272528 | 0.96713357 | -3.970878 | 2.2078777 |
| LCN2 | 2.04144081 | -4.2713043 | 2.24820432 | 0.02736796 | 0.96713357 | -3.9895361 | 2.20622908 |
| BET1L | 0.13465845 | 4.80289839 | 2.24798017 | 0.0274002 | 0.96713357 | -3.9881195 | 2.20576873 |
| SNORD113-3 | -1.0389442 | -4.8410684 | -2.2458374 | 0.02743401 | 0.96713357 | -3.9368932 | -2.2052863 |
| KIAA1217 | 0.53183892 | 4.24703741 | 2.2453138 | 0.02752819 | 0.96713357 | -3.9785957 | 2.20394533 |
| SLC11A2 | 0.24214077 | 6.75353592 | 2.24608192 | 0.02756561 | 0.96713357 | -3.976739 | 2.20341362 |
| MRC2 | 0.62431268 | 7.84464633 | 2.24416874 | 0.0276137 | 0.96713357 | -3.9758393 | 2.20273118 |
| RN7SL622P | -1.2171416 | -5.7101983 | -2.2397193 | 0.02780206 | 0.96713357 | -3.9968705 | -2.2000682 |
| SMIM47 | 0.77380445 | -2.2241348 | 2.23984843 | 0.02797219 | 0.96713357 | -4.0473067 | 2.19767616 |
| EIF3A | 0.16175517 | 8.13317916 | 2.23404681 | 0.02828766 | 0.96713357 | -3.982154 | 2.19327381 |
| BLTP2 | 0.13167872 | 7.40540777 | 2.23295457 | 0.0283328 | 0.96713357 | -3.9853965 | 2.19264731 |
| PSME2P1 | -0.7202167 | -1.7751782 | -2.2328642 | 0.02836361 | 0.96713357 | -3.9810906 | -2.1922203 |
| BIRC3 | 0.85473038 | 0.09255669 | 2.23114999 | 0.02848172 | 0.96713357 | -4.0007095 | 2.19058657 |
| MARCHF10 | -0.5929739 | -0.6565923 | -2.2297384 | 0.02860658 | 0.96713357 | -4.0112678 | -2.1888661 |
| YAE1-DT | -1.0344158 | -2.7950994 | -2.2252435 | 0.02860706 | 0.96713357 | -4.0260575 | -2.1888593 |
| LINC02074 | -1.2275437 | -5.5807051 | -2.2282455 | 0.02863139 | 0.96713357 | -3.9827982 | -2.1885249 |
| CEBPA-DT | -0.6096839 | 0.02117262 | -2.2292913 | 0.02863456 | 0.96713357 | -3.9612347 | -2.1884814 |
| MMP3 | 1.25588179 | -6.091954 | 2.22442627 | 0.02866412 | 0.96713357 | -3.9485235 | 2.18807529 |
| PSIP1P1 | -0.728304 | -2.276883 | -2.225501 | 0.02871911 | 0.96713357 | -3.9573069 | -2.1873209 |
| BMP1 | 0.29292982 | 6.13079449 | 2.22800823 | 0.0287226 | 0.96713357 | -3.9903574 | 2.18727298 |
| PTGFRN | 0.29672257 | 8.0922056 | 2.22625565 | 0.02884637 | 0.96713357 | -3.9913179 | 2.18557971 |
| EIF4BP6 | 0.28777913 | 0.86968618 | 2.22509837 | 0.0288907 | 0.96713357 | -3.9872407 | 2.18497474 |
| CKAP4 | 0.23031094 | 7.02080611 | 2.22528046 | 0.02890756 | 0.96713357 | -3.9902565 | 2.18474478 |
| MMP9 | -0.7548529 | -1.7248572 | -2.225068 | 0.02892242 | 0.96713357 | -4.026376 | -2.1845424 |
| CDIN1 | -0.1686986 | 3.99448522 | -2.2247202 | 0.02895656 | 0.96713357 | -4.0102931 | -2.1840774 |
| UQCRB-AS1 | -0.623184 | -2.0999659 | -2.2224258 | 0.02898113 | 0.96713357 | -4.0016605 | -2.1837431 |
| CRB1 | 0.38136812 | 5.31609719 | 2.22295182 | 0.02905485 | 0.96713357 | -3.9757102 | 2.18274153 |
| TPI1P3 | -1.2843145 | -6.3106868 | -2.2176311 | 0.02914057 | 0.96713357 | -3.9737777 | -2.1815797 |
| ABI3BP | -0.5472265 | 0.97892518 | -2.2212537 | 0.02917143 | 0.96713357 | -3.9090871 | -2.1811621 |
| CLDN6 | 0.79785075 | -0.4746391 | 2.21964904 | 0.02927604 | 0.96713357 | -4.0661012 | 2.17974945 |
| PLEKHA2 | 0.22889899 | 4.65374953 | 2.21975347 | 0.02929646 | 0.96713357 | -4.004579 | 2.17947422 |
| NEUROD6 | 1.23556936 | 1.62926574 | 2.21875469 | 0.02937571 | 0.96713357 | -4.0149421 | 2.17840758 |
| ALOX5AP | -1.2956645 | -5.493297 | -2.2160459 | 0.02939123 | 0.96713357 | -3.9641316 | -2.1781989 |
| GPBAR1 | 0.62533788 | -1.7469447 | 2.21706488 | 0.02941438 | 0.96713357 | -4.0464577 | 2.17788793 |
| LINC01191 | 1.44545331 | -4.1504713 | 2.21553842 | 0.02950087 | 0.96713357 | -4.0490387 | 2.176728 |
| ASB13 | 0.2254621 | 4.99584784 | 2.21551395 | 0.02955552 | 0.96713357 | -4.0083445 | 2.17599654 |
| CYCSP34 | 0.67871358 | -1.945372 | 2.21360654 | 0.02962857 | 0.96713357 | -4.0223549 | 2.17502062 |
| HMGN2P46 | 0.43148138 | -1.4411265 | 2.21443801 | 0.02972325 | 0.96713357 | -3.9744747 | 2.17375882 |
| DNAJA1P3 | -1.3393723 | -3.1334928 | -2.2070833 | 0.02986688 | 0.96713357 | -4.0078386 | -2.1718513 |
| LINC00923 | -1.1107669 | -4.0571387 | -2.2112373 | 0.02987151 | 0.96713357 | -4.0190437 | -2.17179 |
| TNFAIP2 | 1.21391474 | 2.93426949 | 2.21042325 | 0.02992232 | 0.96713357 | -4.0388672 | 2.17111718 |
| EPCAM-DT | -0.4028621 | 1.11106135 | -2.2074512 | 0.03012034 | 0.96713357 | -3.9613779 | -2.1685043 |
| AIMP2 | 0.20187195 | 3.9175104 | 2.20694373 | 0.03015481 | 0.96713357 | -4.004909 | 2.16805095 |
| IGF2BP2-AS1 | 1.54650467 | -5.7550543 | 2.20812199 | 0.03015707 | 0.96713357 | -4.0215574 | 2.16802127 |
| LINC01352 | 1.24184747 | -4.993124 | 2.20585474 | 0.03019831 | 0.96713357 | -4.016688 | 2.16747956 |
| IMPDH1P8 | 0.53774385 | -0.4614073 | 2.20341488 | 0.03032919 | 0.96713357 | -4.0539648 | 2.1657644 |
| TMEM249 | -0.7859933 | -0.1148145 | -2.2031807 | 0.03047882 | 0.96713357 | -4.0837457 | -2.1638115 |
| FAM13A | 0.14208871 | 4.57357222 | 2.20240568 | 0.03050204 | 0.96713357 | -4.0259145 | 2.16350922 |
| PHB1P4 | -1.3791354 | -3.4105684 | -2.1999709 | 0.03052959 | 0.96713357 | -3.9860254 | -2.1631507 |
| STEAP3 | 1.11825965 | 3.39845624 | 2.20149098 | 0.03060035 | 0.96713357 | -4.0219891 | 2.16223129 |
| C1QL3 | 0.70607497 | 1.62875544 | 2.20143028 | 0.03060699 | 0.96713357 | -4.0821118 | 2.16214512 |
| MIR3192 | -0.9307046 | -3.1101293 | -2.2003061 | 0.03063976 | 0.96713357 | -4.0309569 | -2.1617201 |
| PTGES3P2 | -1.2850679 | -5.8173156 | -2.1995562 | 0.03063982 | 0.96713357 | -4.0117395 | -2.1617193 |
| RPL21P134 | 1.49609015 | -4.8640587 | 2.19684681 | 0.03068816 | 0.96713357 | -4.0203931 | 2.16109293 |
| MIR589 | -1.1916679 | -5.0681376 | -2.1975158 | 0.03082194 | 0.96713357 | -3.9900342 | -2.159364 |
| RNU6-19P | 1.22195373 | -5.9878739 | 2.19867868 | 0.03082841 | 0.96713357 | -4.0119521 | 2.1592805 |
| LINC02717 | 1.44991706 | -4.4052431 | 2.1969935 | 0.03088515 | 0.96713357 | -4.0295099 | 2.15854936 |
| DNLZ | -0.4667157 | -0.6380752 | -2.1968529 | 0.03092772 | 0.96713357 | -4.0357486 | -2.1580014 |
| GPX5 | -1.1429459 | -5.4573819 | -2.1953936 | 0.0310019 | 0.96713357 | -4.0049733 | -2.1570484 |
| CYB5A | -0.1729852 | 4.54475616 | -2.1957529 | 0.03102003 | 0.96713357 | -4.0044973 | -2.1568156 |
| PARD3-DT | -0.7961611 | -1.5421823 | -2.1927805 | 0.03110658 | 0.96713357 | -4.0738126 | -2.1557067 |
| SHMT1 | 0.29589099 | 4.1646651 | 2.19477947 | 0.03111425 | 0.96713357 | -4.0133809 | 2.15560849 |
| TEDC2-AS1 | -0.6884493 | -0.9717114 | -2.1903956 | 0.03115241 | 0.96713357 | -4.003948 | -2.1551205 |
| SNORD14E | -1.3603239 | -3.9378301 | -2.1922523 | 0.03116005 | 0.96713357 | -4.0468807 | -2.1550228 |
| ALPK1 | 0.72102765 | 3.58527801 | 2.19400418 | 0.03120709 | 0.96713357 | -4.0469381 | 2.15442198 |
| TOB2P1 | 0.64710263 | -1.9174064 | 2.19104754 | 0.03132554 | 0.96713357 | -3.9916587 | 2.15291265 |
| HMGB3P24 | -0.5927315 | -1.4219034 | -2.1863757 | 0.03142732 | 0.96713357 | -4.0234073 | -2.1516197 |
| RNU6ATAC16P | 0.95969526 | -3.963212 | 2.18928903 | 0.03144721 | 0.96713357 | -4.0656682 | 2.15136745 |
| FCF1P8 | -1.2491044 | -6.1397792 | -2.1865545 | 0.03155259 | 0.96713357 | -4.0190986 | -2.1500332 |
| LINC00861 | -1.3200792 | -5.0144911 | -2.1860025 | 0.03169171 | 0.96713357 | -4.0341179 | -2.1482776 |
| LINC00299 | 0.94411844 | -1.921729 | 2.18755812 | 0.03171974 | 0.96713357 | -4.1309973 | 2.14792465 |
| AMHR2 | 1.34447697 | -5.9317742 | 2.18436356 | 0.03176387 | 0.96713357 | -4.0467515 | 2.1473696 |
| SLC2A8 | 0.19109252 | 5.20829938 | 2.18393166 | 0.03186434 | 0.96713357 | -4.0267746 | 2.14610835 |
| GYPC | 0.78944678 | 2.3831307 | 2.18182911 | 0.0320496 | 0.96713357 | -3.994804 | 2.14379148 |
| ZBED3 | 0.23052289 | 4.9354702 | 2.18111826 | 0.03216479 | 0.96713357 | -4.0236423 | 2.14235666 |
| FAM156A | 0.31469723 | 0.70654346 | 2.17968026 | 0.03226608 | 0.96713357 | -4.0343142 | 2.14109876 |
| CCND2 | 0.35664548 | 6.86249923 | 2.17977224 | 0.03228031 | 0.96713357 | -4.0216617 | 2.14092225 |
| ANGPT4 | -1.1999406 | -3.60682 | -2.1780104 | 0.03238978 | 0.96713357 | -4.0309377 | -2.139567 |
| PMS2P2 | -0.8144789 | -2.1673771 | -2.1771181 | 0.03241542 | 0.96713357 | -4.0413005 | -2.1392501 |
| SPX | -0.4942069 | 0.13908861 | -2.1764533 | 0.03246345 | 0.96713357 | -3.993409 | -2.1386571 |
| RNU6-1091P | 1.15660285 | -4.4199877 | 2.17523816 | 0.03246565 | 0.96713357 | -4.0819872 | 2.13862993 |
| VDAC2P5 | -0.3844608 | -0.982039 | -2.1747101 | 0.03248601 | 0.96713357 | -3.997615 | -2.1383788 |
| SERPING1 | 0.78864581 | 4.61375338 | 2.17622725 | 0.03252239 | 0.96713357 | -4.0211168 | 2.13793039 |
| CPD | 0.43357914 | 6.67871901 | 2.175185 | 0.03255679 | 0.96713357 | -4.0221986 | 2.13750678 |
| NAGA | 0.27515348 | 4.87808974 | 2.17541274 | 0.03256546 | 0.96713357 | -4.0109098 | 2.13740014 |
| MELTF-AS1 | -0.3463861 | 1.31867524 | -2.1741916 | 0.03260848 | 0.96713357 | -4.0312005 | -2.1368711 |
| BACH1 | 0.16345587 | 5.27192085 | 2.17233832 | 0.03277513 | 0.96713357 | -4.0284534 | 2.13482721 |
| LINC01886 | -1.2728757 | -5.5570706 | -2.1725049 | 0.03281948 | 0.96713357 | -4.0220057 | -2.1342848 |
| CRTAP | 0.26549702 | 6.91568834 | 2.17224706 | 0.03286287 | 0.96713357 | -4.0267772 | 2.13375469 |
| MT1A | -1.3175801 | -5.6771056 | -2.1698476 | 0.03294403 | 0.96713357 | -4.01986 | -2.1327648 |
| MEI1 | -1.0870451 | -2.2979227 | -2.1688665 | 0.03300828 | 0.96713357 | -4.066357 | -2.1319826 |
| RPL12P17 | 1.20350967 | -3.9338072 | 2.16841209 | 0.03301799 | 0.96713357 | -4.0294657 | 2.13186446 |
| PPP1R14BP2 | -1.2393932 | -4.1152194 | -2.1666622 | 0.03315301 | 0.96713357 | -4.0024311 | -2.1302254 |
| SLC35A2 | 0.20473436 | 4.88811215 | 2.16773937 | 0.03317654 | 0.96713357 | -4.0410253 | 2.12994029 |
| PGRMC2 | 0.22895009 | 6.33813593 | 2.16671837 | 0.03323507 | 0.96713357 | -4.0310461 | 2.12923202 |
| TYW1B | 0.24930659 | 1.39104043 | 2.16658642 | 0.03325391 | 0.96713357 | -4.0364018 | 2.12900421 |
| GAPDHP60 | 1.3731592 | -5.7056453 | 2.16241069 | 0.03325407 | 0.96713357 | -4.032137 | 2.12900232 |
| FGF7P8 | -0.7689067 | -2.862039 | -2.1655635 | 0.03329256 | 0.96713357 | -4.0348535 | -2.1285373 |
| HELZ2 | 0.4147617 | 4.44605604 | 2.16651326 | 0.03329726 | 0.96713357 | -3.9968234 | 2.12848051 |
| LINC00319 | -1.2855636 | -5.0638141 | -2.1654229 | 0.03334917 | 0.96713357 | -4.0469476 | -2.1278542 |
| HEXIM2 | -0.3770508 | 0.03249496 | -2.1624187 | 0.0333549 | 0.96713357 | -4.0394157 | -2.1277851 |
| PRSS23 | 0.48854882 | 5.00692834 | 2.16393905 | 0.03346674 | 0.96713357 | -4.0121749 | 2.12643876 |
| NPLOC4 | 0.11268317 | 7.23089928 | 2.16234247 | 0.0336147 | 0.96713357 | -4.0346797 | 2.12466352 |
| KRT77 | -1.118612 | -5.6255789 | -2.1619298 | 0.03363198 | 0.96713357 | -4.0227123 | -2.1244566 |
| FA2H | 0.96427581 | -1.6176331 | 2.16335492 | 0.03364415 | 0.96713357 | -4.1564497 | 2.12431095 |
| MIR3180-4 | -1.0113552 | -4.1409642 | -2.1620048 | 0.03364915 | 0.96713357 | -4.0470124 | -2.1242511 |
| MIR4453HG | -0.1647188 | 3.65111175 | -2.1614514 | 0.03367595 | 0.96713357 | -4.0533074 | -2.1239306 |
| MAGEE2 | -0.8186569 | -1.0223248 | -2.159426 | 0.03376862 | 0.96713357 | -4.0545556 | -2.1228238 |
| BVES-AS1 | -0.6833899 | -1.063756 | -2.1602208 | 0.03378328 | 0.96713357 | -4.0216432 | -2.1226489 |
| IL13RA1 | 0.66450052 | 4.64825419 | 2.16018692 | 0.0337902 | 0.96713357 | -4.0320322 | 2.12256641 |
| GJC3 | -0.9609861 | -3.0625995 | -2.1549383 | 0.03388258 | 0.96713357 | -4.0603227 | -2.1214663 |
| POC5 | -0.1517975 | 3.60542916 | -2.1563626 | 0.0339705 | 0.96713357 | -4.041028 | -2.1204217 |
| SBSPON | 0.6741618 | 2.06631766 | 2.15877933 | 0.03397705 | 0.96713357 | -4.0571676 | 2.12034394 |
| EIF3B | 0.14031916 | 7.11019159 | 2.15756896 | 0.03400166 | 0.96713357 | -4.038281 | 2.12005203 |
| HAGLR | -1.2032604 | -5.3308164 | -2.1570313 | 0.0340167 | 0.96713357 | -4.0085626 | -2.1198737 |
| RPL5P12 | -1.3190629 | -5.2792715 | -2.1506805 | 0.03419709 | 0.96713357 | -4.0134739 | -2.11774 |
| RPS7P14 | -0.8202465 | -2.8117303 | -2.154404 | 0.03427152 | 0.96713357 | -4.0334503 | -2.1168624 |
| LINC01219 | -1.128732 | -5.9006883 | -2.1523428 | 0.03432784 | 0.96713357 | -4.0497511 | -2.1161996 |
| CXCL6 | 1.15948145 | 0.23714581 | 2.15340273 | 0.03438774 | 0.96713357 | -4.0143975 | 2.11549551 |
| TLCD3A | 0.18703264 | 4.76558041 | 2.15091091 | 0.034548 | 0.96713357 | -4.0306354 | 2.11361691 |
| SLC52A1 | -1.2551484 | -3.5372281 | -2.1480123 | 0.03461805 | 0.96713357 | -4.1063136 | -2.1127981 |
| NPRL2 | -0.1781081 | 4.88694463 | -2.1493446 | 0.03461862 | 0.96713357 | -4.0545804 | -2.1127915 |
| ESYT3 | -0.5257006 | 1.75848765 | -2.1487352 | 0.03469057 | 0.96713357 | -4.0642983 | -2.1119519 |
| TIAM1-AS1 | -0.6682295 | -0.9167798 | -2.1478441 | 0.03469293 | 0.96713357 | -4.0526238 | -2.1119244 |
| PHF10P1 | -1.1525188 | -5.1148365 | -2.1452417 | 0.03476342 | 0.96713357 | -4.0228484 | -2.1111035 |
| CLP1 | 0.18255526 | 3.08851153 | 2.14701352 | 0.0348033 | 0.96713357 | -4.0604836 | 2.11063959 |
| MIR548XHG | -1.1080754 | -6.3587153 | -2.1458163 | 0.03480423 | 0.96713357 | -4.0063531 | -2.1106288 |
| C5orf47 | -0.8923436 | -2.8809914 | -2.1456058 | 0.03482328 | 0.96713357 | -4.0101069 | -2.1104074 |
| FMN1 | 0.78584557 | -0.4820185 | 2.14695324 | 0.03482394 | 0.96713357 | -4.0662981 | 2.11039973 |
| RN7SL268P | -0.4972255 | 0.23994035 | -2.1469745 | 0.03485972 | 0.96713357 | -4.0614285 | -2.1099841 |
| CXCL2 | 1.10810351 | 1.36773817 | 2.15449633 | 0.03496558 | 0.96713357 | -4.0065507 | 2.10875674 |
| HSD17B7P2 | 1.24783687 | -6.1400709 | 2.14302388 | 0.03506968 | 0.96713357 | -4.0633188 | 2.10755291 |
| SRP72P2 | -1.2519192 | -3.3265957 | -2.139575 | 0.03514132 | 0.96713357 | -4.065198 | -2.1067262 |
| TP73-AS1 | 0.20186764 | 5.63853506 | 2.14257469 | 0.03519336 | 0.96713357 | -4.0468243 | 2.10612654 |
| KRT18P25 | -1.1147952 | -6.4526048 | -2.1425751 | 0.03521693 | 0.96713357 | -4.0311877 | -2.1058553 |
| PHLDA1 | 0.4372867 | 6.85796176 | 2.1417317 | 0.03523113 | 0.96713357 | -4.0469873 | 2.10569185 |
| OAF | 0.63610404 | 3.59170748 | 2.1426282 | 0.03526476 | 0.96713357 | -4.0654354 | 2.10530504 |
| NHERF4 | -1.0792682 | -3.296247 | -2.1401813 | 0.03535119 | 0.96713357 | -4.0563151 | -2.1043126 |
| RPS27P2 | 1.27638085 | -4.1117931 | 2.13663178 | 0.0353876 | 0.96713357 | -4.0435605 | 2.10389505 |
| TNK2-AS1 | -0.7438104 | -2.2160671 | -2.1378051 | 0.03557132 | 0.96713357 | -4.0519889 | -2.1017941 |
| IL17RB | -0.1768211 | 3.44257826 | -2.1362348 | 0.03573141 | 0.96713357 | -4.0408243 | -2.0999707 |
| RNU6-181P | -0.9420196 | -3.4425364 | -2.1315227 | 0.03581823 | 0.96713357 | -4.0662471 | -2.0989849 |
| PKM | 0.25514006 | 9.59237943 | 2.13384913 | 0.03590325 | 0.96713357 | -4.0510115 | 2.09802146 |
| LINC01750 | -0.5010983 | 1.65292029 | -2.1330464 | 0.03616107 | 0.96713357 | -4.0254686 | -2.0951116 |
| LINC02885 | -0.919977 | -1.8577116 | -2.1312375 | 0.03617162 | 0.96713357 | -4.0249611 | -2.0949929 |
| TAPT1 | -0.1378299 | 5.6168826 | -2.1297137 | 0.03626545 | 0.96713357 | -4.0587186 | -2.0939386 |
| DUSP3 | 0.26224499 | 6.18054828 | 2.12921345 | 0.03631073 | 0.96713357 | -4.0560894 | 2.09343065 |
| SMG1P5 | -0.5165641 | -0.9599787 | -2.1299583 | 0.03631494 | 0.96713357 | -4.0935214 | -2.0933835 |
| HACL1 | 0.1631618 | 3.79419477 | 2.129656 | 0.03632031 | 0.96713357 | -4.068776 | 2.09332325 |
| SLITRK6 | 0.51186245 | 2.64954859 | 2.12822298 | 0.03641472 | 0.96713357 | -4.0772622 | 2.09226605 |
| IKBKGP1 | -0.6899533 | -2.9335385 | -2.1268682 | 0.03668676 | 0.96713357 | -4.0682026 | -2.089233 |
| OGFOD3 | 0.16855166 | 5.59150139 | 2.12540803 | 0.03668932 | 0.96713357 | -4.065438 | 2.08920453 |
| LRRC31 | -1.1727167 | -6.1237462 | -2.1205647 | 0.03672529 | 0.96713357 | -4.0493551 | -2.0888049 |
| LINC02599 | -1.2154929 | -6.2488718 | -2.1237013 | 0.03673102 | 0.96713357 | -4.0544902 | -2.0887413 |
| PCDH8 | 0.50089528 | 6.5857073 | 2.12319987 | 0.03679863 | 0.96713357 | -4.0585244 | 2.08799118 |
| ETF1 | 0.14336808 | 6.73548473 | 2.12416777 | 0.03680145 | 0.96713357 | -4.0576348 | 2.08795995 |
| DNAH6 | -0.2799392 | 4.2238867 | -2.1230696 | 0.03685062 | 0.96713357 | -4.0414307 | -2.0874152 |
| MIR3677HG | -0.4101054 | 0.63991116 | -2.1228704 | 0.03685921 | 0.96713357 | -4.0888164 | -2.0873201 |
| DHX37 | 0.15031928 | 5.12296304 | 2.12183355 | 0.03689957 | 0.96713357 | -4.0681279 | 2.08687352 |
| POFUT1 | 0.23371344 | 6.37132967 | 2.12101673 | 0.03705171 | 0.96713357 | -4.0584732 | 2.08519381 |
| MIR3976HG | 1.34915665 | -5.1973116 | 2.1195315 | 0.03707248 | 0.96713357 | -4.1264623 | 2.08496496 |
| RPL11P3 | -1.0376043 | -3.2442547 | -2.1249674 | 0.03708326 | 0.96713357 | -4.0683226 | -2.0848462 |
| GAPDHP38 | -1.2507997 | -3.7516072 | -2.1180661 | 0.03718741 | 0.96713357 | -4.031352 | -2.0837006 |
| H2AC14 | -0.9547161 | -6.6666084 | -2.1171299 | 0.03728523 | 0.96713357 | -4.0489799 | -2.082627 |
| PLAT | 0.71986709 | 4.01116966 | 2.11791391 | 0.03728976 | 0.96713357 | -4.0452938 | 2.0825774 |
| FAM53A | 0.45609424 | 0.08052666 | 2.11477701 | 0.03744972 | 0.96713357 | -4.0987291 | 2.08082726 |
| RNF121 | 0.12603454 | 4.86611972 | 2.11192131 | 0.03778325 | 0.96713357 | -4.074705 | 2.07719842 |
| AKNAD1 | -0.5582898 | -0.098243 | -2.1111964 | 0.03783466 | 0.96713357 | -4.0257988 | -2.0766415 |
| STAP2 | 0.57708126 | 1.49983783 | 2.10995681 | 0.03806844 | 0.96713357 | -4.0965207 | 2.07411709 |
| SHISA2 | 0.49501484 | 2.66520708 | 2.10902451 | 0.03809426 | 0.96713357 | -4.0852268 | 2.07383904 |
| TMEM212 | -0.6986481 | -6.3773476 | -2.1080954 | 0.03816899 | 0.96713357 | -4.0582653 | -2.0730353 |
| NPM2 | -0.4085964 | 1.36256338 | -2.107651 | 0.03821576 | 0.96713357 | -4.0624747 | -2.072533 |
| TMEM98 | 0.24608596 | 6.0673043 | 2.10737822 | 0.03821911 | 0.96713357 | -4.0694709 | 2.07249711 |
| NBAT1 | -0.4584177 | -1.0915835 | -2.1074024 | 0.03823455 | 0.96713357 | -4.0743104 | -2.0723314 |
| CDC37L1-DT | -0.5959457 | -1.2669153 | -2.1055661 | 0.03839369 | 0.96713357 | -4.0641297 | -2.0706267 |
| VAMP5 | 0.85256966 | 2.16227441 | 2.10507184 | 0.03846115 | 0.96713357 | -4.0698646 | 2.06990597 |
| SHC1P2 | -1.3841733 | -4.7434422 | -2.1042306 | 0.03847693 | 0.96713357 | -4.0910349 | -2.0697375 |
| ZC3HAV1L | 0.26262053 | 2.67360822 | 2.10324645 | 0.03856049 | 0.96713357 | -4.0849813 | 2.06884658 |
| PTS | -0.2071413 | 4.73451372 | -2.1038437 | 0.03856347 | 0.96713357 | -4.0822057 | -2.0688148 |
| GCAT | 0.18462886 | 4.50040651 | 2.10361496 | 0.0385869 | 0.96713357 | -4.0630301 | 2.06856532 |
| PDP1 | 0.24972392 | 6.00459882 | 2.10103709 | 0.03879741 | 0.96713357 | -4.0815063 | 2.06632915 |
| CACNG5 | 0.83476771 | 5.04580976 | 2.09994744 | 0.03881697 | 0.96713357 | -4.0711599 | 2.06612186 |
| CLMP | 0.35382868 | 2.98118084 | 2.10076877 | 0.03883045 | 0.96713357 | -4.1065759 | 2.06597912 |
| FLT3LG | -1.1041598 | -2.56484 | -2.0995479 | 0.03886821 | 0.96713357 | -4.085924 | -2.0655794 |
| BDKRB2 | 1.04057539 | -1.0479431 | 2.09941499 | 0.03891734 | 0.96713357 | -4.0821816 | 2.06505982 |
| SNORD124 | -1.0471938 | -3.0273688 | -2.1000321 | 0.03894569 | 0.96713357 | -4.0903266 | -2.0647603 |
| OR7L1P | -1.1965968 | -5.8518997 | -2.0979364 | 0.03896829 | 0.96713357 | -4.0702898 | -2.0645216 |
| P4HB | 0.19308305 | 7.9952031 | 2.09860136 | 0.0390428 | 0.96713357 | -4.0762972 | 2.06373546 |
| ANGPTL8 | 1.04273219 | -3.3779279 | 2.09686189 | 0.03908048 | 0.96713357 | -4.1249495 | 2.06333852 |
| SOX21-AS1 | 0.29869203 | 5.19692064 | 2.09798845 | 0.0390806 | 0.96713357 | -4.0754117 | 2.06333721 |
| TP53RK | 0.21973452 | 5.08014506 | 2.09707922 | 0.03908125 | 0.96713357 | -4.0804838 | 2.06333035 |
| GRN | 0.40310576 | 7.03095044 | 2.09796461 | 0.03908948 | 0.96713357 | -4.0742115 | 2.06324366 |
| DMBT1 | -1.3796543 | -3.9835958 | -2.0971725 | 0.03910822 | 0.96713357 | -4.0946902 | -2.0630464 |
| ZSCAN32 | 0.14395978 | 4.07668855 | 2.09789661 | 0.03912015 | 0.96713357 | -4.097401 | 2.06292085 |
| LSG1 | 0.11494222 | 5.70982876 | 2.09691253 | 0.03918443 | 0.96713357 | -4.0826617 | 2.06224488 |
| HK3 | -0.9215824 | -2.519865 | -2.0959094 | 0.0392744 | 0.96713357 | -4.0967537 | -2.0613003 |
| RNF139 | 0.14703765 | 5.61562829 | 2.09322507 | 0.03947745 | 0.96713357 | -4.07535 | 2.05917527 |
| MTMR14 | 0.13854009 | 5.02353346 | 2.09232651 | 0.03955509 | 0.96713357 | -4.0904082 | 2.05836525 |
| HOXA9 | -1.0909922 | -5.9174621 | -2.0920689 | 0.03960238 | 0.96713357 | -4.0206992 | -2.0578724 |
| RPS26P28 | -1.3866128 | -4.5559131 | -2.088215 | 0.03965193 | 0.96713357 | -4.0587933 | -2.0573566 |
| CCNYL7 | -1.1280142 | -3.3233707 | -2.0911126 | 0.03966846 | 0.96713357 | -4.107709 | -2.0571847 |
| CHRAC1 | 0.23566571 | 4.13098102 | 2.09137905 | 0.03971306 | 0.96713357 | -4.0957404 | 2.05672109 |
| SIGLEC10-AS1 | 1.11006901 | -5.194551 | 2.09146113 | 0.03971951 | 0.96713357 | -4.0797816 | 2.05665415 |
| VPS18 | 0.16566613 | 5.86907269 | 2.08901901 | 0.03973271 | 0.96713357 | -4.0809251 | 2.05651695 |
| FHDC1 | -0.401985 | 3.59539031 | -2.0901916 | 0.03979644 | 0.96713357 | -4.0909741 | -2.0558556 |
| PRDM4-AS1 | -0.5725623 | -1.01728 | -2.088879 | 0.03982761 | 0.96713357 | -4.1060289 | -2.0555324 |
| C1QTNF6 | 0.65457145 | 5.37577638 | 2.08993202 | 0.03990528 | 0.96713357 | -4.0904845 | 2.05472809 |
| FAM210B | 0.20795952 | 6.03116472 | 2.08884685 | 0.03991994 | 0.96713357 | -4.0794605 | 2.05457641 |
| TPTE2 | -0.564119 | -0.7340921 | -2.0883506 | 0.03992247 | 0.96713357 | -4.1249863 | -2.0545502 |
| LEAP2 | -0.6743388 | -0.3191013 | -2.0878974 | 0.03996185 | 0.96713357 | -4.121168 | -2.054143 |
| RCBTB1 | 0.09330695 | 6.12339229 | 2.08689233 | 0.04002396 | 0.96713357 | -4.0867411 | 2.05350151 |
| RBM44 | -0.4155179 | 1.22739292 | -2.08822 | 0.04004413 | 0.96713357 | -4.1362347 | -2.0532934 |
| LINC03014 | -0.4899085 | -0.6827442 | -2.0868878 | 0.04010065 | 0.96713357 | -4.121689 | -2.0527106 |
| KCNMB2-AS1 | -0.4414843 | -0.7958667 | -2.0865684 | 0.0401387 | 0.96713357 | -4.0424404 | -2.0523187 |
| CERS4 | 0.13198729 | 5.76621443 | 2.08629491 | 0.04017976 | 0.96713357 | -4.0882562 | 2.05189612 |
| DDOST | 0.19560195 | 7.39463374 | 2.08539484 | 0.04026837 | 0.96713357 | -4.0850886 | 2.05098539 |
| SGPL1 | 0.17473473 | 6.64415905 | 2.08519601 | 0.0402756 | 0.96713357 | -4.08408 | 2.05091115 |
| RESP18 | -1.1185257 | -4.380411 | -2.0841522 | 0.04034389 | 0.96713357 | -4.1045751 | -2.0502105 |
| CCDC144A | -0.8255943 | -1.6667062 | -2.0829315 | 0.04037525 | 0.96713357 | -4.1108952 | -2.0498892 |
| TST | 0.24935573 | 3.24644811 | 2.08323028 | 0.04045093 | 0.96713357 | -4.0746769 | 2.0491144 |
| ZBTB49 | -0.1820354 | 3.10888886 | -2.0825386 | 0.04045663 | 0.96713357 | -4.1022891 | -2.0490561 |
| RNA5SP242 | -1.2251858 | -5.6453846 | -2.0792443 | 0.04048852 | 0.96713357 | -4.0965822 | -2.0487301 |
| CSTF1 | 0.12510496 | 4.75780666 | 2.08178207 | 0.04054239 | 0.96713357 | -4.0921869 | 2.04817977 |
| GULOP | 1.11061294 | -6.1809552 | 2.07904136 | 0.04070329 | 0.96713357 | -4.10822 | 2.04653979 |
| CCDC62 | -0.4089984 | 0.65749462 | -2.0801014 | 0.04075299 | 0.96713357 | -4.1182787 | -2.0460343 |
| SMPD5 | -0.5573247 | -0.4984308 | -2.0795938 | 0.04080335 | 0.96713357 | -4.0392032 | -2.0455227 |
| MYO5B | 0.78529936 | 3.64083119 | 2.07825047 | 0.04087996 | 0.96713357 | -4.0555715 | 2.04474544 |
| FAM78A | 0.24575581 | 1.53779827 | 2.07822072 | 0.04092234 | 0.96713357 | -4.1008156 | 2.04431604 |
| NMNAT1 | 0.11520787 | 3.57130762 | 2.07749882 | 0.04097275 | 0.96713357 | -4.0805899 | 2.04380569 |
| COL4A4 | -0.5267353 | 1.13131102 | -2.0772058 | 0.0409949 | 0.96713357 | -4.1236066 | -2.0435816 |
| SNAI1 | 0.50401157 | -0.425378 | 2.07685873 | 0.04109138 | 0.96713357 | -4.1060558 | 2.04260681 |
| IL1RL2 | 1.35310583 | -4.1149077 | 2.07629618 | 0.04109303 | 0.96713357 | -4.0959844 | 2.0425901 |
| PBX1-AS1 | 1.19720796 | -5.9283375 | 2.07491441 | 0.04110703 | 0.96713357 | -4.0843732 | 2.04244882 |
| ZBTB42 | 0.45533039 | 2.39065935 | 2.07600388 | 0.04113102 | 0.96713357 | -4.0937428 | 2.04220678 |
| SNORD115-4 | -0.9219639 | -4.3771416 | -2.0747351 | 0.04120044 | 0.96713357 | -4.1184576 | -2.0415072 |
| CLEC4D | -1.1442326 | -5.6653273 | -2.0738979 | 0.04122266 | 0.96713357 | -4.0910904 | -2.0412834 |
| RPL7P47 | 1.35835292 | -4.983565 | 2.07271421 | 0.04139144 | 0.96713357 | -4.0840637 | 2.03958737 |
| BAIAP2-DT | 0.27785464 | 6.61155742 | 2.072264 | 0.0414309 | 0.96713357 | -4.0908631 | 2.03919168 |
| MMP19 | 0.86501245 | 1.92235479 | 2.0730643 | 0.04147344 | 0.96713357 | -4.0739368 | 2.03876548 |
| POLRMT | 0.12487221 | 6.16613207 | 2.07192571 | 0.04148465 | 0.96713357 | -4.0996847 | 2.03865322 |
| SIMC1P1 | -0.2945301 | 0.26513338 | -2.0724902 | 0.04149472 | 0.96713357 | -4.1114473 | -2.0385524 |
| ATP6V0A4 | -0.9571768 | -2.9913434 | -2.0706231 | 0.0415699 | 0.96713357 | -4.0833036 | -2.0378004 |
| NMRK2 | -1.2332147 | -3.8380548 | -2.0702267 | 0.04157694 | 0.96713357 | -4.1348697 | -2.03773 |
| ABCC3 | 1.06740006 | 0.54148157 | 2.07129829 | 0.04158177 | 0.96713357 | -4.0715084 | 2.0376818 |
| CYP2D8P | -0.4096952 | 1.84524508 | -2.0712628 | 0.04159172 | 0.96713357 | -4.1553019 | -2.0375823 |
| ZFX-AS1 | -0.5732805 | -1.9949452 | -2.0694115 | 0.04159434 | 0.96713357 | -4.0881298 | -2.0375562 |
| LINC01164 | -1.3806936 | -5.4388557 | -2.0702178 | 0.04159445 | 0.96713357 | -4.1062842 | -2.0375551 |
| CNBP | 0.16552112 | 7.68997703 | 2.0705743 | 0.04163318 | 0.96713357 | -4.095453 | 2.03716827 |
| LINC01933 | -0.8778003 | -2.1392902 | -2.0698524 | 0.04168648 | 0.96713357 | -4.1058863 | -2.0366366 |
| RPL9P3 | -1.3348714 | -4.2631037 | -2.0695401 | 0.04170377 | 0.96713357 | -4.0650949 | -2.0364642 |
| TGFBI | 1.32643643 | 2.25657607 | 2.06948484 | 0.04176884 | 0.96713357 | -4.0816861 | 2.03581595 |
| LINC02093 | -1.174015 | -5.7831796 | -2.0685704 | 0.04180288 | 0.96713357 | -4.1009283 | -2.0354772 |
| OS9 | 0.14336566 | 7.51320021 | 2.06837255 | 0.04182019 | 0.96713357 | -4.0953511 | 2.03530499 |
| RELL1 | 0.35405429 | 4.1595301 | 2.06813754 | 0.04186941 | 0.96713357 | -4.0754763 | 2.03481576 |
| LINC00957 | -0.5711418 | -0.8302195 | -2.0666539 | 0.04201796 | 0.96713357 | -4.039479 | -2.0333422 |
| MYL12A | 0.39111816 | 4.57247133 | 2.06650895 | 0.04205964 | 0.96713357 | -4.0959272 | 2.03292955 |
| DENND6A-AS1 | -1.1009692 | -4.7585653 | -2.0644757 | 0.04207862 | 0.96713357 | -4.0820981 | -2.0327417 |
| DACT2 | 0.49172532 | 0.25071611 | 2.06536088 | 0.04212578 | 0.96713357 | -4.0881473 | 2.03227539 |
| ZNF180 | 0.12262146 | 4.2252134 | 2.06463739 | 0.04218718 | 0.96713357 | -4.1146352 | 2.03166898 |
| SDC4 | 0.89252235 | 2.84411279 | 2.0652067 | 0.04220267 | 0.96713357 | -4.0887557 | 2.03151607 |
| TRAM1 | 0.31011443 | 6.58136457 | 2.06475595 | 0.04220653 | 0.96713357 | -4.0965756 | 2.03147796 |
| MAP3K11 | 0.1357756 | 5.37170431 | 2.06359885 | 0.04233578 | 0.96713357 | -4.1044922 | 2.03020422 |
| KCNH6 | -0.8796513 | -2.0898277 | -2.0618519 | 0.04241884 | 0.96713357 | -4.1371278 | -2.0293875 |
| LINC01615 | 1.32807448 | -5.0262721 | 2.0609472 | 0.04249679 | 0.96713357 | -4.1019802 | 2.02862218 |
| CMTM2 | -0.8818093 | -2.006353 | -2.0581695 | 0.0425432 | 0.96713357 | -4.1554041 | -2.0281671 |
| DUOXA2 | -1.0655822 | -4.027478 | -2.0602506 | 0.04257597 | 0.96713357 | -4.1221451 | -2.0278459 |
| CICP25 | -1.068501 | -6.4832515 | -2.0597624 | 0.0425837 | 0.96713357 | -4.0859719 | -2.0277702 |
| HLA-DQB1 | 1.19433896 | -1.4753899 | 2.0600584 | 0.04273752 | 0.96713357 | -4.1681058 | 2.02626615 |
| AOC3 | -0.3684391 | 0.88467051 | -2.0582075 | 0.04281441 | 0.96713357 | -4.1563319 | -2.025516 |
| PDCL3P5 | 0.57711358 | -1.2965573 | 2.05809809 | 0.04285871 | 0.96713357 | -4.107642 | 2.02508428 |
| ARIH2OS | -0.227484 | 1.60521769 | -2.057343 | 0.04286569 | 0.96713357 | -4.1075054 | -2.0250163 |
| C1R | 1.01642658 | 5.42224774 | 2.05782447 | 0.0429358 | 0.96713357 | -4.1218339 | 2.024334 |
| IGHD | 1.03718495 | -6.8668614 | 2.05586612 | 0.0430318 | 0.96713357 | -4.104447 | 2.02340124 |
| FGF13-AS1 | -0.4100148 | -0.6730454 | -2.055484 | 0.04306914 | 0.96713357 | -4.075648 | -2.0230389 |
| ROR1 | 0.6277504 | 2.57698794 | 2.05509153 | 0.04307193 | 0.96713357 | -4.0855226 | 2.02301178 |
| HMGA2 | 0.99048565 | -0.2117041 | 2.0550696 | 0.0430756 | 0.96713357 | -4.0562792 | 2.02297617 |
| CSNK1G2P1 | -1.2196473 | -5.4447797 | -2.0546269 | 0.04307619 | 0.96713357 | -4.1065093 | -2.0229705 |
| ITGA1 | 0.73727521 | 0.31398891 | 2.05563832 | 0.04317347 | 0.96713357 | -4.0757053 | 2.0220279 |
| FEM1AP2 | 1.26135699 | -4.0203663 | 2.05399821 | 0.04324431 | 0.96713357 | -4.1411513 | 2.02134259 |
| ELF3 | 1.2517356 | -0.5059265 | 2.05950095 | 0.04333942 | 0.96713357 | -4.2022841 | 2.02042404 |
| RIN3 | 0.32821349 | 2.67031738 | 2.05289315 | 0.04337258 | 0.96713357 | -4.0966784 | 2.02010425 |
| TANAR | -1.1917617 | -5.665419 | -2.0498505 | 0.04358546 | 0.96713357 | -4.1029333 | -2.0180557 |
| EEF1DP4 | -0.5656683 | -0.3503327 | -2.0490161 | 0.04373178 | 0.96713357 | -4.112987 | -2.0166526 |
| SPHK1 | 0.75807871 | 2.61009484 | 2.04873408 | 0.04374897 | 0.96713357 | -4.132788 | 2.01648798 |
| UBBP1 | 1.22502125 | -5.0364777 | 2.04698523 | 0.04376353 | 0.96713357 | -4.1149628 | 2.01634865 |
| CXCL5 | 1.38555793 | -1.2849032 | 2.04940249 | 0.0438518 | 0.96713357 | -4.0819305 | 2.0155046 |
| ZNF286A-TBC1D26 | -0.9514824 | -2.586717 | -2.0441178 | 0.04391443 | 0.96713357 | -4.1203815 | -2.0149066 |
| NPC1 | 0.18351188 | 5.5356465 | 2.04731297 | 0.04393237 | 0.96713357 | -4.1075672 | 2.01473555 |
| RECQL | 0.19388516 | 4.53565273 | 2.04732019 | 0.04394939 | 0.96713357 | -4.106594 | 2.01457315 |
| PRR16 | 0.37803913 | 1.61424441 | 2.04724966 | 0.04396157 | 0.96713357 | -4.0952151 | 2.01445706 |
| RPL35AP14 | -1.1895744 | -6.2877432 | -2.0430547 | 0.04398863 | 0.96713357 | -4.1017634 | -2.0141992 |
| CHSY1 | 0.19066632 | 5.81809525 | 2.04343172 | 0.04402043 | 0.96713357 | -4.1007086 | 2.01389627 |
| RGPD4 | -0.3637366 | 0.43453398 | -2.0452459 | 0.04404984 | 0.96713357 | -4.1162925 | -2.0136162 |
| SNX8 | 0.19741668 | 5.37392314 | 2.04427908 | 0.04418361 | 0.96713357 | -4.1079329 | 2.01234473 |
| DUSP8P1 | -1.1421377 | -2.867002 | -2.0431321 | 0.044196 | 0.96713357 | -4.1449201 | -2.0122271 |
| SIM2 | -1.4875801 | -3.3268452 | -2.0443955 | 0.04419902 | 0.96713357 | -4.1248557 | -2.0121985 |
| PPP1R9A-AS1 | 0.9370707 | -1.2003752 | 2.04327327 | 0.04425866 | 0.96713357 | -4.1416376 | 2.01163279 |
| ERVMER34-1 | -1.2541044 | -3.7398441 | -2.0439841 | 0.04427973 | 0.96713357 | -4.1030138 | -2.0114331 |
| NR1I2 | -0.7029662 | -0.5664536 | -2.0435166 | 0.04428712 | 0.96713357 | -4.1711945 | -2.0113631 |
| LINC02197 | 0.98540924 | -2.606161 | 2.0429447 | 0.04430531 | 0.96713357 | -4.165362 | 2.01119074 |
| PRADC1P1 | 1.1932064 | -3.5566296 | 2.04504253 | 0.04432346 | 0.96713357 | -4.1490721 | 2.01101888 |
| LINC02010 | 1.18155085 | -5.2392557 | 2.04336958 | 0.04433925 | 0.96713357 | -4.1322026 | 2.01086947 |
| C1QTNF8 | -0.9247999 | -1.5171182 | -2.0420153 | 0.04441231 | 0.96713357 | -4.1620525 | -2.0101784 |
| TUBB6 | 0.35178023 | 5.48389897 | 2.04168846 | 0.04452378 | 0.96713357 | -4.0954178 | 2.00912597 |
| AP4B1-AS1 | -0.6655924 | -0.3497339 | -2.0408035 | 0.04455878 | 0.96713357 | -4.149134 | -2.0087959 |
| NDST1 | 0.15955049 | 8.23852259 | 2.0400073 | 0.04465276 | 0.96713357 | -4.1152725 | 2.00791093 |
| NRG2 | -0.226699 | 3.76813256 | -2.0403736 | 0.04466241 | 0.96713357 | -4.131199 | -2.0078201 |
| CPA4 | 0.81849924 | -0.9883746 | 2.03945578 | 0.04467204 | 0.96713357 | -4.1116756 | 2.00772953 |
| ZNF665 | 0.20793814 | 2.43551221 | 2.0385786 | 0.04478096 | 0.96713357 | -4.1185642 | 2.00670617 |
| HBP1 | 0.22081441 | 4.56938407 | 2.03840106 | 0.04484465 | 0.96713357 | -4.1189006 | 2.00610869 |
| BAAT | -0.3711264 | 0.2467594 | -2.0376909 | 0.04486309 | 0.96713357 | -4.1620658 | -2.0059359 |
| SLA | 0.56932922 | 3.50730579 | 2.0375796 | 0.04492296 | 0.96713357 | -4.1671383 | 2.00537513 |
| TP63 | 0.98575911 | -2.3960008 | 2.0376189 | 0.0449359 | 0.96713357 | -4.1492016 | 2.005254 |
| CTSS | 1.06053588 | 1.36178399 | 2.03804098 | 0.04498815 | 0.96713357 | -4.1248729 | 2.00476529 |
| ZKSCAN3 | -0.1580397 | 3.97935096 | -2.0355625 | 0.04517431 | 0.96713357 | -4.1113416 | -2.0030278 |
| C16orf46-DT | 1.06172505 | -3.3550388 | 2.03438962 | 0.04518117 | 0.96713357 | -4.1704523 | 2.00296386 |
| CTSC | 0.59984043 | 4.02353624 | 2.03377276 | 0.04527979 | 0.96713357 | -4.099467 | 2.00204593 |
| DMC1 | 0.69636735 | -1.5191928 | 2.03312811 | 0.04528297 | 0.96713357 | -4.1534798 | 2.00201642 |
| GPR146 | 0.7025075 | -1.989843 | 2.03331812 | 0.04528936 | 0.96713357 | -4.1243468 | 2.00195699 |
| RPS15AP24 | -1.1923056 | -4.718357 | -2.0310895 | 0.04529018 | 0.96713357 | -4.0946243 | -2.0019493 |
| ABCC4 | 0.50773247 | 4.12882231 | 2.03370876 | 0.04530755 | 0.96713357 | -4.1221872 | 2.00178792 |
| LINC02076 | -0.5086121 | -0.6152055 | -2.0326551 | 0.04538073 | 0.96713357 | -4.1184759 | -2.0011082 |
| APLF | 0.39178986 | 1.45756828 | 2.03320463 | 0.04543374 | 0.96713357 | -4.1408932 | 2.00061647 |
| SMIM38 | -0.6202 | 0.18632869 | -2.0311982 | 0.04560216 | 0.96713357 | -4.1824568 | -1.9990573 |
| RAB32 | 0.46950676 | 2.49264386 | 2.03068735 | 0.04562638 | 0.96713357 | -4.0944154 | 1.9988334 |
| CD81-AS1 | -0.2762964 | 0.65881057 | -2.0298631 | 0.04562649 | 0.96713357 | -4.1065229 | -1.9988324 |
| LINC02763 | -1.0088977 | -1.7211482 | -2.0288542 | 0.04567555 | 0.96713357 | -4.1741332 | -1.9983793 |
| ACTG1P17 | -0.7834867 | -1.9631068 | -2.0307307 | 0.0457217 | 0.96713357 | -4.1104166 | -1.9979536 |
| EP300-AS1 | -0.4227015 | -0.9135726 | -2.0293054 | 0.04577759 | 0.96713357 | -4.1018061 | -1.9974383 |
| LDHBP2 | -1.1767864 | -3.4217311 | -2.0294672 | 0.04580804 | 0.96713357 | -4.1335166 | -1.9971578 |
| LATS2 | 0.25251735 | 3.73418483 | 2.02867757 | 0.04585069 | 0.96713357 | -4.108343 | 1.99676527 |
| RPL15P3 | 0.27346382 | 0.96031318 | 2.02748666 | 0.04587653 | 0.96713357 | -4.1126787 | 1.99652757 |
| SBNO2 | 0.38252266 | 5.3422898 | 2.02961282 | 0.04587704 | 0.96713357 | -4.1277592 | 1.99652284 |
| SLC24A1 | 0.22296814 | 3.43613622 | 2.02701234 | 0.04601984 | 0.96713357 | -4.1166563 | 1.99521127 |
| P2RX4 | 0.24690514 | 3.54749562 | 2.02503141 | 0.04626779 | 0.96713357 | -4.1362142 | 1.99294203 |
| DNASE1L2 | -0.3267688 | 1.80785462 | -2.0258465 | 0.04630069 | 0.96713357 | -4.1382521 | -1.9926418 |
| LHX2-AS1 | 1.05672419 | -2.8169473 | 2.02179693 | 0.04650142 | 0.96713357 | -4.2063339 | 1.99081328 |
| PRH2 | -0.8105999 | -2.6357845 | -2.021611 | 0.0465091 | 0.96713357 | -4.1291822 | -1.9907434 |
| GPR39 | 0.43556183 | 0.15071375 | 2.0215609 | 0.04655076 | 0.96713357 | -4.1418123 | 1.99036483 |
| CENPP | 0.16446414 | 2.6903479 | 2.02119749 | 0.04661864 | 0.96713357 | -4.1353744 | 1.98974854 |
| LINC02764 | -1.1098378 | -6.0167662 | -2.0193189 | 0.04665328 | 0.96713357 | -4.1280868 | -1.9894343 |
| MET | 0.65804658 | 0.95509786 | 2.02047306 | 0.04666878 | 0.96713357 | -4.1169347 | 1.98929386 |
| ALPK2 | 1.13392786 | 0.63086661 | 2.02097389 | 0.04670301 | 0.96713357 | -4.1128465 | 1.98898362 |
| RANP4 | -0.3961365 | 0.67394701 | -2.0194988 | 0.04673154 | 0.96713357 | -4.1382846 | -1.9887252 |
| DUSP28 | -0.1412506 | 3.63429482 | -2.0202738 | 0.04673303 | 0.96713357 | -4.1537761 | -1.9887117 |
| MTND1P9 | -1.1850671 | -5.9919335 | -2.019629 | 0.0467428 | 0.96713357 | -4.0992099 | -1.9886233 |
| GRHL1 | -0.4010578 | 2.4569912 | -2.0206536 | 0.04676176 | 0.96713357 | -4.103437 | -1.9884517 |
| A2M | 1.34864985 | 6.21128908 | 2.02004374 | 0.04679528 | 0.96713357 | -4.1131058 | 1.98814836 |
| EQTN | -1.0838589 | -5.5082813 | -2.0188814 | 0.04680365 | 0.96713357 | -4.1247013 | -1.9880727 |
| ARHGAP29 | 0.66888558 | 5.14424154 | 2.01877986 | 0.04687925 | 0.96713357 | -4.1255458 | 1.98738953 |
| NECTIN2 | 0.21573412 | 6.24760514 | 2.01872472 | 0.04689663 | 0.96713357 | -4.1273651 | 1.98723256 |
| LTBP1 | 0.57626331 | 5.67626162 | 2.01695365 | 0.04706972 | 0.96713357 | -4.1143551 | 1.98567231 |
| SUPT6H | 0.10098758 | 7.29369569 | 2.01697669 | 0.04710453 | 0.96713357 | -4.1315539 | 1.9853591 |
| GALT | -0.5051462 | 2.05666562 | -2.0161597 | 0.04712446 | 0.96713357 | -4.173109 | -1.9851799 |
| RNU6-794P | -1.061355 | -6.1638228 | -2.0151539 | 0.04713105 | 0.96713357 | -4.1347515 | -1.9851206 |
| MAN2B1 | 0.34469167 | 6.2708474 | 2.01617916 | 0.04717325 | 0.96713357 | -4.1335845 | 1.98474142 |
| MIR5582 | -1.1933413 | -5.7334807 | -2.0151528 | 0.047177 | 0.96713357 | -4.1440843 | -1.9847078 |
| SMC2-DT | 0.68244859 | -1.5981911 | 2.01613173 | 0.04718799 | 0.96713357 | -4.1854628 | 1.984609 |
| R3HCC1L | 0.1355099 | 3.90185591 | 2.01562967 | 0.04719589 | 0.96713357 | -4.1485794 | 1.98453804 |
| ZNFX1 | 0.12885177 | 5.93208697 | 2.01550014 | 0.04723474 | 0.96713357 | -4.1334655 | 1.98418928 |
| HS6ST1P1 | -0.4905518 | -1.1107695 | -2.016204 | 0.0472462 | 0.96713357 | -4.1233503 | -1.9840865 |
| HS3ST6 | 0.75886037 | -4.1111524 | 2.01425711 | 0.04730888 | 0.96713357 | -4.1404803 | 1.98352443 |
| SPOCD1 | 1.35041864 | 0.17384406 | 2.01525028 | 0.04737168 | 0.96713357 | -4.1115587 | 1.98296194 |
| EPHB4 | 0.43035922 | 4.99601022 | 2.01391257 | 0.04739832 | 0.96713357 | -4.1199755 | 1.98272357 |
| KIF13B | 0.23780544 | 5.13486604 | 2.01388302 | 0.04740983 | 0.96713357 | -4.1322271 | 1.98262053 |
| LINC02338 | 0.75238597 | -4.043712 | 2.01352909 | 0.04745129 | 0.96713357 | -4.1421632 | 1.98224981 |
| LINC02857 | -1.2189233 | -5.4756601 | -2.0110601 | 0.04769751 | 0.96713357 | -4.1073178 | -1.9800535 |
| MIR4527HG | -0.9879573 | -5.468121 | -2.01088 | 0.04773063 | 0.96713357 | -4.0932413 | -1.9797589 |
| CDH1 | 0.8835772 | 1.90876025 | 2.0103095 | 0.04774692 | 0.96713357 | -4.0979103 | 1.97961398 |
| ABCA1 | 0.29880978 | 6.76233145 | 2.01059995 | 0.04776558 | 0.96713357 | -4.1347444 | 1.97944805 |
| TPM1-AS | 1.10557065 | -4.3399852 | 2.00945847 | 0.04780835 | 0.96713357 | -4.1492696 | 1.97906797 |
| BNIP3P9 | -1.1408731 | -5.8308268 | -2.0106989 | 0.04785563 | 0.96713357 | -4.1304492 | -1.9786482 |
| ANKRD36 | -0.3420742 | 4.75550486 | -2.0089676 | 0.04792624 | 0.96713357 | -4.1535497 | -1.9780219 |
| HSD17B1 | -0.4207415 | -0.4604886 | -2.0053712 | 0.04797884 | 0.96713357 | -4.1422663 | -1.9775558 |
| NSA2P2 | -1.2181646 | -4.7189409 | -2.0067516 | 0.0479908 | 0.96713357 | -4.129002 | -1.9774499 |
| CXCL11 | 1.26403545 | -4.4520466 | 2.00910316 | 0.04799355 | 0.96713357 | -4.118032 | 1.9774255 |
| ITPRIPL1 | 0.8453639 | 0.3681032 | 2.00800478 | 0.04806652 | 0.96713357 | -4.1919872 | 1.97677989 |
| VCAM1 | 0.42456585 | 6.4443239 | 2.00773246 | 0.04807546 | 0.96713357 | -4.1289638 | 1.97670083 |
| FAM161A | -0.1894022 | 5.03171676 | -2.0070641 | 0.04810489 | 0.96713357 | -4.1373507 | -1.9764407 |
| TAS2R4 | -0.4039283 | 0.36952885 | -2.0064978 | 0.04811545 | 0.96713357 | -4.1336141 | -1.9763474 |
| LINC02347 | -1.1726871 | -6.0946118 | -2.0057545 | 0.04820098 | 0.96713357 | -4.1498375 | -1.9755922 |
| LINC02321 | -1.1237987 | -4.6914571 | -2.0031477 | 0.0482205 | 0.96713357 | -4.097958 | -1.9754201 |
| LRRC37A12P | -1.1583236 | -5.3099976 | -2.0055335 | 0.04823368 | 0.96713357 | -4.1441993 | -1.9753038 |
| MLN | -1.0711967 | -4.2835121 | -2.0025197 | 0.04833614 | 0.96713357 | -4.1368185 | -1.9744012 |
| DNPEP-AS1 | -0.8529019 | -3.2947658 | -2.0030863 | 0.04835685 | 0.96713357 | -4.1018129 | -1.9742189 |
| SMIM39 | -0.2924279 | 2.18500861 | -2.0042038 | 0.04838329 | 0.96713357 | -4.1518104 | -1.9739864 |
| DTX3L | 0.45994966 | 3.85116646 | 2.00473027 | 0.04841809 | 0.96713357 | -4.1167756 | 1.97368045 |
| ZKSCAN8P1 | -0.4675392 | 1.02421681 | -2.0029927 | 0.04847458 | 0.96713357 | -4.1214634 | -1.9731842 |
| GOLIM4 | 0.30834219 | 6.31636242 | 2.00371969 | 0.0485158 | 0.96713357 | -4.1356339 | 1.97282241 |
| HPS1-AS1 | 0.67565196 | -1.1882908 | 2.00281774 | 0.04865632 | 0.96713357 | -4.1996384 | 1.97159092 |
| B3GNT2 | 0.33433622 | 3.69869309 | 2.00159398 | 0.04866741 | 0.96713357 | -4.1529225 | 1.97149386 |
| LINC02211 | -0.8340617 | -2.2351376 | -2.000406 | 0.04876409 | 0.96713357 | -4.1211138 | -1.9706485 |
| COPS8P2 | -1.1073694 | -3.7648928 | -1.9993509 | 0.04876563 | 0.96713357 | -4.1463808 | -1.9706351 |
| STIMATE-MUSTN1 | -0.6291213 | -1.1750652 | -1.9999457 | 0.04878607 | 0.96713357 | -4.1772933 | -1.9704565 |
| PLEKHA4 | 0.65026806 | 4.61187274 | 2.00073701 | 0.04884142 | 0.96713357 | -4.1216233 | 1.96997336 |
| HPN | -1.0459551 | -2.5880466 | -1.9999541 | 0.04888638 | 0.96713357 | -4.2087977 | -1.9695813 |
| ATXN2-AS | -0.8709544 | -2.5846626 | -1.9978707 | 0.04901002 | 0.96713357 | -4.1523759 | -1.9685045 |
| LGALS3 | 0.70514733 | 3.89357708 | 1.99917544 | 0.04903391 | 0.96713357 | -4.1098732 | 1.96829669 |
| NBPF19 | -0.7268862 | -1.1868818 | -2.0003798 | 0.04908075 | 0.96713357 | -4.1715145 | -1.9678896 |
| CYB5RL | 0.21422 | 3.21601213 | 1.99810856 | 0.04908137 | 0.96713357 | -4.1400045 | 1.96788415 |
| SNHG16 | 0.19488888 | 4.40597867 | 1.99785445 | 0.04911367 | 0.96713357 | -4.1461399 | 1.96760355 |
| BRICD5 | -0.3962143 | 2.73115163 | -1.9964864 | 0.04925946 | 0.96713357 | -4.167791 | -1.9663391 |
| MBD3L3 | -0.8783294 | -3.7395876 | -1.9991516 | 0.0492632 | 0.96713357 | -4.1328083 | -1.9663066 |
| PLIN3 | 0.31005154 | 4.89228969 | 1.9965302 | 0.04929515 | 0.96713357 | -4.122346 | 1.96602994 |
| PEA15 | -0.2738284 | 9.81085964 | -1.9964514 | 0.04929555 | 0.96713357 | -4.1418752 | -1.9660265 |
| ECHDC3 | 0.58546453 | 0.61509995 | 1.99619655 | 0.04930342 | 0.96713357 | -4.1586584 | 1.96595835 |
| RN7SL678P | -1.0307907 | -5.0810443 | -1.9952159 | 0.04932203 | 0.96713357 | -4.132433 | -1.9657973 |
| WTAP | 0.20466819 | 6.49100785 | 1.99589365 | 0.04934225 | 0.96713357 | -4.143321 | 1.96562232 |
| ENPP7P7 | -0.8574616 | -2.5268572 | -1.9943634 | 0.04946644 | 0.96713357 | -4.1470653 | -1.9645492 |
| LPCAT3 | 0.23901291 | 3.62543216 | 1.99504934 | 0.04949627 | 0.96713357 | -4.1478871 | 1.96429177 |
| DCDC2B | -0.5220624 | 0.59474114 | -1.993795 | 0.04949688 | 0.96713357 | -4.1720058 | -1.9642865 |
| FKBP9 | 0.28663636 | 7.29440252 | 1.9947965 | 0.04950094 | 0.96713357 | -4.1453536 | 1.96425143 |
| LY75 | 1.18983046 | -3.4539401 | 1.99343931 | 0.04953906 | 0.96713357 | -4.2002301 | 1.96392266 |
| CRYGA | -0.9815672 | -2.6715669 | -1.99359 | 0.04954345 | 0.96713357 | -4.186323 | -1.9638849 |
| RASD1 | 0.64052101 | 4.5114925 | 1.99377689 | 0.04962799 | 0.96713357 | -4.1591641 | 1.96315653 |
| NDUFB5 | -0.1392998 | 4.26893673 | -1.9935046 | 0.04964077 | 0.96713357 | -4.1272918 | -1.9630465 |
| FAHD2A | -0.2058355 | 5.46911225 | -1.9920329 | 0.04969044 | 0.96713357 | -4.1538706 | -1.9626192 |
| ZNF385C | -0.3703708 | 1.36314765 | -1.9918989 | 0.04980318 | 0.96713357 | -4.1518655 | -1.9616506 |
| OLFML2B | 0.64266723 | 3.64480691 | 1.99196849 | 0.0498148 | 0.96713357 | -4.1363758 | 1.96155085 |
| LGSN | 0.99803055 | -2.000136 | 1.99192939 | 0.04986359 | 0.96713357 | -4.1795322 | 1.96113228 |
| RNU2-38P | -0.9155846 | -2.9176623 | -1.9911476 | 0.04991742 | 0.96713357 | -4.1753916 | -1.960671 |
| PSD3 | 0.30832303 | 7.32974618 | 1.99010153 | 0.04996667 | 0.96713357 | -4.1478555 | 1.96024919 |
| FAM222A-AS1 | -0.7425126 | -0.3888729 | -1.9897926 | 0.04999211 | 0.96713357 | -4.1931277 | -1.9600315 |

**Supplementary Table S9:** Differentially Expressed Genes after Psilocybin at 24 Hours

| **Gene Name** | **logFC** | **AveExpr** | **t** | **P.Value** | **adj.P.Val** | **B** | **z.std** |
| --- | --- | --- | --- | --- | --- | --- | --- |
| VSTM1 | 2.76730004 | -1.5775228 | 4.29897609 | 4.8042E-05 | 0.47837487 | 0.20518044 | 4.06495367 |
| ETV7 | 1.98630558 | -1.646434 | 4.03704083 | 0.00012242 | 0.47837487 | -0.3519482 | 3.84123189 |
| HPAT5 | 5.03786059 | -0.9122371 | 4.02613988 | 0.00012308 | 0.47837487 | -0.5041385 | 3.83990303 |
| MLPH | 3.29882769 | -1.8096486 | 3.97968333 | 0.00014718 | 0.47837487 | -0.4029305 | 3.79577208 |
| LINC01993 | 2.55801906 | -0.1271592 | 3.97767204 | 0.00015062 | 0.47837487 | -0.4931995 | 3.79003869 |
| FNDC8 | 1.61910642 | -2.3409946 | 3.95797002 | 0.00015424 | 0.47837487 | -0.5463287 | 3.78414582 |
| IL26 | 2.30117113 | -6.5804954 | 3.94869794 | 0.00016035 | 0.47837487 | -0.3486649 | 3.77447032 |
| GBP2 | 2.8076706 | 0.97009987 | 3.94227457 | 0.0001713 | 0.47837487 | -0.3373351 | 3.75796716 |
| ALPK2 | 2.36945485 | 0.63086661 | 3.8434485 | 0.00023892 | 0.59307765 | -0.5750361 | 3.67385385 |
| MCTP2 | 2.63857713 | 1.03966057 | 3.79317872 | 0.00028033 | 0.61357165 | -0.7760153 | 3.63282839 |
| GBP1 | 2.07649808 | 1.62464706 | 3.75828279 | 0.00032545 | 0.61357165 | -0.8309799 | 3.59415039 |
| HRCT1 | 2.04680505 | -1.8837306 | 3.73447668 | 0.0003543 | 0.61357165 | -0.8190276 | 3.57197337 |
| SPOCD1 | 2.67259013 | 0.17384406 | 3.72459271 | 0.00035753 | 0.61357165 | -0.7487427 | 3.56960026 |
| RNU4-47P | 1.69531549 | -3.876984 | 3.70932684 | 0.0003845 | 0.61357165 | -1.0189284 | 3.55050351 |
| MAL | 1.56243289 | -0.6646216 | 3.67262005 | 0.00042557 | 0.63157506 | -1.1248848 | 3.52369661 |
| BANCR | 2.63814417 | -5.4743063 | 3.64780449 | 0.00045232 | 0.63157506 | -1.0178766 | 3.50751291 |
| SMIM27 | 0.3799702 | 1.56515309 | 3.62978023 | 0.00049084 | 0.63675367 | -1.1621002 | 3.48570612 |
| HLA-H | 1.28964066 | 1.36974075 | 3.62232901 | 0.00051303 | 0.63675367 | -1.0814376 | 3.47386016 |
| STX11 | 1.38800586 | 0.55867188 | 3.54467314 | 0.00064793 | 0.65324075 | -1.3202088 | 3.41071578 |
| MBD3L3 | -1.8806817 | -3.7395876 | -3.5436567 | 0.00065657 | 0.65324075 | -1.3998413 | -3.4070987 |
| SELENOKP1 | -1.3367679 | -1.8566381 | -3.5209242 | 0.00068182 | 0.65324075 | -1.3613263 | -3.396789 |
| APLNR | 2.26326599 | 0.03063994 | 3.53172788 | 0.00068444 | 0.65324075 | -1.2843495 | 3.39573714 |
| HOXA11 | -1.219296 | -5.7811207 | -3.5044394 | 0.00074897 | 0.65324075 | -1.3765842 | -3.3709941 |
| PNN-AS1 | 1.71048935 | -3.3782381 | 3.48328718 | 0.0007893 | 0.65324075 | -1.5432672 | 3.35651844 |
| LINC02669 | 2.40625613 | -4.6752452 | 3.47845533 | 0.0008082 | 0.65324075 | -1.4376765 | 3.34996955 |
| APLF | 0.74272461 | 1.45756828 | 3.47504294 | 0.00083073 | 0.65324075 | -1.4810234 | 3.34234886 |
| DOCK2 | 1.14420018 | -0.9126395 | 3.46868521 | 0.00083293 | 0.65324075 | -1.4882489 | 3.34161182 |
| C3 | 2.93553073 | 0.78233119 | 3.44840035 | 0.00091006 | 0.65324075 | -1.303251 | 3.31695075 |
| LINC00458 | 2.61095506 | -0.9410769 | 3.41359885 | 0.0009854 | 0.65324075 | -1.5606096 | 3.29466334 |
| HTR3B | 2.06962449 | -2.3470379 | 3.40562529 | 0.00102731 | 0.65324075 | -1.7473748 | 3.28293749 |
| LINC02775 | 2.13857153 | -6.2422309 | 3.39875259 | 0.00103197 | 0.65324075 | -1.5197064 | 3.28166257 |
| HOXA9 | -2.192708 | -5.9174621 | -3.4054605 | 0.00103202 | 0.65324075 | -1.5633994 | -3.2816486 |
| HNRNPA1P33 | -2.0603974 | -4.2004122 | -3.3930886 | 0.0010552 | 0.65324075 | -1.7108938 | -3.2753799 |
| APCDD1L | 2.95511173 | -2.1272077 | 3.39109685 | 0.00107054 | 0.65324075 | -1.7024498 | 3.27130199 |
| TENT5B | 1.58038348 | 0.98198355 | 3.39025461 | 0.00107843 | 0.65324075 | -1.487052 | 3.26922482 |
| IL21-AS1 | -2.4039952 | -5.8045289 | -3.3792423 | 0.00110007 | 0.65324075 | -1.5815039 | -3.2635994 |
| CA3 | 1.38786099 | 0.51492866 | 3.37272464 | 0.00114304 | 0.65324075 | -1.6402866 | 3.2527263 |
| IRF2 | 0.35108408 | 3.89051417 | 3.37261663 | 0.00115208 | 0.65324075 | -1.6490428 | 3.25048504 |
| ABCC3 | 1.86851422 | 0.54148157 | 3.35750445 | 0.00120504 | 0.65324075 | -1.5278865 | 3.23768386 |
| PIEZO2 | 1.5420842 | 4.86756445 | 3.34228426 | 0.00125339 | 0.65324075 | -1.7183554 | 3.22644284 |
| CLDN1 | 1.40990411 | 1.96182313 | 3.34343677 | 0.00125399 | 0.65324075 | -1.6260894 | 3.22630735 |
| RELB | 0.92402727 | 3.07444808 | 3.34354108 | 0.00127415 | 0.65324075 | -1.7440878 | 3.2217401 |
| LGSN | 1.81863696 | -2.000136 | 3.32299441 | 0.00133828 | 0.65324075 | -1.7840751 | 3.20764111 |
| ICAM1 | 2.67463059 | 0.84598631 | 3.32279909 | 0.00136258 | 0.65324075 | -1.6162078 | 3.20246361 |
| HOXA10 | -2.0195575 | -5.5333941 | -3.3162129 | 0.00136502 | 0.65324075 | -1.7481515 | -3.2019464 |
| CAV2 | 1.24618384 | 2.98085813 | 3.31670208 | 0.0013716 | 0.65324075 | -1.6503778 | 3.20056098 |
| TGFBI | 2.27488175 | 2.25657607 | 3.29596176 | 0.00145415 | 0.65324075 | -1.6659811 | 3.18368074 |
| PXDNL | 1.88386032 | 2.72947386 | 3.29468692 | 0.00145691 | 0.65324075 | -1.7686208 | 3.18313292 |
| CXCL5 | 2.42245011 | -1.2849032 | 3.29642297 | 0.00146987 | 0.65324075 | -1.6462293 | 3.18056736 |
| FA2H | 1.56447381 | -1.6176331 | 3.28776918 | 0.00150254 | 0.65324075 | -2.0303679 | 3.17419198 |
| LGALS3 | 1.30499767 | 3.89357708 | 3.28699693 | 0.001503 | 0.65324075 | -1.7016219 | 3.17410286 |
| LINC01623 | -1.4792325 | -2.2022777 | -3.2846086 | 0.00152046 | 0.65324075 | -1.8690201 | -3.1707505 |
| ANKRD33 | 1.88002847 | -1.5502999 | 3.27741181 | 0.00158422 | 0.66779209 | -2.0350894 | 3.15879727 |
| LINC01508 | 1.55749548 | 0.86065347 | 3.25606819 | 0.00164502 | 0.68058029 | -1.7982693 | 3.14780437 |
| LINC00861 | -2.3210196 | -5.0144911 | -3.2449733 | 0.00169296 | 0.68767966 | -1.9956237 | -3.1393972 |
| CLPTM1L | 0.24080039 | 6.59739537 | 3.24104251 | 0.0017242 | 0.68786171 | -1.912355 | 3.13403622 |
| LINC00939 | -2.2115442 | -4.8845797 | -3.2236417 | 0.00180703 | 0.70131826 | -1.998142 | -3.1202407 |
| CXCL6 | 1.88631586 | 0.23714581 | 3.22624085 | 0.00182071 | 0.70131826 | -1.7960297 | 3.11801927 |
| MTND5P15 | -1.8396144 | -6.0173454 | -3.2031785 | 0.00192358 | 0.70220208 | -1.9565714 | -3.1017845 |
| MYL12A | 0.68157509 | 4.57247133 | 3.20586835 | 0.00193773 | 0.70220208 | -1.9373395 | 3.09961464 |
| LINC00626 | 1.94156016 | -5.8737413 | 3.19602976 | 0.00196266 | 0.70220208 | -1.9558811 | 3.09582589 |
| C2 | 1.65419028 | 0.60930598 | 3.19908305 | 0.00198934 | 0.70220208 | -1.9732534 | 3.09181897 |
| LINC01358 | -2.3419515 | -4.3023367 | -3.1926294 | 0.00201332 | 0.70220208 | -2.0173271 | -3.088261 |
| CISH | 0.83027029 | 1.06507663 | 3.17404465 | 0.00209906 | 0.70220208 | -1.9807938 | 3.0758461 |
| WNT10A | 1.63612238 | -0.9747805 | 3.17409333 | 0.00211289 | 0.70220208 | -2.2470562 | 3.07388804 |
| LINC01825 | 2.25716653 | -5.584835 | 3.16797572 | 0.00213668 | 0.70220208 | -1.9270154 | 3.07054588 |
| FEV | -2.4430875 | -4.4390991 | -3.1676834 | 0.00214745 | 0.70220208 | -2.081831 | -3.0690438 |
| IL1R1 | 1.68744502 | 2.69330357 | 3.16680387 | 0.00217975 | 0.70220208 | -2.0164771 | 3.06458142 |
| CXCL1 | 2.53308741 | -2.457304 | 3.171249 | 0.00220496 | 0.70220208 | -1.8830829 | 3.06113982 |
| CACNG5 | 1.4015264 | 5.04580976 | 3.15115604 | 0.00224742 | 0.70220208 | -2.0225658 | 3.05542525 |
| RAB42 | 1.02005927 | 1.2277891 | 3.15693182 | 0.00226103 | 0.70220208 | -2.0741196 | 3.05361313 |
| SUGT1P2 | 2.12600507 | -3.9667067 | 3.15460508 | 0.00226304 | 0.70220208 | -2.1552237 | 3.05334728 |
| BIRC3 | 1.32578671 | 0.09255669 | 3.14561017 | 0.00230893 | 0.706629 | -2.0488453 | 3.04731774 |
| PBOV1 | -2.0342831 | -5.877551 | -3.1379867 | 0.00235359 | 0.70685724 | -2.0766211 | -3.0415557 |
| IL15 | 1.62981534 | -1.1385079 | 3.13752456 | 0.00237397 | 0.70685724 | -2.026453 | 3.03895912 |
| CD47 | 0.42492243 | 7.59096396 | 3.12978206 | 0.00244756 | 0.70685724 | -2.0938466 | 3.02975071 |
| TASP1 | 0.24894708 | 3.15057437 | 3.12013949 | 0.00250588 | 0.70685724 | -2.1335471 | 3.02263105 |
| MCM8-AS1 | 1.98377904 | -4.8351253 | 3.10804213 | 0.00252479 | 0.70685724 | -2.1254132 | 3.0203539 |
| PURG | -0.4990777 | 4.09828314 | -3.109911 | 0.00256879 | 0.70685724 | -2.1320176 | -3.0151185 |
| TDH | -1.4711657 | -1.6141487 | -3.0983699 | 0.00260551 | 0.70685724 | -2.2141144 | -3.0108104 |
| RPS26P45 | 2.256747 | -5.7885779 | 3.08639592 | 0.00269666 | 0.70685724 | -2.1517354 | 3.00035373 |
| MALT1 | 0.27104193 | 5.16496657 | 3.09582085 | 0.00270436 | 0.70685724 | -2.1871841 | 2.9994851 |
| RNA5SP490 | -1.7974687 | -4.1457735 | -3.0873436 | 0.00272495 | 0.70685724 | -2.1248164 | -2.9971746 |
| BATF3 | 1.21223582 | 0.80987088 | 3.08541468 | 0.00275468 | 0.70685724 | -2.1396063 | 2.99386501 |
| HOXD8 | -1.7185549 | -4.1830075 | -3.0876676 | 0.00276073 | 0.70685724 | -2.0901179 | -2.9931948 |
| BDKRB2 | 1.81628092 | -1.0479431 | 3.08318417 | 0.00279053 | 0.70685724 | -2.2602211 | 2.98991778 |
| HOXB8 | -2.9257047 | -6.0426122 | -3.0806766 | 0.00280253 | 0.70685724 | -2.1956477 | -2.9886058 |
| DES | 2.31593124 | -1.6716199 | 3.07894871 | 0.00281253 | 0.70685724 | -2.1432134 | 2.98751742 |
| RN7SL236P | 2.16287183 | -5.9637502 | 3.06934098 | 0.00291571 | 0.70685724 | -2.1447205 | 2.97648776 |
| CXCL2 | 1.6958153 | 1.36773817 | 3.08388119 | 0.00295695 | 0.70685724 | -2.0346814 | 2.9721787 |
| DISP1 | 0.36129331 | 4.19389861 | 3.05697619 | 0.00301631 | 0.70685724 | -2.2356071 | 2.96607057 |
| MIR3976HG | 2.08987825 | -5.1973116 | 3.05104586 | 0.00304451 | 0.70685724 | -2.298498 | 2.96320733 |
| C20orf141 | 1.59606389 | -6.8130948 | 3.04336105 | 0.003116 | 0.70685724 | -2.2000224 | 2.95605641 |
| TPST2 | 0.7162069 | 4.62544843 | 3.04486393 | 0.00313007 | 0.70685724 | -2.2359665 | 2.954667 |
| ANPEP | 1.53364928 | -1.6256874 | 3.04415335 | 0.0031505 | 0.70685724 | -2.2687063 | 2.95265869 |
| VAMP5 | 1.35080112 | 2.16227441 | 3.03912675 | 0.0032045 | 0.70685724 | -2.2081704 | 2.94740773 |
| CHCHD3P3 | 1.90790276 | -6.0047692 | 3.00943295 | 0.00339981 | 0.70685724 | -2.2198678 | 2.92906668 |
| PRX | 0.60450742 | 3.29950765 | 3.01626055 | 0.00342112 | 0.70685724 | -2.315401 | 2.92712475 |
| DPH6 | 0.26075426 | 3.0384695 | 3.00665557 | 0.00352318 | 0.70685724 | -2.324395 | 2.91797012 |
| CPNE4 | 0.70910163 | 6.43494092 | 3.00567019 | 0.00352785 | 0.70685724 | -2.3267832 | 2.91755699 |
| MATN3 | 0.90612231 | -0.5900624 | 3.00487942 | 0.0035873 | 0.70685724 | -2.3367869 | 2.91234141 |
| SDR42E1 | -1.9727792 | -5.8503918 | -2.9954031 | 0.00360033 | 0.70685724 | -2.3359934 | -2.9112088 |
| KRT7-AS | 1.79606756 | -6.475876 | 2.9900428 | 0.00361603 | 0.70685724 | -2.2499045 | 2.90984972 |
| CHRNA9 | 2.18433833 | 0.96860329 | 2.99744294 | 0.00363509 | 0.70685724 | -2.3408394 | 2.90820571 |
| DOCK7-DT | 0.53066952 | 1.88131077 | 2.99084459 | 0.00366064 | 0.70685724 | -2.4057332 | 2.9060148 |
| OR3A2 | -1.686259 | -6.3528106 | -2.9903032 | 0.00367185 | 0.70685724 | -2.3006595 | -2.9050583 |
| BCAS1 | 1.80622695 | 0.77170671 | 2.98930878 | 0.0036919 | 0.70685724 | -2.2826573 | 2.90335354 |
| C16orf46-DT | 1.67774254 | -3.3550388 | 2.98612412 | 0.00369532 | 0.70685724 | -2.3984435 | 2.90306312 |
| IGF2 | -2.1838971 | -0.6598495 | -2.9832276 | 0.00374858 | 0.70685724 | -2.3264164 | -2.8985793 |
| PLAT | 1.15689548 | 4.01116966 | 2.98157786 | 0.00377308 | 0.70685724 | -2.3294458 | 2.89653516 |
| STAMBPL1 | 0.58623861 | 3.13957898 | 2.98213202 | 0.00379003 | 0.70685724 | -2.3952515 | 2.89512853 |
| RN7SL330P | -1.5198014 | -6.3020413 | -2.9797701 | 0.00379303 | 0.70685724 | -2.36403 | -2.8948802 |
| HTATIP2 | 0.99558403 | 0.49475154 | 2.97784674 | 0.00383613 | 0.70685724 | -2.3900107 | 2.89133207 |
| HOXC6 | -1.9555463 | -5.9700659 | -2.9715599 | 0.00386563 | 0.70685724 | -2.3615979 | -2.888924 |
| CHODL | 0.88465494 | 4.83985882 | 2.96850964 | 0.00394079 | 0.70685724 | -2.4091697 | 2.88286272 |
| CSRP1 | 0.90866444 | 5.47058068 | 2.96832454 | 0.00394589 | 0.70685724 | -2.3887816 | 2.88245501 |
| FAM242A | -1.7573005 | -5.1916485 | -2.9648603 | 0.0039885 | 0.70685724 | -2.4408845 | -2.8790696 |
| LINC00997 | 0.55044873 | 2.92379249 | 2.96371595 | 0.00401055 | 0.70685724 | -2.4459545 | 2.877331 |
| API5P2 | 2.03688973 | -6.1779235 | 2.95068645 | 0.00404689 | 0.70685724 | -2.3567047 | 2.87448378 |
| MCAM | 0.57983708 | 5.54066708 | 2.95709565 | 0.00408622 | 0.70685724 | -2.4114211 | 2.87142787 |
| CLEC20A | -1.7969076 | -6.0599058 | -2.9504815 | 0.00408641 | 0.70685724 | -2.4114916 | -2.8714129 |
| RCAN1 | 1.16910558 | 7.49960491 | 2.95398532 | 0.0041216 | 0.70685724 | -2.4050046 | 2.86870226 |
| IL6STP1 | 2.06507251 | -5.5970077 | 2.94247666 | 0.0042048 | 0.70685724 | -2.4022599 | 2.86237438 |
| ARHGAP29 | 1.11508006 | 5.14424154 | 2.94457246 | 0.0042129 | 0.70685724 | -2.4238624 | 2.86176464 |
| EHF | 1.66671115 | -1.8298502 | 2.94101242 | 0.00428587 | 0.70685724 | -2.4678134 | 2.85631688 |
| SBSPON | 1.01163085 | 2.06631766 | 2.93827673 | 0.00432008 | 0.70685724 | -2.4351048 | 2.85379201 |
| HMGN1P8 | 1.68586832 | -3.5870699 | 2.92700726 | 0.00440579 | 0.70685724 | -2.5242522 | 2.84754447 |
| RARRES2 | 1.91917087 | 0.00962807 | 2.92759035 | 0.00442424 | 0.70685724 | -2.4363001 | 2.8462146 |
| LINC02970 | 2.17108496 | -5.6240562 | 2.92950045 | 0.00443724 | 0.70685724 | -2.4668537 | 2.84527987 |
| LRRC37A11P | -1.7714463 | -4.1438574 | -2.9196809 | 0.00452848 | 0.70685724 | -2.4381184 | -2.8387913 |
| ASS1 | 1.13427692 | 4.95537136 | 2.91453323 | 0.00459536 | 0.70685724 | -2.4484735 | 2.8341093 |
| ICOSLG | 1.41528109 | -0.3061149 | 2.91367178 | 0.00471105 | 0.70685724 | -2.3539704 | 2.82615487 |
| CBR3 | 0.9179397 | -1.077734 | 2.90712835 | 0.00473726 | 0.70685724 | -2.455918 | 2.82437737 |
| GNAT2 | 0.75880224 | -1.4787381 | 2.90524224 | 0.00475353 | 0.70685724 | -2.5136366 | 2.8232785 |
| CEBPD | 1.25963067 | 4.40367652 | 2.90381776 | 0.00476826 | 0.70685724 | -2.4237546 | 2.82228594 |
| LINC02940 | -1.6785256 | -3.5563269 | -2.8985708 | 0.0047872 | 0.70685724 | -2.4868322 | -2.8210148 |
| EPSTI1 | 1.53268008 | -0.3121127 | 2.90422965 | 0.00478723 | 0.70685724 | -2.4851858 | 2.82101282 |
| RPL7L1P16 | -1.7572304 | -3.622841 | -2.8886075 | 0.00490057 | 0.70685724 | -2.5497669 | -2.8134979 |
| CD207 | -1.8356316 | -6.0008358 | -2.8880457 | 0.00492207 | 0.70685724 | -2.5024883 | -2.8120904 |
| DNAJA2-DT | 0.86361396 | -0.7200942 | 2.88339956 | 0.00493761 | 0.70685724 | -2.5863913 | 2.81107594 |
| PDE8B | 0.68252201 | 4.29508641 | 2.88600813 | 0.00500154 | 0.70685724 | -2.5631365 | 2.80693453 |
| FUT6 | -1.9835 | -4.4449835 | -2.8804953 | 0.00504602 | 0.70685724 | -2.6082797 | -2.804081 |
| KRT10-AS1 | -0.3794468 | 1.56791914 | -2.8806258 | 0.00507121 | 0.70685724 | -2.5136044 | -2.8024755 |
| ZKSCAN8P1 | -0.7946992 | 1.02421681 | -2.8772575 | 0.00508382 | 0.70685724 | -2.5469894 | -2.8016742 |
| ABHD17AP4 | -2.0955637 | -5.7198607 | -2.8722262 | 0.00508877 | 0.70685724 | -2.507358 | -2.8013603 |
| MIR4489 | -1.3296649 | -3.2338591 | -2.8850522 | 0.00509776 | 0.70685724 | -2.5424868 | -2.8007906 |
| CATSPERB | 1.0449517 | -0.8362549 | 2.87951329 | 0.00511196 | 0.70685724 | -2.5190492 | 2.7998925 |
| CCL2 | 1.47499498 | 3.69980997 | 2.88150965 | 0.00512007 | 0.70685724 | -2.4643269 | 2.79938113 |
| STEAP3 | 1.6203238 | 3.39845624 | 2.87585554 | 0.0051368 | 0.70685724 | -2.5364986 | 2.79832764 |
| CALHM2 | 0.5178959 | 1.85982995 | 2.87190662 | 0.00519834 | 0.70685724 | -2.6098138 | 2.79447884 |
| LINC00299 | 1.32695492 | -1.921729 | 2.87321916 | 0.00520084 | 0.70685724 | -2.6767171 | 2.79432379 |
| MIR4285 | -1.6386052 | -5.964975 | -2.8658474 | 0.00524681 | 0.70685724 | -2.5529203 | -2.7914771 |
| RMDN2 | 0.65320262 | 2.46980479 | 2.86629357 | 0.0052844 | 0.70685724 | -2.5901797 | 2.789166 |
| DPY19L1P1 | 0.48410152 | 2.40918407 | 2.86078463 | 0.00538207 | 0.70685724 | -2.5986339 | 2.78322969 |
| RNF145 | 0.41846528 | 7.35025473 | 2.85844173 | 0.00538448 | 0.70685724 | -2.5597274 | 2.78308427 |
| LINC01614 | 2.35002876 | -5.4063968 | 2.85361517 | 0.0054329 | 0.70685724 | -2.5846936 | 2.78017844 |
| LINC02721 | -1.8748312 | -5.5842145 | -2.8534222 | 0.00543372 | 0.70685724 | -2.6011807 | -2.7801295 |
| BET1L | 0.19443171 | 4.80289839 | 2.85831354 | 0.00543705 | 0.70685724 | -2.5896653 | 2.77993053 |
| CARD10 | 0.99593552 | 3.1158085 | 2.85562632 | 0.00545253 | 0.70685724 | -2.5809511 | 2.77900715 |
| GPX5 | -1.7359571 | -5.4573819 | -2.8518461 | 0.00547628 | 0.70685724 | -2.602063 | -2.7775953 |
| TBXA2R | -2.1110459 | -4.2369253 | -2.848809 | 0.00554028 | 0.70685724 | -2.6246404 | -2.7738171 |
| OSBPL2 | -0.1304505 | 6.36670923 | -2.8514228 | 0.00554127 | 0.70685724 | -2.5944726 | -2.7737593 |
| ADAT3 | 0.46599236 | 1.94023245 | 2.84665144 | 0.00554237 | 0.70685724 | -2.5843081 | 2.77369472 |
| CCDC68 | 2.12245499 | -3.6747039 | 2.8500352 | 0.00559526 | 0.70685724 | -2.5528183 | 2.77060315 |
| DRD4 | 1.01108735 | 3.35494903 | 2.84638004 | 0.00562753 | 0.70685724 | -2.5836731 | 2.76872949 |
| RAB32 | 0.74900164 | 2.49264386 | 2.84369481 | 0.00563135 | 0.70685724 | -2.5477255 | 2.76850877 |
| HACD4 | 0.87485973 | 2.89026019 | 2.84450815 | 0.00563931 | 0.70685724 | -2.6491348 | 2.7680483 |
| TMEM239 | 1.69374013 | -6.5378101 | 2.83436053 | 0.00573548 | 0.70685724 | -2.5971924 | 2.76253256 |
| GASK1A | -1.0126822 | -0.8372744 | -2.8327623 | 0.0058083 | 0.70685724 | -2.6244389 | -2.7584119 |
| ITK | 1.96446607 | -1.616111 | 2.8269268 | 0.00588146 | 0.70685724 | -2.6411042 | 2.75431768 |
| MIR3180-4 | -1.5206741 | -4.1409642 | -2.8269256 | 0.00591315 | 0.70685724 | -2.6546055 | -2.7525589 |
| HOXA3 | -1.9454725 | -6.2759006 | -2.8218792 | 0.00594997 | 0.70685724 | -2.6123297 | -2.7505256 |
| LINC02211 | -1.4062822 | -2.2351376 | -2.8200023 | 0.00597558 | 0.70685724 | -2.6476354 | -2.7491183 |
| BTBD18 | 0.7650256 | -0.6988703 | 2.81478488 | 0.00601944 | 0.70685724 | -2.6382536 | 2.7467208 |
| PYCARD | 1.39029291 | -2.7239649 | 2.81972138 | 0.00602776 | 0.70685724 | -2.6873011 | 2.74626737 |
| RBM3 | 0.43520986 | 8.32167835 | 2.81916648 | 0.00604843 | 0.70685724 | -2.6514272 | 2.74514451 |
| GPR34 | 1.71519813 | -2.9929216 | 2.81298739 | 0.00608453 | 0.70685724 | -2.7024387 | 2.74319078 |
| MMP7 | 1.92422396 | -2.6534552 | 2.81678545 | 0.00618956 | 0.70685724 | -2.5355114 | 2.73756678 |
| ADPRH | 0.90122887 | 0.16389876 | 2.81072325 | 0.00621817 | 0.70685724 | -2.6838793 | 2.73604927 |
| RETREG1 | 0.55589636 | 6.07582409 | 2.80630626 | 0.00624892 | 0.70685724 | -2.6487771 | 2.73442586 |
| LINC02136 | -1.8071457 | -5.1587524 | -2.8036841 | 0.00628273 | 0.70685724 | -2.6753906 | -2.7326485 |
| ELAC2 | 0.15769004 | 6.43092568 | 2.80597901 | 0.00629708 | 0.70685724 | -2.6766697 | 2.73189693 |
| LINC02348 | 1.48499469 | -2.4340701 | 2.79908026 | 0.00636858 | 0.70685724 | -2.68658 | 2.72817528 |
| RNU7-186P | 1.50277012 | -6.0481233 | 2.79774864 | 0.0063959 | 0.70685724 | -2.6400313 | 2.72676287 |
| CHI3L1 | 2.58286761 | 1.58672863 | 2.79900032 | 0.00641009 | 0.70685724 | -2.5366174 | 2.72603115 |
| IRAK3 | 0.79795932 | -0.2595728 | 2.80068943 | 0.00641145 | 0.70685724 | -2.686748 | 2.72596162 |
| PRXL2AP2 | -1.816851 | -3.4724816 | -2.7937403 | 0.0064265 | 0.70685724 | -2.6990486 | -2.7251876 |
| ABI3BP | -0.8495354 | 0.97892518 | -2.7981656 | 0.0064303 | 0.70685724 | -2.6387778 | -2.724992 |
| CT70 | 2.0005998 | -4.4481897 | 2.79381152 | 0.00643997 | 0.70685724 | -2.6614179 | 2.72449609 |
| GIMAP2 | -1.8229345 | -6.2135406 | -2.7946936 | 0.00644652 | 0.70685724 | -2.6298739 | -2.7241603 |
| DUSP3 | 0.39015374 | 6.18054828 | 2.79295679 | 0.00647901 | 0.70685724 | -2.6762386 | 2.72249971 |
| LINC02393 | -1.5108118 | -6.0199954 | -2.7930675 | 0.00649682 | 0.70685724 | -2.7049623 | -2.7215924 |
| RPS3AP38 | -0.843982 | -0.871277 | -2.7909023 | 0.00650521 | 0.70685724 | -2.7242659 | -2.7211658 |
| LINC01583 | 1.62639893 | -5.8148263 | 2.78288295 | 0.006572 | 0.70685724 | -2.6386967 | 2.71778767 |
| TNFAIP2 | 1.65628307 | 2.93426949 | 2.78811012 | 0.00658556 | 0.70685724 | -2.6906178 | 2.71710549 |
| SFXN3 | 0.31582947 | 6.47072874 | 2.78548961 | 0.0066118 | 0.70685724 | -2.6988101 | 2.71578946 |
| RAMP3 | 1.39791564 | 0.84938053 | 2.78472619 | 0.00667896 | 0.70685724 | -2.6575269 | 2.71244128 |
| GDF11 | -0.2775684 | 6.79763418 | -2.7828692 | 0.00672287 | 0.70685724 | -2.7102756 | -2.7102688 |
| PRSS23 | 0.72490312 | 5.00692834 | 2.78159954 | 0.0067354 | 0.70685724 | -2.6839579 | 2.70965135 |
| MIR548I2 | -1.2104027 | -6.6024115 | -2.7752794 | 0.00680667 | 0.70685724 | -2.696602 | -2.706158 |
| CPN2 | 1.39671246 | -6.7330086 | 2.77399648 | 0.00682485 | 0.70685724 | -2.6990075 | 2.70527178 |
| FKBP5 | 0.66643715 | 2.45014721 | 2.7746757 | 0.00682973 | 0.70685724 | -2.7075111 | 2.70503435 |
| LEISA1 | 1.0329245 | 2.09142245 | 2.77103449 | 0.00688893 | 0.70685724 | -2.7183339 | 2.70216605 |
| LINC02577 | 1.72663167 | -6.2893682 | 2.76729355 | 0.00696295 | 0.70685724 | -2.6999476 | 2.69861129 |
| IFNGR2 | 0.3582244 | 4.95105489 | 2.76548191 | 0.00697069 | 0.70685724 | -2.7193111 | 2.69824119 |
| EDIL3-DT | 0.97347088 | 1.78238594 | 2.76687037 | 0.00700388 | 0.70685724 | -2.7539423 | 2.69665954 |
| LAMA1 | -0.6170411 | 4.1771304 | -2.7623024 | 0.00710905 | 0.70685724 | -2.7360229 | -2.6916925 |
| LINC02732 | 1.9192377 | -3.3522436 | 2.76003728 | 0.00712687 | 0.70685724 | -2.6620855 | 2.69085721 |
| GCSAM | 1.50856677 | -1.3629754 | 2.76031725 | 0.00713405 | 0.70685724 | -2.8003919 | 2.69052108 |
| UROD | 0.25808295 | 6.08874951 | 2.76013725 | 0.007156 | 0.70685724 | -2.7439488 | 2.68949583 |
| KLRF1 | 1.78938948 | -6.3785412 | 2.75450205 | 0.0071954 | 0.70685724 | -2.7187296 | 2.68766298 |
| ITPRIP-AS1 | -1.7645008 | -4.6424345 | -2.7524725 | 0.00722956 | 0.70685724 | -2.7390622 | -2.6860808 |
| CCN3 | 1.13174798 | 3.61289269 | 2.7551361 | 0.00723514 | 0.70685724 | -2.7133169 | 2.68582302 |
| OPHN1 | -0.4493492 | 6.04368754 | -2.7570748 | 0.00725196 | 0.70685724 | -2.7425704 | -2.6850471 |
| ETV7-AS1 | 1.46941635 | -5.3981963 | 2.75430558 | 0.00726209 | 0.70685724 | -2.7208171 | 2.68458049 |
| TCEAL3-AS1 | 1.65533858 | -3.9676657 | 2.74787316 | 0.00731031 | 0.70685724 | -2.780748 | 2.68236731 |
| LINC02091 | -1.5654066 | -3.1515956 | -2.7501986 | 0.0073536 | 0.70685724 | -2.7610733 | -2.680392 |
| PDE7A-DT | 0.69453937 | -0.5730497 | 2.74824666 | 0.00735751 | 0.70685724 | -2.7899536 | 2.68021383 |
| PPIAP7 | 1.62886501 | -5.8441271 | 2.74482365 | 0.00736869 | 0.70685724 | -2.7743253 | 2.67970587 |
| BANF1P2 | 1.25364438 | -1.4920333 | 2.74371055 | 0.0074841 | 0.70685724 | -2.8722975 | 2.67449881 |
| ITIH6 | -1.7540652 | -5.9424538 | -2.7398427 | 0.00749195 | 0.70685724 | -2.7436492 | -2.6741476 |
| RBM24 | 0.44204426 | 3.65842305 | 2.73735378 | 0.00762028 | 0.70685724 | -2.7852125 | 2.66844704 |
| CP | 2.31617133 | 3.92295268 | 2.7369073 | 0.00763499 | 0.70685724 | -2.7500987 | 2.66779911 |
| GASAL1 | 0.50958523 | -0.3818419 | 2.73429955 | 0.00766219 | 0.70685724 | -2.7887554 | 2.66660418 |
| UPK3BL3P | -0.9125846 | -1.479548 | -2.7346141 | 0.00766542 | 0.70685724 | -2.8278269 | -2.6664624 |
| AATBC | -0.9548855 | 0.0233866 | -2.7341515 | 0.00769988 | 0.70685724 | -2.8465732 | -2.6649544 |
| EP300-AS1 | -0.6871524 | -0.9135726 | -2.7325154 | 0.00770105 | 0.70685724 | -2.8031464 | -2.6649033 |
| DNPEP | 0.29861862 | 5.73510049 | 2.72820224 | 0.0078155 | 0.70685724 | -2.8012441 | 2.65993829 |
| TGFB3-AS1 | -1.6150231 | -4.6381159 | -2.7238309 | 0.00782792 | 0.70685724 | -2.7812507 | -2.6594032 |
| CD74 | 1.60166444 | 2.24833037 | 2.72654584 | 0.00786372 | 0.70685724 | -2.7030824 | 2.65786567 |
| RDH10 | 0.87809037 | 4.93030199 | 2.72584993 | 0.00788802 | 0.70685724 | -2.8005432 | 2.65682564 |
| RLN1 | 1.17006897 | -2.3078703 | 2.72082268 | 0.00789096 | 0.70685724 | -2.8023668 | 2.65670033 |
| PARP12 | 0.88914801 | 2.49780553 | 2.724902 | 0.00789895 | 0.70685724 | -2.7707655 | 2.65635875 |
| SNORA31 | 1.51454682 | -5.1279153 | 2.72105569 | 0.00790099 | 0.70685724 | -2.7945873 | 2.65627193 |
| SPATA1 | 0.73327056 | -1.4595482 | 2.724019 | 0.00790393 | 0.70685724 | -2.7863585 | 2.6561462 |
| SEPSECS-AS1 | -0.6352749 | -0.5061811 | -2.7158383 | 0.00805789 | 0.70685724 | -2.8060918 | -2.6496343 |
| QPCT | 1.21628318 | 2.5703677 | 2.71465257 | 0.00806964 | 0.70685724 | -2.8557133 | 2.649142 |
| SMIM19 | 0.23692591 | 4.38209355 | 2.71402846 | 0.00807393 | 0.70685724 | -2.79303 | 2.64896227 |
| MIR1281 | -1.1139325 | -2.3578538 | -2.7090885 | 0.00809977 | 0.70685724 | -2.8303991 | -2.6478823 |
| LRRIQ4 | -1.872065 | -5.0819078 | -2.7114094 | 0.00811481 | 0.70685724 | -2.8297918 | -2.6472552 |
| ARL9 | 0.80882398 | -0.2816403 | 2.7141409 | 0.0081281 | 0.70685724 | -2.8290678 | 2.6467019 |
| LINC01465 | 0.53548721 | -0.0676222 | 2.71295598 | 0.00815195 | 0.70685724 | -2.8244157 | 2.6457106 |
| TMPRSS7 | 1.75676848 | 1.69210389 | 2.71043376 | 0.00818459 | 0.70685724 | -2.8322842 | 2.6443585 |
| IL32 | 1.47143265 | -0.9362115 | 2.7130332 | 0.00820236 | 0.70685724 | -2.7319738 | 2.64362442 |
| CNMD | 0.99277849 | 0.93538601 | 2.70346178 | 0.00833549 | 0.70685724 | -2.8871249 | 2.63816952 |
| PIGH | 0.2531258 | 4.25895943 | 2.70246877 | 0.00839334 | 0.70685724 | -2.8415008 | 2.63582328 |
| GBP5 | 2.05098795 | -0.5062318 | 2.70059469 | 0.00845208 | 0.70685724 | -2.7973635 | 2.63345586 |
| CDYL | 0.31668056 | 5.0875829 | 2.69961042 | 0.00846916 | 0.70685724 | -2.8434369 | 2.63277017 |
| LY96 | 2.0525721 | -3.5691794 | 2.69722855 | 0.00849049 | 0.70685724 | -2.8370626 | 2.63191595 |
| RASD1 | 0.96305706 | 4.5114925 | 2.69791622 | 0.00849783 | 0.70685724 | -2.8391797 | 2.63162239 |
| SLC36A4 | 0.34830674 | 6.46367355 | 2.69472418 | 0.00852926 | 0.70685724 | -2.8431727 | 2.63036744 |
| ANKRD20A4P | -1.8190002 | -5.6251009 | -2.6949855 | 0.00854203 | 0.70685724 | -2.8416216 | -2.6298589 |
| GPIHBP1 | 2.05697104 | -3.4179573 | 2.69583492 | 0.00856001 | 0.70685724 | -2.891619 | 2.62914395 |
| FAAP24 | 0.31824757 | 2.13017747 | 2.68955701 | 0.00863157 | 0.70685724 | -2.8416103 | 2.62631172 |
| C5AR1 | 1.18877456 | 1.11949259 | 2.69157568 | 0.00864074 | 0.70685724 | -2.7772983 | 2.6259505 |
| FGF19 | -2.5810545 | -3.5606574 | -2.6852684 | 0.00866945 | 0.70685724 | -2.8061458 | -2.624821 |
| KIAA0040 | 1.27209585 | 0.42635905 | 2.68779788 | 0.00873848 | 0.70685724 | -2.8115216 | 2.62211926 |
| C22orf31 | 1.05696718 | -0.7718035 | 2.68487798 | 0.00878202 | 0.70685724 | -2.864849 | 2.62042507 |
| PLEKHF1 | 0.69525199 | 1.25762149 | 2.68563519 | 0.0088024 | 0.70685724 | -2.8066565 | 2.61963477 |
| SP6 | 1.47322753 | 1.10206975 | 2.68245235 | 0.00880961 | 0.70685724 | -2.8375364 | 2.61935562 |
| YIPF1 | 0.28607806 | 4.84535205 | 2.68139376 | 0.00884916 | 0.70685724 | -2.8630496 | 2.61782734 |
| TRIM74 | 0.91390209 | 0.50224091 | 2.68500172 | 0.00885316 | 0.70685724 | -2.9532396 | 2.61767285 |
| PPP1CB | 0.48549717 | 8.10189003 | 2.67955057 | 0.00888667 | 0.70685724 | -2.8645933 | 2.61638331 |
| NAPA | 0.15621868 | 5.90157421 | 2.67856887 | 0.00889049 | 0.70685724 | -2.8652068 | 2.61623662 |
| CELF6 | -1.2233585 | -3.2436929 | -2.6812327 | 0.00890161 | 0.70685724 | -2.8995964 | -2.6158098 |
| SPI1 | 1.90485549 | -3.8310027 | 2.67426355 | 0.00891125 | 0.70685724 | -2.8886759 | 2.61544019 |
| MIR579 | 1.57047792 | -5.9303363 | 2.67761914 | 0.00893466 | 0.70685724 | -2.8792582 | 2.61454419 |
| PBLD | 0.20126458 | 4.7564454 | 2.68055695 | 0.00894021 | 0.70685724 | -2.8818159 | 2.614332 |
| H3C8 | -1.6796296 | -6.2173477 | -2.6759419 | 0.00897796 | 0.70685724 | -2.8674249 | -2.6128922 |
| INAFM1 | 0.48259252 | 5.09234868 | 2.67604627 | 0.00898567 | 0.70685724 | -2.8678758 | 2.61259888 |
| GLRX | 0.46681543 | 3.84497408 | 2.67045559 | 0.00901291 | 0.70685724 | -2.8537047 | 2.61156389 |
| C1S | 1.28155578 | 5.66827481 | 2.67453396 | 0.00909391 | 0.70685724 | -2.8859434 | 2.60850339 |
| OSMR-DT | 1.05971501 | -0.347078 | 2.67531894 | 0.00910583 | 0.70685724 | -2.8519899 | 2.60805507 |
| KCTD9 | 0.55630922 | 4.27076841 | 2.67156604 | 0.00913434 | 0.70685724 | -2.8885703 | 2.60698496 |
| TDGP1 | -1.5148424 | -4.0215509 | -2.667756 | 0.00917534 | 0.70685724 | -2.9480519 | -2.6054511 |
| CD34 | 2.16528914 | 1.653768 | 2.66914291 | 0.00917865 | 0.70685724 | -2.8459718 | 2.60532726 |
| GSX1 | -1.4860991 | 0.74100733 | -2.6691039 | 0.00919828 | 0.70685724 | -2.9504655 | -2.6045954 |
| IL13RA1 | 0.9241005 | 4.64825419 | 2.6683124 | 0.00922957 | 0.70685724 | -2.8697845 | 2.60343144 |
| ACVRL1 | 1.63028904 | -2.7219264 | 2.66000847 | 0.00939563 | 0.70685724 | -2.971853 | 2.5973128 |
| ZNF665 | 0.30876112 | 2.43551221 | 2.65940634 | 0.00940994 | 0.70685724 | -2.9021087 | 2.59679018 |
| FITM1 | 0.78822313 | -1.1901691 | 2.66072816 | 0.00945879 | 0.70685724 | -2.9360564 | 2.59501082 |
| TNRC6B-DT | -1.4051276 | -2.9915264 | -2.6523091 | 0.00947963 | 0.70685724 | -2.9859715 | -2.5942543 |
| BECN1 | 0.19175393 | 5.48390453 | 2.65697392 | 0.00952051 | 0.70685724 | -2.9161115 | 2.59277484 |
| PSIP1P1 | -1.0105509 | -2.276883 | -2.6517417 | 0.00952416 | 0.70685724 | -2.8883709 | -2.5926429 |
| SMC2-DT | 0.97873286 | -1.5981911 | 2.65383506 | 0.00956035 | 0.70685724 | -2.9589293 | 2.5913383 |
| OSBPL3 | 0.59660964 | 4.72664001 | 2.65086468 | 0.00965051 | 0.70685724 | -2.9192701 | 2.58810671 |
| KCTD11 | 0.29764207 | 3.98061546 | 2.65099234 | 0.00966135 | 0.70685724 | -2.9099968 | 2.58771977 |
| SPP2 | 1.53967576 | -5.9047879 | 2.64995095 | 0.00966239 | 0.70685724 | -2.9313287 | 2.58768285 |
| CLEC18B | 1.06215698 | 3.47362837 | 2.64946102 | 0.00970784 | 0.70685724 | -2.9134228 | 2.58606595 |
| GS1-24F4.2 | -1.6106119 | -3.0262148 | -2.6474936 | 0.00971355 | 0.70685724 | -2.9420519 | -2.5858631 |
| CFAP107 | -0.9645877 | 1.13803196 | -2.6481669 | 0.00972171 | 0.70685724 | -2.8601258 | -2.5855737 |
| CAAP1 | 0.22329483 | 4.45454642 | 2.64692913 | 0.00976985 | 0.70685724 | -2.9331379 | 2.58387025 |
| LHX8 | -2.6479284 | -3.9768013 | -2.645482 | 0.009777 | 0.70685724 | -3.0317448 | -2.5836182 |
| PLB1 | 0.84732918 | 2.63609962 | 2.64390169 | 0.00982745 | 0.70685724 | -2.9305984 | 2.5818422 |
| FOXF2 | 1.27483789 | -0.5087412 | 2.64465773 | 0.00983562 | 0.70685724 | -2.8819252 | 2.58155525 |
| RPL23AP49 | -0.6523309 | -0.462866 | -2.6432476 | 0.00985156 | 0.70685724 | -2.9663767 | -2.5809964 |
| THORLNC | 0.90891826 | -1.7578464 | 2.63915946 | 0.00988843 | 0.70685724 | -2.9528282 | 2.57970673 |
| CRABP2 | 0.43764362 | 3.00893272 | 2.64191527 | 0.00989825 | 0.70685724 | -2.9196854 | 2.5793639 |
| CYP1B1-AS1 | 0.97170869 | -0.5395124 | 2.63998107 | 0.00994438 | 0.70685724 | -2.9242043 | 2.57775736 |
| SLC22A4 | 0.67981692 | 2.88557205 | 2.63852432 | 0.00995028 | 0.70685724 | -2.9262271 | 2.57755233 |
| ENPP1 | 0.66463176 | 1.84020542 | 2.63991084 | 0.00995589 | 0.70685724 | -2.9542297 | 2.57735751 |
| FMN1 | 1.05644955 | -0.4820185 | 2.63561636 | 0.01003063 | 0.70685724 | -2.9302513 | 2.5747717 |
| A2M | 2.01202916 | 6.21128908 | 2.63458697 | 0.0101241 | 0.70685724 | -2.9191876 | 2.57156168 |
| LINC01503 | 0.678697 | 1.64398453 | 2.63027606 | 0.01017076 | 0.70685724 | -2.9489142 | 2.56996904 |
| PRG4 | -1.0658508 | -2.3902667 | -2.6315311 | 0.0101735 | 0.70685724 | -2.9384612 | -2.5698757 |
| FAM87B | 0.95251959 | 0.057661 | 2.6317676 | 0.01017948 | 0.70685724 | -2.9659692 | 2.56967228 |
| ITPRID2-DT | 1.05259129 | -1.5862182 | 2.62795227 | 0.01020013 | 0.70685724 | -2.9962827 | 2.56897008 |
| GALK1 | 0.34862993 | 3.98345546 | 2.63149328 | 0.01020075 | 0.70685724 | -2.9360857 | 2.56894893 |
| BACE2 | 0.87871345 | 3.77721876 | 2.63025916 | 0.01021298 | 0.70685724 | -2.8973901 | 2.56853365 |
| JPH2 | 1.16815173 | 0.52242726 | 2.6294532 | 0.01024488 | 0.70685724 | -2.967176 | 2.56745273 |
| NFKBIA | 0.64471682 | 4.78158344 | 2.62953987 | 0.01026459 | 0.70685724 | -2.9460482 | 2.56678597 |
| CCDC192 | 0.60504723 | -1.1388387 | 2.6287439 | 0.01027847 | 0.70685724 | -2.9245381 | 2.56631763 |
| TEDC2-AS1 | -0.9609869 | -0.9717114 | -2.6216946 | 0.01031123 | 0.70685724 | -2.9603603 | -2.5652136 |
| NADK | 0.23539666 | 6.4417219 | 2.62339999 | 0.01032592 | 0.70685724 | -2.9465066 | 2.56471964 |
| LINC00987 | 0.69506586 | -0.7320065 | 2.61602896 | 0.0105061 | 0.70685724 | -2.9569039 | 2.55871126 |
| C5orf58 | 1.81866255 | -4.6001992 | 2.61514198 | 0.01053157 | 0.70685724 | -2.9381284 | 2.55786914 |
| CA3-AS1 | 0.50649477 | 1.29673735 | 2.61892733 | 0.01054907 | 0.70685724 | -2.9707675 | 2.5572919 |
| GOLGA6C | 1.37038583 | -2.0470987 | 2.61631422 | 0.01059346 | 0.70685724 | -3.0480089 | 2.55583087 |
| CLP1 | 0.25080464 | 3.08851153 | 2.61479999 | 0.01060996 | 0.70685724 | -2.9813432 | 2.55528923 |
| LINC00681 | -1.9782599 | -4.9801007 | -2.6131952 | 0.01062693 | 0.70685724 | -2.9335961 | -2.5547328 |
| ZNF233 | -0.4547516 | 1.42483629 | -2.6152639 | 0.0106531 | 0.70685724 | -3.0031072 | -2.5538765 |
| AFP | 1.05986266 | -2.5262568 | 2.6126918 | 0.01069927 | 0.70685724 | -2.9425302 | 2.55237048 |
| SERPING1 | 1.07065356 | 4.61375338 | 2.61332351 | 0.01070296 | 0.70685724 | -2.9612845 | 2.55225038 |
| IL18R1 | 1.51145386 | -0.1142294 | 2.61022184 | 0.01074277 | 0.70685724 | -2.967704 | 2.55095667 |
| C1QTNF6 | 0.92102415 | 5.37577638 | 2.61152353 | 0.0107781 | 0.70685724 | -2.9755929 | 2.54981213 |
| MMP19 | 1.19112106 | 1.92235479 | 2.61103544 | 0.01081315 | 0.70685724 | -2.9173728 | 2.54867987 |
| FAM181B | -0.518712 | 5.46224013 | -2.6089313 | 0.01081557 | 0.70685724 | -2.9798792 | -2.5486018 |
| KREMEN1 | 0.44491368 | 6.09612751 | 2.60738378 | 0.01085942 | 0.70685724 | -2.9851516 | 2.54719043 |
| P2RX4 | 0.35685632 | 3.54749562 | 2.6081081 | 0.01086595 | 0.70685724 | -2.9844553 | 2.54698047 |
| NPS | 1.88302506 | -4.4529135 | 2.60441509 | 0.01086885 | 0.70685724 | -2.9332884 | 2.54688725 |
| IL4I1 | 1.62063006 | -2.5817863 | 2.60389016 | 0.01090611 | 0.70685724 | -2.9988941 | 2.54569283 |
| PCDH8 | 0.70452523 | 6.5857073 | 2.60388185 | 0.01092721 | 0.70685724 | -2.9894483 | 2.54501812 |
| LSG1 | 0.16353319 | 5.70982876 | 2.60291513 | 0.01099371 | 0.70685724 | -2.9995828 | 2.54289866 |
| PCSK6 | 0.96877267 | 3.50746129 | 2.60329904 | 0.01101244 | 0.70685724 | -3.0160106 | 2.54230396 |
| NDUFA3P1 | -1.5477685 | -4.2239139 | -2.5971722 | 0.01106309 | 0.70685724 | -3.0117272 | -2.5406997 |
| BLVRA | 0.27461954 | 4.63008703 | 2.60060035 | 0.01106785 | 0.70685724 | -2.9878109 | 2.54054918 |
| QPCTL | 0.37774069 | 3.87311887 | 2.60046029 | 0.01107411 | 0.70685724 | -3.0090509 | 2.54035158 |
| ETF1 | 0.1993355 | 6.73548473 | 2.59885497 | 0.011085 | 0.70685724 | -2.9888974 | 2.54000763 |
| NFKB1 | 0.55170567 | 4.36603264 | 2.59975837 | 0.0111043 | 0.70685724 | -3.0032949 | 2.53939926 |
| MIR22HG | 0.55740115 | 3.47698844 | 2.60078385 | 0.01112314 | 0.70685724 | -3.0234616 | 2.5388064 |
| AIMP2 | 0.26878245 | 3.9175104 | 2.59610218 | 0.01113319 | 0.70685724 | -2.9944157 | 2.53849041 |
| FURIN | 0.29255521 | 6.30036021 | 2.59807133 | 0.01116044 | 0.70685724 | -3.0055797 | 2.53763459 |
| OR7C1 | -1.658752 | -4.7358704 | -2.5969671 | 0.01117085 | 0.70685724 | -3.0005088 | -2.5373084 |
| NAAA | 0.31067501 | 3.28893278 | 2.59797941 | 0.01117359 | 0.70685724 | -3.0138158 | 2.53722266 |
| HNRNPRP1 | -1.8638323 | -4.8273321 | -2.5939171 | 0.0111831 | 0.70685724 | -2.986521 | -2.5369249 |
| IER3 | 0.64002141 | 1.88049779 | 2.59684231 | 0.01119251 | 0.70685724 | -2.9658081 | 2.53663014 |
| GIN1 | -0.2183521 | 2.58741212 | -2.5929252 | 0.01119953 | 0.70685724 | -2.9950069 | -2.5364106 |
| WASL-DT | 0.28262271 | 3.27893681 | 2.59394634 | 0.01120383 | 0.70685724 | -2.9952975 | 2.53627625 |
| LINC01894 | -1.1267633 | -1.9829883 | -2.5948858 | 0.01121393 | 0.70685724 | -2.9949382 | -2.5359607 |
| ACTG1P12 | 1.71406978 | -5.7159726 | 2.58719696 | 0.01138367 | 0.70685724 | -2.9950977 | 2.53069519 |
| DRAM1 | 0.68016401 | 3.32569767 | 2.58897722 | 0.01142205 | 0.70685724 | -3.0001416 | 2.52951431 |
| LPCAT3 | 0.35106963 | 3.62543216 | 2.58815013 | 0.01142398 | 0.70685724 | -3.0119435 | 2.52945516 |
| RBM26-AS1 | 0.39108931 | 3.61691218 | 2.58598058 | 0.01148655 | 0.70685724 | -3.0207835 | 2.52753775 |
| RNU6-969P | -1.7645626 | -4.7263258 | -2.5830056 | 0.01149976 | 0.70685724 | -2.9968845 | -2.5271341 |
| LBX2-AS1 | 1.03606568 | -2.2237545 | 2.586287 | 0.01155902 | 0.70685724 | -3.031704 | 2.52532858 |
| CTHRC1 | 1.34754807 | 1.01713817 | 2.582535 | 0.01156118 | 0.70685724 | -3.0853481 | 2.5252628 |
| CPNE2-DT | -1.6947339 | -4.0887668 | -2.5815479 | 0.01158812 | 0.70685724 | -3.0287071 | -2.5244449 |
| TM9SF1 | 0.39391163 | 2.96159209 | 2.58327392 | 0.01159252 | 0.70685724 | -3.0248403 | 2.52431122 |
| HSP90AA2P | -0.8506335 | 0.68118519 | -2.580057 | 0.01159376 | 0.70685724 | -3.0178412 | -2.5242739 |
| LRRC37A | -0.3466001 | 1.68975659 | -2.5810415 | 0.01163046 | 0.70685724 | -3.0544357 | -2.5231626 |
| PPP6R2 | 0.14889877 | 7.07238357 | 2.57895498 | 0.0116557 | 0.70685724 | -3.0251473 | 2.52240021 |
| EPCAM-DT | -0.5567808 | 1.11106135 | -2.5786741 | 0.01168595 | 0.70685724 | -3.0098713 | -2.5214884 |
| PLS3 | 0.84107331 | 7.2711085 | 2.57696533 | 0.01179315 | 0.70685724 | -3.0362016 | 2.51827398 |
| RNU6ATAC16P | 1.23914515 | -3.963212 | 2.573561 | 0.01181516 | 0.70685724 | -3.0616214 | 2.51761723 |
| PIN1-DT | -1.5568042 | -3.5400793 | -2.5732554 | 0.01182058 | 0.70685724 | -3.0123951 | -2.5174559 |
| TMEM217 | 0.55897662 | -0.0678643 | 2.57582567 | 0.01182128 | 0.70685724 | -3.0096999 | 2.51743487 |
| FPGS | 0.22527723 | 5.87334204 | 2.57635495 | 0.01182824 | 0.70685724 | -3.0377695 | 2.51722771 |
| LINC01044 | 1.65333788 | -6.1365201 | 2.57176741 | 0.01183402 | 0.70685724 | -3.0432908 | 2.51705542 |
| RPL21P54 | -0.8583657 | -1.9801453 | -2.5729911 | 0.01183728 | 0.70685724 | -3.055897 | -2.5169585 |
| PSMA5 | 0.1960322 | 5.77088169 | 2.56881268 | 0.01189637 | 0.70685724 | -3.0177717 | 2.51520346 |
| CTRB2 | 1.51525722 | -2.7175936 | 2.57170566 | 0.01194798 | 0.70685724 | -3.0815768 | 2.51367715 |
| SDC4 | 1.22963026 | 2.84411279 | 2.56839689 | 0.01209154 | 0.70685724 | -3.0077088 | 2.50946136 |
| TBX4 | -1.4383033 | -4.3992567 | -2.5650601 | 0.01215241 | 0.70685724 | -3.0999875 | -2.5076875 |
| IFITM1 | 2.1531433 | 3.90353151 | 2.5671309 | 0.01216208 | 0.70685724 | -3.0396775 | 2.50740639 |
| TRIM75 | 1.52062713 | -6.3392815 | 2.55944167 | 0.01216242 | 0.70685724 | -2.9917994 | 2.50739642 |
| LINC02015 | 2.21070763 | -5.0970247 | 2.56263133 | 0.0121744 | 0.70685724 | -3.0570966 | 2.50704849 |
| SOCS5P2 | -1.3482018 | -3.1390071 | -2.5611152 | 0.01220462 | 0.70685724 | -3.0445516 | -2.5061721 |
| AFG1L | -0.1958873 | 3.12495015 | -2.5634439 | 0.01221549 | 0.70685724 | -3.0420446 | -2.5058574 |
| DAZL | -1.4884857 | -2.3066773 | -2.5631071 | 0.01222297 | 0.70685724 | -3.1101714 | -2.5056409 |
| NAMPT | 0.72514945 | 6.62789291 | 2.56315186 | 0.01225579 | 0.70685724 | -3.0534762 | 2.50469253 |
| IL34 | 0.95914525 | 1.96376999 | 2.56039669 | 0.01227925 | 0.70685724 | -3.076129 | 2.50401609 |
| TC2N | 0.58312258 | 5.2588953 | 2.56219482 | 0.01228107 | 0.70685724 | -3.0526201 | 2.50396337 |
| FRZB | 1.19385011 | 2.93709319 | 2.5623722 | 0.01228704 | 0.70685724 | -3.0574102 | 2.50379166 |
| PORCN | 0.2432303 | 5.11770426 | 2.55883325 | 0.01231824 | 0.70685724 | -3.0646695 | 2.50289418 |
| ITPRIPL1 | 1.14295406 | 0.3681032 | 2.55805583 | 0.01235041 | 0.70685724 | -3.0834041 | 2.50197089 |
| VPREB3 | 1.32025923 | -2.9762922 | 2.55355729 | 0.01237243 | 0.70685724 | -3.0458983 | 2.50134013 |
| COL13A1 | 1.56308858 | 0.06697679 | 2.55523024 | 0.01239357 | 0.70685724 | -3.0355574 | 2.50073539 |
| ZNRF2P1 | 0.45888635 | 3.36089674 | 2.55714356 | 0.01242806 | 0.70685724 | -3.0708514 | 2.49975121 |
| IL17RB | -0.2520417 | 3.44257826 | -2.5570117 | 0.01243817 | 0.70685724 | -3.0816569 | -2.4994631 |
| NGF | -1.9695759 | -3.8331276 | -2.5558058 | 0.012458 | 0.70685724 | -3.1670665 | -2.4988984 |
| GRWD1 | 0.18081577 | 5.28389086 | 2.55099314 | 0.01245859 | 0.70685724 | -3.0494921 | 2.49888164 |
| TOMM20L | 0.50384997 | -0.7053319 | 2.55466533 | 0.01246101 | 0.70685724 | -3.0709394 | 2.49881299 |
| HOXC9 | -1.6416679 | -5.6123615 | -2.5541747 | 0.01249527 | 0.70685724 | -3.0395229 | -2.4978395 |
| H19 | -1.6299731 | -1.671343 | -2.5523403 | 0.0125847 | 0.70685724 | -3.0116684 | -2.4953103 |
| KYAT3 | 0.27330754 | 5.19056415 | 2.55309667 | 0.01259065 | 0.70685724 | -3.069887 | 2.49514275 |
| PARVA | 0.29378144 | 5.90386349 | 2.55276637 | 0.01259783 | 0.70685724 | -3.0720194 | 2.49494037 |
| SLC25A13 | 0.26842223 | 4.44300719 | 2.55115519 | 0.01262016 | 0.70685724 | -3.0572584 | 2.494312 |
| BMPR1AP2 | -1.5794712 | -3.9760401 | -2.549928 | 0.01262806 | 0.70685724 | -3.0917133 | -2.4940898 |
| DNASE1L2 | -0.4797435 | 1.80785462 | -2.5515883 | 0.01264551 | 0.70685724 | -3.1059141 | -2.4935996 |
| TNXB | 0.46349031 | 4.28741566 | 2.54953726 | 0.01272087 | 0.70685724 | -3.1041795 | 2.49148955 |
| CRTC3-AS1 | 0.55866252 | -1.0197983 | 2.54387432 | 0.01276271 | 0.70685724 | -3.0636615 | 2.49032287 |
| MIR3142HG | 1.72353716 | -6.0845772 | 2.54370325 | 0.01280198 | 0.70685724 | -3.0378279 | 2.48923075 |
| DNAJB5-DT | -1.6101681 | -4.3667374 | -2.542466 | 0.01284118 | 0.70685724 | -3.0817538 | -2.4881439 |
| DIRAS3 | 1.12115122 | 5.37341361 | 2.54365794 | 0.01286798 | 0.70685724 | -3.0965268 | 2.48740225 |
| IFITM2 | 1.50368145 | 3.38940204 | 2.54180609 | 0.01293847 | 0.70685724 | -3.0546279 | 2.4854584 |
| ATP1A2 | -0.9927009 | 8.72636833 | -2.5394193 | 0.01296438 | 0.70685724 | -3.0879072 | -2.4847462 |
| CKAP4 | 0.30110041 | 7.02080611 | 2.53935589 | 0.01301357 | 0.70685724 | -3.0901675 | 2.48339763 |
| ISG20 | 0.99118463 | 0.31308358 | 2.54006826 | 0.01305531 | 0.70685724 | -3.0650097 | 2.48225707 |
| ARHGAP23P1 | 1.86706738 | -5.4063876 | 2.53419675 | 0.01306649 | 0.70685724 | -3.0965364 | 2.48195194 |
| CTSS | 1.41141432 | 1.36178399 | 2.53996066 | 0.0130763 | 0.70685724 | -3.0488418 | 2.48168453 |
| ADGRL3-AS1 | 1.62581864 | -5.2613108 | 2.5344234 | 0.01311264 | 0.70685724 | -3.0909718 | 2.48069524 |
| WWC2-AS2 | 0.874319 | -1.1866933 | 2.5378137 | 0.01312941 | 0.70685724 | -3.0776858 | 2.48023973 |
| ABCC2 | 0.7518989 | -0.5046462 | 2.53648636 | 0.01318115 | 0.70685724 | -3.0908842 | 2.47883713 |
| ARV1 | 0.22624322 | 4.38238942 | 2.5347999 | 0.01318226 | 0.70685724 | -3.0874827 | 2.47880711 |
| NUAK2 | 0.613315 | 3.8400967 | 2.53483583 | 0.0132007 | 0.70685724 | -3.0719866 | 2.47830843 |
| SGPP2 | 0.65432372 | 4.38212493 | 2.53300177 | 0.01322672 | 0.70685724 | -3.0959046 | 2.47760573 |
| ZKSCAN3 | -0.2243236 | 3.97935096 | -2.5294425 | 0.01331441 | 0.70685724 | -3.09383 | -2.4752469 |
| ABCA1 | 0.43132843 | 6.76233145 | 2.53115347 | 0.01332411 | 0.70685724 | -3.104983 | 2.47498672 |
| HOXC13 | -1.5671439 | -6.4834586 | -2.5289628 | 0.01334939 | 0.70685724 | -3.1001698 | -2.4743097 |
| DYNLL1P4 | 1.74376634 | -4.9914137 | 2.52230891 | 0.01343938 | 0.70685724 | -3.108133 | 2.47190873 |
| BCDIN3D | 0.21894053 | 2.80609249 | 2.52517867 | 0.01348164 | 0.70685724 | -3.1145082 | 2.47078628 |
| TRIM58 | -0.658249 | 2.26074459 | -2.5252681 | 0.01348798 | 0.70685724 | -3.1445089 | -2.4706181 |
| NFKBIZ | 0.82635482 | 2.89580317 | 2.52444176 | 0.01355941 | 0.70685724 | -3.0718819 | 2.46872842 |
| SNHG4 | 0.6374963 | 0.62466185 | 2.52265412 | 0.0136227 | 0.70685724 | -3.1629283 | 2.46706142 |
| PLSCR1 | 0.73484461 | 2.76519896 | 2.52240917 | 0.01364311 | 0.70685724 | -3.0629614 | 2.4665252 |
| FTLP12 | 1.31744711 | -2.6866879 | 2.51850776 | 0.01371573 | 0.70685724 | -3.1421083 | 2.4646234 |
| CABP4 | 1.13557221 | -1.5769275 | 2.51202518 | 0.01381606 | 0.70685724 | -3.1597857 | 2.4620106 |
| DLK1 | 1.7130847 | 9.39301952 | 2.51740359 | 0.01382108 | 0.70685724 | -3.1308807 | 2.46188022 |
| DUOXA2 | -1.4718403 | -4.027478 | -2.5131037 | 0.01389378 | 0.70685724 | -3.1448562 | -2.4599981 |
| B4GALT4 | 0.35734695 | 5.2897744 | 2.51504744 | 0.0139015 | 0.70685724 | -3.1317927 | 2.45979867 |
| PLP2 | 0.68821633 | 3.12897872 | 2.51487825 | 0.01392116 | 0.70685724 | -3.0941415 | 2.45929128 |
| SMCO2 | -1.0728905 | -2.8601102 | -2.5121732 | 0.01392969 | 0.70685724 | -3.0990166 | -2.4590715 |
| POU5F1 | -1.0779997 | -2.5856505 | -2.5108598 | 0.0139642 | 0.70685724 | -3.173873 | -2.4581831 |
| HMBS | 0.23024911 | 4.48791833 | 2.51194937 | 0.01400427 | 0.70685724 | -3.1336287 | 2.45715387 |
| PGBD2 | -0.293172 | 2.94789155 | -2.5077543 | 0.01408354 | 0.70685724 | -3.1394994 | -2.4551256 |
| GBP1P1 | 1.68663774 | -5.7531187 | 2.50993301 | 0.01411983 | 0.70685724 | -3.1032305 | 2.45420044 |
| SLC44A1 | 0.32181234 | 6.64738194 | 2.50928576 | 0.01412101 | 0.70685724 | -3.1408212 | 2.45417022 |
| WBP2P1 | -1.3759501 | -6.6368643 | -2.5056399 | 0.01413723 | 0.70685724 | -3.0970076 | -2.4537576 |
| AQP7P2 | 1.879291 | -5.2068835 | 2.506116 | 0.01415317 | 0.70685724 | -3.1309796 | 2.45335206 |
| NT5E | 0.83749117 | 3.58202322 | 2.50712151 | 0.01417512 | 0.70685724 | -3.1079054 | 2.45279476 |
| HOXB2 | -1.2614779 | -2.856036 | -2.5052023 | 0.01424143 | 0.70685724 | -3.1311005 | -2.4511153 |
| SLC30A7 | 0.23663603 | 5.14886117 | 2.50541151 | 0.01426656 | 0.70685724 | -3.1554584 | 2.45048077 |
| NAT16 | 0.65133365 | 4.52630941 | 2.50234991 | 0.01432182 | 0.70685724 | -3.1797316 | 2.44908869 |
| STAT5A | 0.90341018 | 2.57839257 | 2.50337327 | 0.0143318 | 0.70685724 | -3.1629734 | 2.44883774 |
| PXT1 | 0.84384667 | -1.897949 | 2.50039959 | 0.01436711 | 0.70685724 | -3.1908601 | 2.44795122 |
| COX20P2 | -1.7623013 | -5.4481148 | -2.4965205 | 0.01437291 | 0.70685724 | -3.1443804 | -2.4478059 |
| DCAF4 | 0.25118996 | 3.8322467 | 2.50007679 | 0.01437961 | 0.70685724 | -3.1662887 | 2.44763778 |
| JAK3 | 0.69153538 | 0.91821662 | 2.49992891 | 0.01439361 | 0.70685724 | -3.1735996 | 2.4472872 |
| NIPAL2 | 0.77395312 | 2.68623571 | 2.49972281 | 0.01444331 | 0.70685724 | -3.1661349 | 2.44604472 |
| LINC02009 | 1.83085187 | -6.0158757 | 2.49593708 | 0.01448235 | 0.70685724 | -3.1381492 | 2.44507125 |
| DCPS | 0.19414804 | 4.20714885 | 2.49909166 | 0.01450318 | 0.70685724 | -3.1530593 | 2.444553 |
| LINC01019 | 1.87579677 | -3.9767131 | 2.49606575 | 0.01451978 | 0.70685724 | -3.1755238 | 2.44414017 |
| DYNLT3 | 0.42894646 | 3.62466414 | 2.49805145 | 0.01452246 | 0.70685724 | -3.1344277 | 2.44407381 |
| TUBA1C | 0.35960413 | 4.65620636 | 2.49861822 | 0.01455219 | 0.70685724 | -3.1431153 | 2.4433357 |
| ACKR4 | -1.0861927 | -2.4489418 | -2.4998099 | 0.01455834 | 0.70685724 | -3.1733203 | -2.4431834 |
| SOCS1 | 0.59423586 | 0.82546113 | 2.49291667 | 0.01460972 | 0.70685724 | -3.1419779 | 2.44191168 |
| RMDN1 | 0.17383212 | 6.02816708 | 2.4950249 | 0.0146519 | 0.70685724 | -3.1599785 | 2.44087072 |
| CLEC2B | 1.48232413 | -2.2572802 | 2.49311413 | 0.01465685 | 0.70685724 | -3.1396993 | 2.4407486 |
| MYO3B-AS1 | -1.454622 | -5.9821162 | -2.4927057 | 0.01467311 | 0.70685724 | -3.17508 | -2.4403482 |
| GSTO1 | 0.37350846 | 3.89840997 | 2.49450243 | 0.01468642 | 0.70685724 | -3.1421622 | 2.4400206 |
| FBLN5 | 1.04377652 | 8.96950103 | 2.49389779 | 0.01468811 | 0.70685724 | -3.1617417 | 2.43997924 |
| PRPH | -0.9250734 | -1.5943002 | -2.4949283 | 0.01470034 | 0.70685724 | -3.1548509 | -2.4396785 |
| SPX | -0.6712006 | 0.13908861 | -2.4927787 | 0.01470354 | 0.70685724 | -3.1544823 | -2.4395998 |
| PODXL | 0.80073833 | 6.84746306 | 2.49239004 | 0.01471931 | 0.70685724 | -3.152818 | 2.43921261 |
| MREG | 0.67667355 | 3.94154413 | 2.49205092 | 0.01473748 | 0.70685724 | -3.1433888 | 2.43876671 |
| GYPC | 1.05098029 | 2.3831307 | 2.49062366 | 0.01476783 | 0.70685724 | -3.1512588 | 2.43802303 |
| ERVFRD-1 | 1.52306174 | -6.2486229 | 2.48806801 | 0.0147793 | 0.70685724 | -3.1474446 | 2.43774246 |
| FOXE1 | -1.6765666 | -3.4125775 | -2.4855573 | 0.01486771 | 0.70685724 | -3.1792037 | -2.4355855 |
| SIRT4 | -0.3345189 | 0.69652461 | -2.4889049 | 0.01486925 | 0.70685724 | -3.1772778 | -2.435548 |
| STAP2 | 0.75937577 | 1.49983783 | 2.48752705 | 0.01490184 | 0.70685724 | -3.1811922 | 2.43475592 |
| SLC9A1 | 0.2323249 | 5.58122881 | 2.48663478 | 0.01490219 | 0.70685724 | -3.179912 | 2.43474744 |
| KCNK13 | 1.5018911 | 1.68115358 | 2.48417227 | 0.01504214 | 0.7084335 | -3.1841555 | 2.43136283 |
| DHX32 | 0.2063022 | 4.85214561 | 2.48233866 | 0.01514317 | 0.7084335 | -3.1821028 | 2.42893692 |
| GLP2R | 1.85163622 | -4.5242476 | 2.48008252 | 0.01516509 | 0.7084335 | -3.169906 | 2.42841242 |
| LINC02470 | -1.1320675 | -6.8641699 | -2.4800881 | 0.01518709 | 0.7084335 | -3.185054 | -2.4278865 |
| MAMDC2 | 2.00079758 | 5.09753914 | 2.48336259 | 0.01520296 | 0.7084335 | -3.2102618 | 2.42750773 |
| KCNJ14 | 0.34739999 | 2.15530508 | 2.48070498 | 0.01522422 | 0.7084335 | -3.2109487 | 2.42700077 |
| LASP1NB | -0.4893907 | 1.38225659 | -2.4802694 | 0.01523869 | 0.7084335 | -3.1870738 | -2.4266561 |
| OXT | 1.80161942 | -4.0957355 | 2.47689416 | 0.01526016 | 0.7084335 | -3.2361131 | 2.42614521 |
| GAPLINC | 1.7465562 | -5.2195085 | 2.47680023 | 0.01526582 | 0.7084335 | -3.1779474 | 2.42601063 |
| IFI16 | 0.75910618 | 4.29722845 | 2.47660115 | 0.01540659 | 0.7084335 | -3.1610717 | 2.42267768 |
| UNC93A | 2.21622041 | -4.4497613 | 2.47366101 | 0.01543024 | 0.7084335 | -3.1883798 | 2.42212036 |
| ARL14EPL | 1.74800412 | -5.4266368 | 2.47098017 | 0.01547904 | 0.7084335 | -3.1748813 | 2.42097273 |
| FCF1P7 | -1.7865786 | -5.0057113 | -2.4697821 | 0.01553711 | 0.7084335 | -3.2069677 | -2.4196112 |
| NOP16 | 0.18548842 | 3.32324478 | 2.46924206 | 0.0155925 | 0.7084335 | -3.2134483 | 2.41831665 |
| IL1RAP | 0.53406702 | 3.96870777 | 2.47118558 | 0.01559623 | 0.7084335 | -3.2041488 | 2.41822975 |
| RNA5SP61 | -1.6139626 | -4.860363 | -2.4675145 | 0.01565706 | 0.7084335 | -3.2549294 | -2.416813 |
| HNRNPA1P22 | 1.47572734 | -6.2877399 | 2.46560659 | 0.01566331 | 0.7084335 | -3.1763433 | 2.41666775 |
| POU2AF1 | 1.36076509 | -6.0739381 | 2.46587964 | 0.0156677 | 0.7084335 | -3.157478 | 2.41656567 |
| LINC01133 | -1.7759117 | -5.2776961 | -2.4674575 | 0.01567393 | 0.7084335 | -3.1788999 | -2.416421 |
| AURKBP1 | 1.74969216 | -5.9471669 | 2.46504144 | 0.01567845 | 0.7084335 | -3.1943603 | 2.41631591 |
| CCNH | 0.2057069 | 5.00380586 | 2.46750676 | 0.01568616 | 0.7084335 | -3.2031327 | 2.41613697 |
| CEBPA-DT | -0.8081982 | 0.02117262 | -2.4675165 | 0.01570403 | 0.7084335 | -3.2051971 | -2.4157224 |
| SAXO3 | -1.1113805 | -2.6481537 | -2.4652031 | 0.01571658 | 0.7084335 | -3.2211043 | -2.4154314 |
| SEPTIN10P1 | -1.4591557 | -3.8945878 | -2.4641783 | 0.01574565 | 0.7084335 | -3.2134552 | -2.4147584 |
| RBM7 | 0.33888503 | 4.17025558 | 2.46655828 | 0.01577939 | 0.7084335 | -3.1776616 | 2.4139785 |
| SRGAP3-AS3 | -1.5560818 | -6.1481801 | -2.4629959 | 0.01583912 | 0.7084335 | -3.2115758 | -2.4126016 |
| HACL1 | 0.2136485 | 3.79419477 | 2.46372945 | 0.01588862 | 0.7084335 | -3.2099857 | 2.41146409 |
| PDGFB | 0.64383047 | 2.70838522 | 2.4631013 | 0.01589318 | 0.7084335 | -3.209705 | 2.41135938 |
| C18orf21 | 0.1799911 | 3.38739105 | 2.45854628 | 0.01602268 | 0.7084335 | -3.2151296 | 2.40839842 |
| DKK1 | 1.70487175 | 0.10667837 | 2.4580715 | 0.01604298 | 0.7084335 | -3.2107251 | 2.40793631 |
| AHCYP3 | -1.5920236 | -5.3863113 | -2.4568092 | 0.01604436 | 0.7084335 | -3.152261 | -2.4079049 |
| ATP6V1B2 | 0.28143947 | 7.61063908 | 2.45763313 | 0.01604917 | 0.7084335 | -3.2104037 | 2.40779541 |
| TRAC | 1.52581688 | 0.31971439 | 2.45720311 | 0.01605919 | 0.7084335 | -3.1942461 | 2.40756752 |
| OSMR | 1.18292707 | 4.60306126 | 2.45935533 | 0.01612099 | 0.7084335 | -3.1879233 | 2.4061649 |
| MYH2 | -2.041238 | -4.3512365 | -2.4553206 | 0.01612666 | 0.7084335 | -3.1870227 | -2.4060364 |
| TPM4 | 0.20686099 | 7.65048717 | 2.45711311 | 0.01612732 | 0.7084335 | -3.2169639 | 2.40602134 |
| FES | 0.70693516 | -0.4701754 | 2.45678876 | 0.01621682 | 0.7084335 | -3.2853225 | 2.403999 |
| MIR4527HG | -1.4715069 | -5.468121 | -2.4543563 | 0.0162263 | 0.7084335 | -3.2112299 | -2.4037854 |
| EDC3 | -0.1349127 | 5.57586153 | -2.4553678 | 0.01624073 | 0.7084335 | -3.2278779 | -2.4034602 |
| PTPRQ | 1.25380526 | -2.3656461 | 2.45315901 | 0.0162442 | 0.7084335 | -3.2365692 | 2.40338223 |
| SLC35D2 | 0.61863745 | 0.68119595 | 2.45432494 | 0.01625463 | 0.7084335 | -3.1859064 | 2.40314746 |
| DENND10 | 0.23663886 | 5.53500362 | 2.4517219 | 0.01631349 | 0.7084335 | -3.2212292 | 2.40182537 |
| THA1P | 1.09300104 | -0.7351414 | 2.45580505 | 0.01632986 | 0.7084335 | -3.2086756 | 2.40145858 |
| CAV1 | 1.09401184 | 3.04936222 | 2.4506092 | 0.01641624 | 0.7084335 | -3.1718897 | 2.39952767 |
| TFPI2 | 1.84793566 | 5.82896175 | 2.45103378 | 0.01643743 | 0.7084335 | -3.2113194 | 2.3990553 |
| BCL3 | 0.92783796 | 2.74620282 | 2.45014817 | 0.01645977 | 0.7084335 | -3.2131161 | 2.39855802 |
| CSF1R | 0.62758031 | -0.9461141 | 2.44780918 | 0.01646788 | 0.7084335 | -3.2422167 | 2.39837756 |
| ITPA | 0.24852729 | 5.1867142 | 2.44944123 | 0.01647078 | 0.7084335 | -3.2244135 | 2.39831304 |
| RHBDD3 | 0.16158078 | 5.66012816 | 2.44766429 | 0.01654828 | 0.7084335 | -3.2366788 | 2.39659322 |
| OPRL1 | -0.5012882 | 1.43074796 | -2.447542 | 0.01657593 | 0.7084335 | -3.2916935 | -2.3959813 |
| ADAM12 | 0.80333119 | 3.78369557 | 2.44706196 | 0.01657602 | 0.7084335 | -3.2143399 | 2.39597945 |
| PARP15 | 1.64338203 | -4.6361224 | 2.4454072 | 0.01660674 | 0.7084335 | -3.2646479 | 2.39530079 |
| DBTP1 | -1.9126159 | -4.947701 | -2.4432874 | 0.01664466 | 0.7084335 | -3.2536513 | -2.3944643 |
| LINC02605 | 1.61528657 | -3.9005104 | 2.44601007 | 0.01664776 | 0.7084335 | -3.1797281 | 2.39439611 |
| FGFBP2 | 1.4840253 | 2.226571 | 2.44337968 | 0.01671347 | 0.70966533 | -3.2461231 | 2.39295113 |
| PCSK5 | 0.6794962 | 4.72670666 | 2.44224713 | 0.01674024 | 0.70966533 | -3.2404479 | 2.39236387 |
| CYCSP45 | -1.0212562 | -2.3499386 | -2.4389217 | 0.01686476 | 0.71317683 | -3.2545825 | -2.3896429 |
| LINC01338 | -0.722635 | 0.25047502 | -2.4400864 | 0.01689428 | 0.71317683 | -3.2672404 | -2.3890004 |
| PZP | 1.1247989 | -1.5978276 | 2.43823237 | 0.01694596 | 0.71317683 | -3.2377915 | 2.38787803 |
| PTX3 | 0.94970308 | 4.18880989 | 2.43757262 | 0.01695076 | 0.71317683 | -3.2222796 | 2.38777407 |
| CFAP410 | 0.25009438 | 4.27921563 | 2.43251402 | 0.01701791 | 0.71465605 | -3.2476937 | 2.38632066 |
| SMIM7 | 0.18386483 | 5.19438944 | 2.43631389 | 0.01706208 | 0.7150535 | -3.2534062 | 2.38536724 |
| VAMP1-AS1 | 1.68520638 | -3.5705737 | 2.43009292 | 0.01709138 | 0.7150535 | -3.2245837 | 2.38473599 |
| RPL23AP95 | 1.59446409 | -4.8928073 | 2.4294574 | 0.01714002 | 0.71574818 | -3.2259972 | 2.38369021 |
| LAMB1 | 0.39080307 | 6.13691608 | 2.43377339 | 0.01717267 | 0.71577363 | -3.2507775 | 2.38298976 |
| KRT17P3 | -1.3915075 | -5.2663618 | -2.4310739 | 0.01720921 | 0.71596094 | -3.2509028 | -2.3822072 |
| LNMICC | 0.44432056 | 0.70593801 | 2.42891978 | 0.01730438 | 0.71844593 | -3.2302835 | 2.38017586 |
| TRADD | 0.47339332 | 2.03156801 | 2.42972044 | 0.01733326 | 0.71844593 | -3.2417846 | 2.3795613 |
| RNU6-583P | -1.3873486 | -5.0909391 | -2.425918 | 0.01740848 | 0.72022732 | -3.2255901 | -2.3779651 |
| GSTM4 | 0.3313859 | 4.38406189 | 2.42484796 | 0.01756666 | 0.7249483 | -3.2630845 | 2.37462766 |
| ELF3 | 1.52984081 | -0.5059265 | 2.43263566 | 0.01758748 | 0.7249483 | -3.3016308 | 2.37419029 |
| OLIG1 | -2.0635052 | 1.50896912 | -2.4230204 | 0.0176591 | 0.72655966 | -3.3020485 | -2.3726896 |
| C1R | 1.31477433 | 5.42224774 | 2.42084705 | 0.01777536 | 0.72922819 | -3.2619858 | 2.37026448 |
| TMEM212 | -0.9225728 | -6.3773476 | -2.4198873 | 0.01778924 | 0.72922819 | -3.2600446 | -2.369976 |
| MAN1C1 | 0.70372101 | 5.16254874 | 2.41763215 | 0.01790365 | 0.73000972 | -3.2605102 | 2.36760473 |
| NPY5R | 0.79814608 | 2.04289433 | 2.41533539 | 0.01795923 | 0.73000972 | -3.3125411 | 2.36645772 |
| ENTR1P2 | 1.67904188 | -5.2410095 | 2.41217711 | 0.01797956 | 0.73000972 | -3.2838336 | 2.36603876 |
| RIN3 | 0.44012396 | 2.67031738 | 2.41455652 | 0.01802669 | 0.73000972 | -3.264007 | 2.36506954 |
| LINC01219 | -1.4490546 | -5.9006883 | -2.4118806 | 0.01807287 | 0.73000972 | -3.2898543 | -2.3641219 |
| PMP22 | 0.85493064 | 5.51348698 | 2.41345636 | 0.01808064 | 0.73000972 | -3.2627293 | 2.36396253 |
| MINPP1 | 0.20978835 | 4.80961899 | 2.41185815 | 0.01809342 | 0.73000972 | -3.2788521 | 2.36370082 |
| ALDH1L1-AS1 | 1.48672213 | -4.0638405 | 2.4107679 | 0.01809873 | 0.73000972 | -3.3205072 | 2.36359215 |
| LINC02857 | -1.6994791 | -5.4756601 | -2.4116907 | 0.01810238 | 0.73000972 | -3.2574686 | -2.3635172 |
| TMEM150A | 0.37005912 | 4.34482457 | 2.41165968 | 0.01816701 | 0.73079176 | -3.2861087 | 2.36219643 |
| LINC00954 | 1.01023768 | -2.6329975 | 2.40991737 | 0.0181872 | 0.73079176 | -3.3207191 | 2.36178474 |
| TBX2-AS1 | -1.7574997 | -3.4124249 | -2.406785 | 0.01830469 | 0.73125472 | -3.3454752 | -2.3593965 |
| TPH2 | -1.7456998 | -2.9217319 | -2.4057948 | 0.01832502 | 0.73125472 | -3.2877453 | -2.3589846 |
| DPP4 | 1.10764765 | 0.95596004 | 2.40752738 | 0.01832863 | 0.73125472 | -3.2745241 | 2.35891135 |
| HYCC1 | 0.44500997 | 6.33334743 | 2.40695561 | 0.01834182 | 0.73125472 | -3.2840617 | 2.3586444 |
| LINC01970 | 0.88169254 | -1.6921242 | 2.40627858 | 0.01839652 | 0.73125472 | -3.3199231 | 2.35753905 |
| CLCA2 | 1.41232383 | -2.8286589 | 2.40224048 | 0.01849577 | 0.73125472 | -3.2992188 | 2.35554081 |
| VDAC2P5 | -0.4909559 | -0.982039 | -2.4011251 | 0.01850325 | 0.73125472 | -3.2775212 | -2.3553904 |
| CLDN19 | 1.77353337 | -3.4801625 | 2.40166932 | 0.01857461 | 0.73125472 | -3.3354155 | 2.35396003 |
| H2BC18 | -0.6687176 | -0.9322487 | -2.3992705 | 0.01865643 | 0.73125472 | -3.2982196 | -2.3523258 |
| GATA2 | -1.5736593 | -1.9496327 | -2.3996979 | 0.0186724 | 0.73125472 | -3.3788147 | -2.3520074 |
| ZNF844 | -0.2583516 | 3.01093783 | -2.3966433 | 0.01876688 | 0.73125472 | -3.2876878 | -2.3501294 |
| OR2A7 | 1.20681272 | -5.9066169 | 2.39643929 | 0.01878972 | 0.73125472 | -3.3028903 | 2.34967683 |
| SLC35A2 | 0.25838578 | 4.88811215 | 2.39814471 | 0.01879293 | 0.73125472 | -3.3086751 | 2.34961324 |
| FAM53A | 0.56738796 | 0.08052666 | 2.39502595 | 0.01880597 | 0.73125472 | -3.3109372 | 2.34935488 |
| EMILIN2 | 0.53199525 | 0.46024688 | 2.39771025 | 0.01881997 | 0.73125472 | -3.308326 | 2.3490779 |
| MGAT1 | 0.20347447 | 6.43882121 | 2.39755025 | 0.01884147 | 0.73125472 | -3.3033426 | 2.34865267 |
| RNF19A | 0.40909087 | 6.77380448 | 2.39725447 | 0.01885602 | 0.73125472 | -3.3060925 | 2.34836517 |
| MIR649 | 1.52784931 | -5.7890432 | 2.39344304 | 0.01887303 | 0.73125472 | -3.3163555 | 2.34802945 |
| MFSD12 | 0.18612891 | 5.97990941 | 2.39459549 | 0.01890205 | 0.73125472 | -3.3108857 | 2.34745702 |
| EPN2 | -0.2637473 | 5.55802264 | -2.3958313 | 0.01893175 | 0.73125472 | -3.3100189 | -2.3468721 |
| BUD23 | 0.22521511 | 5.79921113 | 2.39582127 | 0.01893702 | 0.73125472 | -3.3068327 | 2.34676834 |
| MKRN5P | 1.53798911 | -4.65236 | 2.39264106 | 0.0189459 | 0.73125472 | -3.289544 | 2.34659382 |
| RNF14P4 | 1.6711862 | -4.1700562 | 2.39213195 | 0.01897393 | 0.73125472 | -3.312303 | 2.34604273 |
| GALNT16 | -0.2742705 | 5.76626749 | -2.3913086 | 0.01905366 | 0.73125472 | -3.311214 | -2.3444794 |
| CAHM | -0.3235826 | 1.98224223 | -2.3915506 | 0.01906629 | 0.73125472 | -3.3007213 | -2.3442323 |
| GRHL1 | -0.577019 | 2.4569912 | -2.3924842 | 0.0190845 | 0.73125472 | -3.3194989 | -2.3438764 |
| FNIP1 | 0.20936756 | 5.59004672 | 2.39661727 | 0.01909625 | 0.73125472 | -3.2887794 | 2.34364676 |
| LINC01979 | 0.6523825 | -0.659015 | 2.3916108 | 0.01915888 | 0.73125472 | -3.3697326 | 2.34242501 |
| ZFAND2A-DT | 0.24424404 | 3.31198916 | 2.39051162 | 0.01919259 | 0.73125472 | -3.3116212 | 2.34176895 |
| RINL | 0.46928118 | 0.58609183 | 2.38764488 | 0.01928132 | 0.73125472 | -3.3360773 | 2.34004681 |
| RN7SL68P | -1.4910504 | -5.1034376 | -2.3845609 | 0.01928598 | 0.73125472 | -3.3114977 | -2.3399567 |
| ADCYAP1R1 | -0.5888515 | 7.51680523 | -2.3876686 | 0.01929712 | 0.73125472 | -3.3222466 | -2.339741 |
| NDRG2 | -0.4287089 | 7.34293317 | -2.3870308 | 0.01930659 | 0.73125472 | -3.3174481 | -2.3395577 |
| GJA3 | 2.05449513 | -3.880047 | 2.38499368 | 0.01933673 | 0.73125472 | -3.3185423 | 2.33897494 |
| PEDS1 | 0.3136388 | 5.50524358 | 2.38481446 | 0.01934432 | 0.73125472 | -3.3190965 | 2.33882825 |
| UBE2D3P3 | 1.55111545 | -5.9532668 | 2.38134807 | 0.01946208 | 0.73251959 | -3.3272559 | 2.33655995 |
| MTHFD2P1 | 1.74650663 | -4.9060193 | 2.38283381 | 0.0194722 | 0.73251959 | -3.3294824 | 2.3363655 |
| RPS20P10 | -1.6568685 | -5.5166817 | -2.381462 | 0.01949149 | 0.73251959 | -3.2919387 | -2.3359952 |
| TNPO1P2 | -1.7007258 | -5.5197618 | -2.3785081 | 0.01952035 | 0.73251959 | -3.3163313 | -2.3354418 |
| PLA2R1 | 0.84104066 | 1.88803496 | 2.38231622 | 0.01954172 | 0.73251959 | -3.3292811 | 2.33503246 |
| SNORA66 | 0.81132248 | -1.8920879 | 2.3791561 | 0.01965153 | 0.73369275 | -3.3371082 | 2.33293554 |
| ELK3 | 0.78176124 | 4.26017716 | 2.37794021 | 0.01979573 | 0.73369275 | -3.3172065 | 2.33019728 |
| H2AZP1 | -1.2270187 | -6.3243489 | -2.3760459 | 0.01979879 | 0.73369275 | -3.315695 | -2.3301393 |
| DHH | -1.0655256 | -1.0131736 | -2.3763044 | 0.01980555 | 0.73369275 | -3.3828347 | -2.3300114 |
| UBE2CP2 | 1.50412874 | -3.9211892 | 2.37547421 | 0.01980619 | 0.73369275 | -3.360486 | 2.32999925 |
| NRG4 | -0.6391747 | 0.77722136 | -2.3744409 | 0.01981697 | 0.73369275 | -3.3508945 | -2.3297954 |
| TACC2 | -0.2872946 | 6.55632249 | -2.3772907 | 0.01982451 | 0.73369275 | -3.340653 | -2.3296527 |
| RPL36AP26 | -1.4952808 | -3.2429442 | -2.3739744 | 0.01988756 | 0.73369275 | -3.326283 | -2.3284624 |
| PARP11 | 0.17447528 | 4.69546102 | 2.37499152 | 0.01991935 | 0.73369275 | -3.3430079 | 2.32786354 |
| BNAT1 | 0.95747338 | -0.370286 | 2.37510301 | 0.01994397 | 0.73369275 | -3.3139238 | 2.32740029 |
| TNFAIP3 | 0.88966123 | 3.1652291 | 2.37473591 | 0.01995654 | 0.73369275 | -3.314199 | 2.32716394 |
| SNORD115-4 | -1.2011424 | -4.3771416 | -2.3729167 | 0.01996711 | 0.73369275 | -3.3604531 | -2.3269654 |
| MATN1-AS1 | 0.9126508 | -1.0112392 | 2.36667117 | 0.02020672 | 0.73879047 | -3.3580234 | 2.32248708 |
| ITGA6-AS1 | -1.5698346 | -5.1207439 | -2.3656968 | 0.02025318 | 0.73879047 | -3.3369107 | -2.3216242 |
| HAS2 | 0.87816009 | 2.55528971 | 2.36581633 | 0.02038356 | 0.73879047 | -3.3093047 | 2.31921165 |
| SH2D3A | -0.7737684 | 0.45404485 | -2.3659102 | 0.02039901 | 0.73879047 | -3.4339456 | -2.3189268 |
| GPR39 | 0.57254279 | 0.15071375 | 2.36496156 | 0.02040594 | 0.73879047 | -3.3539475 | 2.31879895 |
| PSMD7 | 0.1936784 | 5.23102661 | 2.36280955 | 0.0204243 | 0.73879047 | -3.344876 | 2.31846064 |
| CCDC25 | 0.21544278 | 5.64176962 | 2.36534138 | 0.02043846 | 0.73879047 | -3.3505223 | 2.31819985 |
| BAALC | 0.40438767 | 7.00272628 | 2.36406772 | 0.02044113 | 0.73879047 | -3.3493992 | 2.31815069 |
| MTDH | 0.24472571 | 7.4433491 | 2.36408051 | 0.02046614 | 0.73879047 | -3.3501031 | 2.31769069 |
| GPX1P1 | -0.9693507 | -0.2265614 | -2.3643298 | 0.02046917 | 0.73879047 | -3.3631923 | -2.317635 |
| R3HCC1L | 0.1795865 | 3.90185591 | 2.36293266 | 0.02047572 | 0.73879047 | -3.3631914 | 2.3175145 |
| NUSAP1 | -1.0391868 | -3.1182915 | -2.3589577 | 0.02050267 | 0.73879047 | -3.3307727 | -2.3170195 |
| AMDHD2 | 0.28513914 | 4.33088661 | 2.36141221 | 0.02055065 | 0.73903507 | -3.3626093 | 2.31613964 |
| ELMOD1 | 0.51031531 | 6.36447992 | 2.35820662 | 0.02075737 | 0.73903507 | -3.3661889 | 2.31236872 |
| TMEM267 | 0.29814834 | 3.20479666 | 2.35749196 | 0.02079563 | 0.73903507 | -3.3579166 | 2.31167442 |
| MIR181A1HG | -0.7918968 | -1.3981021 | -2.3575331 | 0.02080443 | 0.73903507 | -3.3917534 | -2.311515 |
| NKX3-2 | -1.7345147 | -3.4187326 | -2.3562738 | 0.02081605 | 0.73903507 | -3.3549777 | -2.3113044 |
| KDM4D | 0.26108697 | 2.40084932 | 2.35407574 | 0.02089394 | 0.73903507 | -3.3512185 | 2.30989558 |
| LIF | 1.04069955 | 1.07420664 | 2.35570975 | 0.02093978 | 0.73903507 | -3.354968 | 2.30906845 |
| CNN3-DT | 0.4727462 | 4.70121826 | 2.35376345 | 0.0209736 | 0.73903507 | -3.3558462 | 2.30845941 |
| PTPRM | -0.2064749 | 7.34154086 | -2.3535017 | 0.02100262 | 0.73903507 | -3.3690453 | -2.3079373 |
| GOLGA5 | 0.18707171 | 5.56451728 | 2.353618 | 0.02103509 | 0.73903507 | -3.3644029 | 2.307354 |
| IP6K3 | 1.93311569 | -4.1632958 | 2.35292216 | 0.0210426 | 0.73903507 | -3.3576174 | 2.30721922 |
| DNAJC10 | 0.21055532 | 7.51115089 | 2.3528635 | 0.02107092 | 0.73903507 | -3.3704928 | 2.30671132 |
| FKBP14 | 0.26101307 | 4.66988342 | 2.35220115 | 0.02111166 | 0.73903507 | -3.3693601 | 2.30598163 |
| PEA15 | -0.3715446 | 9.81085964 | -2.3517776 | 0.02111218 | 0.73903507 | -3.3705332 | -2.3059724 |
| MAP3K6 | 0.36212295 | 4.36134848 | 2.35154969 | 0.02112428 | 0.73903507 | -3.3611828 | 2.30575575 |
| EXOSC10-AS1 | -0.9476124 | -2.5398645 | -2.349237 | 0.02113103 | 0.73903507 | -3.3669357 | -2.3056351 |
| BMP4 | 1.01500906 | 1.41524718 | 2.3515103 | 0.02113687 | 0.73903507 | -3.35526 | 2.3055307 |
| LINC02256 | 0.49196304 | -1.1419117 | 2.34938133 | 0.02116145 | 0.73903507 | -3.3954973 | 2.30509145 |
| LINC01213 | 1.31451616 | -3.0892449 | 2.3502373 | 0.02116886 | 0.73903507 | -3.3642813 | 2.30495913 |
| LINC02773 | 1.3886261 | -3.9160984 | 2.35010774 | 0.02117105 | 0.73903507 | -3.3322486 | 2.30492006 |
| CUTC | 0.2817397 | 3.37118863 | 2.34944088 | 0.02127156 | 0.73972372 | -3.3682575 | 2.30312948 |
| PLIN2 | 0.61642637 | 3.71140022 | 2.34917659 | 0.02128325 | 0.73972372 | -3.3866254 | 2.30292169 |
| STK10 | 0.34887503 | 4.01457638 | 2.34735659 | 0.02133605 | 0.73972372 | -3.3649931 | 2.3019845 |
| LINC01063 | -1.1890356 | -3.0755688 | -2.3450247 | 0.02141252 | 0.73972372 | -3.3936977 | -2.3006307 |
| RPH3A | -0.8115329 | 3.79293877 | -2.344544 | 0.02150191 | 0.73972372 | -3.4147278 | -2.2990534 |
| TSPO | 0.47957804 | 4.53609261 | 2.34376134 | 0.02154897 | 0.73972372 | -3.3727242 | 2.29822521 |
| ZNF221 | -0.3198771 | 3.14488085 | -2.343068 | 0.02157718 | 0.73972372 | -3.3727747 | -2.2977296 |
| PABPC1P4 | -1.5026177 | -3.6512965 | -2.3405728 | 0.02160238 | 0.73972372 | -3.3572849 | -2.2972874 |
| KIF25-AS1 | 0.77560419 | -1.0720604 | 2.34254239 | 0.02160436 | 0.73972372 | -3.4278586 | 2.29725259 |
| ADAM32 | 0.77613153 | 1.51973656 | 2.34126258 | 0.0216283 | 0.73972372 | -3.3927678 | 2.29683298 |
| PNRC2P1 | -1.3967548 | -3.367511 | -2.3376964 | 0.02163331 | 0.73972372 | -3.3808403 | -2.2967452 |
| FSCN2 | 0.44686916 | 1.95684021 | 2.34463893 | 0.02166749 | 0.73972372 | -3.40888 | 2.29614685 |
| SP100 | 1.12862073 | 0.83323936 | 2.34166815 | 0.02168771 | 0.73972372 | -3.3479825 | 2.29579317 |
| EPHB1 | -0.4293785 | 6.80970481 | -2.3415846 | 0.02169276 | 0.73972372 | -3.3885453 | -2.2957049 |
| UROS | 0.17160098 | 6.46025191 | 2.3393159 | 0.02170734 | 0.73972372 | -3.3858807 | 2.29545012 |
| CRLS1 | 0.42387542 | 3.36850301 | 2.33891399 | 0.02172055 | 0.73972372 | -3.3749939 | 2.29521951 |
| NRBP1 | 0.16971983 | 7.3528744 | 2.33853972 | 0.02180248 | 0.74138372 | -3.3885387 | 2.29379154 |
| MEI1 | -1.3695917 | -2.2979227 | -2.3326976 | 0.02207014 | 0.7490981 | -3.4402181 | -2.2891585 |
| SMIM2-AS1 | -1.0248598 | -2.5265461 | -2.3324411 | 0.0220964 | 0.7490981 | -3.3807558 | -2.2887067 |
| ZNF563 | 0.36889134 | 0.98543029 | 2.33164135 | 0.02220689 | 0.75125643 | -3.3914056 | 2.28681047 |
| KCNJ16 | -0.9712846 | 0.87575226 | -2.3309777 | 0.0222477 | 0.75125643 | -3.3631827 | -2.2861122 |
| RNA5SP290 | -1.4176052 | -5.0053673 | -2.3294067 | 0.02226094 | 0.75125643 | -3.4313515 | -2.2858857 |
| LINC01589 | -1.2382261 | -3.7290524 | -2.3285296 | 0.02232098 | 0.7517679 | -3.3978237 | -2.284861 |
| ZNF404 | -0.4546499 | 2.25409984 | -2.3271668 | 0.02242037 | 0.7517679 | -3.4070139 | -2.2831698 |
| RNA5SP260 | -1.4094103 | -5.8751819 | -2.326035 | 0.0224243 | 0.7517679 | -3.4138801 | -2.283103 |
| RHBG | 1.47913881 | -2.3766356 | 2.32686911 | 0.02243905 | 0.7517679 | -3.4285408 | 2.28285261 |
| PSMB8 | 0.85809967 | 2.56137841 | 2.32751376 | 0.02253593 | 0.7517679 | -3.3778731 | 2.28121153 |
| SGMS1-AS1 | 0.34340623 | 3.06686694 | 2.32580234 | 0.02256543 | 0.7517679 | -3.4162738 | 2.28071301 |
| LINC02936 | 1.0568056 | -1.9912974 | 2.32625112 | 0.02256868 | 0.7517679 | -3.433389 | 2.28065828 |
| ARHGAP6 | 0.57010167 | 2.1302842 | 2.32243821 | 0.02266311 | 0.7517679 | -3.4282616 | 2.27906658 |
| ZNF518B | -0.2572344 | 5.53985284 | -2.3231367 | 0.02268185 | 0.7517679 | -3.4124434 | -2.2787513 |
| DERL3 | -0.6173793 | -0.4540826 | -2.3224981 | 0.02268634 | 0.7517679 | -3.4200887 | -2.2786758 |
| ETNPPL | -0.6900806 | 0.24281092 | -2.3229947 | 0.02271007 | 0.7517679 | -3.4543116 | -2.2782772 |
| CHI3L2 | 1.74660282 | -0.2792952 | 2.32422144 | 0.02272344 | 0.7517679 | -3.3571402 | 2.27805264 |
| AP1AR-DT | 0.37952919 | 1.52509534 | 2.32348301 | 0.0227282 | 0.7517679 | -3.4179239 | 2.27797271 |
| POLRMT | 0.16001853 | 6.16613207 | 2.3204545 | 0.02280979 | 0.7517679 | -3.4175038 | 2.27660552 |
| JAGN1 | 0.23873598 | 4.68331739 | 2.3208181 | 0.02281452 | 0.7517679 | -3.4118608 | 2.27652638 |
| LIPH | 0.96453802 | -0.0815339 | 2.31992463 | 0.02283225 | 0.7517679 | -3.4407316 | 2.27622999 |
| ENPP7P9 | -1.5804535 | -4.8222316 | -2.3193573 | 0.02286865 | 0.7517679 | -3.47303 | -2.2756218 |
| TATDN1P1 | 1.63081393 | -4.9527811 | 2.31533279 | 0.02288179 | 0.7517679 | -3.4091397 | 2.27540252 |
| C14orf119 | 0.22947424 | 4.41924322 | 2.31754319 | 0.02293424 | 0.75238442 | -3.4057701 | 2.27452837 |
| PYGL | 0.39980352 | 4.13687576 | 2.31740347 | 0.02299715 | 0.75316675 | -3.4148653 | 2.27348194 |
| TBX19 | 0.4156272 | 0.75125991 | 2.31696847 | 0.02303033 | 0.75316675 | -3.4162195 | 2.27293107 |
| KRT7 | 1.58814187 | -4.2474737 | 2.31652973 | 0.02307537 | 0.75316675 | -3.3838597 | 2.27218443 |
| RUNX2 | 0.68128017 | 2.30362931 | 2.3157598 | 0.02312111 | 0.75316675 | -3.3955193 | 2.27142748 |
| CLEC4D | -1.4769268 | -5.6653273 | -2.3131692 | 0.02314739 | 0.75316675 | -3.4267112 | -2.2709931 |
| NUP153-AS1 | 1.33434703 | -3.0135157 | 2.31539072 | 0.02319884 | 0.75316675 | -3.4194324 | 2.27014411 |
| LY6H | 0.62069282 | 8.32153822 | 2.31260939 | 0.02326875 | 0.75316675 | -3.4249487 | 2.26899294 |
| RNU4-50P | -1.4141665 | -5.5725513 | -2.3074293 | 0.02336219 | 0.75316675 | -3.4219792 | -2.2674591 |
| NCAN | -0.6756304 | 8.69138897 | -2.3116141 | 0.02336859 | 0.75316675 | -3.4290331 | -2.2673544 |
| KRT18P31 | -1.6298529 | -5.6688339 | -2.3090353 | 0.02338032 | 0.75316675 | -3.4429082 | -2.2671622 |
| EIF5 | 0.17470611 | 8.01570348 | 2.30972611 | 0.02341065 | 0.75316675 | -3.4246044 | 2.2666658 |
| ANGPT1 | 0.78738251 | 3.30035444 | 2.31002042 | 0.02341821 | 0.75316675 | -3.4146136 | 2.26654213 |
| OR52E2 | 1.24667312 | -6.6666234 | 2.30816598 | 0.02343419 | 0.75316675 | -3.421698 | 2.26628096 |
| ISM2 | -0.7199479 | 1.16657812 | -2.3081781 | 0.02349214 | 0.75316675 | -3.4488059 | -2.2653348 |
| CDH8-AS1 | 1.73119051 | -5.5718814 | 2.30569629 | 0.02353428 | 0.75316675 | -3.4184063 | 2.26464815 |
| MTND1P9 | -1.5714715 | -5.9919335 | -2.3057855 | 0.02359807 | 0.75316675 | -3.4130678 | -2.2636107 |
| PCDHA8 | 0.51841897 | 0.41428469 | 2.30537475 | 0.02361594 | 0.75316675 | -3.4521327 | 2.26332057 |
| KRCC1 | 0.23230827 | 4.12238073 | 2.3074341 | 0.02361619 | 0.75316675 | -3.4202104 | 2.26331651 |
| RPL9P21 | -1.6691795 | -5.6731593 | -2.3043127 | 0.02362857 | 0.75316675 | -3.4055477 | -2.2631156 |
| OSBPL5 | 0.23007935 | 6.40624128 | 2.30552779 | 0.02363233 | 0.75316675 | -3.4352978 | 2.26305449 |
| NKX2-8 | -1.6603082 | -4.9568609 | -2.3039139 | 0.02370288 | 0.75391925 | -3.4713337 | -2.2619113 |
| CLCF1 | 0.78566911 | 1.01236298 | 2.30498286 | 0.02373256 | 0.75391925 | -3.4419978 | 2.26143132 |
| TNFRSF11B | 1.45794025 | 3.88933441 | 2.30468595 | 0.02375718 | 0.75391925 | -3.4106195 | 2.26103355 |
| MKRN4P | 1.18195453 | -1.153011 | 2.30193101 | 0.02385001 | 0.75522969 | -3.44887 | 2.25953679 |
| TCEAL9 | 0.36984756 | 5.79325382 | 2.30311902 | 0.023888 | 0.75522969 | -3.4351086 | 2.25892581 |
| SMIM12 | 0.17322513 | 4.77707361 | 2.30182319 | 0.02396456 | 0.75522969 | -3.4408224 | 2.25769688 |
| DSG2 | 0.78498223 | 3.73456631 | 2.30057845 | 0.02397601 | 0.75522969 | -3.4301362 | 2.25751348 |
| MYRF-AS1 | 1.44872897 | -5.7957955 | 2.29818578 | 0.02399123 | 0.75522969 | -3.4383907 | 2.25726966 |
| HSP90AA3P | -1.0902648 | -2.0104856 | -2.2977888 | 0.0240013 | 0.75522969 | -3.4329353 | -2.2571084 |
| TRIM43B | 1.60021636 | -5.9306283 | 2.29615024 | 0.02412249 | 0.75797542 | -3.4318724 | 2.25517273 |
| C2CD2 | 0.42932811 | 4.46948428 | 2.29716787 | 0.02419143 | 0.75801033 | -3.4493362 | 2.25407534 |
| DUSP22 | 0.18709104 | 5.36218235 | 2.29686553 | 0.02419146 | 0.75801033 | -3.4507637 | 2.25407487 |
| MIR548XHG | -1.373013 | -6.3587153 | -2.2939543 | 0.02422788 | 0.75808835 | -3.4272752 | -2.2534962 |
| LCN2 | 2.21648144 | -4.2713043 | 2.29636944 | 0.02428399 | 0.75878135 | -3.4204029 | 2.25260616 |
| ACSS3 | 0.73457252 | 3.96063324 | 2.29543982 | 0.02432125 | 0.75888417 | -3.4383062 | 2.25201616 |
| CD109 | 0.83308086 | 3.5557066 | 2.29198656 | 0.02453455 | 0.76031302 | -3.4354585 | 2.24865357 |
| LINC03072 | 0.20939513 | 5.48817133 | 2.29098954 | 0.02458085 | 0.76031302 | -3.4578089 | 2.2479269 |
| FOXI1 | -1.27746 | -6.7162182 | -2.2899398 | 0.02459705 | 0.76031302 | -3.4430098 | -2.2476731 |
| GCLM | 0.34887697 | 5.03963546 | 2.29038815 | 0.02461946 | 0.76031302 | -3.4635376 | 2.24732192 |
| TMEM89 | 1.02148846 | -2.3179683 | 2.29414663 | 0.0246478 | 0.76031302 | -3.4864068 | 2.24687836 |
| CRYBA2 | -1.6870336 | -5.7297581 | -2.2871573 | 0.02465391 | 0.76031302 | -3.4420947 | -2.2467828 |
| IRF7 | 0.42135594 | 3.59633664 | 2.29001826 | 0.024658 | 0.76031302 | -3.4442471 | 2.24671882 |
| C7 | 1.5580533 | -2.7017477 | 2.28916746 | 0.02466814 | 0.76031302 | -3.4325243 | 2.24656034 |
| HSPA2 | 0.67234306 | 3.34726981 | 2.28988881 | 0.02467333 | 0.76031302 | -3.4737086 | 2.24647916 |
| POGLUT3 | 0.36580224 | 4.80074945 | 2.28814499 | 0.02477504 | 0.76171503 | -3.4576464 | 2.24489248 |
| APOL6 | 1.20575439 | 2.79548519 | 2.28887913 | 0.02478702 | 0.76171503 | -3.4412922 | 2.24470587 |
| PPP1R13L | 0.67650618 | 1.68739382 | 2.28587047 | 0.02484394 | 0.76197924 | -3.4506382 | 2.24382072 |
| SLC10A3 | 0.29544851 | 3.49571734 | 2.28358246 | 0.02503463 | 0.76197924 | -3.467351 | 2.24086793 |
| MIR3125 | 1.09715778 | -2.8709254 | 2.28138236 | 0.02504408 | 0.76197924 | -3.469478 | 2.24072206 |
| IL16 | -0.563725 | 0.67673964 | -2.2829538 | 0.02505768 | 0.76197924 | -3.4656169 | -2.2405123 |
| PEG3 | -0.5692065 | 2.29264667 | -2.2834833 | 0.02507294 | 0.76197924 | -3.4581286 | -2.2402771 |
| IL31RA | 0.97289953 | -2.0013909 | 2.28056729 | 0.02514245 | 0.76197924 | -3.4580194 | 2.23920706 |
| DPAGT1 | 0.25552446 | 4.40482348 | 2.28027979 | 0.02522304 | 0.76197924 | -3.4708221 | 2.2379696 |
| FAM120B | -0.1667237 | 5.94848806 | -2.2793904 | 0.02525464 | 0.76197924 | -3.47372 | -2.2374853 |
| ATL3 | 0.38528457 | 5.31547605 | 2.28018541 | 0.025256 | 0.76197924 | -3.4658608 | 2.2374644 |
| RPS27AP6 | -0.9700946 | -2.8144322 | -2.2804784 | 0.0252755 | 0.76197924 | -3.4686431 | -2.2371659 |
| DDX6P2 | -1.5896379 | -5.878426 | -2.2772048 | 0.02528949 | 0.76197924 | -3.4716663 | -2.2369519 |
| RNU6-1091P | 1.31818445 | -4.4199877 | 2.27491874 | 0.02543138 | 0.76197924 | -3.4928703 | 2.23478627 |
| NRCAM | 0.44668286 | 8.90832513 | 2.27485026 | 0.02552379 | 0.76197924 | -3.4781461 | 2.23338157 |
| RPL12P37 | -1.5322587 | -5.1819142 | -2.2740465 | 0.02553944 | 0.76197924 | -3.4824847 | -2.2331442 |
| LINC02027 | 1.82390731 | -3.3865606 | 2.2752491 | 0.02554681 | 0.76197924 | -3.4574093 | 2.23303231 |
| ZNG1A | 0.19720335 | 4.9358621 | 2.27536628 | 0.02558214 | 0.76197924 | -3.4844355 | 2.23249684 |
| IFTAP | -0.1791846 | 3.99816343 | -2.272828 | 0.02561047 | 0.76197924 | -3.4768566 | -2.232068 |
| IFITM3P2 | 1.72304291 | -4.9946417 | 2.27366099 | 0.02561859 | 0.76197924 | -3.4598626 | 2.23194508 |
| DUS4L-BCAP29 | 0.26872248 | 2.97260572 | 2.27434557 | 0.02563673 | 0.76197924 | -3.4808323 | 2.23167085 |
| LINC01505 | -0.7982332 | -1.3504205 | -2.2737214 | 0.02566554 | 0.76197924 | -3.4986042 | -2.2312354 |
| RIPOR3 | 0.76581996 | 3.50322648 | 2.27331951 | 0.02568393 | 0.76197924 | -3.4855642 | 2.23095768 |
| LINC01278 | -0.3231243 | 4.82920556 | -2.2723953 | 0.0257009 | 0.76197924 | -3.47924 | -2.2307016 |
| LTV1 | 0.22013936 | 3.33686825 | 2.27187929 | 0.02571765 | 0.76197924 | -3.4864792 | 2.23044896 |
| PLD3 | 0.3485329 | 9.17978665 | 2.27169464 | 0.02572291 | 0.76197924 | -3.4820287 | 2.23036964 |
| TRAF7 | 0.21524464 | 6.90197515 | 2.27005857 | 0.02573882 | 0.76197924 | -3.4788915 | 2.2301299 |
| H4C8 | -0.4319528 | -0.3294703 | -2.2717249 | 0.02576269 | 0.76197924 | -3.4746652 | -2.2297705 |
| CAMKK1 | -0.2666732 | 4.27080095 | -2.2717056 | 0.02578407 | 0.76197924 | -3.5027348 | -2.2294486 |
| SEPTIN14P12 | -1.2874756 | -5.132077 | -2.2696681 | 0.02580072 | 0.76197924 | -3.4802384 | -2.2291981 |
| SV2C-AS1 | 1.08253473 | -0.8901317 | 2.26976908 | 0.02582929 | 0.76197924 | -3.4660921 | 2.22876887 |
| POTEE | -0.536753 | 0.80105602 | -2.2712503 | 0.02582992 | 0.76197924 | -3.4834903 | -2.2287594 |
| OSTM1 | 0.30785026 | 5.94086175 | 2.26775294 | 0.02590354 | 0.76197924 | -3.4817025 | 2.2276549 |
| TLR2 | 1.20625655 | -2.1392197 | 2.26898456 | 0.02591359 | 0.76197924 | -3.4806719 | 2.2275043 |
| FUOM | 0.57190072 | 1.75124498 | 2.26967602 | 0.02592114 | 0.76197924 | -3.4876853 | 2.22739118 |
| ALOX12 | 0.33250568 | 2.06420543 | 2.26624298 | 0.0261332 | 0.76312492 | -3.5096816 | 2.22422661 |
| IAH1 | 0.29021958 | 3.99440041 | 2.26600597 | 0.02618198 | 0.76312492 | -3.4923526 | 2.22350187 |
| SLC9A9 | -0.5750132 | 1.4791441 | -2.263868 | 0.02625228 | 0.76312492 | -3.5000034 | -2.2224593 |
| DLL3 | -0.6584419 | 4.92483463 | -2.2626939 | 0.02633216 | 0.76312492 | -3.4909693 | -2.2212777 |
| FABP5 | 0.28638205 | 3.79011616 | 2.2623518 | 0.02637847 | 0.76312492 | -3.4777631 | 2.22059397 |
| NTMT2 | -1.0120942 | -1.6356996 | -2.260906 | 0.02640901 | 0.76312492 | -3.4719428 | -2.2201437 |
| CD80 | 1.48961558 | -5.7666788 | 2.25828237 | 0.02645387 | 0.76312492 | -3.4966816 | 2.21948316 |
| OLIG2 | -1.7750928 | 0.38822887 | -2.2601761 | 0.02646221 | 0.76312492 | -3.5035941 | -2.2193605 |
| MGAT4B | 0.25778091 | 7.6320646 | 2.25968134 | 0.02646508 | 0.76312492 | -3.4980126 | 2.21931825 |
| LINC03041 | 1.412216 | -6.1350665 | 2.25943394 | 0.0264718 | 0.76312492 | -3.4823582 | 2.21921946 |
| H3C2 | -1.6333328 | -5.7328649 | -2.2590396 | 0.02647374 | 0.76312492 | -3.482523 | -2.2191909 |
| ATAD3A | 0.15998982 | 5.59048454 | 2.26024553 | 0.02648127 | 0.76312492 | -3.5012955 | 2.21908011 |
| UQCC1 | 0.12485277 | 5.81355923 | 2.25903984 | 0.02653712 | 0.76312492 | -3.5028889 | 2.2182599 |
| UBE2V1P2 | -1.1890588 | -3.7806171 | -2.256965 | 0.02657332 | 0.76312492 | -3.4933457 | -2.2177289 |
| LRRC14B | 1.28642595 | -0.6598491 | 2.25764761 | 0.02660333 | 0.76312492 | -3.4954786 | 2.21728934 |
| IQCG | 0.47499693 | 5.66735931 | 2.25855055 | 0.02663494 | 0.76312492 | -3.4952854 | 2.21682658 |
| TNIP2 | 0.26737545 | 4.51839411 | 2.25791862 | 0.0266454 | 0.76312492 | -3.4967566 | 2.21667361 |
| CCDC160 | 0.53982333 | 1.71131601 | 2.25711079 | 0.0266614 | 0.76312492 | -3.4855238 | 2.21643978 |
| HIBADH | 0.26748118 | 4.01675077 | 2.25771568 | 0.02667785 | 0.76312492 | -3.4960146 | 2.21619936 |
| ATP5MFP4 | -1.6031499 | -5.743633 | -2.2528921 | 0.02671063 | 0.76312492 | -3.5124796 | -2.2157208 |
| CCDC171 | -0.1846777 | 3.5039994 | -2.2551775 | 0.02683424 | 0.76312492 | -3.5217026 | -2.2139206 |
| YWHAZP6 | -1.6196878 | -4.9736692 | -2.2514808 | 0.02690615 | 0.76312492 | -3.5206173 | -2.2128767 |
| INCA1 | 0.26003843 | 2.3350569 | 2.25358179 | 0.02693911 | 0.76312492 | -3.5258573 | 2.21239903 |
| MTX1P1 | 0.38375011 | 2.45216872 | 2.25346561 | 0.02694539 | 0.76312492 | -3.5072476 | 2.21230808 |
| GRM6 | -0.6013517 | 0.30036066 | -2.2516374 | 0.02694582 | 0.76312492 | -3.5234631 | -2.2123018 |
| NFE4 | -1.6553928 | -4.9400517 | -2.2492173 | 0.02697615 | 0.76312492 | -3.516454 | -2.2118628 |
| NPY2R | 1.06322942 | 0.66599146 | 2.2517717 | 0.02697907 | 0.76312492 | -3.5246909 | 2.21182044 |
| ZNF286A-TBC1D26 | -1.2022575 | -2.586717 | -2.2484907 | 0.02702827 | 0.76312492 | -3.519097 | -2.2111092 |
| HOXB-AS1 | -1.2695542 | -5.0080388 | -2.2513092 | 0.02704367 | 0.76312492 | -3.5295491 | -2.2108869 |
| VEPH1 | -0.7323007 | 2.70002984 | -2.2515578 | 0.0270504 | 0.76312492 | -3.5069408 | -2.2107898 |
| TNPO1-DT | -1.0882212 | -3.6550691 | -2.2494343 | 0.02706893 | 0.76312492 | -3.5090102 | -2.2105224 |
| C4orf19 | 0.27236518 | 2.09024479 | 2.25119339 | 0.02709937 | 0.76312492 | -3.5101937 | 2.2100834 |
| SLC11A2 | 0.27766048 | 6.75353592 | 2.25144514 | 0.02710391 | 0.76312492 | -3.5095362 | 2.21001806 |
| H3-4 | 1.39411921 | -4.0036111 | 2.24798924 | 0.02716116 | 0.76312492 | -3.5029894 | 2.20919382 |
| NLGN3 | -0.3110189 | 7.31999815 | -2.2498283 | 0.0271708 | 0.76312492 | -3.5135477 | -2.2090552 |
| PWRN1 | -1.0179439 | -0.6005652 | -2.2478242 | 0.02725755 | 0.76312492 | -3.5622503 | -2.2078096 |
| RALBP1 | 0.2036982 | 6.00264348 | 2.24750289 | 0.02730951 | 0.76312492 | -3.5146885 | 2.20706507 |
| GFPT2 | 0.50946156 | 4.16432006 | 2.24617347 | 0.02733542 | 0.76312492 | -3.5090092 | 2.20669442 |
| KMT5AP2 | -1.2416307 | -3.7184082 | -2.242076 | 0.02743296 | 0.76312492 | -3.5275891 | -2.2053013 |
| TEX38 | 1.14076944 | -2.0954255 | 2.24474312 | 0.02744296 | 0.76312492 | -3.5228244 | 2.20515876 |
| ROPN1B | -0.7563574 | -1.0893863 | -2.2444236 | 0.02745741 | 0.76312492 | -3.5232901 | -2.2049527 |
| ALG1 | 0.17464968 | 4.49154687 | 2.24528738 | 0.0274699 | 0.76312492 | -3.5231762 | 2.20477482 |
| PCAT1 | 0.87342456 | -0.9428229 | 2.24408463 | 0.02747918 | 0.76312492 | -3.5240898 | 2.20464264 |
| TNFRSF12A | 0.93228552 | 3.63749839 | 2.24554192 | 0.02749404 | 0.76312492 | -3.5027371 | 2.20443112 |
| MAB21L4 | -1.0655028 | -5.6443375 | -2.2446814 | 0.02749723 | 0.76312492 | -3.5328822 | -2.2043857 |
| DCDC2C | 1.35351129 | -4.0293002 | 2.24375432 | 0.02754602 | 0.76353069 | -3.5487712 | 2.20369182 |
| USP26 | 1.78234909 | -5.1780908 | 2.24102169 | 0.02759194 | 0.76385577 | -3.5079971 | 2.2030398 |
| LINC01678 | -1.4198643 | -3.5122368 | -2.2412079 | 0.02766718 | 0.76499079 | -3.5589936 | -2.2019735 |
| PTGDS | 1.25535125 | 1.97471768 | 2.2396414 | 0.02780094 | 0.7651867 | -3.5363359 | 2.2000839 |
| DUSP19 | -0.3569753 | 2.47921459 | -2.2394156 | 0.0278321 | 0.7651867 | -3.5165992 | -2.1996449 |
| XKR6 | -0.344751 | 3.01883112 | -2.240102 | 0.02785218 | 0.7651867 | -3.5364816 | -2.1993622 |
| LNX1 | 0.55680777 | 3.34886543 | 2.23854261 | 0.02786834 | 0.7651867 | -3.5402535 | 2.19913483 |
| FLNC | 0.61690406 | 7.31777489 | 2.23960167 | 0.02789468 | 0.7651867 | -3.5305747 | 2.19876446 |
| MAP1LC3C | 0.89176853 | -0.0408474 | 2.23894394 | 0.02791789 | 0.7651867 | -3.504176 | 2.19843831 |
| TAF7L | 0.67907523 | -0.2951725 | 2.23729473 | 0.02799353 | 0.7651867 | -3.5475932 | 2.19737698 |
| ARF4-AS1 | 1.33724658 | -5.6120047 | 2.23358794 | 0.0280138 | 0.7651867 | -3.5039852 | 2.19709308 |
| SNTB2 | 0.24237745 | 4.70277696 | 2.23751974 | 0.02802537 | 0.7651867 | -3.5358133 | 2.19693112 |
| PTGS1 | 1.077157 | 0.68164734 | 2.23614428 | 0.02804378 | 0.7651867 | -3.5530298 | 2.19667346 |
| FGF17 | -0.7694455 | 1.43763814 | -2.2369516 | 0.02805102 | 0.7651867 | -3.5416631 | -2.1965721 |
| ALPK1 | 0.8024016 | 3.58527801 | 2.23626214 | 0.02814194 | 0.76673048 | -3.531347 | 2.19530208 |
| PEX11G | 0.3475291 | 1.48006284 | 2.23414664 | 0.02827343 | 0.76854916 | -3.537658 | 2.19347149 |
| DNAH6 | -0.3460659 | 4.2238867 | -2.2337978 | 0.02827749 | 0.76854916 | -3.5326797 | -2.193415 |
| PDCL3P5 | 0.6987573 | -1.2965573 | 2.2312593 | 0.02841278 | 0.76862298 | -3.5298592 | 2.19153943 |
| NMRK1 | 0.27123979 | 3.07699989 | 2.23145734 | 0.02842073 | 0.76862298 | -3.5371415 | 2.19142943 |
| OTOGL | -0.7134379 | -0.6618375 | -2.2291371 | 0.02850355 | 0.76862298 | -3.568522 | -2.1902854 |
| MPRIPP1 | -0.3976086 | 0.91189568 | -2.2289606 | 0.02854783 | 0.76862298 | -3.5470091 | -2.1896748 |
| EFNA1 | 0.62833349 | 2.67256081 | 2.22985051 | 0.02855041 | 0.76862298 | -3.5215788 | 2.18963926 |
| THBS2-AS1 | 0.35235257 | 0.92861266 | 2.22765405 | 0.02855869 | 0.76862298 | -3.5445847 | 2.1895252 |
| FAM86B1 | 0.25270409 | 2.60377316 | 2.22907517 | 0.02860748 | 0.76862298 | -3.5465145 | 2.18885359 |
| JCHAIN | 1.59468868 | -5.0394275 | 2.22450474 | 0.02873019 | 0.76862298 | -3.5611145 | 2.18716901 |
| RAD51D | 0.16900552 | 4.07428029 | 2.22636346 | 0.02874217 | 0.76862298 | -3.543394 | 2.18700478 |
| GLB1 | 0.34849935 | 5.73157207 | 2.22606523 | 0.02878732 | 0.76862298 | -3.5435881 | 2.18638684 |
| KCNK2 | 0.44840224 | 4.46502964 | 2.22553104 | 0.02882104 | 0.76862298 | -3.5615115 | 2.1859257 |
| RNU6-584P | -1.5180796 | -5.743723 | -2.2231308 | 0.02883258 | 0.76862298 | -3.5514907 | -2.185768 |
| PELP1-DT | 0.99970241 | -3.8195012 | 2.22333763 | 0.02884698 | 0.76862298 | -3.5302216 | 2.18557141 |
| ITGB3 | 0.99419815 | 2.80441472 | 2.22548819 | 0.02885333 | 0.76862298 | -3.5260764 | 2.18548463 |
| GOLGA4-AS1 | 0.6006736 | 0.56151823 | 2.22485128 | 0.02893187 | 0.76862298 | -3.5349314 | 2.18441365 |
| ELOVL2 | -0.3491046 | 5.78222787 | -2.2236242 | 0.02895156 | 0.76862298 | -3.5491771 | -2.1841455 |
| GABRG3 | 0.53118856 | 5.78821834 | 2.22343371 | 0.02897964 | 0.76862298 | -3.5568547 | 2.18376343 |
| ARR3 | 0.5571365 | -0.6229455 | 2.2230383 | 0.02902114 | 0.76862298 | -3.5780083 | 2.18319923 |
| MIF4GD | 0.21796657 | 4.5703625 | 2.22203441 | 0.02911495 | 0.76862298 | -3.5533227 | 2.18192657 |
| RPL7AP63 | -0.632569 | -0.9392639 | -2.2203422 | 0.02915006 | 0.76862298 | -3.5583986 | -2.1814512 |
| NAV2-IT1 | -1.4160276 | -5.7782263 | -2.2182521 | 0.02917838 | 0.76862298 | -3.5512846 | -2.1810681 |
| ABCC4 | 0.62629876 | 4.12882231 | 2.21987033 | 0.02919241 | 0.76862298 | -3.5498241 | 2.18087839 |
| REM1 | -1.4319443 | -6.1312676 | -2.2193831 | 0.0292284 | 0.76862298 | -3.5552493 | -2.1803922 |
| LINC02207 | 1.28949576 | -3.3221656 | 2.21903052 | 0.02923977 | 0.76862298 | -3.5783904 | 2.18023873 |
| ELMOD2 | 0.20367735 | 5.52623194 | 2.21867589 | 0.02926632 | 0.76862298 | -3.5544271 | 2.17988051 |
| SPRY3 | -0.9394529 | -1.1709537 | -2.2192986 | 0.02929524 | 0.76862298 | -3.5919222 | -2.1794906 |
| LATS2 | 0.31672207 | 3.73418483 | 2.21933983 | 0.02929873 | 0.76862298 | -3.5441015 | 2.17944355 |
| SNHG16 | 0.24836064 | 4.40597867 | 2.21702886 | 0.02940065 | 0.76862298 | -3.5615522 | 2.1780723 |
| SYNGR2 | 0.39759096 | 4.12628624 | 2.2176789 | 0.02940068 | 0.76862298 | -3.5542427 | 2.17807202 |
| MVP | 0.54993182 | 4.97711851 | 2.21818864 | 0.02940604 | 0.76862298 | -3.5497211 | 2.17800001 |
| TBILA | 1.60743792 | -1.3321229 | 2.21711991 | 0.02941677 | 0.76862298 | -3.5850662 | 2.17785581 |
| PNO1 | 0.2673944 | 4.06095674 | 2.21603243 | 0.02947083 | 0.76862298 | -3.5585121 | 2.17713053 |
| FBXW9 | 0.22827738 | 5.00830022 | 2.2160131 | 0.02953014 | 0.76862298 | -3.5567433 | 2.17633602 |
| AKR1C8 | -1.5382955 | -3.935131 | -2.214094 | 0.02960005 | 0.76862298 | -3.6055615 | -2.1754013 |
| NOD1 | 0.37980036 | 2.47907834 | 2.21359998 | 0.02966206 | 0.76862298 | -3.5592034 | 2.17457399 |
| B4GALT5 | 0.3222786 | 7.62848635 | 2.21228162 | 0.02969392 | 0.76862298 | -3.5654142 | 2.17414935 |
| MYL6B-AS1 | 1.17932009 | -3.3023874 | 2.21071721 | 0.02971187 | 0.76862298 | -3.5859946 | 2.1739103 |
| NAGA | 0.32337675 | 4.87808974 | 2.21241021 | 0.02976397 | 0.76862298 | -3.5587949 | 2.17321731 |
| PEMT | 0.20488792 | 4.10556521 | 2.21140541 | 0.02976554 | 0.76862298 | -3.5665007 | 2.17319642 |
| PSMD6-AS2 | -0.50687 | 0.2350725 | -2.2124456 | 0.02978935 | 0.76862298 | -3.5876938 | -2.1728801 |
| FOSL2 | 0.70922601 | 6.07696032 | 2.21235107 | 0.02985669 | 0.76862298 | -3.574665 | 2.17198643 |
| PPDPF | -0.283173 | 7.00707955 | -2.2102018 | 0.02988824 | 0.76862298 | -3.5674763 | -2.1715683 |
| SIRPA | -0.3208434 | 5.58829682 | -2.2096377 | 0.02990967 | 0.76862298 | -3.5710086 | -2.1712845 |
| SNX30-DT | 0.73393893 | -1.3959026 | 2.21005667 | 0.02991158 | 0.76862298 | -3.5784116 | 2.17125925 |
| IL17RE | 0.42296505 | 0.68736284 | 2.20938867 | 0.02991849 | 0.76862298 | -3.5752791 | 2.17116778 |
| AMER2 | -0.4440142 | 6.66167828 | -2.2099802 | 0.02995433 | 0.76862298 | -3.5701865 | -2.1706938 |
| TMEM62 | 0.23736585 | 4.05455559 | 2.20905713 | 0.02995552 | 0.76862298 | -3.5697229 | 2.17067808 |
| MPZ | -1.0761431 | -1.1492694 | -2.2096535 | 0.02999119 | 0.76862298 | -3.5996441 | -2.1702067 |
| SSTR5 | -1.6572199 | -4.3433949 | -2.2079234 | 0.03002557 | 0.76862298 | -3.5766799 | -2.1697529 |
| AKAP13-AS1 | -0.7148795 | -2.0252391 | -2.2083288 | 0.03005115 | 0.76862298 | -3.6084225 | -2.1694155 |
| DAO | -0.9788761 | -2.0067016 | -2.2081906 | 0.03006204 | 0.76862298 | -3.5992915 | -2.169272 |
| RNF144B | 0.77643 | -0.0789587 | 2.20733141 | 0.03006922 | 0.76862298 | -3.5725745 | 2.16917735 |
| RPL22L1 | 0.28906203 | 2.39455067 | 2.20650193 | 0.03015433 | 0.76939636 | -3.5525208 | 2.16805728 |
| FNTAP2 | -1.4884738 | -5.3181895 | -2.2035268 | 0.03019676 | 0.76939636 | -3.579542 | -2.1674999 |
| SUMF2 | 0.15204418 | 6.12659177 | 2.2052649 | 0.03031045 | 0.76939636 | -3.5762171 | 2.16600966 |
| FAM90A20P | -1.2367159 | -3.5381039 | -2.2047616 | 0.03035331 | 0.76939636 | -3.5787508 | -2.165449 |
| TOMM40P2 | 1.55096325 | -5.6497794 | 2.20185582 | 0.03038282 | 0.76939636 | -3.5547709 | 2.16506356 |
| PLPP2 | -1.0648901 | -1.5242795 | -2.2007311 | 0.03053839 | 0.76939636 | -3.588574 | -2.1630363 |
| PTPRE | 0.48317784 | 5.04197506 | 2.20077134 | 0.03060974 | 0.76939636 | -3.5801663 | 2.16210944 |
| LINC01054 | -1.2240622 | -3.2803135 | -2.1987741 | 0.03065197 | 0.76939636 | -3.5863506 | -2.1615618 |
| LINC01068 | -1.1989648 | -3.4718026 | -2.1964809 | 0.0306605 | 0.76939636 | -3.6025906 | -2.1614512 |
| DHRS1 | 0.27006164 | 4.74357119 | 2.19900023 | 0.03082032 | 0.76939636 | -3.5873462 | 2.15938486 |
| BNIP3P10 | -1.4930596 | -3.6877471 | -2.1942832 | 0.03083001 | 0.76939636 | -3.5921271 | -2.1592599 |
| FLII | 0.16617006 | 7.138228 | 2.19741335 | 0.03084069 | 0.76939636 | -3.5847433 | 2.15912214 |
| TRMT61A | 0.17699665 | 4.86824637 | 2.19727573 | 0.03088986 | 0.76939636 | -3.5905114 | 2.15848866 |
| NRSN2-AS1 | -0.2316453 | 3.617381 | -2.1957412 | 0.0309866 | 0.76939636 | -3.5827119 | -2.1572447 |
| DNAJB12 | 0.11808806 | 6.08369564 | 2.19595724 | 0.03098797 | 0.76939636 | -3.5920488 | 2.15722719 |
| FOXA1 | 1.15393055 | -0.086567 | 2.1946508 | 0.0310192 | 0.76939636 | -3.5994822 | 2.15682635 |
| PI4K2B | 0.2751875 | 3.67883016 | 2.1948745 | 0.03104998 | 0.76939636 | -3.5966211 | 2.15643165 |
| AMPD3 | 0.60115615 | 1.21173871 | 2.19344982 | 0.03108942 | 0.76939636 | -3.5820812 | 2.1559263 |
| MIR4754 | 1.03750727 | -3.2499722 | 2.19031094 | 0.0311254 | 0.76939636 | -3.5956804 | 2.1554658 |
| NUDCP2 | -0.6874075 | -1.458015 | -2.1898228 | 0.03116385 | 0.76939636 | -3.5897171 | -2.1549743 |
| PCGF6 | 0.22096728 | 3.55853282 | 2.19231265 | 0.03121885 | 0.76939636 | -3.5928016 | 2.15427197 |
| CEP250 | -0.2324819 | 6.49929391 | -2.1924487 | 0.03122711 | 0.76939636 | -3.5951783 | -2.1541666 |
| HS3ST3A1 | 0.62923073 | 3.37043904 | 2.19210677 | 0.0312278 | 0.76939636 | -3.5766253 | 2.15415784 |
| ORAI1 | 0.31652546 | 3.43303135 | 2.18920621 | 0.03141808 | 0.76939636 | -3.5826176 | 2.15173688 |
| ZNF248-AS1 | 0.5497747 | -1.4179146 | 2.18838312 | 0.03142203 | 0.76939636 | -3.6021043 | 2.15168683 |
| NFKB2 | 0.4931698 | 4.05047895 | 2.18899476 | 0.03152441 | 0.76939636 | -3.5811999 | 2.15038957 |
| KAZN | 0.39333647 | 7.10143829 | 2.18800558 | 0.03154731 | 0.76939636 | -3.594535 | 2.15009991 |
| FUCA1 | 0.21753186 | 4.87353166 | 2.18631857 | 0.03156345 | 0.76939636 | -3.5995417 | 2.14989595 |
| GOLGA8T | 0.55080356 | -0.2700379 | 2.18717798 | 0.0315807 | 0.76939636 | -3.6301568 | 2.14967788 |
| CD44 | 0.98732842 | 6.55404201 | 2.18813415 | 0.03158906 | 0.76939636 | -3.5899943 | 2.14957234 |
| SNORA35B | -1.3708215 | -5.3964848 | -2.1864323 | 0.03163581 | 0.76939636 | -3.6030195 | -2.1489822 |
| MIR6835 | -1.2217492 | -4.4050695 | -2.1840873 | 0.03166514 | 0.76939636 | -3.6053686 | -2.1486123 |
| EFEMP2 | 0.65984878 | 5.75600063 | 2.18661697 | 0.03171916 | 0.76939636 | -3.6025908 | 2.14793192 |
| CYP27A1 | 0.73180841 | 3.53626595 | 2.18391433 | 0.03187448 | 0.76939636 | -3.5950337 | 2.14598115 |
| DPPA2 | -1.3524527 | -5.9814081 | -2.182561 | 0.03187686 | 0.76939636 | -3.6157605 | -2.1459514 |
| TUBB6 | 0.43459484 | 5.48389897 | 2.18346621 | 0.03190624 | 0.76939636 | -3.5936902 | 2.1455833 |
| CD9 | 0.5232586 | 5.59773467 | 2.18324131 | 0.03196115 | 0.76939636 | -3.5985799 | 2.14489612 |
| LCIIAR | 0.56434031 | 0.38310502 | 2.17988078 | 0.03203763 | 0.76939636 | -3.6063014 | 2.14394085 |
| CELSR3 | -0.2540715 | 7.65439962 | -2.1821701 | 0.03203795 | 0.76939636 | -3.6109164 | -2.1439368 |
| RFT1 | 0.16610201 | 4.79032712 | 2.18211395 | 0.03204987 | 0.76939636 | -3.6076462 | 2.14378808 |
| MT1M | 1.12703747 | -1.3518066 | 2.18182695 | 0.03205365 | 0.76939636 | -3.6200734 | 2.14374097 |
| PLD5 | -0.4475894 | 4.30648519 | -2.1803547 | 0.03209354 | 0.76939636 | -3.6079313 | -2.1432436 |
| MYL11 | -1.0903162 | -2.8632952 | -2.1813188 | 0.03224887 | 0.76939636 | -3.612751 | -2.1413123 |
| PRR16 | 0.45456986 | 1.61424441 | 2.17777588 | 0.03225265 | 0.76939636 | -3.5952435 | 2.14126535 |
| LINC01353 | -1.3707398 | -3.7182537 | -2.1750842 | 0.03235383 | 0.76939636 | -3.5883171 | -2.1400116 |
| CHCHD10 | 0.43645488 | 4.33277305 | 2.17599122 | 0.0323965 | 0.76939636 | -3.6087839 | 2.13948389 |
| HIGD1AP18 | -1.4024809 | -5.4558109 | -2.1746459 | 0.03248456 | 0.76939636 | -3.612636 | -2.1383967 |
| TBXT | -1.4432868 | -5.6787866 | -2.1747291 | 0.0325063 | 0.76939636 | -3.6410477 | -2.1381287 |
| ACTL7A | -1.1319843 | -6.4623904 | -2.171352 | 0.03255052 | 0.76939636 | -3.6110259 | -2.137584 |
| BNIP3P37 | -1.6987522 | -4.8614891 | -2.1735515 | 0.03258902 | 0.76939636 | -3.6437285 | -2.1371103 |
| SNRNP35 | -0.2131738 | 3.4976191 | -2.1721153 | 0.03258953 | 0.76939636 | -3.6100427 | -2.1371041 |
| FZD6 | 0.71416296 | 4.7366358 | 2.17476082 | 0.0325965 | 0.76939636 | -3.6146772 | 2.13701828 |
| SPDYE12 | 0.35012985 | 0.73050673 | 2.17236425 | 0.0326646 | 0.76939636 | -3.6156819 | 2.13618182 |
| MAP3K15 | -0.5063139 | 0.17865612 | -2.172338 | 0.03267013 | 0.76939636 | -3.6084444 | -2.1361139 |
| CBFA2T2 | -0.1539545 | 6.70011075 | -2.1736075 | 0.03268943 | 0.76939636 | -3.6203552 | -2.1358772 |
| PLBD2 | 0.29241102 | 6.70204316 | 2.17210907 | 0.03269164 | 0.76939636 | -3.620235 | 2.13585003 |
| LYPLAL1-DT | -0.44443 | -0.4097677 | -2.171677 | 0.03270688 | 0.76939636 | -3.6085156 | -2.1356631 |
| PAIP2B | -0.3311834 | 4.36749953 | -2.1730638 | 0.03272196 | 0.76939636 | -3.6265456 | -2.1354784 |
| TRAF6 | -0.1609276 | 5.28643601 | -2.1727039 | 0.03276625 | 0.76939636 | -3.6211795 | -2.1349359 |
| HOXB9 | -1.8238777 | -5.9076442 | -2.1707099 | 0.03276967 | 0.76939636 | -3.6063827 | -2.134894 |
| PPIAP67 | -1.3088621 | -3.5981284 | -2.1717907 | 0.03278122 | 0.76939636 | -3.6464528 | -2.1347527 |
| ZBED3-AS1 | 0.44422107 | 3.54599067 | 2.17173088 | 0.03279151 | 0.76939636 | -3.6163636 | 2.13462673 |
| KAT2B | -0.3180777 | 5.80643235 | -2.1717457 | 0.03279579 | 0.76939636 | -3.6177624 | -2.1345744 |
| ERICH2 | -0.3632653 | 2.81780584 | -2.1705231 | 0.03283393 | 0.76939636 | -3.6175258 | -2.1341081 |
| PRSS56 | 1.57268904 | -1.1130076 | 2.16973916 | 0.03284213 | 0.76939636 | -3.6005339 | 2.13400793 |
| CDC20-DT | 1.44508916 | -4.1920568 | 2.16873114 | 0.0328449 | 0.76939636 | -3.6226568 | 2.13397416 |
| ZNF285 | -0.2841736 | 3.95654689 | -2.1707769 | 0.03287975 | 0.76939636 | -3.634109 | -2.1335486 |
| CPA4 | 0.97814167 | -0.9883746 | 2.16926347 | 0.03290254 | 0.76939636 | -3.6121274 | 2.13327059 |
| AVPR1A | 1.27142597 | 1.67618208 | 2.17049425 | 0.03290272 | 0.76939636 | -3.6321633 | 2.13326832 |
| SAR1AP4 | -1.6380817 | -5.7856962 | -2.1662863 | 0.03294751 | 0.76939636 | -3.6265593 | -2.1327223 |
| ZNF516-AS1 | -0.442505 | 0.05280566 | -2.1680427 | 0.03295627 | 0.76939636 | -3.6262182 | -2.1326156 |
| ACOT9 | 0.32038615 | 4.97532199 | 2.16937932 | 0.03296378 | 0.76939636 | -3.6196538 | 2.13252418 |
| DNASE1L1 | 0.44535808 | 3.19487481 | 2.17009545 | 0.03297175 | 0.76939636 | -3.6224723 | 2.13242716 |
| RPS6KA4 | 0.2415164 | 4.80941897 | 2.16927475 | 0.03301868 | 0.76939636 | -3.6226201 | 2.13185602 |
| ITGA1 | 0.90077908 | 0.31398891 | 2.16939165 | 0.03302806 | 0.76939636 | -3.6075443 | 2.13174203 |
| ME3 | 0.34960511 | 3.86233165 | 2.16831872 | 0.03303277 | 0.76939636 | -3.6244489 | 2.13168471 |
| CBX8 | -0.2049961 | 5.06117406 | -2.1685132 | 0.03307924 | 0.76939636 | -3.6314071 | -2.1311202 |
| LMAN1L | -1.4137382 | -5.2845784 | -2.1665697 | 0.03311975 | 0.76939636 | -3.6392666 | -2.1306287 |
| CD44-DT | 1.07026217 | -1.7881412 | 2.16867377 | 0.03312262 | 0.76939636 | -3.613969 | 2.13059379 |
| MEIS3P1 | 0.45600218 | 2.78949514 | 2.16764967 | 0.03317096 | 0.76939636 | -3.6247695 | 2.13000786 |
| LNCARSR | 1.40363623 | -6.1154299 | 2.16368881 | 0.03319268 | 0.76939636 | -3.6112986 | 2.12974493 |
| ARID5B | 0.39006716 | 5.33354152 | 2.16565457 | 0.03320412 | 0.76939636 | -3.6224243 | 2.12960638 |
| OR52I1 | -1.1846464 | -6.6416528 | -2.1629002 | 0.03321521 | 0.76939636 | -3.611344 | -2.1294722 |
| CSAG3 | 1.82135098 | -3.7102597 | 2.16707715 | 0.03321868 | 0.76939636 | -3.5864553 | 2.12943019 |
| RASGRF2-AS1 | 1.27583388 | -2.4805882 | 2.16439924 | 0.03324809 | 0.76939636 | -3.6373135 | 2.12907464 |
| CTSO | 0.70276847 | 4.65836976 | 2.16639691 | 0.03327648 | 0.76939636 | -3.6188988 | 2.12873151 |
| JMJD6 | -0.1999431 | 5.13579328 | -2.1659711 | 0.03330613 | 0.76939636 | -3.6380146 | -2.1283735 |
| CA5BP1 | -1.1411177 | -3.5670939 | -2.163739 | 0.0333263 | 0.76939636 | -3.6208085 | -2.1281301 |
| KRT18P27 | 1.28521682 | -6.6422797 | 2.16204126 | 0.03343141 | 0.76939636 | -3.624379 | 2.1268637 |
| MIDEAS | 0.27241613 | 6.19927836 | 2.16335959 | 0.03348003 | 0.76939636 | -3.6314337 | 2.12627906 |
| SLC25A10 | -0.3065669 | 1.63553893 | -2.1625132 | 0.03350333 | 0.76939636 | -3.6359532 | -2.1259992 |
| ZMAT4 | 0.42764328 | 3.89437075 | 2.1629415 | 0.03352045 | 0.76939636 | -3.6317949 | 2.12579356 |
| BID | 0.29129747 | 4.82467173 | 2.1609219 | 0.03355437 | 0.76939636 | -3.6341375 | 2.12538661 |
| SHANK2-AS1 | -1.6520874 | -4.6911538 | -2.1584863 | 0.03356701 | 0.76939636 | -3.6206994 | -2.125235 |
| RAB20 | 0.91998362 | -0.1739545 | 2.16200582 | 0.03356717 | 0.76939636 | -3.6183178 | 2.12523301 |
| PROS1 | 0.66890008 | 5.36478834 | 2.16226426 | 0.03358175 | 0.76939636 | -3.6219592 | 2.12505825 |
| H2AC14 | -1.1114133 | -6.6666084 | -2.1602407 | 0.03359185 | 0.76939636 | -3.6233032 | -2.1249372 |
| PIGA | 0.21680245 | 3.54011397 | 2.16038113 | 0.03366353 | 0.76939636 | -3.6330426 | 2.12407911 |
| SLC18B1 | 0.47830434 | 6.09320385 | 2.16056634 | 0.03371229 | 0.76939636 | -3.6347374 | 2.12349629 |
| CEP78 | -0.2678803 | 6.09723955 | -2.1617999 | 0.03373517 | 0.76939636 | -3.6315753 | -2.123223 |
| SSR3 | 0.27151512 | 6.85113424 | 2.15957786 | 0.03375262 | 0.76939636 | -3.6342855 | 2.12301466 |
| ARHGEF7-AS2 | -0.376833 | 0.26468087 | -2.1602594 | 0.03378715 | 0.76939636 | -3.6512414 | -2.1226028 |
| HLA-C | 0.73079067 | 7.3586788 | 2.15932933 | 0.03382707 | 0.76939636 | -3.6332448 | 2.12212709 |
| BEGAIN | 0.45312003 | 5.83154139 | 2.15811175 | 0.03387261 | 0.76939636 | -3.6472427 | 2.1215849 |
| TAS2R4 | -0.5047156 | 0.36952885 | -2.1553453 | 0.03398707 | 0.76939636 | -3.6439795 | -2.120225 |
| RDH13 | -0.2138338 | 4.00905311 | -2.1568849 | 0.03398922 | 0.76939636 | -3.647111 | -2.1201995 |
| MORC2 | -0.1528001 | 6.11412795 | -2.1570659 | 0.03399817 | 0.76939636 | -3.6434351 | -2.1200934 |
| FRMD1 | 1.41976571 | -6.0858175 | 2.15578289 | 0.03402154 | 0.76939636 | -3.6280231 | 2.11981625 |
| HPS1-AS1 | 0.78585974 | -1.1882908 | 2.15512642 | 0.03417309 | 0.76939636 | -3.6632831 | 2.11802337 |
| AK3 | 0.19425642 | 6.49645368 | 2.15469621 | 0.03420466 | 0.76939636 | -3.6434503 | 2.11765074 |
| GZF1 | -0.1281945 | 4.9322334 | -2.1537992 | 0.0342608 | 0.76939636 | -3.6454283 | -2.1169888 |
| PDLIM4 | 0.65464764 | 5.42353992 | 2.15397525 | 0.03426741 | 0.76939636 | -3.6355576 | 2.11691087 |
| ECHDC3 | 0.6889422 | 0.61509995 | 2.15280274 | 0.03428271 | 0.76939636 | -3.6367685 | 2.11673067 |
| FKBP14-AS1 | 0.39317177 | 0.08798896 | 2.15119246 | 0.03428284 | 0.76939636 | -3.643655 | 2.11672914 |
| MXRA7 | 0.27959897 | 6.90233052 | 2.15320144 | 0.03429716 | 0.76939636 | -3.6449908 | 2.1165606 |
| RPS6KA3 | 0.20207761 | 5.69575325 | 2.15087887 | 0.03432882 | 0.76939636 | -3.6394372 | 2.11618803 |
| ERRFI1 | -0.3804543 | 5.13121143 | -2.151064 | 0.03435127 | 0.76939636 | -3.6381293 | -2.115924 |
| OXSM | 0.19386744 | 2.52670161 | 2.15142661 | 0.03444577 | 0.76939636 | -3.6422092 | 2.11481445 |
| DDOST | 0.23146114 | 7.39463374 | 2.15052201 | 0.03450728 | 0.76939636 | -3.6474815 | 2.1140935 |
| LINC00381 | -1.4152944 | -4.5077522 | -2.1485145 | 0.03453284 | 0.76939636 | -3.645503 | -2.1137943 |
| CRACR2A | 0.8841589 | -0.1761363 | 2.1485847 | 0.0345347 | 0.76939636 | -3.6538599 | 2.11377254 |
| ELOVL1 | 0.3643348 | 4.57752463 | 2.1499045 | 0.03458172 | 0.76939636 | -3.6469885 | 2.11322259 |
| CACNG8 | -0.3357855 | 6.87288782 | -2.1486488 | 0.03465931 | 0.76939636 | -3.6512157 | -2.1123165 |
| RAP1BL | -0.5693885 | -1.6989996 | -2.1461852 | 0.03471704 | 0.76939636 | -3.6517515 | -2.1116435 |
| ACTN1 | 0.32335489 | 6.96398696 | 2.14822777 | 0.03472594 | 0.76939636 | -3.6517065 | 2.11153976 |
| PTGR1 | 0.37438916 | 3.81688179 | 2.14787971 | 0.03473987 | 0.76939636 | -3.6448033 | 2.11137759 |
| BABAM2 | 0.21514149 | 4.61479603 | 2.14671261 | 0.03474236 | 0.76939636 | -3.6483733 | 2.11134859 |
| CXCL13 | -1.4097781 | -5.1775623 | -2.1465718 | 0.03474786 | 0.76939636 | -3.6601748 | -2.1112845 |
| KLF10 | 0.38222778 | 4.74156098 | 2.14748755 | 0.03477047 | 0.76939636 | -3.666633 | 2.11102138 |
| BOLA2P3 | -1.2747929 | -6.1163649 | -2.1454097 | 0.03479193 | 0.76939636 | -3.648326 | -2.1107718 |
| HLA-DRB1 | 1.28803084 | -0.8308068 | 2.14696331 | 0.03483068 | 0.76939636 | -3.6290643 | 2.11032142 |
| TNK2-AS1 | -0.8518544 | -2.2160671 | -2.1456022 | 0.03483352 | 0.76939636 | -3.6522997 | -2.1102884 |
| GPX4 | 0.23747704 | 7.39649469 | 2.1454768 | 0.03489745 | 0.76939636 | -3.6437656 | 2.10954633 |
| SLC35B1 | 0.20136214 | 6.00446628 | 2.14597107 | 0.03490868 | 0.76939636 | -3.6536634 | 2.10941609 |
| SUMF1 | 0.4557373 | 4.78924783 | 2.14555853 | 0.03493755 | 0.76939636 | -3.6488893 | 2.10908151 |
| NRIP2 | -0.3471154 | 1.6124917 | -2.1436497 | 0.03499774 | 0.76939636 | -3.6658283 | -2.1083845 |
| PRR12 | -0.1927283 | 7.40675449 | -2.1446866 | 0.03501526 | 0.76939636 | -3.6587644 | -2.1081819 |
| LINC00963 | 0.46984898 | 5.25301117 | 2.14476015 | 0.03502463 | 0.76939636 | -3.6576453 | 2.10807347 |
| GALNT3 | 0.42404697 | 1.77929546 | 2.14502088 | 0.03502754 | 0.76939636 | -3.6820516 | 2.10803988 |
| TPRG1 | 0.7956711 | -0.7458755 | 2.14379207 | 0.03509631 | 0.76939636 | -3.651028 | 2.10724543 |
| SLC35B3 | 0.14353346 | 4.15937822 | 2.14251695 | 0.03518901 | 0.76939636 | -3.6618021 | 2.10617664 |
| PIM1 | 0.27571537 | 4.46731209 | 2.14104725 | 0.03522656 | 0.76939636 | -3.6656723 | 2.10574439 |
| SLC10A4 | 0.29763672 | 3.37046632 | 2.14016587 | 0.0352787 | 0.76939636 | -3.6591216 | 2.10514488 |
| MAP3K7 | 0.14125766 | 6.44174092 | 2.13996458 | 0.03531633 | 0.76939636 | -3.6545287 | 2.10471262 |
| ALDH3B1 | 0.44481717 | 3.50381439 | 2.1404538 | 0.03540285 | 0.76939636 | -3.6438045 | 2.10372037 |
| PDE7B | 0.69241734 | 4.02214998 | 2.13816366 | 0.03543058 | 0.76939636 | -3.6618715 | 2.1034027 |
| RPL27P12 | 1.25478855 | -6.4317257 | 2.13551506 | 0.0355032 | 0.76939636 | -3.6481587 | 2.10257198 |
| BTBD17 | -0.6857203 | 4.18474448 | -2.1393908 | 0.03552056 | 0.76939636 | -3.658219 | -2.1023737 |
| RABGAP1 | -0.1664507 | 7.13840225 | -2.1370768 | 0.03556628 | 0.76939636 | -3.6646943 | -2.1018516 |
| LINC01854 | 1.33341228 | -4.6297594 | 2.13708995 | 0.03558943 | 0.76939636 | -3.6631672 | 2.1015875 |
| CCDC148 | -0.3931982 | 1.54186071 | -2.1362304 | 0.03559448 | 0.76939636 | -3.6615673 | -2.1015299 |
| FAM81A | -0.2680171 | 3.28393785 | -2.1368841 | 0.03560586 | 0.76939636 | -3.6557016 | -2.1014001 |
| GSTP1P1 | 1.34146791 | -5.7300801 | 2.13327065 | 0.03566061 | 0.76939636 | -3.6456933 | 2.10077632 |
| IGBP1P1 | 1.58127001 | -4.2018758 | 2.13398826 | 0.03567405 | 0.76939636 | -3.6733515 | 2.1006233 |
| LCLAT1 | 0.23814508 | 4.59519312 | 2.13548355 | 0.03570533 | 0.76939636 | -3.6695548 | 2.1002674 |
| GABBR2 | -0.4755526 | 8.81516209 | -2.1357343 | 0.03571651 | 0.76939636 | -3.6676509 | -2.1001402 |
| RLIMP1 | 1.30612966 | -6.5274246 | 2.13289937 | 0.03578178 | 0.76939636 | -3.6593404 | 2.09939857 |
| CAPN1 | 0.14711638 | 7.5385975 | 2.1339664 | 0.03578398 | 0.76939636 | -3.6689879 | 2.09937359 |
| LINC02813 | 0.91260488 | -1.4647137 | 2.13204023 | 0.03579264 | 0.76939636 | -3.6888396 | 2.09927531 |
| PDLIM7-AS1 | -1.5053752 | -5.0627317 | -2.1317277 | 0.03579857 | 0.76939636 | -3.6593212 | -2.099208 |
| ZNF114 | 0.38298239 | 1.76220735 | 2.13370948 | 0.03580911 | 0.76939636 | -3.6631223 | 2.09908835 |
| DLGAP1-AS4 | -1.4016273 | -5.7841928 | -2.1340762 | 0.03584152 | 0.76939636 | -3.6671919 | -2.0987208 |
| VCL | 0.28032901 | 7.0519282 | 2.13443321 | 0.03585903 | 0.76939636 | -3.6700519 | 2.09852233 |
| RAG2 | -1.4615799 | -5.42766 | -2.1328339 | 0.03586036 | 0.76939636 | -3.6769358 | -2.0985073 |
| KIFC3 | 0.28500933 | 5.93142972 | 2.13226175 | 0.03586826 | 0.76939636 | -3.6682883 | 2.09841776 |
| GATM | 0.40295844 | 6.09017965 | 2.13425564 | 0.03590485 | 0.76939636 | -3.6715221 | 2.09800336 |
| PI4K2A | 0.19853704 | 5.17917442 | 2.13282265 | 0.03596977 | 0.76939636 | -3.6673996 | 2.09726899 |
| CRTC3 | 0.26733497 | 6.23534364 | 2.13248971 | 0.03602422 | 0.76939636 | -3.6721432 | 2.09665396 |
| TMX4-AS1 | -0.6871376 | -1.7759229 | -2.1304534 | 0.03606535 | 0.76939636 | -3.6669647 | -2.0961898 |
| SMG7-AS1 | -0.370709 | 1.5712217 | -2.1311318 | 0.03607429 | 0.76939636 | -3.6842199 | -2.0960891 |
| LINC02977 | 0.34550613 | 3.2304383 | 2.13074689 | 0.03616038 | 0.76939636 | -3.6731416 | 2.09511944 |
| IFNE | 1.39372448 | -6.2687539 | 2.12914956 | 0.03617326 | 0.76939636 | -3.6752071 | 2.09497446 |
| TNFRSF14 | 0.79966929 | 0.81086626 | 2.13013662 | 0.03618989 | 0.76939636 | -3.6849089 | 2.09478745 |
| CTSLP2 | -0.8567719 | -2.5598293 | -2.1284563 | 0.03628962 | 0.76939636 | -3.6848514 | -2.0936674 |
| MESTIT1 | -0.4837305 | 0.07474823 | -2.1274428 | 0.03636533 | 0.76939636 | -3.6729915 | -2.0928188 |
| FAM246C | -0.468577 | 0.05949897 | -2.1296478 | 0.03636539 | 0.76939636 | -3.7047031 | -2.0928181 |
| NEK1 | -0.1890019 | 5.54927099 | -2.1270023 | 0.03642902 | 0.76939636 | -3.6803219 | -2.0921062 |
| TMBIM4 | 0.27026895 | 4.36418874 | 2.12757871 | 0.03644255 | 0.76939636 | -3.6806723 | 2.0919549 |
| CPD | 0.48571347 | 6.67871901 | 2.12635952 | 0.03645763 | 0.76939636 | -3.6776314 | 2.09178639 |
| DEPDC5 | 0.1305113 | 4.28842172 | 2.12562909 | 0.03655092 | 0.76939636 | -3.6847506 | 2.09074511 |
| LEPROT | 0.23679631 | 6.3910922 | 2.12594481 | 0.03658804 | 0.76939636 | -3.6800197 | 2.09033137 |
| CTLA4 | -1.3056731 | -6.1217289 | -2.1243615 | 0.03665735 | 0.76939636 | -3.6802056 | -2.0895599 |
| TXNRD2 | 0.24754713 | 3.14225248 | 2.12513342 | 0.03666528 | 0.76939636 | -3.677857 | 2.08947175 |
| HM13-AS1 | 1.19990018 | -3.0840262 | 2.1218289 | 0.03666561 | 0.76939636 | -3.6850819 | 2.08946805 |
| WDR45B | 0.16006533 | 7.26702823 | 2.12538247 | 0.0366687 | 0.76939636 | -3.681877 | 2.08943366 |
| COLGALT2 | -0.5195947 | 6.17269414 | -2.1233649 | 0.03674274 | 0.76939636 | -3.6803835 | -2.0886112 |
| H2BC6 | -0.7364926 | -1.348954 | -2.1221165 | 0.03679487 | 0.76939636 | -3.6702781 | -2.0880328 |
| EEPD1 | -0.4391983 | 4.50080056 | -2.1234066 | 0.03681735 | 0.76939636 | -3.6848375 | -2.0877837 |
| CPZ | 1.44657279 | -3.0705048 | 2.12182948 | 0.03683596 | 0.76939636 | -3.721766 | 2.08757754 |
| PTP4A2 | 0.191874 | 7.76673628 | 2.12031307 | 0.03688509 | 0.76939636 | -3.6799466 | 2.08703366 |
| TCEAL8 | 0.1771959 | 5.60555081 | 2.12247377 | 0.03690108 | 0.76939636 | -3.6821115 | 2.08685679 |
| LINC02551 | 1.23120859 | -4.1030615 | 2.11849804 | 0.03692654 | 0.76939636 | -3.6865442 | 2.08657534 |
| ALKBH2 | 0.20344666 | 3.92574793 | 2.12100384 | 0.03693857 | 0.76939636 | -3.6959792 | 2.08644233 |
| RPL22P19 | -1.4154929 | -5.543039 | -2.1196733 | 0.03698559 | 0.76939636 | -3.6817048 | -2.0859231 |
| HTR3A | 1.33980401 | -5.0064176 | 2.11964702 | 0.0369963 | 0.76939636 | -3.6767237 | 2.08580486 |
| MCMDC2 | -0.3911201 | -0.3013673 | -2.1203061 | 0.0370218 | 0.76939636 | -3.6810844 | -2.0855236 |
| C1RL | 1.03253138 | 4.07836378 | 2.12179596 | 0.03702838 | 0.76939636 | -3.6830439 | 2.085451 |
| SPHK1 | 0.84517373 | 2.61009484 | 2.12009717 | 0.03702972 | 0.76939636 | -3.6802048 | 2.08543622 |
| ATOH8 | 0.50156555 | 3.23084548 | 2.11974814 | 0.03703702 | 0.76939636 | -3.6693417 | 2.0853558 |
| FAM228A | 0.3651785 | 0.41567214 | 2.12189594 | 0.03711131 | 0.76939636 | -3.7065186 | 2.08453739 |
| GPR156 | 0.27318567 | 1.97816542 | 2.12050016 | 0.03711665 | 0.76939636 | -3.6963273 | 2.08447861 |
| MTCYBP35 | -1.2341639 | -6.4315129 | -2.118365 | 0.03712844 | 0.76939636 | -3.6899302 | -2.0843489 |
| LINC00323 | 1.22065953 | -3.0095638 | 2.11864165 | 0.03718512 | 0.76939636 | -3.6700447 | 2.08372576 |
| LRRC37A17P | 1.13969459 | -3.5900708 | 2.11586021 | 0.03724173 | 0.76939636 | -3.6873465 | 2.08310418 |
| LIMA1 | 0.25565893 | 5.65404862 | 2.11850242 | 0.03724907 | 0.76939636 | -3.6885617 | 2.08302362 |
| IMMP2L | 0.31665351 | 1.99604072 | 2.1179243 | 0.03726949 | 0.76939636 | -3.6800172 | 2.08279959 |
| LINC02069 | -1.5172332 | -5.4771532 | -2.1151978 | 0.03729659 | 0.76939636 | -3.6881313 | -2.0825025 |
| ZBTB42 | 0.51641161 | 2.39065935 | 2.11701374 | 0.03736001 | 0.76939636 | -3.6829131 | 2.08180797 |
| AGPAT2 | 0.45496351 | 3.61542745 | 2.11662264 | 0.03740052 | 0.76939636 | -3.6882516 | 2.08136485 |
| NPNT | -0.7466833 | 4.17927561 | -2.1149153 | 0.03743068 | 0.76939636 | -3.6925094 | -2.0810353 |
| TSPAN33 | -0.3851285 | 4.07818826 | -2.1162484 | 0.03743775 | 0.76939636 | -3.7006861 | -2.080958 |
| ZNF496-DT | 0.22724891 | 4.46670581 | 2.11604088 | 0.0374437 | 0.76939636 | -3.7010661 | 2.08089301 |
| NBAT1 | -0.5222891 | -1.0915835 | -2.1151369 | 0.03746975 | 0.76939636 | -3.6922345 | -2.0806086 |
| MMP17 | 0.51416572 | 6.46988106 | 2.11409011 | 0.03754993 | 0.76939636 | -3.7015143 | 2.07973404 |
| CCT8P1 | -0.4539134 | 0.08021745 | -2.1131008 | 0.03755433 | 0.76939636 | -3.6992713 | -2.0796861 |
| LRRC4 | -0.2748214 | 6.08448198 | -2.1138956 | 0.03757275 | 0.76939636 | -3.69312 | -2.0794855 |
| LYSMD1 | -0.1918864 | 4.29348928 | -2.113695 | 0.03765863 | 0.76939636 | -3.698935 | -2.0785511 |
| PROKR1 | 0.78756695 | 1.6736009 | 2.11211302 | 0.03766938 | 0.76939636 | -3.6955991 | 2.07843421 |
| ANOS1 | -0.8124527 | 5.6457145 | -2.1118332 | 0.03767132 | 0.76939636 | -3.6938986 | -2.0784131 |
| KDSR | 0.20691591 | 5.83768652 | 2.11286178 | 0.03772422 | 0.76939636 | -3.6987006 | 2.07783868 |
| PDE4DIPP1 | -0.938289 | -3.0239782 | -2.111581 | 0.03772818 | 0.76939636 | -3.711676 | -2.0777957 |
| FKBP9 | 0.34869573 | 7.29440252 | 2.1126151 | 0.03776111 | 0.76939636 | -3.6989452 | 2.07743841 |
| AKTIPP2 | -0.9401914 | -3.0423892 | -2.11106 | 0.03778183 | 0.76939636 | -3.6875097 | -2.0772138 |
| MYADM | 0.30779983 | 5.51439543 | 2.11085658 | 0.03786239 | 0.76939636 | -3.6998578 | 2.07634134 |
| PGAM1P5 | -1.3436697 | -3.9082753 | -2.1094003 | 0.03786378 | 0.76939636 | -3.7059626 | -2.0763264 |
| LINC02340 | 0.67074683 | 0.24210022 | 2.10893344 | 0.03792459 | 0.76939636 | -3.7031063 | 2.07566888 |
| LINC01823 | 1.28254278 | -4.2520025 | 2.10782578 | 0.03794456 | 0.76939636 | -3.7067136 | 2.07545315 |
| PTPN11P4 | -1.5936865 | -5.0457615 | -2.1081434 | 0.03803321 | 0.76939636 | -3.7021679 | -2.0744966 |
| ADRB1 | -0.9154755 | -0.4311831 | -2.1091387 | 0.0380505 | 0.76939636 | -3.7433597 | -2.0743103 |
| MTHFS | 0.3955968 | -0.5562201 | 2.10681897 | 0.03808519 | 0.76939636 | -3.7036831 | 2.07393665 |
| TMEM144 | 0.56126136 | 0.90281048 | 2.10840456 | 0.03816095 | 0.76939636 | -3.7005849 | 2.07312175 |
| ZNF586 | -0.3314068 | 3.50935713 | -2.1070926 | 0.03818351 | 0.76939636 | -3.7059756 | -2.0728793 |
| RPS3AP5 | -0.7556204 | -1.1874673 | -2.106267 | 0.03818708 | 0.76939636 | -3.6938756 | -2.072841 |
| NPLOC4 | 0.12505572 | 7.23089928 | 2.10480106 | 0.03834655 | 0.76939636 | -3.7058138 | 2.07113106 |
| MIR6514 | 1.1254353 | -4.7697863 | 2.10052977 | 0.03849553 | 0.76939636 | -3.6996834 | 2.06953908 |
| FECH | 0.22131389 | 5.36329012 | 2.10369323 | 0.03851788 | 0.76939636 | -3.71131 | 2.06930065 |
| NAGLU | 0.48188135 | 5.38030422 | 2.10400277 | 0.03852602 | 0.76939636 | -3.7054621 | 2.06921387 |
| HPS3 | 0.17939326 | 4.22228982 | 2.10316195 | 0.03855253 | 0.76939636 | -3.709032 | 2.06893136 |
| NTHL1 | 0.18349657 | 3.71491612 | 2.10271071 | 0.03859082 | 0.76939636 | -3.710249 | 2.0685235 |
| RBM4B | -0.179491 | 5.4370713 | -2.103057 | 0.03861608 | 0.76939636 | -3.7113971 | -2.0682546 |
| MIR5693 | 1.43094387 | -5.6444954 | 2.10029786 | 0.03861898 | 0.76939636 | -3.7233175 | 2.06822388 |
| ATPAF2 | -0.1314777 | 4.16536593 | -2.1020074 | 0.03866884 | 0.76939636 | -3.7145077 | -2.0676937 |
| TVP23B | 0.22196598 | 4.83966087 | 2.10104861 | 0.03875844 | 0.76939636 | -3.713754 | 2.06674237 |
| GPR142 | -1.3056914 | -6.1825079 | -2.098605 | 0.03884434 | 0.76939636 | -3.7101294 | -2.0658321 |
| TAS2R1 | -1.2852204 | -4.1560248 | -2.0987312 | 0.03886367 | 0.76939636 | -3.7094554 | -2.0656275 |
| SLC35C2 | 0.13075413 | 6.01171495 | 2.10018683 | 0.03887206 | 0.76939636 | -3.7170033 | 2.06553864 |
| RAB43 | 0.36600553 | 0.97190015 | 2.09980268 | 0.03888122 | 0.76939636 | -3.7148509 | 2.06544175 |
| ADAMTS9-AS2 | 0.78073097 | -0.4939541 | 2.09995763 | 0.03889189 | 0.76939636 | -3.6928479 | 2.06532896 |
| VIPAS39 | -0.1736267 | 4.82521939 | -2.0981955 | 0.03901275 | 0.76939636 | -3.7175094 | -2.0640524 |
| UBE2SP1 | -0.4538844 | -0.2887375 | -2.0968722 | 0.03904899 | 0.76939636 | -3.7214693 | -2.0636703 |
| PIGT | 0.28600183 | 6.8391455 | 2.09795227 | 0.03905436 | 0.76939636 | -3.7176733 | 2.06361362 |
| RPL9P2 | 1.19908304 | -3.8749551 | 2.09597352 | 0.03906802 | 0.76939636 | -3.7332125 | 2.06346974 |
| SNORD3A | -1.4710778 | -3.820685 | -2.094512 | 0.0391285 | 0.76939636 | -3.7163461 | -2.062833 |
| CSMD2 | -0.5235374 | 5.62408907 | -2.097086 | 0.03914767 | 0.76939636 | -3.7291518 | -2.0626314 |
| FAM174C | 0.26322118 | 4.35607579 | 2.0962202 | 0.03915268 | 0.76939636 | -3.7149853 | 2.06257862 |
| TRIM7-AS2 | -0.7080747 | -1.6298862 | -2.0985449 | 0.03919341 | 0.76939636 | -3.7122613 | -2.0621505 |
| MFSD5 | 0.23934077 | 4.84982138 | 2.09558828 | 0.03919909 | 0.76939636 | -3.7196298 | 2.06209082 |
| PDE4B | 0.37563117 | 6.96397539 | 2.09545482 | 0.03921106 | 0.76939636 | -3.7203647 | 2.06196506 |
| TENT5A | 0.6368204 | 4.93485323 | 2.0957262 | 0.03922856 | 0.76939636 | -3.7215783 | 2.06178131 |
| KLF6 | 0.26003797 | 6.3300606 | 2.09486321 | 0.03924753 | 0.76939636 | -3.7207537 | 2.06158215 |
| KIF13B | 0.28459322 | 5.13486604 | 2.09569202 | 0.03926395 | 0.76939636 | -3.7207759 | 2.06140994 |
| TMED3 | 0.29829403 | 6.23333653 | 2.09472761 | 0.03926531 | 0.76939636 | -3.721108 | 2.06139562 |
| UPF3AP3 | -1.5376971 | -4.1332524 | -2.0931857 | 0.03930017 | 0.76939636 | -3.7301231 | -2.06103 |
| CCDC33 | -0.8260903 | -1.721543 | -2.0944347 | 0.0393386 | 0.76939636 | -3.7125223 | -2.0606274 |
| PSMC1P5 | -1.4612126 | -5.8483798 | -2.0927888 | 0.03934615 | 0.76939636 | -3.7075807 | -2.0605483 |
| UFSP2 | 0.16025938 | 4.69467842 | 2.09496826 | 0.03934969 | 0.76939636 | -3.7218887 | 2.06051131 |
| RPL23AP81 | -0.9676009 | -3.2923746 | -2.0934 | 0.0393526 | 0.76939636 | -3.7309533 | -2.0604808 |
| SORT1 | 0.33082678 | 8.35220434 | 2.09454852 | 0.0393561 | 0.76939636 | -3.7210622 | 2.06044413 |
| FAM106A | -1.0086524 | -3.0596568 | -2.0933879 | 0.0393635 | 0.76939636 | -3.7064436 | -2.0603666 |
| ACKR1 | 0.58810124 | 4.23994172 | 2.09335372 | 0.0394025 | 0.76948527 | -3.730665 | 2.05995864 |
| MAPK6P5 | -1.5786062 | -5.2353979 | -2.0891702 | 0.03954289 | 0.76982112 | -3.7231345 | -2.0584924 |
| VN1R67P | -1.3277243 | -5.982166 | -2.0908505 | 0.03956849 | 0.76982112 | -3.7220699 | -2.0582255 |
| PCDHB19P | -1.2181073 | -3.8611914 | -2.0890231 | 0.03956863 | 0.76982112 | -3.7239162 | -2.058224 |
| RPSAP4 | -1.335526 | -6.1872209 | -2.0896258 | 0.03958076 | 0.76982112 | -3.7183991 | -2.0580976 |
| FAM201B | 0.54090183 | 0.11578851 | 2.09166092 | 0.03959198 | 0.76982112 | -3.7227371 | 2.05798075 |
| MEIS3P2 | 0.56198873 | 1.50935076 | 2.09097326 | 0.03969268 | 0.77048008 | -3.7251575 | 2.05693295 |
| SBNO2 | 0.44633127 | 5.3422898 | 2.09103815 | 0.03972046 | 0.77048008 | -3.7249235 | 2.05664421 |
| NDUFB4P8 | 1.27612863 | -4.6917739 | 2.08746822 | 0.03972933 | 0.77048008 | -3.7248239 | 2.05655206 |
| TNFRSF13C | 0.59267821 | 2.35924961 | 2.08930839 | 0.03984364 | 0.77202665 | -3.7590507 | 2.05536629 |
| AKNAD1 | -0.6916258 | -0.098243 | -2.0876572 | 0.0398789 | 0.77204027 | -3.7371827 | -2.0550011 |
| LMF2 | 0.21731709 | 6.80225058 | 2.08755236 | 0.03999855 | 0.77368622 | -3.7316837 | 2.05376389 |
| LRIG2-DT | -0.4495482 | -0.3558651 | -2.0865364 | 0.04004402 | 0.77374443 | -3.7154573 | -2.0532946 |
| YIPF5 | 0.17972983 | 5.9142327 | 2.08510111 | 0.04017382 | 0.77374443 | -3.7332226 | 2.05195725 |
| IRF1 | 0.63200317 | 2.55795529 | 2.08620586 | 0.0401782 | 0.77374443 | -3.7237514 | 2.0519122 |
| CTSC | 0.69889509 | 4.02353624 | 2.0851936 | 0.04018224 | 0.77374443 | -3.720704 | 2.05187064 |
| CYB5RL | 0.25452122 | 3.21601213 | 2.0843822 | 0.04023208 | 0.77374443 | -3.7311079 | 2.0513582 |
| LYPLA2 | 0.20782593 | 5.99753214 | 2.08438405 | 0.04023599 | 0.77374443 | -3.7338428 | 2.05131803 |
| POLH-AS1 | -0.3813531 | 1.0947744 | -2.0835446 | 0.04025273 | 0.77374443 | -3.7385717 | -2.051146 |
| SGSH | 0.33961597 | 5.49632099 | 2.08468971 | 0.04027863 | 0.77374443 | -3.7344056 | 2.05088009 |
| ALDH6A1 | -0.2978579 | 5.64116488 | -2.0835969 | 0.04033763 | 0.77378134 | -3.7313217 | -2.0502748 |
| GGT5 | 1.5595848 | 0.41119698 | 2.08406543 | 0.04034982 | 0.77378134 | -3.7090553 | 2.05014978 |
| RAD9B | 0.38065889 | 0.13972395 | 2.08367695 | 0.04047055 | 0.77456192 | -3.7275319 | 2.04891377 |
| ZNF426-DT | -0.3011332 | 1.07537527 | -2.0799775 | 0.04064789 | 0.77456192 | -3.7362283 | -2.0471039 |
| LY75 | 1.35172686 | -3.4539401 | 2.07887821 | 0.04065072 | 0.77456192 | -3.7589335 | 2.04707497 |
| PDGFRL | 0.78953331 | 1.07338075 | 2.081598 | 0.04067778 | 0.77456192 | -3.738693 | 2.04679941 |
| TSBP1 | 1.3211832 | -5.3980104 | 2.07790489 | 0.04068441 | 0.77456192 | -3.7328695 | 2.04673193 |
| CYBC1 | 0.2025331 | 6.38622252 | 2.07811614 | 0.04076464 | 0.77456192 | -3.7398229 | 2.045916 |
| NDUFB9 | 0.18366653 | 5.71398057 | 2.07693845 | 0.04082404 | 0.77456192 | -3.7333493 | 2.0453127 |
| CTDNEP1P1 | -0.6658977 | -1.7641686 | -2.0772239 | 0.04083791 | 0.77456192 | -3.7467737 | -2.045172 |
| ADRA1A | -0.8011533 | 2.9267159 | -2.0775717 | 0.04087846 | 0.77456192 | -3.7569173 | -2.0447606 |
| NTSR2 | -1.6637312 | -4.4217442 | -2.0765154 | 0.04093363 | 0.77456192 | -3.7411961 | -2.0442017 |
| INO80D-AS1 | 0.3536548 | 2.06976566 | 2.07716469 | 0.04098255 | 0.77456192 | -3.7437783 | 2.0437065 |
| ADAM3B | -1.2671735 | -6.1097664 | -2.0746149 | 0.0410195 | 0.77456192 | -3.7316545 | -2.0433329 |
| MFSD14B | 0.22297027 | 6.11067617 | 2.07641782 | 0.04106325 | 0.77456192 | -3.7455478 | 2.04289079 |
| NECTIN2 | 0.25543485 | 6.24760514 | 2.07619657 | 0.04108309 | 0.77456192 | -3.7453398 | 2.04269044 |
| GPR108 | 0.18062227 | 5.63569785 | 2.07549451 | 0.04117464 | 0.77456192 | -3.7470234 | 2.04176714 |
| TERF1P2 | -1.4220102 | -5.8828524 | -2.0715548 | 0.04120374 | 0.77456192 | -3.73395 | -2.041474 |
| GTF2IP13 | -1.1600666 | -4.8658461 | -2.0710774 | 0.0412773 | 0.77456192 | -3.7427533 | -2.0407338 |
| NPHP3-ACAD11 | -1.1969724 | -3.8343463 | -2.0722992 | 0.04134651 | 0.77456192 | -3.7514662 | -2.0400383 |
| RPL14P3 | -1.2036867 | -4.118588 | -2.0707346 | 0.04138606 | 0.77456192 | -3.75875 | -2.0396414 |
| LINC01703 | 0.59432514 | -0.3289713 | 2.07550042 | 0.04141792 | 0.77456192 | -3.7626047 | 2.03932185 |
| RASA3 | 0.31846957 | 6.76694721 | 2.07247337 | 0.0414232 | 0.77456192 | -3.7488475 | 2.03926889 |
| DHX37 | 0.16743175 | 5.12296304 | 2.0704462 | 0.04147239 | 0.77456192 | -3.7536596 | 2.03877594 |
| ESM1 | -1.7851996 | -4.5437 | -2.0699205 | 0.04160469 | 0.77456192 | -3.7481626 | -2.0374527 |
| BCL2L10 | 1.01395857 | -2.834881 | 2.06808657 | 0.04168059 | 0.77456192 | -3.7562988 | 2.03669529 |
| HMGB1P41 | -1.5195584 | -4.4205511 | -2.0677001 | 0.04172818 | 0.77456192 | -3.7422595 | -2.0362209 |
| OCEL1 | 0.25097049 | 3.61015088 | 2.06789047 | 0.04175681 | 0.77456192 | -3.7533871 | 2.03593571 |
| SHISA5 | 0.25003066 | 7.47035993 | 2.06836552 | 0.0418011 | 0.77456192 | -3.7535483 | 2.03549492 |
| TTC27 | 0.21059328 | 3.93889194 | 2.06719774 | 0.04188264 | 0.77456192 | -3.7535673 | 2.03468434 |
| NXPE3 | 0.22907418 | 5.89559754 | 2.06678553 | 0.04191717 | 0.77456192 | -3.7539741 | 2.03434153 |
| FCF1P8 | -1.3409833 | -6.1397792 | -2.064925 | 0.04193012 | 0.77456192 | -3.756079 | -2.034213 |
| RPL11P3 | -1.1678448 | -3.2442547 | -2.0701095 | 0.04195952 | 0.77456192 | -3.7593265 | -2.0339214 |
| NBPF1 | -0.2252455 | 5.93590271 | -2.0670113 | 0.04198058 | 0.77456192 | -3.7597105 | -2.0337126 |
| APEH | 0.14408994 | 6.78851582 | 2.06658073 | 0.04198736 | 0.77456192 | -3.7582985 | 2.03364537 |
| EARS2 | 0.13064188 | 5.49275405 | 2.0649504 | 0.04203418 | 0.77456192 | -3.7579148 | 2.03318157 |
| LINC01498 | -1.3125554 | -4.2522178 | -2.0628734 | 0.04205328 | 0.77456192 | -3.7629685 | -2.0329925 |
| LINC01460 | 1.26695213 | -3.1965498 | 2.0643877 | 0.04205404 | 0.77456192 | -3.7741625 | 2.03298495 |
| FMO3 | -1.0672113 | -6.0154981 | -2.0652313 | 0.04208105 | 0.77456192 | -3.7539869 | -2.0327177 |
| GABRQ | 0.88499602 | 4.74997713 | 2.06399251 | 0.04216052 | 0.77456192 | -3.7544572 | 2.03193219 |
| CPXM2 | 0.64871842 | 3.69103648 | 2.06435895 | 0.04219431 | 0.77456192 | -3.7668783 | 2.03159861 |
| FCER1G | 0.65046796 | -1.6452367 | 2.06446856 | 0.04224143 | 0.77456192 | -3.7720382 | 2.03113374 |
| ARHGEF17-AS1 | 0.57677569 | -1.1558188 | 2.06351651 | 0.04225889 | 0.77456192 | -3.7513739 | 2.03096159 |
| CALHM6 | -1.317287 | -3.5888801 | -2.0630819 | 0.04226844 | 0.77456192 | -3.7809643 | -2.0308675 |
| HIKESHIP2 | -1.4248768 | -5.9424647 | -2.0603331 | 0.04227897 | 0.77456192 | -3.7546895 | -2.0307637 |
| ENPP7P7 | -1.0087615 | -2.5268572 | -2.0622933 | 0.04230141 | 0.77456192 | -3.7616882 | -2.0305426 |
| CAMSAP1-DT | -0.2400485 | 2.40366436 | -2.0631186 | 0.04232696 | 0.77456192 | -3.7657826 | -2.0302911 |
| MPHOSPH8 | -0.1309883 | 6.23552607 | -2.0630587 | 0.04234451 | 0.77456192 | -3.7615647 | -2.0301184 |
| CDH15 | -1.1239661 | -1.4385084 | -2.0625134 | 0.04234547 | 0.77456192 | -3.7673618 | -2.0301089 |
| PDE3A | 0.53458225 | 6.79064876 | 2.06262242 | 0.04236892 | 0.77456192 | -3.7610845 | 2.02987818 |
| LINC00184 | 1.31125081 | -5.3248247 | 2.05909928 | 0.04241751 | 0.77456192 | -3.7559081 | 2.0294005 |
| STH | -1.0198687 | -3.256909 | -2.0615123 | 0.04242268 | 0.77456192 | -3.7684894 | -2.0293497 |
| KCNE1 | 0.60654317 | -0.1409009 | 2.06080995 | 0.0424263 | 0.77456192 | -3.7676012 | 2.02931421 |
| KRT81 | 1.33213411 | -3.3213233 | 2.0609875 | 0.0424317 | 0.77456192 | -3.7700193 | 2.02926111 |
| ACOT12 | 0.72149721 | -0.2510835 | 2.05892216 | 0.04259586 | 0.77456192 | -3.7746577 | 2.02765121 |
| SPDYA | 0.47548548 | -0.369529 | 2.05872796 | 0.04260294 | 0.77456192 | -3.7756493 | 2.02758189 |
| HOXA5 | -2.0079026 | -4.7732845 | -2.0571482 | 0.0426453 | 0.77456192 | -3.761842 | -2.0271674 |
| PDE4D | 0.41022356 | 7.24031511 | 2.05935384 | 0.04266663 | 0.77456192 | -3.7663394 | 2.02695881 |
| SH2D1A | -1.3467968 | -5.507607 | -2.0585238 | 0.04268339 | 0.77456192 | -3.7692416 | -2.0267949 |
| POMGNT1 | 0.16701462 | 7.20171416 | 2.05871151 | 0.04268704 | 0.77456192 | -3.7667056 | 2.02675929 |
| OVCH1 | -0.5469962 | -0.6797817 | -2.0579103 | 0.04276462 | 0.77456192 | -3.7844142 | -2.0260016 |
| FAM181A | 0.35263752 | 4.08254364 | 2.05870358 | 0.04276963 | 0.77456192 | -3.7523156 | 2.02595271 |
| TMEM250 | 0.22670063 | 5.8357473 | 2.05681612 | 0.0427889 | 0.77456192 | -3.7653423 | 2.02576471 |
| ADGRE2 | 1.52012338 | -4.4812409 | 2.05634323 | 0.04284332 | 0.77456192 | -3.7790792 | 2.02523418 |
| FOXD2 | -1.2563996 | -5.7292897 | -2.0558946 | 0.04295276 | 0.77456192 | -3.7661411 | -2.0241691 |
| LIPT2 | 0.25699454 | 1.47047148 | 2.05737594 | 0.04295762 | 0.77456192 | -3.7789501 | 2.02412176 |
| APOL1 | 1.25516428 | -0.8273233 | 2.05744695 | 0.04296 | 0.77456192 | -3.7442426 | 2.02409864 |
| IFI6 | 0.73892061 | 5.61938571 | 2.05644819 | 0.04300593 | 0.77456192 | -3.7650299 | 2.0236524 |
| RBP1 | 0.475901 | 5.25687993 | 2.05652188 | 0.04300948 | 0.77456192 | -3.7698423 | 2.02361792 |
| HPS1 | 0.44943834 | 3.56745858 | 2.0566834 | 0.0430119 | 0.77456192 | -3.776352 | 2.02359442 |
| PRDM11 | -0.2371432 | 5.02533273 | -2.055004 | 0.04308521 | 0.77456192 | -3.7775398 | -2.022883 |
| CCDC26 | 1.16827032 | -4.8094253 | 2.05283175 | 0.04312723 | 0.77456192 | -3.7586367 | 2.02247575 |
| HCCS | 0.15171443 | 3.75398307 | 2.05366586 | 0.04315958 | 0.77456192 | -3.7741824 | 2.0221624 |
| SHANK1 | -0.4312574 | 7.02153951 | -2.0541532 | 0.04322767 | 0.77456192 | -3.7769633 | -2.0215035 |
| ADGRF5P1 | -0.9710321 | -3.2426248 | -2.0534513 | 0.04327339 | 0.77456192 | -3.7904742 | -2.0210616 |
| ZNF837 | 0.28001314 | 3.05541503 | 2.05299217 | 0.04328298 | 0.77456192 | -3.7805872 | 2.02096892 |
| DNAJB11 | 0.16153017 | 5.58415403 | 2.05369837 | 0.04328925 | 0.77456192 | -3.7769559 | 2.02090838 |
| M1AP | 1.15370451 | -1.4027472 | 2.05268926 | 0.04331152 | 0.77456192 | -3.7712226 | 2.02069334 |
| ADAM1B | -0.475594 | -0.6802445 | -2.052061 | 0.0433776 | 0.77456192 | -3.7760023 | -2.0200558 |
| MFAP1P1 | -1.4203047 | -4.3926525 | -2.0522023 | 0.04340331 | 0.77456192 | -3.7781578 | -2.019808 |
| LINC00612 | 1.03655878 | -1.4623499 | 2.05145894 | 0.04340491 | 0.77456192 | -3.7855323 | 2.01979252 |
| LINC00513 | 0.90498497 | -2.6889628 | 2.05106815 | 0.04344294 | 0.77456192 | -3.7752544 | 2.01942625 |
| GALNT7-DT | -0.6341324 | -0.8050267 | -2.0501693 | 0.04344435 | 0.77456192 | -3.7773384 | -2.0194126 |
| NARF-AS2 | 0.40561869 | 1.23017459 | 2.05044573 | 0.0435656 | 0.77456192 | -3.7682717 | 2.01824643 |
| PRDM2 | -0.176846 | 6.59241653 | -2.0505306 | 0.0435668 | 0.77456192 | -3.781194 | -2.0182349 |
| GAPDHP33 | -1.7559392 | -4.9042352 | -2.0487893 | 0.04359492 | 0.77456192 | -3.8011582 | -2.0179649 |
| SPOCK2 | 0.47621053 | 9.16629992 | 2.04999099 | 0.04361098 | 0.77456192 | -3.780156 | 2.01781067 |
| ERGIC3 | 0.22031959 | 7.64151896 | 2.0499371 | 0.04362711 | 0.77456192 | -3.7796638 | 2.01765592 |
| MGAT3-AS1 | -0.9674144 | -2.0403432 | -2.0490666 | 0.04364862 | 0.77456192 | -3.7870107 | -2.0174496 |
| QARS1 | 0.20197312 | 7.27609944 | 2.04963895 | 0.04367985 | 0.77456192 | -3.778687 | 2.0171501 |
| FLT1 | -1.4611722 | -3.8743491 | -2.0485931 | 0.0437304 | 0.77456192 | -3.8023007 | -2.0166658 |
| PIRT | -0.636354 | 0.69568395 | -2.0488334 | 0.04375289 | 0.77456192 | -3.7604905 | -2.0164505 |
| C2orf72 | -0.4971657 | 5.63307663 | -2.0481985 | 0.04376807 | 0.77456192 | -3.7783051 | -2.0163052 |
| MUL1 | 0.19674208 | 5.45801589 | 2.04807613 | 0.04377838 | 0.77456192 | -3.7821473 | 2.01620655 |
| TMEM230P2 | -1.5040319 | -5.0282392 | -2.0448198 | 0.04381041 | 0.77456192 | -3.7794974 | -2.0159003 |
| ARSD | 0.26915647 | 5.50666054 | 2.04844604 | 0.04381081 | 0.77456192 | -3.7818293 | 2.01589639 |
| ADGRL4 | 1.03358769 | -0.7614731 | 2.0477135 | 0.04384112 | 0.77456192 | -3.758872 | 2.01560667 |
| KCTD21-AS1 | 0.38071807 | 1.06258159 | 2.04703767 | 0.04390265 | 0.77456192 | -3.7810426 | 2.01501908 |
| ZNF544 | -0.2165974 | 3.6606973 | -2.0458779 | 0.04390768 | 0.77456192 | -3.7808772 | -2.014971 |
| SNRPA1P1 | -1.1193004 | -3.4357747 | -2.045064 | 0.0439442 | 0.77456192 | -3.7874431 | -2.0146227 |
| MXRA5 | 1.04238251 | 3.28534311 | 2.04589616 | 0.04394474 | 0.77456192 | -3.7805269 | 2.01461749 |
| KDELR2 | 0.18260408 | 7.17684607 | 2.04606078 | 0.0440002 | 0.77456192 | -3.7842891 | 2.01408895 |
| ARHGAP33 | -0.3077812 | 7.88858158 | -2.0463491 | 0.04401064 | 0.77456192 | -3.786335 | -2.0139895 |
| TM2D2 | 0.16481224 | 5.58527135 | 2.04546016 | 0.04402124 | 0.77456192 | -3.7820913 | 2.01388851 |
| PAUPAR | 0.52534253 | -0.8023929 | 2.04571092 | 0.04417174 | 0.77456192 | -3.7817824 | 2.01245745 |
| DNAH11 | -0.6063171 | 2.37277048 | -2.0448004 | 0.04419019 | 0.77456192 | -3.7815577 | -2.0122822 |
| ZFHX4-AS1 | -0.6250798 | 1.83334953 | -2.0445346 | 0.04420705 | 0.77456192 | -3.791898 | -2.0121223 |
| GRIK5 | -0.3792895 | 6.9357503 | -2.0437977 | 0.04423166 | 0.77456192 | -3.7883624 | -2.0118888 |
| CCL20 | 1.22762772 | -6.4071294 | 2.04281672 | 0.04423565 | 0.77456192 | -3.7699641 | 2.01185094 |
| INHBA | 0.70797187 | 2.24038929 | 2.04320495 | 0.04425229 | 0.77456192 | -3.8118352 | 2.01169318 |
| NSA2P1 | -1.4808367 | -5.1167015 | -2.045267 | 0.04425388 | 0.77456192 | -3.7774153 | -2.0116781 |
| LINC01433 | -1.3506573 | -4.1118443 | -2.0415961 | 0.0442613 | 0.77456192 | -3.7904118 | -2.0116078 |
| UBXN6 | 0.18847921 | 7.09620813 | 2.04316713 | 0.04427546 | 0.77456192 | -3.7819624 | 2.0114736 |
| FILIP1L | 0.50450169 | 2.4165662 | 2.04284205 | 0.04433297 | 0.77456192 | -3.7737116 | 2.01092889 |
| ARHGAP1 | 0.15955917 | 6.96990065 | 2.04241373 | 0.04437827 | 0.77456192 | -3.7887877 | 2.01050027 |
| TBX15 | -0.5085815 | 0.57659426 | -2.0428182 | 0.04440435 | 0.77456192 | -3.7769493 | -2.0102536 |
| SDF4 | 0.2160652 | 7.5397957 | 2.04226 | 0.04441171 | 0.77456192 | -3.7893857 | 2.01018408 |
| TMEM216 | 0.29173321 | 3.25228122 | 2.04160506 | 0.04441628 | 0.77456192 | -3.7934809 | 2.01014085 |
| LAMA3 | -0.5749891 | 1.29627889 | -2.0421003 | 0.04444691 | 0.77456192 | -3.8047561 | -2.0098515 |
| SPA17P1 | -1.1462565 | -6.1035578 | -2.0399512 | 0.04451469 | 0.77488388 | -3.7916681 | -2.0092117 |
| CDCP1 | 1.23646958 | -0.6049672 | 2.04033105 | 0.04463941 | 0.77488388 | -3.7761492 | 2.00803657 |
| ZNF501 | -0.2528106 | 3.56558304 | -2.0389471 | 0.04469219 | 0.77488388 | -3.796435 | -2.00754 |
| LIF-AS2 | 1.53783122 | -3.8621017 | 2.03714998 | 0.04480155 | 0.77488388 | -3.7942454 | 2.00651292 |
| AIDAP1 | -1.4114002 | -5.3348183 | -2.0354487 | 0.0448315 | 0.77488388 | -3.7900057 | -2.006232 |
| RBBP5 | -0.1801984 | 4.95515412 | -2.0369914 | 0.04486916 | 0.77488388 | -3.7989839 | -2.005879 |
| PLA2G1B | -0.9952981 | -2.8418353 | -2.0353244 | 0.04487486 | 0.77488388 | -3.800776 | -2.0058256 |
| RHOBTB1 | 0.32573444 | 5.30510612 | 2.03714975 | 0.04489323 | 0.77488388 | -3.7952391 | 2.00565346 |
| TESC | 0.76600277 | 4.80767176 | 2.03694116 | 0.04491762 | 0.77488388 | -3.7931315 | 2.00542506 |
| RPS3AP40 | -1.326672 | -5.6256661 | -2.0361065 | 0.04496946 | 0.77488388 | -3.7972073 | -2.0049401 |
| LDHAP7 | -1.1143221 | -6.4361848 | -2.0355319 | 0.04500096 | 0.77488388 | -3.7968675 | -2.0046455 |
| OLFM2 | -0.3416355 | 7.63354984 | -2.0359354 | 0.0450037 | 0.77488388 | -3.7967687 | -2.0046199 |
| RNU6-1099P | 0.97909189 | -3.1257613 | 2.03428505 | 0.04501928 | 0.77488388 | -3.7946626 | 2.00447432 |
| NOMO1 | 0.24875834 | 6.83535096 | 2.03611535 | 0.04503398 | 0.77488388 | -3.7974946 | 2.00433697 |
| PFDN1P1 | -1.3145874 | -5.9176898 | -2.0348544 | 0.04503894 | 0.77488388 | -3.7927144 | -2.0042906 |
| RN7SL614P | 1.38673919 | -5.3729932 | 2.03372087 | 0.04505373 | 0.77488388 | -3.7932288 | 2.00415246 |
| STOML2 | 0.19292666 | 6.08603319 | 2.03510196 | 0.04508944 | 0.77488388 | -3.7954877 | 2.00381915 |
| CPPED1 | 0.40335543 | 3.62117004 | 2.03507447 | 0.0450897 | 0.77488388 | -3.7937832 | 2.00381667 |
| TEX54 | 1.29882088 | -4.8794317 | 2.0332674 | 0.04513041 | 0.7749381 | -3.7841737 | 2.00343693 |
| EREG | 1.46848388 | -3.5771167 | 2.03296646 | 0.04521681 | 0.7749381 | -3.8094897 | 2.00263196 |
| CRLF1 | -1.1063921 | 3.39564868 | -2.0334027 | 0.04531725 | 0.7749381 | -3.7838447 | -2.0016977 |
| FOSB | -1.1140238 | 1.02100104 | -2.0324427 | 0.04535484 | 0.7749381 | -3.8097398 | -2.0013486 |
| HLA-DRB5 | 1.46555235 | -3.3725919 | 2.03243063 | 0.04539334 | 0.7749381 | -3.79076 | 2.00099119 |
| HEXB | 0.42637291 | 5.37227715 | 2.03270178 | 0.04540241 | 0.7749381 | -3.7981543 | 2.00090699 |
| TPT1P4 | 1.0134141 | -2.3494395 | 2.03108546 | 0.0454505 | 0.7749381 | -3.807581 | 2.00046105 |
| BNIP3P4 | -1.171619 | -5.7148899 | -2.0307549 | 0.04545585 | 0.7749381 | -3.8073021 | -2.0004115 |
| TCHH | -0.5910187 | 0.97138815 | -2.0318369 | 0.04545806 | 0.7749381 | -3.8252608 | -2.000391 |
| PARS2 | -0.2200852 | 2.41734337 | -2.0311061 | 0.04550248 | 0.7749381 | -3.8047566 | -1.9999795 |
| C6orf132 | 1.14756842 | -3.2511664 | 2.02985177 | 0.0455172 | 0.7749381 | -3.8029316 | 1.99984318 |
| SURF1 | 0.24210008 | 4.89169567 | 2.03130049 | 0.04553223 | 0.7749381 | -3.8001221 | 1.99970402 |
| ELAVL3 | -0.3009641 | 8.40181385 | -2.0313766 | 0.04554379 | 0.7749381 | -3.8049739 | -1.9995971 |
| GGTA1 | -1.0800309 | -2.8946269 | -2.0298196 | 0.04559518 | 0.77510709 | -3.7972195 | -1.9991218 |
| ZSWIM5 | -0.297485 | 5.1808852 | -2.0300507 | 0.04567192 | 0.77510709 | -3.8107029 | -1.9984129 |
| ALG1L6P | 0.45799892 | 1.80496916 | 2.02884109 | 0.04572514 | 0.77510709 | -3.797409 | 1.99792181 |
| ADAM20 | -1.2563946 | -2.8211505 | -2.0264213 | 0.0457255 | 0.77510709 | -3.8174432 | -1.9979185 |
| BMP6 | -0.7326372 | 0.81852501 | -2.0290604 | 0.04575397 | 0.77510709 | -3.7870761 | -1.997656 |
| LAMP3 | 0.87701481 | -0.5558909 | 2.0279129 | 0.04576188 | 0.77510709 | -3.7938803 | 1.99758303 |
| TSSK6 | 0.26137607 | 1.71263804 | 2.02744359 | 0.04580262 | 0.77520936 | -3.8055781 | 1.99720774 |
| ASXL3-DT | -1.2310484 | -5.1654218 | -2.0272319 | 0.04584291 | 0.77530397 | -3.8101611 | -1.9968368 |
| PTGES3P1 | 0.70409666 | -0.6158399 | 2.02555344 | 0.046038 | 0.77645074 | -3.7970951 | 1.99504475 |
| PSME1 | 0.26839719 | 5.54618081 | 2.0263358 | 0.04605752 | 0.77645074 | -3.8063221 | 1.99486582 |
| LINC02986 | 0.47007732 | 0.38328701 | 2.02739345 | 0.04606407 | 0.77645074 | -3.8121038 | 1.99480572 |
| PLAAT4 | 0.67172058 | 2.07879982 | 2.02639861 | 0.04607392 | 0.77645074 | -3.7937136 | 1.99471548 |
| RPS3P2 | -1.3482074 | -4.4501393 | -2.0240998 | 0.04612137 | 0.77645074 | -3.819739 | -1.9942809 |
| RAB28P5 | -0.4820411 | -0.9720005 | -2.0227537 | 0.04613805 | 0.77645074 | -3.8016735 | -1.9941281 |
| RFTN1 | 0.3248957 | 5.34434452 | 2.02404181 | 0.04626748 | 0.77645074 | -3.80957 | 1.99294495 |
| NLRC4 | 0.70050273 | -2.1810029 | 2.02078774 | 0.04631899 | 0.77645074 | -3.8149046 | 1.99247478 |
| RPL5P12 | -1.4172877 | -5.2792715 | -2.0197217 | 0.04640363 | 0.77645074 | -3.8046639 | -1.9917032 |
| PLEKHA4 | 0.75198405 | 4.61187274 | 2.02265318 | 0.04645132 | 0.77645074 | -3.8045631 | 1.99126906 |
| ATP5MGP1 | 1.28478697 | -4.6639015 | 2.0198357 | 0.04657923 | 0.77645074 | -3.8194014 | 1.99010626 |
| RPL23AP3 | -1.4315823 | -5.1446197 | -2.0180481 | 0.04660049 | 0.77645074 | -3.8105308 | -1.9899133 |
| RER1 | 0.14158034 | 6.38352295 | 2.0211456 | 0.04660549 | 0.77645074 | -3.816643 | 1.98986789 |
| CHRM4 | -0.6205161 | 2.19848415 | -2.0198344 | 0.0466116 | 0.77645074 | -3.8147786 | -1.9898124 |
| CDHR3 | 0.45521837 | 3.68730641 | 2.01940423 | 0.04661448 | 0.77645074 | -3.8154177 | 1.98978629 |
| LGALS8 | 0.32192648 | 4.9650394 | 2.02090136 | 0.04662723 | 0.77645074 | -3.8216299 | 1.98967066 |
| LINC01981 | 1.45892938 | -5.6270379 | 2.01902478 | 0.04664187 | 0.77645074 | -3.8155862 | 1.9895378 |
| PPM1L | -0.3654828 | 6.86141309 | -2.0211121 | 0.0467098 | 0.77645074 | -3.8142761 | -1.9889221 |
| DLK2 | -0.2454943 | 3.12217563 | -2.018933 | 0.04684094 | 0.77645074 | -3.8289548 | -1.9877356 |
| NHLRC1 | 0.42872616 | 1.01805776 | 2.01784506 | 0.0468735 | 0.77645074 | -3.8181969 | 1.98744148 |
| MTHFD1L | 0.21776822 | 5.45454627 | 2.01691498 | 0.0468847 | 0.77645074 | -3.8208672 | 1.98734025 |
| NOC3L | 0.20824829 | 4.50452009 | 2.01825857 | 0.04689053 | 0.77645074 | -3.8238963 | 1.98728769 |
| SLC35E3 | 0.21149474 | 5.73208558 | 2.01780279 | 0.04690243 | 0.77645074 | -3.8185928 | 1.98718025 |
| HSPD1P12 | -1.4800504 | -4.9312175 | -2.0181199 | 0.04691606 | 0.77645074 | -3.8157544 | -1.9870572 |
| DNAH10 | -0.2821166 | 4.26598154 | -2.0175022 | 0.0469518 | 0.77645074 | -3.8303143 | -1.9867347 |
| GSTZ1 | 0.28098757 | 4.33974012 | 2.01741156 | 0.04703321 | 0.77645074 | -3.8260506 | 1.98600106 |
| GLIDR | 0.18075893 | 2.65583954 | 2.0167901 | 0.04704511 | 0.77645074 | -3.8264007 | 1.98589385 |
| ARFIP2 | 0.11071884 | 6.26006252 | 2.01658107 | 0.04705763 | 0.77645074 | -3.8211559 | 1.9857811 |
| KDR | 1.14518162 | -0.2217092 | 2.01530297 | 0.04716902 | 0.77645074 | -3.8081007 | 1.98477944 |
| CLINT1 | 0.11038722 | 6.26650181 | 2.01561567 | 0.04717387 | 0.77645074 | -3.8234159 | 1.98473585 |
| IFNA5 | 1.21198375 | -6.1427194 | 2.01366535 | 0.04719851 | 0.77645074 | -3.8231697 | 1.98451456 |
| ADGRV1 | -0.6350275 | 6.67707892 | -2.013982 | 0.04728326 | 0.77645074 | -3.8243578 | -1.9837541 |
| PKM | 0.27497408 | 9.59237943 | 2.0136042 | 0.04729387 | 0.77645074 | -3.8227289 | 1.98365894 |
| P4HB | 0.21350892 | 7.9952031 | 2.01461391 | 0.04730303 | 0.77645074 | -3.8243578 | 1.98357686 |
| ADAM9 | 0.32709094 | 7.63536289 | 2.01441375 | 0.04733138 | 0.77645074 | -3.8233824 | 1.98332285 |
| PHF10P1 | -1.2320756 | -5.1148365 | -2.0117541 | 0.04733772 | 0.77645074 | -3.8165843 | -1.983266 |
| HOXD4 | -1.4678995 | -5.3849514 | -2.0137313 | 0.04735268 | 0.77645074 | -3.8174489 | -1.9831321 |
| TNFAIP1 | 0.15576259 | 6.87307824 | 2.01278184 | 0.04737236 | 0.77645074 | -3.8203564 | 1.9829559 |
| MARCHF3 | -0.4347901 | 3.08184651 | -2.0123713 | 0.04739391 | 0.77645074 | -3.8276132 | -1.982763 |
| APOBEC3G | 1.24231039 | -0.9389682 | 2.01353043 | 0.04741922 | 0.77645074 | -3.8073373 | 1.98253654 |
| BCRP9 | 1.09035859 | -6.7016085 | 2.01188009 | 0.04745168 | 0.77645074 | -3.8230446 | 1.98224632 |
| KIF19 | -0.7233439 | 0.1503468 | -2.012592 | 0.04747491 | 0.77645074 | -3.8351185 | -1.9820387 |
| MACROH2A2 | -0.236952 | 6.36943998 | -2.0128217 | 0.04749462 | 0.77645074 | -3.8285793 | -1.9818626 |
| LINC01864 | -1.1568628 | -1.152685 | -2.0118807 | 0.04749991 | 0.77645074 | -3.8567021 | -1.9818154 |
| CCT6P3 | 0.69423064 | -2.5755087 | 2.00968925 | 0.04750156 | 0.77645074 | -3.8186772 | 1.98180059 |
| ZNF225-AS1 | -0.3142441 | 1.2231151 | -2.0121494 | 0.04753791 | 0.77645074 | -3.8340233 | -1.981476 |
| LINC01356 | -0.7731295 | -2.7791748 | -2.011069 | 0.04757288 | 0.77645074 | -3.8308249 | -1.981164 |
| PRR36 | -0.2605973 | 6.89328313 | -2.0120489 | 0.04757894 | 0.77645074 | -3.8293269 | -1.98111 |
| LHX6 | -1.7335004 | -2.1525796 | -2.0099289 | 0.04764718 | 0.7769969 | -3.8809646 | -1.9805017 |
| LINC02485 | -1.121035 | -6.6484653 | -2.0092818 | 0.04769244 | 0.77714768 | -3.8200483 | -1.9800987 |
| CA2 | 0.45427461 | 4.32497263 | 2.01039047 | 0.04774198 | 0.77714768 | -3.8264585 | 1.97965792 |
| TRAM2-AS1 | 0.16756228 | 4.35847578 | 2.01019637 | 0.0477847 | 0.77714768 | -3.8301036 | 1.97927815 |
| AMOT | -0.3598133 | 5.84518905 | -2.0095985 | 0.04780809 | 0.77714768 | -3.8296594 | -1.9790704 |
| SCD5 | -0.2963691 | 8.49377678 | -2.0096515 | 0.04783036 | 0.77714768 | -3.8303941 | -1.9788725 |
| RPS5P3 | 1.48131322 | -6.0643216 | 2.00688234 | 0.04792014 | 0.77730132 | -3.8332627 | 1.97807597 |
| APAF1 | -0.1959275 | 5.53329667 | -2.0089003 | 0.04793611 | 0.77730132 | -3.8336417 | -1.9779344 |
| MFSD3 | 0.22126456 | 4.72853275 | 2.00768706 | 0.04794419 | 0.77730132 | -3.8326442 | 1.97786274 |
| DTHD1 | -0.5451628 | 1.83209375 | -2.0082508 | 0.04801856 | 0.77794248 | -3.8208855 | -1.9772041 |
| DIO3 | 0.96209411 | 5.90705122 | 2.00600578 | 0.04818062 | 0.77998613 | -3.8459345 | 1.97577183 |
| TAPBPL | 0.69867169 | 1.54866144 | 2.00599281 | 0.04825516 | 0.77998613 | -3.8349917 | 1.97511447 |
| KLHL34 | -0.5534087 | 0.31327838 | -2.0046996 | 0.0483179 | 0.77998613 | -3.8546889 | -1.9745618 |
| SHC1 | 0.55552031 | 5.59383625 | 2.00507329 | 0.04831855 | 0.77998613 | -3.833004 | 1.97455603 |
| RNU6ATAC18P | -1.4161941 | -5.639824 | -2.0022083 | 0.04831927 | 0.77998613 | -3.8371335 | -1.9745497 |
| ARID3B | -0.2826895 | 3.96123558 | -2.0042988 | 0.0483587 | 0.78005903 | -3.8458373 | -1.9742027 |
| PRKCA | -0.4074031 | 6.75237822 | -2.0032993 | 0.04844336 | 0.78079701 | -3.8384335 | -1.9734584 |
| SNORA73B | 0.95522727 | -3.1430173 | 2.00158734 | 0.04847435 | 0.78079701 | -3.8349877 | 1.97318622 |
| MAEA | 0.0912237 | 6.45616404 | 2.00278255 | 0.04854511 | 0.7813734 | -3.8394288 | 1.97256531 |
| CYB5R2 | 0.69381048 | -0.6293002 | 2.00040976 | 0.04867075 | 0.78157106 | -3.8289707 | 1.97146464 |
| DCAF5 | -0.0843511 | 7.07463971 | -1.999948 | 0.04881099 | 0.78157106 | -3.8417312 | -1.9702389 |
| STON1-GTF2A1L | 1.1622876 | -5.386914 | 1.99871425 | 0.04887231 | 0.78157106 | -3.8323819 | 1.96970396 |
| CRYZL2P | 0.81322095 | 0.7908473 | 2.00038495 | 0.04888895 | 0.78157106 | -3.8388686 | 1.96955889 |
| LINC01891 | 1.28706412 | -5.4689695 | 1.99835789 | 0.04893235 | 0.78157106 | -3.8396919 | 1.96918065 |
| LAPTM4A | 0.27155285 | 7.16114119 | 1.99939533 | 0.04894849 | 0.78157106 | -3.8429444 | 1.96904005 |
| AGTR2 | -1.3679271 | -5.6109298 | -1.9979725 | 0.04897848 | 0.78157106 | -3.8402209 | -1.968779 |
| RHBDD2 | 0.2261452 | 8.08014285 | 1.99837369 | 0.04903865 | 0.78157106 | -3.8448553 | 1.9682555 |
| MICAL3 | -0.283544 | 6.92982194 | -1.998231 | 0.04908225 | 0.78157106 | -3.8471865 | -1.9678765 |
| PRXL2C | 0.28696052 | 3.05207966 | 1.99756394 | 0.04916143 | 0.78157106 | -3.8407011 | 1.96718899 |
| GNB2 | 0.20231171 | 8.39382347 | 1.99657212 | 0.04916266 | 0.78157106 | -3.8431934 | 1.96717826 |
| UNC79 | -0.3628492 | 5.86991347 | -1.9974288 | 0.04916745 | 0.78157106 | -3.8484391 | -1.9671368 |
| EGR1 | -0.9986272 | 5.17212162 | -1.996794 | 0.04916881 | 0.78157106 | -3.8418487 | -1.9671249 |
| LINC02010 | 1.29946954 | -5.2392557 | 1.99558307 | 0.04934675 | 0.78157106 | -3.8508281 | 1.96558345 |
| DUBR | 0.25385697 | 5.09667445 | 1.99485723 | 0.04935694 | 0.78157106 | -3.8488721 | 1.96549533 |
| TRAPPC3 | 0.16343186 | 5.63987339 | 1.99530236 | 0.04941791 | 0.78157106 | -3.8439332 | 1.96496826 |
| GHET1 | -0.53984 | 0.28656223 | -1.9948533 | 0.04945217 | 0.78157106 | -3.8552188 | -1.9646723 |
| CICP3 | -0.9454436 | -2.3183281 | -1.9923327 | 0.04954069 | 0.78157106 | -3.8569851 | -1.9639087 |
| DHRS9 | 1.20822415 | -5.5480995 | 1.99291454 | 0.04954179 | 0.78157106 | -3.8404796 | 1.96389913 |
| SNRPB | 0.26387524 | 5.68177114 | 1.99281698 | 0.04957819 | 0.78157106 | -3.8493958 | 1.96358541 |
| ARRDC3-AS1 | 0.40783491 | 0.71663665 | 1.99380335 | 0.04958834 | 0.78157106 | -3.8557296 | 1.963498 |
| MFSD1 | 0.19696258 | 5.51799476 | 1.9934175 | 0.04959956 | 0.78157106 | -3.8501773 | 1.96340136 |
| ACVR2B | -0.2754027 | 5.90526219 | -1.9931637 | 0.04966792 | 0.78157106 | -3.8538251 | -1.9628129 |
| ZPR1P1 | -1.3188959 | -5.2072865 | -1.9913386 | 0.04970748 | 0.78157106 | -3.8608911 | -1.9624727 |
| MT1G | -1.2730306 | -4.8936726 | -1.9921866 | 0.04970966 | 0.78157106 | -3.8417439 | -1.9624539 |
| PUS7 | 0.20783623 | 3.86723467 | 1.99166944 | 0.04972618 | 0.78157106 | -3.8590555 | 1.96231196 |
| ALX1 | 1.03356368 | -2.7675864 | 1.99139377 | 0.04978898 | 0.78157106 | -3.8426718 | 1.96177248 |
| SMN1 | 0.19894821 | 3.69333871 | 1.99055885 | 0.04979853 | 0.78157106 | -3.8485574 | 1.96169051 |
| SLC26A9 | -1.3171888 | -3.262789 | -1.9884708 | 0.04981526 | 0.78157106 | -3.8485862 | -1.9615469 |
| SLC10A6 | 1.61389729 | -4.1876553 | 1.99046788 | 0.04983205 | 0.78157106 | -3.8531553 | 1.9614028 |
| IQSEC2 | -0.1912565 | 5.91843767 | -1.9898842 | 0.0499464 | 0.78157106 | -3.8523847 | -1.9604227 |

**Supplementary Table S10:** Differentially Expressed Genes after LSD at 24 Hours

| **Gene Name** | **logFC** | **AveExpr** | **t** | **P.Value** | **adj.P.Val** | **B** | **z.std** |
| --- | --- | --- | --- | --- | --- | --- | --- |
| C16orf96 | 0.74535268 | 0.71973556 | 4.74145531 | 9.206E-06 | 0.20567098 | 2.39786045 | 4.43502903 |
| C3 | 3.70521755 | 0.78233119 | 4.33435743 | 4.2881E-05 | 0.38456392 | 1.53237975 | 4.09138392 |
| HPAT5 | 5.12691522 | -0.9122371 | 4.07584254 | 0.00010317 | 0.38456392 | 0.52006267 | 3.88301612 |
| ALPK2 | 2.46547434 | 0.63086661 | 4.03244693 | 0.00012384 | 0.38456392 | 0.61937925 | 3.83839896 |
| ETV7 | 2.0311612 | -1.646434 | 4.03354142 | 0.00012402 | 0.38456392 | 0.45311508 | 3.83804268 |
| MCTP2 | 2.76133174 | 1.03966057 | 4.02418058 | 0.00012536 | 0.38456392 | 0.54903263 | 3.83540918 |
| HTR3B | 2.38084125 | -2.3470379 | 3.99174583 | 0.00014297 | 0.38456392 | 0.2919659 | 3.80297003 |
| LPO | 1.85805072 | -3.2106977 | 3.92831879 | 0.00017326 | 0.38456392 | 0.26168851 | 3.75511887 |
| KCNJ16 | -1.730963 | 0.87575226 | -3.8919974 | 0.00020363 | 0.38456392 | 0.13879053 | -3.714465 |
| GBP1 | 2.16825698 | 1.62464706 | 3.88298181 | 0.00021258 | 0.38456392 | 0.14664109 | 3.70358359 |
| HOXD8 | -2.3063165 | -4.1830075 | -3.8471716 | 0.00023741 | 0.38456392 | 0.01069988 | -3.6754714 |
| GNGT2 | -2.2467347 | -5.5840751 | -3.8121146 | 0.0002614 | 0.38456392 | -0.0713647 | -3.6508239 |
| STAP2 | 1.13397555 | 1.49983783 | 3.79968212 | 0.00027861 | 0.38456392 | -0.0910334 | 3.63441549 |
| HTATIP2 | 1.26971445 | 0.49475154 | 3.80050365 | 0.00027961 | 0.38456392 | -0.114366 | 3.63349428 |
| CLDN1 | 1.64268099 | 1.96182313 | 3.79947076 | 0.00028032 | 0.38456392 | -0.0611034 | 3.63284028 |
| LINC01993 | 2.45874077 | -0.1271592 | 3.76821656 | 0.00031095 | 0.38456392 | -0.2096146 | 3.60600036 |
| GPR34 | 2.21926388 | -2.9929216 | 3.75500493 | 0.00031515 | 0.38456392 | -0.218566 | 3.60252047 |
| MLPH | 3.04332964 | -1.8096486 | 3.73093587 | 0.00034852 | 0.38456392 | -0.2250872 | 3.576277 |
| PNLDC1 | -1.7796066 | -3.0513701 | -3.7192083 | 0.00036186 | 0.38456392 | -0.3690696 | -3.5664436 |
| CD74 | 2.16313516 | 2.24833037 | 3.66936941 | 0.00043697 | 0.38456392 | -0.3130844 | 3.51668561 |
| CA3 | 1.51060275 | 0.51492866 | 3.66663829 | 0.00043842 | 0.38456392 | -0.4193094 | 3.51580622 |
| LINC02895 | -2.284348 | -6.2884228 | -3.6493024 | 0.00044225 | 0.38456392 | -0.3796491 | -3.5134951 |
| PIEZO2 | 1.68368983 | 4.86756445 | 3.66035886 | 0.00044513 | 0.38456392 | -0.4440224 | 3.51177106 |
| GOLGA8T | 0.89191919 | -0.2700379 | 3.64538895 | 0.00046772 | 0.38456392 | -0.5447493 | 3.49859086 |
| RAB42 | 1.17755627 | 1.2277891 | 3.64996126 | 0.00047028 | 0.38456392 | -0.483747 | 3.49713772 |
| LGSN | 1.92385293 | -2.000136 | 3.64421825 | 0.0004725 | 0.38456392 | -0.463235 | 3.49588003 |
| APLF | 0.78151089 | 1.45756828 | 3.64349308 | 0.00047891 | 0.38456392 | -0.5038333 | 3.4922821 |
| THBS2-AS1 | 0.56367404 | 0.92861266 | 3.62912438 | 0.00048663 | 0.38456392 | -0.5031232 | 3.4880107 |
| MIR4527HG | -2.2142163 | -5.468121 | -3.6200072 | 0.0005089 | 0.38456392 | -0.5567985 | -3.4760287 |
| LINC02716 | -2.6130191 | -4.7109824 | -3.6115417 | 0.0005164 | 0.38456392 | -0.5860426 | -3.4721015 |
| DPY19L1P1 | 0.60160748 | 2.40918407 | 3.59254199 | 0.00056308 | 0.39272237 | -0.6281442 | 3.44880215 |
| LINC01353 | -2.4283172 | -3.7182537 | -3.5556953 | 0.00061708 | 0.39272237 | -0.7073744 | -3.4239926 |
| PXDNL | 2.02277924 | 2.72947386 | 3.55400844 | 0.00063445 | 0.39272237 | -0.677258 | 3.41644329 |
| RARRES2 | 2.30913507 | 0.00962807 | 3.54970431 | 0.00064417 | 0.39272237 | -0.6844993 | 3.41229989 |
| LINC01503 | 0.8982032 | 1.64398453 | 3.54240907 | 0.00065544 | 0.39272237 | -0.7172811 | 3.40756831 |
| STEAP3 | 1.96985181 | 3.39845624 | 3.53037777 | 0.00068607 | 0.39272237 | -0.7421079 | 3.39508441 |
| MIR3976HG | 2.39381592 | -5.1973116 | 3.51492189 | 0.00070919 | 0.39272237 | -0.8313918 | 3.3860022 |
| TMEM239 | 2.07090568 | -6.5378101 | 3.51050186 | 0.0007192 | 0.39272237 | -0.7740083 | 3.38215223 |
| ICAM1 | 2.91989663 | 0.84598631 | 3.51930375 | 0.00072817 | 0.39272237 | -0.7335441 | 3.37874702 |
| SBSPON | 1.19635307 | 2.06631766 | 3.51557107 | 0.00073043 | 0.39272237 | -0.7815744 | 3.37789655 |
| MIR548XHG | -2.1354655 | -6.3587153 | -3.5031376 | 0.00073344 | 0.39272237 | -0.7923507 | -3.3767635 |
| HLA-H | 1.2508131 | 1.36974075 | 3.51177679 | 0.0007383 | 0.39272237 | -0.7618943 | 3.374947 |
| LINC00458 | 2.69234721 | -0.9410769 | 3.48446338 | 0.00078377 | 0.39471083 | -0.8698038 | 3.35846407 |
| ALOX12 | 0.50407277 | 2.06420543 | 3.49179498 | 0.00078379 | 0.39471083 | -0.8841367 | 3.35845631 |
| VSTM1 | 2.30335478 | -1.5775228 | 3.48818413 | 0.00079504 | 0.39471083 | -0.9007986 | 3.35451587 |
| CXCL2 | 1.92931294 | 1.36773817 | 3.49579324 | 0.00083575 | 0.39786796 | -0.7364317 | 3.34067567 |
| KRT89P | 2.04553163 | -5.8713893 | 3.46348069 | 0.00083702 | 0.39786796 | -0.8732018 | 3.34025437 |
| IL32 | 1.91743435 | -0.9362115 | 3.46282557 | 0.00087031 | 0.40088311 | -0.8552445 | 3.32940736 |
| FAR1-IT1 | -2.0683547 | -5.7839575 | -3.4478384 | 0.00087925 | 0.40088311 | -0.9389542 | -3.326561 |
| FAM86B1 | 0.38610834 | 2.60377316 | 3.42618128 | 0.00096833 | 0.4326703 | -1.0231175 | 3.29956951 |
| CSRP1 | 1.03640614 | 5.47058068 | 3.40660736 | 0.00103031 | 0.43400787 | -1.0527784 | 3.28211535 |
| HMGN1P8 | 1.94816537 | -3.5870699 | 3.40167276 | 0.00103104 | 0.43400787 | -1.1145449 | 3.28191621 |
| ZNF665 | 0.39425562 | 2.43551221 | 3.40090492 | 0.00104115 | 0.43400787 | -1.0720726 | 3.27916406 |
| PBLD | 0.25411368 | 4.7564454 | 3.40087039 | 0.00105544 | 0.43400787 | -1.0835229 | 3.27531639 |
| MATN3 | 1.00814347 | -0.5900624 | 3.39074418 | 0.0011065 | 0.43400787 | -1.0903377 | 3.26194624 |
| RELB | 0.93465748 | 3.07444808 | 3.3845419 | 0.00112214 | 0.43400787 | -1.1269216 | 3.2579657 |
| LINC02321 | -2.3092109 | -4.6914571 | -3.3667184 | 0.00112435 | 0.43400787 | -1.1213846 | -3.2574074 |
| LINC02970 | 2.4827937 | -5.6240562 | 3.38153087 | 0.00112674 | 0.43400787 | -1.1161412 | 3.25680499 |
| CARD10 | 1.16307353 | 3.1158085 | 3.37138652 | 0.00114869 | 0.4349633 | -1.1315674 | 3.25132422 |
| CXCL11 | 2.27445784 | -4.4520466 | 3.35762754 | 0.00119553 | 0.43584826 | -1.0965177 | 3.23994383 |
| ZNF225-AS1 | -0.5380621 | 1.2231151 | -3.3527739 | 0.00122158 | 0.43584826 | -1.2462202 | -3.2337923 |
| HLA-DRB5 | 2.42679432 | -3.3725919 | 3.34641857 | 0.00124161 | 0.43584826 | -1.1710106 | 3.22914385 |
| DISP1 | 0.39260652 | 4.19389861 | 3.34200971 | 0.00125527 | 0.43584826 | -1.2052986 | 3.22601442 |
| BIRC3 | 1.39551001 | 0.09255669 | 3.34028356 | 0.00126216 | 0.43584826 | -1.188471 | 3.22444829 |
| FNIP1 | 0.29229132 | 5.59004672 | 3.35149576 | 0.00127669 | 0.43584826 | -1.1574401 | 3.22116865 |
| BCAS1 | 1.98046326 | 0.77170671 | 3.33207095 | 0.001297 | 0.43584826 | -1.1706908 | 3.21664366 |
| FAM81A | -0.4266955 | 3.28393785 | -3.3306433 | 0.0013071 | 0.43584826 | -1.2333909 | -3.2144172 |
| FMN1 | 1.32365698 | -0.4820185 | 3.32222932 | 0.00133317 | 0.43800477 | -1.2401175 | 3.20874239 |
| C2 | 1.73108463 | 0.60930598 | 3.321015 | 0.00137083 | 0.43970362 | -1.259759 | 3.20072269 |
| GBP2 | 2.41910508 | 0.97009987 | 3.31040752 | 0.00139914 | 0.43970362 | -1.239861 | 3.19482852 |
| LINC00997 | 0.60827367 | 2.92379249 | 3.31030476 | 0.00140403 | 0.43970362 | -1.3086862 | 3.19382044 |
| LINC01508 | 1.59709077 | 0.86065347 | 3.30060011 | 0.00143386 | 0.43970362 | -1.273992 | 3.18774781 |
| GDF11 | -0.3292643 | 6.79763418 | -3.3023236 | 0.00143722 | 0.43970362 | -1.3093535 | -3.1870706 |
| LINC02977 | 0.52874273 | 3.2304383 | 3.2965236 | 0.00145881 | 0.43970362 | -1.3081523 | 3.1827559 |
| RAB32 | 0.87007106 | 2.49264386 | 3.28900591 | 0.0014867 | 0.43970362 | -1.2954794 | 3.17726687 |
| CBLN3 | 0.65522979 | 0.96781551 | 3.28883668 | 0.00149579 | 0.43970362 | -1.3178428 | 3.17549881 |
| RNU1-154P | -2.0843535 | -4.7735223 | -3.2744592 | 0.00155765 | 0.43989884 | -1.3932599 | -3.1637224 |
| DES | 2.52743169 | -1.6716199 | 3.26664917 | 0.00158332 | 0.43989884 | -1.3729671 | 3.15896208 |
| MCAM | 0.63955143 | 5.54066708 | 3.26863643 | 0.00159795 | 0.43989884 | -1.3797211 | 3.15628075 |
| SOCS5P2 | -1.7682284 | -3.1390071 | -3.2604981 | 0.00160575 | 0.43989884 | -1.399998 | -3.1548599 |
| NPY5R | 1.04419838 | 2.04289433 | 3.2604587 | 0.00162495 | 0.43989884 | -1.4209029 | 3.15139095 |
| IRF2 | 0.33953762 | 3.89051417 | 3.25482785 | 0.00166746 | 0.43989884 | -1.4083551 | 3.1438405 |
| HMOX1 | 0.77193061 | 3.21378586 | 3.24418385 | 0.00171456 | 0.43989884 | -1.4354779 | 3.13568058 |
| H4C8 | -0.6382115 | -0.3294703 | -3.243097 | 0.00172071 | 0.43989884 | -1.456734 | -3.1346306 |
| ZNF473CR | -0.4640035 | 0.37752955 | -3.2413673 | 0.00173472 | 0.43989884 | -1.4476055 | -3.1322509 |
| FA2H | 1.54609626 | -1.6176331 | 3.23776458 | 0.00175795 | 0.43989884 | -1.5660722 | 3.12834225 |
| MOCOS | 0.84566092 | -0.1403864 | 3.23416846 | 0.00176071 | 0.43989884 | -1.4612312 | 3.12788236 |
| SGMS1-AS1 | 0.4713866 | 3.06686694 | 3.23149475 | 0.00179043 | 0.43989884 | -1.4725755 | 3.12295803 |
| CTSS | 1.80048371 | 1.36178399 | 3.2310994 | 0.0018057 | 0.43989884 | -1.4253918 | 3.1204583 |
| PURG | -0.5189748 | 4.09828314 | -3.2253644 | 0.00180574 | 0.43989884 | -1.4725435 | -3.1204512 |
| C1orf21 | -0.3137284 | 6.37869118 | -3.2253941 | 0.00181727 | 0.43989884 | -1.4828769 | -3.1185758 |
| MIR1281 | -1.35433 | -2.3578538 | -3.2146505 | 0.00182331 | 0.43989884 | -1.516884 | -3.1175992 |
| VWF | 0.98964639 | -0.6173805 | 3.22355494 | 0.00183119 | 0.43989884 | -1.4874945 | 3.11632732 |
| HOXA3 | -2.2215857 | -6.2759006 | -3.2126173 | 0.00186387 | 0.44298677 | -1.4886909 | -3.1111074 |
| JPH2 | 1.41901503 | 0.52242726 | 3.20146691 | 0.00195833 | 0.45288283 | -1.5472423 | 3.09648038 |
| ACOT8 | -0.2029145 | 4.41964915 | -3.2013715 | 0.00196528 | 0.45288283 | -1.5442633 | -3.0954289 |
| LBHD2 | 1.88661571 | -4.1305969 | 3.19716088 | 0.00196821 | 0.45288283 | -1.5440435 | 3.09498833 |
| CDK6-AS1 | -1.5942324 | -1.8236555 | -3.1928995 | 0.00198659 | 0.45288283 | -1.5889011 | -3.0922291 |
| LINC00562 | 0.70270498 | -1.0500247 | 3.18265669 | 0.00203789 | 0.45888424 | -1.5844935 | 3.08465425 |
| FABP7 | -0.653022 | 7.72531039 | -3.1823752 | 0.00207113 | 0.45888424 | -1.5737059 | -3.0798376 |
| EHF | 1.84793501 | -1.8298502 | 3.1843427 | 0.00207454 | 0.45888424 | -1.6105756 | 3.07934822 |
| RNU2-17P | 2.19486644 | -4.867765 | 3.17529211 | 0.00210369 | 0.46024578 | -1.5835479 | 3.07518985 |
| CXCL5 | 2.45828768 | -1.2849032 | 3.17780152 | 0.0021257 | 0.46024578 | -1.5513908 | 3.07208431 |
| IRAK3 | 0.90254047 | -0.2595728 | 3.17476709 | 0.0021425 | 0.46024578 | -1.6002175 | 3.06973349 |
| ZFX-AS1 | -1.0349547 | -1.9949452 | -3.1553451 | 0.00221657 | 0.47162223 | -1.6427978 | -3.0595676 |
| CHRNA9 | 2.3039693 | 0.96860329 | 3.15622459 | 0.00226478 | 0.47412784 | -1.6331366 | 3.05311608 |
| PDE8B | 0.74086345 | 4.29508641 | 3.15282602 | 0.00227079 | 0.47412784 | -1.6572354 | 3.0523214 |
| CD34 | 2.58167173 | 1.653768 | 3.14525829 | 0.00231488 | 0.47885851 | -1.6326308 | 3.04654486 |
| HOXA2 | -2.3872174 | -5.3931665 | -3.1406992 | 0.0023571 | 0.48311834 | -1.6795274 | -3.0411075 |
| C1S | 1.49200702 | 5.66827481 | 3.13140454 | 0.00244004 | 0.48672814 | -1.6912857 | 3.03068014 |
| C1QTNF6 | 1.09685768 | 5.37577638 | 3.13062302 | 0.00244407 | 0.48672814 | -1.684602 | 3.03018129 |
| APCDD1L | 2.70116128 | -2.1272077 | 3.12279367 | 0.00247275 | 0.48672814 | -1.7326028 | 3.02665661 |
| SNORD91A | 1.24443772 | -2.8675032 | 3.11579641 | 0.00247669 | 0.48672814 | -1.6838934 | 3.02617548 |
| CLEC18B | 1.24635877 | 3.47362837 | 3.12172212 | 0.00250358 | 0.48672814 | -1.7041141 | 3.02290834 |
| OR3A2 | -1.7585685 | -6.3528106 | -3.1180286 | 0.00250543 | 0.48672814 | -1.6901087 | -3.0226853 |
| OSMR-DT | 1.22573393 | -0.347078 | 3.11435126 | 0.00258424 | 0.49771157 | -1.7040042 | 3.013299 |
| SCD5 | -0.4573074 | 8.49377678 | -3.1059869 | 0.00262232 | 0.49945308 | -1.7489172 | -3.008858 |
| CALHM2 | 0.56039528 | 1.85982995 | 3.09764683 | 0.00267628 | 0.49945308 | -1.8003563 | 3.00266406 |
| RETREG1 | 0.61258358 | 6.07582409 | 3.0968189 | 0.00267629 | 0.49945308 | -1.7536117 | 3.00266215 |
| CACNG5 | 1.37432861 | 5.04580976 | 3.08688635 | 0.00273507 | 0.49945308 | -1.7655195 | 2.99604371 |
| C11orf86 | 2.42454054 | -5.4997057 | 3.0851284 | 0.00275384 | 0.49945308 | -1.7500773 | 2.99395807 |
| CACNG8 | -0.4844063 | 6.87288782 | -3.0859568 | 0.00278258 | 0.49945308 | -1.7970137 | -2.9907889 |
| ENPP1 | 0.76120155 | 1.84020542 | 3.07949491 | 0.00283714 | 0.49945308 | -1.807643 | 2.9848542 |
| NFKB2 | 0.68810269 | 4.05047895 | 3.08006794 | 0.00284041 | 0.49945308 | -1.7753467 | 2.98450127 |
| C2CD2 | 0.56795421 | 4.46948428 | 3.07708293 | 0.00285361 | 0.49945308 | -1.8061666 | 2.98308214 |
| HLA-DRB1 | 1.87665948 | -0.8308068 | 3.07564333 | 0.00287485 | 0.49945308 | -1.7807494 | 2.98081174 |
| GABBR2 | -0.6835215 | 8.81516209 | -3.0695437 | 0.00291735 | 0.49945308 | -1.8285993 | -2.9763149 |
| TMEM212 | -1.1778095 | -6.3773476 | -3.0705845 | 0.00291796 | 0.49945308 | -1.8177789 | -2.9762512 |
| SPI1 | 2.15251908 | -3.8310027 | 3.05595367 | 0.00296267 | 0.49945308 | -1.8433357 | 2.97158445 |
| GTF2H3 | 0.1936518 | 5.25701859 | 3.06519968 | 0.0029646 | 0.49945308 | -1.8326014 | 2.97138516 |
| HOXA10 | -1.864642 | -5.5333941 | -3.0623794 | 0.0029763 | 0.49945308 | -1.8330069 | -2.970175 |
| AHCYP3 | -2.0169301 | -5.3863113 | -3.0522647 | 0.00303337 | 0.49945308 | -1.8126276 | -2.9643357 |
| TGFBI | 2.14305859 | 2.25657607 | 3.05464087 | 0.00303829 | 0.49945308 | -1.8159736 | 2.96383679 |
| CHI3L2 | 2.33319013 | -0.2792952 | 3.0564602 | 0.00306002 | 0.49945308 | -1.8013707 | 2.96164288 |
| RCAN1 | 1.20635843 | 7.49960491 | 3.05406808 | 0.00307558 | 0.49945308 | -1.8522076 | 2.96008146 |
| PLIN2 | 0.79889508 | 3.71140022 | 3.04914836 | 0.00311106 | 0.49945308 | -1.8861742 | 2.95654546 |
| LCN2 | 3.08631283 | -4.2713043 | 3.04081249 | 0.00318708 | 0.49945308 | -1.8842744 | 2.94909295 |
| LINC01191 | 2.12389992 | -4.1504713 | 3.03566312 | 0.00318938 | 0.49945308 | -1.8967801 | 2.9488702 |
| TOMM20L | 0.59571333 | -0.7053319 | 3.0376537 | 0.0031894 | 0.49945308 | -1.8938854 | 2.94886832 |
| LDB3 | 0.59560066 | 1.18263431 | 3.03716045 | 0.0031967 | 0.49945308 | -1.9005531 | 2.94816148 |
| SMIM10 | 0.82762201 | -0.1475003 | 3.03801546 | 0.003204 | 0.49945308 | -1.874612 | 2.94745604 |
| PRX | 0.61246215 | 3.29950765 | 3.03701585 | 0.00321929 | 0.49945308 | -1.9041814 | 2.94598417 |
| LAMA1 | -0.6916772 | 4.1771304 | -3.0345376 | 0.00324544 | 0.49945308 | -1.9146283 | -2.9434812 |
| PDE7B | 0.96041757 | 4.02214998 | 3.0308722 | 0.00325035 | 0.49945308 | -1.8926754 | 2.94301283 |
| SP6 | 1.64629185 | 1.10206975 | 3.03077548 | 0.00325314 | 0.49945308 | -1.8845562 | 2.94274718 |
| WNT10A | 1.57102337 | -0.9747805 | 3.02696504 | 0.00329745 | 0.49945308 | -2.0257377 | 2.93855631 |
| SNORA66 | 1.01573321 | -1.8920879 | 3.02614305 | 0.0033065 | 0.49945308 | -1.9114909 | 2.9377068 |
| CCL2 | 1.55865506 | 3.69980997 | 3.02702307 | 0.00335069 | 0.49945308 | -1.8829797 | 2.93358738 |
| FAM87B | 1.08404156 | 0.057661 | 3.01946992 | 0.00339549 | 0.49945308 | -1.9430362 | 2.92946258 |
| GUSBP5 | 1.64730135 | -3.1099943 | 3.01432372 | 0.00340923 | 0.49945308 | -1.9583355 | 2.92820671 |
| CRTC3 | 0.37664145 | 6.23534364 | 3.01817542 | 0.00340998 | 0.49945308 | -1.9373 | 2.92813848 |
| CAV2 | 1.15244461 | 2.98085813 | 3.01727741 | 0.00341919 | 0.49945308 | -1.9090693 | 2.92730019 |
| ITPRID2-DT | 1.18955838 | -1.5862182 | 3.00984842 | 0.00344745 | 0.49945308 | -1.9753419 | 2.92473914 |
| CHI3L1 | 2.78593952 | 1.58672863 | 3.00754434 | 0.00350974 | 0.49945308 | -1.890579 | 2.91916159 |
| FES | 0.84979309 | -0.4701754 | 3.00592305 | 0.00355052 | 0.49945308 | -2.0168 | 2.91555918 |
| ISM2 | -1.0043128 | 1.16657812 | -2.9971215 | 0.00360564 | 0.49945308 | -2.0562985 | -2.9107483 |
| SLCO5A1-AS1 | -0.5300242 | 0.42996294 | -2.9958624 | 0.00364162 | 0.49945308 | -2.0428305 | -2.9076447 |
| EPHA1-AS1 | -1.0294037 | -1.5301893 | -2.9877442 | 0.00367987 | 0.49945308 | -2.0206385 | -2.9043751 |
| MIDEAS | 0.37466159 | 6.19927836 | 2.98876438 | 0.00371352 | 0.49945308 | -2.0005672 | 2.9015246 |
| PIMREGP1 | 2.00130339 | -4.5739576 | 2.97850847 | 0.00372834 | 0.49945308 | -1.990858 | 2.90027611 |
| SERPINB9P1 | 1.02812055 | -0.68357 | 2.9861358 | 0.00374385 | 0.49945308 | -2.0277929 | 2.8989747 |
| LIPH | 1.2244148 | -0.0815339 | 2.98411045 | 0.00374523 | 0.49945308 | -2.031891 | 2.89885923 |
| ASS1 | 1.16165618 | 4.95537136 | 2.9831715 | 0.00376399 | 0.49945308 | -1.996259 | 2.89729252 |
| JDP2 | 0.46050957 | 4.27133938 | 2.98347882 | 0.00377047 | 0.49945308 | -1.9981549 | 2.8967525 |
| IL1R1 | 1.62290977 | 2.69330357 | 2.98124906 | 0.00380063 | 0.49945308 | -2.0192936 | 2.894252 |
| FAM13C | -0.2837555 | 4.49714129 | -2.9792114 | 0.00382428 | 0.49945308 | -2.0391091 | -2.8923039 |
| SIRT4 | -0.4040571 | 0.69652461 | -2.9788904 | 0.00382566 | 0.49945308 | -2.0364522 | -2.8921907 |
| GBP1P1 | 1.99882144 | -5.7531187 | 2.98010402 | 0.00382866 | 0.49945308 | -1.9933479 | 2.89194379 |
| PCSK6 | 1.11182316 | 3.50746129 | 2.97822298 | 0.00384708 | 0.49945308 | -2.0423327 | 2.89043569 |
| IL34 | 1.12283155 | 1.96376999 | 2.97263854 | 0.00387439 | 0.49945308 | -2.0534015 | 2.88821172 |
| TRAC | 1.80067616 | 0.31971439 | 2.96545095 | 0.00391783 | 0.49945308 | -2.0177639 | 2.8847031 |
| STAT5A | 1.06185653 | 2.57839257 | 2.965713 | 0.00397995 | 0.49945308 | -2.05963 | 2.87974684 |
| LINC02721 | -1.9447178 | -5.5842145 | -2.9595573 | 0.003996 | 0.49945308 | -2.0653169 | -2.8784773 |
| NFKB1 | 0.62641073 | 4.36603264 | 2.9638348 | 0.0040035 | 0.49945308 | -2.0559203 | 2.87788593 |
| SERPING1 | 1.21323293 | 4.61375338 | 2.96357327 | 0.00400354 | 0.49945308 | -2.0447692 | 2.87788243 |
| CXCL6 | 1.84134585 | 0.23714581 | 2.96388377 | 0.00401163 | 0.49945308 | -2.0371268 | 2.87724563 |
| HES2 | 1.50971484 | 0.97370154 | 2.95935821 | 0.00403421 | 0.49945308 | -2.0030362 | 2.87547398 |
| LEISA1 | 1.08705735 | 2.09142245 | 2.95536368 | 0.00406283 | 0.49945308 | -2.0617852 | 2.87324168 |
| OLFM2 | -0.4959959 | 7.63354984 | -2.9553423 | 0.00408506 | 0.49945308 | -2.0738999 | -2.8715174 |
| TNFAIP2 | 1.76182367 | 2.93426949 | 2.9539765 | 0.00409196 | 0.49945308 | -2.0739611 | 2.87098408 |
| PRICKLE2-AS3 | 1.63877199 | -5.6752367 | 2.94684318 | 0.00416447 | 0.49945308 | -2.0696652 | 2.86542684 |
| KLHL21 | 0.32521708 | 5.41150171 | 2.94964237 | 0.00417114 | 0.49945308 | -2.0897707 | 2.8649203 |
| PDCD1 | 1.84067469 | -4.6381518 | 2.94539609 | 0.00417242 | 0.49945308 | -2.1009627 | 2.86482332 |
| IL13RA1 | 1.01977023 | 4.64825419 | 2.94940686 | 0.00417553 | 0.49945308 | -2.0723702 | 2.86458712 |
| CP | 2.51251927 | 3.92295268 | 2.94656249 | 0.00420727 | 0.49945308 | -2.0779926 | 2.8621883 |
| MAL | 1.28727948 | -0.6646216 | 2.94090793 | 0.00425379 | 0.49945308 | -2.1306396 | 2.85870164 |
| BMP6 | -1.1731332 | 0.81852501 | -2.9414718 | 0.0042646 | 0.49945308 | -2.1116855 | -2.8578964 |
| OCSTAMP | 2.28858333 | -2.6411313 | 2.93435813 | 0.00428282 | 0.49945308 | -2.1216606 | 2.85654303 |
| CCDC33 | -1.2288145 | -1.721543 | -2.9389562 | 0.00428681 | 0.49945308 | -2.1239174 | -2.8562476 |
| SLC30A7 | 0.27688478 | 5.14886117 | 2.93799691 | 0.00431867 | 0.49945308 | -2.1180564 | 2.85389567 |
| LINC00963 | 0.63823157 | 5.25301117 | 2.9350592 | 0.00435768 | 0.49945308 | -2.1155991 | 2.85103751 |
| GYPC | 1.22462152 | 2.3831307 | 2.93208806 | 0.00436511 | 0.49945308 | -2.106636 | 2.85049635 |
| POMC | -1.8493776 | -3.3867928 | -2.9248956 | 0.00442525 | 0.49945308 | -2.1828115 | -2.846142 |
| LINC01101 | 2.14076774 | -6.2712088 | 2.92130937 | 0.00446829 | 0.49945308 | -2.131669 | 2.84305812 |
| THBS2 | 0.64405221 | 5.21463072 | 2.91962931 | 0.00454814 | 0.49945308 | -2.1433132 | 2.83740857 |
| C2orf72 | -0.7136524 | 5.63307663 | -2.9164071 | 0.00458045 | 0.49945308 | -2.1534633 | -2.8351476 |
| TPRG1 | 1.05282759 | -0.7458755 | 2.91678009 | 0.00459069 | 0.49945308 | -2.1325671 | 2.83443461 |
| ARHGAP1 | 0.22697108 | 6.96990065 | 2.91600357 | 0.00459222 | 0.49945308 | -2.1553781 | 2.83432747 |
| DRD4 | 1.03759084 | 3.35494903 | 2.91617092 | 0.0046064 | 0.49945308 | -2.1483239 | 2.83334242 |
| SEPTIN14P12 | -1.679622 | -5.132077 | -2.9100801 | 0.00463265 | 0.49945308 | -2.1708749 | -2.8315261 |
| LINC02813 | 1.21875971 | -1.4647137 | 2.90555887 | 0.00463617 | 0.49945308 | -2.1820707 | 2.83128255 |
| POGLUT3 | 0.46352178 | 4.80074945 | 2.91331856 | 0.00463788 | 0.49945308 | -2.1576306 | 2.83116523 |
| CEBPD | 1.2609217 | 4.40367652 | 2.91290437 | 0.00464658 | 0.49945308 | -2.1289239 | 2.83056536 |
| HACD4 | 0.89339511 | 2.89026019 | 2.91166516 | 0.00465173 | 0.49945308 | -2.1859276 | 2.83021136 |
| CYP1B1-AS1 | 1.06160377 | -0.5395124 | 2.90985027 | 0.00467108 | 0.49945308 | -2.1578376 | 2.82888309 |
| H2AZP1 | -1.5073936 | -6.3243489 | -2.9057835 | 0.00469882 | 0.49945308 | -2.162185 | -2.8269875 |
| TTLL10-AS1 | -1.4499698 | -5.0854725 | -2.907963 | 0.00469916 | 0.49945308 | -2.1650075 | -2.8269642 |
| SOAT2 | -1.9898483 | -4.9458913 | -2.9047985 | 0.00475586 | 0.49945308 | -2.189247 | -2.8231208 |
| UBE2CP2 | 1.81438876 | -3.9211892 | 2.89972895 | 0.00476893 | 0.49945308 | -2.203473 | 2.8222413 |
| C15orf48 | 1.60749191 | -3.6896232 | 2.88782088 | 0.00487466 | 0.49945308 | -2.1871331 | 2.81520212 |
| NOL4L | -0.2200767 | 7.03870461 | -2.8958294 | 0.00487775 | 0.49945308 | -2.2048514 | -2.8149983 |
| VAMP5 | 1.32066404 | 2.16227441 | 2.89483771 | 0.00488838 | 0.49945308 | -2.1896187 | 2.81429885 |
| TEKT4P2 | -1.5169583 | -6.0758041 | -2.8830426 | 0.005003 | 0.49945308 | -2.2321854 | -2.8068406 |
| HTR1B | -1.0419718 | -0.3906587 | -2.8861729 | 0.00501974 | 0.49945308 | -2.2913616 | -2.805764 |
| EDEM1 | 0.25383661 | 5.02105406 | 2.88325424 | 0.00502848 | 0.49945308 | -2.2259343 | 2.80520359 |
| DNAJA2-DT | 0.86845271 | -0.7200942 | 2.87667722 | 0.00503424 | 0.49945308 | -2.2560377 | 2.80483466 |
| PLA2R1 | 1.0057032 | 1.88803496 | 2.88298279 | 0.00504329 | 0.49945308 | -2.2225724 | 2.80425564 |
| GOLGA4-AS1 | 0.77499075 | 0.56151823 | 2.88243394 | 0.00507724 | 0.49945308 | -2.2108579 | 2.80209205 |
| RHBDF2 | 1.35273833 | -1.1755828 | 2.87760901 | 0.00507867 | 0.49945308 | -2.2058599 | 2.80200111 |
| HOXC9 | -1.9037052 | -5.6123615 | -2.8770019 | 0.00512007 | 0.49945308 | -2.2327984 | -2.799381 |
| ERMAP | 0.51569563 | 3.93576294 | 2.87643306 | 0.00515012 | 0.49945308 | -2.2430602 | 2.79749073 |
| TENT5B | 1.41492812 | 0.98198355 | 2.87261911 | 0.00518368 | 0.49945308 | -2.2330346 | 2.79539213 |
| RNASEH2B-AS1 | 0.59979511 | -0.2020017 | 2.87674496 | 0.00518522 | 0.49945308 | -2.2356689 | 2.79529608 |
| ABCC3 | 1.64548717 | 0.54148157 | 2.87329511 | 0.0051949 | 0.49945308 | -2.2150811 | 2.79469318 |
| UBE2V1P14 | 1.6932605 | -5.6832764 | 2.86985157 | 0.00521769 | 0.49945308 | -2.2639289 | 2.79327756 |
| ACAN | -2.1339112 | -3.0353976 | -2.8679073 | 0.00523929 | 0.49945308 | -2.2879936 | -2.791941 |
| PTPRO | -0.466328 | 6.59578176 | -2.8696266 | 0.00525903 | 0.49945308 | -2.2573865 | -2.7907241 |
| BANCR | 2.13625336 | -5.4743063 | 2.86317722 | 0.00526793 | 0.49945308 | -2.2518092 | 2.79017688 |
| RAMP3 | 1.43269271 | 0.84938053 | 2.86723727 | 0.00528693 | 0.49945308 | -2.2358254 | 2.78901104 |
| P2RX4 | 0.39231494 | 3.54749562 | 2.86778585 | 0.00528938 | 0.49945308 | -2.2560811 | 2.78886074 |
| PPP1R9A-AS1 | 1.44384095 | -1.2003752 | 2.86156489 | 0.00533056 | 0.49945308 | -2.2725264 | 2.78634825 |
| PARP15 | 1.89257329 | -4.6361224 | 2.86252876 | 0.00533309 | 0.49945308 | -2.2823332 | 2.78619455 |
| TICAM1 | 0.58835398 | 1.7539333 | 2.86413666 | 0.00533889 | 0.49945308 | -2.2575242 | 2.78584168 |
| FABP5P11 | -1.9355129 | -5.6408039 | -2.8600963 | 0.00534098 | 0.49945308 | -2.2584799 | -2.7857149 |
| ATP5IF1 | -0.278676 | 4.75411495 | -2.8667504 | 0.005347 | 0.49945308 | -2.2544277 | -2.7853496 |
| CDYL | 0.33531656 | 5.0875829 | 2.86274081 | 0.0053612 | 0.49945308 | -2.2678517 | 2.78448996 |
| CAMSAP1-DT | -0.3382595 | 2.40366436 | -2.8623405 | 0.00536267 | 0.49945308 | -2.2829017 | -2.7844008 |
| HESX1 | 0.64414485 | 0.06293573 | 2.86003374 | 0.0053644 | 0.49945308 | -2.2692879 | 2.78429616 |
| CNKSR2 | -0.4135657 | 4.86234557 | -2.8595877 | 0.00540701 | 0.49945308 | -2.2966667 | -2.7817295 |
| R3HCC1L | 0.21685373 | 3.90185591 | 2.85535935 | 0.00543436 | 0.49945308 | -2.2888488 | 2.78009144 |
| PKP3 | 1.62552551 | -1.8519102 | 2.85493224 | 0.00546248 | 0.49945308 | -2.2576979 | 2.7784152 |
| A2M | 2.17386587 | 6.21128908 | 2.85546565 | 0.00548216 | 0.49945308 | -2.2678238 | 2.77724667 |
| HOXA9 | -1.8320953 | -5.9174621 | -2.8537338 | 0.00548985 | 0.49945308 | -2.2743401 | -2.7767907 |
| IL21-AS1 | -2.0130573 | -5.8045289 | -2.8497821 | 0.00549082 | 0.49945308 | -2.2704287 | -2.7767331 |
| LINC00299 | 1.3015182 | -1.921729 | 2.85263372 | 0.00551853 | 0.49945308 | -2.3350542 | 2.77509657 |
| FGFR3 | -0.6312496 | 5.40537226 | -2.8497121 | 0.00555229 | 0.49945308 | -2.2921487 | -2.7731124 |
| SLC7A2 | 1.02783709 | 4.23826587 | 2.84997626 | 0.00555479 | 0.49945308 | -2.2811138 | 2.77296619 |
| GLCE | -0.2866935 | 5.68352672 | -2.8483718 | 0.00556961 | 0.49945308 | -2.2992887 | -2.772099 |
| PEA15 | -0.4487709 | 9.81085964 | -2.8476095 | 0.00558255 | 0.49945308 | -2.2984153 | -2.7713432 |
| STIM2 | -0.1619699 | 6.23189072 | -2.8483892 | 0.00558897 | 0.49945308 | -2.3006886 | -2.770969 |
| NAMPT | 0.80111714 | 6.62789291 | 2.84468804 | 0.00564893 | 0.5027998 | -2.3013445 | 2.76749275 |
| ZNF221 | -0.3933724 | 3.14488085 | -2.8355817 | 0.00577995 | 0.51158013 | -2.3164988 | -2.7600105 |
| IL18R1 | 1.65304592 | -0.1142294 | 2.83332342 | 0.00579337 | 0.51158013 | -2.3226007 | 2.75925256 |
| IL2RB | 1.84492331 | -2.1029654 | 2.83666631 | 0.0058185 | 0.5117764 | -2.2822771 | 2.7578379 |
| INCA1 | 0.32521951 | 2.3350569 | 2.82959266 | 0.00588146 | 0.51250181 | -2.3514429 | 2.75431805 |
| IFITM3P2 | 2.11049435 | -4.9946417 | 2.82535268 | 0.00592917 | 0.51250181 | -2.3184808 | 2.75167319 |
| LPCAT3 | 0.38371033 | 3.62543216 | 2.82437969 | 0.00596089 | 0.51250181 | -2.3441267 | 2.74992503 |
| AARSD1P1 | 0.53712325 | 0.04091186 | 2.82126073 | 0.00597297 | 0.51250181 | -2.3695078 | 2.74926141 |
| ARHGAP17 | 0.19720054 | 5.89623802 | 2.82135604 | 0.00601768 | 0.51250181 | -2.3547723 | 2.74681672 |
| RAB20 | 1.17557397 | -0.1739545 | 2.81984694 | 0.00604254 | 0.51250181 | -2.3322069 | 2.74546381 |
| C1R | 1.52619577 | 5.42224774 | 2.82047064 | 0.00605877 | 0.51250181 | -2.347599 | 2.74458354 |
| TFB2M | 0.29799476 | 3.46752262 | 2.81680148 | 0.00607267 | 0.51250181 | -2.3597953 | 2.74383139 |
| EDIL3-DT | 0.96893609 | 1.78238594 | 2.81586396 | 0.00610035 | 0.51250181 | -2.362318 | 2.74233798 |
| MMP17 | 0.68205026 | 6.46988106 | 2.81515702 | 0.00610784 | 0.51250181 | -2.3709286 | 2.74193521 |
| DTWD2 | 0.27161325 | 3.93906214 | 2.81388874 | 0.0061496 | 0.51250181 | -2.3681624 | 2.73969633 |
| JDP2-AS1 | 0.49364359 | 0.80358922 | 2.81402477 | 0.00615271 | 0.51250181 | -2.3502089 | 2.73952977 |
| PTPRM | -0.2463104 | 7.34154086 | -2.8118148 | 0.00617021 | 0.51250181 | -2.3728977 | -2.7385961 |
| DOCK2 | 0.9485604 | -0.9126395 | 2.81079937 | 0.0061751 | 0.51250181 | -2.3852003 | 2.73833596 |
| DRAM1 | 0.73943875 | 3.32569767 | 2.81011106 | 0.00621867 | 0.51250181 | -2.3681357 | 2.73602308 |
| DNPEP | 0.30709782 | 5.73510049 | 2.80995391 | 0.00622107 | 0.51250181 | -2.3777854 | 2.73589603 |
| LOXL4 | 0.93377392 | -0.2054548 | 2.80966667 | 0.0062373 | 0.51250181 | -2.3739854 | 2.73503815 |
| MPL | 1.06889207 | -1.4896106 | 2.80763012 | 0.00624991 | 0.51250181 | -2.3965207 | 2.73437329 |
| SIGLEC10 | -1.1321227 | -2.1354122 | -2.8071607 | 0.00627034 | 0.51250181 | -2.3884718 | -2.733299 |
| GMDS-DT | 0.24077219 | 3.75374088 | 2.80721147 | 0.00628555 | 0.51250181 | -2.3866521 | 2.73250084 |
| STX11 | 1.10051985 | 0.55867188 | 2.80089442 | 0.00634091 | 0.5151356 | -2.3917451 | 2.72961094 |
| BDKRB2 | 1.66046143 | -1.0479431 | 2.80047994 | 0.0063646 | 0.51518674 | -2.4194605 | 2.72838123 |
| ELF3 | 1.75449813 | -0.5059265 | 2.80909683 | 0.0064587 | 0.51751468 | -2.4085374 | 2.72353703 |
| ANKRD44-DT | -1.6512891 | -5.8854066 | -2.7937217 | 0.00646725 | 0.51751468 | -2.3974596 | -2.7230997 |
| PHYH | 0.24879988 | 4.61970844 | 2.79515347 | 0.00649841 | 0.51751468 | -2.3986754 | 2.72151178 |
| LRP8 | -0.2616166 | 7.02109111 | -2.7940115 | 0.00652188 | 0.51751468 | -2.4126336 | -2.7203199 |
| NCAN | -0.8156669 | 8.69138897 | -2.7925877 | 0.00653834 | 0.51751468 | -2.4128428 | -2.7194865 |
| LINC02348 | 1.48127274 | -2.4340701 | 2.7900275 | 0.00654326 | 0.51751468 | -2.4154737 | 2.71923737 |
| OSBPL3 | 0.62588263 | 4.72664001 | 2.79084548 | 0.00655551 | 0.51751468 | -2.4133701 | 2.71861884 |
| HINT2 | -0.6460943 | -0.5555638 | -2.7890029 | 0.00662933 | 0.52033899 | -2.4276143 | -2.7149126 |
| AGPAT2 | 0.59601251 | 3.61542745 | 2.78730165 | 0.00663787 | 0.52033899 | -2.4148071 | 2.71448612 |
| LINC02236 | 0.95607309 | -1.421441 | 2.78525233 | 0.0067549 | 0.52599537 | -2.4462616 | 2.70869183 |
| SHANK1 | -0.5867925 | 7.02153951 | -2.7810583 | 0.00675711 | 0.52599537 | -2.4420058 | -2.7085834 |
| MARCHF3 | -0.6121032 | 3.08184651 | -2.7764912 | 0.0067822 | 0.52611538 | -2.453625 | -2.7073533 |
| IL17RE | 0.52789227 | 0.68736284 | 2.77608737 | 0.00681403 | 0.52675489 | -2.4470642 | 2.70579906 |
| DBX2 | -1.8459408 | -2.8368513 | -2.7741676 | 0.00685971 | 0.52845799 | -2.4666227 | -2.7035791 |
| TMPRSS11F | 1.70406878 | -4.8615002 | 2.76787133 | 0.00690719 | 0.52907721 | -2.4381255 | 2.70128595 |
| FOXD2 | -1.7265507 | -5.7292897 | -2.7705301 | 0.00691846 | 0.52907721 | -2.4552918 | -2.7007439 |
| SLC7A10 | -1.3253491 | -1.9352381 | -2.7697921 | 0.0069388 | 0.52907721 | -2.4935947 | -2.6997675 |
| UBE2Q1-AS1 | 1.01109693 | -2.4013167 | 2.76403247 | 0.00700367 | 0.5322072 | -2.4768186 | 2.69666986 |
| MED18 | 0.2128982 | 3.46797228 | 2.76234077 | 0.00703198 | 0.53254762 | -2.4531553 | 2.6953258 |
| ZNF239 | -0.3199925 | 2.86349799 | -2.7628529 | 0.00709154 | 0.5338604 | -2.4819361 | -2.6925146 |
| GCSAM | 1.52199073 | -1.3629754 | 2.75936039 | 0.00715584 | 0.5338604 | -2.5063919 | 2.6895035 |
| PRSS56 | 1.92195634 | -1.1130076 | 2.7558719 | 0.00717579 | 0.5338604 | -2.4538828 | 2.68857404 |
| LINC02207 | 1.55702898 | -3.3221656 | 2.75659301 | 0.00719155 | 0.5338604 | -2.4909489 | 2.6878415 |
| LRRC4 | -0.3592639 | 6.08448198 | -2.7566559 | 0.00720751 | 0.5338604 | -2.4803156 | -2.6871014 |
| QPCT | 1.22790377 | 2.5703677 | 2.75455299 | 0.00722612 | 0.5338604 | -2.5015983 | 2.68623983 |
| LCNL1 | 1.43535026 | 3.08974912 | 2.75402918 | 0.00723558 | 0.5338604 | -2.514096 | 2.68580289 |
| ETV7-AS1 | 1.48055754 | -5.3981963 | 2.75543272 | 0.00724049 | 0.5338604 | -2.4763707 | 2.68557621 |
| TMEM144 | 0.72934391 | 0.90281048 | 2.75450034 | 0.00729107 | 0.53463835 | -2.483008 | 2.68324889 |
| TNXB | 0.49881557 | 4.28741566 | 2.75347637 | 0.00731592 | 0.53463835 | -2.5030566 | 2.68211094 |
| IGF2 | -2.0520933 | -0.6598495 | -2.7482695 | 0.00735462 | 0.53463835 | -2.4940964 | -2.6803457 |
| SYNGR2 | 0.49227888 | 4.12628624 | 2.74994267 | 0.00736143 | 0.53463835 | -2.4940761 | 2.68003557 |
| LACTB2 | 0.43591917 | 2.0845697 | 2.74903586 | 0.00737855 | 0.53463835 | -2.4847922 | 2.6792579 |
| B4GALT4 | 0.38900679 | 5.2897744 | 2.74793284 | 0.00740791 | 0.53463835 | -2.5013813 | 2.67792824 |
| LSG1 | 0.17236689 | 5.70982876 | 2.74682739 | 0.00741855 | 0.53463835 | -2.505493 | 2.67744727 |
| GABRG3 | 0.64875402 | 5.78821834 | 2.7428335 | 0.00750209 | 0.53804579 | -2.5139933 | 2.67369409 |
| SEZ6 | -0.3576981 | 7.58715515 | -2.7421093 | 0.0075211 | 0.53804579 | -2.5164981 | -2.672845 |
| NNMT | 1.63180737 | 0.77423633 | 2.74310285 | 0.00756075 | 0.53804579 | -2.4756176 | 2.67108034 |
| CNMD | 0.99262841 | 0.93538601 | 2.73584936 | 0.00762072 | 0.53804579 | -2.541058 | 2.66842742 |
| LINC02226 | 1.49803543 | -5.7685403 | 2.73312451 | 0.0076278 | 0.53804579 | -2.5233931 | 2.66811579 |
| TRAM2-AS1 | 0.22634806 | 4.35847578 | 2.73412921 | 0.00770441 | 0.53804579 | -2.5277164 | 2.66475665 |
| ABI3BP | -0.8293208 | 0.97892518 | -2.7314286 | 0.00775134 | 0.53804579 | -2.5204493 | -2.6627135 |
| HOXD4 | -2.059011 | -5.3849514 | -2.7306927 | 0.00775767 | 0.53804579 | -2.5365078 | -2.6624385 |
| MMP19 | 1.29069583 | 1.92235479 | 2.73191322 | 0.00777616 | 0.53804579 | -2.517893 | 2.66163733 |
| SH2D4A | 0.91552761 | -0.9014686 | 2.72743313 | 0.00780346 | 0.53804579 | -2.5495479 | 2.66045738 |
| LINC02043 | -1.5067932 | -2.3530519 | -2.7251807 | 0.00782169 | 0.53804579 | -2.5660032 | -2.6596713 |
| GBP5 | 2.11529182 | -0.5062318 | 2.72878175 | 0.00783362 | 0.53804579 | -2.5290802 | 2.65915811 |
| LHFPL3-AS2 | 1.24938602 | -2.0536297 | 2.72694261 | 0.00787041 | 0.53804579 | -2.5594121 | 2.65757906 |
| IFITM1 | 2.30510799 | 3.90353151 | 2.72642558 | 0.00790234 | 0.53804579 | -2.5417172 | 2.65621416 |
| FAM131B | -0.3115067 | 6.88220573 | -2.7241919 | 0.00791469 | 0.53804579 | -2.5525104 | -2.6556874 |
| DCAF5 | -0.1145982 | 7.07463971 | -2.7199439 | 0.00797159 | 0.53804579 | -2.5562054 | -2.6532709 |
| LINC01623 | -1.1937011 | -2.2022777 | -2.7220361 | 0.00797381 | 0.53804579 | -2.5512881 | -2.6531767 |
| NIPAL2 | 0.82642624 | 2.68623571 | 2.71969304 | 0.00799298 | 0.53804579 | -2.5577106 | 2.65236632 |
| PSMD9 | 0.2719146 | 2.89815953 | 2.71731022 | 0.00805175 | 0.53804579 | -2.5619304 | 2.64989207 |
| CHODL | 0.81806529 | 4.83985882 | 2.71732168 | 0.00805861 | 0.53804579 | -2.5703429 | 2.64960414 |
| PTGDS | 1.50129665 | 1.97471768 | 2.71348658 | 0.00810654 | 0.53804579 | -2.5714418 | 2.64760001 |
| TESC | 1.00373838 | 4.80767176 | 2.71453044 | 0.0081096 | 0.53804579 | -2.5593801 | 2.64747238 |
| PHF5AP5 | 1.72307359 | -6.0714412 | 2.7125034 | 0.00812851 | 0.53804579 | -2.5496767 | 2.64668456 |
| SMIM7 | 0.2045017 | 5.19438944 | 2.71448746 | 0.00813068 | 0.53804579 | -2.5710811 | 2.64659442 |
| NCF1C | 1.65650013 | -3.3611484 | 2.70721138 | 0.0081366 | 0.53804579 | -2.5659617 | 2.64634845 |
| MIR4653 | 1.6809472 | -4.8249453 | 2.70501768 | 0.00818734 | 0.53804579 | -2.5697445 | 2.64424468 |
| ZNF496-DT | 0.28940217 | 4.46670581 | 2.70967517 | 0.00823658 | 0.53804579 | -2.5825127 | 2.64221496 |
| CLCA2 | 1.58596981 | -2.8286589 | 2.70599733 | 0.00824515 | 0.53804579 | -2.5821246 | 2.64186245 |
| C1RL | 1.31710706 | 4.07836378 | 2.71090065 | 0.00824627 | 0.53804579 | -2.5711621 | 2.64181674 |
| RAD9B | 0.49120075 | 0.13972395 | 2.70966118 | 0.00825945 | 0.53804579 | -2.5600828 | 2.64127545 |
| RN7SL812P | -1.5562188 | -4.8888578 | -2.7037565 | 0.00831193 | 0.53804579 | -2.5867054 | -2.6391291 |
| CACNG7 | -0.3659563 | 7.54085457 | -2.7056943 | 0.00832479 | 0.53804579 | -2.5836182 | -2.6386049 |
| HOXB9 | -2.3068233 | -5.9076442 | -2.7022331 | 0.00833274 | 0.53804579 | -2.5833734 | -2.6382815 |
| DPP4 | 1.26563649 | 0.95596004 | 2.70339114 | 0.00836336 | 0.53804579 | -2.5877031 | 2.63703753 |
| C22orf31 | 1.04676356 | -0.7718035 | 2.70262064 | 0.00836977 | 0.53804579 | -2.5851694 | 2.63677743 |
| RMDN2 | 0.61463738 | 2.46980479 | 2.70212743 | 0.00838778 | 0.53804579 | -2.5983838 | 2.63604831 |
| EPHA5 | -0.5201896 | 5.93564165 | -2.7028141 | 0.00840007 | 0.53804579 | -2.598965 | -2.6355516 |
| CSAG3 | 2.51497232 | -3.7102597 | 2.70086004 | 0.00844812 | 0.53804579 | -2.5876612 | 2.63361524 |
| CTSO | 0.87117201 | 4.65836976 | 2.69994048 | 0.00847121 | 0.53804579 | -2.5884925 | 2.63268812 |
| RBMS1P1 | 1.20863802 | -3.6905127 | 2.69676245 | 0.00848386 | 0.53804579 | -2.5840207 | 2.63218101 |
| MIR22HG | 0.57647516 | 3.47698844 | 2.70042479 | 0.0085038 | 0.53804579 | -2.6050208 | 2.63138345 |
| PDGFRL | 1.01280607 | 1.07338075 | 2.69926756 | 0.00853391 | 0.53804579 | -2.5944403 | 2.63018243 |
| LIN7A | -0.313898 | 5.38074595 | -2.6907719 | 0.00867747 | 0.53804579 | -2.6219929 | -2.6245062 |
| HOXC6 | -1.7654152 | -5.9700659 | -2.6851595 | 0.00873728 | 0.53804579 | -2.6175045 | -2.6221664 |
| GPIHBP1 | 2.05933537 | -3.4179573 | 2.68777239 | 0.00875247 | 0.53804579 | -2.6387547 | 2.62157426 |
| CALB1 | -0.7067859 | 5.57663905 | -2.6859605 | 0.00876505 | 0.53804579 | -2.6337966 | -2.6210847 |
| GSTZ1 | 0.37341717 | 4.33974012 | 2.68587165 | 0.00880829 | 0.53804579 | -2.6317859 | 2.61940644 |
| NPLOC4 | 0.15913752 | 7.23089928 | 2.68297718 | 0.00881926 | 0.53804579 | -2.6260904 | 2.618982 |
| SLC9A1 | 0.25004672 | 5.58122881 | 2.68177247 | 0.00884066 | 0.53804579 | -2.6354478 | 2.61815532 |
| DNASE1L1 | 0.54725282 | 3.19487481 | 2.68410963 | 0.0088419 | 0.53804579 | -2.6263966 | 2.61810738 |
| MUC5B | -1.6864923 | -2.5599674 | -2.6830865 | 0.00885572 | 0.53804579 | -2.6742216 | -2.6175743 |
| AVPI1 | 0.40773622 | 2.54530445 | 2.68076746 | 0.00885637 | 0.53804579 | -2.623399 | 2.61754914 |
| KCNQ2 | -0.5442314 | 8.17345124 | -2.6827015 | 0.00886947 | 0.53804579 | -2.6335401 | -2.6170449 |
| CPNE4 | 0.63237272 | 6.43494092 | 2.6818144 | 0.00888068 | 0.53804579 | -2.6309771 | 2.61661365 |
| TRAPPC12-AS1 | -0.535762 | 1.30548174 | -2.6800771 | 0.0089268 | 0.53804579 | -2.64849 | -2.6148449 |
| ICOSLG | 1.37892492 | -0.3061149 | 2.68502716 | 0.00893972 | 0.53804579 | -2.6073421 | 2.61435068 |
| ARHGAP29 | 1.01852142 | 5.14424154 | 2.67722681 | 0.008979 | 0.53804579 | -2.639678 | 2.61285263 |
| FRZB | 1.26151721 | 2.93709319 | 2.67763626 | 0.00901479 | 0.53804579 | -2.6444212 | 2.61149275 |
| RBM4B | -0.2287626 | 5.4370713 | -2.6762323 | 0.00903809 | 0.53804579 | -2.645013 | -2.6106099 |
| APOL1 | 1.67624365 | -0.8273233 | 2.67648888 | 0.00904858 | 0.53804579 | -2.6211703 | 2.6102134 |
| FGFBP2 | 1.6161832 | 2.226571 | 2.67326996 | 0.00907586 | 0.53804579 | -2.6515298 | 2.60918347 |
| IL17RC | 0.53261771 | 3.37325263 | 2.66946532 | 0.00920448 | 0.53804579 | -2.65716 | 2.60436439 |
| TLX1 | -2.1470334 | -3.8320478 | -2.6631385 | 0.00929458 | 0.53804579 | -2.6824368 | -2.6010247 |
| ACOXL-AS1 | 0.62724349 | -0.3641611 | 2.66482648 | 0.00929619 | 0.53804579 | -2.6625403 | 2.60096515 |
| NPSR1-AS1 | 1.72705516 | -4.7632867 | 2.6615621 | 0.00933175 | 0.53804579 | -2.6700425 | 2.59965527 |
| AMN1 | -0.2511536 | 3.35976104 | -2.6637977 | 0.00934757 | 0.53804579 | -2.6774542 | -2.5990738 |
| PCSK5 | 0.73968106 | 4.72670666 | 2.661824 | 0.00935068 | 0.53804579 | -2.6723974 | 2.59895953 |
| AMHR2 | 1.78712069 | -5.9317742 | 2.65911875 | 0.00935889 | 0.53804579 | -2.6679569 | 2.59865831 |
| PACSIN2 | -0.1756466 | 5.93301353 | -2.6629099 | 0.0093594 | 0.53804579 | -2.6707834 | -2.5986396 |
| LCIIAR | 0.68289753 | 0.38310502 | 2.65770289 | 0.00940553 | 0.53804579 | -2.6743641 | 2.59695113 |
| MEIS3P2 | 0.71323905 | 1.50935076 | 2.66091619 | 0.00940988 | 0.53804579 | -2.6716452 | 2.59679218 |
| UGT8 | 0.52344262 | 2.38203199 | 2.65959982 | 0.00941742 | 0.53804579 | -2.676755 | 2.5965171 |
| LINC03008 | 1.50581722 | -5.8119292 | 2.65386834 | 0.00947574 | 0.53804579 | -2.6683202 | 2.59439567 |
| EPHB1 | -0.4878116 | 6.80970481 | -2.6582554 | 0.00948429 | 0.53804579 | -2.6812937 | -2.5940854 |
| APAF1 | -0.259564 | 5.53329667 | -2.6575024 | 0.00951015 | 0.53804579 | -2.6843121 | -2.593149 |
| TAPBPL | 0.91282219 | 1.54866144 | 2.65739544 | 0.0095135 | 0.53804579 | -2.6767391 | 2.59302805 |
| UNC119 | -0.2089 | 5.82241961 | -2.6553319 | 0.00956058 | 0.53804579 | -2.68932 | -2.5913297 |
| NAT16 | 0.68587496 | 4.52630941 | 2.6534754 | 0.00956796 | 0.53804579 | -2.6993531 | 2.59106417 |
| FTLP12 | 1.38707459 | -2.6866879 | 2.65230616 | 0.00959045 | 0.53804579 | -2.697264 | 2.59025629 |
| PKP4 | -0.3786062 | 6.15895143 | -2.6523106 | 0.00960816 | 0.53804579 | -2.6888108 | -2.5896213 |
| LINC02916 | 0.60452369 | 0.22574804 | 2.65031233 | 0.00963705 | 0.53804579 | -2.6949458 | 2.5885872 |
| NMRAL1 | 0.34533307 | 4.33474924 | 2.65164354 | 0.00966688 | 0.53804579 | -2.6986084 | 2.58752269 |
| DISP3 | -0.5060635 | 6.21943982 | -2.6504268 | 0.00967286 | 0.53804579 | -2.6998943 | -2.5873098 |
| SPP2 | 1.52731997 | -5.9047879 | 2.64700781 | 0.00974387 | 0.53804579 | -2.7019767 | 2.58478877 |
| CLINT1 | 0.14451753 | 6.26650181 | 2.64759348 | 0.00975013 | 0.53804579 | -2.6996326 | 2.58456714 |
| CHMP1B | 0.59019767 | 5.8263035 | 2.64748743 | 0.00975076 | 0.53804579 | -2.6946263 | 2.58454479 |
| JMJD6 | -0.2451106 | 5.13579328 | -2.648038 | 0.00975923 | 0.53804579 | -2.7082714 | -2.5842456 |
| LINC00443 | -1.6310099 | -5.5940557 | -2.6436885 | 0.00979171 | 0.53804579 | -2.6901856 | -2.5830994 |
| ELAVL3 | -0.3914702 | 8.40181385 | -2.6459464 | 0.00980973 | 0.53804579 | -2.7051231 | -2.5824651 |
| PZP | 1.24173568 | -1.5978276 | 2.64411899 | 0.00983459 | 0.53804579 | -2.7086184 | 2.58159162 |
| RNU6-658P | 1.66519057 | -3.8821266 | 2.64090731 | 0.00983482 | 0.53804579 | -2.7180229 | 2.58158342 |
| CCN3 | 1.11353538 | 3.61289269 | 2.64341126 | 0.00985136 | 0.53804579 | -2.703235 | 2.5810033 |
| AP1AR-DT | 0.43219807 | 1.52509534 | 2.64385092 | 0.0098858 | 0.53804579 | -2.7111845 | 2.57979852 |
| MFSD2B | 0.59307383 | -1.0004829 | 2.63950821 | 0.00994094 | 0.53804579 | -2.7081592 | 2.57787678 |
| BECN1 | 0.19022073 | 5.48390453 | 2.64011187 | 0.00996732 | 0.53804579 | -2.7155982 | 2.57696103 |
| LINC02986 | 0.60716658 | 0.38328701 | 2.64232954 | 0.00997212 | 0.53804579 | -2.7091969 | 2.57679453 |
| CLEC18C | 0.9438874 | -0.8830456 | 2.63760839 | 0.01000146 | 0.53804579 | -2.7063288 | 2.57577899 |
| ITIH6 | -1.6815701 | -5.9424538 | -2.6346008 | 0.01001409 | 0.53804579 | -2.7123799 | -2.5753422 |
| PPP1R14BP4 | 1.76581296 | -5.5510867 | 2.630947 | 0.01007424 | 0.53804579 | -2.7072526 | 2.57327058 |
| ZNRF2P1 | 0.47138561 | 3.36089674 | 2.63513904 | 0.01009582 | 0.53804579 | -2.7239738 | 2.57253019 |
| EIF4A2 | -0.3138064 | 8.3558682 | -2.6339856 | 0.01015144 | 0.53804579 | -2.7257541 | -2.5706277 |
| MTND5P15 | -1.4920856 | -6.0173454 | -2.6301186 | 0.01015644 | 0.53804579 | -2.7235754 | -2.570457 |
| KREMEN1 | 0.44799234 | 6.09612751 | 2.63210708 | 0.01016463 | 0.53804579 | -2.727869 | 2.57017796 |
| CD2BP2-DT | 0.33615677 | 1.11880013 | 2.62965109 | 0.010168 | 0.53804579 | -2.7302704 | 2.57006324 |
| GIMAP2 | -1.7103379 | -6.2135406 | -2.6303804 | 0.01016846 | 0.53804579 | -2.7173265 | -2.5700475 |
| PANK4 | -0.2300357 | 5.46017727 | -2.6324522 | 0.01017989 | 0.53804579 | -2.7359405 | -2.5696583 |
| AMER2 | -0.5298112 | 6.66167828 | -2.6319791 | 0.01018158 | 0.53804579 | -2.7318528 | -2.5696006 |
| HAS2 | 0.95275175 | 2.55528971 | 2.63040964 | 0.01021405 | 0.53804579 | -2.711983 | 2.56849732 |
| MAMDC2 | 2.11670169 | 5.09753914 | 2.63282965 | 0.0102479 | 0.53804579 | -2.7403804 | 2.56735053 |
| TBX19 | 0.47337454 | 0.75125991 | 2.62871871 | 0.0102524 | 0.53804579 | -2.7353705 | 2.56719829 |
| RDH10 | 0.84740113 | 4.93030199 | 2.62772941 | 0.01031826 | 0.53804579 | -2.7382584 | 2.56497712 |
| PRR12 | -0.235781 | 7.40675449 | -2.6266393 | 0.01033534 | 0.53804579 | -2.742239 | -2.5644033 |
| ITPRIPL1 | 1.17634985 | 0.3681032 | 2.62468459 | 0.01033641 | 0.53804579 | -2.7503877 | 2.56436732 |
| GOLGA6C | 1.37978197 | -2.0470987 | 2.62436807 | 0.01037127 | 0.53804579 | -2.7738357 | 2.56319862 |
| PLAT | 1.03230027 | 4.01116966 | 2.62311002 | 0.01039261 | 0.53804579 | -2.7413303 | 2.56248491 |
| H2BC6 | -0.9360584 | -1.348954 | -2.6222087 | 0.01039284 | 0.53804579 | -2.7425726 | -2.5624772 |
| EIF4EP1 | 1.81204289 | -4.1374589 | 2.618227 | 0.01040597 | 0.53804579 | -2.747461 | 2.5620388 |
| FPGS | 0.22890132 | 5.87334204 | 2.62339842 | 0.0104274 | 0.53804579 | -2.746538 | 2.56132428 |
| SMIM19 | 0.22956245 | 4.38209355 | 2.62049762 | 0.01044145 | 0.53804579 | -2.7437238 | 2.56085629 |
| FAM91A1 | 0.32692324 | 6.62995709 | 2.61808142 | 0.0105521 | 0.53804579 | -2.7556062 | 2.55719186 |
| OPHN1 | -0.4269024 | 6.04368754 | -2.6200698 | 0.01056659 | 0.53804579 | -2.7503562 | -2.5567144 |
| BRI3P1 | 1.63021447 | -4.6383609 | 2.61278944 | 0.01060878 | 0.53804579 | -2.7465295 | 2.55532781 |
| CT70 | 1.87885542 | -4.4481897 | 2.61239636 | 0.01061915 | 0.53804579 | -2.757367 | 2.55498783 |
| PARP12 | 0.86544604 | 2.49780553 | 2.61601262 | 0.01063495 | 0.53804579 | -2.7557944 | 2.55447036 |
| HACL1 | 0.22680932 | 3.79419477 | 2.61532966 | 0.010649 | 0.53804579 | -2.7633467 | 2.55401065 |
| ADAM1B | -0.6142791 | -0.6802445 | -2.6137696 | 0.01067513 | 0.53804579 | -2.7682185 | -2.5531572 |
| NUAK2 | 0.63836285 | 3.8400967 | 2.61395738 | 0.01069142 | 0.53804579 | -2.7575106 | 2.55262608 |
| DLK1 | 1.77597501 | 9.39301952 | 2.61296664 | 0.01072328 | 0.53804579 | -2.7680181 | 2.55158942 |
| LINC02988 | 0.68196942 | 0.05019394 | 2.61148719 | 0.0107343 | 0.53804579 | -2.7678608 | 2.55123138 |
| IGSF11-AS1 | -1.6545333 | -6.1425264 | -2.6096595 | 0.01073509 | 0.53804579 | -2.7639646 | -2.5512058 |
| KICS2 | 0.36105046 | 2.90798818 | 2.6104102 | 0.01076751 | 0.53804579 | -2.7702789 | 2.55015474 |
| LINC02424 | 1.41347916 | -5.7761459 | 2.60712215 | 0.01078388 | 0.53804579 | -2.7683373 | 2.54962508 |
| ELK3 | 0.85736568 | 4.26017716 | 2.61078818 | 0.01078445 | 0.53804579 | -2.763914 | 2.54960658 |
| RNU5B-1 | 1.5802856 | -6.3974812 | 2.60564288 | 0.0108069 | 0.53804579 | -2.7641655 | 2.54888141 |
| NDP | 0.65216406 | 3.13553685 | 2.60919615 | 0.01082557 | 0.53804579 | -2.7612265 | 2.54827949 |
| BMP4 | 1.12742117 | 1.41524718 | 2.60813415 | 0.01084579 | 0.53804579 | -2.7692035 | 2.54762865 |
| SCARNA8 | 1.70493672 | -4.5231597 | 2.60412277 | 0.01085069 | 0.53804579 | -2.7821413 | 2.54747092 |
| H19 | -1.8717547 | -1.671343 | -2.6075911 | 0.01086197 | 0.53804579 | -2.7862351 | -2.5471083 |
| HEXA-AS1 | 0.75145786 | -1.4124036 | 2.60534294 | 0.01087647 | 0.53804579 | -2.7821703 | 2.5466428 |
| RN7SL416P | -1.5731706 | -6.4432299 | -2.6025593 | 0.01089622 | 0.53804579 | -2.7736638 | -2.5460095 |
| CIROP | 1.69447943 | -4.1394459 | 2.6020955 | 0.01090401 | 0.53804579 | -2.7692523 | 2.54575996 |
| CAMP | 1.42948872 | -6.8417055 | 2.60214299 | 0.01092393 | 0.53804579 | -2.7702259 | 2.54512291 |
| LINC00880 | 1.52509885 | -3.0902986 | 2.60443742 | 0.01095507 | 0.53804579 | -2.7831553 | 2.54412873 |
| CXCL1 | 2.34731881 | -2.457304 | 2.609699 | 0.01096079 | 0.53804579 | -2.7750382 | 2.5439463 |
| KIAA1549L | -0.349289 | 7.04903786 | -2.6040646 | 0.01099184 | 0.53804579 | -2.7858738 | -2.5429582 |
| RN7SL239P | -1.636539 | -5.1334117 | -2.5983674 | 0.01101694 | 0.53804579 | -2.7853019 | -2.5421611 |
| NFKBIZ | 0.85168209 | 2.89580317 | 2.60249206 | 0.01102326 | 0.53804579 | -2.7731552 | 2.54196047 |
| MGLL | -0.5597207 | 5.16330857 | -2.6014217 | 0.01103017 | 0.53804579 | -2.7909002 | -2.5417416 |
| RNU6-137P | 1.54109915 | -4.9421578 | 2.59852054 | 0.01112785 | 0.53964743 | -2.7918105 | 2.53865819 |
| MSR1 | 2.05538114 | -4.7325229 | 2.59459088 | 0.0111664 | 0.53964743 | -2.8051746 | 2.53744798 |
| SUGT1P2 | 1.7858114 | -3.9667067 | 2.59721933 | 0.01121282 | 0.53964743 | -2.8186496 | 2.53599533 |
| GSTM4 | 0.35183648 | 4.38406189 | 2.5962367 | 0.01121308 | 0.53964743 | -2.7962034 | 2.53598732 |
| CFAP410 | 0.26675163 | 4.27921563 | 2.59069736 | 0.0112154 | 0.53964743 | -2.80161 | 2.53591475 |
| BISPR | 1.57554178 | -3.0786032 | 2.59330326 | 0.01122719 | 0.53964743 | -2.8054748 | 2.53554702 |
| PDIA5 | 0.59918724 | 4.49946958 | 2.59532231 | 0.01123209 | 0.53964743 | -2.7923994 | 2.53539414 |
| TMEM267 | 0.32750971 | 3.20479666 | 2.58981019 | 0.01137761 | 0.54349522 | -2.8091627 | 2.53088216 |
| AFG1L | -0.1979795 | 3.12495015 | -2.5897056 | 0.01139355 | 0.54349522 | -2.8070187 | -2.530391 |
| MYBPC3 | -1.6825452 | -5.263782 | -2.5853803 | 0.01145655 | 0.54349522 | -2.8322875 | -2.5284559 |
| RBM24 | 0.41794088 | 3.65842305 | 2.58710677 | 0.01148044 | 0.54349522 | -2.8170886 | 2.52772442 |
| RNF145 | 0.377555 | 7.35025473 | 2.58426266 | 0.01151822 | 0.54349522 | -2.8141769 | 2.52657081 |
| LINC01289 | -1.6978837 | -3.7830532 | -2.5858672 | 0.011521 | 0.54349522 | -2.8326503 | -2.5264859 |
| PCDH20 | 1.59689651 | -4.6620761 | 2.58017678 | 0.01160727 | 0.54349522 | -2.8137801 | 2.5238645 |
| BZW2 | -0.3123292 | 5.23988284 | -2.5801275 | 0.01170769 | 0.54349522 | -2.836871 | -2.5208344 |
| RHBG | 1.62126215 | -2.3766356 | 2.57866026 | 0.01171175 | 0.54349522 | -2.8391266 | 2.52071238 |
| XPOTP1 | -1.2136553 | -3.0745037 | -2.5741674 | 0.01175021 | 0.54349522 | -2.832349 | -2.5195587 |
| CSRP1-AS1 | 1.09282975 | -1.7766503 | 2.57806345 | 0.01176458 | 0.54349522 | -2.8278292 | 2.51912813 |
| LINC02370 | 1.76256341 | -4.6837875 | 2.57537609 | 0.01177763 | 0.54349522 | -2.8281104 | 2.51873779 |
| TCF4-AS1 | -1.4911279 | -6.6299583 | -2.575658 | 0.01179179 | 0.54349522 | -2.835362 | -2.5183147 |
| MAGEB17 | 1.47632882 | -1.9222545 | 2.57508893 | 0.01180931 | 0.54349522 | -2.8340941 | 2.51779167 |
| LYSMD1 | -0.2339773 | 4.29348928 | -2.5756616 | 0.01184934 | 0.54349522 | -2.8397965 | -2.5165996 |
| ALPK1 | 0.92974927 | 3.58527801 | 2.57568303 | 0.01186081 | 0.54349522 | -2.8386499 | 2.51625874 |
| TTLL7 | -0.2695118 | 5.80370912 | -2.5729435 | 0.01191469 | 0.54349522 | -2.84414 | -2.5146608 |
| PYCARD | 1.27553047 | -2.7239649 | 2.56951024 | 0.01200155 | 0.54349522 | -2.8608689 | 2.51209876 |
| TRAF7 | 0.24315045 | 6.90197515 | 2.56593805 | 0.01205335 | 0.54349522 | -2.8516439 | 2.51057854 |
| MIR3945HG | 1.5229617 | -6.2273236 | 2.56422836 | 0.01207191 | 0.54349522 | -2.8486764 | 2.51003521 |
| UPP2 | -0.8484461 | -0.7120465 | -2.5639132 | 0.01212068 | 0.54349522 | -2.8579669 | -2.5086112 |
| CCDC122 | 0.55565932 | 2.05917673 | 2.56610524 | 0.01215738 | 0.54349522 | -2.8503659 | 2.50754304 |
| DPH6 | 0.22497507 | 3.0384695 | 2.5652469 | 0.01217415 | 0.54349522 | -2.8592946 | 2.5070557 |
| GRIK5 | -0.4775114 | 6.9357503 | -2.5649079 | 0.01217587 | 0.54349522 | -2.8603556 | -2.5070058 |
| CTTN-DT | 0.38095861 | 0.67349167 | 2.5611258 | 0.01217837 | 0.54349522 | -2.8635506 | 2.50693336 |
| SCIN | -0.6826073 | 0.34422533 | -2.5637238 | 0.01219388 | 0.54349522 | -2.8675154 | -2.5064834 |
| LINC03085 | -1.5003482 | -3.6291375 | -2.5599292 | 0.01220657 | 0.54349522 | -2.8649592 | -2.5061155 |
| SLC11A2 | 0.31542596 | 6.75353592 | 2.56435304 | 0.01221457 | 0.54349522 | -2.8575137 | 2.50588406 |
| TASP1 | 0.20606648 | 3.15057437 | 2.56299425 | 0.01223258 | 0.54349522 | -2.8641859 | 2.50536303 |
| PCDH8 | 0.69102008 | 6.5857073 | 2.56055472 | 0.01227027 | 0.54349522 | -2.8645656 | 2.50427471 |
| SHMT1 | 0.39507268 | 4.1646651 | 2.5616692 | 0.01227153 | 0.54349522 | -2.863849 | 2.50423831 |
| EPCAM-DT | -0.5567096 | 1.11106135 | -2.5602375 | 0.01228267 | 0.54349522 | -2.8616827 | -2.5039173 |
| FECH | 0.26863116 | 5.36329012 | 2.56119244 | 0.01229824 | 0.54349522 | -2.8657233 | 2.50346923 |
| RAP1GAP2 | -0.3913703 | 5.44074847 | -2.5613861 | 0.01230833 | 0.54349522 | -2.8744721 | -2.5031789 |
| DKK1 | 1.69901027 | 0.10667837 | 2.5589152 | 0.01231545 | 0.54349522 | -2.8597906 | 2.5029742 |
| DUSP19 | -0.4111357 | 2.47921459 | -2.5580669 | 0.01236695 | 0.54349522 | -2.8666982 | -2.5014971 |
| KIAA0825 | -0.2966286 | 2.51154554 | -2.5544276 | 0.01246708 | 0.54349522 | -2.8820174 | -2.4986403 |
| SIMC1P1 | -0.4294174 | 0.26513338 | -2.5555921 | 0.01250247 | 0.54349522 | -2.8918742 | -2.4976355 |
| LINC01019 | 1.88555271 | -3.9767131 | 2.55149455 | 0.0125375 | 0.54349522 | -2.8838272 | 2.49664326 |
| FKBP14-AS1 | 0.46445884 | 0.08798896 | 2.55011859 | 0.01255919 | 0.54349522 | -2.8811051 | 2.49603027 |
| SNHG4 | 0.64899502 | 0.62466185 | 2.55328056 | 0.01256879 | 0.54349522 | -2.8974617 | 2.49575906 |
| GRIP1 | -0.3783867 | 5.53255458 | -2.5525976 | 0.01257782 | 0.54349522 | -2.8838631 | -2.4955045 |
| TAF7L | 0.768857 | -0.2951725 | 2.55231836 | 0.01257969 | 0.54349522 | -2.8876578 | 2.49545156 |
| SDK1 | 0.6045036 | 7.73781085 | 2.55182346 | 0.01259752 | 0.54349522 | -2.8828869 | 2.49494916 |
| SULT1C2 | 1.92457713 | -1.6878208 | 2.55300709 | 0.01259764 | 0.54349522 | -2.8973019 | 2.49494574 |
| GLYATL2 | -0.5050168 | 0.20657739 | -2.5502652 | 0.01260505 | 0.54349522 | -2.8920744 | -2.4947371 |
| KCTD11 | 0.28720739 | 3.98061546 | 2.5516583 | 0.01261456 | 0.54349522 | -2.8815759 | 2.49446945 |
| TMPRSS7 | 1.68522411 | 1.69210389 | 2.55072971 | 0.01262009 | 0.54349522 | -2.8911698 | 2.49431396 |
| BCL3 | 0.97052274 | 2.74620282 | 2.55151962 | 0.0126284 | 0.54349522 | -2.8797883 | 2.49408019 |
| ANKS1B | -0.4667152 | 6.514046 | -2.550756 | 0.01265462 | 0.54349522 | -2.887237 | -2.493344 |
| HNRNPA1P36 | 1.56584755 | -4.3832305 | 2.54721531 | 0.01266389 | 0.54349522 | -2.8820706 | 2.49308393 |
| TSSK1A | 1.09387063 | -2.9897182 | 2.54944881 | 0.012716 | 0.54349522 | -2.8851601 | 2.49162566 |
| FFAR4 | 1.55621989 | 1.18534234 | 2.54717019 | 0.0127205 | 0.54349522 | -2.8838255 | 2.49149993 |
| FKBP4 | -0.2401815 | 6.1685596 | -2.5482912 | 0.01274926 | 0.54349522 | -2.890995 | -2.4906976 |
| LINC00954 | 1.07470595 | -2.6329975 | 2.54589711 | 0.01277173 | 0.54349522 | -2.9096174 | 2.49007191 |
| ATP5MGP8 | 1.58686495 | -4.9818706 | 2.54239164 | 0.01282063 | 0.54349522 | -2.9035927 | 2.48871327 |
| FREM2 | -0.936226 | 4.15887498 | -2.5452232 | 0.0128305 | 0.54349522 | -2.8993691 | -2.4884396 |
| ANGPT1 | 0.85321224 | 3.30035444 | 2.54469046 | 0.01283236 | 0.54349522 | -2.8880054 | 2.48838813 |
| NOC3L | 0.26262751 | 4.50452009 | 2.54475499 | 0.01284681 | 0.54349522 | -2.9005979 | 2.48798785 |
| GABRB3 | -0.3743376 | 7.55076375 | -2.5425375 | 0.01293646 | 0.54349522 | -2.9024476 | -2.4855137 |
| POTEI | 0.993621 | -1.0066671 | 2.54046348 | 0.01294094 | 0.54349522 | -2.8939591 | 2.48539043 |
| DIRAS3 | 1.10557207 | 5.37341361 | 2.54144127 | 0.01295302 | 0.54349522 | -2.9020188 | 2.48505848 |
| LINC00659 | 1.65294176 | -2.9913406 | 2.53772404 | 0.01301117 | 0.54349522 | -2.9002683 | 2.48346343 |
| TC2N | 0.57584913 | 5.2588953 | 2.54001531 | 0.01302667 | 0.54349522 | -2.9031442 | 2.48303944 |
| MEIS3P1 | 0.5327378 | 2.78949514 | 2.54010179 | 0.01302866 | 0.54349522 | -2.9042379 | 2.48298488 |
| MIR4754 | 1.19462338 | -3.2499722 | 2.53396335 | 0.01303266 | 0.54349522 | -2.9098549 | 2.48287547 |
| SP100 | 1.2761278 | 0.83323936 | 2.53840242 | 0.0130782 | 0.54349522 | -2.9027355 | 2.48163265 |
| CELSR3 | -0.2951388 | 7.65439962 | -2.5370232 | 0.01313029 | 0.54349522 | -2.9135964 | -2.4802159 |
| PCMTD1-DT | 0.24796839 | 4.22598253 | 2.53659548 | 0.01316485 | 0.54349522 | -2.9097785 | 2.4792783 |
| VEPH1 | -0.825791 | 2.70002984 | -2.5335321 | 0.01321508 | 0.54349522 | -2.9158921 | -2.4779199 |
| ATCAY | -0.3532338 | 7.97076966 | -2.5342877 | 0.0132261 | 0.54349522 | -2.918152 | -2.4776225 |
| LINC00642 | -1.5317975 | -3.4031729 | -2.533581 | 0.01324882 | 0.54349522 | -2.92768 | -2.4770101 |
| KCTD18 | 0.20288874 | 4.41879284 | 2.53340518 | 0.01325737 | 0.54349522 | -2.9149572 | 2.4767797 |
| GGTA1 | -1.4149544 | -2.8946269 | -2.5297446 | 0.01331863 | 0.54349522 | -2.928317 | -2.4751338 |
| OGFOD1P1 | -1.545495 | -3.445204 | -2.5279256 | 0.01333074 | 0.54349522 | -2.9376253 | -2.4748091 |
| KHDRBS3 | -0.4960648 | 4.14075071 | -2.5302373 | 0.01334736 | 0.54349522 | -2.9228541 | -2.4743642 |
| NIPAL1 | -0.9180534 | -0.5539162 | -2.5291674 | 0.0133482 | 0.54349522 | -2.9453937 | -2.4743417 |
| COPRS | 0.35362456 | 5.62792765 | 2.52710766 | 0.01341763 | 0.54349522 | -2.9260408 | 2.4724878 |
| MYL12A | 0.54140897 | 4.57247133 | 2.52805741 | 0.01344394 | 0.54349522 | -2.924231 | 2.47178751 |
| SLC16A6P1 | 1.43433647 | -3.6551884 | 2.52470748 | 0.01344615 | 0.54349522 | -2.937635 | 2.47172865 |
| CKAP4 | 0.29912511 | 7.02080611 | 2.52671267 | 0.01346182 | 0.54349522 | -2.929137 | 2.47131225 |
| APOL6 | 1.34951691 | 2.79548519 | 2.52871582 | 0.01346225 | 0.54349522 | -2.9217419 | 2.471301 |
| FBXO32 | 1.01755713 | 5.19753888 | 2.52720264 | 0.01347698 | 0.54349522 | -2.9209481 | 2.47090988 |
| MARCKS | -0.308544 | 10.2603747 | -2.5256405 | 0.01351805 | 0.54349522 | -2.9318262 | -2.4698215 |
| KRT7 | 1.79316692 | -4.2474737 | 2.52507791 | 0.01352532 | 0.54349522 | -2.9257703 | 2.4696292 |
| MTUS1-DT | 1.63288801 | -5.2577195 | 2.52142023 | 0.0135613 | 0.54349522 | -2.9295939 | 2.46867856 |
| SLC24A1 | 0.31726769 | 3.43613622 | 2.52365233 | 0.01359608 | 0.54349522 | -2.9327983 | 2.46776164 |
| HOPX | -1.0799444 | 3.16657209 | -2.5219822 | 0.01362944 | 0.54349522 | -2.9415872 | -2.4668842 |
| CBLL1-AS1 | -0.3499782 | 0.91422526 | -2.5224181 | 0.01363516 | 0.54349522 | -2.9462729 | -2.466734 |
| SLC18B1 | 0.55567536 | 6.09320385 | 2.5221394 | 0.0136454 | 0.54349522 | -2.9378915 | 2.4664651 |
| NOG | -0.6071214 | 3.40468561 | -2.5218796 | 0.01364779 | 0.54349522 | -2.9376726 | -2.4664026 |
| RNF128 | -0.6109906 | 1.37735047 | -2.5210656 | 0.01365353 | 0.54349522 | -2.9502096 | -2.4662519 |
| DLG4 | -0.2449574 | 8.01544203 | -2.5222781 | 0.01365397 | 0.54349522 | -2.9397845 | -2.4662403 |
| ARR3 | 0.62973256 | -0.6229455 | 2.52111868 | 0.01367653 | 0.54349522 | -2.9532366 | 2.46564899 |
| CTXND1 | -0.5582839 | 4.47950553 | -2.5205383 | 0.01368121 | 0.54349522 | -2.9432244 | -2.4655263 |
| MIR4324 | 1.29085656 | -3.2107524 | 2.51713916 | 0.01369335 | 0.54349522 | -2.9391468 | 2.46520849 |
| KCNIP2 | -0.7163 | 3.66965102 | -2.5196021 | 0.01369684 | 0.54349522 | -2.9465844 | -2.4651172 |
| AFG2B | -0.2104534 | 3.92254137 | -2.5198811 | 0.01370125 | 0.54349522 | -2.9476663 | -2.4650021 |
| TRADD | 0.49611434 | 2.03156801 | 2.51975893 | 0.01372418 | 0.54349522 | -2.9407525 | 2.46440283 |
| KCNJ1 | -1.9894639 | -5.1827136 | -2.5135565 | 0.01375049 | 0.54349522 | -2.9597405 | -2.4637162 |
| PSMB10 | 0.66370206 | 1.03100395 | 2.51818659 | 0.01379721 | 0.54349522 | -2.9406088 | 2.46250028 |
| RIN3 | 0.46323631 | 2.67031738 | 2.51763259 | 0.01380676 | 0.54349522 | -2.9444449 | 2.46225199 |
| GZF1 | -0.1498893 | 4.9322334 | -2.5173062 | 0.01382198 | 0.54349522 | -2.9486753 | -2.461857 |
| MEIS1-AS3 | 0.90043008 | -1.6327709 | 2.51612472 | 0.01386922 | 0.54349522 | -2.9399803 | 2.46063287 |
| LINC02674 | -1.0821895 | -5.850659 | -2.5147593 | 0.01387984 | 0.54349522 | -2.9567715 | -2.4603582 |
| EEPD1 | -0.5230133 | 4.50080056 | -2.5153957 | 0.01389949 | 0.54349522 | -2.9530211 | -2.4598506 |
| UNC93A | 2.19785478 | -4.4497613 | 2.51237802 | 0.01394965 | 0.54349522 | -2.9510879 | 2.45855734 |
| NIPAL4-DT | 1.32945321 | -3.050946 | 2.51064503 | 0.01398241 | 0.54349522 | -2.9606401 | 2.45771502 |
| CD47 | 0.34132937 | 7.59096396 | 2.5136906 | 0.01398706 | 0.54349522 | -2.9512713 | 2.45759561 |
| LTF | 3.42778956 | -4.3158582 | 2.50987546 | 0.01402037 | 0.54349522 | -2.9600644 | 2.45674113 |
| PDE3A | 0.6478944 | 6.79064876 | 2.5111977 | 0.01403605 | 0.54349522 | -2.9571797 | 2.45633954 |
| PROS1 | 0.77427935 | 5.36478834 | 2.51046128 | 0.01407326 | 0.54349522 | -2.9553089 | 2.4553881 |
| CYP27A1 | 0.84559361 | 3.53626595 | 2.50986475 | 0.01408445 | 0.54349522 | -2.9588225 | 2.45510242 |
| PAX5 | -1.1265936 | -1.592939 | -2.5099553 | 0.01408555 | 0.54349522 | -2.9661841 | -2.4550743 |
| RHOBTB1 | 0.39911514 | 5.30510612 | 2.50645982 | 0.01419695 | 0.54349522 | -2.967137 | 2.45224121 |
| HES4 | -0.4050849 | 5.92968476 | -2.5061967 | 0.01421231 | 0.54349522 | -2.9714121 | -2.4518521 |
| CAMKK1 | -0.2956332 | 4.27080095 | -2.5064753 | 0.01422077 | 0.54349522 | -2.9775449 | -2.4516378 |
| CIAPIN1P | 1.55538651 | -3.9611417 | 2.50245793 | 0.01422242 | 0.54349522 | -2.9680231 | 2.45159621 |
| KRT17P3 | -1.4307345 | -5.2663618 | -2.5044866 | 0.01422753 | 0.54349522 | -2.9699427 | -2.4514668 |
| SUSD1 | 0.44793753 | 4.70921135 | 2.50529102 | 0.01426805 | 0.54349522 | -2.9664628 | 2.45044307 |
| LINC00607 | 1.15488262 | -1.9667975 | 2.50325369 | 0.0142853 | 0.54349522 | -2.970235 | 2.45000812 |
| CTHRC1 | 1.3216907 | 1.01713817 | 2.5026377 | 0.01429576 | 0.54349522 | -2.9936988 | 2.44974448 |
| VIPAS39 | -0.2080807 | 4.82521939 | -2.5038355 | 0.01430486 | 0.54349522 | -2.9740227 | -2.4495154 |
| ROBO2 | -0.5691686 | 7.0480288 | -2.5019521 | 0.01434833 | 0.54349522 | -2.9762035 | -2.4484225 |
| CPM | 1.0056312 | -0.2423528 | 2.50126594 | 0.01436487 | 0.54349522 | -2.9823941 | 2.44800744 |
| SPON2 | 0.85948726 | 1.69172585 | 2.50083274 | 0.01441474 | 0.54349522 | -2.9726067 | 2.44675859 |
| PLSCR1 | 0.73773904 | 2.76519896 | 2.50111746 | 0.0144313 | 0.54349522 | -2.9687954 | 2.44634468 |
| QRICH2 | -0.4107605 | 3.75497561 | -2.5000726 | 0.0144602 | 0.54349522 | -2.9883681 | -2.4456232 |
| RNU4-47P | 1.17124463 | -3.876984 | 2.50083298 | 0.01446856 | 0.54349522 | -2.9826387 | 2.44541476 |
| EPHA5-AS1 | -0.4862958 | -0.0939021 | -2.4995816 | 0.01450213 | 0.54349522 | -2.9858028 | -2.4445791 |
| DGKB | -0.8570109 | 2.80856993 | -2.4981064 | 0.0145544 | 0.54349522 | -2.9869714 | -2.443281 |
| ITPKB-IT1 | 1.60886607 | -6.0282751 | 2.49566753 | 0.01456789 | 0.54349522 | -2.9910928 | 2.44294673 |
| LINC01823 | 1.50659699 | -4.2520025 | 2.49283469 | 0.01458646 | 0.54349522 | -2.9911839 | 2.44248689 |
| SLC8B1 | 0.34296283 | 3.43576455 | 2.49531096 | 0.01462749 | 0.54349522 | -2.9876158 | 2.44147276 |
| HOMER2 | -0.3345728 | 5.46728332 | -2.4951252 | 0.01463804 | 0.54349522 | -2.9941252 | -2.4412125 |
| ANKRD33 | 1.44856559 | -1.5502999 | 2.49825187 | 0.01466834 | 0.54349522 | -3.0145829 | 2.44046562 |
| ZFAND2A-DT | 0.25416054 | 3.31198916 | 2.49444898 | 0.01469173 | 0.54349522 | -2.9887438 | 2.43989018 |
| HPS1 | 0.54488256 | 3.56745858 | 2.49460735 | 0.01469335 | 0.54349522 | -2.9937143 | 2.43985038 |
| CD109 | 0.91627019 | 3.5557066 | 2.49397038 | 0.01469366 | 0.54349522 | -2.9866083 | 2.4398426 |
| HIBADH | 0.29454907 | 4.01675077 | 2.4923897 | 0.01475009 | 0.54394095 | -2.9914149 | 2.43845746 |
| HLA-DQA1 | 1.93055688 | -3.9037047 | 2.49126034 | 0.01475441 | 0.54394095 | -2.9912933 | 2.43835179 |
| SDC4 | 1.23007015 | 2.84411279 | 2.49182958 | 0.01479138 | 0.54394558 | -2.99145 | 2.43744705 |
| PDZK1IP1 | 1.88607975 | -6.1482299 | 2.48920544 | 0.01484145 | 0.54394558 | -2.9898618 | 2.43622497 |
| ARHGAP33 | -0.3738603 | 7.88858158 | -2.4872689 | 0.01495698 | 0.54394558 | -3.0052467 | -2.433419 |
| MIR4453HG | -0.2187677 | 3.65111175 | -2.4869362 | 0.01496937 | 0.54394558 | -3.01073 | -2.4331192 |
| GSDMC | 1.3930409 | -0.1711162 | 2.48729341 | 0.01498804 | 0.54394558 | -3.0366391 | 2.43266797 |
| TUBB8 | -1.2396719 | -3.1265108 | -2.4843072 | 0.01499491 | 0.54394558 | -3.0132989 | -2.432502 |
| IL4I1 | 1.57303462 | -2.5817863 | 2.48271437 | 0.0150527 | 0.54394558 | -3.0151541 | 2.43110865 |
| RNU6-516P | 0.7700096 | -1.9195036 | 2.48138102 | 0.01505933 | 0.54394558 | -3.0079121 | 2.43094919 |
| MAP3K7CL | 0.94340155 | -2.0471501 | 2.48358305 | 0.01506728 | 0.54394558 | -3.0024438 | 2.43075788 |
| CCDC148 | -0.458805 | 1.54186071 | -2.4813248 | 0.01510126 | 0.54394558 | -3.0119017 | -2.4299416 |
| FOXE1 | -1.6750688 | -3.4125775 | -2.4786718 | 0.01514375 | 0.54394558 | -3.0176786 | -2.4289229 |
| LINC02055 | -1.1917399 | -2.6412573 | -2.47995 | 0.01515691 | 0.54394558 | -3.0228146 | -2.428608 |
| RINL | 0.49213955 | 0.58609183 | 2.48092828 | 0.01516559 | 0.54394558 | -3.0222358 | 2.42840035 |
| LINC01498 | -1.6102994 | -4.2522178 | -2.4764239 | 0.01517112 | 0.54394558 | -3.022458 | -2.4282682 |
| H3C6 | -0.6103115 | -0.0017028 | -2.4809673 | 0.01517643 | 0.54394558 | -3.0243543 | -2.4281412 |
| KLHL38 | 1.65233901 | -6.0655184 | 2.48347463 | 0.01518764 | 0.54394558 | -3.0023415 | 2.42787355 |
| RBP4 | 1.09898944 | 3.34946589 | 2.47977616 | 0.01521904 | 0.54394558 | -3.0145779 | 2.42712425 |
| MED15P5 | 1.53927241 | -5.8194321 | 2.47893287 | 0.01522625 | 0.54394558 | -3.0150185 | 2.42695249 |
| LGALS3 | 0.99899944 | 3.89357708 | 2.47776409 | 0.01531331 | 0.54394558 | -3.0132755 | 2.42488319 |
| PPDPF | -0.3163366 | 7.00707955 | -2.4758881 | 0.01535685 | 0.54394558 | -3.0232572 | -2.4238522 |
| AP4S1 | -0.211691 | 4.46411729 | -2.475786 | 0.01536249 | 0.54394558 | -3.0250947 | -2.4237188 |
| HNRNPH3 | -0.2912967 | 7.20265827 | -2.4761267 | 0.01536439 | 0.54394558 | -3.0233629 | -2.423674 |
| RBM26-AS1 | 0.37429215 | 3.61691218 | 2.47564635 | 0.01537004 | 0.54394558 | -3.0241207 | 2.42354055 |
| KLK14 | -1.030928 | -0.9750863 | -2.4739309 | 0.01537145 | 0.54394558 | -3.0503976 | -2.4235071 |
| RN7SL154P | 1.54055711 | -5.3592002 | 2.47302671 | 0.01538715 | 0.54394558 | -3.0203678 | 2.42313642 |
| GALNT1 | 0.21021522 | 7.00072636 | 2.47532839 | 0.01538757 | 0.54394558 | -3.0242853 | 2.42312654 |
| MALT1 | 0.2169739 | 5.16496657 | 2.47489315 | 0.01543887 | 0.54489687 | -3.02815 | 2.42191723 |
| KCNK10 | -0.5877435 | 4.51449684 | -2.4746283 | 0.0154664 | 0.54500766 | -3.0264009 | -2.4212696 |
| H4C14 | -1.5918723 | -4.7369098 | -2.4701262 | 0.0155284 | 0.54546294 | -3.0269994 | -2.4198151 |
| SLC35A2 | 0.26601566 | 4.88811215 | 2.47187432 | 0.0155549 | 0.54546294 | -3.033264 | 2.41919499 |
| LINC02640 | 1.56663288 | -5.0417668 | 2.46905133 | 0.015566 | 0.54546294 | -3.0399292 | 2.41893557 |
| SUMF2 | 0.16995134 | 6.12659177 | 2.47102083 | 0.01560296 | 0.54546294 | -3.0338236 | 2.41807263 |
| MAP3K6 | 0.37947254 | 4.36134848 | 2.47022589 | 0.01560806 | 0.54546294 | -3.0321896 | 2.4179538 |
| IFI16 | 0.75608452 | 4.29722845 | 2.4711613 | 0.01562581 | 0.54546294 | -3.0278627 | 2.41754009 |
| PRUNE2 | 0.39774466 | 6.10876096 | 2.46872631 | 0.01567335 | 0.54626877 | -3.0348145 | 2.41643444 |
| OPTC | 1.52740457 | -4.975833 | 2.46556971 | 0.01569835 | 0.54628781 | -3.0352257 | 2.41585417 |
| TNFRSF11B | 1.57337702 | 3.88933441 | 2.46678656 | 0.01576239 | 0.54716644 | -3.0350832 | 2.41437136 |
| IL15 | 1.33776219 | -1.1385079 | 2.46594483 | 0.01577258 | 0.54716644 | -3.0415294 | 2.41413588 |
| GRIK3 | -0.6581895 | 6.79322945 | -2.4631625 | 0.01587288 | 0.5479474 | -3.0510985 | -2.4118255 |
| KIF13B | 0.33310797 | 5.13486604 | 2.46402964 | 0.01588064 | 0.5479474 | -3.046003 | 2.41164725 |
| TBCK | -0.2321085 | 5.32050284 | -2.4619694 | 0.01592285 | 0.5479474 | -3.0484809 | -2.4106792 |
| LINC00652 | -0.8092987 | 0.62095772 | -2.4618352 | 0.01598694 | 0.5479474 | -3.0653949 | -2.4092135 |
| ATP1A2 | -0.9605112 | 8.72636833 | -2.4593591 | 0.01599645 | 0.5479474 | -3.0534621 | -2.4089965 |
| RN7SL787P | -1.3435131 | -5.9656269 | -2.4580443 | 0.01601458 | 0.5479474 | -3.0576693 | -2.408583 |
| RBM3 | 0.37872983 | 8.32167835 | 2.45894005 | 0.01606284 | 0.5479474 | -3.0551201 | 2.40748449 |
| ARSD | 0.32183261 | 5.50666054 | 2.45969886 | 0.01607217 | 0.5479474 | -3.0541469 | 2.40727247 |
| SLC25A21 | 0.44428755 | 0.88356821 | 2.45786821 | 0.0160868 | 0.5479474 | -3.062738 | 2.40694032 |
| OSMR | 1.18721505 | 4.60306126 | 2.46013176 | 0.01608848 | 0.5479474 | -3.0489222 | 2.40690205 |
| WBP2P1 | -1.3491572 | -6.6368643 | -2.4562055 | 0.01609025 | 0.5479474 | -3.0493982 | -2.406862 |
| LINC01460 | 1.48677645 | -3.1965498 | 2.45637973 | 0.01609157 | 0.5479474 | -3.064602 | 2.40683195 |
| DYRK3-AS1 | 1.20605934 | -2.8497237 | 2.45759252 | 0.01617071 | 0.5479474 | -3.0616368 | 2.40503968 |
| TTC39C | 0.42522703 | 4.47576303 | 2.45525781 | 0.01619382 | 0.5479474 | -3.0595688 | 2.40451777 |
| TFPI2 | 1.85659174 | 5.82896175 | 2.45664551 | 0.01620191 | 0.5479474 | -3.0569627 | 2.40433526 |
| SLC35C2 | 0.15251205 | 6.01171495 | 2.45615582 | 0.01621594 | 0.5479474 | -3.0616123 | 2.40401871 |
| PRKAG3 | 1.60539276 | -5.3860792 | 2.45253349 | 0.01621807 | 0.5479474 | -3.063719 | 2.40397073 |
| PODXL | 0.78886194 | 6.84746306 | 2.45492941 | 0.01623657 | 0.5479474 | -3.0603563 | 2.40355401 |
| ANXA4 | 0.35144021 | 3.62629203 | 2.45381193 | 0.01630915 | 0.54825499 | -3.0605705 | 2.40192277 |
| LINC01614 | 2.0781209 | -5.4063968 | 2.45059202 | 0.01633312 | 0.54825499 | -3.0740545 | 2.40138546 |
| IRAK4 | 0.32852447 | 3.80338002 | 2.45296701 | 0.01635122 | 0.54825499 | -3.0644648 | 2.40098016 |
| PLB1 | 0.77935348 | 2.63609962 | 2.45127345 | 0.01635571 | 0.54825499 | -3.0680863 | 2.40087986 |
| GHSR | -1.6453058 | -6.1946184 | -2.448463 | 0.01639601 | 0.54825499 | -3.070824 | -2.399979 |
| HLA-DMA | 1.18151927 | 1.12740236 | 2.4509837 | 0.01641725 | 0.54825499 | -3.072998 | 2.39950508 |
| ABCG8 | 1.28603855 | -3.2091372 | 2.44851351 | 0.01641747 | 0.54825499 | -3.073178 | 2.39950032 |
| FCER1G | 0.767979 | -1.6452367 | 2.45029278 | 0.01647693 | 0.54882713 | -3.0765432 | 2.39817628 |
| PCP4 | -0.4360033 | 3.80003344 | -2.4490059 | 0.0165105 | 0.54882713 | -3.0772921 | -2.3974308 |
| ZNF114-AS1 | 1.55409891 | -5.6759536 | 2.44709288 | 0.01654138 | 0.54882713 | -3.0722958 | 2.39674622 |
| PTPRQ | 1.25452842 | -2.3656461 | 2.44589674 | 0.01655333 | 0.54882713 | -3.0823249 | 2.39648149 |
| KIF25-AS1 | 0.79770722 | -1.0720604 | 2.44719328 | 0.01655879 | 0.54882713 | -3.0873488 | 2.39636071 |
| LINC01891 | 1.55213114 | -5.4689695 | 2.44501024 | 0.01659383 | 0.54882713 | -3.0763036 | 2.39558586 |
| SNORA79B | 1.27987039 | -2.7965477 | 2.44423106 | 0.01660656 | 0.54882713 | -3.0822578 | 2.39530468 |
| MX1 | 0.90620142 | 3.16768081 | 2.44579497 | 0.01665673 | 0.549672 | -3.075704 | 2.39419859 |
| TFRC | 0.28875752 | 6.87424247 | 2.44431095 | 0.0167013 | 0.54973449 | -3.0818456 | 2.39321832 |
| MTX1P1 | 0.41466478 | 2.45216872 | 2.44283076 | 0.01676527 | 0.54973449 | -3.0842032 | 2.39181537 |
| RABGAP1 | -0.1898729 | 7.13840225 | -2.4418196 | 0.0167676 | 0.54973449 | -3.0853696 | -2.3917644 |
| PEG3 | -0.5954097 | 2.29264667 | -2.443331 | 0.01676902 | 0.54973449 | -3.0797043 | -2.3917335 |
| SBF2-AS1 | 0.43462868 | 0.68888535 | 2.43919327 | 0.01691321 | 0.54973449 | -3.0949553 | 2.38858897 |
| RBM15 | -0.291824 | 3.76621649 | -2.4392889 | 0.01697504 | 0.54973449 | -3.095269 | -2.3872479 |
| CYB5R1 | 0.36727677 | 5.55707008 | 2.43813921 | 0.01698144 | 0.54973449 | -3.0935904 | 2.38710941 |
| GPR39 | 0.59314493 | 0.15071375 | 2.43403424 | 0.01711908 | 0.54973449 | -3.1026566 | 2.38414017 |
| SHANK2 | -0.4573463 | 6.19594466 | -2.4342279 | 0.01715419 | 0.54973449 | -3.1037109 | -2.3833861 |
| SEMA6B | -0.4454045 | 6.56500611 | -2.4337684 | 0.01717664 | 0.54973449 | -3.1045525 | -2.3829047 |
| WASF1 | -0.2734422 | 6.78066839 | -2.4334046 | 0.01719595 | 0.54973449 | -3.1042289 | -2.382491 |
| OXT | 1.76116964 | -4.0957355 | 2.43089986 | 0.01719777 | 0.54973449 | -3.116363 | 2.38245214 |
| CYP4Z1 | -1.6609663 | -4.8520975 | -2.4319304 | 0.01720318 | 0.54973449 | -3.1088396 | -2.3823362 |
| NFKBIA | 0.59705878 | 4.78158344 | 2.43308804 | 0.01721975 | 0.54973449 | -3.1016197 | 2.38198169 |
| ATG13 | 0.15969435 | 6.72627437 | 2.43215728 | 0.0172455 | 0.54973449 | -3.1039375 | 2.38143147 |
| BCL10-AS1 | 0.64829337 | -1.3756238 | 2.42959287 | 0.01726976 | 0.54973449 | -3.1063069 | 2.38091368 |
| APOBEC3G | 1.48411888 | -0.9389682 | 2.43112457 | 0.01728937 | 0.54973449 | -3.099665 | 2.38049545 |
| ALOX15 | -1.5631884 | -2.4366487 | -2.4307987 | 0.01731093 | 0.54973449 | -3.13972 | -2.3800363 |
| LASP1NB | -0.4794238 | 1.38225659 | -2.4306026 | 0.01732402 | 0.54973449 | -3.1082763 | -2.3797578 |
| FUT9 | -0.4908213 | 7.13439106 | -2.4305495 | 0.0173243 | 0.54973449 | -3.1077098 | -2.3797519 |
| ZBTB34 | -0.2883016 | 4.98712256 | -2.4301124 | 0.01732597 | 0.54973449 | -3.1119857 | -2.3797163 |
| PTBP3 | 0.30260583 | 6.33042119 | 2.4291795 | 0.01737017 | 0.54973449 | -3.1082718 | 2.37877726 |
| TACC2 | -0.2931446 | 6.55632249 | -2.4289499 | 0.01738266 | 0.54973449 | -3.111781 | -2.3785122 |
| DUSP3 | 0.33927035 | 6.18054828 | 2.42671896 | 0.01740695 | 0.54973449 | -3.1118672 | 2.37799735 |
| FAM210CP | 1.42697207 | -6.4722411 | 2.42497231 | 0.01741511 | 0.54973449 | -3.1134903 | 2.37782464 |
| ADGRV1 | -0.7695156 | 6.67707892 | -2.4268659 | 0.01741562 | 0.54973449 | -3.113931 | -2.3778138 |
| QPCTL | 0.35472928 | 3.87311887 | 2.42793611 | 0.0174226 | 0.54973449 | -3.1145321 | 2.37766604 |
| LINC00165 | -1.2502038 | -6.6971268 | -2.4247841 | 0.01743329 | 0.54973449 | -3.1150092 | -2.3774397 |
| RN7SL146P | 1.72957187 | -5.0948821 | 2.42220408 | 0.01746245 | 0.54973449 | -3.1115838 | 2.37682341 |
| CPB1 | 1.39847691 | -1.4644293 | 2.42477331 | 0.01754037 | 0.54973449 | -3.1159411 | 2.37518057 |
| WSCD2 | -0.6507867 | 3.90123258 | -2.4241867 | 0.01757427 | 0.54973449 | -3.1227097 | -2.3744677 |
| IP6K3 | 2.00689139 | -4.1632958 | 2.42147164 | 0.01768601 | 0.54973449 | -3.1223673 | 2.37212697 |
| ATL3 | 0.40823557 | 5.31547605 | 2.42219301 | 0.01768715 | 0.54973449 | -3.1209543 | 2.37210328 |
| ELOVL2 | -0.3795146 | 5.78222787 | -2.4207683 | 0.0177187 | 0.54973449 | -3.1241513 | -2.3714446 |
| FHL1P1 | -0.5212317 | -0.2370956 | -2.4210493 | 0.01772201 | 0.54973449 | -3.12442 | -2.3713757 |
| APLNR | 1.6091854 | 0.03063994 | 2.4192508 | 0.01780099 | 0.54973449 | -3.1269775 | 2.36973177 |
| PRSS23 | 0.63242087 | 5.00692834 | 2.417491 | 0.01789108 | 0.54973449 | -3.1287746 | 2.36786461 |
| UNC79 | -0.4407964 | 5.86991347 | -2.4155453 | 0.01798997 | 0.54973449 | -3.1368068 | -2.3658244 |
| H2AC14 | -1.2561487 | -6.6666084 | -2.4126205 | 0.01800434 | 0.54973449 | -3.1362782 | -2.365529 |
| SRPRA | 0.2404047 | 6.66706417 | 2.41415883 | 0.01804401 | 0.54973449 | -3.1371292 | 2.36471372 |
| PNN-AS1 | 1.22837902 | -3.3782381 | 2.41178886 | 0.01807084 | 0.54973449 | -3.1540017 | 2.3641634 |
| INO80D-AS1 | 0.40768537 | 2.06976566 | 2.41337029 | 0.01808896 | 0.54973449 | -3.1373416 | 2.36379208 |
| ESPN | 1.38779622 | 4.85711673 | 2.41169543 | 0.01814129 | 0.54973449 | -3.1387209 | 2.36272169 |
| ANKRD13A | 0.20370081 | 5.77108528 | 2.41104505 | 0.01821064 | 0.54973449 | -3.1432916 | 2.36130708 |
| ALG1L2 | 0.62759022 | -1.6943889 | 2.40954971 | 0.01822524 | 0.54973449 | -3.1453907 | 2.3610099 |
| GSTA4 | -0.200394 | 6.66392714 | -2.4095293 | 0.01827164 | 0.54973449 | -3.1461003 | -2.3600669 |
| CRYZL2P | 0.96365733 | 0.7908473 | 2.40990781 | 0.01827813 | 0.54973449 | -3.141542 | 2.3599351 |
| TLR2 | 1.29698415 | -2.1392197 | 2.40792652 | 0.01834308 | 0.54973449 | -3.1466035 | 2.35861886 |
| CCP110 | -0.2062154 | 5.95695507 | -2.4072863 | 0.01838377 | 0.54973449 | -3.1511243 | -2.3577964 |
| LINC00663 | 0.2804618 | 2.23054493 | 2.40607819 | 0.01844947 | 0.54973449 | -3.1501491 | 2.35647177 |
| ELFN2 | -0.5117724 | 6.16534883 | -2.4056007 | 0.01845339 | 0.54973449 | -3.1555431 | -2.3563929 |
| SLC35D2 | 0.60708869 | 0.68119595 | 2.40438354 | 0.01848717 | 0.54973449 | -3.1506299 | 2.35571356 |
| HNRNPA1P22 | 1.44110057 | -6.2877399 | 2.40042558 | 0.0185341 | 0.54973449 | -3.153825 | 2.35477145 |
| FAM76A | 0.23753626 | 4.66193877 | 2.40280676 | 0.01859129 | 0.54973449 | -3.1565869 | 2.35362628 |
| MINPP1 | 0.20887489 | 4.80961899 | 2.40112793 | 0.01860838 | 0.54973449 | -3.1592215 | 2.35328472 |
| IRF6 | -1.3773376 | -2.5296003 | -2.4009683 | 0.01861747 | 0.54973449 | -3.1636936 | -2.3531031 |
| TEDC2-AS1 | -0.8689132 | -0.9717114 | -2.397451 | 0.01863176 | 0.54973449 | -3.1603983 | -2.3528177 |
| GREM1 | 2.53632183 | -0.0656707 | 2.39890153 | 0.01867646 | 0.54973449 | -3.1871651 | 2.35192665 |
| C1orf74 | 1.28330032 | -4.3464169 | 2.39711204 | 0.01870019 | 0.54973449 | -3.160709 | 2.35145416 |
| FOSL2 | 0.76899962 | 6.07696032 | 2.40113908 | 0.01871806 | 0.54973449 | -3.1629665 | 2.35109883 |
| TRIM74 | 0.82135281 | 0.50224091 | 2.40123386 | 0.01873662 | 0.54973449 | -3.1761713 | 2.35073012 |
| HSPA9P1 | -1.1346884 | -2.4747477 | -2.3938798 | 0.01876177 | 0.54973449 | -3.1666081 | -2.3502309 |
| UST | 0.51043148 | 5.3051737 | 2.39810148 | 0.0187728 | 0.54973449 | -3.1648677 | 2.3500121 |
| TMSB15A | -0.4498519 | 4.51399579 | -2.3980525 | 0.018818 | 0.54973449 | -3.1675913 | -2.3491168 |
| MAN2B1 | 0.46796511 | 6.2708474 | 2.39749859 | 0.01883805 | 0.54973449 | -3.1669259 | 2.34872038 |
| PARP11 | 0.17612669 | 4.69546102 | 2.39623277 | 0.01888263 | 0.54973449 | -3.1704314 | 2.34783992 |
| CLPTM1L | 0.17794749 | 6.59739537 | 2.39551191 | 0.01889074 | 0.54973449 | -3.1703435 | 2.34767997 |
| TNK2-AS1 | -0.9796421 | -2.2160671 | -2.3946312 | 0.01891794 | 0.54973449 | -3.1733974 | -2.347144 |
| LINC02234 | 1.09988098 | -1.2449972 | 2.39392655 | 0.01892127 | 0.54973449 | -3.1700931 | 2.34707848 |
| CCDC28A | 0.36310142 | 2.58471209 | 2.39570899 | 0.01892487 | 0.54973449 | -3.1707337 | 2.34700751 |
| CRACR2A | 0.98115028 | -0.1761363 | 2.39215813 | 0.0189709 | 0.54973449 | -3.1738632 | 2.34610238 |
| SMC2-DT | 0.88568478 | -1.5981911 | 2.39340869 | 0.01899112 | 0.54973449 | -3.1804688 | 2.34570531 |
| CBX3P2 | 0.39269217 | 2.00138114 | 2.39460893 | 0.01899117 | 0.54973449 | -3.1757936 | 2.34570426 |
| PLD5 | -0.4897429 | 4.30648519 | -2.3927657 | 0.01901279 | 0.54973449 | -3.1748541 | -2.3452801 |
| JCHAIN | 1.70975835 | -5.0394275 | 2.39017655 | 0.01902401 | 0.54973449 | -3.179295 | 2.34506017 |
| SNORD114-6 | 1.22738746 | -6.2431838 | 2.38850346 | 0.01912638 | 0.54973449 | -3.1791464 | 2.34305859 |
| DUSP15 | 0.28422267 | 3.79364113 | 2.38981974 | 0.0192024 | 0.54973449 | -3.18095 | 2.34157822 |
| COX19 | -0.1809734 | 4.97562336 | -2.3884567 | 0.01925291 | 0.54973449 | -3.1868318 | -2.3405976 |
| TMBIM4 | 0.30160195 | 4.36418874 | 2.38741403 | 0.01931989 | 0.54973449 | -3.1850881 | 2.33930052 |
| TXNRD2 | 0.27848665 | 3.14225248 | 2.38607834 | 0.01939502 | 0.54973449 | -3.1865964 | 2.33785027 |
| CEBPB | 0.66357051 | 3.70388546 | 2.38492727 | 0.01940823 | 0.54973449 | -3.1867262 | 2.33759566 |
| PIGB | 0.32115233 | 2.93388168 | 2.38544948 | 0.01941699 | 0.54973449 | -3.1885432 | 2.33742699 |
| ADGRB3 | -0.3387168 | 6.67326351 | -2.3854871 | 0.0194195 | 0.54973449 | -3.1894628 | -2.3373787 |
| RN7SL809P | -1.3775717 | -3.2799702 | -2.3815379 | 0.01944153 | 0.54973449 | -3.1957619 | -2.3369549 |
| MITA1 | 0.98780028 | -0.8432421 | 2.38411389 | 0.0194546 | 0.54973449 | -3.1873967 | 2.33670353 |
| BICRA-AS2 | -0.8460098 | -0.9738707 | -2.3799216 | 0.01946303 | 0.54973449 | -3.195904 | -2.3365416 |
| PHF11 | 0.70447336 | 2.94838064 | 2.38466153 | 0.01947235 | 0.54973449 | -3.19019 | 2.33636261 |
| IFITM2 | 1.43066096 | 3.38940204 | 2.38252098 | 0.01955109 | 0.54973449 | -3.1911111 | 2.33485326 |
| TRAPPC6A | 0.34355172 | 3.3700052 | 2.38380754 | 0.01955229 | 0.54973449 | -3.1909081 | 2.33483027 |
| APOBEC3B | 0.60136576 | -0.3434913 | 2.37747423 | 0.01972516 | 0.54973449 | -3.19933 | 2.33153513 |
| RAB28P5 | -0.573277 | -0.9720005 | -2.3748386 | 0.01973632 | 0.54973449 | -3.1998387 | -2.3313233 |
| BCO1 | 1.0214269 | -0.1099669 | 2.37793289 | 0.01976471 | 0.54973449 | -3.1987916 | 2.33078477 |
| DENND10 | 0.22886932 | 5.53500362 | 2.37657322 | 0.01976727 | 0.54973449 | -3.2032159 | 2.33073621 |
| SNORA11 | -0.8593651 | -1.6481967 | -2.3733954 | 0.01981021 | 0.54973449 | -3.2052406 | -2.3299233 |
| CNFN | 1.08315524 | -1.2292048 | 2.37477112 | 0.01984572 | 0.54973449 | -3.2082018 | 2.32925194 |
| SLC22A18AS | 1.00056548 | -1.8931629 | 2.37575617 | 0.01986542 | 0.54973449 | -3.2028271 | 2.3288801 |
| PXYLP1 | -0.3052548 | 6.53375827 | -2.3754013 | 0.01987357 | 0.54973449 | -3.2058217 | -2.3287262 |
| UTP15 | 0.23739033 | 4.48517562 | 2.37530587 | 0.01988879 | 0.54973449 | -3.2061826 | 2.32843927 |
| SLC6A11 | -0.8060314 | 4.46634057 | -2.3756389 | 0.0199162 | 0.54973449 | -3.2098268 | -2.3279228 |
| MIA2 | 0.17120697 | 4.413549 | 2.37430027 | 0.01993626 | 0.54973449 | -3.208731 | 2.32754525 |
| PRPSAP2 | -0.1908439 | 5.63073494 | -2.374904 | 0.01994377 | 0.54973449 | -3.2076021 | -2.327404 |
| APH1B | 0.25261848 | 4.73082432 | 2.37375964 | 0.01996851 | 0.54973449 | -3.2087508 | 2.32693901 |
| MT3 | -0.7011794 | 3.72769086 | -2.3737007 | 0.01998982 | 0.54973449 | -3.2115299 | -2.3265389 |
| CIDEA | 1.89312586 | -4.7430364 | 2.37084237 | 0.02001087 | 0.54973449 | -3.2138543 | 2.32614398 |
| LINC02940 | -1.3530827 | -3.5563269 | -2.3717452 | 0.02001725 | 0.54973449 | -3.2077846 | -2.3260244 |
| ADAMTS9 | 0.66805617 | 4.26481486 | 2.37214012 | 0.02003267 | 0.54973449 | -3.2108176 | 2.32573536 |
| HIP1R | -0.3675109 | 7.23752282 | -2.3733649 | 0.02003377 | 0.54973449 | -3.211281 | -2.3257149 |
| WASF3 | -0.2980913 | 6.23699178 | -2.3729217 | 0.02004508 | 0.54973449 | -3.2111217 | -2.325503 |
| HNRNPA3P5 | -0.694651 | -0.2566451 | -2.3712668 | 0.02004615 | 0.54973449 | -3.2121482 | -2.3254829 |
| SERPINB6 | 0.35951981 | 5.40098262 | 2.37218843 | 0.02009366 | 0.54973449 | -3.2111784 | 2.32459445 |
| TTC39A-AS1 | 1.6598181 | -5.3297422 | 2.36858441 | 0.02009579 | 0.54973449 | -3.2148451 | 2.32455462 |
| LOH12CR2 | 0.33664362 | 1.88974681 | 2.3693735 | 0.0200994 | 0.54973449 | -3.2137964 | 2.32448705 |
| LINC00987 | 0.63203643 | -0.7320065 | 2.36865192 | 0.02010234 | 0.54973449 | -3.2147149 | 2.3244323 |
| LINC03016 | 1.31935521 | -2.2601765 | 2.36819104 | 0.02010954 | 0.54973449 | -3.2143297 | 2.32429785 |
| IGDCC4 | 0.35602996 | 6.25919246 | 2.37128379 | 0.02012242 | 0.54973449 | -3.2126504 | 2.3240573 |
| CSNK1A1L | -1.7002448 | -4.7772864 | -2.3666572 | 0.02012494 | 0.54973449 | -3.2165868 | -2.3240103 |
| DYNLT3 | 0.40948039 | 3.62466414 | 2.37087227 | 0.02013631 | 0.54973449 | -3.2127421 | 2.3237983 |
| SEPTIN7P13 | 1.36344567 | -5.442097 | 2.36607318 | 0.02023296 | 0.54973449 | -3.2190917 | 2.32199953 |
| ARID4A | -0.2144795 | 6.04051662 | -2.3684496 | 0.02023406 | 0.54973449 | -3.2193623 | -2.321979 |
| ZC3H11A | 0.55140731 | -0.300103 | 2.36689203 | 0.02024987 | 0.54973449 | -3.219752 | 2.3216857 |
| TESC-AS1 | 1.55199092 | -5.560333 | 2.36451296 | 0.02031555 | 0.54973449 | -3.2211263 | 2.32046842 |
| UBE2O | -0.1519351 | 7.15189202 | -2.3666933 | 0.02037325 | 0.54973449 | -3.2229776 | -2.319402 |
| THORLNC | 0.81809503 | -1.7578464 | 2.36360998 | 0.02038122 | 0.54973449 | -3.2284123 | 2.31925491 |
| ELOVL3 | -1.627386 | -4.4913227 | -2.3657987 | 0.02038933 | 0.54973449 | -3.2223654 | -2.3191053 |
| SRL | -1.1092104 | -1.9524096 | -2.3640498 | 0.0203957 | 0.54973449 | -3.2233462 | -2.3189878 |
| FZD6 | 0.77803843 | 4.7366358 | 2.36549898 | 0.02042202 | 0.54973449 | -3.2238056 | 2.31850262 |
| CAPSL | -0.6893393 | 0.59260134 | -2.3656265 | 0.02042288 | 0.54973449 | -3.22743 | -2.3184868 |
| PPP2R1B | 0.33106679 | 4.79641169 | 2.36488022 | 0.02042312 | 0.54973449 | -3.2243531 | 2.31848242 |
| KCNK2 | 0.47309152 | 4.46502964 | 2.36495885 | 0.02042398 | 0.54973449 | -3.2267303 | 2.31846647 |
| SNORD114-3 | 1.22728341 | -6.319031 | 2.36318066 | 0.02042872 | 0.54973449 | -3.2260834 | 2.31837928 |
| AMD1P4 | 1.67270669 | -5.8206837 | 2.36372304 | 0.02043881 | 0.54973449 | -3.2251174 | 2.31819338 |
| LINC02389 | -1.1005211 | -3.2815198 | -2.3631847 | 0.02043939 | 0.54973449 | -3.2228038 | -2.3181827 |
| GLDN | 0.67982796 | 0.68110591 | 2.36618058 | 0.0204425 | 0.54973449 | -3.2202615 | 2.31812548 |
| IL1RAPL1 | -0.5979173 | 2.25176286 | -2.3632469 | 0.02046048 | 0.54973449 | -3.229406 | -2.3177948 |
| IGDCC3 | -0.5470336 | 4.74422811 | -2.3642215 | 0.02048008 | 0.54973449 | -3.2271144 | -2.3174344 |
| IER3 | 0.59387238 | 1.88049779 | 2.36435447 | 0.02048654 | 0.54973449 | -3.224165 | 2.31731569 |
| ZSWIM5 | -0.3478946 | 5.1808852 | -2.3637652 | 0.02051831 | 0.54973449 | -3.22982 | -2.3167325 |
| ZKSCAN8P1 | -0.6429491 | 1.02421681 | -2.3613534 | 0.0205243 | 0.54973449 | -3.2291503 | -2.3166226 |
| TXNRD3 | 0.32964006 | 3.28526096 | 2.36291878 | 0.0205347 | 0.54973449 | -3.2282621 | 2.31643193 |
| PABPC4L | 0.56318468 | 0.79265978 | 2.36264679 | 0.02054082 | 0.54973449 | -3.2298238 | 2.31631975 |
| CBX8 | -0.2238779 | 5.06117406 | -2.3630529 | 0.0205476 | 0.54973449 | -3.2302721 | -2.3161955 |
| PTP4A2 | 0.21329941 | 7.76673628 | 2.35972444 | 0.02057851 | 0.54973449 | -3.2308828 | 2.31562949 |
| ADPRH | 0.76965414 | 0.16389876 | 2.36247622 | 0.02059422 | 0.54973449 | -3.232954 | 2.31534205 |
| DIO3 | 1.12532421 | 5.90705122 | 2.36139909 | 0.02060531 | 0.54973449 | -3.2348181 | 2.31513933 |
| LINC02696 | -0.5885416 | 0.23567363 | -2.3597244 | 0.02062315 | 0.54973449 | -3.2310644 | -2.3148133 |
| SV2C-AS1 | 1.11861968 | -0.8901317 | 2.36005999 | 0.02064083 | 0.54973449 | -3.2310541 | 2.31449051 |
| CBX5P1 | -1.7054513 | -5.6313383 | -2.3579411 | 0.02065785 | 0.54973449 | -3.2325156 | -2.31418 |
| LINC00467 | 0.43698624 | 3.1422019 | 2.3604725 | 0.02068933 | 0.54973449 | -3.2308423 | 2.31360626 |
| CXCL9 | -1.343454 | -6.3469396 | -2.3669685 | 0.02069501 | 0.54973449 | -3.2187151 | -2.3135029 |
| ABCC4 | 0.6719771 | 4.12882231 | 2.35926682 | 0.02070461 | 0.54973449 | -3.23468 | 2.3133281 |
| CSMD2 | -0.5946637 | 5.62408907 | -2.3593145 | 0.02075286 | 0.54973449 | -3.2393149 | -2.3124506 |
| ABAT | -0.3830485 | 8.52241802 | -2.3591153 | 0.02075564 | 0.54973449 | -3.2351111 | -2.3124002 |
| RNA5SP435 | -1.4202472 | -4.48057 | -2.3556681 | 0.02076237 | 0.54973449 | -3.241392 | -2.312278 |
| MT1M | 1.18477113 | -1.3518066 | 2.35839098 | 0.02079054 | 0.54973449 | -3.2366119 | 2.31176668 |
| ADRA1A | -0.9300424 | 2.9267159 | -2.3568286 | 0.02084138 | 0.54973449 | -3.246265 | -2.3108457 |
| STK24 | 0.26981021 | 5.9438943 | 2.35498147 | 0.02085065 | 0.54973449 | -3.2399246 | 2.31067802 |
| KCNC3 | -0.389124 | 5.17839644 | -2.3552974 | 0.02096605 | 0.54973449 | -3.24751 | -2.3085953 |
| GLB1 | 0.36800671 | 5.73157207 | 2.35454827 | 0.0209722 | 0.54973449 | -3.2427803 | 2.30848457 |
| SOSTDC1 | -0.7375104 | 1.10445126 | -2.3543951 | 0.02098225 | 0.54973449 | -3.2544332 | -2.3083037 |
| SNX30-DT | 0.77938029 | -1.3959026 | 2.3542297 | 0.02098597 | 0.54973449 | -3.2451041 | 2.30823679 |
| DOCK7-DT | 0.42279306 | 1.88131077 | 2.35273825 | 0.02101053 | 0.54973449 | -3.2506783 | 2.30779527 |
| EDIL3 | 0.79653918 | 7.49689782 | 2.35252083 | 0.02105647 | 0.54973449 | -3.2459746 | 2.3069704 |
| TBC1D20 | 0.12172932 | 6.48713739 | 2.353323 | 0.02107179 | 0.54973449 | -3.2457663 | 2.30669564 |
| HNRNPA1P54 | -1.6616908 | -5.3753985 | -2.3525202 | 0.02108907 | 0.54973449 | -3.2444306 | -2.3063861 |
| TENT5A | 0.7054135 | 4.93485323 | 2.35170231 | 0.02111764 | 0.54973449 | -3.2477715 | 2.30587456 |
| AHR | 0.55817263 | 4.0613598 | 2.35216456 | 0.02112258 | 0.54973449 | -3.2458082 | 2.3057863 |
| RASSF8-AS1 | 0.28173118 | 4.87043818 | 2.35101714 | 0.02118004 | 0.54973449 | -3.2504091 | 2.30475961 |
| MIR7856 | -1.4241266 | -4.3747002 | -2.3484036 | 0.02121858 | 0.54973449 | -3.2525283 | -2.3040723 |
| CHST13 | -1.5439746 | -3.6592347 | -2.3488134 | 0.02123294 | 0.54973449 | -3.2544532 | -2.3038166 |
| ADAM32 | 0.78039487 | 1.51973656 | 2.34823016 | 0.02125532 | 0.54973449 | -3.2549196 | 2.30341824 |
| NRXN2 | -0.5018016 | 8.96962346 | -2.3496428 | 0.02125614 | 0.54973449 | -3.2520763 | -2.3034037 |
| CFAP107 | -0.8779058 | 1.13803196 | -2.3489418 | 0.02126807 | 0.54973449 | -3.2513933 | -2.3031915 |
| PARVA | 0.26987053 | 5.90386349 | 2.34953983 | 0.02128305 | 0.54973449 | -3.2519575 | 2.30292524 |
| FBXO4 | 0.47603624 | 1.99873735 | 2.34970737 | 0.02129734 | 0.54973449 | -3.2512664 | 2.30267141 |
| SIK2 | 0.46169116 | 7.30377211 | 2.34766203 | 0.02130466 | 0.54973449 | -3.2545841 | 2.30254148 |
| RPL24P8 | 0.83349998 | -1.0957399 | 2.34338858 | 0.02133882 | 0.54973449 | -3.2568324 | 2.30193538 |
| CNTNAP3 | 0.41250864 | 4.94342576 | 2.34763994 | 0.02135853 | 0.54973449 | -3.2561147 | 2.30158602 |
| C7 | 1.65561338 | -2.7017477 | 2.3468905 | 0.02137337 | 0.54973449 | -3.255575 | 2.30132316 |
| MIR4489 | -1.0592065 | -3.2338591 | -2.3490434 | 0.02144567 | 0.54973449 | -3.2514307 | -2.300045 |
| PPIAP53 | 0.98484521 | -1.506716 | 2.34453082 | 0.0214867 | 0.54973449 | -3.2645364 | 2.29932124 |
| F3 | -0.4215009 | 5.84856294 | -2.3444666 | 0.02153684 | 0.54973449 | -3.260811 | -2.2984386 |
| OR7C1 | -1.470639 | -4.7358704 | -2.3440903 | 0.02153912 | 0.54973449 | -3.2591954 | -2.2983984 |
| DUXAP7 | -1.7789588 | -4.8956512 | -2.3409195 | 0.02156715 | 0.54973449 | -3.2644444 | -2.2979058 |
| MIR4285 | -1.3246224 | -5.964975 | -2.3408903 | 0.02159742 | 0.54973449 | -3.2627074 | -2.2973744 |
| CEP78 | -0.2905881 | 6.09723955 | -2.3434709 | 0.02170151 | 0.54973449 | -3.2622418 | -2.2955519 |
| IL31RA | 1.02281457 | -2.0013909 | 2.33926383 | 0.02174563 | 0.54973449 | -3.2698978 | 2.29478188 |
| SSBP3P2 | -1.5349748 | -6.0249817 | -2.3381634 | 0.02176381 | 0.54973449 | -3.2688828 | -2.2944648 |
| CRTAP | 0.32730017 | 6.91568834 | 2.33954013 | 0.02180305 | 0.54973449 | -3.2694258 | 2.29378152 |
| LINC01885 | 1.70925614 | -5.765434 | 2.33691869 | 0.02180667 | 0.54973449 | -3.2725684 | 2.29371853 |
| PARN | 0.21617046 | 5.08908424 | 2.33919924 | 0.02182071 | 0.54973449 | -3.2697994 | 2.29347441 |
| FKBP14 | 0.26072589 | 4.66988342 | 2.33804399 | 0.02187944 | 0.54973449 | -3.2727011 | 2.29245442 |
| PRKCA | -0.4761557 | 6.75237822 | -2.336823 | 0.02189242 | 0.54973449 | -3.2746675 | -2.2922293 |
| RAP1BP1 | -1.2367917 | -4.6852931 | -2.3365127 | 0.02189399 | 0.54973449 | -3.2752619 | -2.292202 |
| RHBDD3 | 0.15425045 | 5.66012816 | 2.33726824 | 0.02190746 | 0.54973449 | -3.2743405 | 2.29196849 |
| OLIG2 | -1.9182114 | 0.38822887 | -2.3367131 | 0.02190805 | 0.54973449 | -3.2792164 | -2.2919583 |
| RIMBP3B | -1.453119 | -2.8447514 | -2.3360875 | 0.02191085 | 0.54973449 | -3.2785845 | -2.2919098 |
| HOMER1 | -0.3045374 | 5.55997414 | -2.3374268 | 0.02192154 | 0.54973449 | -3.2768853 | -2.2917247 |
| VWA1 | 0.43637306 | 4.83099471 | 2.33699167 | 0.02193218 | 0.54973449 | -3.2735936 | 2.29154034 |
| COL18A1-AS2 | 1.26269691 | -6.0723857 | 2.33428938 | 0.02195968 | 0.54973449 | -3.2780656 | 2.29106459 |
| BCKDHB | 0.76084504 | 6.42812068 | 2.33579257 | 0.02197426 | 0.54973449 | -3.275656 | 2.29081255 |
| NR4A3 | 1.15938075 | 4.72340962 | 2.33722612 | 0.02199702 | 0.54973449 | -3.2720878 | 2.29041931 |
| ZNF132-DT | -0.3317093 | 0.87491126 | -2.3357653 | 0.02202023 | 0.54973449 | -3.2771664 | -2.2900187 |
| MPV17L | 0.5454193 | 3.70018893 | 2.33371502 | 0.02208388 | 0.54973449 | -3.2799923 | 2.28892215 |
| OR6L2P | -1.3756078 | -3.6310157 | -2.3298601 | 0.02208554 | 0.54973449 | -3.2820782 | -2.2888935 |
| RNU7-171P | 1.23710967 | -4.3639562 | 2.32970004 | 0.02208989 | 0.54973449 | -3.2802426 | 2.28881866 |
| LINC00578 | -0.7435831 | -1.7689784 | -2.3364517 | 0.02209637 | 0.54973449 | -3.278474 | -2.2887073 |
| FBXO3 | -0.1856833 | 6.14359217 | -2.3322418 | 0.02213381 | 0.54973449 | -3.2819184 | -2.2880638 |
| LINC02765 | 1.57468107 | -3.8435016 | 2.3334145 | 0.02213553 | 0.54973449 | -3.2783457 | 2.28803413 |
| DNAJC10 | 0.20828042 | 7.51115089 | 2.33319483 | 0.0221385 | 0.54973449 | -3.2809475 | 2.28798326 |
| FOXR1 | 1.27195376 | -2.5764932 | 2.33497178 | 0.02215575 | 0.54973449 | -3.2837958 | 2.28768701 |
| SETP12 | -1.3736748 | -5.6151055 | -2.3303404 | 0.02219191 | 0.54973449 | -3.2839421 | -2.287067 |
| IFNGR2 | 0.30262493 | 4.95105489 | 2.32981595 | 0.0221977 | 0.54973449 | -3.2852014 | 2.28696789 |
| CYCSP24 | -1.4878479 | -5.2818553 | -2.3288363 | 0.02220133 | 0.54973449 | -3.2838853 | -2.2869057 |
| GALC | 0.21153347 | 6.20247579 | 2.33211249 | 0.02221603 | 0.54973449 | -3.2827513 | 2.28665386 |
| KRCC1 | 0.23487307 | 4.12238073 | 2.33110253 | 0.02226817 | 0.54973449 | -3.2835641 | 2.28576221 |
| GPBAR1 | 0.72103921 | -1.7469447 | 2.32909813 | 0.02227296 | 0.54973449 | -3.2861816 | 2.28568044 |
| SGPP2 | 0.60370633 | 4.38212493 | 2.32867063 | 0.02236309 | 0.54973449 | -3.2884773 | 2.2841437 |
| XPO1 | -0.1889579 | 7.80101479 | -2.3284843 | 0.02237332 | 0.54973449 | -3.2887141 | -2.2839696 |
| ACSS3 | 0.75008861 | 3.96063324 | 2.32902075 | 0.02238093 | 0.54973449 | -3.2874148 | 2.2838401 |
| FOXA1 | 1.24771232 | -0.086567 | 2.32796497 | 0.02238375 | 0.54973449 | -3.2935214 | 2.28379208 |
| MYL1 | -1.482531 | -6.2963614 | -2.3271826 | 0.02241645 | 0.54973449 | -3.2904338 | -2.2832364 |
| RFPL4B | -1.6545018 | -5.8376041 | -2.3237946 | 0.02241886 | 0.54973449 | -3.2908487 | -2.2831953 |
| LINC01937 | 1.45459042 | -4.8808885 | 2.32436405 | 0.02243401 | 0.54973449 | -3.2929114 | 2.28293812 |
| WASL-DT | 0.25431532 | 3.27893681 | 2.32608114 | 0.02244305 | 0.54973449 | -3.2915467 | 2.28278472 |
| NLRP3 | 1.07373794 | -2.7762376 | 2.32792279 | 0.02245406 | 0.54973449 | -3.2891372 | 2.28259791 |
| MIR5693 | 1.57876154 | -5.6444954 | 2.32410557 | 0.0224748 | 0.54973449 | -3.2959974 | 2.28224634 |
| ARL4A | 0.40888307 | 4.6179692 | 2.32686399 | 0.02248384 | 0.54973449 | -3.2913315 | 2.28209321 |
| CEND1 | -0.4585735 | 5.20285222 | -2.3263964 | 0.02252005 | 0.54973449 | -3.2945983 | -2.2814802 |
| EFEMP2 | 0.69997005 | 5.75600063 | 2.32697431 | 0.02252007 | 0.54973449 | -3.2919475 | 2.28147983 |
| GOLGA5 | 0.18459218 | 5.56451728 | 2.32644739 | 0.02252784 | 0.54973449 | -3.2919246 | 2.28134838 |
| ATAT1 | -0.2398384 | 7.6451961 | -2.3262889 | 0.02253875 | 0.54973449 | -3.2933771 | -2.2811639 |
| FAAP24 | 0.27750785 | 2.13017747 | 2.3243018 | 0.02254369 | 0.54973449 | -3.2954304 | 2.2810804 |
| HSPA5-DT | 1.54038209 | -4.7981031 | 2.32259851 | 0.02258555 | 0.54973449 | -3.2983873 | 2.28037336 |
| PTGR3 | -0.2653619 | 5.88245251 | -2.3241144 | 0.02260285 | 0.54973449 | -3.2962576 | -2.2800816 |
| FAM106A | -1.1615867 | -3.0596568 | -2.3226442 | 0.0226587 | 0.54973449 | -3.2983073 | -2.2791407 |
| EPHA3 | -0.6304482 | 4.20791634 | -2.3236885 | 0.02268207 | 0.54973449 | -3.2982869 | -2.2787477 |
| RNF149 | 0.2397653 | 5.19390265 | 2.32331815 | 0.02270254 | 0.54973449 | -3.2980762 | 2.27840362 |
| AGAP2 | -0.5064505 | 7.45193225 | -2.3233858 | 0.02270909 | 0.54973449 | -3.2988188 | -2.2782937 |
| WARS2-IT1 | 0.8513653 | -1.6383437 | 2.3215435 | 0.02272326 | 0.54973449 | -3.300284 | 2.27805576 |
| ATP1B2 | -0.1756842 | 8.60864215 | -2.3228368 | 0.02273967 | 0.54973449 | -3.2991583 | -2.2777804 |
| ANKRD13B | -0.2923833 | 6.78038299 | -2.3209496 | 0.02284082 | 0.54973449 | -3.3031983 | -2.2760866 |
| ADH1B | -1.7253601 | -3.7604641 | -2.3182406 | 0.02285289 | 0.54973449 | -3.3035988 | -2.2758851 |
| LINC02885 | -1.2969918 | -1.8577116 | -2.3190898 | 0.02292898 | 0.54973449 | -3.3128605 | -2.2746159 |
| EFCAB14P1 | 1.4152289 | -5.9499484 | 2.31601511 | 0.02293738 | 0.54973449 | -3.3085018 | 2.27447599 |
| C12orf75 | -0.4011649 | 4.13120851 | -2.3183388 | 0.02295748 | 0.54973449 | -3.3070511 | -2.2741415 |
| RNF19A | 0.39467834 | 6.77380448 | 2.3189658 | 0.02295817 | 0.54973449 | -3.3057493 | 2.27412997 |
| APH1A | 0.23691042 | 7.31924525 | 2.31871962 | 0.02297514 | 0.54973449 | -3.306065 | 2.27384775 |
| LARP4 | 0.13066493 | 5.79813269 | 2.31828393 | 0.02297583 | 0.54973449 | -3.3070281 | 2.27383628 |
| LINC02925 | 0.91990637 | 1.42672021 | 2.31802509 | 0.02297589 | 0.54973449 | -3.3066757 | 2.27383529 |
| CCDC13 | 0.55533068 | 0.4654163 | 2.31811843 | 0.02298535 | 0.54973449 | -3.3067887 | 2.27367806 |
| RNU6-204P | 1.37634563 | -5.8750905 | 2.31542766 | 0.02299954 | 0.54973449 | -3.3096161 | 2.27344226 |
| RASSF2 | -0.3839591 | 6.99632943 | -2.3172064 | 0.02303033 | 0.54973449 | -3.3085966 | -2.2729311 |
| BAALC-AS1 | 0.65577659 | 3.04711206 | 2.31737444 | 0.02303777 | 0.54973449 | -3.3082798 | 2.27280778 |
| THA1P | 1.03557312 | -0.7351414 | 2.31953221 | 0.02305047 | 0.54973449 | -3.2988342 | 2.27259705 |
| CPVL | 0.80456127 | 4.13780395 | 2.31708254 | 0.02305956 | 0.54973449 | -3.3081865 | 2.27244638 |
| HNRNPA3P6 | -0.4278791 | -0.2718623 | -2.3144168 | 0.02306703 | 0.54973449 | -3.3124042 | -2.2723226 |
| NUDT9P1 | 0.89779652 | -0.477069 | 2.31551969 | 0.02307856 | 0.54973449 | -3.3101757 | 2.27213165 |
| NLGN3 | -0.3192839 | 7.31999815 | -2.3159908 | 0.0230871 | 0.54973449 | -3.3101529 | -2.2719902 |
| BAHCC1 | -0.3373594 | 7.26756027 | -2.3150433 | 0.02308726 | 0.54973449 | -3.3121721 | -2.2719876 |
| MIR5695 | -1.6541185 | -5.2513918 | -2.315421 | 0.0231077 | 0.54973449 | -3.3096286 | -2.2716494 |
| ARRDC3-AS1 | 0.47098067 | 0.71663665 | 2.31646769 | 0.02310871 | 0.54973449 | -3.3110031 | 2.27163266 |
| DOCK6-AS1 | -1.1635089 | -2.952778 | -2.312754 | 0.02313208 | 0.54973449 | -3.3138798 | -2.2712462 |
| RAB43 | 0.40109272 | 0.97190015 | 2.31541035 | 0.02314782 | 0.54973449 | -3.3116287 | 2.27098614 |
| TSNAX | 0.23233025 | 5.28696428 | 2.31375867 | 0.02317778 | 0.54973449 | -3.3139154 | 2.27049145 |
| BTD | 0.33086735 | 5.23914823 | 2.31495704 | 0.02317935 | 0.54973449 | -3.3126906 | 2.27046554 |
| RAB29 | 0.39218822 | 4.71427278 | 2.31387564 | 0.02324829 | 0.55078476 | -3.313838 | 2.26932961 |
| ZNF197 | -0.1793873 | 5.86182644 | -2.3127452 | 0.02329477 | 0.55109553 | -3.3171384 | -2.2685653 |
| BCCIP | -0.2365728 | 5.35254692 | -2.3125238 | 0.02331074 | 0.55109553 | -3.3167262 | -2.2683031 |
| CPPED1 | 0.45774319 | 3.62117004 | 2.31111776 | 0.02335254 | 0.55150001 | -3.3185917 | 2.2676174 |
| UROD | 0.21589951 | 6.08874951 | 2.31061443 | 0.02342767 | 0.55247947 | -3.3201803 | 2.26638741 |
| RPL22P19 | -1.5577388 | -5.543039 | -2.3081587 | 0.02345446 | 0.55247947 | -3.321583 | -2.2659498 |
| CACNA1H | -0.3728338 | 6.93543622 | -2.3090619 | 0.02352564 | 0.55247947 | -3.3244393 | -2.2647888 |
| OCA2 | 0.87622942 | 1.27880613 | 2.30811904 | 0.02354904 | 0.55247947 | -3.3268333 | 2.26440795 |
| ERRFI1 | -0.4102549 | 5.13121143 | -2.3042367 | 0.02369621 | 0.55247947 | -3.329073 | -2.2620193 |
| FSCN2 | 0.44026151 | 1.95684021 | 2.30835691 | 0.0237261 | 0.55247947 | -3.3276065 | 2.2615358 |
| RPS12P31 | -1.5516096 | -4.5993834 | -2.3032058 | 0.02374104 | 0.55247947 | -3.3333245 | -2.2612942 |
| PLD3 | 0.35278314 | 9.17978665 | 2.30410591 | 0.02375311 | 0.55247947 | -3.3312498 | 2.26109926 |
| MXRA5 | 1.14684246 | 3.28534311 | 2.30309369 | 0.02379255 | 0.55247947 | -3.3323124 | 2.26046274 |
| CREG1 | 0.22151746 | 5.63293746 | 2.30361085 | 0.02382051 | 0.55247947 | -3.3325356 | 2.26001196 |
| MANSC4 | 1.13490483 | -1.8302488 | 2.30042026 | 0.02384581 | 0.55247947 | -3.3359969 | 2.25960453 |
| LRRC37A | -0.3094772 | 1.68975659 | -2.3023817 | 0.02385079 | 0.55247947 | -3.3362658 | -2.2595243 |
| HNRNPA3P4 | -1.4603311 | -5.8283677 | -2.3005653 | 0.02387751 | 0.55247947 | -3.3350274 | -2.2590945 |
| LINC02288 | 1.18048337 | -2.142948 | 2.3006686 | 0.02388241 | 0.55247947 | -3.3375522 | 2.25901563 |
| PPM1E | -0.3974959 | 6.56541668 | -2.3031496 | 0.02389115 | 0.55247947 | -3.332974 | -2.2588752 |
| CHD3 | -0.2638538 | 9.18422153 | -2.3020348 | 0.02394146 | 0.55247947 | -3.335484 | -2.2580674 |
| PPM1L | -0.4165007 | 6.86141309 | -2.3033018 | 0.02395653 | 0.55247947 | -3.332146 | -2.2578257 |
| ADAM17 | 0.22042733 | 6.1881302 | 2.30159158 | 0.02395777 | 0.55247947 | -3.3358915 | 2.25780572 |
| LINC02076 | -0.6771128 | -0.6152055 | -2.2998616 | 0.02397554 | 0.55247947 | -3.33924 | -2.2575211 |
| CHASERR | -0.2363842 | 7.09580779 | -2.2996656 | 0.0240177 | 0.55247947 | -3.3389454 | -2.2568459 |
| LINC02256 | 0.47940907 | -1.1419117 | 2.29822078 | 0.0240527 | 0.55247947 | -3.3432559 | 2.25628642 |
| ABCA1 | 0.39086622 | 6.76233145 | 2.29990413 | 0.02405642 | 0.55247947 | -3.3387352 | 2.25622699 |
| HNRNPA1P8 | -1.2700691 | -2.4216135 | -2.2948113 | 0.024083 | 0.55247947 | -3.3422764 | -2.2558026 |
| RASD1 | 0.82962948 | 4.5114925 | 2.29900744 | 0.02409807 | 0.55247947 | -3.340366 | 2.25556213 |
| FCHO1 | -0.3056507 | 5.25716182 | -2.2994362 | 0.02409811 | 0.55247947 | -3.3423986 | -2.2555615 |
| PDE4D | 0.45640675 | 7.24031511 | 2.29876159 | 0.02409872 | 0.55247947 | -3.3399292 | 2.25555173 |
| SUMF1 | 0.48811252 | 4.78924783 | 2.29844748 | 0.02414686 | 0.55247947 | -3.3409876 | 2.2547844 |
| BMP8B | -0.6375564 | 1.20818044 | -2.2987281 | 0.02415296 | 0.55247947 | -3.3428876 | -2.2546873 |
| MARCHF10 | -0.684553 | -0.6565923 | -2.2982392 | 0.02417423 | 0.55247947 | -3.3412865 | -2.2543488 |
| ADAM22 | -0.4555062 | 6.46264209 | -2.2976462 | 0.02419534 | 0.55247947 | -3.3409573 | -2.2540132 |
| HNRNPA3 | -0.23224 | 7.50231704 | -2.2970958 | 0.02420328 | 0.55247947 | -3.3436173 | -2.253887 |
| ADCYAP1R1 | -0.565015 | 7.51680523 | -2.2967724 | 0.02422946 | 0.55247947 | -3.344314 | -2.253471 |
| LTV1 | 0.22335516 | 3.33686825 | 2.29548558 | 0.02426782 | 0.55247947 | -3.3467104 | 2.25286252 |
| IL12RB2 | -0.7191506 | -1.1745395 | -2.2961213 | 0.02427193 | 0.55247947 | -3.3472562 | -2.2527973 |
| MKX | 0.69059454 | 3.20047368 | 2.29578047 | 0.02427847 | 0.55247947 | -3.3459083 | 2.25269373 |
| GNS | 0.38644855 | 7.99883841 | 2.29575025 | 0.02429109 | 0.55247947 | -3.3459315 | 2.25249371 |
| ARHGAP11B-DT | 0.48224705 | -1.2901852 | 2.29615891 | 0.024309 | 0.55247947 | -3.3442635 | 2.2522101 |
| RALBP1 | 0.20733465 | 6.00264348 | 2.29355273 | 0.02438025 | 0.55353582 | -3.3491417 | 2.25108343 |
| TMEM62 | 0.2460286 | 4.05455559 | 2.29211189 | 0.02446806 | 0.55496535 | -3.3521665 | 2.24969902 |
| BSG-AS1 | 0.63365895 | -0.0361404 | 2.29100223 | 0.02454334 | 0.55610821 | -3.3539873 | 2.24851551 |
| PLBD2 | 0.30732901 | 6.70204316 | 2.28899894 | 0.0246118 | 0.55709443 | -3.3569422 | 2.24744191 |
| SGSH | 0.37296493 | 5.49632099 | 2.29007486 | 0.02464873 | 0.55736571 | -3.3558003 | 2.24686381 |
| PEAK1 | -0.3444491 | 5.8708952 | -2.2875201 | 0.02470748 | 0.55782121 | -3.3586148 | -2.2459458 |
| GLIDR | 0.20608902 | 2.65583954 | 2.28866481 | 0.02471881 | 0.55782121 | -3.3593315 | 2.24576892 |
| GRM3-AS1 | -0.5229234 | 0.07995774 | -2.285397 | 0.02479262 | 0.5585288 | -3.3629147 | -2.2446188 |
| CCDC25 | 0.20805014 | 5.64176962 | 2.28738992 | 0.02482208 | 0.5585288 | -3.3603302 | 2.24416051 |
| LINC03072 | 0.20896807 | 5.48817133 | 2.28662135 | 0.02484908 | 0.5585288 | -3.3615328 | 2.24374091 |
| ALG2 | 0.22336207 | 4.65143247 | 2.28642609 | 0.02487 | 0.5585288 | -3.3622427 | 2.243416 |
| CENPVL2 | 1.30167677 | -4.8877517 | 2.28294271 | 0.0249129 | 0.5585288 | -3.3653155 | 2.2427506 |
| HM13-AS1 | 1.28388606 | -3.0840262 | 2.28184016 | 0.02491392 | 0.5585288 | -3.3653596 | 2.24273485 |
| TMEM79 | 0.26052077 | 2.51563776 | 2.28478777 | 0.02492517 | 0.5585288 | -3.3647039 | 2.24256051 |
| MIR649 | 1.45876886 | -5.7890432 | 2.28191529 | 0.02497953 | 0.55918607 | -3.3684072 | 2.24171913 |
| FRMPD3 | -0.3698447 | 4.84011333 | -2.2815889 | 0.0251245 | 0.5618683 | -3.3710625 | -2.2394831 |
| ANXA9 | 0.302345 | 3.07791882 | 2.28174235 | 0.02515109 | 0.56190044 | -3.3700368 | 2.23907422 |
| NGEF | -0.7127636 | 5.06707177 | -2.2803981 | 0.02522014 | 0.56247924 | -3.3742094 | -2.238014 |
| PPP4R2P1 | -1.05319 | -2.1840758 | -2.2761001 | 0.02524485 | 0.56247924 | -3.3747183 | -2.2376352 |
| BEGAIN | 0.47922929 | 5.83154139 | 2.27966476 | 0.02525253 | 0.56247924 | -3.3744493 | 2.23751767 |
| RBM47 | 0.81428002 | 2.7620416 | 2.27988318 | 0.02530103 | 0.56248679 | -3.3728041 | 2.23677525 |
| FAM53A | 0.54476893 | 0.08052666 | 2.27631088 | 0.02533647 | 0.56248679 | -3.3785546 | 2.23623371 |
| NRSN2-AS1 | -0.2409635 | 3.617381 | -2.2784396 | 0.02535518 | 0.56248679 | -3.3755129 | -2.2359479 |
| LINC00938 | -0.1845235 | 5.3727835 | -2.2782301 | 0.0253763 | 0.56248679 | -3.3765531 | -2.2356257 |
| NPNT | -0.8192941 | 4.17927561 | -2.2763977 | 0.02539417 | 0.56248679 | -3.3788622 | -2.2353532 |
| PIK3CD-AS2 | 0.73481093 | -0.3677497 | 2.27747267 | 0.02541422 | 0.56248679 | -3.3773896 | 2.23504763 |
| ECHDC3 | 0.74597417 | 0.61509995 | 2.27615393 | 0.02545513 | 0.56248679 | -3.3794544 | 2.23442492 |
| CERS2 | 0.31217838 | 5.80800594 | 2.27612776 | 0.02551428 | 0.56248679 | -3.3797466 | 2.23352589 |
| MIR589 | -1.4263406 | -5.0681376 | -2.2740439 | 0.02553992 | 0.56248679 | -3.3819566 | -2.2331368 |
| PCGF6 | 0.23054726 | 3.55853282 | 2.27491223 | 0.02555417 | 0.56248679 | -3.3819021 | 2.23292082 |
| DUS4L-BCAP29 | 0.26859236 | 2.97260572 | 2.27510833 | 0.02559155 | 0.56248679 | -3.3814489 | 2.2323544 |
| ELMOD1 | 0.49041982 | 6.36447992 | 2.27396415 | 0.02559646 | 0.56248679 | -3.3834745 | 2.23228006 |
| R3HDM1 | -0.2894368 | 6.76130095 | -2.274522 | 0.0256223 | 0.56248679 | -3.3827441 | -2.231889 |
| NOD1 | 0.39137999 | 2.47907834 | 2.27374289 | 0.0256326 | 0.56248679 | -3.383723 | 2.23173325 |
| PSMB8 | 0.85217422 | 2.56137841 | 2.27518056 | 0.02564519 | 0.56248679 | -3.3815528 | 2.23154284 |
| LPAL2 | 0.75263327 | -1.7784595 | 2.27231815 | 0.02567048 | 0.56248679 | -3.3857816 | 2.23116084 |
| PSMC1P5 | -1.5994356 | -5.8483798 | -2.2706647 | 0.02570624 | 0.56248679 | -3.3871398 | -2.230621 |
| FMO3 | -1.1844311 | -6.0154981 | -2.2721949 | 0.025711 | 0.56248679 | -3.3861654 | -2.2305493 |
| GPR12 | -0.3485543 | 4.91148253 | -2.2716844 | 0.02580509 | 0.56248679 | -3.3898767 | -2.2291325 |
| BHLHE41 | 0.75570283 | 5.79582182 | 2.2703598 | 0.02587926 | 0.56248679 | -3.3897228 | 2.22801881 |
| NPHP4 | 0.27726471 | 5.94219587 | 2.26939709 | 0.02588736 | 0.56248679 | -3.3910427 | 2.22789735 |
| NCOA3 | -0.2275723 | 5.87319587 | -2.2698462 | 0.02588836 | 0.56248679 | -3.3905745 | -2.2278823 |
| ARK2C | -0.2827161 | 6.86089601 | -2.2699487 | 0.02590979 | 0.56248679 | -3.3908612 | -2.2275612 |
| BRSK1 | -0.2699194 | 7.73534693 | -2.269785 | 0.02592519 | 0.56248679 | -3.3908545 | -2.2273306 |
| RNU4-29P | 1.45244248 | -4.8249314 | 2.26806311 | 0.02593453 | 0.56248679 | -3.3942873 | 2.22719066 |
| LRCH3 | 0.3510228 | 6.37021832 | 2.26921479 | 0.02594678 | 0.56248679 | -3.3915378 | 2.22700736 |
| LAPTM4A | 0.30720961 | 7.16114119 | 2.26915326 | 0.02594684 | 0.56248679 | -3.3917036 | 2.22700644 |
| MORF4 | -1.0290812 | -1.2836808 | -2.2656204 | 0.02596697 | 0.56248679 | -3.394646 | -2.2267054 |
| CCDC144CP | -0.5893879 | 1.88282063 | -2.2674464 | 0.02599162 | 0.56248679 | -3.3949335 | -2.2263369 |
| TMEM145 | -0.4167297 | 6.00708658 | -2.2683741 | 0.02600818 | 0.56248679 | -3.3941327 | -2.2260895 |
| GRHL1 | -0.5409091 | 2.4569912 | -2.2671965 | 0.02608448 | 0.56342598 | -3.3948985 | -2.2249517 |
| FER1L6-AS2 | 1.04554047 | -3.38273 | 2.2653737 | 0.02610205 | 0.56342598 | -3.3942826 | 2.2246901 |
| MGMT | 0.43400587 | 2.67266504 | 2.26465144 | 0.02615197 | 0.56395854 | -3.3991667 | 2.22394767 |
| DBI | -0.3494411 | 6.29669353 | -2.2645893 | 0.02622564 | 0.56469322 | -3.399461 | -2.2228541 |
| VPREB3 | 1.18519673 | -2.9762922 | 2.26053131 | 0.02623659 | 0.56469322 | -3.4015124 | 2.22269185 |
| ATP6V0A4 | -1.2420778 | -2.9913434 | -2.2619963 | 0.02628856 | 0.56526737 | -3.4031462 | -2.2219222 |
| CCT6B | 0.4184128 | 2.83536858 | 2.26414495 | 0.02635741 | 0.5660954 | -3.4003991 | 2.22090481 |
| PRG4 | -0.917392 | -2.3902667 | -2.2618278 | 0.0263913 | 0.5660954 | -3.4039425 | -2.2204048 |
| HSH2D | 1.40593029 | -6.2042086 | 2.25789713 | 0.02640906 | 0.5660954 | -3.4067469 | 2.22014297 |
| GALNT16 | -0.2587343 | 5.76626749 | -2.2597605 | 0.02646767 | 0.5660954 | -3.4074925 | -2.2192802 |
| NAGA | 0.33089204 | 4.87808974 | 2.26008382 | 0.0265166 | 0.5660954 | -3.4072371 | 2.21856107 |
| ELMOD2 | 0.20751789 | 5.52623194 | 2.25938882 | 0.02651814 | 0.5660954 | -3.4081519 | 2.21853853 |
| NBPF1 | -0.2464777 | 5.93590271 | -2.2604107 | 0.02652593 | 0.5660954 | -3.4067585 | -2.2184241 |
| GABRQ | 0.95200878 | 4.74997713 | 2.25838226 | 0.02655796 | 0.5660954 | -3.4107601 | 2.21795418 |
| GDI1 | -0.1518824 | 9.65997044 | -2.259512 | 0.02657117 | 0.5660954 | -3.4082242 | -2.2177605 |
| PPID | -0.2327871 | 4.24770001 | -2.2583849 | 0.02658046 | 0.5660954 | -3.4098322 | -2.2176243 |
| RN7SL538P | 1.45400391 | -5.9679876 | 2.25497856 | 0.02671564 | 0.56843256 | -3.4117681 | 2.21564764 |
| TSPAN7 | -0.3180627 | 6.69586358 | -2.2563936 | 0.02676257 | 0.56872918 | -3.4135909 | -2.2149635 |
| AIRIM | 0.17416813 | 4.7174885 | 2.25587259 | 0.02683439 | 0.56872918 | -3.4142776 | 2.21391845 |
| KLRC3 | -1.2409277 | -6.6732162 | -2.2535016 | 0.02683652 | 0.56872918 | -3.4173113 | -2.2138876 |
| LINC02997 | -1.1325545 | -3.0408539 | -2.253784 | 0.0268556 | 0.56872918 | -3.4194829 | -2.2136102 |
| RNU6-593P | 1.36710401 | -5.6588152 | 2.25100553 | 0.02693803 | 0.56872918 | -3.4192365 | 2.21241456 |
| C1orf43 | 0.22552818 | 7.22879712 | 2.25357057 | 0.02695727 | 0.56872918 | -3.418032 | 2.21213594 |
| CNTNAP2 | -0.5499042 | 7.86675581 | -2.2524449 | 0.02700979 | 0.56872918 | -3.4202311 | -2.2113763 |
| ODF2-AS1 | -1.1149349 | -3.2993407 | -2.25024 | 0.02703166 | 0.56872918 | -3.4235836 | -2.2110604 |
| ITK | 1.56785478 | -1.616111 | 2.25071038 | 0.02704907 | 0.56872918 | -3.4228399 | 2.21080895 |
| WNT7B | -0.7468147 | 4.58894236 | -2.2522901 | 0.02705753 | 0.56872918 | -3.4206293 | -2.2106869 |
| MAPK6P3 | -1.3497634 | -3.828636 | -2.248453 | 0.0271471 | 0.56872918 | -3.426012 | -2.2093961 |
| ACTMAP | 0.17133915 | 4.17026233 | 2.25006175 | 0.02719203 | 0.56872918 | -3.4243523 | 2.20875003 |
| SCPEP1 | 0.62327172 | 4.51438045 | 2.2494941 | 0.02720617 | 0.56872918 | -3.425191 | 2.20854698 |
| TOM1 | 0.24352617 | 5.42334965 | 2.24935415 | 0.02720714 | 0.56872918 | -3.4254713 | 2.20853305 |
| KCND3 | -0.5303265 | 6.01585518 | -2.2494079 | 0.02723135 | 0.56872918 | -3.4252708 | -2.2081854 |
| GRIK2 | -0.2908876 | 5.7984472 | -2.2489764 | 0.0272597 | 0.56872918 | -3.426129 | -2.2077787 |
| RN7SL487P | 1.59426076 | -4.2229325 | 2.24591029 | 0.02728929 | 0.56872918 | -3.4290208 | 2.20735467 |
| IAH1 | 0.28676793 | 3.99440041 | 2.24888501 | 0.02730231 | 0.56872918 | -3.4262898 | 2.20716828 |
| HLA-DQB1 | 1.35383947 | -1.4753899 | 2.24835287 | 0.02733615 | 0.56872918 | -3.4268934 | 2.20668391 |
| ADGRF2P | -1.4981494 | -4.9974546 | -2.2462277 | 0.02735377 | 0.56872918 | -3.4303862 | -2.206432 |
| C2CD5 | -0.1805255 | 6.28598167 | -2.2474569 | 0.02737078 | 0.56872918 | -3.4286984 | -2.2061888 |
| DSP | 1.48403964 | -2.386311 | 2.24717159 | 0.02738782 | 0.56872918 | -3.4305075 | 2.20594549 |
| MNX1 | -2.0704428 | -4.0212162 | -2.2454418 | 0.02739966 | 0.56872918 | -3.4291972 | -2.2057764 |
| ZSCAN31 | 0.23234564 | 2.94138356 | 2.24623007 | 0.02743864 | 0.56872918 | -3.4312537 | 2.20522034 |
| AIMP2 | 0.23387089 | 3.9175104 | 2.24446406 | 0.02745546 | 0.56872918 | -3.4332546 | 2.20498054 |
| CASTOR1 | -0.7148729 | -1.1208029 | -2.2453715 | 0.02749482 | 0.56872918 | -3.4329016 | -2.2044201 |
| MPHOSPH8 | -0.1424004 | 6.23552607 | -2.2442362 | 0.02758864 | 0.56872918 | -3.4339426 | -2.2030867 |
| PLAAT5 | 0.87214022 | -0.5833428 | 2.24404673 | 0.02758919 | 0.56872918 | -3.4348656 | 2.20307887 |
| SPATA3 | -1.3878746 | -5.8933196 | -2.2416028 | 0.02763725 | 0.56872918 | -3.4378835 | -2.2023973 |
| LIPE | -0.2681741 | 3.79553528 | -2.2432349 | 0.02764539 | 0.56872918 | -3.4358842 | -2.202282 |
| WASHC2C | 0.11166096 | 6.31381136 | 2.24198843 | 0.02764897 | 0.56872918 | -3.4376844 | 2.20223138 |
| DENND2C | 0.39665447 | 1.55191351 | 2.24133678 | 0.02765124 | 0.56872918 | -3.4391508 | 2.20219915 |
| NDUFA3P4 | -1.3272513 | -3.7417162 | -2.241315 | 0.0276697 | 0.56872918 | -3.4381873 | -2.2019378 |
| FGF14-AS2 | 0.41101027 | 1.182377 | 2.24255128 | 0.02768439 | 0.56872918 | -3.4380304 | 2.20172988 |
| CTDP1-DT | -1.5367338 | -4.6651495 | -2.240143 | 0.02769267 | 0.56872918 | -3.4388369 | -2.2016128 |
| OSBPL2 | -0.1023617 | 6.36670923 | -2.2428426 | 0.027694 | 0.56872918 | -3.4363695 | -2.2015941 |
| LINC01978 | 0.67202466 | -1.5734623 | 2.24326126 | 0.02770848 | 0.56872918 | -3.4368393 | 2.20138931 |
| QPRT | 0.46907153 | 5.1794524 | 2.24216941 | 0.02772765 | 0.56872918 | -3.4379621 | 2.20111828 |
| EMILIN2 | 0.50194266 | 0.46024688 | 2.24157234 | 0.0277537 | 0.56872918 | -3.4387145 | 2.20075035 |
| TMEM139 | -0.6894271 | -1.8641165 | -2.2405088 | 0.02776308 | 0.56872918 | -3.4393958 | -2.2006179 |
| FAM66B | 0.45985127 | 1.58497159 | 2.24118577 | 0.02780766 | 0.56872918 | -3.4393158 | 2.19998923 |
| APOBEC3C | 0.84924309 | 2.26931085 | 2.24059899 | 0.027818 | 0.56872918 | -3.4411832 | 2.19984347 |
| SLC14A2 | 0.44789993 | 0.06890505 | 2.23924121 | 0.02789886 | 0.56872918 | -3.4426439 | 2.19870557 |
| ZNF235 | -0.197363 | 2.96199505 | -2.2372443 | 0.02790258 | 0.56872918 | -3.4449137 | -2.1986534 |
| MREG | 0.61456118 | 3.94154413 | 2.23874524 | 0.02792874 | 0.56872918 | -3.4436253 | 2.1982859 |
| MEPE | -1.5290967 | -5.9336995 | -2.236789 | 0.02795576 | 0.56872918 | -3.4455079 | -2.1979066 |
| RN7SL674P | 1.02689792 | -2.1645366 | 2.23636173 | 0.02799597 | 0.56872918 | -3.4466962 | 2.19734285 |
| PCGF5 | 0.60452547 | 6.11754396 | 2.23752307 | 0.02801732 | 0.56872918 | -3.4455985 | 2.19704379 |
| RNU6-1053P | 0.85031974 | -1.933803 | 2.23434994 | 0.02804539 | 0.56872918 | -3.4500741 | 2.19665086 |
| CHRFAM7A | 0.42704938 | 0.85379673 | 2.2371626 | 0.02806111 | 0.56872918 | -3.4459158 | 2.19643099 |
| ADGRB1 | -0.5595135 | 6.77100315 | -2.2365347 | 0.02807619 | 0.56872918 | -3.4470527 | -2.1962201 |
| HECW1-IT1 | -1.7035552 | -5.1818721 | -2.2346533 | 0.02807722 | 0.56872918 | -3.4487232 | -2.1962058 |
| SLCO1B1 | -1.5891129 | -4.9485718 | -2.2341709 | 0.02810286 | 0.56872918 | -3.4529347 | -2.1958475 |
| CT45A11P | -1.7321829 | -4.9183646 | -2.2323407 | 0.02810425 | 0.56872918 | -3.449677 | -2.1958281 |
| SH2B2 | -0.250018 | 4.53886322 | -2.2358014 | 0.02816137 | 0.56886123 | -3.4493544 | -2.195031 |
| NLGN1-AS1 | 1.46430681 | -6.240615 | 2.23248961 | 0.0281617 | 0.56886123 | -3.4521002 | 2.19502647 |
| AQP5 | 1.23124568 | 0.21408768 | 2.23494561 | 0.02819256 | 0.5689702 | -3.4512687 | 2.19459641 |
| WDR4 | -0.1667532 | 3.48652627 | -2.2346441 | 0.02822271 | 0.56906451 | -3.4506538 | -2.1941767 |
| RELA | 0.15011782 | 6.36560002 | 2.23372434 | 0.02827963 | 0.56923836 | -3.4518774 | 2.19338536 |
| SMILR | 1.62413098 | -5.4645959 | 2.2334793 | 0.02829581 | 0.56923836 | -3.4567912 | 2.19316054 |
| TSPO | 0.4571432 | 4.53609261 | 2.23300765 | 0.02831797 | 0.56923836 | -3.4533641 | 2.19285302 |
| RBBP7 | 0.14606408 | 7.22385556 | 2.23319621 | 0.02833325 | 0.56923836 | -3.4527934 | 2.19264112 |
| CYP2U1 | 0.2580174 | 5.05812934 | 2.23250404 | 0.02837248 | 0.56925668 | -3.4539613 | 2.19209734 |
| ARHGAP23P1 | 1.65915044 | -5.4063876 | 2.22927522 | 0.02838724 | 0.56925668 | -3.456896 | 2.19189289 |
| QARS1 | 0.21943086 | 7.27609944 | 2.23017349 | 0.02855032 | 0.56925668 | -3.4574954 | 2.18964044 |
| ZNF277-AS1 | -0.5574322 | -1.0396673 | -2.226874 | 0.02857592 | 0.56925668 | -3.4625508 | -2.1892879 |
| LINC02392 | 1.79310771 | -5.4465007 | 2.22670448 | 0.02858967 | 0.56925668 | -3.4616856 | 2.1890986 |
| FAM180B | 0.53767942 | -0.0531169 | 2.22932009 | 0.02859678 | 0.56925668 | -3.4600279 | 2.18900085 |
| CXCL16 | 0.54715803 | 3.84537959 | 2.22969252 | 0.02859713 | 0.56925668 | -3.4589271 | 2.18899602 |
| TCEAL9 | 0.35716004 | 5.79325382 | 2.22959792 | 0.02860621 | 0.56925668 | -3.4589944 | 2.18887115 |
| PAK5 | -0.3473899 | 5.18243516 | -2.2279599 | 0.02870432 | 0.56925668 | -3.4617714 | -2.1875236 |
| CXADRP3 | -1.5358161 | -5.1122817 | -2.2268677 | 0.02870895 | 0.56925668 | -3.4634691 | -2.1874602 |
| SNORD127 | 1.31785557 | -3.9035963 | 2.22363026 | 0.02872778 | 0.56925668 | -3.4645821 | 2.18720205 |
| NAALADL2 | 0.45563271 | 1.83117357 | 2.22729779 | 0.02874638 | 0.56925668 | -3.4632794 | 2.18694719 |
| PTK2B | -0.1842733 | 4.95477514 | -2.2270764 | 0.02876551 | 0.56925668 | -3.4632475 | -2.1866852 |
| BACE2 | 0.75310432 | 3.77721876 | 2.22664811 | 0.02876818 | 0.56925668 | -3.4664707 | 2.18664866 |
| NT5DC4 | -1.447437 | -4.6871281 | -2.2245571 | 0.02877171 | 0.56925668 | -3.4660254 | -2.1866004 |
| SPMAP2L | 0.86632098 | -2.4148679 | 2.22563228 | 0.02881119 | 0.56925668 | -3.4651966 | 2.18606037 |
| IL17RB | -0.2183612 | 3.44257826 | -2.2256058 | 0.02884247 | 0.56925668 | -3.4654635 | -2.1856329 |
| SNRPEP2 | 1.59268571 | -4.5209086 | 2.22303912 | 0.02886827 | 0.56925668 | -3.4662074 | 2.1852807 |
| MZB1 | 1.48207832 | -3.9259437 | 2.22280153 | 0.02892145 | 0.56925668 | -3.4698877 | 2.18455557 |
| EPSTI1 | 1.22754835 | -0.3121127 | 2.22571576 | 0.02892239 | 0.56925668 | -3.4646414 | 2.18454281 |
| DOCK4 | -0.2340764 | 6.36562851 | -2.2240744 | 0.02894454 | 0.56925668 | -3.4680111 | -2.1842411 |
| PXT1 | 0.75244613 | -1.897949 | 2.22251148 | 0.02897463 | 0.56925668 | -3.4711505 | 2.18383147 |
| FAAH2 | 0.48734438 | 0.8531159 | 2.22224061 | 0.02899115 | 0.56925668 | -3.4708871 | 2.18360689 |
| RBP1 | 0.50959265 | 5.25687993 | 2.22369685 | 0.02900456 | 0.56925668 | -3.4691729 | 2.18342449 |
| FUOM | 0.5682066 | 1.75124498 | 2.22341733 | 0.0290137 | 0.56925668 | -3.4690136 | 2.18330027 |
| AMIGO1 | -0.2956024 | 5.06949509 | -2.2228256 | 0.02903862 | 0.56925668 | -3.4700341 | -2.1829618 |
| MGAT1 | 0.18820075 | 6.43882121 | 2.22216184 | 0.0291064 | 0.56925668 | -3.4712778 | 2.18204241 |
| LINC01451 | 1.16266689 | -3.1051565 | 2.22049093 | 0.02919497 | 0.56925668 | -3.4751098 | 2.18084388 |
| VENTXP6 | 1.44823678 | -5.8138665 | 2.21774172 | 0.02921504 | 0.56925668 | -3.4780476 | 2.18057271 |
| PLAAT2 | 1.2110259 | -3.2018614 | 2.21962388 | 0.02923036 | 0.56925668 | -3.4768345 | 2.18036582 |
| ARHGAP32 | -0.2236813 | 6.93285945 | -2.2190394 | 0.02931107 | 0.56925668 | -3.4764719 | -2.1792773 |
| RASEF | 0.71173526 | 0.58726664 | 2.21844251 | 0.0293535 | 0.56925668 | -3.4823107 | 2.17870615 |
| PLEKHA2 | 0.25948197 | 4.65374953 | 2.21755232 | 0.02941174 | 0.56925668 | -3.4790278 | 2.17792337 |
| DAPK1 | -0.2578318 | 7.11288185 | -2.2168135 | 0.02942931 | 0.56925668 | -3.480212 | -2.1776875 |
| MBD3L3 | -1.1274881 | -3.7395876 | -2.2166311 | 0.02945444 | 0.56925668 | -3.4778355 | -2.1773503 |
| FBXW7 | -0.2250563 | 5.95496182 | -2.2165143 | 0.02946461 | 0.56925668 | -3.4806647 | -2.1772139 |
| EFCAB10-AS1 | -0.2918174 | 1.49749263 | -2.2147713 | 0.02952795 | 0.56925668 | -3.483242 | -2.1763654 |
| DPYSL4 | -0.1741358 | 7.32028091 | -2.2155579 | 0.0295692 | 0.56925668 | -3.4823542 | -2.1758136 |
| ELP1 | -0.2167252 | 6.93551206 | -2.2154111 | 0.02957501 | 0.56925668 | -3.4825818 | -2.1757359 |
| FKBP9 | 0.36440013 | 7.29440252 | 2.21541483 | 0.02958369 | 0.56925668 | -3.4827528 | 2.17562001 |
| CA9 | 1.49772171 | -2.578882 | 2.21364456 | 0.0295851 | 0.56925668 | -3.4863181 | 2.17560108 |
| MYO18A | -0.2036099 | 7.41348466 | -2.2141629 | 0.02966584 | 0.56925668 | -3.4846386 | -2.1745235 |
| MYH2 | -1.7636356 | -4.3512365 | -2.2114531 | 0.02970578 | 0.56925668 | -3.4963164 | -2.1739915 |
| HYCC1 | 0.40889168 | 6.33334743 | 2.21251831 | 0.02973585 | 0.56925668 | -3.4876745 | 2.17359126 |
| NUDT19P3 | 1.49877509 | -5.1426494 | 2.2095671 | 0.029797 | 0.56925668 | -3.4922849 | 2.17277835 |
| TBILA | 1.60826965 | -1.3321229 | 2.21168731 | 0.02981154 | 0.56925668 | -3.4891114 | 2.17258529 |
| TGM5 | 1.43409041 | -3.5777755 | 2.21089774 | 0.0298214 | 0.56925668 | -3.4925485 | 2.17245453 |
| CDO1 | -0.3161299 | 6.40773858 | -2.2096359 | 0.02988353 | 0.56925668 | -3.4924336 | -2.1716307 |
| GALNT5 | 0.98645599 | 1.69667362 | 2.21123539 | 0.0298952 | 0.56925668 | -3.4893066 | 2.17147606 |
| MMD2 | -0.742348 | 3.12625972 | -2.2102436 | 0.0299555 | 0.56925668 | -3.4920563 | -2.1706782 |
| OR52N4 | 1.54995466 | -4.6903232 | 2.20802971 | 0.03001304 | 0.56925668 | -3.4943912 | 2.16991819 |
| BCL7A | -0.2780248 | 6.59654259 | -2.2092878 | 0.03001697 | 0.56925668 | -3.4927905 | -2.1698664 |
| HACD2 | -0.2877734 | 5.91883819 | -2.2078179 | 0.03001855 | 0.56925668 | -3.494682 | -2.1698455 |
| GREM1-AS1 | 1.43339784 | -6.4266461 | 2.20650096 | 0.03003474 | 0.56925668 | -3.4970527 | 2.16963189 |
| EIF4BP6 | 0.33414495 | 0.86968618 | 2.20793422 | 0.03006927 | 0.56925668 | -3.494789 | 2.16917673 |
| LMLN | 0.19232333 | 4.79013962 | 2.20815734 | 0.0300845 | 0.56925668 | -3.4947512 | 2.16897605 |
| FOS | -0.7120809 | 4.96928021 | -2.2072731 | 0.03012677 | 0.56925668 | -3.4985706 | -2.1684197 |
| BABAM2 | 0.22077846 | 4.61479603 | 2.20651532 | 0.03012819 | 0.56925668 | -3.4980501 | 2.16840103 |
| PTGS1 | 1.07583867 | 0.68164734 | 2.20640804 | 0.03014802 | 0.56925668 | -3.4975527 | 2.16814026 |
| NLRP9P1 | -1.3480492 | -5.90316 | -2.2056521 | 0.03021615 | 0.56925668 | -3.4985037 | -2.1672454 |
| LINC01278 | -0.3143464 | 4.82920556 | -2.2056187 | 0.03024611 | 0.56925668 | -3.4989654 | -2.1668525 |
| PRPH | -0.79478 | -1.5943002 | -2.2059916 | 0.03031482 | 0.56925668 | -3.5002115 | -2.1659525 |
| TTBK1 | -0.2290527 | 6.58976742 | -2.204722 | 0.03035665 | 0.56925668 | -3.5002296 | -2.1654054 |
| RN7SKP12 | 1.24283223 | -6.6670412 | 2.20088058 | 0.03036435 | 0.56925668 | -3.5057702 | 2.16530484 |
| TBL1X | 0.1579285 | 5.72915145 | 2.20440306 | 0.03037576 | 0.56925668 | -3.5010893 | 2.1651557 |
| RN7SL236P | 1.58716567 | -5.9637502 | 2.20384104 | 0.03038392 | 0.56925668 | -3.5013897 | 2.16504912 |
| ECEL1 | 1.01754538 | 5.08624353 | 2.20237084 | 0.0304037 | 0.56925668 | -3.5041155 | 2.16479094 |
| LRRIQ4 | -1.5149592 | -5.0819078 | -2.2015981 | 0.03042961 | 0.56925668 | -3.5051185 | -2.1644529 |
| MMP24OS | 0.25796585 | 4.9199618 | 2.20318026 | 0.03043732 | 0.56925668 | -3.5036862 | 2.16435234 |
| RNASE7 | 1.52383126 | -6.0444613 | 2.19897631 | 0.03047257 | 0.56925668 | -3.5098626 | 2.16389287 |
| KDSR | 0.21515075 | 5.83768652 | 2.20259657 | 0.03049746 | 0.56925668 | -3.5041401 | 2.16356874 |
| TANAR | -1.4939551 | -5.665419 | -2.2012156 | 0.03049938 | 0.56925668 | -3.5055759 | -2.1635437 |
| SLC36A4 | 0.28451554 | 6.46367355 | 2.20075532 | 0.03055369 | 0.56925668 | -3.5064975 | 2.16283735 |
| ACOT9 | 0.32425702 | 4.97532199 | 2.2009834 | 0.03057202 | 0.56925668 | -3.5071907 | 2.16259916 |
| LGALS8 | 0.34923344 | 4.9650394 | 2.20135133 | 0.030587 | 0.56925668 | -3.5051351 | 2.16240461 |
| RP9P | -1.4522432 | -4.6331557 | -2.1989625 | 0.03060224 | 0.56925668 | -3.5081976 | -2.1622068 |
| KRT7-AS | 1.34986564 | -6.475876 | 2.19777448 | 0.03060369 | 0.56925668 | -3.5120274 | 2.16218794 |
| PLEKHO2 | 0.23344729 | 2.89652311 | 2.2012883 | 0.03060519 | 0.56925668 | -3.5067794 | 2.16216853 |
| CEMIP | 1.19572787 | 3.50868081 | 2.19961994 | 0.03060991 | 0.56925668 | -3.5089087 | 2.16210726 |
| SPOCK2 | 0.50977025 | 9.16629992 | 2.20063283 | 0.03062246 | 0.56925668 | -3.507372 | 2.16194439 |
| PPIAP20 | 1.34530155 | -4.8768874 | 2.19837064 | 0.03062545 | 0.56925668 | -3.5097594 | 2.16190567 |
| GRM8 | -0.4154774 | 3.27857962 | -2.2002496 | 0.03066363 | 0.56925668 | -3.5075791 | -2.1614106 |
| KRT77 | -1.340103 | -5.6255789 | -2.1995228 | 0.03067126 | 0.56925668 | -3.5086933 | -2.1613118 |
| DNAJC8P2 | -1.2058553 | -3.5562646 | -2.196715 | 0.03068 | 0.56925668 | -3.511378 | -2.1611987 |
| LOXL1-AS1 | 0.7198587 | 0.21541157 | 2.1994594 | 0.0306843 | 0.56925668 | -3.5111316 | 2.16114289 |
| TYW1 | 0.1739371 | 4.74698506 | 2.1999041 | 0.0306966 | 0.56925668 | -3.508444 | 2.16098363 |
| RN7SL744P | 1.37771798 | -6.0325032 | 2.19650438 | 0.03074244 | 0.56925668 | -3.5135988 | 2.16039063 |
| ISCA1P6 | -1.4358954 | -5.2562622 | -2.1976992 | 0.03074849 | 0.56925668 | -3.5109136 | -2.1603125 |
| CALM1 | -0.19941 | 9.93066661 | -2.1989757 | 0.03078219 | 0.56925668 | -3.5101289 | -2.159877 |
| MAN1C1 | 0.64103207 | 5.16254874 | 2.19889585 | 0.03078909 | 0.56925668 | -3.5113373 | 2.15978789 |
| SMPD5 | -0.7849706 | -0.4984308 | -2.1987593 | 0.0307953 | 0.56925668 | -3.510849 | -2.1597077 |
| CCDC68 | 1.6871219 | -3.6747039 | 2.19975113 | 0.03080096 | 0.56925668 | -3.5049878 | 2.1596347 |
| A2M-AS1 | 0.75909108 | 0.69608921 | 2.19749666 | 0.03084396 | 0.56925668 | -3.5120321 | 2.15907995 |
| LIMS1-AS1 | -1.4607768 | -3.9229305 | -2.1942128 | 0.03085902 | 0.56925668 | -3.5138101 | -2.1588859 |
| RDM1P3 | 1.07190277 | -3.2517761 | 2.19513905 | 0.03091368 | 0.56925668 | -3.517991 | 2.15818208 |
| TMPRSS15 | 0.8523407 | -2.0480376 | 2.19597965 | 0.03094339 | 0.56925668 | -3.5144541 | 2.15779993 |
| MYOT | -1.0775211 | -3.2620872 | -2.1954924 | 0.03098602 | 0.56925668 | -3.5209642 | -2.1572521 |
| GRIA2 | -0.4990749 | 7.9359365 | -2.1954892 | 0.03099901 | 0.56925668 | -3.5157112 | -2.1570854 |
| DDN | -0.6250191 | 3.09947951 | -2.1944821 | 0.03105337 | 0.56925668 | -3.5169339 | -2.1563881 |
| TAF9P3 | 0.88694873 | -2.9345824 | 2.19077246 | 0.03106696 | 0.56925668 | -3.520236 | 2.15621401 |
| C1GALT1 | 0.35528997 | 5.29901234 | 2.19311992 | 0.0310992 | 0.56925668 | -3.5206244 | 2.15580105 |
| SAT1 | 0.66262983 | 6.26726735 | 2.19426155 | 0.03110432 | 0.56925668 | -3.5181181 | 2.15573555 |
| SEMG2 | -1.3835508 | -5.8163568 | -2.1940312 | 0.03114525 | 0.56925668 | -3.5176347 | -2.155212 |
| CPXM1 | 0.64218299 | 6.39114128 | 2.19536631 | 0.03114682 | 0.56925668 | -3.5161657 | 2.15519187 |
| EDC3 | -0.1203558 | 5.57586153 | -2.1937387 | 0.03115815 | 0.56925668 | -3.5185221 | -2.1550471 |
| SSPN | 0.60728993 | 4.94374563 | 2.19375064 | 0.03116123 | 0.56925668 | -3.5207459 | 2.15500774 |
| NMUR1 | 1.14595121 | -2.3370713 | 2.19229329 | 0.03119586 | 0.56925668 | -3.5203258 | 2.15456536 |
| ZBTB42 | 0.54155065 | 2.39065935 | 2.191988 | 0.03128235 | 0.56925668 | -3.5225496 | 2.15346243 |
| MEG8 | 0.94182854 | 0.79981918 | 2.19158053 | 0.03130154 | 0.56925668 | -3.5285256 | 2.15321819 |
| MTMR14 | 0.16484587 | 5.02353346 | 2.18985616 | 0.03136536 | 0.56925668 | -3.5245228 | 2.1524064 |
| ITGA1 | 0.95066519 | 0.31398891 | 2.19104486 | 0.03138254 | 0.56925668 | -3.5231376 | 2.15218817 |
| FHIP1A-DT | 1.21897956 | -3.6080566 | 2.18923966 | 0.03140947 | 0.56925668 | -3.5259038 | 2.15184624 |
| HMGA2-AS1 | 1.47698738 | -6.0502992 | 2.18868742 | 0.03141742 | 0.56925668 | -3.5264953 | 2.15174536 |
| MAGEC3 | -0.9198721 | -0.1760383 | -2.1883418 | 0.03144084 | 0.56925668 | -3.5292039 | -2.1514482 |
| PRADC1P1 | 1.39007141 | -3.5566296 | 2.18998302 | 0.03144589 | 0.56925668 | -3.5220037 | 2.15138419 |
| GCNT4 | 0.48766141 | 1.57896626 | 2.18902029 | 0.03147288 | 0.56925668 | -3.5290183 | 2.15104208 |
| CD200R1 | -1.2369085 | -2.9989512 | -2.1849598 | 0.03152905 | 0.56925668 | -3.5301828 | -2.1503309 |
| SCG3 | -0.3877849 | 7.22689972 | -2.1885849 | 0.0315343 | 0.56925668 | -3.5272486 | -2.1502645 |
| PLS3 | 0.71291404 | 7.2711085 | 2.18842159 | 0.03153907 | 0.56925668 | -3.527635 | 2.15020414 |
| B3GALT9 | 0.30869521 | 2.98447045 | 2.1881739 | 0.03154458 | 0.56925668 | -3.5318792 | 2.15013451 |
| DTX3L | 0.58184682 | 3.85116646 | 2.18869061 | 0.03154657 | 0.56925668 | -3.5290745 | 2.15010928 |
| LINC01750 | -0.6111186 | 1.65292029 | -2.1889495 | 0.03154731 | 0.56925668 | -3.5266128 | -2.1501 |
| PPP1R3B | 0.75606664 | 3.26459782 | 2.18790169 | 0.03158127 | 0.56925668 | -3.5364599 | 2.14967068 |
| AGA | 0.30322871 | 4.16750771 | 2.18810954 | 0.03159223 | 0.56925668 | -3.5295906 | 2.14953231 |
| FBLN5 | 0.91345572 | 8.96950103 | 2.18779366 | 0.03159786 | 0.56925668 | -3.528791 | 2.14946114 |
| FAM171A2 | -0.2649641 | 7.8145368 | -2.1878706 | 0.03161055 | 0.56925668 | -3.5283264 | -2.149301 |
| DCAF4 | 0.22009445 | 3.8322467 | 2.18610893 | 0.03162617 | 0.56925668 | -3.5304367 | 2.1491038 |
| C11orf97 | -1.1050657 | -1.8658919 | -2.186916 | 0.03162787 | 0.56925668 | -3.5294001 | -2.1490823 |
| NT5DC1 | 0.38636933 | 4.17729758 | 2.18695663 | 0.03163966 | 0.56925668 | -3.5302621 | 2.14893363 |
| IGFALS | 0.41247485 | 0.94047517 | 2.1903384 | 0.0316466 | 0.56925668 | -3.5243959 | 2.14884606 |
| IP6K2 | -0.1669931 | 6.85172749 | -2.1859536 | 0.03171954 | 0.5696569 | -3.531567 | -2.1479272 |
| BICD1 | -0.3012722 | 6.35570385 | -2.1853638 | 0.03173732 | 0.5696569 | -3.5325987 | -2.1477034 |
| NRON | -1.1529069 | -6.1847227 | -2.1842788 | 0.03175673 | 0.5696569 | -3.5351326 | -2.1474594 |
| TMEM65 | -0.2758645 | 6.50245809 | -2.1819535 | 0.03179127 | 0.5696569 | -3.5367329 | -2.1470252 |
| APC2 | -0.2621527 | 9.84372275 | -2.1852519 | 0.03179635 | 0.5696569 | -3.5328664 | -2.1469615 |
| AGAP1-IT1 | -1.5266251 | -5.2534006 | -2.1818605 | 0.03187683 | 0.57064123 | -3.538004 | -2.1459517 |
| PPP1CB | 0.39491923 | 8.10189003 | 2.18165299 | 0.031972 | 0.57117269 | -3.5391608 | 2.14476047 |
| GPR162 | -0.1537943 | 6.59420282 | -2.1819775 | 0.03198932 | 0.57117269 | -3.5378488 | -2.144544 |
| BDKRB1 | -1.2994321 | -6.5778061 | -2.1823701 | 0.03200263 | 0.57117269 | -3.5378368 | -2.1443778 |
| PHF10P1 | -1.3479188 | -5.1148365 | -2.1790853 | 0.03202995 | 0.57117269 | -3.5419033 | -2.1440367 |
| PDE4DIPP2 | -0.3041853 | 4.50550878 | -2.180848 | 0.03207185 | 0.57117269 | -3.5392558 | -2.1435139 |
| SLC25A13 | 0.2301869 | 4.44300719 | 2.18113302 | 0.0320846 | 0.57117269 | -3.54095 | 2.14335507 |
| JMJD8 | 0.21418983 | 4.8855638 | 2.18153616 | 0.03208548 | 0.57117269 | -3.5388981 | 2.14334407 |
| DLL3 | -0.6343871 | 4.92483463 | -2.1799089 | 0.03217467 | 0.57230433 | -3.5422306 | -2.1422339 |
| SNRPA1-DT | -0.7561875 | -0.6279401 | -2.1795473 | 0.03221077 | 0.57234242 | -3.5430636 | -2.1417852 |
| DSG2 | 0.75385597 | 3.73456631 | 2.17899117 | 0.03222805 | 0.57234242 | -3.5433593 | 2.14157067 |
| IQCG | 0.45768513 | 5.66735931 | 2.17848481 | 0.03231736 | 0.57246093 | -3.5449981 | 2.14046315 |
| ESRP2 | -0.9323591 | -2.2468119 | -2.1743004 | 0.03232913 | 0.57246093 | -3.5459565 | -2.1403173 |
| NUP107-DT | 1.01722688 | -2.8079811 | 2.17583913 | 0.03235398 | 0.57246093 | -3.5500278 | 2.14000979 |
| PLEKHF1 | 0.572782 | 1.25762149 | 2.17778808 | 0.03237926 | 0.57246093 | -3.5492678 | 2.13969708 |
| LINGO1-AS1 | -1.2155139 | -2.8070775 | -2.1741625 | 0.03238774 | 0.57246093 | -3.5464714 | -2.1395922 |
| KCNH3 | -0.4248884 | 3.31472344 | -2.177617 | 0.03238846 | 0.57246093 | -3.5445837 | -2.1395833 |
| PDE4B | 0.38848242 | 6.96397539 | 2.1759378 | 0.03243403 | 0.57281325 | -3.5484714 | 2.13902018 |
| RN7SL268P | -0.5712441 | 0.23994035 | -2.1755881 | 0.03249447 | 0.57342725 | -3.5476061 | -2.1382745 |
| RIPK1 | 0.25036477 | 5.26401548 | 2.175412 | 0.03255837 | 0.57410145 | -3.5491366 | 2.1374874 |
| DCT | 1.30925412 | 2.07265164 | 2.17345743 | 0.03261886 | 0.57457658 | -3.5521197 | 2.1367435 |
| NXPE3 | 0.24071004 | 5.89559754 | 2.17370921 | 0.03263675 | 0.57457658 | -3.5520183 | 2.13652369 |
| ETF1 | 0.16667349 | 6.73548473 | 2.17236761 | 0.03272751 | 0.57572073 | -3.5544187 | 2.13541028 |
| DPAGT1 | 0.24414148 | 4.40482348 | 2.17159023 | 0.03282255 | 0.57637676 | -3.5553869 | 2.13424722 |
| CCL18 | -1.1728465 | -6.8624373 | -2.1680143 | 0.03283474 | 0.57637676 | -3.560751 | -2.1340983 |
| LINC01505 | -0.7621159 | -1.3504205 | -2.1718804 | 0.0328422 | 0.57637676 | -3.5537389 | -2.1340071 |
| KYAT3 | 0.2320034 | 5.19056415 | 2.17115048 | 0.03290425 | 0.57668766 | -3.5564425 | 2.13324963 |
| GAPLINC | 1.55328846 | -5.2195085 | 2.16916207 | 0.03291393 | 0.57668766 | -3.5578671 | 2.13313163 |
| ELAC2 | 0.12178048 | 6.43092568 | 2.1697724 | 0.03299636 | 0.57668766 | -3.5582315 | 2.13212754 |
| VAMP3 | 0.24902464 | 5.75862962 | 2.16972601 | 0.03300307 | 0.57668766 | -3.5592089 | 2.13204589 |
| XKR9 | 0.79693551 | -2.0122112 | 2.16923313 | 0.03300892 | 0.57668766 | -3.5580468 | 2.13197481 |
| INPP5B | 0.16650642 | 4.61997148 | 2.16931193 | 0.03301923 | 0.57668766 | -3.5587933 | 2.13184937 |
| RAB33B-AS1 | -0.2355583 | 2.41976461 | -2.1692328 | 0.03304061 | 0.57668766 | -3.5581668 | -2.1315895 |
| F2 | -1.6969644 | -4.3455251 | -2.1660905 | 0.03309504 | 0.57710396 | -3.5610239 | -2.1309284 |
| HSPH1 | -0.1578238 | 7.6824205 | -2.1684792 | 0.03311612 | 0.57710396 | -3.5603553 | -2.1306726 |
| MIR9-1HG | -0.4836059 | 8.14014875 | -2.166843 | 0.03315259 | 0.57728917 | -3.5630999 | -2.1302305 |
| PROB1 | 0.4592125 | 3.14359652 | 2.16741177 | 0.03318433 | 0.5773918 | -3.5627366 | 2.12984602 |
| PAIP2B | -0.3298888 | 4.36749953 | -2.1667126 | 0.03322429 | 0.57763723 | -3.5625632 | -2.1293624 |
| SCRT2 | -0.585877 | 4.92493701 | -2.1658727 | 0.03326731 | 0.57793541 | -3.5637155 | -2.1288423 |
| LINC02516 | 1.3555921 | -4.8326781 | 2.16193334 | 0.0334025 | 0.57983322 | -3.5703225 | 2.12721161 |
| STX17-DT | 0.52743367 | 0.28215324 | 2.16222308 | 0.03347382 | 0.58014074 | -3.5709645 | 2.12635373 |
| PLA2G4D | 1.39812378 | -4.9826655 | 2.16152718 | 0.03356847 | 0.58014074 | -3.5751936 | 2.12521753 |
| SLC51B | -1.5031542 | -4.2264047 | -2.1606935 | 0.03357026 | 0.58014074 | -3.5712104 | -2.125196 |
| ABRAXAS1 | 0.20523752 | 2.78294915 | 2.16198963 | 0.03359005 | 0.58014074 | -3.5714277 | 2.12495885 |
| RPF2 | 0.15758161 | 4.00314694 | 2.15982434 | 0.03360633 | 0.58014074 | -3.5746361 | 2.1247638 |
| SMIM3 | 0.34498529 | 2.25252745 | 2.16127346 | 0.03365712 | 0.58014074 | -3.5733155 | 2.12415573 |
| SNORD83A | -1.015336 | -2.7742717 | -2.1581401 | 0.03370902 | 0.58014074 | -3.5745037 | -2.1235353 |
| TRIM71 | -0.5134851 | 3.32374098 | -2.1607135 | 0.03373663 | 0.58014074 | -3.5718283 | -2.1232056 |
| DPF3 | -0.4080354 | 5.17087671 | -2.160098 | 0.03373926 | 0.58014074 | -3.5733819 | -2.1231741 |
| PCK1 | 1.20665644 | -1.4830329 | 2.15991283 | 0.03378737 | 0.58014074 | -3.5743575 | 2.1226002 |
| DMGDH | 0.35355008 | 1.76567396 | 2.16050512 | 0.03379705 | 0.58014074 | -3.5720318 | 2.12248479 |
| RBM41 | -0.186242 | 4.77409761 | -2.1584219 | 0.03385778 | 0.58014074 | -3.5763001 | -2.1217614 |
| FAM104B | -0.2180785 | 4.3260391 | -2.1571978 | 0.03388178 | 0.58014074 | -3.5782039 | -2.1214758 |
| NAIPP2 | -0.2714596 | 1.61386616 | -2.1579376 | 0.03389413 | 0.58014074 | -3.5770733 | -2.1213289 |
| LY96 | 1.66871579 | -3.5691794 | 2.15809176 | 0.03389724 | 0.58014074 | -3.5763014 | 2.12129199 |
| ATAD3A | 0.15291747 | 5.59048454 | 2.15761176 | 0.03392033 | 0.58014074 | -3.5780261 | 2.12101747 |
| CLIP4 | 0.33309476 | 5.20112956 | 2.1577558 | 0.0339232 | 0.58014074 | -3.5779525 | 2.12098338 |
| ITPRIPL2 | 0.57537421 | 3.90589813 | 2.15640255 | 0.03405011 | 0.58014074 | -3.583286 | 2.11947779 |
| SRRM4 | -0.3640355 | 6.55612513 | -2.1565801 | 0.03405133 | 0.58014074 | -3.5787534 | -2.1194633 |
| NAPA | 0.12571901 | 5.90157421 | 2.15438854 | 0.03407873 | 0.58014074 | -3.5837254 | 2.1191389 |
| PHYHIPL | -0.419849 | 6.31537432 | -2.1564012 | 0.03408179 | 0.58014074 | -3.5805874 | -2.1191027 |
| SERPINB1 | 0.80499207 | 1.21541685 | 2.15585177 | 0.03409931 | 0.58014074 | -3.579734 | 2.11889537 |
| VNN2 | 1.7308063 | -5.8015993 | 2.15650735 | 0.03411568 | 0.58014074 | -3.5825374 | 2.11870178 |
| CBFA2T2 | -0.1523809 | 6.70011075 | -2.1553727 | 0.03413991 | 0.58014074 | -3.5817853 | -2.1184153 |
| CD44-DT | 1.09234376 | -1.7881412 | 2.1557057 | 0.03416315 | 0.58014074 | -3.581469 | 2.11814069 |
| ZNG1A | 0.18640063 | 4.9358621 | 2.15468787 | 0.03422155 | 0.58014074 | -3.5825388 | 2.11745147 |
| FABP12 | 1.3134384 | -6.0544453 | 2.15167537 | 0.03422815 | 0.58014074 | -3.5916628 | 2.11737368 |
| AMDHD2 | 0.26090154 | 4.33088661 | 2.15257505 | 0.03426792 | 0.58014074 | -3.5851848 | 2.11690493 |
| USP3 | -0.1584007 | 5.14249855 | -2.153538 | 0.03427259 | 0.58014074 | -3.5843973 | -2.1168499 |
| RFT1 | 0.16387726 | 4.79032712 | 2.15383038 | 0.03428106 | 0.58014074 | -3.5844009 | 2.1167501 |
| LINC02048 | 1.440955 | -4.4191527 | 2.15288717 | 0.03428151 | 0.58014074 | -3.5889169 | 2.11674488 |
| NHLRC1 | 0.4565803 | 1.01805776 | 2.15240845 | 0.03428453 | 0.58014074 | -3.5868164 | 2.11670925 |
| CDRT15 | 1.02524311 | -2.9523009 | 2.15094486 | 0.03433218 | 0.58014074 | -3.5881531 | 2.11614851 |
| LMOD3 | -1.2741144 | -3.569093 | -2.1499266 | 0.03439107 | 0.58014074 | -3.5887752 | -2.1154564 |
| LINC03100 | -0.6654586 | 0.33069412 | -2.1524993 | 0.03439381 | 0.58014074 | -3.5848311 | -2.1154242 |
| NRXN3 | -0.6982816 | 6.58902481 | -2.150418 | 0.03440733 | 0.58014074 | -3.5894722 | -2.1152655 |
| SOD2 | 0.71760625 | 7.80408126 | 2.15161138 | 0.03443186 | 0.58014074 | -3.5885964 | 2.11497762 |
| SMAP2 | -0.2558376 | 6.50848457 | -2.1511281 | 0.03448726 | 0.58014074 | -3.588377 | -2.114328 |
| RIPOR3 | 0.71328571 | 3.50322648 | 2.15095736 | 0.03449807 | 0.58014074 | -3.5897891 | 2.1142014 |
| VDAC1P8 | 0.32295245 | 2.20545006 | 2.1495106 | 0.03457102 | 0.58014074 | -3.5914646 | 2.11334764 |
| DRG1P1 | 1.42166927 | -4.8988887 | 2.14627234 | 0.03457165 | 0.58014074 | -3.5969115 | 2.11334037 |
| RHPN1-AS1 | 0.49206605 | 0.96345802 | 2.14922428 | 0.03457607 | 0.58014074 | -3.5941371 | 2.11328866 |
| PIM2 | 0.30118293 | 3.74529314 | 2.1491236 | 0.03460168 | 0.58014074 | -3.5940069 | 2.11298938 |
| FLJ30679 | 1.22847 | -3.0309607 | 2.14738661 | 0.03462383 | 0.58014074 | -3.5949636 | 2.11273064 |
| RASGRF1 | -0.6023517 | 4.66115023 | -2.1496872 | 0.03463714 | 0.58014074 | -3.5893717 | -2.1125753 |
| MTTP | 0.54192294 | 3.44359407 | 2.14873496 | 0.03466131 | 0.58014074 | -3.5997267 | 2.11229318 |
| XRCC6P4 | -1.48657 | -5.1934143 | -2.1461962 | 0.03466532 | 0.58014074 | -3.5951173 | -2.1122464 |
| SLC7A5 | 0.38363267 | 6.83972023 | 2.14659826 | 0.03468486 | 0.58014074 | -3.5963724 | 2.1120185 |
| ERCC6 | -0.2552023 | 4.85005455 | -2.1482529 | 0.03472956 | 0.58014074 | -3.5922689 | -2.1114976 |
| PRR13P2 | 1.49090155 | -6.0303289 | 2.14555588 | 0.03474954 | 0.58014074 | -3.5987255 | 2.11126502 |
| E2F5 | 0.24056738 | 4.85635814 | 2.14747271 | 0.03475948 | 0.58014074 | -3.5963849 | 2.11114933 |
| MRC2 | 0.68541513 | 7.84464633 | 2.14771516 | 0.03477053 | 0.58014074 | -3.5949143 | 2.11102071 |
| KDM4D | 0.23894838 | 2.40084932 | 2.14535079 | 0.0348174 | 0.58048915 | -3.5993097 | 2.11047576 |
| CISH | 0.58017663 | 1.06507663 | 2.14497102 | 0.03485391 | 0.58066466 | -3.598719 | 2.11005156 |
| PLEKHA4 | 0.80045272 | 4.61187274 | 2.14593164 | 0.03491194 | 0.58069298 | -3.6008324 | 2.10937832 |
| GCNT1P1 | 1.1682388 | -2.1260016 | 2.14320517 | 0.03496011 | 0.58069298 | -3.597905 | 2.10882015 |
| MRRF | 0.2184287 | 4.9740291 | 2.14497527 | 0.03498555 | 0.58069298 | -3.5989768 | 2.10852564 |
| RN7SL165P | 1.21222274 | -6.3605461 | 2.14252554 | 0.03500016 | 0.58069298 | -3.6064235 | 2.10835659 |
| RNU6-969P | -1.4485079 | -4.7263258 | -2.1424035 | 0.03502093 | 0.58069298 | -3.6051529 | -2.1081163 |
| DNAJB12P1 | -1.1842214 | -4.3861012 | -2.1429285 | 0.03504476 | 0.58069298 | -3.6031842 | -2.1078408 |
| TMEM89 | 0.9508805 | -2.3179683 | 2.1480434 | 0.03505828 | 0.58069298 | -3.5914707 | 2.10768464 |
| PTGFRN | 0.32770388 | 8.0922056 | 2.14410894 | 0.03507062 | 0.58069298 | -3.6005182 | 2.10754208 |
| MRLN | -1.2347223 | -4.3307676 | -2.1435209 | 0.03510932 | 0.58069298 | -3.598791 | -2.1070953 |
| AVPR1A | 1.28440736 | 1.67618208 | 2.14305401 | 0.03511554 | 0.58069298 | -3.5998455 | 2.10702356 |
| BPIFB1 | -1.3119996 | -5.7753285 | -2.1412157 | 0.03515785 | 0.58096269 | -3.6030788 | -2.1065356 |
| SPATA12 | 1.13840401 | -3.6129881 | 2.14236842 | 0.03519973 | 0.58122477 | -3.6081072 | 2.10605324 |
| JPH4 | -0.3426879 | 7.51513827 | -2.1415816 | 0.03527979 | 0.58182753 | -3.6038037 | -2.1051323 |
| CPLX2 | -0.418458 | 7.03481271 | -2.1411804 | 0.03528832 | 0.58182753 | -3.6042324 | -2.1050344 |
| TMEM229A | -0.4526543 | 2.04023769 | -2.1401815 | 0.0353177 | 0.58188255 | -3.6045355 | -2.1046969 |
| ST6GALNAC2 | 1.15909689 | -1.4856018 | 2.13912254 | 0.03547511 | 0.58404526 | -3.6100221 | 2.10289317 |
| NEAT1 | 1.01125156 | 6.97009095 | 2.1381426 | 0.03551698 | 0.58429136 | -3.6098687 | 2.10241447 |
| TCTN3 | 0.26974845 | 6.05744173 | 2.13823188 | 0.03554735 | 0.58429136 | -3.6103883 | 2.10206768 |
| HES5 | -1.2502715 | 2.20050602 | -2.1385514 | 0.03556852 | 0.58429136 | -3.6073725 | -2.101826 |
| SDR42E1 | -1.3731894 | -5.8503918 | -2.136032 | 0.03559576 | 0.58430921 | -3.6159846 | -2.1015153 |
| PLPPR5 | -0.4478086 | 4.77977906 | -2.1367215 | 0.03566837 | 0.58507124 | -3.6101864 | -2.1006879 |
| MEAK7 | -0.1253111 | 4.05754672 | -2.1348359 | 0.03570754 | 0.58528411 | -3.6144349 | -2.1002422 |
| MICAL3 | -0.3023854 | 6.92982194 | -2.1359095 | 0.03573895 | 0.58536942 | -3.613119 | -2.0998851 |
| ENTREP2 | -0.3805412 | 4.417554 | -2.1354196 | 0.03578217 | 0.58564788 | -3.6120625 | -2.0993942 |
| PDZRN4 | -0.5610113 | 3.30431254 | -2.1341908 | 0.03582452 | 0.58591187 | -3.6192637 | -2.0989136 |
| PDE4DIP | -0.3458657 | 7.62684548 | -2.1336548 | 0.03592782 | 0.58632093 | -3.6175804 | -2.0977434 |
| TXLNA | 0.16730259 | 6.52087833 | 2.1329457 | 0.0359669 | 0.58632093 | -3.6186197 | 2.09730141 |
| FTSJ3 | 0.15480108 | 6.063509 | 2.13332881 | 0.03596766 | 0.58632093 | -3.6178855 | 2.09729284 |
| APOO | -0.1741187 | 4.63770186 | -2.1327596 | 0.03599359 | 0.58632093 | -3.6184998 | -2.0969998 |
| ZBED3 | 0.25684139 | 4.9354702 | 2.13290881 | 0.03601014 | 0.58632093 | -3.6190597 | 2.09681293 |
| SP140L | 0.7674376 | 0.60749113 | 2.13266518 | 0.0360282 | 0.58632093 | -3.6189321 | 2.09660899 |
| MATR3P3 | -1.3915821 | -3.7527829 | -2.1291742 | 0.03607805 | 0.58632093 | -3.6289314 | -2.0960466 |
| PARP10 | 0.40559456 | 4.1312801 | 2.13143885 | 0.0361076 | 0.58632093 | -3.6212529 | 2.09571362 |
| CELF5 | -0.4087603 | 7.57732521 | -2.1313889 | 0.03613314 | 0.58632093 | -3.6203128 | -2.095426 |
| PXMP4 | 0.21971314 | 3.82783016 | 2.13037952 | 0.03620201 | 0.58632093 | -3.6222228 | 2.09465123 |
| LINC00161 | 1.25533112 | -6.3776146 | 2.12741746 | 0.03621415 | 0.58632093 | -3.6265207 | 2.09451475 |
| FITM1 | 0.63862119 | -1.1901691 | 2.13098788 | 0.03622592 | 0.58632093 | -3.6185724 | 2.09438246 |
| NBPF7P | -1.1326663 | -6.4949974 | -2.128151 | 0.0362403 | 0.58632093 | -3.6261905 | -2.094221 |
| NSMCE2 | 0.23005467 | 3.42433594 | 2.12762894 | 0.03629188 | 0.58632093 | -3.6281908 | 2.09364206 |
| MPPE1 | 0.22016697 | 4.48553197 | 2.12808616 | 0.03630895 | 0.58632093 | -3.6255138 | 2.09345054 |
| SPDYA | 0.49293924 | -0.369529 | 2.1271666 | 0.03633983 | 0.58632093 | -3.6247973 | 2.09310442 |
| CNTNAP3B | 0.30205836 | 2.32631128 | 2.1286095 | 0.03636048 | 0.58632093 | -3.6252185 | 2.09287317 |
| WTAP | 0.24909751 | 6.49100785 | 2.12746981 | 0.0363794 | 0.58632093 | -3.6278357 | 2.0926613 |
| FBXO38-DT | -0.3298983 | 1.55061971 | -2.1278777 | 0.03642153 | 0.58632093 | -3.6258064 | -2.0921899 |
| PLEKHG4B | 0.69772072 | 6.78519977 | 2.12753384 | 0.03643864 | 0.58632093 | -3.6283578 | 2.09199859 |
| TNFSF14 | 1.67436228 | -5.6847647 | 2.1273548 | 0.03650007 | 0.58632093 | -3.6359587 | 2.09131236 |
| CELF2-AS1 | -0.4666501 | 1.39568507 | -2.1257241 | 0.03651445 | 0.58632093 | -3.6270555 | -2.0911519 |
| RN7SKP23 | 0.88644368 | 0.28431206 | 2.1279741 | 0.03657552 | 0.58632093 | -3.6233847 | 2.09047087 |
| G0S2 | -1.2220896 | -2.538362 | -2.1235335 | 0.03665458 | 0.58632093 | -3.6374956 | -2.0895907 |
| OSBPL9 | 0.20530424 | 6.33171049 | 2.12425309 | 0.03666397 | 0.58632093 | -3.633018 | 2.08948627 |
| ZNF285 | -0.2786031 | 3.95654689 | -2.1247412 | 0.03667037 | 0.58632093 | -3.6296852 | -2.0894151 |
| PGPEP1 | 0.48631535 | 5.16876486 | 2.12496892 | 0.0366741 | 0.58632093 | -3.6328653 | 2.08937367 |
| PNRC2P1 | -1.2605494 | -3.367511 | -2.1206854 | 0.03671484 | 0.58632093 | -3.6356141 | -2.0889209 |
| SLC38A5 | -0.6288069 | 0.02453308 | -2.1236719 | 0.03676088 | 0.58632093 | -3.6302939 | -2.0884098 |
| MIR6739 | 0.90051267 | -2.7715501 | 2.1229485 | 0.03676438 | 0.58632093 | -3.6352432 | 2.08837103 |
| INHCAP | -0.8025665 | -2.1627361 | -2.1222185 | 0.03676927 | 0.58632093 | -3.6299657 | -2.0883167 |
| SLC37A1 | 0.26516992 | 5.67478641 | 2.12366815 | 0.03677539 | 0.58632093 | -3.6336291 | 2.08824886 |
| AQP5-AS1 | 1.05613533 | -2.671399 | 2.12272663 | 0.03681362 | 0.58632093 | -3.6353506 | 2.08782507 |
| CEP250 | -0.2248887 | 6.49929391 | -2.1228801 | 0.03683529 | 0.58632093 | -3.6343583 | -2.087585 |
| GRN | 0.46838431 | 7.03095044 | 2.12282417 | 0.03684607 | 0.58632093 | -3.6354792 | 2.08746553 |
| LINC02080 | -1.5234642 | -4.3377562 | -2.1219648 | 0.03685653 | 0.58632093 | -3.6328147 | -2.0873498 |
| LRIT2 | 1.30403313 | -3.3312307 | 2.12214431 | 0.03685753 | 0.58632093 | -3.6344767 | 2.08733866 |
| LINC01968 | 1.58472595 | -5.1016243 | 2.12027412 | 0.0368703 | 0.58632093 | -3.6443384 | 2.08719734 |
| MCM8-AS1 | 1.39301042 | -4.8351253 | 2.1187669 | 0.03689587 | 0.58632093 | -3.6358448 | 2.08691439 |
| CATSPERB | 0.79885706 | -0.8362549 | 2.12289068 | 0.0368993 | 0.58632093 | -3.6337069 | 2.08687651 |
| CA5BP1 | -1.1231978 | -3.5670939 | -2.1201695 | 0.03695787 | 0.58659426 | -3.6386249 | -2.0862291 |
| TBX15 | -0.5416024 | 0.57659426 | -2.1219902 | 0.03697397 | 0.58659426 | -3.6383583 | -2.0860514 |
| ITIH3 | -0.7447952 | -0.7530214 | -2.122721 | 0.03699527 | 0.58659426 | -3.6310194 | -2.0858163 |
| G6PC2 | -1.5584988 | -5.493165 | -2.1192113 | 0.03702855 | 0.58670564 | -3.6376307 | -2.0854491 |
| TVP23B | 0.22378105 | 4.83966087 | 2.12007722 | 0.0370746 | 0.58701888 | -3.6393729 | 2.08494162 |
| CCNH | 0.1767986 | 5.00380586 | 2.1190321 | 0.03711704 | 0.58702829 | -3.6400931 | 2.08447432 |
| PTGR1 | 0.37340896 | 3.81688179 | 2.1194196 | 0.03714849 | 0.58702829 | -3.6413051 | 2.08412838 |
| IMMP2L | 0.31839287 | 1.99604072 | 2.11886399 | 0.03719156 | 0.58702829 | -3.6445402 | 2.08365505 |
| STAMBPL1 | 0.41943203 | 3.13957898 | 2.11857567 | 0.03723 | 0.58702829 | -3.6400327 | 2.08323284 |
| BATF3 | 0.86582318 | 0.80987088 | 2.1162534 | 0.03732185 | 0.58702829 | -3.6449828 | 2.08222575 |
| DQX1 | -1.1646601 | -2.1168067 | -2.1185384 | 0.0373269 | 0.58702829 | -3.6382144 | -2.0821705 |
| MIF4GD | 0.2081083 | 4.5703625 | 2.11755851 | 0.0373295 | 0.58702829 | -3.6430141 | 2.08214204 |
| POP1 | -0.1696677 | 4.22813263 | -2.1167884 | 0.03733184 | 0.58702829 | -3.6421667 | -2.0821163 |
| NSG2 | -0.4359098 | 9.09967251 | -2.117454 | 0.0373365 | 0.58702829 | -3.6431367 | -2.0820653 |
| CNIH4 | 0.21976925 | 4.73878241 | 2.11702808 | 0.03734557 | 0.58702829 | -3.6446707 | 2.08196601 |
| MTSS1 | -0.2742608 | 6.70974402 | -2.1170786 | 0.03736423 | 0.58702829 | -3.6435123 | -2.0817618 |
| TNFRSF10A | 1.19090651 | -1.1201517 | 2.11497158 | 0.03744433 | 0.58770762 | -3.6509086 | 2.08088611 |
| KAZN | 0.38002622 | 7.10143829 | 2.11570934 | 0.03746008 | 0.58770762 | -3.647038 | 2.08071413 |
| FAM89B | -0.3076737 | 2.26951239 | -2.1151528 | 0.03750929 | 0.58803479 | -3.6466079 | -2.0801771 |
| TMEM38A | 0.23064554 | 4.46694175 | 2.11415626 | 0.03754027 | 0.58803479 | -3.6478846 | 2.07983935 |
| PIGT | 0.28769562 | 6.8391455 | 2.11469231 | 0.03756406 | 0.58803479 | -3.6482104 | 2.07958016 |
| DYNLRB2-AS1 | -1.04122 | -3.193434 | -2.1125801 | 0.03758622 | 0.58803479 | -3.654071 | -2.0793388 |
| ABCD1 | 0.19654881 | 4.31174028 | 2.11325234 | 0.03768133 | 0.58878391 | -3.6511541 | 2.07830442 |
| CDK14 | -0.2396182 | 6.79838343 | -2.1125102 | 0.03769941 | 0.58878391 | -3.6514658 | -2.078108 |
| FLRT2-AS1 | -0.6213983 | 0.31525891 | -2.1131522 | 0.03771316 | 0.58878391 | -3.6468713 | -2.0779587 |
| DGKI | -0.4986058 | 6.04726016 | -2.1124351 | 0.03774047 | 0.58879881 | -3.6503328 | -2.0776623 |
| ELF4 | 0.71479873 | 1.72009128 | 2.11226444 | 0.03780075 | 0.58911802 | -3.6539523 | 2.07700873 |
| IRF1 | 0.64848045 | 2.55795529 | 2.11229084 | 0.03781367 | 0.58911802 | -3.6535512 | 2.07686878 |
| TM9SF1 | 0.3256754 | 2.96159209 | 2.11141468 | 0.03784014 | 0.58911958 | -3.6524379 | 2.07658216 |
| GGACT | 0.43164573 | 0.92149521 | 2.11039457 | 0.03789339 | 0.58953774 | -3.661383 | 2.07600608 |
| TNFRSF13C | 0.599954 | 2.35924961 | 2.11015507 | 0.03796404 | 0.58969559 | -3.6513652 | 2.0752428 |
| NXT2 | 0.26394362 | 3.48875891 | 2.11013751 | 0.03797761 | 0.58969559 | -3.6592658 | 2.07509627 |
| OXCT1-AS1 | 0.328825 | 1.57216681 | 2.10857352 | 0.03798272 | 0.58969559 | -3.6570722 | 2.07504116 |
| LY6L | 1.14689346 | -6.0169035 | 2.10698634 | 0.03812037 | 0.59101875 | -3.6619215 | 2.07355813 |
| GNA14 | -0.479971 | -0.015532 | -2.1069291 | 0.03812086 | 0.59101875 | -3.6571788 | -2.0735529 |
| HCCS | 0.15610474 | 3.75398307 | 2.1066055 | 0.03818057 | 0.59146258 | -3.660671 | 2.07291089 |
| CNN3-DT | 0.42446542 | 4.70121826 | 2.10668297 | 0.03820243 | 0.59146258 | -3.6623827 | 2.07267613 |
| GPATCH1 | -0.1580287 | 4.25815605 | -2.1066944 | 0.038244 | 0.59157876 | -3.6587492 | -2.0722299 |
| PKLR | 1.21941485 | -2.5783381 | 2.11225157 | 0.03828826 | 0.59157876 | -3.647489 | 2.07175542 |
| IFI35 | 0.74502048 | 2.26871835 | 2.10728718 | 0.03829911 | 0.59157876 | -3.6668675 | 2.07163907 |
| AQP7B | 0.6712825 | -0.0372873 | 2.10542334 | 0.03833141 | 0.59157876 | -3.662386 | 2.07129309 |
| SLC2A10 | 0.58689469 | 4.56170152 | 2.10529305 | 0.03838331 | 0.59157876 | -3.6651175 | 2.07073776 |
| ALKBH2 | 0.20235502 | 3.92574793 | 2.10431848 | 0.03841017 | 0.59157876 | -3.6623101 | 2.07045062 |
| B4GALNT1 | -0.2689394 | 6.39755505 | -2.1052383 | 0.03842275 | 0.59157876 | -3.6613292 | -2.0703161 |
| C15orf40 | 0.20180138 | 5.04813013 | 2.10771239 | 0.038446 | 0.59157876 | -3.6596295 | 2.07006773 |
| HSPA2 | 0.61390777 | 3.34726981 | 2.10493044 | 0.03844825 | 0.59157876 | -3.6617397 | 2.07004372 |
| DHX32 | 0.17500721 | 4.85214561 | 2.10447508 | 0.03847714 | 0.59161583 | -3.6640644 | 2.06973528 |
| NALF1 | -0.3679391 | 5.40143031 | -2.1040248 | 0.03851832 | 0.5918416 | -3.6639273 | -2.069296 |
| OXCT1 | -0.2246209 | 6.50349765 | -2.1032725 | 0.03855985 | 0.59207257 | -3.6662097 | -2.0688534 |
| CUX1 | -0.282628 | 7.21470862 | -2.1013973 | 0.03860524 | 0.59236239 | -3.6699063 | -2.0683701 |
| JAK3 | 0.59113508 | 0.91821662 | 2.1010553 | 0.03867676 | 0.59242378 | -3.6662961 | 2.06760948 |
| ACVRL1 | 1.31863442 | -2.7219264 | 2.10131244 | 0.03868587 | 0.59242378 | -3.6644128 | 2.06751272 |
| ZKSCAN7 | -0.2488496 | 3.46825783 | -2.1014108 | 0.03871759 | 0.59242378 | -3.6674523 | -2.0671758 |
| ZNF706 | -0.1284634 | 6.24007476 | -2.1007923 | 0.03875658 | 0.59242378 | -3.6700102 | -2.066762 |
| RNU6-884P | 1.2921382 | -6.2164869 | 2.09861025 | 0.03877727 | 0.59242378 | -3.6763779 | 2.06654266 |
| BNIP3P40 | 1.2822177 | -6.0080593 | 2.09860675 | 0.03880807 | 0.59242378 | -3.6755113 | 2.06621622 |
| NMNAT1 | 0.13491344 | 3.57130762 | 2.10012462 | 0.03884519 | 0.59242378 | -3.67239 | 2.06582304 |
| GRAMD1B | -0.2954694 | 6.30488528 | -2.1002744 | 0.03886197 | 0.59242378 | -3.6696953 | -2.0656455 |
| LRRC74A | 1.22748534 | -5.6153463 | 2.09861497 | 0.03886335 | 0.59242378 | -3.6802732 | 2.06563088 |
| UNGP1 | -1.3676685 | -5.5652148 | -2.0988292 | 0.03888909 | 0.59242378 | -3.6724811 | -2.0653585 |
| TRMO | 0.19870055 | 3.65490422 | 2.09958131 | 0.03890093 | 0.59242378 | -3.6710736 | 2.06523331 |
| SLCO1C1 | 0.91611321 | 7.53256782 | 2.09937169 | 0.03892806 | 0.59243312 | -3.6727462 | 2.06494652 |
| GLMP | 0.60575679 | 3.45053072 | 2.09875406 | 0.03898293 | 0.5924757 | -3.6743198 | 2.06436704 |
| TMEM150A | 0.32314609 | 4.34482457 | 2.09887477 | 0.0389839 | 0.5924757 | -3.6727604 | 2.06435681 |
| PDHA1 | -0.1119973 | 6.16214833 | -2.0983822 | 0.03903399 | 0.59283376 | -3.6736409 | -2.0638284 |
| RETREG1-AS1 | 0.66587729 | -0.7033247 | 2.09545225 | 0.03906358 | 0.59288012 | -3.6797822 | 2.06351647 |
| M1AP | 1.15669039 | -1.4027472 | 2.09692994 | 0.03911217 | 0.5932146 | -3.6806169 | 2.06300477 |
| SLC35E3 | 0.21950803 | 5.73208558 | 2.09664599 | 0.03914072 | 0.59324476 | -3.6774095 | 2.06270444 |
| FGF2 | 0.40193672 | 5.17532474 | 2.09628846 | 0.03918775 | 0.5934768 | -3.6784142 | 2.06221002 |
| TVP23C-CDRT4 | 0.97020476 | -1.1469549 | 2.09520717 | 0.03920916 | 0.5934768 | -3.6758945 | 2.06198508 |
| SOWAHB | 0.49514333 | 0.40799544 | 2.09476851 | 0.03924896 | 0.59367712 | -3.6786064 | 2.06156715 |
| HMGCS1 | -0.4907348 | 8.42638902 | -2.0947955 | 0.03931103 | 0.5938817 | -3.6801032 | -2.0609163 |
| C5AR1 | 0.95631804 | 1.11949259 | 2.0943563 | 0.03937996 | 0.5938817 | -3.6883169 | 2.06019444 |
| KCNQ5 | -0.5104211 | 2.20397627 | -2.0935523 | 0.03940496 | 0.5938817 | -3.6772374 | -2.0599328 |
| KCNK17 | 1.32489754 | -4.9260884 | 2.09376847 | 0.03941329 | 0.5938817 | -3.6819584 | 2.05984576 |
| TCEAL3-AS1 | 1.28440194 | -3.9676657 | 2.09113982 | 0.03947558 | 0.5938817 | -3.6809944 | 2.05919478 |
| CNDP1 | -0.6989517 | -0.0372613 | -2.0916585 | 0.03953968 | 0.5938817 | -3.6797765 | -2.0585259 |
| LINC02384 | 0.81526973 | -1.7622222 | 2.09116425 | 0.03954387 | 0.5938817 | -3.680412 | 2.0584822 |
| H4C5 | -1.51344 | -3.2746424 | -2.0911536 | 0.03958051 | 0.5938817 | -3.6896817 | -2.0581003 |
| BET1 | 0.21457212 | 3.91833142 | 2.09197146 | 0.03958057 | 0.5938817 | -3.6868672 | 2.05809958 |
| ARHGEF37 | 0.30621078 | 3.60684521 | 2.09214815 | 0.03958373 | 0.5938817 | -3.6843861 | 2.05806675 |
| ZNRF2P2 | -0.4428923 | -0.1610077 | -2.0916299 | 0.03964956 | 0.5938817 | -3.6804334 | -2.0573813 |
| KERA | 0.91822483 | -4.7501761 | 2.09114438 | 0.03966305 | 0.5938817 | -3.6887171 | 2.05724097 |
| RNU6-529P | -0.9271779 | -2.8329552 | -2.0893884 | 0.03969398 | 0.5938817 | -3.6912733 | -2.0569194 |
| SMUG1 | -0.148486 | 4.68183548 | -2.0911055 | 0.03970261 | 0.5938817 | -3.6862771 | -2.0568297 |
| LINC01970 | 0.77707828 | -1.6921242 | 2.09065954 | 0.03970375 | 0.5938817 | -3.6815952 | 2.05681788 |
| FEV | -1.5728828 | -4.4390991 | -2.0895294 | 0.03971258 | 0.5938817 | -3.6879002 | -2.0567261 |
| IMMP1L | -0.3048909 | 2.28836921 | -2.089046 | 0.03971439 | 0.5938817 | -3.6892319 | -2.0567073 |
| CRMP1 | -0.2688215 | 9.72400592 | -2.0902928 | 0.03977223 | 0.59434874 | -3.6867724 | -2.0561067 |
| RNA5SP490 | -1.1764974 | -4.1457735 | -2.087122 | 0.03986944 | 0.59473417 | -3.7033055 | -2.055099 |
| CA13 | 0.43780704 | 2.36499442 | 2.08853332 | 0.03990178 | 0.59473417 | -3.6958792 | 2.05476423 |
| ZNF252P-AS1 | 0.46199221 | -0.2960115 | 2.08754727 | 0.03990496 | 0.59473417 | -3.6870722 | 2.05473134 |
| EPHB3 | -0.4437493 | 4.73136615 | -2.0886366 | 0.0399115 | 0.59473417 | -3.6877634 | -2.0546637 |
| CALHM4 | -1.5458479 | -5.7107223 | -2.0904211 | 0.03994952 | 0.59473417 | -3.6881893 | -2.0542704 |
| ZNF286A-TBC1D26 | -1.1206876 | -2.586717 | -2.0845667 | 0.03998998 | 0.59473417 | -3.6907509 | -2.0538524 |
| RNU1-73P | 1.14853474 | -5.9394446 | 2.08373402 | 0.04003591 | 0.59473417 | -3.7071859 | 2.05337817 |
| NUP133 | 0.17570305 | 6.90970598 | 2.08606756 | 0.04008095 | 0.59473417 | -3.6944691 | 2.0529137 |
| PAUPAR | 0.53575458 | -0.8023929 | 2.08772726 | 0.04010212 | 0.59473417 | -3.6925301 | 2.05269549 |
| GAPDHP20 | 1.48111682 | -5.3314251 | 2.08395686 | 0.04017054 | 0.59473417 | -3.6944284 | 2.05199093 |
| GABBR1 | -0.3591604 | 8.85580467 | -2.0853679 | 0.04018784 | 0.59473417 | -3.6949668 | -2.051813 |
| AOX1 | 0.72490469 | 0.5915029 | 2.08549618 | 0.04019374 | 0.59473417 | -3.6972491 | 2.05175235 |
| LINC01285 | 1.23613784 | -1.8563163 | 2.0860651 | 0.04024938 | 0.59473417 | -3.6907286 | 2.05118042 |
| TRAF3IP3 | -1.1144023 | -2.892337 | -2.0813249 | 0.04027855 | 0.59473417 | -3.6978348 | -2.0508808 |
| CDK5R1 | -0.2661717 | 8.29594246 | -2.0845642 | 0.04031267 | 0.59473417 | -3.6955001 | -2.0505307 |
| OGN | -1.661542 | -1.3443358 | -2.0834427 | 0.04032558 | 0.59473417 | -3.6930267 | -2.0503983 |
| ELN | 0.56330376 | 5.32806011 | 2.08403218 | 0.04034646 | 0.59473417 | -3.7005322 | 2.05018418 |
| ADAMTS9-AS2 | 0.80716239 | -0.4939541 | 2.08420546 | 0.04035504 | 0.59473417 | -3.7014906 | 2.05009626 |
| GNG7 | -0.6495689 | 5.37868941 | -2.0842771 | 0.04036564 | 0.59473417 | -3.6938865 | -2.0499876 |
| HEXB | 0.43694272 | 5.37227715 | 2.0836455 | 0.0403911 | 0.59473417 | -3.6991387 | 2.04972674 |
| ANPEP | 1.06394071 | -1.6256874 | 2.08275597 | 0.04045567 | 0.59473417 | -3.6973953 | 2.04906596 |
| TIMM21 | -0.1234453 | 4.8828144 | -2.0826463 | 0.04046318 | 0.59473417 | -3.6996241 | -2.0489891 |
| BCAS2P2 | -1.4996183 | -5.8369176 | -2.0790601 | 0.04047392 | 0.59473417 | -3.7018594 | -2.0488793 |
| CACNA1B | -0.4833085 | 5.37484284 | -2.0826869 | 0.04049012 | 0.59473417 | -3.6946405 | -2.0487137 |
| DDOST | 0.22371988 | 7.39463374 | 2.08231311 | 0.04049226 | 0.59473417 | -3.7002422 | 2.04869177 |
| ADGRG1 | -0.2475451 | 9.17121306 | -2.0830625 | 0.04051401 | 0.59473417 | -3.6992695 | -2.0484696 |
| PRDM2 | -0.1792979 | 6.59241653 | -2.0820798 | 0.04051678 | 0.59473417 | -3.6993563 | -2.0484413 |
| VN1R84P | -1.2288975 | -3.8892901 | -2.0819285 | 0.0405806 | 0.59496947 | -3.6962662 | -2.0477898 |
| CCL28 | 1.23499241 | -3.2793496 | 2.08030353 | 0.04061266 | 0.59496947 | -3.708528 | 2.04746284 |
| CHRAC1 | 0.26556803 | 4.13098102 | 2.08029646 | 0.04068274 | 0.59496947 | -3.701953 | 2.04674898 |
| ZC4H2 | -0.24107 | 5.27686268 | -2.0800231 | 0.04070511 | 0.59496947 | -3.7035242 | -2.0465213 |
| LINC03066 | 1.01367328 | -4.0132796 | 2.07974427 | 0.04071777 | 0.59496947 | -3.7003509 | 2.04639253 |
| HSP90AA2P | -0.6758919 | 0.68118519 | -2.0778857 | 0.04072766 | 0.59496947 | -3.7086592 | -2.0462919 |
| FYB1 | -1.5870595 | -5.1174579 | -2.0787243 | 0.04076384 | 0.59496947 | -3.7032969 | -2.0459241 |
| MAP1LC3C | 0.827135 | -0.0408474 | 2.07940124 | 0.04076474 | 0.59496947 | -3.7132162 | 2.04591498 |
| PRKCQ-AS1 | -0.7591888 | -1.1986998 | -2.0772576 | 0.04080796 | 0.59496947 | -3.7052599 | -2.045476 |
| MBNL1 | 0.19824559 | 5.6163121 | 2.07864202 | 0.04085262 | 0.59496947 | -3.7049806 | 2.04502268 |
| YME1L1 | 0.11278225 | 7.47172528 | 2.07815861 | 0.04088682 | 0.59496947 | -3.7064874 | 2.04467588 |
| C10orf71 | 1.3241569 | -5.3407816 | 2.07633395 | 0.04092688 | 0.59496947 | -3.705228 | 2.04427004 |
| SLC10A4 | 0.29005344 | 3.37046632 | 2.07636919 | 0.04097151 | 0.59496947 | -3.7095603 | 2.04381824 |
| ANXA7 | 0.21865267 | 5.41871241 | 2.0772064 | 0.04098104 | 0.59496947 | -3.7085259 | 2.04372177 |
| ROM1 | 0.49918444 | 2.69772767 | 2.0775454 | 0.04098841 | 0.59496947 | -3.7081678 | 2.04364723 |
| GPCPD1 | -0.3029986 | 5.21743553 | -2.0763218 | 0.04100128 | 0.59496947 | -3.7085827 | -2.0435171 |
| UNC13A | -0.495171 | 7.32456323 | -2.0769897 | 0.04100571 | 0.59496947 | -3.7066588 | -2.0434723 |
| HLA-C | 0.70088269 | 7.3586788 | 2.07680472 | 0.04104049 | 0.59496947 | -3.7100336 | 2.04312073 |
| TMEM192 | 0.2547145 | 5.71636819 | 2.07609018 | 0.04104342 | 0.59496947 | -3.7100391 | 2.04309106 |
| EML3 | 0.46954195 | 4.6179475 | 2.07595597 | 0.04110379 | 0.59496947 | -3.7094603 | 2.04248157 |
| QNG1 | 0.96067211 | -0.8763509 | 2.07566073 | 0.0411114 | 0.59496947 | -3.7150403 | 2.04240477 |
| COPS5P2 | 1.39894008 | -5.488686 | 2.07339271 | 0.0411443 | 0.59496947 | -3.7162552 | 2.04207286 |
| CRYBA4 | 1.41762162 | -5.7269447 | 2.07343036 | 0.04119773 | 0.59496947 | -3.7214387 | 2.04153448 |
| XBP1 | 0.51902156 | 5.92784617 | 2.07457 | 0.0412282 | 0.59496947 | -3.7140106 | 2.04122769 |
| MPHOSPH10P9 | 0.91767169 | -6.5664507 | 2.07313518 | 0.04126286 | 0.59496947 | -3.7171327 | 2.04087894 |
| DUOXA2 | -1.2079275 | -4.027478 | -2.0725361 | 0.04133631 | 0.59496947 | -3.7115942 | -2.0401408 |
| LIMA1 | 0.24964809 | 5.65404862 | 2.07362547 | 0.04134553 | 0.59496947 | -3.7146666 | 2.04004817 |
| HOXC4 | -1.3859475 | -5.8694686 | -2.0720361 | 0.04134975 | 0.59496947 | -3.714526 | -2.0400058 |
| PLD2 | 0.5080755 | 5.37638017 | 2.07340312 | 0.04135758 | 0.59496947 | -3.7137797 | 2.03992716 |
| TAS2R4 | -0.492674 | 0.36952885 | -2.0717265 | 0.0413632 | 0.59496947 | -3.7135989 | -2.0398707 |
| DRAXIN | -0.3392348 | 7.17194941 | -2.072791 | 0.04141211 | 0.59496947 | -3.7144449 | -2.03938 |
| AKAP1-DT | 0.32387954 | 1.0987997 | 2.07273181 | 0.04142387 | 0.59496947 | -3.7157305 | 2.03926212 |
| HIRIP3 | -0.3066436 | 4.74997318 | -2.0722003 | 0.04142942 | 0.59496947 | -3.7150645 | -2.0392065 |
| EVX2 | -1.7488908 | -5.3385271 | -2.0708189 | 0.0414663 | 0.59496947 | -3.7151034 | -2.0388369 |
| TSPAN5 | -0.2461287 | 6.83633534 | -2.0702816 | 0.04150921 | 0.59496947 | -3.7188071 | -2.0384073 |
| HLA-G | 1.23111469 | -4.1725592 | 2.07093477 | 0.0415799 | 0.59496947 | -3.724327 | 2.03770049 |
| INTS6P1 | 1.33470389 | -5.78904 | 2.06818887 | 0.04161801 | 0.59496947 | -3.7233168 | 2.03731974 |
| CCDC15-DT | -0.2606999 | 1.22831491 | -2.0700223 | 0.04161962 | 0.59496947 | -3.7189976 | -2.0373036 |
| RNU4-52P | 1.32310384 | -4.9296685 | 2.06877102 | 0.04162136 | 0.59496947 | -3.7225536 | 2.03728631 |
| GPC2 | -0.2819062 | 6.98648635 | -2.0707403 | 0.0416216 | 0.59496947 | -3.7163264 | -2.0372839 |
| LINC01858 | 1.27896277 | -5.781043 | 2.06807195 | 0.04166964 | 0.59496947 | -3.7194937 | 2.03680445 |
| PIK3AP1 | 0.48106027 | 0.93319106 | 2.06934592 | 0.0416738 | 0.59496947 | -3.716662 | 2.03676299 |
| GYG2 | 0.34342565 | 5.74567338 | 2.06998539 | 0.04167796 | 0.59496947 | -3.7223347 | 2.03672152 |
| NINJ2-AS1 | 0.5252873 | 0.60750845 | 2.06896232 | 0.0417598 | 0.59522023 | -3.7259591 | 2.03590596 |
| PHF20L1 | -0.1601875 | 6.53717085 | -2.0682878 | 0.04177291 | 0.59522023 | -3.7215238 | -2.0357754 |
| RNU6-689P | 1.22156411 | -3.6549613 | 2.06657715 | 0.04177545 | 0.59522023 | -3.7254846 | 2.03575016 |
| SMCO2 | -0.8663568 | -2.8601102 | -2.0662169 | 0.04188254 | 0.59581354 | -3.7354031 | -2.0346854 |
| NCDN | -0.2990757 | 7.27939695 | -2.06737 | 0.04194375 | 0.59581354 | -3.7226034 | -2.0340778 |
| NREP | -0.2962826 | 6.1011131 | -2.0671803 | 0.04194911 | 0.59581354 | -3.7217905 | -2.0340247 |
| SHANK2-AS1 | -1.5785114 | -4.6911538 | -2.0632768 | 0.04198367 | 0.59581354 | -3.7317427 | -2.033682 |
| VAV3-AS1 | 1.20348973 | -5.2010627 | 2.06596465 | 0.04200541 | 0.59581354 | -3.7317206 | 2.03346659 |
| ROBO1 | -0.2172001 | 8.57917727 | -2.0655472 | 0.04210392 | 0.59581354 | -3.7263945 | -2.0324915 |
| MATN1-AS1 | 0.80894555 | -1.0112392 | 2.06346432 | 0.04210633 | 0.59581354 | -3.7252434 | 2.03246768 |
| IRF7 | 0.38266197 | 3.59633664 | 2.0652411 | 0.04214969 | 0.59581354 | -3.7296701 | 2.03203916 |
| ADAT3 | 0.34259383 | 1.94023245 | 2.06315008 | 0.04218318 | 0.59581354 | -3.7288028 | 2.0317085 |
| IGHG4 | 1.32334199 | -4.2696312 | 2.06413514 | 0.04220718 | 0.59581354 | -3.7237966 | 2.03147155 |
| EPB41L3 | 0.40318325 | 7.65944996 | 2.06434067 | 0.04221793 | 0.59581354 | -3.7291668 | 2.03136553 |
| CITED1 | -0.495967 | 0.61703456 | -2.0621358 | 0.04222512 | 0.59581354 | -3.7317079 | -2.0312946 |
| TEK | -0.6682661 | -0.1035642 | -2.0639006 | 0.04223501 | 0.59581354 | -3.7261664 | -2.031197 |
| PRDX4 | 0.39635814 | 5.0794939 | 2.06378908 | 0.04226588 | 0.59581354 | -3.7315472 | 2.03089267 |
| MVP | 0.51179596 | 4.97711851 | 2.06375055 | 0.04231612 | 0.59581354 | -3.7315246 | 2.03039783 |
| KALRN | -0.3309231 | 7.04180635 | -2.062725 | 0.04231705 | 0.59581354 | -3.730482 | -2.0303886 |
| LRRC14B | 1.17407386 | -0.6598491 | 2.06189312 | 0.04236632 | 0.59581354 | -3.7335006 | 2.02990379 |
| TBX6 | -0.7566504 | -0.6175834 | -2.0614539 | 0.04240328 | 0.59581354 | -3.7295905 | -2.0295404 |
| CYB5RL | 0.25385308 | 3.21601213 | 2.06121035 | 0.04245722 | 0.59581354 | -3.7336638 | 2.02901055 |
| AK3 | 0.18545055 | 6.49645368 | 2.06199869 | 0.04246793 | 0.59581354 | -3.7320418 | 2.02890534 |
| ACSL5 | 0.62463766 | 0.42776388 | 2.06084572 | 0.0424855 | 0.59581354 | -3.7315919 | 2.02873293 |
| GSTK1 | 0.17555825 | 5.68220411 | 2.06150451 | 0.0424897 | 0.59581354 | -3.7330639 | 2.02869169 |
| MGAT4B | 0.23471243 | 7.6320646 | 2.06051952 | 0.04249523 | 0.59581354 | -3.7348912 | 2.0286375 |
| IL7 | 0.54290745 | 1.02299778 | 2.06234324 | 0.04252558 | 0.59581354 | -3.7303503 | 2.02833976 |
| EPS15-AS1 | 1.36890867 | -4.9707788 | 2.05858023 | 0.04255098 | 0.59581354 | -3.7391413 | 2.02809081 |
| TOP2B | -0.2105746 | 9.13753055 | -2.0599699 | 0.04260214 | 0.59581354 | -3.7351262 | -2.0275897 |
| TXNIP | -0.5632321 | 5.81485457 | -2.0588867 | 0.04262241 | 0.59581354 | -3.7346497 | -2.0273913 |
| SIRPB1 | -1.1098466 | -3.897464 | -2.0587145 | 0.04264009 | 0.59581354 | -3.7315983 | -2.0272184 |
| TNNT1 | -0.5319936 | 1.34122128 | -2.0599452 | 0.04266931 | 0.59581354 | -3.7291138 | -2.0269325 |
| ACVR2B | -0.2845538 | 5.90526219 | -2.0599713 | 0.04266951 | 0.59581354 | -3.733633 | -2.0269306 |
| P3H2-AS1 | 0.7529412 | -1.4360367 | 2.05995687 | 0.0426795 | 0.59581354 | -3.7328246 | 2.02683294 |
| PPP1R13B-DT | -1.5723582 | -4.3713684 | -2.0582438 | 0.04269782 | 0.59581354 | -3.730603 | -2.0266539 |
| SGCE | 0.32817259 | 6.1576896 | 2.05948282 | 0.04271015 | 0.59581354 | -3.7360464 | 2.02653341 |
| TRPV2 | 0.59285449 | -0.8710508 | 2.05765833 | 0.04272384 | 0.59581354 | -3.7386737 | 2.02639972 |
| SHISA8 | -0.5897745 | 2.14993043 | -2.0590963 | 0.04275267 | 0.59584364 | -3.7291775 | -2.0261183 |
| MPZL1 | 0.26041561 | 7.5982373 | 2.0577195 | 0.04279259 | 0.59602819 | -3.739576 | 2.02572876 |
| ICAM5 | -0.5048126 | 4.15218025 | -2.0573821 | 0.04291296 | 0.59705381 | -3.7329 | -2.0245562 |
| MTARC1 | 0.34560116 | 3.84166594 | 2.05660197 | 0.04293338 | 0.59705381 | -3.7400395 | 2.02435752 |
| ESYT2 | 0.22228339 | 6.3648179 | 2.05688879 | 0.0429464 | 0.59705381 | -3.7395385 | 2.02423092 |
| FAM181B | -0.4056721 | 5.46224013 | -2.0557655 | 0.04303545 | 0.59791983 | -3.7438238 | -2.0233657 |
| MYH10 | -0.2104148 | 8.48060776 | -2.0518801 | 0.04310372 | 0.59811468 | -3.7496091 | -2.0227036 |
| CUL7 | 0.15493871 | 6.03731171 | 2.05556086 | 0.04311455 | 0.59811468 | -3.741184 | 2.0225986 |
| DHRS1 | 0.25184715 | 4.74357119 | 2.05578744 | 0.04312979 | 0.59811468 | -3.7406599 | 2.02245086 |
| ZBED3-AS1 | 0.42388324 | 3.54599067 | 2.05357829 | 0.04324889 | 0.5993942 | -3.7451412 | 2.02129835 |
| KIF4B | -1.3352476 | -3.5936756 | -2.0520793 | 0.0432933 | 0.59953777 | -3.7428863 | -2.0208692 |
| CAAP1 | 0.17425634 | 4.45454642 | 2.05308751 | 0.0433257 | 0.59953777 | -3.7442423 | 2.0205565 |
| ULBP1 | 0.41468994 | 1.61203142 | 2.05234925 | 0.043366 | 0.59953777 | -3.7452442 | 2.02016763 |
| SYT8 | 1.67511925 | -6.1246059 | 2.04956154 | 0.04336659 | 0.59953777 | -3.7556413 | 2.02016195 |
| ARID5B | 0.36999493 | 5.33354152 | 2.05127943 | 0.0434045 | 0.59957803 | -3.750411 | 2.0197965 |
| TMCO4 | 0.61925054 | 1.39461669 | 2.05237718 | 0.04342954 | 0.59957803 | -3.7484553 | 2.01955523 |
| RELL1 | 0.40746521 | 4.1595301 | 2.0515978 | 0.04345002 | 0.59957803 | -3.7521328 | 2.01935806 |
| LINC01979 | 0.56362206 | -0.659015 | 2.05171915 | 0.04352531 | 0.60024629 | -3.7404947 | 2.01863364 |
| NCAM1 | -0.3724713 | 9.48811105 | -2.0505939 | 0.04359019 | 0.60050167 | -3.7494031 | -2.0180103 |
| UBA3 | -0.0997445 | 5.87276119 | -2.0493805 | 0.04362485 | 0.60050167 | -3.7523238 | -2.0176776 |
| IGFBP4 | 0.35136548 | 6.41840771 | 2.05033604 | 0.04362622 | 0.60050167 | -3.7505307 | 2.01766445 |
| IQCB1 | 0.18290057 | 4.79368586 | 2.04901999 | 0.04367112 | 0.60050167 | -3.7511837 | 2.01723376 |
| CELF6 | -0.9187092 | -3.2436929 | -2.0496484 | 0.04367822 | 0.60050167 | -3.7496386 | -2.0171657 |
| LINC02773 | 1.25566398 | -3.9160984 | 2.04768455 | 0.04382419 | 0.60213787 | -3.7602935 | 2.01576851 |
| CPD | 0.46710475 | 6.67871901 | 2.04619671 | 0.04392924 | 0.60236431 | -3.7576914 | 2.0147654 |
| EMSY | -0.19341 | 5.69641713 | -2.0467932 | 0.04393172 | 0.60236431 | -3.7537237 | -2.0147417 |
| CADM1 | 0.29423907 | 8.74610311 | 2.04673271 | 0.04394728 | 0.60236431 | -3.7557632 | 2.01459325 |
| EPN2 | -0.2244288 | 5.55802264 | -2.0468591 | 0.04397551 | 0.60236431 | -3.7554936 | -2.0143242 |
| RPS26P45 | 1.54244005 | -5.7885779 | 2.04264676 | 0.0440443 | 0.60236431 | -3.7567163 | 2.01366895 |
| ZNF271P | -0.1675123 | 5.75175614 | -2.0449905 | 0.04410494 | 0.60236431 | -3.7587591 | -2.0130922 |
| WFDC3 | -0.741777 | -1.5154516 | -2.0441946 | 0.0441636 | 0.60236431 | -3.7613273 | -2.0125347 |
| PGC | 1.51504615 | -4.146028 | 2.04313302 | 0.04417014 | 0.60236431 | -3.7558013 | 2.01247265 |
| HK3 | -1.0302696 | -2.519865 | -2.0441963 | 0.04418292 | 0.60236431 | -3.7562291 | -2.0123513 |
| TET3 | -0.282038 | 7.02491202 | -2.0442813 | 0.04418437 | 0.60236431 | -3.7588734 | -2.0123375 |
| ZNF691-DT | 0.54440094 | 0.15279603 | 2.04415368 | 0.04421988 | 0.60236431 | -3.7615229 | 2.01200055 |
| TPM4P2 | 0.55105287 | -0.3567567 | 2.04222393 | 0.04436367 | 0.60236431 | -3.7685097 | 2.01063831 |
| ANKRD20A4P | -1.3587173 | -5.6251009 | -2.0420461 | 0.04438583 | 0.60236431 | -3.7657466 | -2.0104287 |
| NEK1 | -0.1809094 | 5.54927099 | -2.041364 | 0.04443475 | 0.60236431 | -3.7637582 | -2.0099663 |
| PLAAT4 | 0.68854962 | 2.07879982 | 2.04199254 | 0.04446942 | 0.60236431 | -3.7719283 | 2.00963889 |
| PECAM1 | 1.06275408 | -1.6396152 | 2.04335732 | 0.04447706 | 0.60236431 | -3.7753952 | 2.0095668 |
| RBM7 | 0.28220241 | 4.17025558 | 2.0415049 | 0.04449887 | 0.60236431 | -3.7690564 | 2.00936095 |
| CDH3-AS1 | -1.1638643 | -3.1926401 | -2.040208 | 0.04451343 | 0.60236431 | -3.7609061 | -2.0092235 |
| LINC02172 | 1.40422429 | -5.9660264 | 2.0393081 | 0.0445387 | 0.60236431 | -3.7728019 | 2.00898522 |
| TACSTD2 | -1.5236521 | -3.5571483 | -2.0400063 | 0.04455586 | 0.60236431 | -3.7589351 | -2.0088235 |
| COLGALT2 | -0.4984628 | 6.17269414 | -2.0401021 | 0.04456536 | 0.60236431 | -3.7679621 | -2.0087339 |
| TRIML2 | 1.66484163 | -5.5104535 | 2.03981662 | 0.04458725 | 0.60236431 | -3.7784948 | 2.00852762 |
| HECTD4 | -0.3478041 | 8.21560801 | -2.0404886 | 0.04462456 | 0.60236431 | -3.7651021 | -2.0081763 |
| CD164 | 0.35008769 | 6.7523353 | 2.04024501 | 0.04463178 | 0.60236431 | -3.7664452 | 2.00810832 |
| SPDYE19P | 1.14505177 | -3.482973 | 2.03818977 | 0.0446401 | 0.60236431 | -3.7654179 | 2.00802999 |
| NDRG2 | -0.3651978 | 7.34293317 | -2.0396318 | 0.04464807 | 0.60236431 | -3.7677621 | -2.0079551 |
| RPS27P2 | 1.3446222 | -4.1117931 | 2.0366247 | 0.0446612 | 0.60236431 | -3.7766732 | 2.00783146 |
| ZSCAN32 | 0.15889162 | 4.07668855 | 2.03950736 | 0.04470251 | 0.60236431 | -3.7644837 | 2.007443 |
| FGF19 | -2.0550586 | -3.5606574 | -2.0361724 | 0.04475531 | 0.60236431 | -3.7708794 | -2.006947 |
| IGFBP1 | 1.39717753 | -5.437719 | 2.03734283 | 0.04476776 | 0.60236431 | -3.7717678 | 2.00683005 |
| LINC00907 | 0.90386573 | 0.48374652 | 2.03732113 | 0.04479943 | 0.60236431 | -3.7741404 | 2.00653286 |
| LONRF3 | -0.5442789 | 1.42792719 | -2.0388577 | 0.044811 | 0.60236431 | -3.7652792 | -2.0064243 |
| ADAMTSL5 | -0.6600669 | -1.0089033 | -2.0368406 | 0.04487847 | 0.60236431 | -3.7672888 | -2.0057918 |
| ST13P15 | 0.9549514 | -3.2387701 | 2.03496152 | 0.04491558 | 0.60236431 | -3.788094 | 2.00544425 |
| CBFA2T3 | -0.2960148 | 4.57980048 | -2.0372323 | 0.04494934 | 0.60236431 | -3.765914 | -2.0051283 |
| EIF2AK4 | 0.20188222 | 6.05052107 | 2.03622816 | 0.04495781 | 0.60236431 | -3.7724605 | 2.00504903 |
| C1orf54 | 0.4834887 | 2.64785297 | 2.03671112 | 0.04495977 | 0.60236431 | -3.7782505 | 2.00503065 |
| TMEM216 | 0.29159694 | 3.25228122 | 2.03626521 | 0.04496227 | 0.60236431 | -3.7695316 | 2.00500725 |
| BST2 | 0.88111712 | 1.55439434 | 2.03846719 | 0.04500302 | 0.60236431 | -3.7665464 | 2.00462627 |
| PGRMC2 | 0.24643108 | 6.33813593 | 2.03587217 | 0.0450108 | 0.60236431 | -3.7732086 | 2.00455353 |
| TPM4 | 0.17102319 | 7.65048717 | 2.03546558 | 0.0450586 | 0.60236431 | -3.7737291 | 2.004107 |
| DNAJB6P8 | -1.2342266 | -6.5470774 | -2.0328242 | 0.04505875 | 0.60236431 | -3.7821581 | -2.0041056 |
| SGPL1 | 0.19623027 | 6.64415905 | 2.0359674 | 0.04506076 | 0.60236431 | -3.7729383 | 2.00408683 |
| LINS1 | -0.1563136 | 4.03615934 | -2.0346919 | 0.04511692 | 0.60236431 | -3.7722436 | -2.0035628 |
| TATDN1P1 | 1.44593344 | -4.9527811 | 2.03201019 | 0.04511796 | 0.60236431 | -3.7762317 | 2.00355301 |
| CBR3 | 0.65434852 | -1.077734 | 2.03596621 | 0.0451483 | 0.60236431 | -3.7758577 | 2.00327016 |
| NUDT18 | 0.25913564 | 2.69176702 | 2.0351269 | 0.04517083 | 0.60236431 | -3.7725793 | 2.00306021 |
| H3-7 | -0.6930017 | -0.5457301 | -2.0364389 | 0.04518815 | 0.60236431 | -3.7644352 | -2.0028988 |
| FLVCR2 | 0.81545305 | 0.71468491 | 2.03429126 | 0.04519498 | 0.60236431 | -3.7853671 | 2.00283519 |
| CLEC2B | 1.2348564 | -2.2572802 | 2.0337358 | 0.04520676 | 0.60236431 | -3.7779214 | 2.00272548 |
| LINC01250 | -1.2505644 | -3.8971052 | -2.033304 | 0.04523897 | 0.60236431 | -3.7749451 | -2.0024257 |
| PMF1 | 0.19915446 | 3.82839095 | 2.034553 | 0.04524271 | 0.60236431 | -3.7756249 | 2.0023909 |
| TMEM167B-DT | 0.28280496 | 1.15976083 | 2.03309458 | 0.04536601 | 0.6036463 | -3.7811569 | 2.0012448 |
| KLF9 | 0.51732041 | 5.4533292 | 2.03258492 | 0.04546731 | 0.60443795 | -3.7736323 | 2.00030528 |
| BNIP3P30 | 1.3002085 | -5.2504462 | 2.03066636 | 0.04547962 | 0.60443795 | -3.7731164 | 2.00019122 |
| ADAM3B | -1.239608 | -6.1097664 | -2.0294857 | 0.0455204 | 0.60462024 | -3.7909602 | -1.9998136 |
| IL16 | -0.4964982 | 0.67673964 | -2.0309415 | 0.0455512 | 0.60466984 | -3.7826752 | -1.9995285 |
| ACTL6B | -0.3412294 | 5.67858573 | -2.0302117 | 0.04566856 | 0.6055466 | -3.7784739 | -1.9984438 |
| PSMD7 | 0.16662714 | 5.23102661 | 2.02826541 | 0.04568374 | 0.6055466 | -3.7858814 | 1.99830378 |
| SMIM2-AS1 | -0.8687289 | -2.5265461 | -2.0287558 | 0.04569856 | 0.6055466 | -3.7936793 | -1.998167 |
| PCNT | -0.1790951 | 7.01070525 | -2.0290602 | 0.04574179 | 0.60566866 | -3.7828617 | -1.9977683 |
| SLC26A2 | 0.27140084 | 4.56547041 | 2.02895723 | 0.04577653 | 0.60566866 | -3.7826318 | 1.99744806 |
| SHC1 | 0.56140257 | 5.59383625 | 2.02882298 | 0.0457891 | 0.60566866 | -3.7858664 | 1.99733223 |
| GPX4 | 0.22463437 | 7.39649469 | 2.02800871 | 0.04585023 | 0.6060145 | -3.7881697 | 1.99676952 |
| GJA3 | 1.77522275 | -3.880047 | 2.02676898 | 0.04588258 | 0.6060145 | -3.7842778 | 1.99647194 |
| YIPF1 | 0.21703345 | 4.84535205 | 2.0270241 | 0.04589663 | 0.6060145 | -3.7873492 | 1.99634276 |
| AARS2 | -0.2167484 | 5.06239034 | -2.0276854 | 0.0459255 | 0.60603763 | -3.783021 | -1.9960773 |
| ARHGEF7 | -0.1569981 | 7.87085016 | -2.0273431 | 0.04596693 | 0.60617326 | -3.7850196 | -1.9956968 |
| HDGFL3 | -0.2065984 | 7.7510766 | -2.0270949 | 0.04599858 | 0.60617326 | -3.785819 | -1.9954064 |
| NEURL4 | -0.1744766 | 6.43297777 | -2.0269958 | 0.04601718 | 0.60617326 | -3.7848772 | -1.9952357 |
| TLK2P1 | 0.56656913 | -1.6604389 | 2.02407376 | 0.046079 | 0.60662992 | -3.7936057 | 1.99466892 |
| MDFIC2 | -1.0574574 | -6.5197868 | -2.0250612 | 0.04613737 | 0.60697599 | -3.7884467 | -1.9941344 |
| NEU4 | 0.48534095 | 0.42506808 | 2.02528782 | 0.04623281 | 0.60697599 | -3.7814968 | 1.99326157 |
| RUNX2 | 0.61640028 | 2.30362931 | 2.02424103 | 0.04627352 | 0.60697599 | -3.7937114 | 1.99288974 |
| DNAH3 | -0.7294455 | -1.8217809 | -2.0239071 | 0.04628421 | 0.60697599 | -3.7928659 | -1.9927922 |
| NHERF4 | -1.1823369 | -3.296247 | -2.023176 | 0.04628832 | 0.60697599 | -3.7894113 | -1.9927547 |
| MYLK3 | 0.54467611 | 2.73738227 | 2.02404498 | 0.04629466 | 0.60697599 | -3.7967541 | 1.99269682 |
| CHD9 | -0.1810128 | 7.13582151 | -2.0228286 | 0.04632166 | 0.60697599 | -3.7943025 | -1.9924504 |
| MMP15 | -0.2845613 | 6.95353269 | -2.0231778 | 0.04635386 | 0.60697599 | -3.7926809 | -1.9921568 |
| NAGLU | 0.46338045 | 5.38030422 | 2.02344996 | 0.04637653 | 0.60697599 | -3.7941199 | 1.99195016 |
| KMT2D | -0.2698983 | 7.64917354 | -2.0222606 | 0.04641393 | 0.60697599 | -3.7941964 | -1.9916094 |
| RAVER2 | 0.26693549 | 5.56218169 | 2.02202775 | 0.04646616 | 0.60697599 | -3.7961247 | 1.99113397 |
| CNTNAP1 | 0.29533757 | 6.61055219 | 2.02125119 | 0.04649531 | 0.60697599 | -3.7962975 | 1.99086887 |
| TRAPPC5 | -0.2764035 | 2.79453819 | -2.0221545 | 0.04649558 | 0.60697599 | -3.790562 | -1.9908664 |
| HSP90AB2P | -1.06275 | -2.6074593 | -2.0199496 | 0.04650953 | 0.60697599 | -3.7936113 | -1.9907396 |
| DOLK | 0.24128898 | 4.89059692 | 2.02121415 | 0.04657823 | 0.60697599 | -3.7965285 | 1.99011538 |
| C17orf67 | -0.2466907 | 2.11798087 | -2.0210647 | 0.04661162 | 0.60697599 | -3.7905356 | -1.9898122 |
| LINC01821 | -1.1846307 | -4.9110184 | -2.0196199 | 0.04666653 | 0.60697599 | -3.7968399 | -1.9893142 |
| LINC02575 | -0.7772066 | -0.7820473 | -2.0207979 | 0.04671945 | 0.60697599 | -3.787622 | -1.9888347 |
| KLF12 | -0.3554555 | 7.13413987 | -2.0196312 | 0.04675263 | 0.60697599 | -3.7979277 | -1.9885343 |
| FABP5 | 0.25677641 | 3.79011616 | 2.01962114 | 0.04676236 | 0.60697599 | -3.8044045 | 1.98844619 |
| TP53 | 0.34670222 | 5.59534137 | 2.01950208 | 0.04678289 | 0.60697599 | -3.7994536 | 1.98826048 |
| FBXO7 | 0.11531916 | 6.49946074 | 2.01953745 | 0.04679207 | 0.60697599 | -3.7979695 | 1.98817739 |
| TEX15 | -0.6830687 | 1.47929709 | -2.0186574 | 0.04680577 | 0.60697599 | -3.7995148 | -1.9880536 |
| ZNF250 | -0.1510053 | 4.84063726 | -2.0185394 | 0.04689686 | 0.60697599 | -3.7963189 | -1.9872305 |
| LRRC8C | 0.46129122 | 4.5173791 | 2.01810703 | 0.04689837 | 0.60697599 | -3.8037588 | 1.98721683 |
| RPL26 | -0.2838996 | 2.78390452 | -2.0167778 | 0.0469109 | 0.60697599 | -3.8023727 | -1.9871038 |
| PDE10A | 0.45181687 | 7.52675362 | 2.01791042 | 0.04691455 | 0.60697599 | -3.8002817 | 1.9870708 |
| ZFP69B | -0.2642898 | 4.05203303 | -2.0176864 | 0.0469665 | 0.60697599 | -3.7938529 | -1.9866022 |
| EHMT2 | -0.1691511 | 7.29190546 | -2.0174769 | 0.04698964 | 0.60697599 | -3.8007299 | -1.9863935 |
| MIR6867 | 1.21053572 | -5.4331332 | 2.01448204 | 0.04701209 | 0.60697599 | -3.8009632 | 1.98619128 |
| SNORD115-25 | -1.143891 | -6.2154134 | -2.0165073 | 0.04701587 | 0.60697599 | -3.8062737 | -1.9861572 |
| TMEM223 | 0.19413642 | 3.62292411 | 2.01724284 | 0.04701612 | 0.60697599 | -3.8024498 | 1.98615501 |
| PRICKLE3 | 0.71925561 | 0.56477029 | 2.01681205 | 0.04704117 | 0.60697599 | -3.8030977 | 1.9859293 |
| SPHK1 | 0.82531187 | 2.61009484 | 2.01617793 | 0.04706315 | 0.60697599 | -3.8027149 | 1.98573144 |
| FOXRED2 | 0.18176077 | 6.69494096 | 2.0163273 | 0.0470823 | 0.60697599 | -3.8031662 | 1.98555913 |
| TNIP2 | 0.2390649 | 4.51839411 | 2.01637338 | 0.04709175 | 0.60697599 | -3.8048517 | 1.98547406 |
| FMNL3 | -0.2567592 | 4.95949114 | -2.0182793 | 0.0471187 | 0.60697599 | -3.7995222 | -1.9852317 |
| EXPH5 | 0.53558118 | 2.5311424 | 2.01625708 | 0.0471377 | 0.60697599 | -3.805866 | 1.98506087 |
| RPS27AP7 | -1.5261169 | -6.0350186 | -2.011823 | 0.04730875 | 0.60841338 | -3.8094044 | -1.9835256 |
| KIAA1217 | 0.54229336 | 4.24703741 | 2.01392794 | 0.04736616 | 0.60841338 | -3.8081844 | 1.98301136 |
| PRECSIT | 0.70437323 | 0.83895094 | 2.01204254 | 0.04742556 | 0.60841338 | -3.8059353 | 1.98247986 |
| STMN4 | -0.4295225 | 7.13641196 | -2.0134171 | 0.0474363 | 0.60841338 | -3.8059427 | -1.9823838 |
| GXYLT1P4 | -0.9804075 | -3.0033821 | -2.0122413 | 0.04744734 | 0.60841338 | -3.8036313 | -1.9822851 |
| NLRC4 | 0.69959334 | -2.1810029 | 2.01017263 | 0.04747631 | 0.60841338 | -3.808379 | 1.98202621 |
| GPER1 | -0.5476367 | 0.57233316 | -2.0124097 | 0.04747634 | 0.60841338 | -3.8090179 | -1.9820259 |
| TRIM9 | -0.245861 | 7.18519202 | -2.0119555 | 0.04755293 | 0.60841338 | -3.8092796 | -1.981342 |
| LDLRAP1 | 0.59675457 | 2.31035491 | 2.01184708 | 0.04756731 | 0.60841338 | -3.8127278 | 1.98121372 |
| PAFAH2 | 0.21230925 | 3.14525817 | 2.01213691 | 0.04756756 | 0.60841338 | -3.81737 | 1.98121151 |
| BANF1P2 | 0.95718013 | -1.4920333 | 2.01207139 | 0.04756867 | 0.60841338 | -3.7978226 | 1.9812016 |
| IGBP1P1 | 1.49110903 | -4.2018758 | 2.00945525 | 0.04760685 | 0.60841338 | -3.8093252 | 1.98086115 |
| ACE | 0.36995284 | 1.86555208 | 2.01126139 | 0.04762536 | 0.60841338 | -3.8060286 | 1.98069618 |
| P2RY14 | 1.27986901 | -4.238292 | 2.01005631 | 0.04763628 | 0.60841338 | -3.8091457 | 1.98059886 |
| SLC7A8 | 0.56648415 | 4.89978426 | 2.01027308 | 0.04765782 | 0.60841338 | -3.8118413 | 1.98040692 |
| MALT1-AS1 | 0.66653597 | -1.1988548 | 2.00851076 | 0.04779624 | 0.609832 | -3.8183677 | 1.97917558 |
| BNAT1 | 0.82524268 | -0.370286 | 2.00969319 | 0.04785033 | 0.60998663 | -3.8169559 | 1.9786952 |
| NPY1R | 0.49798497 | 3.68169304 | 2.00913477 | 0.04786297 | 0.60998663 | -3.8097045 | 1.97858306 |
| GABRA6 | 1.05647334 | -2.8929486 | 2.00841639 | 0.04792854 | 0.61024705 | -3.8049715 | 1.97800146 |
| SERTM2 | -0.6807431 | -0.5894299 | -2.008386 | 0.04795238 | 0.61024705 | -3.8187014 | -1.9777902 |
| SRC | -0.1523948 | 7.13105467 | -2.0085081 | 0.04796535 | 0.61024705 | -3.814006 | -1.9776753 |
| REXO2 | 0.25332573 | 4.3672951 | 2.0076054 | 0.04806693 | 0.61101606 | -3.8181575 | 1.97677624 |
| OTUB2 | -0.2580436 | 2.88011548 | -2.007052 | 0.04811978 | 0.61101606 | -3.8138835 | -1.9763091 |
| HOXC13 | -1.2406922 | -6.4834586 | -2.0059798 | 0.04816181 | 0.61101606 | -3.8196339 | -1.9759379 |
| RBM48 | -0.1749706 | 4.01234753 | -2.0052461 | 0.04822677 | 0.61101606 | -3.815838 | -1.9753647 |
| DLC1 | -0.4266522 | 2.64678003 | -2.0054458 | 0.0482441 | 0.61101606 | -3.8162646 | -1.975212 |
| TMEM154 | 0.4276531 | 1.35147629 | 2.00572371 | 0.04828044 | 0.61101606 | -3.8260975 | 1.97489167 |
| SLIT1 | -0.3751174 | 8.32077031 | -2.0052216 | 0.04831077 | 0.61101606 | -3.8189682 | -1.9746245 |
| LINC01165 | 0.51006437 | -0.3057201 | 2.00579756 | 0.04833526 | 0.61101606 | -3.8166961 | 1.974409 |
| SLC35B1 | 0.18787867 | 6.00446628 | 2.00496617 | 0.04836641 | 0.61101606 | -3.821018 | 1.9741349 |
| UACA | 0.57160515 | 4.51592829 | 2.00377266 | 0.04842806 | 0.61101606 | -3.8270272 | 1.97359283 |
| PDGFB | 0.53011304 | 2.70838522 | 2.00391081 | 0.04843825 | 0.61101606 | -3.8199144 | 1.97350327 |
| HMCN2 | 0.92683335 | 2.73271864 | 2.00358383 | 0.04843955 | 0.61101606 | -3.823847 | 1.97349189 |
| HMGCL | 0.22413866 | 4.23736733 | 2.00423994 | 0.04844651 | 0.61101606 | -3.8247637 | 1.9734307 |
| CPNE6 | -0.9461229 | 1.60914502 | -2.0031901 | 0.04846534 | 0.61101606 | -3.8187832 | -1.9732653 |
| ZDHHC14 | -0.2308627 | 5.9144842 | -2.0030423 | 0.04848545 | 0.61101606 | -3.8238402 | -1.9730888 |
| GPR89A | 0.17170707 | 3.24111491 | 2.00326034 | 0.04850966 | 0.61101606 | -3.8224552 | 1.97287629 |
| ADGRL4 | 1.09178329 | -0.7614731 | 2.00216989 | 0.04862346 | 0.61101606 | -3.829228 | 1.9718787 |
| FRMD8 | 0.19453347 | 4.9129495 | 2.00246251 | 0.0486307 | 0.61101606 | -3.8256284 | 1.97181523 |
| TNS4 | 1.30090011 | -6.5177488 | 1.99945223 | 0.04867715 | 0.61101606 | -3.8303391 | 1.97140868 |
| MIR4292 | -0.435013 | 0.31474971 | -2.0011753 | 0.04869105 | 0.61101606 | -3.8209691 | -1.9712871 |
| PRDM11 | -0.230825 | 5.02533273 | -2.0011971 | 0.04870952 | 0.61101606 | -3.8236622 | -1.9711255 |
| DNAJB5-DT | -1.2514096 | -4.3667374 | -2.0001562 | 0.04872529 | 0.61101606 | -3.8303346 | -1.9709876 |
| HLA-DOA | 1.12274466 | -0.4565724 | 2.00135212 | 0.04873308 | 0.61101606 | -3.8241943 | 1.97091953 |
| LINC02596 | -1.1562034 | -6.4846001 | -1.9993546 | 0.04880439 | 0.61101606 | -3.8270283 | -1.9702966 |
| HCN4 | -0.3478069 | 5.61767499 | -2.0003271 | 0.04882188 | 0.61101606 | -3.8255917 | -1.9701439 |
| H2AC8 | -1.0539966 | -2.7224853 | -1.9983079 | 0.04886403 | 0.61101606 | -3.8355871 | -1.9697762 |
| PAQR8 | -0.2819224 | 4.95460055 | -1.9994174 | 0.04889295 | 0.61101606 | -3.8296965 | -1.969524 |
| LAP3 | 0.25030003 | 5.74309102 | 2.00002406 | 0.04890749 | 0.61101606 | -3.8285862 | 1.96939726 |
| BTBD17 | -0.6377658 | 4.18474448 | -2.0002305 | 0.04893408 | 0.61101606 | -3.8306692 | -1.9691656 |
| LIPT2 | 0.25018838 | 1.47047148 | 1.99956222 | 0.04898828 | 0.61101606 | -3.8234798 | 1.96869367 |
| MT1E | 0.85416319 | -0.0966956 | 1.99898993 | 0.04898907 | 0.61101606 | -3.8529057 | 1.96868674 |
| LINC02979 | 0.81480906 | -2.0712222 | 1.99826107 | 0.04900604 | 0.61101606 | -3.8248844 | 1.96853912 |
| JAZF1-AS1 | -1.0841076 | -2.4198381 | -1.998962 | 0.04901845 | 0.61101606 | -3.8193303 | -1.9684311 |
| INSR | -0.2382976 | 5.73107053 | -1.9984465 | 0.04903452 | 0.61101606 | -3.827721 | -1.9682914 |
| GNL2 | 0.14098676 | 5.54196489 | 1.99846001 | 0.04904783 | 0.61101606 | -3.8290821 | 1.9681757 |
| FPASL | 1.58805848 | -4.7818347 | 1.99631338 | 0.04907586 | 0.61101606 | -3.8312723 | 1.967932 |
| LINC01686 | 0.71613069 | -1.5601209 | 1.99591086 | 0.04910296 | 0.61101606 | -3.8606892 | 1.96769661 |
| CSTF1 | 0.1366213 | 4.75780666 | 1.99700201 | 0.04910872 | 0.61101606 | -3.832846 | 1.96764653 |
| RBMS1 | 0.65136478 | 5.14698842 | 1.99751581 | 0.04912133 | 0.61101606 | -3.8337998 | 1.96753704 |
| PPIAP41 | -1.4406225 | -4.6365828 | -1.9971974 | 0.04913267 | 0.61101606 | -3.8259012 | -1.9674386 |
| MYL6B-AS1 | 1.07169162 | -3.3023874 | 1.99519089 | 0.04919753 | 0.61101606 | -3.8263508 | 1.96687578 |
| CLEC18A | 0.60470868 | -0.020599 | 1.99796858 | 0.04920222 | 0.61101606 | -3.8337091 | 1.96683516 |
| MPC1L | 1.26355055 | -6.0844498 | 1.99466235 | 0.04926049 | 0.61101606 | -3.8442515 | 1.96633013 |
| DUSP6 | -0.3739576 | 3.96875245 | -1.9962011 | 0.04926586 | 0.61101606 | -3.8328177 | -1.9662836 |
| LONP2 | -0.1272912 | 6.69561771 | -1.9959339 | 0.04928237 | 0.61101606 | -3.8348604 | -1.9661406 |
| BTC | 0.80694815 | -0.1739562 | 1.99696529 | 0.04928387 | 0.61101606 | -3.8382844 | 1.96612762 |
| LRRCC1 | 0.19012575 | 4.92598959 | 1.99528255 | 0.04939499 | 0.6120541 | -3.8392235 | 1.9651663 |
| LRRC31 | -1.2564849 | -6.1237462 | -1.9917875 | 0.04944331 | 0.61231322 | -3.8421889 | -1.9647489 |
| PRR36 | -0.2580292 | 6.89328313 | -1.9944772 | 0.0495082 | 0.61254518 | -3.8354798 | -1.9641888 |
| LINC02253 | -0.8803616 | -1.9734121 | -1.9938476 | 0.04951688 | 0.61254518 | -3.8347347 | -1.9641139 |
| LINC01703 | 0.57233102 | -0.3289713 | 1.9968237 | 0.04957287 | 0.61257584 | -3.8254338 | 1.96363128 |
| FIGN | 0.43599585 | 5.47511785 | 1.99364313 | 0.04958158 | 0.61257584 | -3.8375232 | 1.9635562 |
| DERL3 | -0.5163 | -0.4540826 | -1.9927709 | 0.04961962 | 0.61257584 | -3.8411667 | -1.9632286 |
| SLC35F6 | 0.2512691 | 6.11597176 | 1.99263017 | 0.04965222 | 0.61257584 | -3.8403088 | 1.962948 |
| PAPPA-AS2 | -1.45239 | -5.0720408 | -1.98995 | 0.04967567 | 0.61257584 | -3.8374576 | -1.9627463 |
| PRRT1 | -0.227879 | 4.23059966 | -1.9919766 | 0.04974669 | 0.61257584 | -3.8369182 | -1.9621357 |
| LINC01756 | 1.06828165 | -6.5631668 | 1.99072625 | 0.04975043 | 0.61257584 | -3.8444655 | 1.96210351 |
| TPST2 | 0.47691722 | 4.62544843 | 1.99150953 | 0.04977146 | 0.61257584 | -3.8417315 | 1.96192287 |
| NFE2L2 | 0.21734781 | 6.17965252 | 1.99210625 | 0.04978085 | 0.61257584 | -3.8417326 | 1.96184225 |
| CAMK2A | -0.4859136 | 4.57256891 | -1.9916913 | 0.04981439 | 0.61257584 | -3.8336928 | -1.9615543 |
| FLJ38576 | 0.41923967 | -0.0564253 | 1.9904904 | 0.04987929 | 0.61257584 | -3.844174 | 1.96099768 |
| TTC8 | 0.20528406 | 5.13369518 | 1.99106031 | 0.04989413 | 0.61257584 | -3.8441195 | 1.96087053 |
| KCND1 | 0.34253784 | 3.12652479 | 1.99093761 | 0.04990033 | 0.61257584 | -3.8450521 | 1.96081737 |
| LATS1 | -0.2249083 | 6.21210636 | -1.9904797 | 0.04992761 | 0.61257584 | -3.8423864 | -1.9605837 |
| HSPA1L | 0.36601371 | 2.02065097 | 1.99095138 | 0.04993065 | 0.61257584 | -3.8416215 | 1.96055764 |
| STEAP1B | 0.82363526 | -1.9549161 | 1.98998875 | 0.04998929 | 0.61295868 | -3.8469738 | 1.96005562 |

**Supplementary Table S11:** Differentially Expressed Genes after DOI at 24 Hours

| **Gene Name** | **logFC** | **AveExpr** | **t** | **P.Value** | **adj.P.Val** | **B** | **z.std** |
| --- | --- | --- | --- | --- | --- | --- | --- |
| RNA5SP490 | -4.2901309 | -4.1457735 | -4.7576548 | 8.1001E-06 | 0.18096471 | -3.2490188 | -4.4625205 |
| FBRSL1P1 | 2.77837553 | -5.8965301 | 4.08372742 | 0.00010012 | 0.99976948 | -2.9920283 | 3.89029976 |
| RPH3AL-AS2 | 1.91654005 | -6.7613252 | 3.44626925 | 0.00088876 | 0.99976948 | -3.5110836 | 3.32355954 |
| FTLP14 | 2.61301385 | -6.179277 | 3.35081206 | 0.00135781 | 0.99976948 | -3.5325696 | 3.20347314 |
| HMGA2-AS1 | 2.70548935 | -6.0502992 | 3.31135755 | 0.00137668 | 0.99976948 | -3.6465238 | 3.19949582 |
| SPATA12 | 2.55092975 | -3.6129881 | 3.31697678 | 0.00137979 | 0.99976948 | -3.8172965 | 3.19884505 |
| H4C8 | -0.908908 | -0.3294703 | -3.273068 | 0.00156526 | 0.99976948 | -3.8850256 | -3.1623046 |
| CPEB1-AS1 | -1.5789181 | -1.5957702 | -3.2787496 | 0.00156618 | 0.99976948 | -3.8729107 | -3.1621333 |
| LINC02970 | 3.19493559 | -5.6240562 | 3.27402764 | 0.00159766 | 0.99976948 | -3.7818489 | 3.15633335 |
| PIP5K1P1 | 2.62870404 | -5.601248 | 3.22849116 | 0.00176213 | 0.99976948 | -3.5627228 | 3.12764488 |
| LINC00443 | -2.7018427 | -5.5940557 | -3.1774322 | 0.00209294 | 0.99976948 | -3.8103589 | -3.0767168 |
| IKZF3 | -2.8132485 | -4.8945555 | -3.1585869 | 0.002171 | 0.99976948 | -3.9104884 | -3.0657841 |
| PLA2G3 | 1.43766593 | -0.5697057 | 3.15075719 | 0.00226904 | 0.99976948 | -3.7178178 | 3.05255284 |
| KIAA1191P2 | -2.9073502 | -4.7719489 | -3.0964017 | 0.00262891 | 0.99976948 | -3.9298133 | -3.008095 |
| LINC01044 | 2.3604181 | -6.1365201 | 3.04866973 | 0.00306037 | 0.99976948 | -3.7834929 | 2.96160811 |
| ST7-OT4 | 1.97761343 | -6.1766474 | 3.0801273 | 0.00323218 | 0.99976948 | -3.6164568 | 2.94474826 |
| DDX10P2 | 2.0690864 | -6.3941828 | 3.02802976 | 0.00325766 | 0.99976948 | -3.7262384 | 2.94231742 |
| SEPTIN14 | -2.7174906 | -5.3986859 | -3.0246236 | 0.00330164 | 0.99976948 | -3.9392227 | -2.938162 |
| ASNSP6 | 2.24728511 | -6.1661964 | 3.00305163 | 0.00353101 | 0.99976948 | -3.72905 | 2.91727788 |
| RN7SL68P | -2.5767775 | -5.1034376 | -2.9961953 | 0.00357342 | 0.99976948 | -3.944198 | -2.9135524 |
| PPIAP7 | 2.04913123 | -5.8441271 | 2.89209186 | 0.00485508 | 0.99976948 | -3.8642627 | 2.81649531 |
| DYRK3-AS1 | 1.79112677 | -2.8497237 | 2.8992345 | 0.00487143 | 0.99976948 | -3.9399792 | 2.81541474 |
| RAX2 | -2.5612879 | -5.7161646 | -2.8885468 | 0.00493275 | 0.99976948 | -4.0027096 | -2.8113927 |
| ATP5MGP8 | 2.1513671 | -4.9818706 | 2.90555367 | 0.00494932 | 0.99976948 | -3.8667166 | 2.81031383 |
| RN7SL812P | -2.2740625 | -4.8888578 | -2.9120867 | 0.00498683 | 0.99976948 | -3.9817106 | -2.8078833 |
| LINC02424 | 1.88599608 | -5.7761459 | 2.86624104 | 0.00522827 | 0.99976948 | -3.8648309 | 2.79262252 |
| FNDC8 | 1.42739869 | -2.3409946 | 2.83155982 | 0.00576709 | 0.99976948 | -3.9419393 | 2.76073804 |
| SIM2 | -3.6959467 | -3.3268452 | -2.8084333 | 0.00622308 | 0.99976948 | -4.1693321 | -2.7357894 |
| POU4F3 | -1.0188804 | -1.401221 | -2.8100566 | 0.00630482 | 0.99976948 | -4.0344312 | -2.7314922 |
| MIR589 | -2.3918547 | -5.0681376 | -2.7949541 | 0.00644721 | 0.99976948 | -4.0207897 | -2.724125 |
| LINC00299 | 1.49652674 | -1.921729 | 2.78360922 | 0.00673172 | 0.99976948 | -3.9878761 | 2.70983265 |
| LINC01691 | 2.31257317 | -5.065633 | 2.79898751 | 0.00684593 | 0.99976948 | -3.9017738 | 2.70424725 |
| RN7SL239P | -2.3647032 | -5.1334117 | -2.7708348 | 0.00714717 | 0.99976948 | -4.0301464 | -2.6899079 |
| CBX5P1 | -2.7444317 | -5.6313383 | -2.7493561 | 0.00728234 | 0.99976948 | -4.054391 | -2.6836493 |
| FAM81A | -0.484508 | 3.28393785 | -2.7367415 | 0.00765852 | 0.99976948 | -4.0680211 | -2.666765 |
| SBF2-AS1 | 0.62436901 | 0.68888535 | 2.71708983 | 0.00805737 | 0.99976948 | -4.0433431 | 2.64965625 |
| MDFIC2 | -1.8762722 | -6.5197868 | -2.7002719 | 0.00845035 | 0.99976948 | -4.0655016 | -2.6335254 |
| PNN-AS1 | 1.63607865 | -3.3782381 | 2.69598735 | 0.00849817 | 0.99976948 | -4.0346383 | 2.63160857 |
| CA9 | 2.03793187 | -2.578882 | 2.6932286 | 0.00855739 | 0.99976948 | -3.9647215 | 2.62924799 |
| CD80 | 2.11701574 | -5.7666788 | 2.69017216 | 0.00858248 | 0.99976948 | -3.9566721 | 2.62825263 |
| FADS1 | 0.3326252 | 7.37270435 | 2.69306558 | 0.00859884 | 0.99976948 | -4.0420828 | 2.6276047 |
| PIK3CG | 1.95276357 | -6.4203922 | 2.67595776 | 0.00894989 | 0.99976948 | -3.920359 | 2.61396227 |
| LINC00161 | 1.89198262 | -6.3776146 | 2.65951118 | 0.00931632 | 0.99976948 | -3.971574 | 2.60022284 |
| RNU4-50P | -2.2158868 | -5.5725513 | -2.6574262 | 0.0093296 | 0.99976948 | -4.0826645 | -2.5997341 |
| RNU6-583P | -2.0939871 | -5.0909391 | -2.6536101 | 0.00954111 | 0.99976948 | -4.060044 | -2.5920314 |
| NUDT18 | 0.42409058 | 2.69176702 | 2.65451119 | 0.00957299 | 0.99976948 | -4.0375781 | 2.59088334 |
| LINC02300 | 2.44962603 | -4.4508409 | 2.65073068 | 0.00963552 | 0.99976948 | -3.9664822 | 2.58864207 |
| OR51B5 | -2.2927462 | -5.6561931 | -2.6477315 | 0.00970493 | 0.99976948 | -4.098632 | -2.586169 |
| LINC02047 | -1.8777278 | -6.0853801 | -2.6374687 | 0.00996466 | 0.99976948 | -4.0762938 | -2.5770531 |
| ZNF473CR | -0.5246212 | 0.37752955 | -2.6336153 | 0.01014162 | 0.99976948 | -4.1241221 | -2.5709628 |
| TCHH | -0.9493135 | 0.97138815 | -2.6255762 | 0.01035679 | 0.99976948 | -4.0982273 | -2.5636835 |
| FUT7 | -1.4640714 | -1.6322591 | -2.6229298 | 0.01040748 | 0.99976948 | -4.1133442 | -2.5619883 |
| GCG | 1.95754511 | -5.6187716 | 2.6202289 | 0.01043934 | 0.99976948 | -3.9760294 | 2.56092658 |
| WFDC5 | -2.3341813 | -5.747716 | -2.6148227 | 0.01065554 | 0.99976948 | -4.131321 | -2.553797 |
| THORLNC | 1.08214955 | -1.7578464 | 2.62167132 | 0.01067171 | 0.99976948 | -4.0193544 | 2.55326897 |
| RPS26P45 | 2.32821086 | -5.7885779 | 2.60704088 | 0.01070403 | 0.99976948 | -4.0113129 | 2.55221544 |
| EIF5A2P1 | -2.2664544 | -5.7317811 | -2.6059667 | 0.01075118 | 0.99976948 | -4.1165045 | -2.5506839 |
| EIF3LP3 | -1.8755029 | -3.0580834 | -2.6036493 | 0.01094857 | 0.99976948 | -4.1263066 | -2.5443361 |
| LINC00159 | -2.1960245 | -3.8989152 | -2.5969307 | 0.01111062 | 0.99976948 | -4.1579206 | -2.5392003 |
| PHB1P19 | 2.01715351 | -5.7792988 | 2.5909132 | 0.01124275 | 0.99976948 | -4.021358 | 2.53506187 |
| LDLR | 0.57752616 | 5.7820918 | 2.59391033 | 0.0112924 | 0.99976948 | -4.0849011 | 2.53351788 |
| HPAT5 | 3.95614534 | -0.9122371 | 2.58592352 | 0.01142683 | 0.99976948 | -4.0822386 | 2.52936739 |
| NMNAT1 | 0.21901391 | 3.57130762 | 2.58306742 | 0.01161627 | 0.99976948 | -4.0968626 | 2.52359187 |
| C16orf96 | 0.51126066 | 0.71973556 | 2.57262763 | 0.01203573 | 0.99976948 | -4.0863086 | 2.51109501 |
| GOT2P3 | 1.99156501 | -5.7191261 | 2.54324589 | 0.01278037 | 0.99976948 | -4.0022484 | 2.48983143 |
| MIR548I2 | -1.4583367 | -6.6024115 | -2.5381874 | 0.01302409 | 0.99976948 | -4.1041452 | -2.4831098 |
| PAPPA-AS2 | -2.558622 | -5.0720408 | -2.5234262 | 0.01338722 | 0.99976948 | -4.1781674 | -2.4732987 |
| HSD17B7 | 0.44497719 | 4.17762528 | 2.52650089 | 0.0134463 | 0.99976948 | -4.1083113 | 2.47172465 |
| SEPTIN7P13 | 1.78429496 | -5.442097 | 2.52264148 | 0.01356671 | 0.99976948 | -4.0580558 | 2.46853581 |
| RBM12B-DT | 2.12930566 | -5.0116718 | 2.52977964 | 0.01357115 | 0.99976948 | -4.0463905 | 2.46841874 |
| CPNE6 | -1.460909 | 1.60914502 | -2.519985 | 0.01368059 | 0.99976948 | -4.0954021 | -2.4655426 |
| DLG1-AS1 | -2.1471853 | -5.8467228 | -2.5154476 | 0.01386119 | 0.99976948 | -4.1444037 | -2.4608406 |
| LINC00700 | -1.4337095 | -6.6107491 | -2.5069134 | 0.01413184 | 0.99976948 | -4.1088776 | -2.4538945 |
| RPS27AP6 | -1.455116 | -2.8144322 | -2.5113291 | 0.01413503 | 0.99976948 | -4.154125 | -2.4538133 |
| FAM106A | -1.6878122 | -3.0596568 | -2.5058998 | 0.01418955 | 0.99976948 | -4.1361097 | -2.4524287 |
| TAX1BP1-AS1 | -2.5385775 | -5.2744016 | -2.5090587 | 0.0143144 | 0.99976948 | -4.1513821 | -2.4492752 |
| EEF1DP7 | -2.5483181 | -5.4356133 | -2.5005017 | 0.01433617 | 0.99976948 | -4.1591472 | -2.4487278 |
| BTF3L4P3 | 2.11089503 | -4.9735639 | 2.49202184 | 0.01454397 | 0.99976948 | -4.0358998 | 2.44353973 |
| SULT1A2 | 2.18350201 | -5.1952938 | 2.50875959 | 0.01456804 | 0.99976948 | -4.0358045 | 2.44294294 |
| LINC01119 | -0.9814701 | -1.0582775 | -2.4911782 | 0.01470988 | 0.99976948 | -4.2046326 | -2.4394439 |
| IL22RA1 | 2.11441688 | -5.7833755 | 2.51574695 | 0.01479972 | 0.99976948 | -4.0381077 | 2.43724328 |
| RIC3-DT | -1.4714803 | -2.9471512 | -2.4838003 | 0.0151504 | 0.99976948 | -4.1990193 | -2.4287637 |
| ZNF665 | 0.37622963 | 2.43551221 | 2.47565765 | 0.0153892 | 0.99976948 | -4.1394248 | 2.42308788 |
| RN7SL487P | 2.10890888 | -4.2229325 | 2.47234382 | 0.01547697 | 0.99976948 | -4.1018545 | 2.42102121 |
| RPS5P3 | 2.18858704 | -6.0643216 | 2.46844488 | 0.015571 | 0.99976948 | -4.0840556 | 2.41881874 |
| LINC01019 | 2.23657779 | -3.9767131 | 2.46872269 | 0.01560667 | 0.99976948 | -4.1032279 | 2.4179862 |
| CARD8-AS1 | 0.64092091 | -0.4202236 | 2.46690887 | 0.01568758 | 0.99976948 | -4.129704 | 2.41610396 |
| NPC1 | 0.33775877 | 5.5356465 | 2.46888016 | 0.01569605 | 0.99976948 | -4.1528607 | 2.41590755 |
| DHCR7-DT | 0.39905032 | 2.23362673 | 2.46606407 | 0.01576576 | 0.99976948 | -4.0927244 | 2.41429348 |
| HOXB-AS1 | -1.8402429 | -5.0080388 | -2.4644601 | 0.01582762 | 0.99976948 | -4.1811641 | -2.4128663 |
| CICP20 | 2.11639375 | -5.306105 | 2.46143974 | 0.01583314 | 0.99976948 | -4.0637127 | 2.4127393 |
| ENPP7P7 | -1.6717349 | -2.5268572 | -2.4630611 | 0.015862 | 0.99976948 | -4.1849522 | -2.4120754 |
| TOX4P1 | 2.02887336 | -5.3464058 | 2.45485173 | 0.01611397 | 0.99976948 | -4.074049 | 2.40632397 |
| LINC01907 | 1.67971555 | -3.6348504 | 2.458713 | 0.01613618 | 0.99976948 | -4.0792849 | 2.40582072 |
| RNU6-969P | -2.2624603 | -4.7263258 | -2.4503416 | 0.01634868 | 0.99976948 | -4.1531745 | -2.4010371 |
| DHCR24 | 0.59813311 | 7.66762182 | 2.44846356 | 0.01652332 | 0.99976948 | -4.1488038 | 2.39714648 |
| TAS2R1 | -2.0162021 | -4.1560248 | -2.4449124 | 0.01663302 | 0.99976948 | -4.1611559 | -2.3947209 |
| MIR5693 | 2.00875032 | -5.6444954 | 2.44218609 | 0.01665034 | 0.99976948 | -4.1200678 | 2.3943392 |
| MIR579 | 1.777662 | -5.9303363 | 2.46056421 | 0.01694515 | 0.99976948 | -4.1103959 | 2.38789579 |
| OR3A2 | -1.8095278 | -6.3528106 | -2.4332294 | 0.01713833 | 0.99976948 | -4.1343776 | -2.3837265 |
| VPS35P1 | -2.4913783 | -5.127402 | -2.4289664 | 0.01715659 | 0.99976948 | -4.1724909 | -2.3833347 |
| HOXC10 | -2.2452953 | -5.0348185 | -2.4144876 | 0.01795564 | 0.99976948 | -4.200232 | -2.3665317 |
| TFRC | 0.36805325 | 6.87424247 | 2.41546439 | 0.01796381 | 0.99976948 | -4.1558827 | 2.36636318 |
| GRB7 | 1.69470513 | -2.7845737 | 2.40835134 | 0.01825761 | 0.99976948 | -4.1858623 | 2.36035181 |
| RN7SL130P | 2.03291099 | -4.3100171 | 2.40519519 | 0.01842968 | 0.99976948 | -4.1207669 | 2.35687043 |
| LIF-AS2 | 2.39674524 | -3.8621017 | 2.40402802 | 0.01848435 | 0.99976948 | -4.1765546 | 2.35577025 |
| RPL21P134 | 2.20723914 | -4.8640587 | 2.40100118 | 0.01851686 | 0.99976948 | -4.1081423 | 2.35511738 |
| GRIK2 | -0.4078785 | 5.7984472 | -2.4013142 | 0.0186557 | 0.99976948 | -4.183268 | -2.3523403 |
| CRTC3-AS1 | 0.65524248 | -1.0197983 | 2.4044796 | 0.01872818 | 0.99976948 | -4.1166877 | 2.35089768 |
| LINC02396 | -2.5041156 | -4.8098583 | -2.3918167 | 0.01888224 | 0.99976948 | -4.2029138 | -2.3478477 |
| JAGN1 | 0.32523596 | 4.68331739 | 2.39041011 | 0.01919917 | 0.99976948 | -4.1822875 | 2.34164099 |
| FA2H | 1.37459311 | -1.6176331 | 2.39031058 | 0.01922937 | 0.99976948 | -4.2010964 | 2.34105433 |
| STH | -1.5204127 | -3.256909 | -2.3868972 | 0.01934417 | 0.99976948 | -4.1793534 | -2.3388312 |
| LINC00519 | 1.83202268 | -4.7947363 | 2.38696403 | 0.01934433 | 0.99976948 | -4.1356198 | 2.33882821 |
| RPL21P25 | 1.64625466 | -6.2920466 | 2.38241613 | 0.01943807 | 0.99976948 | -4.117193 | 2.33702137 |
| FGF16 | -2.2580423 | -5.7959326 | -2.3822544 | 0.01951531 | 0.99976948 | -4.2108863 | -2.3355385 |
| VSTM1 | 2.31359648 | -1.5775228 | 2.38209574 | 0.01962731 | 0.99976948 | -4.2677893 | 2.33339709 |
| RAB42 | 1.06137302 | 1.2277891 | 2.38120707 | 0.01972442 | 0.99976948 | -4.2191616 | 2.33154927 |
| KCTD21-AS1 | 0.57574143 | 1.06258159 | 2.37718981 | 0.0198105 | 0.99976948 | -4.1762478 | 2.32991776 |
| BICRA-AS1 | -2.0128756 | -6.2089307 | -2.3718423 | 0.02009492 | 0.99976948 | -4.1860785 | -2.3245709 |
| HSPE1P8 | 2.05984915 | -5.4493155 | 2.3707532 | 0.02013384 | 0.99976948 | -4.1197594 | 2.32384422 |
| OSGEPL1-AS1 | -0.5763813 | -0.7027666 | -2.3601669 | 0.02072247 | 0.99976948 | -4.2109708 | -2.313003 |
| DPH6 | 0.27230222 | 3.0384695 | 2.35703521 | 0.02088594 | 0.99976948 | -4.2001177 | 2.31004005 |
| RPL21P54 | -1.0777641 | -1.9801453 | -2.3551388 | 0.02088969 | 0.99976948 | -4.2328592 | -2.3099722 |
| OR2AJ1 | 1.87008747 | -5.4769911 | 2.3517587 | 0.02096498 | 0.99976948 | -4.1275189 | 2.3086145 |
| HRCT1 | 2.19007029 | -1.8837306 | 2.35650743 | 0.02101595 | 0.99976948 | -4.3018251 | 2.30769789 |
| LSS | 0.33885598 | 7.42552938 | 2.35158392 | 0.02113802 | 0.99976948 | -4.1876876 | 2.3055102 |
| DOCK7-DT | 0.51688226 | 1.88131077 | 2.35042469 | 0.02116612 | 0.99976948 | -4.1839975 | 2.30500818 |
| KCNE3 | 1.65315125 | -5.7617228 | 2.34944127 | 0.0211731 | 0.99976948 | -4.1586638 | 2.30488344 |
| KCNQ5-AS1 | 1.92463853 | -5.1093033 | 2.34380406 | 0.02144215 | 0.99976948 | -4.1275637 | 2.3001072 |
| C10orf71 | 1.8438632 | -5.3407816 | 2.34238241 | 0.02154857 | 0.99976948 | -4.1842243 | 2.2982324 |
| MIR4736 | 1.59693112 | -5.3530983 | 2.33908792 | 0.02172364 | 0.99976948 | -4.1078192 | 2.2951656 |
| TRIM7-AS2 | -1.0587787 | -1.6298862 | -2.3408654 | 0.02191937 | 0.99976948 | -4.2080303 | -2.2917622 |
| PAUPAR | 0.74509617 | -0.8023929 | 2.33678879 | 0.02199692 | 0.99976948 | -4.1597834 | 2.29042114 |
| ELOVL5 | 0.25854184 | 7.1838722 | 2.32407811 | 0.02266331 | 0.99976948 | -4.2056475 | 2.27906317 |
| NSDHL | 0.43864571 | 5.16509528 | 2.31789356 | 0.02302914 | 0.99976948 | -4.203229 | 2.27295081 |
| SLC25A15P3 | -1.7318385 | -5.9435666 | -2.3147027 | 0.02311905 | 0.99976948 | -4.1854898 | -2.2714616 |
| MCAM | 0.60496014 | 5.54066708 | 2.31626398 | 0.02314575 | 0.99976948 | -4.2168514 | 2.27102031 |
| LINC01744 | -1.6840521 | -6.4155036 | -2.3090925 | 0.02345065 | 0.99976948 | -4.2298087 | -2.2660119 |
| CFAP95-DT | 2.45905405 | -4.9881848 | 2.30498073 | 0.02365707 | 0.99976948 | -4.1795078 | 2.26265333 |
| LINC01111 | 1.94031628 | -5.3251475 | 2.30358197 | 0.02369948 | 0.99976948 | -4.1617822 | 2.26196636 |
| MMAB | 0.27518756 | 5.21462618 | 2.30346161 | 0.02382871 | 0.99976948 | -4.2001887 | 2.25987979 |
| VDAC2P5 | -0.6124188 | -0.982039 | -2.2998117 | 0.02391153 | 0.99976948 | -4.191829 | -2.2585477 |
| PKP1 | -2.441613 | -4.4731676 | -2.300803 | 0.02393592 | 0.99976948 | -4.2707504 | -2.2581563 |
| MTATP6P17 | -1.8728203 | -6.3095439 | -2.2891732 | 0.02443909 | 0.99976948 | -4.1987886 | -2.2501552 |
| IQSEC2 | -0.2878482 | 5.91843767 | -2.2923072 | 0.02454392 | 0.99976948 | -4.2112279 | -2.2485063 |
| SETP5 | 1.60464502 | -6.2440992 | 2.28757021 | 0.0246865 | 0.99976948 | -4.1672381 | 2.24627336 |
| LINC01096 | 1.66542756 | -6.5593366 | 2.29081868 | 0.02470824 | 0.99976948 | -4.1638336 | 2.24593401 |
| NFS1 | -0.1941549 | 4.13367794 | -2.2878176 | 0.02484708 | 0.99976948 | -4.2183066 | -2.2437719 |
| CCDC148 | -0.5569774 | 1.54186071 | -2.2825255 | 0.02503711 | 0.99976948 | -4.2209375 | -2.2408297 |
| SMYD3-IT1 | -2.0024494 | -6.0405253 | -2.273299 | 0.02543 | 0.99976948 | -4.2352307 | -2.2348073 |
| ARSI | 1.80118366 | -2.3942997 | 2.276774 | 0.02543543 | 0.99976948 | -4.2629155 | 2.23472473 |
| LINC02313 | -1.9135488 | -5.83265 | -2.2716795 | 0.02554121 | 0.99976948 | -4.2318382 | -2.2331174 |
| AIRN | -1.9549622 | -6.3091727 | -2.2750694 | 0.02555705 | 0.99976948 | -4.2211479 | -2.2328771 |
| LINC02895 | -1.8527852 | -6.2884228 | -2.2843824 | 0.02555789 | 0.99976948 | -4.1819511 | -2.2328643 |
| SCAT1 | -1.8570108 | -6.1248396 | -2.2731862 | 0.02563287 | 0.99976948 | -4.229477 | -2.2317291 |
| ZNF252P-AS1 | 0.61326603 | -0.2960115 | 2.27225893 | 0.02568692 | 0.99976948 | -4.2142834 | 2.23091252 |
| SMC2-DT | 1.03501093 | -1.5981911 | 2.27122881 | 0.02579675 | 0.99976948 | -4.2209308 | 2.22925789 |
| GREM1 | 2.8491568 | -0.0656707 | 2.26625617 | 0.02603667 | 0.99976948 | -4.2447478 | 2.2256644 |
| KRT89P | 1.77198085 | -5.8713893 | 2.26491394 | 0.02608178 | 0.99976948 | -4.2047338 | 2.22499197 |
| LINC01608 | 1.64125446 | -6.4833185 | 2.2642784 | 0.02612997 | 0.99976948 | -4.1827265 | 2.2242747 |
| ADAMTS7P1 | -0.8958069 | -0.1181021 | -2.261363 | 0.02642994 | 0.99976948 | -4.2681155 | -2.2198355 |
| MIR4733HG | -0.8424383 | -1.2838167 | -2.2591819 | 0.02644425 | 0.99976948 | -4.228175 | -2.2196248 |
| FUT4 | 0.47389083 | 1.45635412 | 2.26062153 | 0.02651941 | 0.99976948 | -4.2605233 | 2.21851979 |
| PFKFB4 | 0.23723401 | 4.83222714 | 2.25724199 | 0.02673557 | 0.99976948 | -4.213287 | 2.21535705 |
| INSIG1 | 0.57178453 | 6.34253245 | 2.255386 | 0.02676144 | 0.99976948 | -4.2185255 | 2.21497991 |
| CDH19 | 1.87552317 | -4.5360541 | 2.25673808 | 0.0267626 | 0.99976948 | -4.2169126 | 2.21496302 |
| RNU6-593P | 1.78386616 | -5.6588152 | 2.24995928 | 0.02699918 | 0.99976948 | -4.2118067 | 2.21152967 |
| HSPD1P12 | -2.3072015 | -4.9312175 | -2.2501814 | 0.02707835 | 0.99976948 | -4.2641385 | -2.2103864 |
| ACVRL1 | 1.63306527 | -2.7219264 | 2.24785148 | 0.02729943 | 0.99976948 | -4.2196019 | 2.20720942 |
| UTS2B | -0.7017404 | -0.5657689 | -2.2463902 | 0.02734577 | 0.99976948 | -4.2743702 | -2.2065464 |
| AGRP | -2.1739714 | -3.6524241 | -2.2444951 | 0.02746218 | 0.99976948 | -4.2969599 | -2.2048849 |
| TRPC4 | -0.6184376 | 3.45483889 | -2.2452874 | 0.02750672 | 0.99976948 | -4.2854223 | -2.2042507 |
| H3C2 | -2.1863828 | -5.7328649 | -2.2437606 | 0.02751655 | 0.99976948 | -4.2345164 | -2.2041108 |
| RAB32 | 0.89197977 | 2.49264386 | 2.24530532 | 0.02751939 | 0.99976948 | -4.2830552 | 2.20407048 |
| COSMOC | 1.68102505 | -3.5197372 | 2.23997597 | 0.02779218 | 0.99976948 | -4.145949 | 2.20020745 |
| CLCA2 | 1.7835422 | -2.8286589 | 2.23513604 | 0.02811769 | 0.99976948 | -4.263466 | 2.19564049 |
| MIR4754 | 1.35116747 | -3.2499722 | 2.23101261 | 0.02818719 | 0.99976948 | -4.2338165 | 2.19467117 |
| PHF10P1 | -1.8561915 | -5.1148365 | -2.231251 | 0.02825217 | 0.99976948 | -4.2399295 | -2.1937669 |
| CLDN7 | 1.72627622 | -5.2769434 | 2.22913587 | 0.02845106 | 0.99976948 | -4.2027597 | 2.19101014 |
| BARX1 | -2.5221059 | -4.1066447 | -2.2279197 | 0.02855199 | 0.99976948 | -4.275517 | -2.1896175 |
| GPIHBP1 | 2.25679026 | -3.4179573 | 2.23055416 | 0.02856616 | 0.99976948 | -4.2733124 | 2.18942232 |
| RNU5A-3P | -1.8385501 | -6.0604729 | -2.229051 | 0.028724 | 0.99976948 | -4.2461985 | -2.1872538 |
| MIR3180-5 | -1.3365376 | -4.2975482 | -2.2245436 | 0.02888087 | 0.99976948 | -4.2308167 | -2.1851087 |
| TAF13 | 0.23321625 | 5.3289443 | 2.22386366 | 0.02896648 | 0.99976948 | -4.2477209 | 2.18394248 |
| CD207 | -1.8563864 | -6.0008358 | -2.2208462 | 0.02906511 | 0.99976948 | -4.2355312 | -2.1826023 |
| HNRNPA1P62 | -2.2953008 | -5.4611706 | -2.2196173 | 0.02913073 | 0.99976948 | -4.2846662 | -2.1817129 |
| MYL6B-AS1 | 1.43942169 | -3.3023874 | 2.21893364 | 0.02913979 | 0.99976948 | -4.2310516 | 2.18159027 |
| ALG14-AS1 | 1.90687116 | -5.4611771 | 2.21701576 | 0.02920297 | 0.99976948 | -4.2060364 | 2.18073575 |
| LINC02475 | -2.0775873 | -5.834207 | -2.2185713 | 0.02923925 | 0.99976948 | -4.2461928 | -2.1802458 |
| STARD4 | 0.41754269 | 5.25711702 | 2.21856844 | 0.02935846 | 0.99976948 | -4.2071698 | 2.17863945 |
| MIR7856 | -1.8036024 | -4.3747002 | -2.2172025 | 0.02936648 | 0.99976948 | -4.2626911 | -2.1785316 |
| KCNIP2-AS1 | -0.8934057 | -1.1646207 | -2.216262 | 0.02941436 | 0.99976948 | -4.2727217 | -2.1778882 |
| RPL7P23 | 1.09832798 | -1.6214434 | 2.21470218 | 0.02958303 | 0.99976948 | -4.1788676 | 2.17562883 |
| GOLGA2P11 | 1.3803131 | -6.096824 | 2.21276413 | 0.0296166 | 0.99976948 | -4.2228134 | 2.17518047 |
| RNASEH2A | 1.04347586 | -2.629769 | 2.20526288 | 0.03028195 | 0.99976948 | -4.2026886 | 2.16638282 |
| BANCR | 2.28131591 | -5.4743063 | 2.20311804 | 0.03029614 | 0.99976948 | -4.27413 | 2.1661969 |
| VCAN-AS1 | 1.18214438 | -2.287434 | 2.20542126 | 0.03033574 | 0.99976948 | -4.2222992 | 2.16567883 |
| DGKG | 0.56154607 | 1.80907077 | 2.20268805 | 0.03046186 | 0.99976948 | -4.2059015 | 2.16403242 |
| CRACR2A | 1.16696446 | -0.1761363 | 2.19932277 | 0.03061233 | 0.99976948 | -4.2530628 | 2.16207587 |
| ASB4 | 1.54351494 | -2.5248452 | 2.19792674 | 0.03063744 | 0.99976948 | -4.182521 | 2.16175014 |
| NHERF4 | -1.7974183 | -3.296247 | -2.2016454 | 0.03072472 | 0.99976948 | -4.2970561 | -2.1606198 |
| RNU6-726P | -1.8417001 | -5.774254 | -2.1980648 | 0.03074681 | 0.99976948 | -4.2826295 | -2.1603342 |
| WDFY4 | -1.728967 | -3.1813775 | -2.1939029 | 0.03084972 | 0.99976948 | -4.2892016 | -2.1590057 |
| ACADL | -0.4770373 | 0.92530062 | -2.1961678 | 0.03095816 | 0.99976948 | -4.2880531 | -2.15761 |
| DRD5 | -1.5870526 | -3.3395865 | -2.1960222 | 0.03099501 | 0.99976948 | -4.2618803 | -2.1571368 |
| PDSS1P1 | 2.08989196 | -4.8509802 | 2.20349718 | 0.03111848 | 0.99976948 | -4.2085104 | 2.15555439 |
| OR52I1 | -1.5599085 | -6.6416528 | -2.187755 | 0.03129352 | 0.99976948 | -4.229995 | -2.1533202 |
| LINC01823 | 1.61527944 | -4.2520025 | 2.18771098 | 0.03141672 | 0.99976948 | -4.2227595 | 2.15175417 |
| ANKRD34A | -0.3900214 | 2.05354855 | -2.1882082 | 0.0315379 | 0.99976948 | -4.2541769 | -2.150219 |
| HCCS | 0.21443266 | 3.75398307 | 2.18646165 | 0.03165555 | 0.99976948 | -4.2667625 | 2.1487332 |
| LINC02955 | 1.97884748 | -5.2340672 | 2.19175684 | 0.03178272 | 0.99976948 | -4.204457 | 2.14713266 |
| PRTN3 | 1.85191779 | -5.6684061 | 2.19796047 | 0.0318056 | 0.99976948 | -4.2027392 | 2.14684533 |
| LDB3 | 0.52762965 | 1.18263431 | 2.18281344 | 0.03192172 | 0.99976948 | -4.2324575 | 2.14538942 |
| LINC02936 | 1.21288187 | -1.9912974 | 2.18713106 | 0.03204405 | 0.99976948 | -4.2476859 | 2.14386075 |
| HOXC6 | -1.8413516 | -5.9700659 | -2.1796115 | 0.03211218 | 0.99976948 | -4.2400266 | -2.1430114 |
| DOCK6-AS1 | -1.482444 | -2.952778 | -2.1775142 | 0.0321873 | 0.99976948 | -4.2521006 | -2.1420768 |
| PZP | 1.47689077 | -1.5978276 | 2.17832027 | 0.03232558 | 0.99976948 | -4.3144716 | 2.14036132 |
| VIL1 | 1.73688982 | -4.8333898 | 2.17494996 | 0.03237952 | 0.99976948 | -4.22239 | 2.13969379 |
| ASXL3-DT | -1.7398827 | -5.1654218 | -2.1766025 | 0.03239805 | 0.99976948 | -4.2714755 | -2.1394648 |
| BTF3P3 | -2.0101876 | -5.8740699 | -2.1731904 | 0.03240749 | 0.99976948 | -4.2639886 | -2.139348 |
| FNIP1 | 0.25353058 | 5.59004672 | 2.18205238 | 0.03247707 | 0.99976948 | -4.2570496 | 2.13848902 |
| ACOT8 | -0.1778052 | 4.41964915 | -2.1756164 | 0.03261742 | 0.99976948 | -4.2611776 | -2.1367611 |
| GCOM1 | -2.0911445 | -3.9255097 | -2.1747799 | 0.03275494 | 0.99976948 | -4.3178797 | -2.1350743 |
| PRRT1 | -0.3113604 | 4.23059966 | -2.1714512 | 0.03288606 | 0.99976948 | -4.2540855 | -2.1334716 |
| NT5DC4 | -1.8767945 | -4.6871281 | -2.1654343 | 0.03320858 | 0.99976948 | -4.2730966 | -2.1295525 |
| ENTREP2 | -0.4645307 | 4.417554 | -2.1662135 | 0.03328244 | 0.99976948 | -4.2531507 | -2.1286596 |
| LINC02560 | 0.83277564 | -1.700573 | 2.17056678 | 0.03329322 | 0.99976948 | -4.2107104 | 2.1285293 |
| PRSS57 | 1.78431935 | -6.4355236 | 2.17613694 | 0.03342326 | 0.99976948 | -4.1941948 | 2.12696173 |
| RN7SL231P | 1.78537912 | -5.6368004 | 2.15841185 | 0.03372417 | 0.99976948 | -4.2060068 | 2.12335439 |
| FKBP5 | 0.70363242 | 2.45014721 | 2.15972706 | 0.03376441 | 0.99976948 | -4.2811338 | 2.12287404 |
| LINC01629 | 1.91336249 | -5.6142982 | 2.15739554 | 0.03380397 | 0.99976948 | -4.2577887 | 2.12240232 |
| LINC00994 | -1.8332229 | -5.4696037 | -2.1584356 | 0.03404147 | 0.99976948 | -4.2726157 | -2.1195801 |
| ZHX1-C8orf76 | -1.4327932 | -3.5569181 | -2.15526 | 0.03411295 | 0.99976948 | -4.2781289 | -2.118734 |
| TRHR | -1.0923243 | -1.6675773 | -2.154527 | 0.03423374 | 0.99976948 | -4.2493578 | -2.1173078 |
| SLCO4C1 | 0.94243857 | -0.6401478 | 2.15040022 | 0.03443225 | 0.99976948 | -4.2354159 | 2.11497297 |
| TMEM97 | 0.43395104 | 5.42636329 | 2.15069967 | 0.03451475 | 0.99976948 | -4.2605651 | 2.11400606 |
| NOP16 | 0.20322299 | 3.32324478 | 2.15011999 | 0.03460468 | 0.99976948 | -4.2642781 | 2.11295429 |
| C15orf48 | 1.60530119 | -3.6896232 | 2.14338506 | 0.03480089 | 0.99976948 | -4.2734338 | 2.11066758 |
| HSD17B2-AS1 | -1.7405922 | -3.9986312 | -2.1431452 | 0.03481823 | 0.99976948 | -4.2936585 | -2.110466 |
| GAU1 | -0.6551336 | -0.1062825 | -2.1457596 | 0.03482145 | 0.99976948 | -4.3103588 | -2.1104287 |
| EVX1 | -1.5430769 | -6.522147 | -2.145894 | 0.03490661 | 0.99976948 | -4.265868 | -2.1094401 |
| PIGH | 0.26593759 | 4.25895943 | 2.145922 | 0.03501708 | 0.99976948 | -4.282431 | 2.10816079 |
| TIGD6 | 0.25773765 | 2.5594283 | 2.1420522 | 0.03523139 | 0.99976948 | -4.269213 | 2.10568885 |
| FAAP24 | 0.32921837 | 2.13017747 | 2.14058881 | 0.03526842 | 0.99976948 | -4.2646769 | 2.10526296 |
| PIEZO2 | 1.32639077 | 4.86756445 | 2.14033511 | 0.0353504 | 0.99976948 | -4.2877399 | 2.10432162 |
| MSMO1 | 0.39725352 | 7.60225065 | 2.13974587 | 0.0354125 | 0.99976948 | -4.2729501 | 2.10360977 |
| RPF2 | 0.20855571 | 4.00314694 | 2.13750092 | 0.0355027 | 0.99976948 | -4.2821894 | 2.10257769 |
| RINL | 0.52571208 | 0.58609183 | 2.13910516 | 0.03550291 | 0.99976948 | -4.2690016 | 2.1025753 |
| FMNL1 | -0.3363129 | 3.56506099 | -2.1388404 | 0.03552558 | 0.99976948 | -4.2598314 | -2.1023163 |
| MRPL14 | 0.31908244 | 3.94089935 | 2.13780322 | 0.03556172 | 0.99976948 | -4.2708681 | 2.10190366 |
| PTTG2 | 1.61939968 | -6.2900271 | 2.1340958 | 0.03569066 | 0.99976948 | -4.2064835 | 2.1004342 |
| DNTT | -1.8239716 | -6.1815438 | -2.1334812 | 0.03576501 | 0.99976948 | -4.2956466 | -2.0995891 |
| CYCSP8 | -1.6189124 | -3.6424004 | -2.1342156 | 0.03586355 | 0.99976948 | -4.2907044 | -2.0984711 |
| UNC119 | -0.2137501 | 5.82241961 | -2.1343555 | 0.03590321 | 0.99976948 | -4.27273 | -2.0980219 |
| TMEM230P2 | -2.1328951 | -5.0282392 | -2.1277301 | 0.03610952 | 0.99976948 | -4.2944714 | -2.095692 |
| CDK6-AS1 | -1.4982207 | -1.8236555 | -2.13032 | 0.03611597 | 0.99976948 | -4.3337082 | -2.0956194 |
| VEZF1P1 | -2.0415282 | -5.0112965 | -2.1308776 | 0.03611974 | 0.99976948 | -4.311741 | -2.0955769 |
| LARP4 | 0.15725932 | 5.79813269 | 2.13111882 | 0.03618108 | 0.99976948 | -4.2821391 | 2.09488659 |
| KCTD11 | 0.32705294 | 3.98061546 | 2.1300466 | 0.03624748 | 0.99976948 | -4.2968009 | 2.09414033 |
| OR7C1 | -1.747 | -4.7358704 | -2.1288133 | 0.03641442 | 0.99976948 | -4.2709605 | -2.0922694 |
| COA6 | 0.28863473 | 2.52112615 | 2.12110983 | 0.03670255 | 0.99976948 | -4.2731511 | 2.0890575 |
| BDKRB2 | 1.60268 | -1.0479431 | 2.12407638 | 0.03670331 | 0.99976948 | -4.293789 | 2.08904903 |
| LINC02074 | -1.7382381 | -5.5807051 | -2.123545 | 0.03670361 | 0.99976948 | -4.273897 | -2.0890457 |
| FDX2 | -1.7316696 | -4.5701329 | -2.1241836 | 0.0367234 | 0.99976948 | -4.3189172 | -2.0888259 |
| UBE2D3P1 | 1.07431987 | -2.4874216 | 2.12151314 | 0.03678089 | 0.99976948 | -4.223553 | 2.08818792 |
| TTLL10-AS1 | -1.6191858 | -5.0854725 | -2.1207816 | 0.03700237 | 0.99976948 | -4.3472409 | -2.0857379 |
| H2BW1 | 1.63025978 | -5.4863369 | 2.12036618 | 0.03702043 | 0.99976948 | -4.2049692 | 2.08553866 |
| FABP12 | 1.57200494 | -6.0544453 | 2.11869554 | 0.03702062 | 0.99976948 | -4.2203983 | 2.08553663 |
| TNFRSF17 | -1.5934276 | -4.4485657 | -2.1183314 | 0.03716583 | 0.99976948 | -4.2926858 | -2.0839378 |
| LINC01993 | 2.01646577 | -0.1271592 | 2.11865318 | 0.03724127 | 0.99976948 | -4.3466194 | 2.08310922 |
| ISCA1P6 | -1.8295209 | -5.2562622 | -2.1169516 | 0.03730185 | 0.99976948 | -4.2858449 | -2.0824449 |
| ZBTB42 | 0.74075776 | 2.39065935 | 2.11782865 | 0.03731296 | 0.99976948 | -4.3146605 | 2.08232321 |
| C16orf95 | -0.5207685 | -0.0947393 | -2.1136654 | 0.03757246 | 0.99976948 | -4.2824139 | -2.0794887 |
| CFAP58-DT | 0.86930371 | -2.0247862 | 2.11540603 | 0.0376449 | 0.99976948 | -4.2481144 | 2.0787004 |
| LINC02078 | 1.50318679 | -5.3413766 | 2.11301289 | 0.03765712 | 0.99976948 | -4.2374729 | 2.07856752 |
| PPIAP32 | 1.63194874 | -4.5857683 | 2.11109939 | 0.03769866 | 0.99976948 | -4.2527374 | 2.07811614 |
| C11orf86 | 2.30862861 | -5.4997057 | 2.1112721 | 0.03781204 | 0.99976948 | -4.2865311 | 2.07688642 |
| HMGB1P13 | 1.75627555 | -5.673235 | 2.10785693 | 0.03798697 | 0.99976948 | -4.2519849 | 2.07499534 |
| LINC02696 | -0.7001316 | 0.23567363 | -2.1100817 | 0.03806964 | 0.99976948 | -4.2857281 | -2.0741041 |
| PHB1P3 | 1.71371222 | -6.3183095 | 2.10776956 | 0.03808471 | 0.99976948 | -4.2431947 | 2.07394184 |
| PCSK6 | 0.99136473 | 3.50746129 | 2.10687103 | 0.03830065 | 0.99976948 | -4.2854453 | 2.07162257 |
| BTBD6P1 | -1.9296872 | -5.7511596 | -2.1043731 | 0.0383554 | 0.99976948 | -4.3160907 | -2.0710364 |
| MAPK13 | -0.6426154 | 2.39649855 | -2.103173 | 0.03864545 | 0.99976948 | -4.2977457 | -2.0679423 |
| RNU6-377P | 1.40381665 | -4.7891159 | 2.10070457 | 0.03873591 | 0.99976948 | -4.2500501 | 2.0669814 |
| IFT74-AS1 | -2.2019046 | -4.7318915 | -2.0993896 | 0.03881738 | 0.99976948 | -4.3299685 | -2.0661175 |
| KCNH3 | -0.503969 | 3.31472344 | -2.0998619 | 0.03891558 | 0.99976948 | -4.2869974 | -2.0650785 |
| DGKZP1 | -0.5007772 | -0.5894459 | -2.0984772 | 0.03892657 | 0.99976948 | -4.2886905 | -2.0649623 |
| C3orf36 | 1.78901956 | -5.7880532 | 2.10584352 | 0.03899928 | 0.99976948 | -4.2395677 | 2.06419452 |
| DHCR7 | 0.4273031 | 6.65402176 | 2.09801647 | 0.03904583 | 0.99976948 | -4.2864008 | 2.0637036 |
| TCEAL8 | 0.23124451 | 5.60555081 | 2.09874419 | 0.03910382 | 0.99976948 | -4.2924163 | 2.06309273 |
| PPP2R3C | 0.20428069 | 3.84593255 | 2.09660521 | 0.03911962 | 0.99976948 | -4.2917674 | 2.06292643 |
| GLP2R | 2.00220572 | -4.5242476 | 2.09481581 | 0.03924934 | 0.99976948 | -4.2755148 | 2.06156319 |
| GUCY2F | -2.1548248 | -4.3585886 | -2.0967602 | 0.03927765 | 0.99976948 | -4.3356684 | -2.0612662 |
| FAM246B | 1.24012327 | -5.8252908 | 2.09285572 | 0.03950048 | 0.99976948 | -4.2479098 | 2.05893485 |
| MMEL1-AS1 | -1.3920598 | -2.9016699 | -2.0915452 | 0.03966712 | 0.99976948 | -4.3395613 | -2.0571987 |
| PRADC1P1 | 1.61594547 | -3.5566296 | 2.09450258 | 0.03974222 | 0.99976948 | -4.2635612 | 2.05641825 |
| LINC02256 | 0.54510978 | -1.1419117 | 2.08898737 | 0.03977987 | 0.99976948 | -4.2908176 | 2.05602748 |
| RNVU1-29 | -1.663573 | -5.1136288 | -2.0892574 | 0.039784 | 0.99976948 | -4.3013339 | -2.0559846 |
| LINC02709 | 1.46566175 | -3.2282574 | 2.08442444 | 0.04011994 | 0.99976948 | -4.2660026 | 2.05251191 |
| LINC02290 | -1.6731473 | -4.1458987 | -2.0835952 | 0.04013871 | 0.99976948 | -4.325313 | -2.0523186 |
| RPL15P11 | -1.7520648 | -4.3595814 | -2.0854849 | 0.04019189 | 0.99976948 | -4.3082008 | -2.0517713 |
| HIBADH | 0.33701616 | 4.01675077 | 2.08558676 | 0.04022318 | 0.99976948 | -4.3098611 | 2.05144968 |
| LINC02938 | -1.801996 | -4.7255447 | -2.0848152 | 0.04026651 | 0.99976948 | -4.3184571 | -2.0510045 |
| LINC02552 | 1.92574405 | -4.333415 | 2.08736226 | 0.04027128 | 0.99976948 | -4.2790286 | 2.05095553 |
| RAP2CP1 | 1.70255539 | -5.9737522 | 2.08123058 | 0.0402968 | 0.99976948 | -4.242211 | 2.05069351 |
| TRBV5-4 | 1.73017503 | -6.1625796 | 2.08051275 | 0.04049197 | 0.99976948 | -4.2688223 | 2.04869476 |
| MIR153-1 | 1.6361989 | -4.9282469 | 2.08142183 | 0.04049315 | 0.99976948 | -4.2594139 | 2.04868268 |
| LINC02207 | 1.46845667 | -3.3221656 | 2.08042439 | 0.04062545 | 0.99976948 | -4.2876561 | 2.04733246 |
| GPR25 | -2.0997931 | -6.040199 | -2.0779295 | 0.04077735 | 0.99976948 | -4.3166369 | -2.0457868 |
| SHMT1 | 0.42237328 | 4.1646651 | 2.07819703 | 0.04091619 | 0.99976948 | -4.3025011 | 2.04437827 |
| ADAM3B | -1.6752915 | -6.1097664 | -2.0760103 | 0.04093033 | 0.99976948 | -4.2802119 | -2.0442351 |
| KLF6 | 0.33674562 | 6.3300606 | 2.07553129 | 0.04109947 | 0.99976948 | -4.2999955 | 2.04252509 |
| TCEAL9 | 0.43988658 | 5.79325382 | 2.07551646 | 0.04125587 | 0.99976948 | -4.3014283 | 2.04094929 |
| NDUFA3P4 | -1.54364 | -3.7417162 | -2.0714632 | 0.04144677 | 0.99976948 | -4.2854088 | -2.0390327 |
| MPP7 | -0.3526814 | 2.10948851 | -2.0724933 | 0.04145431 | 0.99976948 | -4.2891234 | -2.0389571 |
| RNA5SP379 | 1.75932203 | -5.7654702 | 2.06799116 | 0.04152772 | 0.99976948 | -4.2651385 | 2.03822217 |
| MATR3P3 | -1.8100178 | -3.7527829 | -2.0680471 | 0.04164289 | 0.99976948 | -4.288842 | -2.0370714 |
| SWSAP1 | -0.5658849 | 0.04208827 | -2.0686258 | 0.04165709 | 0.99976948 | -4.3143711 | -2.0369297 |
| LINC02940 | -1.5488082 | -3.5563269 | -2.0673759 | 0.04179243 | 0.99976948 | -4.2896292 | -2.0355812 |
| MAB21L4 | -1.3985677 | -5.6443375 | -2.0678508 | 0.0418141 | 0.99976948 | -4.3510972 | -2.0353656 |
| CCNB1IP1 | 0.26707851 | 5.35818169 | 2.06464381 | 0.04218353 | 0.99976948 | -4.2920447 | 2.03170498 |
| RPS3P3 | 0.74849997 | -0.8151088 | 2.06429724 | 0.04219848 | 0.99976948 | -4.2590291 | 2.03155741 |
| ZNF879 | -0.2425354 | 3.16101777 | -2.0637829 | 0.04225645 | 0.99976948 | -4.3041852 | -2.0309857 |
| LINC00235 | -0.3947043 | 1.24637977 | -2.0628901 | 0.04228697 | 0.99976948 | -4.2942732 | -2.0306848 |
| NXPE3 | 0.29978796 | 5.89559754 | 2.06390355 | 0.04229162 | 0.99976948 | -4.3011002 | 2.03063903 |
| KARS1P2 | 1.80640023 | -5.8690908 | 2.06093127 | 0.04241695 | 0.99976948 | -4.2742389 | 2.02940603 |
| RNA5SP37 | -1.470351 | -3.4694554 | -2.065862 | 0.04256091 | 0.99976948 | -4.3168593 | -2.0279936 |
| CACNG5 | 1.22953738 | 5.04580976 | 2.05789277 | 0.04274813 | 0.99976948 | -4.304306 | 2.02616259 |
| CACNG8 | -0.4163191 | 6.87288782 | -2.0589589 | 0.04278902 | 0.99976948 | -4.3029079 | -2.0257635 |
| G0S2 | -1.7393146 | -2.538362 | -2.0524435 | 0.04328537 | 0.99976948 | -4.3430445 | -2.0209459 |
| ERI3-IT1 | -1.0889086 | -1.8765915 | -2.0530392 | 0.0433092 | 0.99976948 | -4.3499853 | -2.0207157 |
| KRT18P25 | -1.6083348 | -6.4526048 | -2.05172 | 0.04340694 | 0.99976948 | -4.3001381 | -2.019773 |
| SMCO2 | -1.2745946 | -2.8601102 | -2.0499724 | 0.0435396 | 0.99976948 | -4.3356716 | -2.0184963 |
| WNT10A | 1.3288901 | -0.9747805 | 2.04965293 | 0.04360405 | 0.99976948 | -4.3485962 | 2.01787726 |
| MIR3127 | -1.8387416 | -5.7346901 | -2.0467125 | 0.04365246 | 0.99976948 | -4.3072065 | -2.0174127 |
| GALK1 | 0.36069817 | 3.98345546 | 2.04962232 | 0.04370765 | 0.99976948 | -4.3099347 | 2.01688375 |
| ELFN1-AS1 | 1.73800353 | -5.4989671 | 2.0507408 | 0.04376594 | 0.99976948 | -4.2788118 | 2.0163256 |
| XPOTP1 | -1.2662489 | -3.0745037 | -2.046289 | 0.04378076 | 0.99976948 | -4.3083125 | -2.0161838 |
| MED28P7 | 1.45727336 | -5.4131587 | 2.04409628 | 0.0439863 | 0.99976948 | -4.23946 | 2.01422135 |
| RNU5D-1 | -1.7725048 | -6.1013404 | -2.0423038 | 0.0441103 | 0.99976948 | -4.3230195 | -2.0130412 |
| CALHM4 | -2.0266716 | -5.7107223 | -2.0448424 | 0.04448892 | 0.99976948 | -4.315139 | -2.0094548 |
| TBX5-AS1 | -1.4386528 | -4.5816876 | -2.039543 | 0.04463428 | 0.99976948 | -4.3110943 | -2.0080848 |
| LINC02796 | -1.7246385 | -4.8719213 | -2.0389632 | 0.04467457 | 0.99976948 | -4.3367211 | -2.0077057 |
| EHF | 1.67530167 | -1.8298502 | 2.03937539 | 0.04477414 | 0.99976948 | -4.364071 | 2.00677021 |
| ANKRD31 | -0.5342414 | -0.3637235 | -2.0375204 | 0.04479693 | 0.99976948 | -4.3001793 | -2.0065563 |
| CSRP3-AS1 | 1.40596826 | -5.8482798 | 2.03866043 | 0.04499309 | 0.99976948 | -4.2610308 | 2.00471908 |
| CCT8L1P | -1.8116848 | -3.9391197 | -2.0343156 | 0.04503721 | 0.99976948 | -4.3555542 | -2.0043068 |
| ALLC | -2.0253233 | -3.56839 | -2.031278 | 0.04522063 | 0.99976948 | -4.3407601 | -2.0025963 |
| F5 | 1.02644365 | -0.1930489 | 2.03352671 | 0.04522286 | 0.99976948 | -4.2890174 | 2.00257559 |
| DNAJB12P1 | -1.515363 | -4.3861012 | -2.038308 | 0.04525399 | 0.99976948 | -4.3181874 | -2.0022859 |
| SLC28A3-AS1 | -1.5697439 | -5.9775168 | -2.0315308 | 0.0453274 | 0.99976948 | -4.3140557 | -2.0016034 |
| ASAH2 | -0.3729568 | 0.82634485 | -2.0333224 | 0.04533372 | 0.99976948 | -4.3120879 | -2.0015447 |
| RNA5SP108 | 1.57526366 | -4.7692802 | 2.02848314 | 0.04548381 | 0.99976948 | -4.2540735 | 2.00015237 |
| SDHAF4 | 0.24809243 | 3.82390274 | 2.03021616 | 0.04562533 | 0.99976948 | -4.3086254 | 1.99884311 |
| AK3 | 0.23872921 | 6.49645368 | 2.02926409 | 0.04579314 | 0.99976948 | -4.3178099 | 1.99729501 |
| ALOX5AP | -1.8401819 | -5.493297 | -2.0259272 | 0.04589733 | 0.99976948 | -4.3143956 | -1.9963363 |
| ITPRIPL1 | 1.08848326 | 0.3681032 | 2.02584345 | 0.04605631 | 0.99976948 | -4.2933505 | 1.99487686 |
| ETF1 | 0.20498544 | 6.73548473 | 2.02580093 | 0.0461476 | 0.99976948 | -4.3168445 | 1.99404073 |
| FES | 0.70470916 | -0.4701754 | 2.02451272 | 0.04631594 | 0.99976948 | -4.3260609 | 1.99250258 |
| CHCT1 | -1.9013814 | -5.2814309 | -2.0165924 | 0.04688007 | 0.99976948 | -4.3158849 | -1.9873821 |
| PCGF6 | 0.26540938 | 3.55853282 | 2.0179664 | 0.04694088 | 0.99976948 | -4.3216767 | 1.98683325 |
| C14orf119 | 0.2701286 | 4.41924322 | 2.01657322 | 0.04706054 | 0.99976948 | -4.3261679 | 1.98575491 |
| DNM1P41 | 1.43330873 | -4.3352102 | 2.01576811 | 0.04707345 | 0.99976948 | -4.2794563 | 1.98563877 |
| HTATIP2 | 1.04902048 | 0.49475154 | 2.01143516 | 0.04765129 | 0.99976948 | -4.3937475 | 1.98046509 |
| PLAAT2 | 1.54291572 | -3.2018614 | 2.00905913 | 0.04793115 | 0.99976948 | -4.3417678 | 1.9779783 |
| PDE11A-AS1 | 1.42108257 | -4.2038245 | 2.0076074 | 0.04802651 | 0.99976948 | -4.2894043 | 1.97713379 |
| THAP12P3 | 1.59460726 | -6.0408799 | 2.00597212 | 0.04802788 | 0.99976948 | -4.2826275 | 1.97712163 |
| IDI1 | 0.39090089 | 6.65841794 | 2.00960336 | 0.04807003 | 0.99976948 | -4.3178846 | 1.97674879 |
| KLF17 | -1.885632 | -5.7515857 | -2.007409 | 0.04808626 | 0.99976948 | -4.3519007 | -1.9766053 |
| OR52N4 | 1.88588425 | -4.6903232 | 2.0059465 | 0.04819249 | 0.99976948 | -4.3363503 | 1.97566717 |
| LRRC52 | 1.43745298 | -3.855901 | 2.00478708 | 0.04824295 | 0.99976948 | -4.2528375 | 1.97522212 |
| MYO7B | -1.7997118 | -3.7760282 | -2.0039901 | 0.04844737 | 0.99976948 | -4.3646274 | -1.9734232 |
| MLN | -1.6413715 | -4.2835121 | -2.0002457 | 0.04853916 | 0.99976948 | -4.3352681 | -1.9726175 |
| ABCC12 | 1.75847442 | -4.0638731 | 2.00307154 | 0.04854334 | 0.99976948 | -4.2851961 | 1.97258078 |
| MTND5P42 | 1.46127023 | -6.3925026 | 2.0002504 | 0.04863695 | 0.99976948 | -4.2614656 | 1.97176056 |
| BNIP3P16 | 1.75194093 | -4.4713287 | 1.99900856 | 0.0486417 | 0.99976948 | -4.2916463 | 1.97171896 |
| TSSK5P | -0.351217 | 2.40861258 | -2.0025327 | 0.04865806 | 0.99976948 | -4.3282565 | -1.9715757 |
| H2BK1 | 1.75689187 | -4.5847024 | 2.00040808 | 0.04869001 | 0.99976948 | -4.2920114 | 1.97129617 |
| MIR302CHG | 1.58000696 | -5.6592254 | 2.00044489 | 0.04869667 | 0.99976948 | -4.2955534 | 1.97123792 |
| RPS25P9 | 1.39552368 | -5.6573957 | 2.00466843 | 0.04890534 | 0.99976948 | -4.2898748 | 1.96941599 |
| ANAPC16 | 0.20078009 | 5.44287562 | 1.99979638 | 0.04894895 | 0.99976948 | -4.3278059 | 1.96903608 |
| MED15P8 | -1.4838163 | -6.1353796 | -1.9964701 | 0.04919299 | 0.99976948 | -4.3120825 | -1.9669152 |
| SNTB2 | 0.28715211 | 4.70277696 | 1.99753148 | 0.04923313 | 0.99976948 | -4.3398493 | 1.96656718 |
| RPS3AP40 | -1.7236265 | -5.6256661 | -1.9957052 | 0.04938189 | 0.99976948 | -4.3364681 | -1.9652796 |
| PARP4P2 | -1.9119173 | -5.9615864 | -1.9915668 | 0.04946799 | 0.99976948 | -4.3288442 | -1.9645358 |
| TRAPPC6A | 0.36981982 | 3.3700052 | 1.99495851 | 0.04955055 | 0.99976948 | -4.3223405 | 1.96382359 |
| GLYATL1P1 | -1.7416167 | -5.5250098 | -1.9899985 | 0.04993756 | 0.99976948 | -4.3379133 | -1.9604984 |
| CTDNEP1P1 | -0.8815064 | -1.7641686 | -1.9890067 | 0.04997746 | 0.99976948 | -4.358695 | -1.9601568 |

**Supplementary Table S12:** p-Values of Human Hallmark Signaling Pathways as Determined by Zenith

| **Hallmark Geneset** | **HNK (6hrs)** | **Psilocybin (6hrs)** | **LSD (6hrs)** | **DOI (6hrs)** | **HNK (24hrs)** | **Psilocybin (24hrs)** | **LSD (24hrs)** | **DOI (24hrs)** |
| --- | --- | --- | --- | --- | --- | --- | --- | --- |
| **TNFA_SIGNALING_VIA_NFKB** | 0.01 | 0.00 | 0.00 | 0.03 | 0.00 | 0.00 | 0.00 | 0.00 |
| **HYPOXIA** | 0.25 | 0.10 | 0.09 | 0.19 | 0.00 | 0.04 | 0.19 | 0.01 |
| **CHOLESTEROL_HOMEOSTASIS** | 0.31 | 0.02 | 0.01 | 0.15 | 0.02 | 0.18 | 0.71 | 0.00 |
| **MITOTIC_SPINDLE** | 0.19 | 0.52 | 0.46 | 0.04 | 0.08 | 0.11 | 0.07 | 0.96 |
| **WNT_BETA_CATENIN_SIGNALING** | 0.43 | 0.52 | 0.66 | 0.52 | 0.00 | 0.68 | 0.97 | 0.32 |
| **TGF_BETA_SIGNALING** | 0.66 | 0.12 | 0.32 | 0.73 | 0.00 | 0.07 | 0.53 | 0.04 |
| **IL6_JAK_STAT3_SIGNALING** | 0.25 | 0.02 | 0.06 | 0.20 | 0.00 | 0.00 | 0.00 | 0.00 |
| **DNA_REPAIR** | 0.00 | 0.02 | 0.02 | 0.01 | 0.11 | 0.24 | 0.99 | 0.11 |
| **G2M_CHECKPOINT** | 0.69 | 0.21 | 0.28 | 0.73 | 0.02 | 0.02 | 0.00 | 0.99 |
| **APOPTOSIS** | 0.31 | 0.02 | 0.05 | 0.11 | 0.00 | 0.01 | 0.06 | 0.01 |
| **NOTCH_SIGNALING** | 0.43 | 0.26 | 0.32 | 0.52 | 0.02 | 0.78 | 0.57 | 0.40 |
| **ADIPOGENESIS** | 0.02 | 0.02 | 0.06 | 0.01 | 0.01 | 0.00 | 0.16 | 0.00 |
| **ESTROGEN_RESPONSE_EARLY** | 0.69 | 0.17 | 0.15 | 0.63 | 0.00 | 0.05 | 0.18 | 0.00 |
| **ESTROGEN_RESPONSE_LATE** | 0.41 | 0.16 | 0.20 | 0.36 | 0.01 | 0.12 | 0.43 | 0.01 |
| **ANDROGEN_RESPONSE** | 0.43 | 0.10 | 0.20 | 0.36 | 0.01 | 0.14 | 0.73 | 0.00 |
| **MYOGENESIS** | 0.74 | 0.63 | 0.67 | 0.58 | 0.13 | 0.25 | 0.67 | 0.48 |
| **PROTEIN_SECRETION** | 0.94 | 0.17 | 0.28 | 0.17 | 0.00 | 0.00 | 0.02 | 0.00 |
| **INTERFERON_ALPHA_RESPONSE** | 0.44 | 0.00 | 0.01 | 0.07 | 0.00 | 0.00 | 0.00 | 0.00 |
| **INTERFERON_GAMMA_RESPONSE** | 0.50 | 0.00 | 0.02 | 0.10 | 0.00 | 0.00 | 0.00 | 0.00 |
| **APICAL_JUNCTION** | 0.69 | 0.52 | 0.88 | 0.84 | 0.08 | 0.40 | 0.76 | 0.74 |
| **APICAL_SURFACE** | 0.46 | 0.10 | 0.20 | 0.70 | 0.02 | 0.34 | 0.33 | 0.13 |
| **HEDGEHOG_SIGNALING** | 0.24 | 0.31 | 0.96 | 0.11 | 0.44 | 0.02 | 0.02 | 0.04 |
| **COMPLEMENT** | 0.25 | 0.02 | 0.03 | 0.10 | 0.00 | 0.00 | 0.00 | 0.01 |
| **UNFOLDED_PROTEIN_RESPONSE** | 0.39 | 0.53 | 0.41 | 0.27 | 0.00 | 0.26 | 0.37 | 0.01 |
| **PI3K_AKT_MTOR_SIGNALING** | 0.40 | 0.08 | 0.32 | 0.34 | 0.16 | 0.32 | 0.81 | 0.24 |
| **MTORC1_SIGNALING** | 0.02 | 0.02 | 0.01 | 0.01 | 0.00 | 0.06 | 0.34 | 0.00 |
| **E2F_TARGETS** | 0.19 | 0.19 | 0.09 | 0.43 | 0.02 | 0.11 | 0.02 | 0.46 |
| **MYC_TARGETS_V1** | 0.00 | 0.00 | 0.00 | 0.00 | 0.00 | 0.33 | 0.10 | 0.00 |
| **MYC_TARGETS_V2** | 0.00 | 0.06 | 0.01 | 0.04 | 0.01 | 0.08 | 0.67 | 0.00 |
| **EPITHELIAL_MESENCHYMAL_TRANSITION** | 0.31 | 0.02 | 0.01 | 0.16 | 0.00 | 0.00 | 0.00 | 0.00 |
| **INFLAMMATORY_RESPONSE** | 0.19 | 0.01 | 0.01 | 0.12 | 0.00 | 0.00 | 0.00 | 0.00 |
| **XENOBIOTIC_METABOLISM** | 0.39 | 0.15 | 0.41 | 0.17 | 0.09 | 0.02 | 0.05 | 0.03 |
| **FATTY_ACID_METABOLISM** | 0.24 | 0.15 | 0.34 | 0.04 | 0.28 | 0.12 | 0.53 | 0.04 |
| **OXIDATIVE_PHOSPHORYLATION** | 0.00 | 0.00 | 0.01 | 0.00 | 0.34 | 0.04 | 0.46 | 0.01 |
| **GLYCOLYSIS** | 0.13 | 0.07 | 0.11 | 0.17 | 0.00 | 0.02 | 0.32 | 0.00 |
| **REACTIVE_OXYGEN_SPECIES_PATHWAY** | 0.00 | 0.01 | 0.01 | 0.01 | 0.01 | 0.00 | 0.02 | 0.00 |
| **P53_PATHWAY** | 0.29 | 0.17 | 0.28 | 0.10 | 0.00 | 0.22 | 0.34 | 0.11 |
| **UV_RESPONSE_UP** | 0.02 | 0.12 | 0.08 | 0.01 | 0.00 | 0.11 | 0.34 | 0.02 |
| **UV_RESPONSE_DN** | 0.19 | 0.52 | 0.41 | 0.37 | 0.00 | 0.68 | 0.73 | 0.12 |
| **ANGIOGENESIS** | 0.94 | 0.53 | 0.67 | 0.87 | 0.01 | 0.17 | 0.34 | 0.32 |
| **HEME_METABOLISM** | 0.68 | 0.55 | 0.67 | 0.50 | 0.01 | 0.22 | 0.20 | 0.06 |
| **COAGULATION** | 0.19 | 0.12 | 0.20 | 0.37 | 0.00 | 0.00 | 0.00 | 0.01 |
| **IL2_STAT5_SIGNALING** | 0.25 | 0.04 | 0.20 | 0.32 | 0.00 | 0.00 | 0.00 | 0.01 |
| **BILE_ACID_METABOLISM** | 0.66 | 0.54 | 0.88 | 0.73 | 0.98 | 0.74 | 0.57 | 0.21 |
| **PEROXISOME** | 0.52 | 0.24 | 0.42 | 0.61 | 0.32 | 0.25 | 0.57 | 0.11 |
| **ALLOGRAFT_REJECTION** | 0.43 | 0.02 | 0.05 | 0.17 | 0.00 | 0.00 | 0.00 | 0.01 |
| **SPERMATOGENESIS** | 0.69 | 0.53 | 0.67 | 0.92 | 0.58 | 0.68 | 0.33 | 0.96 |
| **KRAS_SIGNALING_UP** | 0.78 | 0.35 | 0.32 | 0.49 | 0.01 | 0.06 | 0.33 | 0.27 |
| **KRAS_SIGNALING_DN** | 0.64 | 0.52 | 0.83 | 0.78 | 0.89 | 0.28 | 0.38 | 0.42 |
| **PANCREAS_BETA_CELLS** | 0.94 | 0.95 | 0.96 | 0.32 | 0.38 | 0.19 | 0.06 | 0.42 |

**Supplementary Table S13:** Top 15 Marker Genes by Cosine Similarity (COSG) for Single Cell Clusters

| **NPC_1** | **NPC_2** | **NPC_gl** | **IN_Early_1** | **IN_Early_2** | **IN_Early_3** | **IN_Mid_1** | **IN_Mid_2** | **IN_Mature** | **In_SST** | **EX_Mid_1** | **EX_Mid_2** | **EX_Mature_1** | **EX_Mature_2** |
| --- | --- | --- | --- | --- | --- | --- | --- | --- | --- | --- | --- | --- | --- |
| KIF18B | PAX7 | APOE | IL1RAPL2 | AL157895.1 | AP005230.1 | SKOR2 | SIX3 | LINC02300 | SST | SLC17A8 | CHRM3-AS2 | MC4R | LINC02027 |
| DLGAP5 | PLP1 | ANXA1 | Z96074.1 | CACNG3 | AP005262.2 | ALKAL2 | LINC00581 | MAFA | SIX2 | CYP26A1 | AC007319.1 | CALB2 | AC108690.1 |
| RRM2 | AC007344.1 | AQP4 | PPP1R1B | PRPH | CRISPLD2 | LINC01630 | ZNF503 | NPFFR1 | GRIN2A | KCNG3 | AC005064.1 | GPR22 | PDGFB |
| MKI67 | GSX1 | CFAP126 | PLSCR5 | AC005229.5 | LINC00609 | CA8 | ZNF503-AS2 | AC114316.1 | AC074096.1 | COL23A1 | AC096747.1 | FGF10 | BDNF |
| KIFC1 | BTG2 | MYOF | MYO3B | ROBO4 | CHST8 | GPR149 | NRGN | CTXN3 | CASZ1 | LMX1A | AC098617.1 | KC877982.1 | ANKRD30BL |
| CKAP2L | MFNG | IFITM2 | TSHZ1 | SLC6A5 | ARSF | IKZF1 | PDE1C | AC010247.2 | AC076968.2 | LYPD6 | AC090579.1 | VWC2 | OBI1-AS1 |
| CDCA2 | AC007402.1 | PLAAT4 | SSTR2 | CMTM7 | ECEL1 | AC106881.1 | TMTC1 | ONECUT1 | DIRAS3 | ADCY8 | LINC00290 | STC1 | LINC02742 |
| TOP2A | DEC1 | AGT | TNMD | ROBO3 | AC132153.1 | LHFPL2 | AC138627.1 | AC110296.1 | LINC01828 | IRX4 | AC104803.1 | GABRQ | ACTG2 |
| HJURP | EGFR | GFAP | PRSS12 | AC006974.2 | NTNG2 | HS6ST3 | AC022433.1 | FOXD3-AS1 | TRIM67 | GLYATL1 | ZPBP | ACTA1 | NPB |
| ASPM | AL139246.5 | TCTEX1D1 | ELMO1 | PAX2 | MSC-AS1 | SLC35F3 | AC114689.3 | LINC00922 | AC117461.1 | MAB21L1 | AC068931.1 | TH | TAC1 |
| KIF2C | SOX3 | S100A11 | LURAP1L | AP000866.1 | NFIA-AS2 | MCTP1 | ADRA2A | SMIM32 | AP003464.1 | SGCZ | AL354771.1 | ADCYAP1 | SNCG |
| SPC25 | DHRS3 | AL512452.1 | NEUROD2 | BHLHE22 | ERVMER61-1 | AC092422.1 | DACT1 | AL356295.1 | AC006145.1 | COL14A1 | CDH18-AS1 | CALCR | GABRB1 |
| AURKB | MYCL | IFITM3 | LHFPL6 | NHLH1 | DPF3 | ISL1 | AC004158.1 | ST8SIA6 | ALK | ASIC4 | AL354949.1 | AL031073.2 | AL157778.1 |
| KIF20A | NPPA | PTGDS | UNC5C | LBX1 | LMO2 | UNC5C | DPP6 | AC124854.1 | ST3GAL1 | SPP1 | AC073571.1 | TTC29 | MXRA5 |
| PIMREG | HES5 | SAMD9L | SLC27A6 | NTRK1 | RPS6KA1 | PCP4 | MIR137HG | ADARB2-AS1 | PAPPA2 | AC092114.1 | TENM3-AS1 | ARHGAP36 | HTR2C |

**Supplementary Table S14. Cell type proportion testing across treatment conditions.**

| **Cell Type** | **BaselineProp** | **PropMean.Control** | **PropMean.HNK** | **PropMean.LSD** | **PropMean.Psilocybin** | **Fstatistic** | **P.Value** | **FDR** |
| --- | --- | --- | --- | --- | --- | --- | --- | --- |
| NPC_2 | 0.069 | 0.068 | 0.066 | 0.093 | 0.056 | 0.641 | 0.619 | 0.995 |
| IN_Early_2 | 0.074 | 0.069 | 0.070 | 0.105 | 0.062 | 0.500 | 0.698 | 0.995 |
| EX_Mid_2 | 0.056 | 0.048 | 0.063 | 0.048 | 0.065 | 0.435 | 0.737 | 0.995 |
| NPC_gl | 0.053 | 0.048 | 0.045 | 0.082 | 0.053 | 0.353 | 0.790 | 0.995 |
| IN_Mid_2 | 0.156 | 0.168 | 0.173 | 0.065 | 0.178 | 0.339 | 0.799 | 0.995 |
| IN_Early_1 | 0.110 | 0.115 | 0.121 | 0.069 | 0.113 | 0.268 | 0.846 | 0.995 |
| IN_Mid_1 | 0.069 | 0.075 | 0.068 | 0.056 | 0.073 | 0.212 | 0.884 | 0.995 |
| EX_Mature_1 | 0.076 | 0.066 | 0.069 | 0.133 | 0.060 | 0.198 | 0.892 | 0.995 |
| EX_Mid_1 | 0.056 | 0.051 | 0.049 | 0.077 | 0.057 | 0.182 | 0.904 | 0.995 |
| EX_Mature_2 | 0.039 | 0.040 | 0.035 | 0.042 | 0.044 | 0.182 | 0.905 | 0.995 |
| IN_Early_3 | 0.071 | 0.077 | 0.068 | 0.057 | 0.074 | 0.155 | 0.922 | 0.995 |
| IN_Mature | 0.058 | 0.062 | 0.054 | 0.061 | 0.060 | 0.049 | 0.984 | 0.995 |
| NPC_1 | 0.062 | 0.065 | 0.063 | 0.059 | 0.057 | 0.037 | 0.989 | 0.995 |
| In_SST | 0.051 | 0.049 | 0.054 | 0.052 | 0.048 | 0.024 | 0.995 | 0.995 |

Proportional representation of each identified cell type cluster across treatment conditions, tested using *propeller* [1]. Baseline proportion reflects the overall proportion of each cell type across the full integrated dataset. Mean proportions are shown for each treatment condition. F-statistics and false discovery rate (FDR)-corrected p-values are from a linear model framework with donor treated as a random effect. No cell type showed significant differences in proportional representation across conditions (all FDRs>0.99), supporting the validity of cross-condition transcriptional comparisons within cell type clusters. HNK: (2R,6R)-hydroxynorketamine; LSD: lysergic acid diethylamide.

**Supplementary Table S15. Western Blot Significance Table,** representing all proteins tested that were found significant for treatment, time, or interactions. Post-hoc analysis was conducted using Tukey’s Honestly Significant Difference (HSD) test. P-values are presented when significantly different from vehicle control.

|  | | | | **Post-hoc analysis (vs. vehicle control)** | | | | | | | |
| --- | --- | --- | --- | --- | --- | --- | --- | --- | --- | --- | --- |
|  |  |  |  | **6 hours** | | | | **24 hours** | | | |
| **Protein** | **Treatment** | **Time** | **Interaction** | **DOI** | **HNK** | **LSD** | **Psilocybin** | **DOI** | **HNK** | **LSD** | **Psilocybin** |
| ERK | F(4,184) = 2.65, p = 0.035 | NS | F(4,184) = 4.82, p = 0.001 | NS | NS | NS | NS | p = 0.004 | p = 0.047 | NS | NS |
| p-eEF2 | F(4,185) = 3.55, p = 0.008 | NS | F(4,185) = 5.41, p <0.001 | NS | NS | NS | NS | NS | p = 0.038 | NS | NS |
| eEF2 | NS | NS | F(4, 184) = 3.98, p = 0.004 | NS | NS | NS | NS | NS | p = 0.017 | NS | NS |
| p-eiF4E | NS | NS | F(4, 184) = 3.31, p = 0.012 | NS | NS | NS | NS | NS | p = 0.04 | NS | NS |
| eIF4E | F(4,184) = 4.2, p = 0.003 | NS | NS | NS | NS | NS | NS | NS | p = 0.002 | NS | NS |
| 4EBP1 | F(4,185) = 3.56, p = 0.008 | NS | F(4,185) = 4.28, p = 0.002 | NS | NS | NS | NS | NS | NS | NS | NS |
| PSD-95 | F(4,184) = 3.53, p = 0.008 | NS | F(4,184) = 6.42, p < 0.001 | NS | NS | NS | NS | NS | p = 0.014 | NS | NS |
| Synaptotagmin | NS | NS | F(4,184) = 3.72, p = 0.006 | NS | NS | NS | NS | NS | NS | NS | NS |
| Synapsin I | NS | NS | F(4,184) = 2.62, p = 0.036 | NS | NS | NS | NS | NS | NS | NS | NS |

HNK: (2*R*,6*R*)-hydroxynorketamine; DOI: 2,5-Dimethoxy-4-iodoamphetamine; LSD: lysergic acid diethylamide; ERK: extracellular signal-regulated kinase; eEF2: eukaryotic translation elongation factor 2; eIF4E: eukaryotic translation initiation factor 4E; 4EBP1: eukaryotic translation initiation factor 4E-binding protein 1; PSD-95: postsynaptic density-95 protein.

**Supplementary References**

1.	Phipson B, Sim CB, Porrello ER, Hewitt AW, Powell J, Oshlack A. *propeller*: testing for differences in cell type proportions in single cell data. *Bioinformatics* 2022; **38:** 4720-4726.
